# Epimutation profiling in Beckwith-Wiedemann syndrome: relationship with assisted reproductive technology

**DOI:** 10.1186/1868-7083-5-23

**Published:** 2013-12-10

**Authors:** Louise Tee, Derek HK Lim, Renuka P Dias, Marie-Odile Baudement, Amy A Slater, Gail Kirby, Tom Hancocks, Helen Stewart, Carol Hardy, Fiona Macdonald, Eamonn R Maher

**Affiliations:** 1 Centre for Rare Diseases and Personalised Medicine, School of Clinical and Experimental Medicine, College of Medical and Dental Sciences, University of Birmingham, Edgbaston, Birmingham B15 2TT, UK; 2 West Midlands Regional Genetics Service, Birmingham Women’s Hospital, Edgbaston, Birmingham B15 2TG, UK; 3 Department of Endocrinology, Birmingham Children’s Hospital, Steelhouse Lane, Birmingham B4 6NH, UK; 4 Department of Clinical Genetics, Oxford Regional Genetics Service, Churchill Hospital, Oxford, UK; 5 Department of Medical Genetics, University of Cambridge, Cambridge CB2 2QQ, UK

**Keywords:** Beckwith-Wiedemann syndrome, Assisted reproductive technology, Imprinting, Genetics

## Abstract

**Background:**

Beckwith-Wiedemann syndrome (BWS) is a congenital overgrowth disorder associated with abnormalities in 11p15.5 imprinted genes. The most common cause is loss of methylation (epimutation) at the imprinting control centre 2 (IC2/KvDMR1). Most IC2 epimutations occur sporadically but an association with conception after assisted reproductive technologies (ART) has been reported. A subgroup of IC2 epimutation cases also harbour epimutations at other imprinting centres (ICs) outside of 11p15.5. We have investigated the relationship between these multiple epimutation cases (ME+), history of ART and clinical phenotype in a cohort of 187 BWS IC2 epimutation patients.

**Results:**

Methylation analysis at *PLAGL1*, *MEST* and *IGF2R* ICs demonstrated an over-representation of patients with abnormally low methylation (8.5%, 12% and 6% respectively). At *IGF2R* some patients (2%) had gain of methylation but this was also detected in controls. Though there were no significant correlations between the methylation index (MIs) at the three ICs tested, a subset of patients appeared to be susceptible to multiple epimutations (ME+) and 21.2% of ME + patients had been conceived by ART compared to 4.5% (P = 0.0033) without additional epimutations. Methylation array profiling (Illumina Goldengate®) of patients and controls (excluding 11p15.5 loci) demonstrated significant differences between patients and controls. No significant associations were found between aspects of the BWS phenotype and individual epimutations but we describe a case presenting with a post-ART BWS-like phenotype in which molecular analysis demonstrated loss of paternal allele methylation at the 11p15.5 IC1 locus (IC1 regulates imprinting of IGF2 and H19). Loss of paternal allele methylation at the IC1 is the molecular finding associated with Silver-Russell syndrome whereas BWS is associated with gain of maternal allele methylation at IC1. Further analysis demonstrated epimutations at *PLAGL1* and *MEST* consistent with the hypothesis that the presence of multiple epimutations may be of clinical relevance.

**Conclusions:**

These findings suggest that the ME + subgroup of BWS patients are preferentially, but not exclusively, associated with a history of ART and that, though at present, there are no clear epigenotype-phenotype correlations for ME + BWS patients, non-11p15.5 IC epimutations can influence clinical phenotype.

## Background

Genomic imprinting is a form of epigenetic control of gene expression in which one allele of a gene is preferentially expressed according to the parent-of-origin of the allele [1]. Only a minority of human genes (approximately 100) are imprinted but many of those identified to date have been implicated in prenatal growth and development [2,3]. A notable feature of imprinted genes is that they tend to be found in clusters and not randomly distributed across the genome. A number of different mechanisms (for example, DNA methylation, chromatin modification and expression of large non-coding RNAs) have been implicated in the establishment and maintenance of genomic imprinting and a key role is played by imprinting control centres (ICs), which may regulate the imprinting of several genes [1]. ICs are often coincident with differentially methylated regions (DMRs) at which the methylation status of the CpG islands differ according to the parent-of-origin. Deletion or abnormal methylation (an epimutation) at these sites may cause abnormal imprinting within the imprinted gene cluster. For example, gain of methylation (GOM) at the normally unmethylated maternal allele of the chromosome 11p15.5 IC1 DMR is associated with bi-allelic expression of the paternally expressed growth promoter *IGF2* and is associated with the Beckwith-Wiedemann congenital overgrowth syndrome (BWS) [4]. In contrast, loss of methylation (LOM) of the paternal allele at the same IC is associated with loss of paternal allele *IGF2* expression and the phenotype of Silver-Russell syndrome (SRS), which is characterised by pre- and postnatal growth restriction [5].

Epimutations at the IC1 DMR occur in 5 to 10% of children with BWS but a more common cause of this disorder is loss of maternal allele methylation at a DMR (KvDMR1) associated with a second 11p15.5 IC (IC2). Thus, IC2 epimutations account for approximately 50% of all cases of BWS [6]. IC2 epimutations are associated with loss of expression of the imprinted maternally expressed growth suppressor *CDKN1C* (loss of function mutations in this gene can also cause BWS). Interestingly, a subset of BWS patients with IC2 epimutations also harbour epimutations at other imprinted gene cluster DMRs outside of 11p15.5 (for example, *PLAGL1* and *MEST* (*PEG1*) on chromosomes 6 and 7 respectively) [7,8]. The clinical consequences and aetiology of these multiple epimutations are mostly unclear, though in a minority of cases a rare genetic cause may be identified and in others there is an association with a history of conception by assisted reproductive technology (ART) [9-11]. To further investigate the potential significance of multiple epimutation epigenotypes we undertook methylation assays and epigenotype-phenotype correlations in patients with BWS and IC2 epimutations, paying particular attention to possible associations between epimutations and ART.

## Methods

### Patients

Children and adults (n = 187) with a clinical and molecular diagnosis of BWS were studied for methylation status at one or more imprinted loci outside 11p15.5 (*PLAGL1, MEST* and *IGF2R* DMRs). Clinical information was collected using a standard questionnaire. Fourteen patients had been conceived after ART by *in vitro* fertilization (IVF) or intracytoplasmic sperm injection (ICSI). Written informed consent was obtained and ethical approval was obtained from South Birmingham Research Ethics committee.

### Molecular analysis

DNA was extracted from peripheral blood lymphocytes by standard procedures. Previously all BWS patients had been shown to have loss of methylation at KvDMR1 (11p15.5 IC2) without evidence of uniparental disomy or copy number abnormalities by methylation-specific-multiplex ligation-dependent probe amplification for genetic disease and genomic imprinting research (MS-MLPA) analysis (MRC-Holland, Amsterdam, The Netherlands). Anonymised DNA samples from healthy individuals without an imprinting disorder were used as controls for the methylation studies. Grandparental origin of the maternally transmitted IC2 region was established by genotyping microsatellite polymorphisms (*TH*, *D11S1318* and *D11S1984*) as described previously [12].

#### Methylation at PLAG1, MEST and IGF2R *DMRs*

For differential digestion of DNA for methylation analysis, 0.5 ug of genomic DNA was treated using the Epitect Methyl DNA Restriction Kit (SA Biosciences, Qiagen Ltd, West Sussex, UK 335451) as per instructions. Predesigned qPCR assays for *MEST* (SA Biosciences, MePH10851) and *PLAGL1* (SA Biosciences, MePH285522-1A) were used. For the CpG island in intron 2 of *IGF2R*, a custom quantitative PCR (qPCR) assay was designed by SA Biosciences. Real-time PCR was performed on the BioRad iCycler thermal cycler using the SensiMix Sybr with fluorescein kit (Bioline, London, UK, QT615-02) conditions as per SA Biosciences protocol.

Data analysis was performed using software supplied on the SA Biosciences website, whereby control values obtained from the real-time PCRs were pasted into an Excel®-based template. The template automatically calculated and reported the percentage of DNA hypermethylated and unmethylated fractions. To establish normal ranges and therefore, a level at which LOM could be assumed for each of the assays tested, a series of normal control samples were run. Methylation index (MI, percentage hypermethylation) values obtained were used for calculations. For *PLAGL1* DMR MI results were obtained for 20 control samples. The average MI was 47.33% and the range was 36.38 to 55.08% with SD of 4.91. Three times the SD from the average MI included all control values and set the lower threshold for LOM at 32.6%. For *MEST* DMR results were obtained for 11 control samples. The average MI was 47.83%, range was 32.54 to 55.48% and SD 7.73%. Two times the SD from the average MI included all control values and set the lower threshold for LOM at 32.37%. For *IGF2R* DMR results were obtained for 13 control samples. The median MI was 50.3% and the average MI was 57.05% with a range of 41.1 to 97.7%. D’Agostino-Pearson testing for normality of the distribution rejected normality due to an outlier value of 97.7%. Excluding the outlier, the median was 50.15%, mean 53.66% (range 41.1 to 74.99) and SD was 9.5%. Two times the SD from the mean MI set the lower threshold for LOM at 34.66%.

Limited amounts of available DNA meant that it was not possible to obtain a methylation index (MI) for each of the three tested loci in all patients. A value for the MI ≥1 locus was obtained for 187 patients but MI values at all three loci were obtained for only 150 samples (11 samples failed for *PLAGL1* MI (5 for both *PLAGL1* and *IGF2R* DMRs): 20 failed for *MEST* DMR (9 for both *MEST* and *IGF2R* DMRs) and 6 samples failed for *IGF2R* DMR only).

#### Illumina Goldengate methylation analysis

DNA samples of 0.5 μg were treated with sodium bisulphite using the EZ DNA methylation Gold kit (Zymo Research, Irvine, USA), and the bisulphite-treated DNA was applied to an Illumina bead array (10) using the Illumina Goldengate Methylation Cancer Panel (http://support.illumina.com/downloads/goldengate_methylation_cancer_panel_product_files.ilmn) (performed at the Wellcome Trust Centre for Human Genetics, University of Oxford, UK), which has been described and validated previously [13]. Hierarchical cluster analysis was performed using Illumina BeadStudio software (http://res.illumina.com/documents/products/datasheets/datasheet_beadstudio.pdf) with the Euclidean algorithm (see http://res.illumina.com/documents/products/technotes/technote_beadstudio_normalization.pdf) and used to create heatmaps and associated dendrograms.

## Results

### Methylation at PLAGL1, MEST and IGF2R DMRs in patients with BWS and 11p15.5 IC2 epimutations

**Figure 1:**
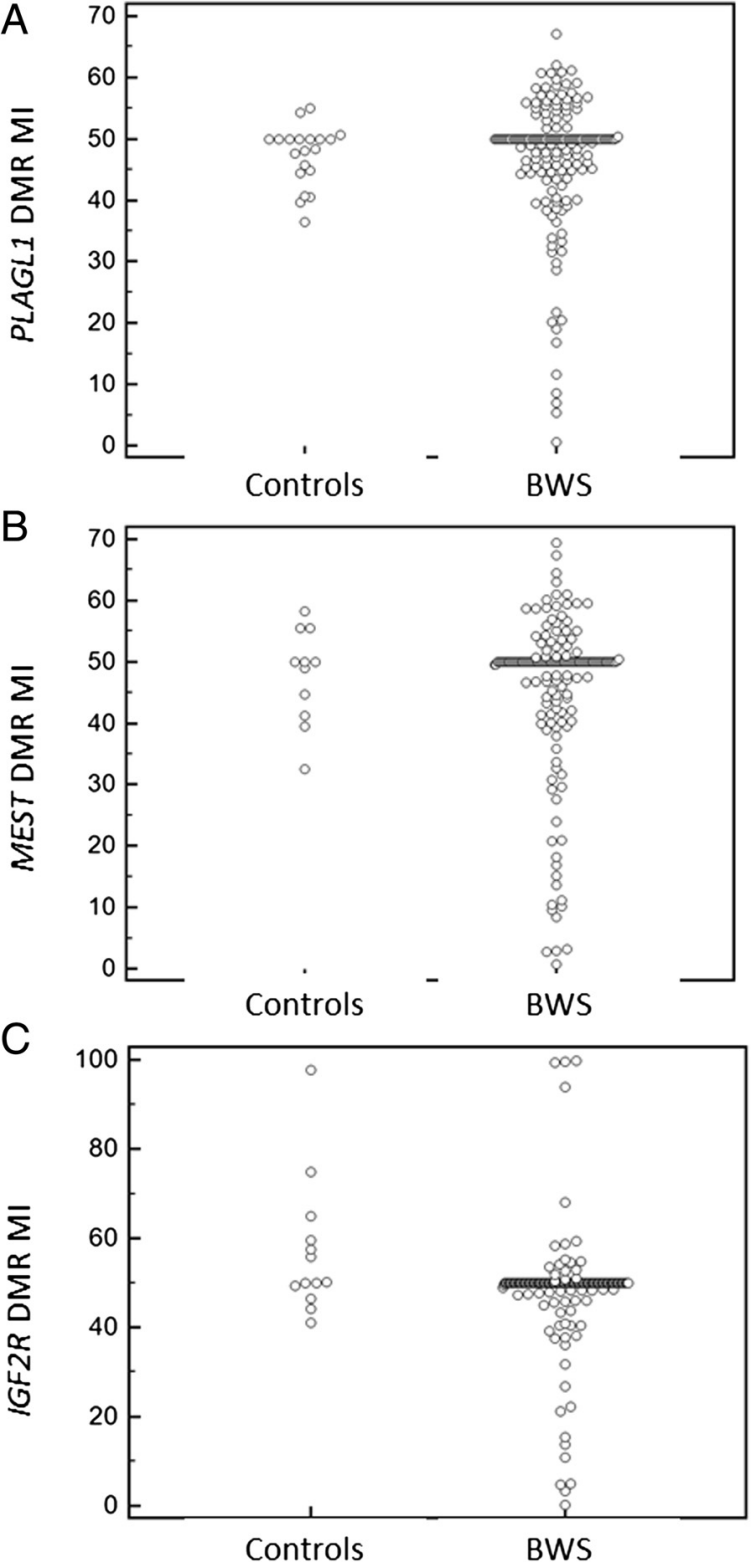
**Comparison of methylation indices in control samples and patients with Beckwith-Wiedemann syndrome (BWS) at *PLAGL1- *(n = 176 BWS patients) (A), *MEST- *(n = 167 BWS patients) (B), and *IGF2R*-associated DMRs (n = 167 BWS patients) (C).** For all three loci the distribution of MIs did not correspond to a normal distribution and there was an increased frequency of outliers with low methylation index in the BWS patient samples (D’Agostino-Pearson test for normal distribution rejected (*P* <0.0001) at *PLAGL1*, *MEST* and IGF2R).

Mean (+ SD) MI at the *PLAGL1* DMR was 47% ± 10.56 (median 50%, range 0.0055 to 67.13) in BWS patients with IC2 epimutations (n = 176). In comparison to normal controls (n = 20), there was an over-representation of low MIs in patient samples (Figure 1A) and the distribution of *PLAGL1* DMR MIs did not conform to a normal distribution (D’Agostino-Pearson test for normal distribution rejected at *P* <0.0001; coefficient of skewness, *P* <0.0001). Thus, 15 (8.5%) patients had an abnormally low MI (MI <33%) at the *PLAGL1* DMR (median 20.2%, range 0.6 to 32.6%).

Mean (+ SD) MI at the *MEST* (also known as *PEG1*) DMR was 46.4% + 12.54 (median 50%, range 0.0012 to 99.76) in BWS patients with IC2 epimutations (n = 167). In comparison to normal controls (n = 11), there was over-representation of low MIs in patient samples (Figure 1B) and the distribution of *MEST* DMR MIs did not conform to a normal distribution (D’Agostino-Pearson test for normal distribution rejected at *P* <0.0001) with significant evidence of kurtosis (coefficient of kurtosis 9.016; *P* <0.0001) (see Figure 1B). Twenty of the BWS patients (12%) who were successfully tested had an abnormally low MI (MI <31%) at the *MEST* DMR (median MI 14.3%, range 0.7 to 30.8%).

Mean (+ SD) MI at the *IGF2R* DMR was 45.7% + 12.98 (median 50%, range 0.0072 to 69.37) in BWS patients with IC2 epimutations (n = 167). *IGF2* DMR MI was measured in 13 control samples. The distribution of *IGF2R* DMR MIs did not conform to a normal distribution (D’Agostino-Pearson test for normal distribution rejected at *P* <0.0001; coefficient of skewness -1.815; *P* <0.0001) (see Figure 1C). However, unlike the *PLAGL1* and MEST DMR MIs, both low and high MIs were detected: 10 patients (6%) had loss of methylation (MI <31%) at the *IGF2R* DMR (median 12.3%, range 0.1 to 26.8%) and 4 patients (2%) had increased methylation (MI >90%) (median 99.5%, range 93.9 to 99.8%), although an individual with high methylation was also detected in control samples.

Though there were was no significant correlations between the MIs at the *PLAGL1*, *MEST* and *IGF2R* DMRs (Spearman rank correlation analysis), a subset of patients with BWS appeared to be susceptible to multiple epimutations. Thus, of the cases for whom data were available at all three non-11p15.5 IC DMRs, 81.7% did not have hypomethylation at the three tested loci, 12.4% had hypomethylation at one locus, 3.9% at two loci and 2% had hypomethylation at all three IC DMRs tested. Thus the frequency of hypomethylation at *MEST* and/or *IGF2R1* DMRs was higher in those with than those without *PLAGL1* DMR hypomethylation (41.6% versus 10.5%; *P* = 0.009). Similarly the frequency of hypomethylation at *PLAGL1* and/or *IGF2R1* DMRs was higher in those with than those without *MEST* DMR hypomethylation (55.6% versus 7.4%; *P* = 0.00003) and the frequency of hypomethylation at *PLAGL1* and/or *MEST* DMRs was higher in those with than those without *IGF2R* DMR hypomethylation (70% versus 13.3%; *P* = 0.00016).

### Multiple epimutation epigenotype and assisted reproductive technologies

Of 187 BWS patients tested for additional epimutations, 14 had been conceived by ART: 7 of 33 BWS patients (21.2%) with LOM at one or more of the tested DMRs (*PLAGL1*, *MEST* and *IGF2R*) had been conceived by IVF or ICSI compared to 7 of 154 (4.5%) (*P* = 0.0033) without evidence of LOM at one or more of the tested DMRs. Thus 50% (7 of 14) of BWS patients with an IC2 epimutation, who were conceived after ART, displayed a multiple epimutation epigenotype compared to 15% (26/173) of naturally conceived patients. Of the 14 ART cases, 9 were conceived by IVF and 5 by ICSI: 5 of 9 patients conceived by IVF and 2 of 5 patients conceived by ICSI had a multiple epimutation epigenotype (*P* = 1.0). None of the four BWS patients with increased methylation at the *IGF2R* DMR were conceived by IVF or ICSI and none demonstrated LOM at *PLAGL1* or *MEST* DMRs.

### ART and multiple epimutation genotype and clinical phenotype

The frequencies of specific clinical features among the cohort of individuals with BWS and IC2 epimutations were: macroglossia (94%), ear creases or pits (80%), neonatal hypoglycaemia (58%), facial naevus flammeus (54%), exomphalos (52%), umbilical hernia (34.8%), hemihypertrophy (28%), cardiovascular abnormality (18%), prenatal pre-eclampsia (15%), developmental delay (9%) and cleft palate (2%). Only one patient had developed an embryonal tumour (a hepatoblastoma). No significant associations were detected between the frequency of any of these specific clinical features and the presence of multiple epimutations (loss of methylation at *PLAGL1*, *MEST* or *IGF2R* DMRs).

Comparison of gestation-adjusted birth weight SD from mean in patients with (+1.4 ± 1.47 (mean ± SD); range -2.19 to +3.31) and without (+1.31 ± 1.32; -3.56 to +4.75) multiple epimutations demonstrated no significant difference (*t* = -0.26, *P* = 0.8). There were no significant correlations between mean gestation-adjusted birth weight SD and MI index at *PLAGL1* (Spearman’s coefficient of rank correlation (rho) = 0.084; *P* = 0.37), *MEST* (rho = -0.037; *P* = 0.7) and *IGF2R* (rho = -0.083; *P* = 0.38).

### Methylation profiling by Illumina Goldengate CpG methylation assay

**Figure 2:**
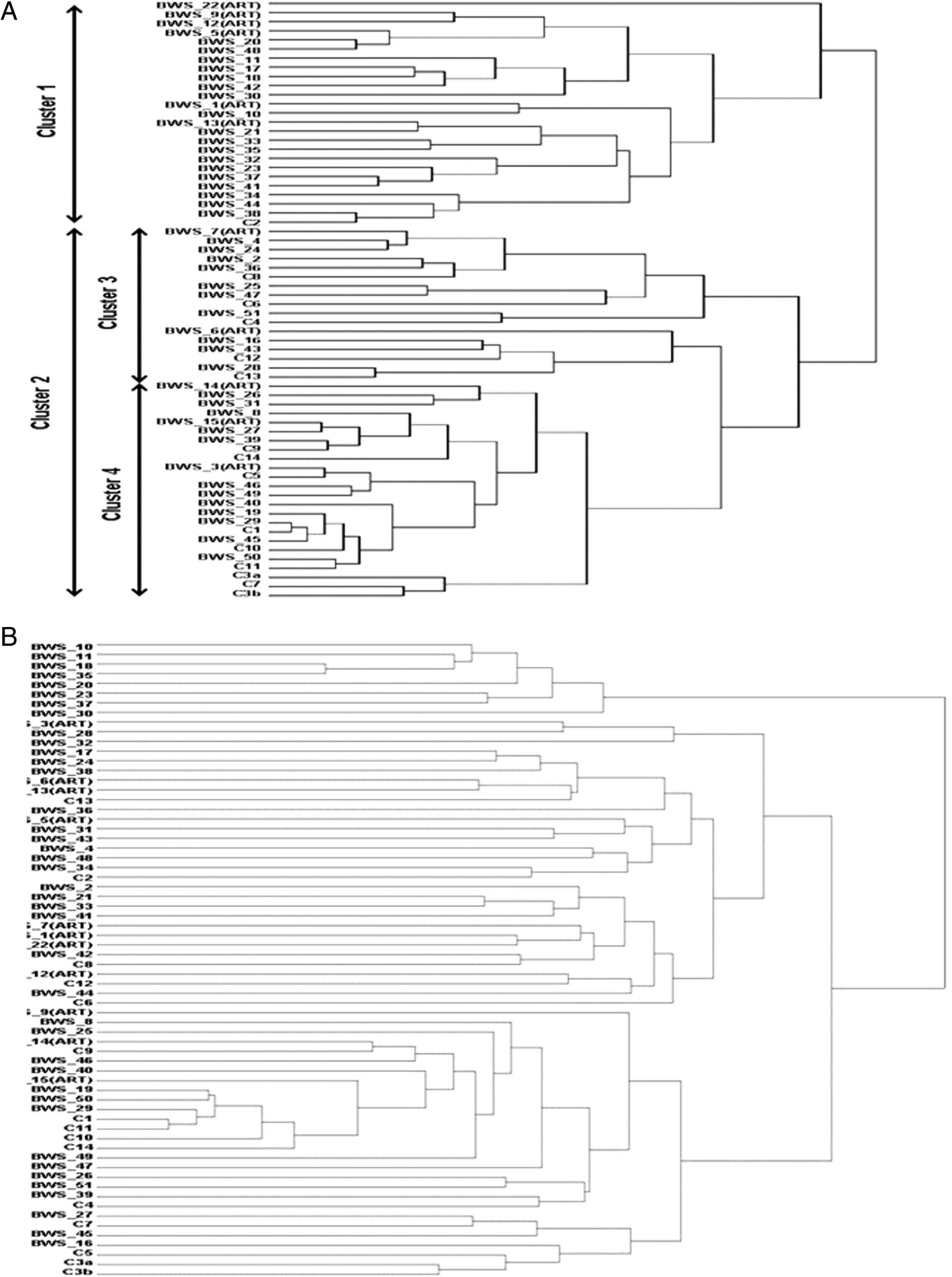
**Clustering dendrograms of Beckwith-Wiedemann syndrome (BWS) patients and controls (samples were assigned arbitrary numbers) according to methylation state of (A) all imprinted genes (excluding H19 and KvDMR1) and (B) all non-imprinted genes.** *BWS indicates samples from patients with BWS and C indicates control samples. Suffix (ART) denotes BWS patients conceived with ART. **(A)** Unsupervised analysis of cluster 1 versus cluster 2 showed significant grouping of controls versus BWS patients (*P* = 0.0054). No significant clustering was detected of (i) BWS patients conceived via ART compared to BWS patients conceived naturally (*P* = 0.3082) and (ii) controls versus BWS between cluster 3 and 4 (*P* = 0.52). **(B)** Analysis of methylation status of CpGs associated with non-imprinted genes showed significant clustering of controls versus BWS (*P* = 0.0402) but no significant clustering of BWS conceived by ART compared to BWS patients conceived naturally (*P* = 0.3308).

The Illumina GoldenGate Methylation Cancer Panel I array provides quantitative CpG methylation data at 1,505 individual CpG dinucleotides associated with 807 human genes. This platform was used to profile the peripheral blood methylation patterns of BWS patients and controls. We initially excluded 638 CpG sites from further analysis because (a) they were on the X chromosome genes or were in the 11p15.5-imprinted gene cluster and (b) the CpG methylation results were poorly replicated in controls (>10% methylation difference) (see Additional file 1: Table S1). Euclidean cluster analysis was performed for (a) all imprinted CpG sites that mapped outside 11p15.5 (n = 53) and (b) all non-imprinted CpG sites (n = 865). Analysis of the imprinted gene CpG methylation data for BWS patients and controls demonstrated two principal clusters (Figure 2A and Additional file 2: Figure S1). Cluster 1 contained 23 BWS patients and one control whereas cluster 2 contained 27 BWS patients and 15 controls (*P* = 0.0058). The four BWS patients whose methylation profile was most different to the control samples were all conceived by ART. However, of the 10 BWS patients who had been conceived by ART, 5 were in cluster 1 and 5 in cluster 2, and overall there was no significant association between ART and the non-11p15.5-imprinted gene methylation profile. Significant clustering of controls versus BWS was seen when non-imprinted CpG sites were analysed (*P* = 0.0402) (Figure 2B and Additional file 2: Figure S2) but there were no significant differences between ART-conceived and naturally conceived BWS patients at non-imprinted CpG sites (*P* = 0.3308). Details of the absolute MI for each CpG analysed for clustering analysis are given in Additional file 1: Table S1.

### Grandparental origin of IC2 epimutations

We investigated the grandpaternal origin of the maternally inherited IC2 region in individuals with loss of methylation at IC2. A bias in the grandpaternal origin would provide clues to the possible mechanism for loss of IC2 methylation. The grandparental origin of the maternal KvDMR1 allele that had lost the correct methylation imprint was determined in 10 patients by genotyping with closely linked microsatellite markers. In seven patients the maternally inherited IC2 allele had been derived from the maternal grandmother and in three patients from the maternal grandfather. There was no significant association between the grandparental origin and presence or absence of additional epimutations at *PLAGL1*, *MEST* or *IGF2R* DMRs (*P* = 1.0) or a history of IVF/ICSI (*P* = 1.0).

### IC1 hypomethylation in BWS: case report

The proband is a male child born at 41 weeks gestation following an ICSI conception. Birth weight was on the 25th centile and no neonatal hypoglycaemia was reported. However, at age 5 weeks he presented to hospital with a history of breathing problems with breath-holding spells and apnoea. Otolaryngological assessment identified severe macroglossia as contributing to his apnoea and he required a tracheostomy at age 2 months. A referral for clinical genetics assessment was made and clinical examination identified severe macroglossia, postnatal overgrowth (75th centile for weight and 91st centile for head circumference at age 16 weeks, and subsequently 91st centile for weight at age 2 years and 99th centile for weight, 95th centile for head circumference and just below 50th centile for height at age 4 years), ear lobe creases, facial naevus flammeus, umbilical hernia and discrepancy in leg size. No organomegaly was detected on ultrasound scans or normal echocardiogram. A clinical diagnosis of BWS was made and MS-MLPA analysis (MRC Holland) was performed to look for epimutations associated with BWS (IC2 (KvDMR1) loss of methylation or IC1 (H19-IGF2 DMR) hypermethylation). Unexpectedly, IC1 (H19-IGF2 DMR) hypomethylation was detected. No chromosomal copy number abnormalities were detected. In view of the discrepancy between the clinical phenotype of BWS and the molecular findings of SRS, additional methylation analysis was performed at the *PLAGL1* and *MEST* DMRs and loss of methylation was detected at both loci.

## Discussion

We profiled a large series of patients with BWS and an IC2/KvDMR1 epimutation to identify additional epimutations at DMRs outside of 11p15.5. We found that the multiple epimutation epigenotype (ME+) occurred in a subset of patients and was associated with a history of ART. However, the multiple epimutation epigenotype was not restricted to ART-conceived patients and was also found in a subset of non-ART BWS patients with IC2 epimutations. Imprinted gene cluster DMRs may act as boundary elements and/or regulate the expression of imprinted non-coding RNAs (for example, *H19, KCN1OT* and *Air*) and the methylation status of the DMR is critical to their normal function. For example, the insulating CTCF zinc finger protein normally binds to the unmethylated maternal allele of IC1 (*H19*) DMR on 11p1.5.5, enabling downstream enhancers to preferentially interact with the *H19* promoter. Methylation of the DMR on the paternal allele (containing the CTCF binding region) prevents CTCF binding and allows the enhancers to interact with the *IGF2* promoter instead [14,15]. Conversely, loss of IC1 methylation on the paternal allele (as seen in SRS) is associated with loss of paternal allele IGF2 expression [5].

Most cases of imprinting disorders associated with a DMR epimutation occur sporadically. In a few cases, apparent epimutations result from a deletion or mutation in the IC (that is, an *in cis* mechanism) and some cases can occur via *trans*-mechanism leading to abnormal DNA methylation, often at multiple imprinted loci. Thus, patients with mutations in *ZFP57* (which is involved in the establishment and maintenance of genomic imprinting) presenting with prenatal growth failure and transient neonatal diabetes mellitus (TNDM, OMIM 601410) showed loss of maternal methylation at the *PLAGL1* DMR and variable loss of maternal methylation at *GRB10*, *PEG3*, *NESPAS* and *MEST*[16]. Overall however, mutations in *ZFP57* and other trans-acting factors implicated in the establishment of genomic imprinting are rare causes of various imprinting disorder phenotypes [16-18]. In addition to genetic causes, environmental factors may be associated with epimutations in a subset of patients. Thus, we and others have found an excess of being conceived by ART among children with sporadic BWS [9,11]. Further studies have demonstrated that this finding reflects an association between post-ART BWS and IC2 epimutations (IC2 epimutations are found in >90% of post-ART BWS compared to approximately 50% of all cases) [10,19]. Though the relative risk of BWS (and possibly other imprinting disorders) is increased after ART, the absolute risk of having a child with an imprinting disorder for an individual ART-pregnancy appears to be small (<0.1%) [20-22]. Nevertheless, there is much interest in any association between ART and imprinting disorders as it would lend support to the hypothesis that congenital anomalies and growth restriction in ART-conceived children could be explained, at least in part, by epimutations [10].

Previously it was reported that about 25% of BWS patients with IC2 epimutations were also reported to have additional epimutations at non-11p15.5 IC DMRs [7,8]. Other imprinting disorders such as SRS and pseudohypoparathyroidism (PHP type 1B) have also shown an ME + epigenotype [23-26]. However, in general the aetiology and clinical significance of ME + cases has been unclear. Although in a previous study of 55 patients with BWS and IC2 epimutations we found an association between BWS ME + cases and a history of ART, such an association has not been detected consistently [26,27]. Possible reasons for these differences might include inadequately powered studies or differences in ART protocols or DNA methylation detection assays. We therefore undertook methylation profiling a larger series of BWS patients at three specific IC DMRs (*PLAGL1*, *MEST* and *IGF2R*) that have been commonly reported to show aberrant methylation in imprinting disorder patients with multiple epimutations.

Loss of maternal allele methylation at the *PLAGL1* DMR at 6q24-25 is the primary epimutation seen in TNDM but has also been reported to be hypomethylated in 2-9% of BWS patients and in SRS [7,8,10,12,23,24]. Interestingly whereas BWS is characterised by prenatal overgrowth and frequent neonatal hypoglycaemia, TNDM is characterised by intra-uterine growth failure and lack of subcutaneous fat and neonatal hyperglycaemia [28,29]. Up to 10% of patients with SRS have maternal uniparental disomy of chromosome 7 (mUPD7) and a small number of cases will have chromosome 7 duplications [30]*.* The *MEST* DMR maps to 7q32 and in mice loss of the paternal allele leads to fetal growth failure [31]. Hence *MEST* has been extensively investigated as a potential candidate gene for SRS and although the precise role of this locus in SRS-associated growth restriction is still unclear, *MEST* DMR epimutations have been described in patients with BWS (approximately 8%), *PHP1B* (10%) and ME + SRS [7,25]. The insulin-like growth factor 2 receptor (IGF2R) inhibits IGF2-induced overgrowth by promoting clearance of IGF2 from the circulation [32]. In mice *Igf2r* is imprinted and maternally expressed but the evidence that *IGF2R* is imprinted in humans is less strong, though it may exhibit polymorphic imprinting [33]. In theory, gain of methylation at the *IGF2R* DMR would increase *IGF2R* expression and inhibit fetal growth whereas loss of methylation would promote overgrowth [34,35].

We found that LOM at *PLAGL1* and *MEST* DMRs occurred in a minority of BWS patients at a frequency that was similar to that in other studies [7]. As reported by others, we found that both LOM and GOM could occur at *IGF2R* DMR [36]. *IGF2R* hypermethylation has been reported in patients with growth retardation with and without SRS and we found *IGF2R* DMR hypermethylation in four BWS patients and a control individual. Though we did not find any overall correlations between methylation levels at *PLAGL1*, *MEST* and *IGF2R* DMRs, there was a tendency for hypomethylation at these loci to cluster in a small subset of patients. However, there was no evidence that patients with *IGF2R* hypermethylation were more susceptible to loss of methylation at *PLAGL1* or *MEST* DMRs. As found in our previous smaller study, ART-conceived children were significantly over-represented among BWS ME + patients but more extensive methylation profiling using the CpG methylation array did not clearly differentiate between post-ART and naturally conceived BWS patients. Previously methylation profiling at imprinted loci in ART-conceived children without imprinting disorders has shown no generalised tendency to methylation changes [37]. We investigated a cohort with IC2 epimutations and though it could be argued that such cases might have been susceptible to ART-associated imprinting errors, only a minority had a ME + epigenotype. At this stage it is not known whether this subset of ME + patients are genetically predisposed to epimutations (for example, in response to environmental factors such as ART) or whether there are specific environmental factors implicated (for example, precise embryo culture conditions in post-ART cases).

We investigated whether the loss of maternal allele KvDMR1 methylation (a paternal epigenotype pattern) in BWS IC2 epimutation cases was associated with the grandparental origin of the allele (hypothesising that the LOM might result from a failure to reset the imprinting on the grandpaternal chromosome). We found no evidence of bias in grandparental origin suggesting that the imprinting defect occurred after erasure of the parental imprint and the defective imprinting resulted from disordered establishment or maintenance of the maternal allele imprint (which might be after fertilization). Interestingly, Buiting *et al*. reported that in patients with Angelman syndrome (AS) and 15q11 IC epimutations (loss of maternal allele methylation), there was no bias in grandparental origin of the chromosome carrying the abnormal imprint, but in Prader-Willi syndrome patients with no IC deletion, the paternal chromosome carrying an incorrect maternal imprint was preferentially derived from the paternal grandmother, raising the possibility that such cases result from a failure to erase the maternal imprint [36]. Both BWS and AS have been associated with ART and the suggestion that epimutations in these disorders might result from failure to establish or maintain germline imprints could fit with either of the major hypotheses for the association of imprinting disorders with ART, namely, (a) that infertility *per se* predisposes to abnormal imprinting and/or (b) that aspects of ART (for example, ovarian hyperstimulation, *in vitro* embryo culture etcetera) predispose to abnormal imprinting [20,38].

## Conclusions

We did not detect any significant associations between loss (or gain) of methylation at *PLAGL1*, *MEST* and *IGF2R* DMRs and gestation-adjusted birth weight scores or specific clinical features of BWS and investigations in other cohorts have not detected consistent statistically significant clinical correlates of an ME + phenotype [7,8]. Though these findings could be interpreted as indicating that extra-11p15.5 epimutations might have no clinical significance, the case report of an ART-conceived child with clinically diagnosed BWS but loss of paternal allele methylation at the IC1 DMR (predicting a clinical diagnosis of SRS) and epimutations at extra-11p15.5 DMRs strongly suggests that there can be clinical significance. The failure to detect a clear relationship between epimutations at specific DMRs and a specific clinical phenotype may reflect a number of variables including, (a) the variability of degree of methylation loss at individual DMRs (consistent with mosaicism that might differ between tissues), (b) that a comprehensive analysis of all IC DMRs has not been undertaken (and it cannot be excluded that epimutations at currently unrecognised IC DMRs might exist) and (c) the complexity of functional interactions between imprinted genes (for example, *PLAGL1* and *IGF2*[39,40]). It was interesting that we found some evidence that there might be differences between BWS patients and controls at non-imprinted CpG sites. Further studies are required to confirm this observation. Analyses to uncover potential effects of specific aspects of an ME + epigenotype on the phenotypic expression of BWS are likely to require both comprehensive IC methylation profiling and multicentre collaboration to generate large cohorts of patients. Nevertheless, such a study would have relevance for BWS and other imprinting disorders and might also inform the potential role of epimutations in children conceived by ART.

## Abbreviations

AS: Angelman syndrome; ART: Assisted reproductive technologies; BWS: Beckwith-Wiedemann syndrome; IC: Imprinting centre; DMR: Differentially methylated region; GOM: Gain of methylation; ICSI: Intracytoplasmic sperm injection; IGF2R: Insulin-like growth factor 2 receptor; IVF: *In vitro* fertilization; LOM: Loss of methylation; MI: Methylation index; MS-MLPA: Methylation-specific-multiplex ligation-dependent probe amplification; qPCR: Quantitative polymerase chain reaction; SRS: Silver-Russell syndrome; TNDM: Transient neonatal diabetes mellitus.

## Competing interests

The authors declare that they have no competing interests.

## Authors’ contributions

LT, AS, MO, TH and CH performed laboratory investigations; DL, GK, HS, FM and EM collected and interpreted clinical and molecular information; LT, RPD and EM performed statistical analysis and data interpretation; EM conceived the study and wrote the first draft of the manuscript. All authors critically revised the manuscript. All authors read and approved the final manuscript.

## Supplementary Material

Additional file 1: Table S1 Absolute methylation indices for each of the analysed CpG on Illumina® GoldenGate Cancer Panel 1. Target ID (as per array output) is given. Red indicates imprinted CpGs. Methylation values given to three decimal places. Sample ID refers to patients with Beckwith-Wiedemann syndrome (BWS) and controls. The suffix (ART) indicates conception by assisted reproductive technologies. Click here for file

Additional file 2: Figure S1 Analysis of the imprinted gene CpG methylation data for Beckwith-Wiedemann syndrome (BWS) patients and controls demonstrated two principal clusters (see Results for details). The colours indicate methylation levels, ranging from red (hypermethylated) to blue (hypomethylated). **Figure S2.** Significant clustering of controls versus BWS seen when methylation at non-imprinted CpG sites was analysed (P = 0.0402) (see Results for details). The colours indicate methylation levels, ranging from red (hypermethylated) to blue (hypomethylated). Click here for file
