# Supplementary material for: Epimutation profiling in Beckwith-Wiedemann syndrome: relationship with assisted reproductive technology
Source: Clin Epigenetics. 2013 Dec 10;5(1):23. doi: 10.1186/1868-7083-5-23 (PMC3878854; doi:10.1186/1868-7083-5-23)
Supplement: Additional file 1: Table S1 — Absolute methylation indices for each of the analysed CpG on Illumina® GoldenGate Cancer Panel 1. Target ID (as per array output) is given. Red indicates imprinted CpGs. Methylation values given to three decimal places. Sample ID refers to patients with Beckwith-Wiedemann syndrome (BWS) and controls. The suffix (ART) indicates conception by assisted reproductive technologies. [file 1868-7083-5-23-S1.pdf]

TargetID	BWS1 (ART)	BWS2	BWS3 (ART)	BWS4	BWS5 (ART)	BWS6 (ART)	BWS7 (ART)	BWS8	BWS9 (ART)	BWS10	BWS11	BWS12 (ART)	BWS13 (ART)	BWS14 (ART)	BWS15 (ART)	BWS16
AATK_E63_R	0.1811	0.0348	0.9887	0.0000	0.9897	0.0000	0.0000	0.0097	0.9862	0.0097	0.0000	0.0255	0.9920	0.0000	0.9423	0.0424
ABCA1_E120_R	0.9823	0.0000	0.9837	0.9906	0.9901	0.9906	0.9910	0.9908	0.9848	0.0000	0.0000	0.9845	0.0000	0.9876	0.9869	0.8759
ABCA1_P45_F	0.9931	0.9922	0.9729	0.9904	0.9828	0.9899	0.0000	0.9862	0.9800	0.9927	0.0000	0.9441	0.0000	0.9867	0.9720	0.9873
ABCB4_E429_F	0.0107	0.0043	0.0108	0.0042	0.0061	0.0056	0.0024	0.0058	0.0000	0.0058	0.0066	0.0026	0.1682	0.0000	0.0045	0.0039
ABCC2_P88_F	0.1428	0.0447	0.0236	0.0199	0.0327	0.0155	0.0000	0.0494	0.0000	0.0711	0.0000	0.0181	0.1610	0.0000	0.0611	0.0150
ABCC5_P444_F	0.0143	0.0142	0.0106	0.3532	0.0048	0.0000	0.0799	0.4560	0.0000	0.1552	0.0975	0.0063	0.0654	0.0000	0.0075	0.1667
ABCG2_P178_R	0.2240	0.0671	0.8109	0.5070	0.0836	0.0240	0.0297	0.0470	0.0000	0.5609	0.6465	0.4483	0.3331	0.0000	0.0279	0.4753
ABCG2_P310_R	0.0000	0.9924	0.9863	0.0000	0.0000	0.0000	0.9859	0.9769	0.9852	0.9833	0.0000	0.9897	0.0000	0.9599	0.9851	0.9902
ABL1_P53_F	0.3073	0.3622	0.9904	0.9413	0.9686	0.9912	0.2897	0.9636	0.9852	0.2632	0.0167	0.2758	0.2658	0.9866	0.9729	0.4553
ABL2_P459_R	0.0000	0.0037	0.0000	0.0000	0.0000	0.0000	0.0000	0.0000	0.0000	0.0072	0.0000	0.0000	0.0000	0.0000	0.0000	0.0000
ABO_E110_F	0.0000	0.0076	0.0117	0.0839	0.0308	0.0300	0.0000	0.3316	0.0000	0.2215	0.0078	0.0055	0.0005	0.2700	0.0110	0.0067
ABO_P312_F	0.0150	0.0418	0.3382	0.0132	0.0116	0.0094	0.0632	0.0152	0.0000	0.0078	0.0132	0.0095	0.0078	0.1370	0.1963	0.2094
ACTG2_P455_R	0.0000	0.0000	0.0000	0.0000	0.0000	0.0000	0.0000	0.0000	0.0000	0.0000	0.0000	0.0021	0.0079	0.5614	0.0000	0.0000
ACVR1_P983_F	0.0104	0.0026	0.7079	0.0000	0.0034	0.0022	0.0000	0.0038	0.0000	0.0000	0.0000	0.0000	0.0000	0.0000	0.0000	0.0000
ACVR1B_E497_R	0.0187	0.0000	0.0000	0.0000	0.0056	0.0000	0.0127	0.0128	0.0000	0.0000	0.3551	0.0060	0.1170	0.0000	0.0086	0.0788
ACVR1B_P572_R	0.0252	0.2794	0.0209	0.0135	0.0335	0.5084	0.0137	0.0177	0.0000	0.3753	0.0732	0.0231	0.3521	0.0000	0.0092	0.0396
ACVR1C_P115_R	0.0000	0.0060	0.0113	0.0049	0.0000	0.0000	0.0000	0.0070	0.0000	0.0000	0.0000	0.0070	0.0063	0.0000	0.0000	0.0000
ACVR1C_P363_F	0.9849	0.0000	0.9898	0.9913	0.9783	0.0000	0.9909	0.0000	0.9925	0.0000	0.0000	0.0000	0.8786	0.9739	0.9875	0.9907
ACVR2B_E27_R	0.4082	0.2641	0.9885	0.9884	0.9896	0.6510	0.9911	0.9803	0.9862	0.3661	0.0000	0.9914	0.3535	0.9521	0.9821	0.9881
ACVR2B_P676_F	0.0089	0.0000	0.0000	0.0000	0.0000	0.0000	0.0000	0.0000	0.0000	0.0000	0.0000	0.0000	0.0000	0.0000	0.0000	0.0000
ADAMTS12_P250_R	0.0000	0.0000	0.0000	0.0025	0.0012	0.0000	0.0000	0.0000	0.0000	0.0000	0.0000	0.0000	0.0000	0.0000	0.0000	0.0000
ADCYAP1_E163_R	0.0000	0.0000	0.0690	0.1599	0.0111	0.0000	0.0213	0.0097	0.0000	0.0091	0.0000	0.0145	0.1041	0.0000	0.0163	0.1397
ADCYAP1_P398_F	0.9643	0.9538	0.6793	0.944	0.9824	0.9349	0.9845	0.9755	0.8737	0.9662	0.9960	0.9376	0.9861	0.9731	0.9854	0.2041
ADCYAP1_P455_R	0.0168	0.0106	0.4077	0.0166	0.0631	0.0098	0.1497	0.0173	0.0043	0.0308	0.1996	0.0097	0.0124	0.0192	0.0489	0.1395
AFF3_P122_F	0.0107	0.0072	0.2309	0.0000	0.0053	0.0040	0.0000	0.1769	0.0000	0.0000	0.1281	0.0058	0.0067	0.3640	0.2654	0.0000
AFF3_P808_F	0.0272	0.0000	0.0101	0.0000	0.0046	0.0107	0.0054	0.0033	0.0000	0.0116	0.2728	0.0076	0.0000	0.3797	0.0000	0.0000
AFP_P824_F	0.3868	0.0116	0.0174	0.0088	0.0165	0.0139	0.0000	0.0219	0.0000	0.1739	0.9959	0.0125	0.0091	0.8168	0.0208	0.9901
AGTR1_P154_F	0.0066	0.0000	0.0000	0.0000	0.0000	0.0000	0.0000	0.0000	0.0000	0.0000	0.0076	0.0000	0.0000	0.0000	0.0000	0.0000
AGTR1_P41_F	0.0238	0.0152	0.0270	0.4541	0.0172	0.0131	0.4753	0.0204	0.0000	0.0176	0.5913	0.0267	0.0162	0.0000	0.0196	0.2752
AHR_E103_F	0.0000	0.0000	0.9719	0.6701	0.0000	0.0000	0.0000	0.0000	0.9775	0.0000	0.0000	0.9528	0.0000	0.9531	0.9673	0.0000
AHR_P166_R	0.7606	0.7202	0.6115	0.7807	0.8316	0.8103	0.5473	0.8557	0.7814	0.7532	0.6921	0.8497	0.4251	0.7322	0.7688	0.5833

AIM2_E208_F	0.1439	0.0739	0.0595	0.2434	0.0147	0.0122	0.1023	0.0224	0.0000	0.3346	0.5640	0.0062	0.4824	0.0000	0.0079	0.0058
AKT1_P310_R	0.0000	0.0000	0.0094	0.0000	0.0074	0.0000	0.0085	0.7163	0.5592	0.0078	0.0000	0.0147	0.0000	0.0000	0.1590	0.0053
ALK_P28_F	0.0176	0.0000	0.0164	0.0074	0.8965	0.4788	0.0052	0.2127	0.0000	0.0067	0.0112	0.0045	0.0031	0.2915	0.0074	0.1134
ALOX12_E85_R	0.0102	0.0000	0.0050	0.0021	0.0000	0.0000	0.0016	0.0025	0.0000	0.0030	0.0038	0.0000	0.0000	0.0000	0.0005	0.0002
ALOX12_P223_R	0.0000	0.0592	0.8378	0.9889	0.9874	0.4672	0.0011	0.9781	0.0000	0.0000	0.0000	0.0000	0.9915	0.9070	0.9729	0.0000
APBA1_E99_R	0.0000	0.0000	0.0000	0.0000	0.0000	0.0000	0.0000	0.9701	0.0000	0.0000	0.0000	0.0000	0.0021	0.6984	0.0000	0.0000
APBA1_P644_F	0.0000	0.0000	0.0000	0.0000	0.0000	0.0000	0.0000	0.0000	0.0000	0.0000	0.0000	0.0000	0.0000	0.0000	0.0000	0.0000
APBA2_P227_F	0.0000	0.0068	0.0000	0.0000	0.0144	0.0046	0.0000	0.0071	0.0000	0.0302	0.0000	0.0000	0.0085	0.0000	0.0000	0.0023
APC_P280_R	0.0170	0.0000	0.1593	0.0000	0.0000	0.0000	0.0000	0.0000	0.9865	0.9954	0.9945	0.0000	0.0000	0.0000	0.1766	0.0000
APOA1_P75_F	0.2739	0.0180	0.0286	0.0000	0.0258	0.0000	0.0032	0.0000	0.0000	0.0227	0.1235	0.0000	0.0441	0.9310	0.0183	0.0000
APOC1_P406_R	0.0142	0.0154	0.4390	0.0071	0.6099	0.0074	0.0063	0.6132	0.7165	0.0070	0.0095	0.0083	0.0764	0.5716	0.2020	0.0063
APP_E8_F	0.0000	0.0000	0.0000	0.0000	0.0000	0.0328	0.0095	0.0029	0.0000	0.0473	0.0000	0.0043	0.0000	0.0000	0.0000	0.0025
APP_P179_R	0.2609	0.2063	0.0303	0.0164	0.0244	0.4810	0.0234	0.5510	0.0000	0.0559	0.4664	0.0488	0.0297	0.0000	0.1157	0.3956
AR_P189_R	0.0344	0.0150	0.2889	0.0141	0.2585	0.2198	0.0000	0.0091	0.0000	0.0660	0.2240	0.1924	0.1524	0.0000	0.0119	0.0054
AREG_E25_F	0.0091	0.0000	0.0070	0.0000	0.0025	0.0008	0.0000	0.0111	0.0000	0.0068	0.0000	0.0000	0.0043	0.0000	0.0028	0.0027
ARHGDIB_P148_R	0.0152	0.0163	0.0282	0.2615	0.0295	0.0120	0.1666	0.3224	0.4583	0.0135	0.1287	0.0189	0.2111	0.4199	0.0271	0.0173
ARNT_P238_R	0.0126	0.4825	0.0140	0.0093	0.0000	0.0064	0.0833	0.0124	0.0000	0.0000	0.0000	0.0054	0.0066	0.0000	0.0037	0.2379
ASB4_P391_F	0.0110	0.0033	0.0000	0.0056	0.0000	0.0668	0.2624	0.0094	0.0000	0.0052	0.0000	0.0040	0.0000	0.9491	0.0000	0.0124
ASB4_P52_R	0.0000	0.0084	0.0000	0.0052	0.0095	0.0000	0.0049	0.0070	0.0000	0.0000	0.0119	0.0000	0.0000	0.0000	0.0114	0.0062
ASCL1_E24_F	0.0156	0.0001	0.0000	0.0000	0.0000	0.0000	0.0000	0.0000	0.0000	0.0000	0.0000	0.0042	0.0000	0.0000	0.0000	0.0000
ASCL1_P747_F	0.0000	0.0048	0.0086	0.0046	0.0071	0.1023	0.0038	0.0035	0.0000	0.1434	0.0710	0.0065	0.0733	0.0000	0.0021	0.0259
ASCL2_E76_R	0.0021	0.0000	0.0000	0.0000	0.0000	0.0000	0.0000	0.0000	0.0000	0.0000	0.0000	0.0000	0.0000	0.0000	0.0000	0.0000
ASCL2_P360_F	0.0000	0.0000	0.0078	0.0022	0.0000	0.0000	0.0000	0.0033	0.0000	0.0000	0.0101	0.0000	0.0035	0.0000	0.0000	0.0014
ASCL2_P609_R	0.0437	0.0035	0.0000	0.0000	0.0000	0.0099	0.0000	0.0000	0.0000	0.0000	0.0000	0.0000	0.0044	0.0000	0.0000	0.0000
ATP10A_P147_F	0.0509	0.0069	0.0273	0.0027	0.0000	0.0706	0.0000	0.0082	0.0000	0.0833	0.0731	0.0016	0.0077	0.0000	0.0000	0.0042
ATP10A_P524_R	0.0000	0.0151	0.0000	0.0000	0.0000	0.0144	0.0095	0.0173	0.0000	0.0000	0.0000	0.0156	0.0000	0.0000	0.0054	0.0000
AXIN1_P995_R	0.0376	0.0291	0.0278	0.0163	0.0378	0.0339	0.0213	0.0206	0.0000	0.5566	0.4248	0.0306	0.4682	0.0000	0.0140	0.0132
AXL_E61_F	0.0108	0.0072	0.0084	0.0000	0.0000	0.0033	0.0031	0.3723	0.0000	0.0035	0.0000	0.0040	0.0000	0.0000	0.0000	0.0013
BAX_E281_R	0.0324	0.6469	0.0202	0.4877	0.0279	0.0139	0.6820	0.0208	0.0000	0.6299	0.6624	0.0121	0.5242	0.0000	0.0119	0.4340
BCAM_E100_R	0.0101	0.0144	0.7218	0.0092	0.0105	0.0052	0.0067	0.0123	0.0000	0.0721	0.1381	0.1121	0.0078	0.0012	0.6393	0.0129
BCAM_P205_F	0.0000	0.3501	0.0166	0.0069	0.4883	0.0228	0.0174	0.0233	0.0000	0.0000	0.0000	0.0139	0.0269	0.0000	0.0261	0.0170
BCAP31_P1131_F	0.0390	0.0253	0.0282	0.0206	0.0366	0.0189	0.0192	0.0222	0.0000	0.8137	0.8147	0.0259	0.7325	0.0072	0.0192	0.0157
BCL2L2_E172_F	0.0000	0.0048	0.0045	0.0008	0.0022	0.0000	0.0028	0.0013	0.0000	0.0044	0.0000	0.0000	0.0015	0.0000	0.0000	0.0000

BCL2L2_P280_F	0.0116	0.0000	0.0144	0.0056	0.0000	0.0036	0.0000	0.0000	0.0000	0.0000	0.0000	0.0000	0.0029	0.0000	0.0008	0.0000
BCL3_E71_F	0.0059	0.0040	0.0089	0.0045	0.0058	0.0031	0.0035	0.0043	0.0000	0.0000	0.0000	0.0029	0.0031	0.0000	0.0004	0.0033
BCL3_P1038_R	0.0177	0.0165	0.0219	0.0104	0.0082	0.0082	0.0075	0.0122	0.0000	0.0114	0.3326	0.0096	0.0000	0.0000	0.0072	0.0187
BCL6_P248_R	0.0000	0.0000	0.0044	0.0000	0.0000	0.0000	0.0000	0.0014	0.0000	0.0000	0.0000	0.0000	0.0034	0.0000	0.0000	0.0000
BDNF_E19_R	0.0113	0.0178	0.0063	0.0033	0.0843	0.0033	0.0087	0.0487	0.0281	0.3865	0.0099	0.0049	0.0096	0.0853	0.0799	0.0037
BDNF_P259_R	0.6262	0.9934	0.9214	0.9887	0.9901	0.9584	0.8409	0.9472	0.9896	0.0371	0.6478	0.9898	0.9932	0.9754	0.9874	0.9933
BGN_E282_R	0.0102	0.0000	0.0111	0.0032	0.0000	0.0000	0.0000	0.3336	0.0000	0.0078	0.0000	0.0071	0.0000	0.0000	0.0000	0.0000
BGN_P333_R	0.0068	0.0419	0.5371	0.0048	0.0189	0.0000	0.0030	0.3582	0.0000	0.2786	0.0000	0.0041	0.1042	0.0956	0.0138	0.0000
BIRC4_P122_R	0.0256	0.2068	0.0323	0.0091	0.0136	0.0146	0.0132	0.3346	0.5321	0.2405	0.0339	0.0221	0.0098	0.0000	0.0087	0.0092
BIRC5_E89_F	0.0679	0.0163	0.0203	0.0134	0.0247	0.0672	0.0741	0.0177	0.0000	0.0107	0.1136	0.0125	0.0888	0.0072	0.0298	0.0752
BLK_P14_F	0.0741	0.2506	0.0135	0.0153	0.0083	0.3506	0.0156	0.0111	0.0000	0.2279	0.0646	0.0062	0.0046	0.0000	0.0126	0.0059
BMP2_E48_R	0.0351	0.2518	0.0219	0.0117	0.0481	0.0106	0.0082	0.0114	0.0000	0.4373	0.0189	0.3831	0.0124	0.0000	0.0115	0.1212
BMP2_P1201_F	0.0040	0.0000	0.0000	0.0000	0.0000	0.0000	0.0000	0.0014	0.0000	0.0000	0.0049	0.0000	0.0019	0.0000	0.0000	0.0000
BMP3_E147_F	0.0000	0.0000	0.0000	0.0000	0.0000	0.0000	0.0000	0.0000	0.0000	0.0000	0.0000	0.0000	0.0000	0.0000	0.0000	0.0000
BMP3_P56_R	0.0000	0.6939	0.0088	0.0000	0.0002	0.0000	0.0052	0.0093	0.0000	0.0000	0.0000	0.0069	0.0047	0.0000	0.0000	0.0000
BMP4_P199_R	0.0642	0.1037	0.0212	0.0179	0.1033	0.0939	0.0823	0.0178	0.0000	0.0103	0.0100	0.0897	0.0144	0.1051	0.4286	0.0847
BMP6_P398_F	0.0000	0.9941	0.9902	0.9649	0.9841	0.9930	0.7727	0.9882	0.9899	0.0058	0.0000	0.9911	0.0000	0.9847	0.9878	0.9907
BMPR1A_P956_F	0.0226	0.0222	0.4681	0.0950	0.0219	0.0175	0.0074	0.3616	0.0000	0.0075	0.0734	0.2501	0.1850	0.0000	0.0237	0.0150
BMPR2_E435_F	0.0000	0.0000	0.0000	0.0000	0.0000	0.0000	0.0000	0.0000	0.0000	0.0000	0.0038	0.0000	0.0000	0.0000	0.0000	0.0000
BMPR2_P1271_F	0.0665	0.0231	0.0066	0.7972	0.0311	0.0221	0.4664	0.0073	0.0000	0.0300	0.0057	0.0174	0.0000	0.0000	0.0234	0.3932
BSG_P211_R	0.2644	0.1106	0.4356	0.5057	0.1435	0.4805	0.4757	0.0731	0.0337	0.4592	0.4200	0.0851	0.4910	0.1476	0.0974	0.3631
BTK_P105_F	0.1093	0.0117	0.0166	0.1247	0.0130	0.0050	0.0496	0.6625	0.0000	0.0066	0.1496	0.0106	0.1196	0.0000	0.0273	0.1632
C20orf47_P225_R	0.0186	0.0067	0.0216	0.0000	0.6000	0.0000	0.0053	0.0000	0.0000	0.0087	0.0000	0.0000	0.0000	0.0000	0.0000	0.0608
CALCA_E174_R	0.6482	0.1281	0.2200	0.1090	0.2942	0.1132	0.0771	0.1533	0.0290	0.6111	0.6201	0.1274	0.0872	0.0563	0.1079	0.5643
CAPG_E228_F	0.0000	0.6688	0.0116	0.0000	0.0000	0.0006	0.0029	0.0011	0.0000	0.0000	0.0000	0.6857	0.0000	0.0000	0.0000	0.0028
CASP10_E139_F	0.0000	0.0118	0.0102	0.0068	0.0190	0.0056	0.0000	0.0061	0.0000	0.0138	0.0096	0.0078	0.1038	0.0000	0.0084	0.0000
CASP10_P186_F	0.0000	0.7439	0.0044	0.9423	0.9026	0.9888	0.9896	0.9764	0.0000	0.0000	0.0000	0.9861	0.9935	0.9699	0.5542	0.9822
CASP2_P192_F	0.0393	0.0396	0.1274	0.4532	0.1065	0.3662	0.3147	0.0312	0.0016	0.2379	0.3623	0.3752	0.0422	0.0158	0.1664	0.3270
CASP3_P420_R	0.0000	0.0080	0.0092	0.0019	0.0042	0.0000	0.0000	0.0095	0.0000	0.0000	0.0150	0.0000	0.0000	0.0000	0.0000	0.0023
CASP6_P201_F	0.0000	0.0027	0.9751	0.0180	0.9765	0.0680	0.0025	0.8351	0.9837	0.2358	0.0000	0.0000	0.0000	0.6260	0.7849	0.0075
CASP6_P230_R	0.0212	0.1685	0.9859	0.0258	0.0083	0.9923	0.0000	0.6275	0.0000	0.0125	0.0259	0.0058	0.0456	0.3198	0.0787	0.0036
CAV1_P130_R	0.2472	0.3923	0.2382	0.0000	0.0000	0.0098	0.0744	0.0000	0.0000	0.3356	0.0153	0.9905	0.2618	0.7382	0.0000	0.1852
CAV1_P169_F	0.1533	0.1705	0.0261	0.0170	0.0291	0.1954	0.1229	0.1697	0.0000	0.1646	0.1749	0.1577	0.0155	0.0061	0.0255	0.1175

CAV2_E33_R	0.1703	0.4041	0.0685	0.0151	0.0191	0.0108	0.0126	0.1863	0.0000	0.0751	0.0218	0.0860	0.0088	0.3419	0.2382	0.6703
CCKBR_P361_R	0.1783	0.0108	0.0174	0.0143	0.0185	0.4653	0.0109	0.0172	0.0000	0.0411	0.1157	0.0155	0.0134	0.0000	0.0100	0.0081
CCKBR_P480_F	0.1503	0.0000	0.0060	0.0000	0.0000	0.0051	0.0041	0.0000	0.0000	0.0000	0.0109	0.0000	0.0029	0.0000	0.0000	0.0034
CCNA1_E7_F	0.9923	0.9875	0.9810	0.9864	0.9848	0.0801	0.9881	0.8071	0.9705	0.0000	0.9947	0.3369	0.0000	0.9061	0.9788	0.9814
CCNA1_P216_F	0.0000	0.0110	0.0000	0.0000	0.0000	0.0000	0.0016	0.0000	0.0000	0.0055	0.0000	0.0000	0.0000	0.0000	0.0000	0.0027
CCNC_P132_R	0.0000	0.0071	0.0000	0.0053	0.0000	0.0000	0.0000	0.0064	0.0000	0.0000	0.0000	0.0000	0.0000	0.0000	0.0000	0.0069
CCND1_E280_R	0.9920	0.0000	0.0000	0.0000	0.6959	0.0025	0.7823	0.8550	0.0000	0.0080	0.0143	0.0031	0.0000	0.5139	0.3376	0.0000
CCND1_P343_R	0.0000	0.0000	0.0000	0.0000	0.0000	0.0000	0.0026	0.0000	0.0000	0.0050	0.0000	0.0000	0.0000	0.0000	0.0000	0.0000
CCND2_P887_F	0.0000	0.0000	0.0000	0.0000	0.0000	0.0000	0.0000	0.0037	0.0000	0.0000	0.0000	0.0000	0.0010	0.0000	0.0000	0.0000
CCND2_P898_R	0.1158	0.5945	0.0726	0.1085	0.9916	0.7743	0.0234	0.0371	0.8022	0.9927	0.0676	0.5358	0.9923	0.9456	0.9009	0.9899
CCNE1_P683_F	0.0000	0.0000	0.0000	0.0000	0.0000	0.0000	0.0000	0.0000	0.0000	0.0000	0.0000	0.0000	0.0000	0.0800	0.0000	0.0000
CD1A_P414_R	0.0000	0.0106	0.0142	0.0046	0.0083	0.0000	0.0000	0.0164	0.0000	0.0081	0.0000	0.0048	0.1162	0.0000	0.0083	0.0532
CD2_P68_F	0.1185	0.7621	0.9194	0.0033	0.6185	0.0054	0.4803	0.5662	0.7699	0.8067	0.0142	0.0092	0.0000	0.3026	0.4013	0.5405
CD34_P339_R	0.0174	0.1973	0.0250	0.0138	0.0241	0.2811	0.1633	0.0226	0.0000	0.2300	0.2340	0.0177	0.0109	0.0031	0.0199	0.2146
CD34_P780_R	0.0257	0.0238	0.6233	0.8524	0.7601	0.8605	0.9864	0.8638	0.8400	0.2136	0.3367	0.9634	0.6620	0.8945	0.6529	0.6615
CD40_E58_R	0.3865	0.0041	0.0050	0.0000	0.0000	0.0046	0.0000	0.0058	0.0000	0.0000	0.0000	0.0029	0.2132	0.0000	0.0000	0.0024
CD40_P372_R	0.0270	0.2185	0.0291	0.2264	0.0472	0.0196	0.0160	0.0235	0.0000	0.1592	0.0155	0.0211	0.0209	0.0136	0.0387	0.0161
CD44_E26_F	0.0000	0.0155	0.0141	0.0000	0.0000	0.0000	0.0000	0.2421	0.0000	0.0000	0.0000	0.0059	0.0000	0.0000	0.0000	0.0037
CD44_P87_F	0.0127	0.0000	0.0036	0.0000	0.8756	0.0023	0.9927	0.8542	0.0000	0.0000	0.0000	0.0000	0.0000	0.7365	0.9827	0.5888
CD86_P3_F	0.0000	0.0000	0.0000	0.0000	0.0000	0.0000	0.0000	0.0000	0.0000	0.0000	0.0000	0.0000	0.0000	0.0000	0.0000	0.0037
CDC25B_E83_F	0.0000	0.0000	0.0046	0.0000	0.0000	0.0013	0.0000	0.0000	0.0000	0.0081	0.0000	0.0000	0.0013	0.0000	0.0000	0.0000
CDC25B_P11_R	0.0000	0.3371	0.0160	0.0308	0.0000	0.0000	0.0000	0.1509	0.0000	0.0000	0.0000	0.0059	0.0000	0.0000	0.0018	0.0062
CDH1_P52_R	0.0000	0.0000	0.0000	0.0000	0.0000	0.0020	0.0000	0.0018	0.0000	0.0046	0.0000	0.0024	0.0000	0.0000	0.0000	0.0004
CDH11_E102_R	0.0000	0.0000	0.0181	0.0036	0.0013	0.0073	0.0057	0.0057	0.0000	0.0000	0.0000	0.0472	0.0056	0.0000	0.0000	0.0000
CDH11_P203_R	0.6092	0.5932	0.0282	0.0169	0.7305	0.4918	0.0131	0.0196	0.0000	0.4417	0.0208	0.0211	0.0105	0.0000	0.5773	0.0102
CDH11_P354_R	0.0000	0.0065	0.0101	0.0000	0.0000	0.0000	0.0038	0.0057	0.0000	0.9951	0.0162	0.0000	0.0032	0.0000	0.0057	0.0000
CDH13_E102_F	0.9931	0.9942	0.6732	0.9898	0.9817	0.9929	0.9908	0.9889	0.0000	0.8384	0.9971	0.9926	0.9935	0.9887	0.9839	0.0000
CDH17_E31_F	0.0000	0.0057	0.0581	0.1848	0.0000	0.0099	0.1158	0.0163	0.0000	0.1277	0.3299	0.3658	0.2977	0.0000	0.0118	0.0397
CDH17_P532_F	0.6494	0.6235	0.9300	0.9911	0.9873	0.9913	0.4390	0.9010	0.9875	0.5806	0.5721	0.8969	0.9923	0.9421	0.9762	0.9891
CDH3_E100_R	0.0177	0.1856	0.0295	0.0211	0.0305	0.0108	0.1372	0.0335	0.0000	0.1192	0.2458	0.0218	0.1586	0.5361	0.0389	0.1918
CDH3_P87_R	0.0000	0.0060	0.0104	0.0005	0.0014	0.0023	0.0020	0.0056	0.0000	0.0072	0.0000	0.0039	0.0000	0.0000	0.0000	0.0039
CDK10_E74_F	0.0432	0.9933	0.7937	0.6913	0.0897	0.5298	0.2888	0.5129	0.0000	0.4460	0.0247	0.0237	0.4548	0.0156	0.7651	0.4373
CDK2_P330_R	0.0000	0.0000	0.9184	0.0000	0.7893	0.9918	0.0000	0.5373	0.9884	0.2043	0.2005	0.9008	0.0027	0.7877	0.9822	0.0000

CDK6_E256_F	0.0000	0.0101	0.0156	0.0052	0.0027	0.0000	0.0000	0.4685	0.0000	0.0000	0.0091	0.6816	0.0078	0.0000	0.0057	0.0000
CDK6_P291_R	0.1911	0.2317	0.3951	0.0155	0.0250	0.3458	0.2696	0.3466	0.0000	0.3459	0.2083	0.0101	0.0128	0.0067	0.0779	0.0422
CDKN1A_E101_F	0.1366	0.0345	0.0011	0.0000	0.0000	0.0000	0.0000	0.0091	0.0000	0.0000	0.0073	0.0077	0.0000	0.0000	0.0000	0.0122
CDKN1A_P242_F	0.0000	0.0000	0.0065	0.0000	0.0013	0.0000	0.0000	0.0089	0.0000	0.0122	0.0000	0.0000	0.0000	0.0000	0.0000	0.1354
CDKN1B_P1161_F	0.0285	0.0000	0.0000	0.0000	0.0000	0.0012	0.0000	0.0000	0.0000	0.8703	0.0000	0.0000	0.0031	0.0000	0.0000	0.0000
CDKN2A_E121_R	0.0186	0.0064	0.0086	0.0000	0.0051	0.0044	0.0086	0.0099	0.0000	0.0589	0.0000	0.0000	0.0078	0.0000	0.0058	0.0047
CDKN2B_E220_F	0.2915	0.0000	0.0192	0.0000	0.0069	0.0000	0.0000	0.8009	0.0000	0.0000	0.0000	0.0000	0.0000	0.0000	0.0033	0.0032
CDM_seq_21_S260_R	0.0000	0.0000	0.0067	0.0052	0.0082	0.0000	0.0000	0.0078	0.0000	0.0075	0.0129	0.0015	0.0000	0.0000	0.0000	0.0097
CEACAM1_E57_R	0.0097	0.0048	0.0057	0.0000	0.0005	0.0000	0.0000	0.0063	0.0000	0.0054	0.0072	0.0047	0.0036	0.0000	0.0000	0.0046
CEACAM1_P44_R	0.0204	0.0815	0.0156	0.2542	0.0100	0.2391	0.1312	0.0114	0.0000	0.0229	0.0000	0.0102	0.0000	0.0000	0.0121	0.0055
CEBPA_P1163_R	0.0161	0.1771	0.0308	0.0115	0.0165	0.1479	0.0121	0.0213	0.0000	0.1526	0.1050	0.1444	0.0094	0.2341	0.0228	0.0094
CEBPA_P706_F	0.2435	0.9927	0.0000	0.0000	0.0000	0.0000	0.0053	0.0117	0.7978	0.0000	0.0000	0.0052	0.0094	0.0000	0.0000	0.0000
CFTR_P115_F	0.0689	0.0064	0.0070	0.0030	0.5896	0.0035	0.0026	0.0134	0.0000	0.0195	0.0000	0.0086	0.0082	0.0000	0.0000	0.0000
CHD2_P451_F	0.0068	0.0000	0.0000	0.0031	0.0000	0.9911	0.0000	0.9007	0.0000	0.0000	0.0000	0.0000	0.9939	0.0000	0.0000	0.0000
CHFR_P501_F	0.0071	0.0034	0.0095	0.0045	0.0006	0.0061	0.0063	0.0075	0.0000	0.0040	0.0057	0.0000	0.0013	0.0000	0.0000	0.0029
CHFR_P635_R	0.9918	0.9895	0.7137	0.9470	0.9846	0.8880	0.2767	0.7490	0.9907	0.7114	0.9728	0.0000	0.9955	0.9473	0.9872	0.8954
CHGA_E52_F	0.0204	0.0000	0.0000	0.0055	0.0000	0.0057	0.0218	0.0010	0.0000	0.0000	0.0651	0.0050	0.0000	0.0000	0.0000	0.0035
CHI3L2_E10_F	0.0732	0.0000	0.3129	0.0071	0.0290	0.0071	0.0121	0.4141	0.8164	0.0072	0.0096	0.6821	0.0061	0.1825	0.0073	0.0077
CLK1_P538_F	0.0000	0.0000	0.0000	0.0000	0.0000	0.0013	0.0000	0.0011	0.0000	0.0000	0.0060	0.0000	0.0000	0.0000	0.0000	0.0000
COL18A1_P365_R	0.1900	0.0149	0.0162	0.0069	0.0159	0.9890	0.0115	0.0127	0.0000	0.0149	0.2211	0.0155	0.2300	0.0000	0.0041	0.0090
COL1A1_P5_F	0.0000	0.0068	0.0000	0.0027	0.0000	0.0000	0.0000	0.0003	0.0000	0.0000	0.0000	0.0000	0.0000	0.0000	0.0000	0.0000
COL1A2_E299_F	0.0117	0.0119	0.0096	0.2488	0.0088	0.2855	0.0097	0.1789	0.0000	0.7218	0.0000	0.0063	0.4761	0.1308	0.4913	0.0060
COL1A2_P407_R	0.0000	0.0031	0.0030	0.0000	0.0029	0.0037	0.0000	0.0000	0.0000	0.0042	0.0000	0.0000	0.0000	0.5200	0.0000	0.0000
COL1A2_P48_R	0.0305	0.1198	0.0405	0.0341	0.1558	0.0234	0.0293	0.0247	0.0000	0.1310	0.1945	0.0223	0.0345	0.0000	0.0525	0.0148
COL4A3_E205_R	0.0126	0.0000	0.0000	0.0000	0.0000	0.0000	0.9567	0.0002	0.0000	0.0000	0.0000	0.0022	0.0000	0.0000	0.0000	0.0000
COL4A3_P545_F	0.1140	0.5500	0.0797	0.8556	0.1423	0.8402	0.7360	0.0324	0.0000	0.7472	0.7647	0.7713	0.7522	0.0123	0.0659	0.7481
COL6A1_P283_F	0.8292	0.0184	0.0300	0.0089	0.4735	0.0296	0.0188	0.0360	0.8002	0.3390	0.0096	0.1151	0.7026	0.0000	0.0419	0.0531
COL6A1_P425_F	0.0000	0.0000	0.0157	0.0104	0.0041	0.2608	0.0081	0.0151	0.0000	0.0422	0.1712	0.0081	0.3388	0.0000	0.0026	0.9884
COPG2_P298_F	0.1185	0.6003	0.0657	0.6283	0.1434	0.5962	0.5308	0.0613	0.0556	0.5995	0.6066	0.1124	0.5890	0.0325	0.0748	0.0689
CPA4_P1265_R	0.0573	0.7786	0.0642	0.7184	0.8083	0.0147	0.0405	0.6703	0.0000	0.0404	0.7707	0.0497	0.7208	0.0073	0.0912	0.5926
CREB1_P819_F	0.0000	0.0166	0.0130	0.0000	0.0071	0.0000	0.0000	0.0086	0.0000	0.0000	0.0097	0.0509	0.0049	0.0000	0.0018	0.0000
CRIP1_P874_R	0.0215	0.9942	0.8475	0.4967	0.0124	0.0104	0.3174	0.0178	0.0000	0.4871	0.0130	0.0102	0.3921	0.0004	0.1707	0.2020
CRK_P721_F	0.0119	0.0063	0.0573	0.0061	0.0061	0.0058	0.0043	0.0053	0.0000	0.0066	0.0094	0.0057	0.0050	0.0053	0.0214	0.0051

CSF1_P217_F	0.1410	0.0119	0.0301	0.3040	0.0183	0.9910	0.0502	0.0241	0.9889	0.1149	0.1460	0.0997	0.1935	0.5181	0.7229	0.0084
CSF1_P339_F	0.0000	0.0000	0.0000	0.0000	0.0000	0.0000	0.0000	0.0042	0.0000	0.0011	0.0000	0.0000	0.0000	0.0000	0.0000	0.0000
CSF1R_E26_F	0.0099	0.0099	0.0049	0.0000	0.0015	0.0062	0.0044	0.0080	0.0000	0.0000	0.0000	0.0063	0.0000	0.0000	0.0000	0.0000
CSF3R_P472_F	0.1592	0.0235	0.1626	0.0518	0.1715	0.0133	0.0353	0.0416	0.0000	0.0315	0.1259	0.1660	0.1554	0.0241	0.0424	0.0360
CSPG2_E38_F	0.0060	0.0000	0.0114	0.0040	0.0037	0.0000	0.0054	0.0068	0.0000	0.0000	0.0041	0.0065	0.0059	0.0000	0.0006	0.0000
CSPG2_P82_R	0.0105	0.3390	0.0092	0.0000	0.0013	0.0000	0.0000	0.0148	0.0000	0.0000	0.0087	0.0091	0.0044	0.0000	0.0002	0.0045
CSTB_E410_F	0.0000	0.9934	0.8329	0.0000	0.9820	0.0056	0.0032	0.6716	0.9848	0.0409	0.0000	0.0967	0.0068	0.9491	0.9809	0.0000
CTAG1B_P4_R	0.0000	0.0000	0.0008	0.0000	0.0055	0.0000	0.0013	0.0000	0.0000	0.0112	0.0000	0.0000	0.0000	0.0000	0.6018	0.9869
CTAG1B_P77_F	0.3423	0.0172	0.0373	0.0215	0.6399	0.0145	0.8605	0.0364	0.0515	0.0149	0.4057	0.3228	0.0174	0.2710	0.1815	0.0993
CTAG2_P1426_F	0.8007	0.0231	0.0634	0.6377	0.0530	0.6581	0.0184	0.0163	0.0000	0.6758	0.7552	0.6005	0.7564	0.0000	0.0275	0.5821
CTGF_E156_F	0.0000	0.0000	0.0023	0.0025	0.0000	0.0000	0.0000	0.0026	0.0000	0.0000	0.0000	0.0050	0.0000	0.0000	0.0000	0.0037
CTLA4_P1128_F	0.9218	0.1197	0.7109	0.6785	0.5903	0.9663	0.9822	0.4064	0.2832	0.9916	0.9746	0.6421	0.9887	0.1356	0.0000	0.1692
CTNNA1_P185_R	0.0125	0.0063	0.0067	0.0031	0.0035	0.0000	0.0000	0.0050	0.0000	0.0000	0.0000	0.0000	0.0060	0.0000	0.0018	0.0025
CTNNA1_P382_R	0.0098	0.1376	0.0095	0.0000	0.0000	0.0050	0.0000	0.0059	0.0000	0.1484	0.0000	0.0056	0.1741	0.0000	0.0082	0.0046
CTNNB1_P757_F	0.0153	0.0836	0.0104	0.0088	0.0674	0.0063	0.0454	0.0659	0.0000	0.0633	0.0413	0.0078	0.0058	0.0000	0.0229	0.0070
CTSD_P726_F	0.0000	0.0000	0.0000	0.0000	0.0000	0.0000	0.0000	0.0007	0.0000	0.0000	0.0000	0.0000	0.0000	0.0000	0.0000	0.0000
CTSH_P238_F	0.0000	0.0000	0.0088	0.0000	0.0000	0.0051	0.0000	0.0000	0.0000	0.0000	0.0000	0.0330	0.0000	0.0000	0.0000	0.0000
CTSL_P264_R	0.0000	0.0021	0.0083	0.0009	0.9769	0.0688	0.0048	0.0076	0.0000	0.0000	0.0000	0.0000	0.0059	0.0000	0.0000	0.0043
CTSL_P81_F	0.1342	0.0094	0.8800	0.0062	0.0000	0.0085	0.0038	0.0074	0.0000	0.0000	0.0000	0.0058	0.0117	0.0000	0.0000	0.0063
CTTN_E29_R	0.0000	0.0000	0.0108	0.0044	0.0038	0.0086	0.0056	0.0081	0.0000	0.0000	0.0102	0.0166	0.0000	0.0000	0.0089	0.0058
CYP1A1_P382_F	0.0445	0.0125	0.0212	0.6344	0.0222	0.0174	0.3679	0.0323	0.0000	0.4752	0.0251	0.1989	0.3254	0.0000	0.0638	0.5466
CYP2E1_P416_F	0.1127	0.0364	0.0157	0.1511	0.0196	0.0024	0.0000	0.0133	0.0000	0.0000	0.0000	0.0133	0.0097	0.0000	0.0141	0.0000
DAB2_P35_F	0.0217	0.0000	0.0000	0.0057	0.0000	0.0000	0.0000	0.0000	0.0000	0.0120	0.0000	0.0000	0.0070	0.0000	0.0000	0.0021
DAB2_P468_F	0.0907	0.0278	0.0307	0.0358	0.0525	0.4540	0.0170	0.0247	0.0000	0.1223	0.0163	0.1499	0.0108	0.0433	0.0751	0.0239
DAB2IP_P9_F	0.0000	0.0026	0.0000	0.0000	0.0000	0.0000	0.0012	0.0066	0.0000	0.0005	0.0055	0.0000	0.0000	0.0000	0.0000	0.0000
DAPK1_P10_F	0.0000	0.0050	0.0079	0.0000	0.0045	0.9918	0.0000	0.5042	0.0000	0.0000	0.0000	0.0022	0.0047	0.0000	0.0000	0.0000
DAPK1_P345_R	0.0000	0.0000	0.0045	0.0000	0.8104	0.0000	0.0000	0.0000	0.0000	0.0000	0.0000	0.0038	0.9937	0.0000	0.0000	0.0025
DBC1_P351_R	0.0000	0.0044	0.6857	0.0000	0.0000	0.0023	0.0000	0.0000	0.0000	0.0000	0.0088	0.0000	0.0000	0.0000	0.0000	0.0000
DCC_P177_F	0.2983	0.4449	0.0139	0.0069	0.0107	0.0123	0.0072	0.0113	0.0000	0.0000	0.0000	0.0054	0.0078	0.1945	0.0141	0.0000
DCC_P471_R	0.1964	0.1808	0.0185	0.0226	0.0337	0.0085	0.0096	0.0202	0.0000	0.1937	0.0111	0.1700	0.2218	0.0017	0.0107	0.0068
DCN_P1320_R	0.0000	0.0000	0.0000	0.0000	0.0000	0.0000	0.0000	0.0021	0.0000	0.0000	0.0199	0.0000	0.0000	0.0000	0.0000	0.0000
DDB2_P407_F	0.0138	0.0000	0.0000	0.0001	0.0000	0.0057	0.0000	0.0000	0.0000	0.0000	0.0000	0.0000	0.0000	0.0000	0.5740	0.0000
DDB2_P613_R	0.4017	0.2802	0.1698	0.1488	0.0060	0.0104	0.0527	0.0092	0.0000	0.0000	0.0103	0.0086	0.0049	0.0000	0.0025	0.0000

DDR1_E23_R	0.0000	0.0000	0.0120	0.0000	0.0092	0.0000	0.0000	0.0091	0.0000	0.0096	0.0000	0.0055	0.0041	0.0000	0.0059	0.0062
DDR2_E331_F	0.2938	0.9931	0.9812	0.9855	0.9818	0.0064	0.0058	0.1779	0.9846	0.0000	0.0000	0.0000	0.9929	0.9785	0.9873	0.1877
DES_E228_R	0.0000	0.0000	0.0000	0.0061	0.0086	0.0000	0.0000	0.0000	0.0000	0.0000	0.0000	0.0000	0.0000	0.0000	0.0023	0.0070
DHCR24_P406_R	0.0244	0.0126	0.2321	0.0087	0.0164	0.3993	0.0150	0.8737	0.0000	0.7332	0.0124	0.0112	0.0138	0.0010	0.0109	0.0173
DIO3_E230_R	0.0000	0.0000	0.0143	0.0081	0.0102	0.0000	0.0059	0.0075	0.0000	0.0000	0.0000	0.0102	0.0076	0.0000	0.0003	0.0049
DIO3_P674_F	0.2828	0.0122	0.0162	0.0126	0.0212	0.3131	0.0076	0.9851	0.0000	0.3140	0.9964	0.0096	0.1779	0.2591	0.8976	0.9739
DIRAS3_E55_R	0.0188	0.3360	0.0276	0.0145	0.0469	0.0143	0.0123	0.0137	0.0000	0.0000	0.0000	0.0151	0.0000	0.0000	0.0207	0.0000
DKC1_E101_F	0.0719	0.0000	0.0011	0.0000	0.0000	0.0000	0.0000	0.0000	0.0000	0.0000	0.0000	0.0000	0.0000	0.0000	0.0000	0.0000
DKFZP564O0823_E45_F	0.0000	0.0000	0.0038	0.0000	0.0000	0.0000	0.0037	0.0009	0.0000	0.0000	0.0076	0.0000	0.0032	0.0000	0.0000	0.0000
DLC1_P88_R	0.0086	0.0000	0.0000	0.0000	0.0000	0.0000	0.0000	0.9250	0.0000	0.0000	0.0000	0.0000	0.0020	0.0000	0.0000	0.0000
DLK1_E227_R	0.9660	0.9973	0.9966	0.9971	0.9860	0.9967	0.9775	0.9848	0.9950	0.9969	0.9980	0.9966	0.9975	0.9941	0.9940	0.8344
DLL1_P386_F	0.0000	0.0000	0.9744	0.9887	0.9779	0.0000	0.0000	0.9384	0.0000	0.0000	0.0093	0.0000	0.0049	0.8036	0.9777	0.9869
DLL1_P832_F	0.0090	0.0082	0.4221	0.0101	0.0024	0.0000	0.0000	0.0097	0.0000	0.0000	0.0000	0.0051	0.0000	0.2892	0.0026	0.0039
DMP1_P134_F	0.0191	0.0232	0.0230	0.2381	0.1500	0.2134	0.0208	0.0216	0.0000	0.0101	0.1336	0.1686	0.0124	0.0096	0.0270	0.2151
DNAJC15_E26_R	0.0458	0.0063	0.1035	0.0083	0.2735	0.0044	0.0043	0.0131	0.0000	0.0000	0.0291	0.0058	0.0000	0.0000	0.0116	0.0042
DNASE1L1_P108_F	0.6359	0.5875	0.0388	0.7504	0.9886	0.9938	0.2253	0.9888	0.8609	0.6161	0.6372	0.8114	0.4558	0.9306	0.9188	0.5784
DNMT1_P100_R	0.9951	0.9925	0.9823	0.9904	0.9876	0.3981	0.8056	0.9906	0.9872	0.1485	0.3975	0.9418	0.9927	0.9888	0.9817	0.9909
DSC2_E90_F	0.0000	0.0000	0.0000	0.9704	0.9457	0.0000	0.7441	0.9802	0.9702	0.6322	0.0000	0.0000	0.0000	0.9250	0.8810	0.0000
DSP_P440_R	0.5261	0.4328	0.0341	0.6630	0.9853	0.6915	0.0154	0.4029	0.9882	0.5531	0.9979	0.3873	0.3748	0.9901	0.9822	0.7562
DST_E31_F	0.0118	0.8558	0.3051	0.0042	0.0555	0.9825	0.0066	0.3760	0.6287	0.1970	0.9964	0.7556	0.1558	0.9037	0.9844	0.9927
DST_P262_R	0.1633	0.9138	0.0950	0.0992	0.8361	0.4614	0.0440	0.1076	0.0349	0.0267	0.6472	0.0315	0.8907	0.0774	0.2899	0.8989
DUSP4_E61_F	0.0000	0.0000	0.0000	0.0695	0.0066	0.1681	0.0066	0.0074	0.0000	0.0098	0.0000	0.0047	0.0000	0.0000	0.0037	0.0375
DUSP4_P925_R	0.1874	0.0919	0.0339	0.6393	0.0343	0.0145	0.0233	0.6313	0.0000	0.0000	0.2211	0.0249	0.0071	0.0001	0.0405	0.4408
E2F3_P840_R	0.0184	0.0053	0.9747	0.0062	0.0000	0.0051	0.0000	0.0084	0.0000	0.0000	0.0071	0.0063	0.0000	0.7655	0.4736	0.0024
E2F5_P516_R	0.9819	0.0721	0.1610	0.9696	0.0670	0.0219	0.9728	0.9798	0.0000	0.0000	0.0000	0.9866	0.9813	0.9440	0.9704	0.9522
EDN1_E50_R	0.9705	0.9905	0.6079	0.5319	0.9883	0.9841	0.9772	0.2410	0.5995	0.7557	0.9623	0.5884	0.9884	0.3207	0.8629	0.8821
EDN1_P39_R	0.9531	0.0266	0.4669	0.9575	0.6763	0.0822	0.4280	0.0200	0.0000	0.0099	0.0177	0.9439	0.7179	0.0000	0.0129	0.0217
EDNRB_P148_R	0.7224	0.9920	0.4155	0.0000	0.4317	0.0000	0.9080	0.9727	0.9903	0.9937	0.0000	0.9885	0.0000	0.8138	0.9660	0.9895
EFNA1_P591_R	0.1073	0.1419	0.0000	0.0000	0.0000	0.0000	0.0000	0.0078	0.0000	0.3582	0.0156	0.0117	0.1608	0.0000	0.0000	0.0000
EFNA1_P7_F	0.0000	0.9932	0.6847	0.0000	0.0000	0.0000	0.0000	0.9551	0.9920	0.6785	0.0000	0.0017	0.0000	0.0000	0.0000	0.6370
EFNB1_E69_F	0.0000	0.0119	0.0083	0.0000	0.0000	0.0071	0.0047	0.1236	0.0000	0.0000	0.0000	0.0109	0.0000	0.0000	0.0000	0.9926
EFNB3_P442_R	0.0095	0.0055	0.0106	0.0000	0.0000	0.0000	0.0000	0.0062	0.0000	0.0000	0.0000	0.0000	0.0069	0.0000	0.0000	0.0000
EGF_E339_F	0.0000	0.0000	0.0035	0.0004	0.0000	0.9492	0.0047	0.8216	0.0000	0.0000	0.0000	0.0016	0.0000	0.0000	0.5534	0.0000

EGFR_E295_R	0.0309	0.1900	0.0114	0.0065	0.0067	0.0000	0.0000	0.0072	0.0000	0.0099	0.0122	0.0058	0.0056	0.0000	0.0050	0.0000
EGFR_P260_R	0.0026	0.6599	0.0060	0.0000	0.9830	0.0015	0.0000	0.0000	0.0000	0.0000	0.0085	0.0050	0.0000	0.5428	0.0000	0.9900
EGR4_P479_F	0.9870	0.7548	0.5235	0.9885	0.8556	0.5667	0.0754	0.3479	0.9862	0.0193	0.3915	0.4246	0.9941	0.8849	0.8303	0.0189
EIF2AK2_E103_R	0.0000	0.0000	0.0000	0.0000	0.0000	0.0000	0.0000	0.9842	0.9903	0.9965	0.0000	0.0000	0.0000	0.9726	0.9710	0.0000
EIF2AK2_P313_F	0.0000	0.9943	0.9920	0.9920	0.7791	0.0000	0.2910	0.8998	0.0000	0.0414	0.0074	0.9920	0.4150	0.9768	0.9888	0.9909
ELK1_E156_F	0.0000	0.9924	0.0000	0.0000	0.7915	0.0000	0.0000	0.9765	0.0000	0.1076	0.0000	0.8118	0.0000	0.9051	0.9014	0.0000
EMR3_P39_R	0.2790	0.3465	0.4908	0.0163	0.5455	0.9925	0.8181	0.4505	0.0000	0.0193	0.2639	0.9848	0.9437	0.5177	0.0277	0.3111
ENC1_P484_R	0.9941	0.8210	0.9829	0.9866	0.9818	0.0148	0.9900	0.7029	0.9877	0.0000	0.0164	0.9937	0.9930	0.9894	0.9867	0.1831
EPHA1_E46_R	0.2444	0.0409	0.3886	0.0361	0.0794	0.1735	0.0140	0.3593	0.7365	0.0400	0.0453	0.0403	0.0249	0.6183	0.2558	0.0256
EPHA1_P119_R	0.0000	0.0081	0.0113	0.0067	0.4676	0.0000	0.3220	0.0055	0.4112	0.0000	0.0000	0.0000	0.0000	0.0000	0.0024	0.0040
EPHA2_P203_F	0.0000	0.0082	0.0067	0.0000	0.4293	0.0052	0.0050	0.0077	0.4778	0.0071	0.0107	0.9914	0.0049	0.2462	0.4695	0.0041
EPHA3_E156_R	0.0000	0.0000	0.0000	0.0000	0.0000	0.0013	0.0000	0.0000	0.0000	0.0000	0.0051	0.0012	0.0000	0.0000	0.0000	0.0000
EPHA7_E6_F	0.0106	0.0234	0.1523	0.9928	0.0063	0.0035	0.0040	0.3096	0.9881	0.0062	0.0000	0.0040	0.0055	0.4446	0.3497	0.0032
EPHA7_P205_R	0.9830	0.0327	0.9935	0.8774	0.9412	0.9896	0.9136	0.9878	0.9922	0.1778	0.9914	0.8961	0.9193	0.9901	0.9405	0.9916
EPHA8_P256_F	0.0191	0.0188	0.0266	0.0394	0.0430	0.1572	0.0126	0.0963	0.0000	0.1063	0.1399	0.0231	0.1587	0.2348	0.1716	0.0122
EPHB1_P503_F	0.0111	0.0094	0.3500	0.0067	0.0093	0.6588	0.0085	0.7436	0.4885	0.0110	0.0000	0.9537	0.0076	0.1310	0.2275	0.6187
EPHB2_E297_F	0.8238	0.8403	0.7998	0.7489	0.8610	0.7252	0.7106	0.0474	0.0000	0.2180	0.7407	0.7601	0.7969	0.0000	0.0473	0.6938
EPHB2_P165_R	0.0000	0.0064	0.0079	0.0000	0.0025	0.0000	0.0000	0.9800	0.0000	0.0002	0.0000	0.0000	0.0380	0.0000	0.0000	0.0137
EPHB3_E0_F	0.0000	0.0000	0.9837	0.9884	0.6671	0.0000	0.0000	0.0000	0.0000	0.0000	0.0000	0.0000	0.0000	0.0000	0.0000	0.0011
EPHB3_P569_R	0.0219	0.1668	0.0289	0.3996	0.3493	0.4041	0.1855	0.0191	0.0000	0.1595	0.0244	0.4617	0.2989	0.0012	0.0135	0.2159
EPHB4_P313_R	0.5063	0.6452	0.0353	0.0389	0.0566	0.7051	0.0000	0.0447	0.3180	0.0750	0.0000	0.5168	0.1970	0.2945	0.9627	0.2807
EPHB6_E342_F	0.0123	0.1606	0.7550	0.0497	0.0302	0.8026	0.3286	0.7335	0.0000	0.0000	0.0129	0.5317	0.6316	0.0000	0.1128	0.0270
EPHB6_P827_R	0.0100	0.0000	0.0000	0.0003	0.0000	0.0000	0.0038	0.0172	0.0000	0.0000	0.0000	0.0020	0.0051	0.0000	0.0000	0.0000
EPHX1_P1358_R	0.0042	0.0155	0.0000	0.0000	0.0000	0.0000	0.0000	0.9732	0.0000	0.0059	0.0000	0.9898	0.0098	0.9484	0.9787	0.9896
EPM2A_P113_F	0.1009	0.0612	0.6970	0.1487	0.1106	0.8175	0.0413	0.6775	0.0000	0.8869	0.8988	0.8394	0.8228	0.0350	0.0825	0.7415
EPM2A_P64_R	0.0213	0.0162	0.0415	0.6001	0.0465	0.0138	0.3511	0.5580	0.0000	0.4539	0.4797	0.5087	0.4721	0.0000	0.0277	0.6070
EPO_E244_R	0.0021	0.0000	0.0048	0.0000	0.0000	0.0000	0.0000	0.0017	0.0000	0.0000	0.0000	0.0033	0.0000	0.0000	0.0000	0.0000
EPO_P162_R	0.0089	0.0000	0.4561	0.0117	0.0000	0.0000	0.0022	0.0047	0.0000	0.0000	0.0000	0.0012	0.0000	0.0000	0.6404	0.0006
EPS8_E231_F	0.1241	0.0146	0.0000	0.0000	0.9888	0.0000	0.0000	0.6589	0.0000	0.0000	0.0000	0.9880	0.9919	0.0000	0.9599	0.0000
EPS8_P437_F	0.0124	0.0031	0.0036	0.0000	0.0000	0.0027	0.0055	0.0000	0.0000	0.0000	0.0000	0.0000	0.0000	0.7370	0.0000	0.0000
ERBB2_P59_R	0.0000	0.0000	0.8190	0.0000	0.0014	0.0170	0.9930	0.0052	0.9508	0.0051	0.0000	0.9904	0.9934	0.8735	0.8443	0.9921
ERBB3_E331_F	0.5548	0.0194	0.0382	0.0285	0.6144	0.0120	0.9426	0.0165	0.0000	0.0185	0.6966	0.0305	0.5602	0.3705	0.0474	0.9915
ERBB3_P870_R	0.0000	0.1339	0.0756	0.2894	0.0190	0.0168	0.0000	0.0163	0.0000	0.0000	0.0000	0.0049	0.0115	0.3131	0.4297	0.0059

ERBB4_P255_F	0.0121	0.0093	0.0249	0.0146	0.0117	0.0063	0.1570	0.0192	0.0000	0.0069	0.0111	0.3607	0.5084	0.0000	0.0291	0.1824
ERBB4_P541_F	0.4220	0.1829	0.7653	0.2656	0.9883	0.9930	0.8947	0.9744	0.0000	0.0000	0.0000	0.2887	0.1601	0.8610	0.9640	0.3065
ERCC1_P354_F	0.9955	0.6670	0.9855	0.9857	0.7264	0.7736	0.6983	0.8438	0.7818	0.0000	0.0074	0.6973	0.0000	0.8810	0.4803	0.0044
ERCC3_P1210_R	0.9954	0.2576	0.7090	0.9907	0.0388	0.4760	0.2134	0.5742	0.9104	0.2628	0.3234	0.9806	0.1735	0.9781	0.9123	0.0154
ERG_E28_F	0.0413	0.8238	0.7535	0.5897	0.6068	0.0168	0.6261	0.6741	0.0000	0.0174	0.0441	0.6014	0.0252	0.0231	0.3506	0.0219
ERN1_P809_R	0.0688	0.0162	0.0767	0.0000	0.9839	0.0000	0.2883	0.0624	0.9900	0.0000	0.0460	0.9913	0.0046	0.9519	0.9770	0.0000
ESR1_E298_R	0.8114	0.0261	0.2042	0.7337	0.9898	0.7645	0.5256	0.9245	0.9223	0.7843	0.7980	0.7778	0.8576	0.8363	0.7154	0.0259
ESR1_P151_R	0.0000	0.0083	0.0117	0.0975	0.5272	0.0945	0.2498	0.7693	0.0000	0.0000	0.0000	0.0063	0.1222	0.6920	0.0012	0.1110
ESR2_E66_F	0.4040	0.4438	0.5369	0.0486	0.5293	0.3619	0.3026	0.0523	0.0162	0.4673	0.4424	0.1096	0.3667	0.6443	0.9738	0.3943
ESR2_P162_F	0.2814	0.1252	0.0291	0.0217	0.6859	0.3362	0.0158	0.6634	0.0000	0.3064	0.0191	0.0274	0.2579	0.0274	0.5081	0.7100
ETS1_E253_R	0.2353	0.1086	0.1962	0.0255	0.1073	0.1575	0.1319	0.1159	0.0000	0.0242	0.0851	0.1281	0.0159	0.0052	0.0340	0.1199
ETS1_P559_R	0.0000	0.0000	0.7067	0.0000	0.9847	0.0000	0.0000	0.9792	0.9901	0.0000	0.0078	0.0000	0.0000	0.9787	0.9815	0.5299
ETS2_P684_F	0.6942	0.6378	0.0258	0.9916	0.1107	0.7465	0.6822	0.0152	0.0000	0.5725	0.5979	0.0130	0.4885	0.9328	0.0913	0.0097
ETS2_P835_F	0.1790	0.0076	0.0208	0.0148	0.0100	0.0000	0.0000	0.1170	0.0000	0.0000	0.0319	0.0047	0.0000	0.0000	0.0000	0.0029
ETV1_P235_F	0.9922	0.9113	0.9870	0.8240	0.0252	0.9868	0.0068	0.8679	0.9733	0.0000	0.0000	0.9864	0.0025	0.9744	0.9759	0.0000
ETV1_P515_F	0.0000	0.0475	0.0000	0.0000	0.0000	0.2572	0.3088	0.0037	0.0000	0.0000	0.0000	0.0000	0.0482	0.0000	0.0000	0.3295
ETV6_E430_F	0.0138	0.0134	0.3812	0.0075	0.0152	0.0200	0.9098	0.5953	0.9484	0.0086	0.0000	0.0577	0.0103	0.0361	0.0095	0.0236
EVI1_E47_R	0.1423	0.0553	0.5328	0.0331	0.7724	0.0411	0.6399	0.9812	0.6834	0.5361	0.8043	0.5007	0.5531	0.9583	0.0433	0.9865
EVI1_P30_R	0.0064	0.0000	0.0093	0.3347	0.0096	0.0064	0.0050	0.0040	0.3509	0.0036	0.0000	0.0097	0.0047	0.0000	0.0048	0.0100
EVI2A_P94_R	0.0126	0.0082	0.0098	0.0055	0.0027	0.0059	0.0000	0.0023	0.0000	0.0000	0.0000	0.0055	0.0078	0.6358	0.0000	0.0039
EXT1_E197_F	0.2960	0.3301	0.6371	0.6858	0.3782	0.0142	0.5820	0.1185	0.0000	0.0136	0.4186	0.0138	0.0144	0.2559	0.3408	0.3374
EYA4_E277_F	0.1552	0.0032	0.0108	0.0047	0.5002	0.0055	0.0377	0.0078	0.0000	0.1534	0.2833	0.0119	0.0058	0.7150	0.0050	0.0031
EYA4_P508_F	0.0000	0.0000	0.0000	0.0000	0.9834	0.9838	0.4525	0.2997	0.0000	0.0000	0.0000	0.7142	0.0000	0.2291	0.9714	0.0026
EYA4_P794_F	0.0000	0.0000	0.0000	0.0000	0.0000	0.0000	0.2594	0.0000	0.9868	0.0000	0.0000	0.0000	0.0000	0.6189	0.5068	0.7250
F2R_P839_F	0.0000	0.0052	0.9849	0.9620	0.0000	0.0000	0.0000	0.8833	0.9838	0.0000	0.0000	0.9872	0.0000	0.9657	0.4916	0.0063
F2R_P88_F	0.3688	0.0121	0.4614	0.9886	0.0091	0.9912	0.0043	0.7623	0.9902	0.0000	0.0083	0.0059	0.7087	0.5062	0.9696	0.0018
FABP3_E113_F	0.0188	0.0131	0.0966	0.3664	0.3366	0.7322	0.0000	0.3199	0.4407	0.0000	0.0000	0.9897	0.0032	0.5844	0.5906	0.9887
FABP3_P598_F	0.9919	0.9379	0.5479	0.2546	0.8104	0.9808	0.9888	0.3803	0.5676	0.3364	0.5425	0.8119	0.3066	0.6286	0.3835	0.7139
FANCE_P356_R	0.2299	0.0777	0.0034	0.0000	0.0043	0.0000	0.9871	0.9672	0.0000	0.0000	0.0000	0.0000	0.0042	0.0000	0.4052	0.0018
FANCF_P13_F	0.7097	0.9879	0.7409	0.6836	0.7853	0.6429	0.6297	0.7689	0.0000	0.6628	0.7314	0.0906	0.6647	0.2221	0.9619	0.5598
FANCG_E207_R	0.9587	0.1525	0.1812	0.1293	0.2893	0.0368	0.1862	0.0505	0.0596	0.9634	0.1183	0.4317	0.1040	0.5233	0.2697	0.0554
FAS_P322_R	0.3940	0.2512	0.0000	0.0086	0.8520	0.9897	0.0000	0.0167	0.0000	0.0000	0.4141	0.0170	0.5873	0.4929	0.7120	0.9874
FASTK_P257_F	0.0872	0.0810	0.0897	0.0351	0.0366	0.0906	0.0960	0.0367	0.0107	0.0303	0.0229	0.7840	0.0989	0.0532	0.0558	0.0828

FAT_P973_R	0.0043	0.0000	0.0153	0.9762	0.0000	0.0960	0.0087	0.2325	0.0000	0.0000	0.7301	0.2326	0.5439	0.0000	0.7873	0.0092
FER_P581_F	0.9923	0.9949	0.4975	0.2675	0.2757	0.2735	0.0420	0.8731	0.9913	0.1603	0.2357	0.9018	0.2368	0.9668	0.7896	0.9025
FES_P223_R	0.0000	0.9916	0.0000	0.0000	0.9764	0.9875	0.0000	0.9696	0.9887	0.0000	0.0000	0.0000	0.0000	0.9389	0.9569	0.9901
FGF1_E5_F	0.9100	0.3057	0.0654	0.3085	0.8358	0.8745	0.9066	0.9242	0.9155	0.2832	0.3600	0.4630	0.3606	0.8722	0.9829	0.1650
FGF12_E61_R	0.2450	0.8311	0.0209	0.5770	0.2813	0.5185	0.2374	0.0250	0.0000	0.3484	0.0078	0.0087	0.3423	0.0000	0.0397	0.0080
FGF12_P210_R	0.3886	0.3796	0.9835	0.7039	0.0153	0.9921	0.9894	0.9327	0.9893	0.9848	0.3961	0.0788	0.9959	0.9027	0.9871	0.9883
FGF2_P153_F	0.2905	0.1558	0.0403	0.0154	0.0533	0.2097	0.1568	0.0278	0.7735	0.9844	0.2664	0.1373	0.1503	0.0132	0.0987	0.0187
FGF2_P229_F	0.5377	0.0518	0.5185	0.0407	0.0672	0.4264	0.0339	0.5905	0.0034	0.4412	0.5379	0.3655	0.0310	0.0771	0.0747	0.0480
FGF3_E198_R	0.9921	0.9902	0.7581	0.9836	0.9910	0.9842	0.9862	0.9722	0.9837	0.9931	0.9938	0.5650	0.9889	0.9772	0.9670	0.9843
FGF3_P171_R	0.0008	0.0048	0.7178	0.9102	0.5417	0.9914	0.5193	0.8926	0.7570	0.6529	0.0000	0.9355	0.9291	0.9022	0.9859	0.9039
FGF5_E16_F	0.9767	0.8144	0.9833	0.9906	0.9872	0.0000	0.0000	0.9829	0.9852	0.0000	0.0000	0.0038	0.0000	0.9886	0.7322	0.9898
FGF5_P238_R	0.0102	0.7066	0.7647	0.0000	0.0160	0.8858	0.0000	0.8792	0.0000	0.0099	0.0102	0.6678	0.0034	0.6465	0.5796	0.9259
FGF6_E294_F	0.0924	0.7534	0.0504	0.0513	0.0814	0.7012	0.9901	0.0409	0.0000	0.0522	0.8179	0.9904	0.7739	0.0045	0.1860	0.7248
FGF7_P44_F	0.0000	0.0000	0.0071	0.0000	0.0007	0.0023	0.0031	0.7259	0.0000	0.0000	0.0000	0.0023	0.0000	0.0000	0.0014	0.5595
FGF8_E183_F	0.2339	0.2841	0.1169	0.2601	0.0054	0.0228	0.1289	0.0306	0.0000	0.0000	0.0709	0.1549	0.1112	0.0000	0.9216	0.0494
FGF8_P473_F	0.0000	0.0003	0.0000	0.0000	0.0000	0.0000	0.0000	0.7961	0.0000	0.0000	0.0000	0.0000	0.0000	0.8172	0.0000	0.0556
FGFR1_E317_F	0.0692	0.0155	0.0153	0.0093	0.0000	0.0153	0.1271	0.0178	0.0000	0.0000	0.2684	0.2677	0.0000	0.0000	0.0322	0.4877
FGFR2_P266_R	0.1693	0.4403	0.0148	0.0094	0.0113	0.1873	0.0079	0.0130	0.0000	0.2944	0.1885	0.0055	0.2675	0.0000	0.0089	0.0069
FGFR3_E297_R	0.0000	0.0000	0.9775	0.0000	0.0000	0.0000	0.0000	0.0072	0.9888	0.0000	0.0977	0.0000	0.0000	0.7644	0.9133	0.9855
FGFR3_P1152_R	0.0000	0.8071	0.7063	0.8125	0.6868	0.5871	0.9920	0.9075	0.6624	0.9950	0.7013	0.5426	0.9939	0.9249	0.9455	0.7480
FHIT_E19_R	0.0164	0.0000	0.0000	0.7129	0.9842	0.9906	0.0000	0.9788	0.9894	0.0093	0.0000	0.9875	0.0000	0.8761	0.8604	0.0042
FHIT_P93_R	0.0114	0.0000	0.0141	0.0062	0.0093	0.0065	0.0032	0.3610	0.9901	0.0000	0.0000	0.0050	0.0050	0.0000	0.0070	0.0000
FHL1_E229_R	0.0000	0.0000	0.0000	0.0026	0.0000	0.0000	0.0018	0.0022	0.0000	0.0000	0.0051	0.0000	0.0000	0.0000	0.9808	0.4846
FLI1_P620_R	0.0000	0.4947	0.0000	0.9855	0.0000	0.0356	0.0000	0.1785	0.0000	0.0000	0.9922	0.9804	0.2074	0.9018	0.2374	0.9852
FLT1_E444_F	0.5960	0.0264	0.0348	0.9869	0.0730	0.5776	0.0148	0.5266	0.0000	0.8144	0.8372	0.0892	0.0321	0.5324	0.5687	0.9902
FLT1_P302_F	0.0581	0.0265	0.4970	0.3938	0.0412	0.0281	0.1384	0.0465	0.0000	0.2821	0.0184	0.0264	0.2789	0.0088	0.0558	0.0302
FLT1_P615_R	0.1352	0.0968	0.0232	0.0084	0.0168	0.9922	0.0967	0.3822	0.0000	0.1462	0.1701	0.0112	0.1326	0.0000	0.0190	0.0860
FLT3_E326_R	0.0135	0.0089	0.9857	0.0139	0.6233	0.0110	0.0114	0.0103	0.4318	0.0000	0.0115	0.1825	0.0068	0.2734	0.0041	0.0128
FLT4_P180_R	0.0239	0.1528	0.9845	0.9902	0.9643	0.9913	0.9410	0.9690	0.9900	0.0235	0.0658	0.9465	0.9802	0.6167	0.9816	0.1396
FMR1_P62_R	0.6087	0.9379	0.6828	0.9345	0.7033	0.4515	0.8859	0.8292	0.3925	0.9332	0.9246	0.4240	0.4567	0.4307	0.4201	0.5223
FN1_E469_F	0.0083	0.0000	0.0072	0.0005	0.0032	0.0000	0.0043	0.3278	0.0000	0.0000	0.0000	0.0000	0.0017	0.0000	0.0000	0.0009
FN1_P229_R	0.0088	0.0110	0.0413	0.0092	0.1375	0.0053	0.0167	0.0194	0.3654	0.2262	0.2961	0.0257	0.2855	0.0072	0.0296	0.0057
FOSL2_E384_R	0.8051	0.9925	0.1844	0.0726	0.9851	0.9934	0.9925	0.9461	0.9851	0.0449	0.0000	0.8761	0.0723	0.9879	0.8634	0.9914

FRK_P36_F	0.0167	0.1492	0.0456	0.7755	0.0095	0.1337	0.0767	0.0186	0.5666	0.0124	0.0000	0.0116	0.3353	0.0000	0.0086	0.1002
FRZB_E186_R	0.0000	0.0115	0.0000	0.0000	0.0000	0.1215	0.0000	0.7519	0.8858	0.0000	0.0000	0.9814	0.1513	0.0000	0.9223	0.1011
FRZB_P406_F	0.0099	0.0040	0.0000	0.0000	0.7203	0.0085	0.0000	0.0113	0.0000	0.0024	0.0000	0.0000	0.0000	0.0000	0.0023	0.0000
FVT1_P225_F	0.0105	0.7965	0.0105	0.0124	0.0022	0.9837	0.0106	0.0000	0.0000	0.0388	0.0106	0.0912	0.9933	0.0000	0.0026	0.0000
FYN_P352_R	0.9955	0.9948	0.9876	0.9323	0.9958	0.9937	0.9952	0.8723	0.9915	0.9948	0.9970	0.9927	0.9939	0.5848	0.9705	0.9922
FZD7_E296_F	0.0424	0.9929	0.9870	0.0000	0.9803	0.0000	0.9865	0.8742	0.9802	0.0000	0.0000	0.9830	0.0000	0.9769	0.9784	0.0000
FZD9_E458_F	0.0000	0.0000	0.9825	0.0000	0.0000	0.9900	0.0000	0.9703	0.9842	0.0000	0.9955	0.0000	0.9922	0.9720	0.9734	0.0000
FZD9_P15_R	0.0544	0.0115	0.0068	0.0043	0.0000	0.0000	0.0049	0.0032	0.0000	0.6046	0.2128	0.0000	0.0000	0.0000	0.0000	0.0021
FZD9_P175_F	0.0203	0.0397	0.8042	0.0106	0.0946	0.6277	0.0898	0.4392	0.0000	0.0127	0.1915	0.1498	0.1226	0.3038	0.7821	0.0095
G6PD_E190_F	0.0000	0.0000	0.8999	0.0022	0.0000	0.0021	0.0008	0.0000	0.0000	0.0000	0.0000	0.0000	0.0000	0.0000	0.0408	0.0000
G6PD_P196_F	0.7172	0.0108	0.7027	0.9923	0.2062	0.5333	0.9921	0.0214	0.0000	0.9834	0.6684	0.6106	0.6447	0.0000	0.0414	0.4499
GABRB3_E42_F	0.1948	0.0000	0.0000	0.0000	0.0000	0.0000	0.0000	0.0033	0.0000	0.0029	0.0000	0.0053	0.0000	0.0000	0.2016	0.0851
GADD45A_P737_R	0.3571	0.4332	0.0310	0.0332	0.5445	0.0147	0.0207	0.0249	0.0000	0.4141	0.0349	0.0368	0.0258	0.0123	0.3903	0.0251
GALR1_E52_F	0.0141	0.2883	0.0197	0.3111	0.3303	0.0140	0.0770	0.8389	0.0000	0.1340	0.0000	0.0106	0.0105	0.0000	0.0271	0.0000
GALR1_P80_F	0.0276	0.0100	0.0102	0.0053	0.0357	0.0072	0.0000	0.0095	0.0000	0.0000	0.0000	0.3416	0.0517	0.0000	0.0201	0.0000
GAS1_E22_F	0.1917	0.0087	0.0138	0.0126	0.0153	0.0580	0.0000	0.0102	0.0000	0.1495	0.0738	0.0062	0.0073	0.0000	0.0153	0.2018
GAS1_P754_R	0.0000	0.0000	0.0000	0.0000	0.0000	0.0000	0.0018	0.0067	0.0000	0.0000	0.0000	0.0028	0.0027	0.0000	0.0000	0.0023
GAS7_E148_F	0.0244	0.0204	0.0108	0.0180	0.0785	0.0135	0.4305	0.0112	0.0000	0.0401	0.0103	0.0208	0.0071	0.7612	0.0800	0.0076
GAS7_P622_R	0.0105	0.0000	0.0000	0.0000	0.0000	0.0000	0.0000	0.0000	0.0000	0.0000	0.0000	0.0000	0.0000	0.0000	0.0000	0.0000
GATA6_P21_R	0.0082	0.0088	0.1187	0.0012	0.0078	0.0000	0.0027	0.0047	0.0000	0.0000	0.0052	0.0070	0.0027	0.2699	0.0057	0.0021
GATA6_P726_F	0.1177	0.2036	0.0331	0.5017	0.2956	0.4931	0.1776	0.0152	0.0000	0.3439	0.5340	0.0130	0.3187	0.0000	0.6669	0.3827
GFI1_E136_F	0.2030	0.0000	0.0000	0.0854	0.0000	0.0056	0.1358	0.0096	0.0000	0.9943	0.0000	0.1782	0.0000	0.0000	0.0000	0.0913
GFI1_P45_R	0.0091	0.0014	0.0068	0.0000	0.7253	0.0000	0.0041	0.0091	0.0000	0.0059	0.0115	0.0053	0.0083	0.0000	0.1587	0.0057
GJB2_E43_F	0.2437	0.0000	0.0000	0.0000	0.0000	0.0000	0.0000	0.0116	0.0000	0.0043	0.0000	0.0000	0.0000	0.0000	0.0000	0.0056
GJB2_P791_R	0.1926	0.0146	0.0146	0.0148	0.0277	0.6677	0.1382	0.0286	0.0000	0.0177	0.2199	0.0900	0.1581	0.0058	0.2118	0.0865
GJB2_P931_R	0.3338	0.2931	0.3398	0.3545	0.3541	0.2736	0.2496	0.0422	0.0000	0.3082	0.0458	0.2960	0.3113	0.0250	0.0560	0.2771
GLA_P112_F	0.4655	0.8945	0.6649	0.0379	0.0000	0.4134	0.8552	0.9837	0.0000	0.0097	0.0000	0.2003	0.3111	0.0000	0.0154	0.0207
GLI2_E90_F	0.0000	0.0000	0.0000	0.9900	0.9654	0.0000	0.9890	0.9868	0.9860	0.0000	0.0000	0.9923	0.0000	0.9888	0.9845	0.9891
GLI3_E148_R	0.9764	0.1369	0.0325	0.4370	0.9294	0.9923	0.8044	0.8458	0.7210	0.9791	0.9821	0.5262	0.0824	0.8635	0.9860	0.9836
GML_P281_R	0.8755	0.0000	0.2198	0.9874	0.9891	0.0000	0.9908	0.9799	0.0000	0.9951	0.0000	0.0000	0.0000	0.6778	0.9746	0.0000
GNAS_E58_F	0.0129	0.0159	0.6836	0.0000	0.0181	0.9719	0.0048	0.5785	0.0000	0.0070	0.0000	0.0038	0.0000	0.9860	0.9821	0.0008
GP1BB_E23_F	0.0934	0.0074	0.0165	0.0090	0.0063	0.0097	0.0063	0.0106	0.0000	0.0088	0.0111	0.0060	0.0083	0.0631	0.0093	0.0069
GPC3_P235_R	0.0000	0.0046	0.6465	0.0000	0.0000	0.0000	0.0000	0.0077	0.9865	0.9954	0.0000	0.0000	0.0000	0.0000	0.0021	0.0000

GPR116_E328_R	0.9939	0.0000	0.9900	0.9918	0.9821	0.9928	0.0000	0.9553	0.9915	0.9916	0.9968	0.0000	0.9930	0.9906	0.9850	0.9919
GPX1_E46_R	0.1820	0.0077	0.1028	0.0107	0.0068	0.1712	0.0000	0.0088	0.0000	0.0093	0.0000	0.0000	0.0038	0.0000	0.0837	0.0083
GPX1_P194_F	0.6452	0.6829	0.7829	0.0356	0.1060	0.0302	0.4291	0.0268	0.0000	0.4273	0.6859	0.0417	0.5376	0.0022	0.0703	0.5725
GPX3_E178_F	0.0099	0.0121	0.0167	0.0112	0.1023	0.0085	0.9773	0.7336	0.0000	0.1108	0.1628	0.0090	0.0135	0.0050	0.2079	0.0060
GRB10_E85_R	0.0165	0.0151	0.0123	0.0101	0.0009	0.0053	0.0000	0.0096	0.0000	0.0102	0.0000	0.0000	0.0000	0.2954	0.0000	0.0000
GRB10_P260_F	0.0098	0.0207	0.0071	0.0000	0.0065	0.0091	0.0600	0.0158	0.0000	0.0000	0.9896	0.0104	0.0040	0.0000	0.0000	0.0135
GRB10_P496_R	0.0000	0.0186	0.0203	0.0071	0.4450	0.1766	0.2177	0.0074	0.0000	0.0097	0.0095	0.0891	0.1383	0.0000	0.0000	0.0225
GRPR_P200_R	0.1330	0.1418	0.9852	0.9883	0.9391	0.0125	0.9742	0.9407	0.0429	0.3745	0.1989	0.9841	0.1751	0.5254	0.9682	0.1566
GSTM1_P363_F	0.0000	0.0068	0.6525	0.1006	0.9860	0.0114	0.0087	0.6776	0.0000	0.0821	0.1258	0.0608	0.0118	0.0000	0.0217	0.0000
GSTM2_E153_F	0.0092	0.0049	0.4823	0.0051	0.0060	0.0041	0.0000	0.0000	0.0000	0.0000	0.0000	0.0063	0.0049	0.0382	0.3059	0.0000
GSTM2_P109_R	0.0087	0.0000	0.0069	0.0007	0.0000	0.0044	0.0000	0.0066	0.0000	0.0018	0.0088	0.0021	0.0000	0.0000	0.0000	0.0000
GSTM2_P453_R	0.0000	0.0117	0.0128	0.0000	0.5224	0.0000	0.9872	0.9724	0.8663	0.9937	0.0000	0.0020	0.0000	0.8486	0.2160	0.0000
GSTP1_E322_R	0.3909	0.0000	0.0118	0.0009	0.0000	0.1399	0.0000	0.0105	0.0000	0.0129	0.0825	0.0018	0.0060	0.7493	0.0000	0.0104
GSTP1_P74_F	0.0320	0.0402	0.4472	0.5271	0.0211	0.0201	0.2103	0.0258	0.0000	0.0222	0.1297	0.3259	0.1615	0.0000	0.0502	0.0275
GSTP1_seq_38_S153_R	0.0000	0.0143	0.0165	0.0048	0.0111	0.0086	0.0000	0.0105	0.0000	0.0000	0.0000	0.0000	0.0092	0.0000	0.0047	0.0000
GUCY2D_E419_R	0.0092	0.0056	0.0000	0.0054	0.0046	0.0000	0.0066	0.4420	0.0000	0.0000	0.0000	0.0000	0.0049	0.0000	0.0032	0.0000
HBEGF_P32_R	0.0000	0.0000	0.0042	0.0000	0.0000	0.0019	0.0052	0.0046	0.0000	0.0000	0.0000	0.0000	0.0000	0.0000	0.0001	0.0044
HBII-13_E48_F	0.0000	0.0000	0.0000	0.9776	0.0000	0.9890	0.0030	0.8648	0.0000	0.0000	0.0000	0.0000	0.0000	0.9622	0.9805	0.9857
HBII-52_P563_F	0.0572	0.7240	0.1340	0.7464	0.0498	0.0441	0.0384	0.9800	0.9838	0.6051	0.6524	0.8522	0.6314	0.6400	0.1533	0.4479
HCK_P46_R	0.0134	0.0000	0.1119	0.0084	0.0000	0.2822	0.3112	0.0172	0.0000	0.0088	0.0000	0.0207	0.0040	0.0000	0.0074	0.0078
HCK_P858_F	0.0733	0.6071	0.0677	0.0278	0.1014	0.5806	0.5100	0.0167	0.0000	0.4959	0.0638	0.0399	0.0761	0.0000	0.0665	0.0487
HDAC1_P414_R	0.0128	0.0000	0.0108	0.0038	0.4367	0.0105	0.0109	0.0044	0.9883	0.0098	0.0103	0.0000	0.0115	0.2614	0.0000	0.0053
HDAC11_P556_F	0.0000	0.0000	0.0000	0.0000	0.0048	0.0062	0.0000	0.0035	0.0000	0.0000	0.0147	0.0060	0.0000	0.0000	0.0000	0.0000
HDAC9_P137_R	0.7570	0.0013	0.0001	0.0000	0.0000	0.0000	0.0024	0.0014	0.0000	0.0000	0.0000	0.0011	0.0025	0.1382	0.0015	0.0029
HFE_E273_R	0.0089	0.0091	0.0045	0.0034	0.0051	0.0028	0.0048	0.0030	0.0000	0.0103	0.0130	0.0000	0.0062	0.0000	0.0003	0.0000
HHIP_P307_R	0.1760	0.1047	0.1315	0.0266	0.2886	0.0241	0.0334	0.0379	0.0003	0.0212	0.1159	0.0245	0.1811	0.0129	0.0313	0.1034
HIC1_E151_F	0.0000	0.0000	0.0000	0.0000	0.3268	0.0000	0.0000	0.0000	0.0000	0.0000	0.0000	0.0000	0.0016	0.0000	0.0000	0.0000
HIC1_P565_R	0.0000	0.0000	0.0124	0.0220	0.0000	0.0000	0.0000	0.1319	0.0000	0.0000	0.0000	0.0123	0.0000	0.0000	0.0089	0.0037
HIC2_P498_F	0.0000	0.0077	0.0000	0.0046	0.0000	0.0000	0.0000	0.0054	0.0000	0.0036	0.9383	0.0000	0.0081	0.0000	0.0000	0.0059
HIC2_P528_R	0.0181	0.0000	0.0132	0.0113	0.0000	0.0055	0.0107	0.0000	0.0000	0.0205	0.0236	0.0000	0.0199	0.0000	0.0056	0.0093
HIF1A_P488_F	0.0000	0.0000	0.0000	0.7994	0.0000	0.0102	0.0000	0.0113	0.0000	0.0102	0.0000	0.0046	0.0042	0.0000	0.0000	0.0038
HLA-DOB_P357_R	0.9909	0.4957	0.0000	0.9857	0.8106	0.9893	0.9822	0.9833	0.0000	0.9935	0.0000	0.0000	0.9927	0.9743	0.9696	0.9862
HLA-DPA1_P205_R	0.0197	0.0117	0.0220	0.8415	0.6860	0.0056	0.1128	0.0150	0.1205	0.0105	0.1295	0.1081	0.0880	0.0000	0.6018	0.0070

HLA-DPA1_P28_R	0.0000	0.0000	0.0121	0.0068	0.0121	0.0076	0.0049	0.0032	0.0000	0.0000	0.0000	0.0047	0.0000	0.0000	0.0080	0.0041
HLA-DPB1_E2_R	0.0000	0.0000	0.1182	0.0146	0.0547	0.1711	0.5683	0.0225	0.0000	0.0000	0.5647	0.0364	0.4274	0.0000	0.1256	0.4377
HLA-DRA_P132_R	0.0013	0.0000	0.0000	0.0000	0.0000	0.0000	0.0000	0.3911	0.0000	0.0000	0.0000	0.8425	0.0000	0.0000	0.0000	0.0000
HLA-F_E402_F	0.0096	0.0000	0.0000	0.0000	0.0000	0.0000	0.0006	0.0000	0.0000	0.0000	0.0000	0.0000	0.0000	0.0000	0.0000	0.0000
HLF_E192_F	0.0443	0.0255	0.0772	0.0234	0.5573	0.7527	0.0224	0.0217	0.0000	0.6635	0.7068	0.5911	0.0288	0.0080	0.0329	0.0140
HOXA11_E35_F	0.0116	0.0053	0.0000	0.0047	0.0044	0.0052	0.0035	0.0045	0.0000	0.0040	0.0000	0.0065	0.0000	0.0000	0.0000	0.0046
HOXA11_P698_F	0.6991	0.0411	0.0589	0.0138	0.0253	0.0226	0.5934	0.0337	0.0000	0.7792	0.7138	0.0247	0.0210	0.0000	0.7809	0.5808
HOXA5_E187_F	0.0000	0.0000	0.9898	0.0000	0.6539	0.0055	0.0000	0.9493	0.9657	0.0074	0.9960	0.9901	0.0032	0.9496	0.0000	0.9897
HOXA9_E252_R	0.0134	0.1088	0.0499	0.0289	0.0644	0.0000	0.0000	0.0155	0.0000	0.0000	0.0000	0.0074	0.0000	0.0000	0.1425	0.0000
HOXA9_P1141_R	0.1402	0.0144	0.0293	0.0247	0.0225	0.2351	0.0087	0.0202	0.0000	0.1148	0.0138	0.0142	0.0118	0.0099	0.0247	0.0215
HOXA9_P303_F	0.0000	0.0000	0.0015	0.0000	0.0000	0.0000	0.0000	0.0016	0.0000	0.0000	0.0000	0.0003	0.0006	0.0000	0.0000	0.0011
HOXB13_E21_F	0.0000	0.0048	0.0086	0.0030	0.0086	0.0000	0.0000	0.1059	0.0000	0.0057	0.0000	0.0042	0.0033	0.0000	0.3076	0.0000
HOXB13_P17_R	0.0000	0.0045	0.0032	0.0004	0.0000	0.0041	0.0000	0.0003	0.0000	0.0000	0.0000	0.0035	0.0000	0.0000	0.5104	0.0000
HOXC6_P456_R	0.5429	0.0464	0.0469	0.0481	0.6846	0.7242	0.0273	0.0369	0.0000	0.6471	0.0357	0.0410	0.0212	0.0310	0.0512	0.0248
HPN_P374_R	0.0000	0.0000	0.0224	0.0000	0.0187	0.0069	0.0000	0.0045	0.0000	0.0000	0.0000	0.0000	0.0000	0.0000	0.0000	0.0055
HPN_P823_F	0.0164	0.0415	0.7097	0.0000	0.0000	0.0007	0.0034	0.9755	0.0000	0.0000	0.0000	0.0000	0.0029	0.0000	0.6812	0.0000
HPSE_P29_F	0.0344	0.0380	0.6687	0.0199	0.0215	0.7404	0.0817	0.0365	0.0000	0.5620	0.5901	0.4180	0.9907	0.4775	0.0226	0.7253
HPSE_P93_F	0.0000	0.0062	0.0098	0.0058	0.0000	0.0053	0.0083	0.0065	0.0000	0.0484	0.0082	0.0067	0.0000	0.0000	0.0000	0.0042
HRASLS_E72_R	0.0000	0.0000	0.0027	0.0000	0.0000	0.0000	0.0000	0.0019	0.0000	0.0037	0.0000	0.0023	0.0000	0.0000	0.0000	0.0000
HS3ST2_E145_R	0.0000	0.0038	0.9790	0.0000	0.0000	0.0000	0.0000	0.0041	0.0000	0.0000	0.0000	0.0000	0.0000	0.0000	0.0000	0.0000
HS3ST2_P546_F	0.0137	0.0000	0.0000	0.0000	0.0000	0.0074	0.0064	0.0000	0.0000	0.0000	0.0000	0.0000	0.0000	0.0000	0.0000	0.0106
HSD17B12_E145_R	0.0412	0.4414	0.0177	0.0177	0.0249	0.0085	0.0163	0.0172	0.0000	0.0000	0.0000	0.0772	0.0231	0.0000	0.0176	0.0072
HTR1B_P222_F	0.0000	0.0000	0.0000	0.0034	0.0000	0.0000	0.0022	0.0000	0.0000	0.1648	0.0000	0.0000	0.0045	0.0000	0.0006	0.0000
HTR2A_P853_F	0.0159	0.0105	0.0000	0.0029	0.0072	0.0000	0.0028	0.0040	0.0000	0.9938	0.0112	0.0046	0.0060	0.0000	0.0000	0.0035
IAPP_E280_F	0.0000	0.0000	0.9863	0.9874	0.0000	0.9641	0.0000	0.9825	0.9876	0.0000	0.0000	0.9897	0.9946	0.9652	0.9845	0.0000
ICA1_P61_F	0.0000	0.0160	0.0000	0.0000	0.0000	0.0027	0.0000	0.0000	0.0000	0.0191	0.0000	0.0000	0.0000	0.0000	0.0000	0.0000
ICA1_P72_R	0.0000	0.0064	0.0000	0.0000	0.0021	0.0000	0.3631	0.0000	0.0000	0.0000	0.0000	0.0000	0.0000	0.2559	0.0000	0.0026
ICAM1_E242_F	0.2109	0.0104	0.7295	0.0144	0.0097	0.0090	0.1512	0.0149	0.0000	0.2995	0.1926	0.0119	0.7663	0.0000	0.0198	0.0067
ICAM1_P119_R	0.0000	0.0109	0.0000	0.0041	0.0000	0.0000	0.0046	0.0020	0.0000	0.0052	0.0000	0.0013	0.0061	0.0000	0.0000	0.0000
ICAM1_P386_R	0.0176	0.0073	0.0205	0.0000	0.0067	0.0000	0.0015	0.0000	0.0000	0.0000	0.0000	0.0061	0.0000	0.0000	0.0000	0.0000
ID1_P659_R	0.0000	0.0000	0.0064	0.0016	0.0000	0.0046	0.0000	0.6697	0.0000	0.0000	0.0090	0.0024	0.0000	0.0000	0.0023	0.0000
IFNG_P459_R	0.6841	0.2396	0.4673	0.9883	0.9843	0.0089	0.0106	0.6383	0.0000	0.2673	0.1511	0.9912	0.1883	0.7816	0.9810	0.9918
IFNGR1_P307_F	0.1493	0.0127	0.0181	0.0086	0.1294	0.0098	0.1426	0.0145	0.0000	0.1410	0.1615	0.0116	0.1711	0.0000	0.0087	0.0082

IFNGR2_E164_F	0.1001	0.0235	0.0896	0.1162	0.0644	0.1231	0.1142	0.0389	0.0000	0.0311	0.0186	0.0491	0.0406	0.0190	0.0379	0.0244
IFNGR2_P377_R	0.0078	0.0000	0.0036	0.0000	0.0008	0.0000	0.0014	0.0000	0.0000	0.0000	0.0000	0.0025	0.0000	0.0000	0.0000	0.9905
IGF1_E394_F	0.0000	0.0021	0.9874	0.9627	0.9815	0.0000	0.0000	0.6405	0.6338	0.0005	0.0000	0.0000	0.0000	0.8403	0.0000	0.0000
IGF1_P933_F	0.2549	0.3603	0.0407	0.9843	0.9831	0.0053	0.0000	0.9766	0.0000	0.0000	0.0040	0.0000	0.0039	0.7210	0.0000	0.0000
IGF1R_E186_R	0.0000	0.0072	0.0000	0.0000	0.0000	0.0000	0.0000	0.0017	0.0000	0.0046	0.0000	0.0013	0.0000	0.0000	0.0000	0.0000
IGF1R_P325_R	0.5276	0.1721	0.0207	0.0142	0.5844	0.2329	0.0094	0.4055	0.0000	0.4096	0.6756	0.4977	0.4259	0.0000	0.0353	0.3817
IGF2_E134_R	0.0000	0.0000	0.0005	0.0000	0.0000	0.0000	0.0000	0.0019	0.0000	0.0000	0.0021	0.0000	0.0000	0.0000	0.0000	0.0000
IGF2_P1036_R	0.0195	0.0182	0.0252	0.2056	0.0250	0.0158	0.0132	0.0474	0.0000	0.0110	0.1929	0.0210	0.1469	0.1673	0.0166	0.0552
IGF2_P36_R	0.0000	0.0000	0.0000	0.0138	0.0322	0.1168	0.0532	0.0110	0.0000	0.1521	0.0000	0.0707	0.2908	0.0000	0.0148	0.0074
IGF2AS_P203_F	0.0000	0.0000	0.0000	0.0000	0.0000	0.0000	0.0000	0.0000	0.0000	0.0000	0.0000	0.0000	0.0000	0.0000	0.0000	0.0000
IGF2R_P396_R	0.2782	0.8727	0.2728	0.9752	0.8011	0.9776	0.8206	0.6602	0.2163	0.2587	0.9511	0.2546	0.9497	0.4025	0.1879	0.2306
IGFBP1_E48_R	0.0784	0.0088	0.0233	0.2553	0.0842	0.0000	0.0070	0.0095	0.9398	0.0056	0.0000	0.0076	0.0000	0.0000	0.0556	0.0070
IGFBP1_P12_R	0.0149	0.2912	0.3762	0.0989	0.0202	0.1629	0.0000	0.0479	0.0000	0.3567	0.0122	0.0103	0.2244	0.0000	0.0091	0.1029
IGFBP2_P306_F	0.0097	0.0000	0.0060	0.0000	0.0000	0.0000	0.0005	0.0000	0.0000	0.0018	0.0053	0.0000	0.0066	0.0000	0.0000	0.0000
IGFBP3_E65_R	0.0170	0.5855	0.3725	0.3342	0.0236	0.0332	0.3524	0.0442	0.0000	0.2754	0.1447	0.0336	0.0051	0.0000	0.0409	0.3504
IGFBP3_P1035_F	0.0000	0.0000	0.0033	0.0000	0.0000	0.0000	0.0000	0.0032	0.0000	0.0000	0.0000	0.0000	0.0000	0.0000	0.0000	0.0021
IGFBP3_P423_R	0.0080	0.0953	0.0109	0.0070	0.0847	0.1713	0.1286	0.0158	0.0000	0.0958	0.0077	0.0057	0.1769	0.0000	0.0139	0.0203
IGFBP7_P371_F	0.0131	0.2824	0.0225	0.0043	0.0137	0.1676	0.2840	0.1563	0.0000	0.0058	0.1735	0.0074	0.1464	0.0000	0.0108	0.0000
IGSF4_P454_F	0.2631	0.0090	0.0177	0.0000	0.0062	0.0000	0.0016	0.0098	0.0000	0.0000	0.0000	0.0000	0.0092	0.0000	0.0000	0.0032
IGSF4_P86_R	0.1674	0.0127	0.5838	0.0202	0.0487	0.3574	0.1342	0.0165	0.0000	0.9865	0.1321	0.1850	0.5289	0.0083	0.0152	0.0094
IGSF4C_E65_F	0.0000	0.9956	0.6245	0.6929	0.7694	0.0035	0.0034	0.9875	0.0000	0.9954	0.0000	0.0000	0.0000	0.4265	0.9857	0.9929
IGSF4C_P533_R	0.0169	0.0000	0.0147	0.0000	0.0024	0.0000	0.0088	0.0140	0.0000	0.0477	0.0122	0.0000	0.0000	0.0000	0.2070	0.0074
IHH_E186_F	0.0000	0.0000	0.0000	0.0000	0.0000	0.0000	0.0000	0.0000	0.0000	0.0036	0.0959	0.0074	0.0000	0.0000	0.0000	0.0020
IHH_P246_R	0.0000	0.0041	0.9796	0.0000	0.0093	0.3693	0.0084	0.0093	0.0000	0.0134	0.0000	0.0150	0.0026	0.0000	0.0105	0.2555
IHH_P529_F	0.0000	0.1566	0.0000	0.0000	0.0094	0.0066	0.0000	0.0095	0.0000	0.0000	0.0000	0.0000	0.0000	0.0000	0.0000	0.0724
IL10_P85_F	0.0000	0.0000	0.0065	0.9868	0.0007	0.0000	0.0031	0.0033	0.0000	0.0000	0.0000	0.0000	0.0000	0.0000	0.5894	0.0000
IL11_P11_R	0.0000	0.8885	0.0000	0.0000	0.0000	0.0000	0.0000	0.0079	0.0000	0.0000	0.0000	0.0087	0.0000	0.6727	0.0000	0.0000
IL12A_E287_R	0.0110	0.0046	0.0062	0.0035	0.0035	0.0033	0.0001	0.0043	0.0000	0.0000	0.0000	0.0015	0.0164	0.0000	0.0435	0.0027
IL12B_P392_R	0.3054	0.0040	0.0010	0.0003	0.0374	0.0021	0.0816	0.4927	0.0000	0.0036	0.0050	0.0014	0.0255	0.4495	0.6656	0.2651
IL13_E75_R	0.9924	0.9933	0.9020	0.9881	0.9907	0.8535	0.0415	0.9822	0.8214	0.2449	0.6654	0.9367	0.7933	0.9899	0.9850	0.0909
IL17RB_E164_R	0.0000	0.0048	0.0000	0.0039	0.0000	0.0000	0.0112	0.0040	0.0000	0.0109	0.0000	0.0063	0.0092	0.0000	0.0000	0.0030
IL17RB_P788_R	0.0000	0.0000	0.0000	0.0000	0.0000	0.0000	0.0000	0.0005	0.0000	0.0000	0.0000	0.0000	0.0051	0.0000	0.0000	0.0000
IL18BP_P51_R	0.2996	0.0309	0.0446	0.0427	0.0737	0.0150	0.2402	0.0483	0.0000	0.2285	0.2807	0.0259	0.1382	0.0163	0.0469	0.0173

IL1A_E113_R	0.9941	0.9928	0.9518	0.9902	0.9916	0.9923	0.9938	0.9445	0.9091	0.0000	0.3153	0.0685	0.1198	0.9882	0.9886	0.6805
IL1RN_E42_F	0.0000	0.0000	0.0000	0.0000	0.0000	0.0000	0.5674	0.9884	0.8846	0.0001	0.0000	0.6980	0.7033	0.9705	0.9206	0.6557
IL3_P556_F	0.9947	0.0000	0.8322	0.0005	0.0000	0.9921	0.0017	0.9845	0.9887	0.9951	0.0000	0.0000	0.0000	0.9842	0.9825	0.9898
IL6_E168_F	0.3226	0.0104	0.0235	0.0000	0.2535	0.1606	0.0000	0.0126	0.0000	0.3462	0.2782	0.0596	0.1906	0.0000	0.0150	0.0052
IL8_E118_R	0.0061	0.0079	0.0088	0.0033	0.0000	0.0000	0.0000	0.0031	0.0000	0.0000	0.0000	0.0055	0.0527	0.0000	0.4328	0.0012
IL8_P83_F	0.0069	0.1513	0.0000	0.0000	0.8920	0.0000	0.0000	0.0000	0.0000	0.0000	0.0000	0.0000	0.0000	0.0000	0.0000	0.0000
IMPACT_P186_F	0.0000	0.0000	0.0000	0.0000	0.0000	0.0000	0.0000	0.0000	0.0000	0.0130	0.0000	0.0000	0.0000	0.0000	0.0000	0.0000
IMPACT_P234_R	0.0000	0.0000	0.0066	0.0000	0.0000	0.0000	0.0003	0.0059	0.0000	0.0025	0.0000	0.0000	0.0000	0.0000	0.0000	0.0000
INSR_P1063_R	0.0000	0.0000	0.0000	0.0000	0.0000	0.0000	0.0023	0.0000	0.0000	0.0000	0.0000	0.0000	0.0000	0.0000	0.0000	0.0000
IPF1_P234_F	0.0000	0.0000	0.0102	0.0000	0.0224	0.0000	0.0000	0.0054	0.0000	0.0000	0.0000	0.0000	0.0000	0.0000	0.0000	0.0040
IRAK1_P455_R	0.0137	0.7643	0.0114	0.0045	0.0003	0.0000	0.0106	0.0314	0.0000	0.0187	0.1279	0.0000	0.0051	0.0000	0.0068	0.0838
IRAK3_E130_F	0.0229	0.0068	0.0127	0.0031	0.0048	0.0000	0.0120	0.0099	0.0000	0.0047	0.0000	0.0003	0.0000	0.0000	0.0045	0.9869
IRAK3_P13_F	0.0000	0.2633	0.0000	0.0149	0.0000	0.0071	0.0150	0.0141	0.0000	0.1576	0.0000	0.0111	0.0105	0.0000	0.0093	0.0025
IRF5_P123_F	0.0152	0.0080	0.0204	0.0147	0.0066	0.0000	0.4981	0.0162	0.0000	0.0166	0.0094	0.0128	0.0028	0.3243	0.0159	0.0025
IRF7_P277_R	0.0273	0.1919	0.0264	0.0160	0.0212	0.0158	0.2100	0.0129	0.0000	0.3062	0.2132	0.0143	0.0136	0.0000	0.0178	0.0134
ISL1_E87_R	0.0000	0.0058	0.0135	0.0029	0.0021	0.0044	0.0000	0.0078	0.0000	0.0059	0.0103	0.0078	0.9929	0.0000	0.0000	0.0061
ISL1_P379_F	0.0000	0.0279	0.0119	0.0037	0.0072	0.0000	0.0023	0.0072	0.3996	0.0000	0.0095	0.0072	0.0000	0.0000	0.0023	0.0041
ISL1_P554_F	0.0000	0.0053	0.0030	0.0000	0.0000	0.0000	0.0000	0.0000	0.0000	0.0000	0.0032	0.0000	0.0000	0.0000	0.0000	0.0000
ITGA2_E120_F	0.0136	0.0067	0.0152	0.0106	0.0054	0.0100	0.0110	0.0122	0.0000	0.3747	0.0000	0.0163	0.1312	0.0000	0.3820	0.0000
ITGA2_P26_R	0.0000	0.0071	0.0000	0.0000	0.0037	0.0050	0.0044	0.0046	0.0000	0.0000	0.0146	0.0000	0.0050	0.0000	0.0000	0.0027
ITGB4_E144_F	0.0215	0.0115	0.0108	0.0086	0.0000	0.0000	0.0000	0.0107	0.0000	0.0175	0.0000	0.0000	0.0000	0.0000	0.0029	0.0000
ITGB4_P517_F	0.0054	0.0000	0.0132	0.0000	0.0000	0.0000	0.0000	0.0056	0.9587	0.0000	0.0000	0.0000	0.0000	0.0000	0.0000	0.0000
ITPR3_E86_R	0.0875	0.0000	0.0011	0.0000	0.0000	0.0000	0.0000	0.0021	0.0000	0.0000	0.0085	0.0000	0.0011	0.0000	0.0000	0.0050
ITPR3_P1112_F	0.0084	0.0053	0.0000	0.0017	0.0000	0.0038	0.0000	0.0002	0.0000	0.0000	0.0000	0.0000	0.0036	0.0000	0.0000	0.0021
JAG2_E54_F	0.0000	0.0058	0.0097	0.0000	0.0055	0.0234	0.0047	0.0078	0.0000	0.0000	0.0000	0.0078	0.0632	0.0000	0.0032	0.1179
JAG2_P264_F	0.0114	0.0044	0.3804	0.5022	0.0158	0.4521	0.2655	0.3719	0.0000	0.0000	0.0000	0.1999	0.0502	0.0000	0.0100	0.0062
JAK2_P772_R	0.0189	0.0139	0.2208	0.0831	0.0138	0.0000	0.0000	0.0129	0.0000	0.0000	0.0000	0.0204	0.0000	0.0000	0.0025	0.2798
JAK3_E64_F	0.0000	0.0119	0.0130	0.0041	0.0125	0.0046	0.0026	0.0107	0.0000	0.0094	0.0000	0.0081	0.0000	0.0000	0.3086	0.0079
JAK3_P156_R	0.0933	0.1367	0.0208	0.0101	0.0205	0.0079	0.0142	0.0143	0.0000	0.0105	0.0120	0.0106	0.1581	0.0012	0.0153	0.0081
JUNB_P1149_R	0.1268	0.5337	0.5564	0.0787	0.2296	0.4813	0.4966	0.0633	0.0355	0.5461	0.6164	0.4780	0.0803	0.1554	0.1747	0.4531
KCNK4_E3_F	0.0149	0.0000	0.0053	0.0037	0.0000	0.0000	0.0027	0.0093	0.0000	0.0000	0.0000	0.0000	0.0047	0.0000	0.5064	0.0046
KDR_E79_F	0.7809	0.0471	0.0717	0.0206	0.0897	0.7332	0.5941	0.0185	0.0000	0.0385	0.0501	0.7887	0.0334	0.0000	0.7197	0.0326
KDR_P445_R	0.1875	0.0000	0.0080	0.0000	0.0000	0.0043	0.2445	0.0116	0.0000	0.0000	0.0000	0.0074	0.0000	0.0332	0.0004	0.0041

KIAA1804_P689_R	0.0230	0.0096	0.2659	0.3038	0.0095	0.4218	0.1014	0.0185	0.0000	0.1411	0.0873	0.0098	0.1684	0.0000	0.0084	0.1380
KIT_P367_R	0.2305	0.0024	0.0127	0.0000	0.0037	0.0050	0.0079	0.0100	0.0000	0.0059	0.5167	0.0000	0.0000	0.0000	0.0000	0.0029
KIT_P405_F	0.0122	0.0063	0.3440	0.0000	0.0636	0.0073	0.0064	0.2836	0.0000	0.0068	0.0000	0.0080	0.0000	0.0000	0.0056	0.0042
KLF5_E190_R	0.9954	0.0058	0.0140	0.0020	0.0073	0.0000	0.0039	0.4932	0.0510	0.0042	0.0000	0.0030	0.0000	0.0000	0.0034	0.0000
KLK11_P103_R	0.9934	0.0000	0.9815	0.0000	0.0000	0.0000	0.9896	0.4846	0.0000	0.9835	0.0000	0.9891	0.0000	0.7791	0.9793	0.0000
KLK11_P1290_F	0.9933	0.1601	0.0074	0.0955	0.0000	0.0000	0.0000	0.0204	0.0000	0.0000	0.9943	0.0000	0.0000	0.9717	0.7223	0.0000
KRAS_E82_F	0.0113	0.0085	0.6765	0.8642	0.0025	0.9890	0.0068	0.6516	0.0000	0.9944	0.0120	0.7305	0.4340	0.8457	0.5373	0.0042
KRAS_P651_F	0.5212	0.1590	0.5481	0.0814	0.5770	0.1049	0.1145	0.1272	0.0997	0.1657	0.5132	0.4900	0.1515	0.1063	0.1655	0.1130
KRT13_P341_R	0.9939	0.0000	0.0082	0.0000	0.9824	0.0039	0.0027	0.9845	0.9914	0.0000	0.0000	0.0032	0.0000	0.7066	0.9850	0.9917
KRT5_P308_F	0.1864	0.1992	0.3643	0.8933	0.5119	0.2562	0.9475	0.6812	0.9586	0.2184	0.2337	0.9233	0.2396	0.7616	0.7854	0.0845
L1CAM_P148_R	0.0000	0.0072	0.8214	0.0101	0.0000	0.0054	0.0000	0.0074	0.0000	0.0000	0.0114	0.0135	0.0000	0.0000	0.1968	0.0043
L1CAM_P19_F	0.0000	0.0000	0.1808	0.7497	0.0878	0.0000	0.0000	0.0075	0.0000	0.0000	0.0000	0.7082	0.0000	0.0000	0.1064	0.0094
LAMC1_E466_R	0.0108	0.0000	0.0093	0.0452	0.0043	0.0674	0.0000	0.0106	0.0000	0.1865	0.1465	0.0126	0.0074	0.3359	0.0025	0.0000
LIF_E208_F	0.0091	0.0000	0.0126	0.0117	0.0127	0.2299	0.0127	0.0168	0.0000	0.0000	0.0000	0.0134	0.1238	0.0000	0.0016	0.1513
LIF_P383_R	0.0122	0.4364	0.9837	0.0000	0.4906	0.0000	0.0000	0.0000	0.0000	0.0000	0.0000	0.0000	0.0000	0.0038	0.0077	0.0000
LIG4_P194_F	0.0000	0.1857	0.0128	0.0000	0.0000	0.0000	0.0000	0.0101	0.0000	0.0000	0.0000	0.0000	0.2382	0.0000	0.0062	0.0055
LMO1_E265_R	0.0000	0.0000	0.0026	0.0000	0.0000	0.0000	0.0068	0.0035	0.0000	0.0000	0.0000	0.0000	0.0000	0.0000	0.0000	0.0029
LMO1_P169_F	0.0000	0.0000	0.0107	0.0000	0.0041	0.0000	0.8524	0.0076	0.0000	0.0000	0.0000	0.0049	0.0000	0.0000	0.0063	0.0010
LOX_P313_R	0.0727	0.0101	0.2367	0.1606	0.0247	0.1644	0.0129	0.0134	0.0000	0.0659	0.0999	0.0141	0.0091	0.0000	0.0208	0.0175
LOX_P71_F	0.0134	0.0000	0.0093	0.0000	0.0052	0.0000	0.0042	0.0056	0.0000	0.0000	0.0000	0.0037	0.0056	0.0000	0.0000	0.0049
LRP2_E20_F	0.5679	0.5034	0.5208	0.0470	0.0632	0.2463	0.6496	0.0880	0.6370	0.4703	0.5064	0.0525	0.0289	0.0257	0.0476	0.0358
LRRC32_P865_R	0.0855	0.0078	0.0164	0.0000	0.0116	0.0000	0.0033	0.0000	0.0000	0.0000	0.0000	0.0098	0.0000	0.0000	0.0012	0.0044
LTB4R_E64_R	0.4791	0.2776	0.0614	0.0268	0.0967	0.0216	0.3443	0.0219	0.0000	0.0402	0.7147	0.0508	0.5628	0.0000	0.0302	0.5314
LYN_E353_F	0.0000	0.0000	0.0074	0.0000	0.9785	0.0065	0.0000	0.0000	0.0000	0.0000	0.0000	0.0000	0.0000	0.0000	0.0000	0.0000
LYN_P241_F	0.3063	0.3643	0.1378	0.0851	0.2508	0.0422	0.0707	0.0619	0.0000	0.0684	0.0603	0.6218	0.3718	0.0344	0.0490	0.0154
MAF_P826_R	0.0080	0.0000	0.0000	0.0000	0.0000	0.0000	0.0000	0.0000	0.0000	0.0000	0.0000	0.0033	0.0000	0.0000	0.0005	0.0000
MAGEC3_P903_F	0.0000	0.0000	0.9797	0.0000	0.0046	0.0071	0.0000	0.0000	0.0000	0.0000	0.0000	0.0000	0.0000	0.0000	0.3851	0.0000
MAGEL2_E166_R	0.0310	0.0000	0.0000	0.0000	0.0000	0.0000	0.0000	0.0000	0.0000	0.0000	0.0000	0.0066	0.9909	0.0000	0.0000	0.0000
MALT1_P406_R	0.0108	0.0000	0.0000	0.0000	0.0000	0.0036	0.0037	0.0007	0.0000	0.0000	0.0000	0.0063	0.0000	0.0000	0.0000	0.0074
MAP2K6_E297_F	0.0000	0.0014	0.0044	0.0017	0.0000	0.0018	0.0000	0.0031	0.0000	0.0000	0.0000	0.0014	0.0000	0.0000	0.0000	0.0000
MAP2K6_P297_R	0.0172	0.0167	0.0246	0.0117	0.1917	0.0119	0.0172	0.3145	0.0000	0.0134	0.1012	0.1314	0.4578	0.0090	0.0145	0.0413
MAP3K1_P7_F	0.0381	0.0238	0.0262	0.0288	0.0367	0.0138	0.6577	0.0228	0.0143	0.3486	0.8287	0.1443	0.4598	0.0267	0.0707	0.0159
MAP3K9_E17_R	0.0161	0.6025	0.5165	0.1775	0.0153	0.0000	0.0000	0.3477	0.0000	0.3997	0.0000	0.0120	0.4759	0.0000	0.3717	0.4625

MAPK12_E165_R	0.0160	0.0057	0.0108	0.0079	0.0023	0.0057	0.0072	0.0121	0.1681	0.0000	0.0000	0.0067	0.0040	0.0000	0.0057	0.0152
MAPK12_P416_F	0.0000	0.0000	0.0000	0.0000	0.0000	0.0000	0.0000	0.0053	0.0000	0.0000	0.0000	0.0011	0.0000	0.0000	0.0000	0.0000
MAPK14_P327_R	0.0125	0.2584	0.0572	0.0000	0.6203	0.0288	0.0015	0.0042	0.4610	0.0732	0.0000	0.0067	0.0042	0.0000	0.0000	0.7628
MAPK4_E273_R	0.0084	0.0036	0.0057	0.9880	0.9556	0.8803	0.9920	0.3013	0.9587	0.0000	0.9926	0.0038	0.9937	0.6991	0.9706	0.7098
MAPK9_P1175_F	0.9961	0.0000	0.0000	0.0000	0.0000	0.0074	0.0000	0.9847	0.9832	0.0000	0.0000	0.0000	0.0000	0.9712	0.5376	0.9928
MAS1_P469_R	0.7788	0.5944	0.6637	0.6627	0.0868	0.2755	0.2809	0.9217	0.9763	0.4751	0.1660	0.8029	0.2365	0.9352	0.9830	0.2813
MATK_P64_F	0.0160	0.0764	0.0140	0.0099	0.0113	0.0074	0.0501	0.0083	0.0000	0.0076	0.0478	0.0089	0.0930	0.0000	0.0064	0.0052
MC2R_P1025_F	0.0000	0.0000	0.0000	0.0000	0.9762	0.0000	0.0000	0.9700	0.0000	0.0000	0.0138	0.0000	0.0000	0.6613	0.0000	0.0000
MCAM_P169_R	0.0069	0.0052	0.0143	0.0066	0.8072	0.0076	0.0000	0.0132	0.0000	0.0000	0.0000	0.0143	0.0000	0.0000	0.0061	0.0000
MCAM_P265_R	0.0155	0.0091	0.0273	0.0905	0.0116	0.0081	0.0489	0.0206	0.0000	0.0242	0.1148	0.0092	0.0816	0.0010	0.7221	0.0193
MCC_P196_R	0.0000	0.0034	0.0000	0.0000	0.0000	0.0000	0.0000	0.0000	0.0000	0.0000	0.0000	0.0000	0.0010	0.0000	0.0000	0.0000
MCM2_P241_R	0.0347	0.1255	0.0179	0.0084	0.0092	0.0074	0.0073	0.0100	0.0000	0.0131	0.0122	0.0133	0.0120	0.2002	0.0070	0.0087
MDR1_seq_42_S300_R	0.0198	0.0465	0.0132	0.0081	0.0115	0.0064	0.0189	0.0120	0.0000	0.0264	0.0112	0.0268	0.0085	0.0000	0.0024	0.0063
MDS1_E45_F	0.2619	0.0094	0.0204	0.0051	0.0010	0.0091	0.0156	0.0092	0.0000	0.0000	0.0119	0.0000	0.0571	0.0000	0.0045	0.0032
MECP2_E90_R	0.0000	0.0026	0.0056	0.1878	0.0125	0.0000	0.0092	0.0120	0.2995	0.0000	0.0138	0.0067	0.0174	0.0000	0.0065	0.0061
MEG3_E91_F	0.0080	0.0025	0.0000	0.0026	0.0000	0.0000	0.0013	0.0000	0.0000	0.0000	0.0000	0.0000	0.0000	0.0000	0.0000	0.0000
MEST_E150_F	0.0000	0.0000	0.0000	0.0000	0.0000	0.0000	0.0000	0.0023	0.5387	0.0000	0.0000	0.0000	0.0000	0.0000	0.0000	0.0000
MEST_P4_F	0.0003	0.0000	0.0000	0.0000	0.0000	0.0000	0.0009	0.0035	0.0000	0.0025	0.0020	0.0024	0.0000	0.0000	0.0000	0.0049
MEST_P62_R	0.0069	0.0000	0.0000	0.0000	0.0000	0.0006	0.0020	0.0000	0.0000	0.0000	0.0000	0.0000	0.0034	0.1340	0.0000	0.0000
MET_E333_F	0.0964	0.0143	0.2793	0.0000	0.0158	0.8460	0.6780	0.8705	0.0000	0.1566	0.0000	0.0527	0.0144	0.0000	0.7439	0.0118
MFAP4_P197_F	0.0344	0.0000	0.0063	0.0000	0.0033	0.0000	0.0011	0.0087	0.0000	0.0133	0.0000	0.0052	0.0000	0.0000	0.7956	0.0000
MGMT_P272_R	0.0000	0.0000	0.0000	0.0000	0.0000	0.0000	0.0000	0.0034	0.0000	0.0000	0.0000	0.0055	0.0038	0.0000	0.0175	0.0000
MGMT_P281_F	0.0303	0.5992	0.0212	0.0219	0.0318	0.4459	0.9916	0.3410	0.0000	0.5717	0.0222	0.0178	0.0115	0.0034	0.0311	0.2936
MKRN3_P108_F	0.2946	0.9883	0.9804	0.8571	0.0000	0.9838	0.9825	0.9827	0.0000	0.9916	0.0000	0.0000	0.0000	0.9731	0.9743	0.0000
MLF1_P97_F	0.9957	0.9949	0.0106	0.0000	0.0111	0.0058	0.0000	0.0057	0.0000	0.0734	0.0067	0.0071	0.0312	0.0000	0.0005	0.0000
MLH1_P381_F	0.0172	0.0061	0.0020	0.0127	0.0063	0.0067	0.0345	0.0090	0.0000	0.5265	0.0000	0.0071	0.0000	0.0000	0.0097	0.0032
MLH3_E72_F	0.0000	0.0127	0.0046	0.0025	0.0000	0.0000	0.0055	0.0057	0.0000	0.0000	0.0000	0.0076	0.0096	0.0000	0.0000	0.0000
MLH3_P25_F	0.4395	0.0000	0.0069	0.0000	0.0041	0.0041	0.0000	0.3697	0.0000	0.1319	0.0069	0.9913	0.0027	0.0000	0.0054	0.7041
MLLT3_E93_R	0.0153	0.0000	0.0119	0.0043	0.0000	0.0000	0.0044	0.0000	0.0000	0.0105	0.0093	0.0001	0.0034	0.0000	0.0000	0.0000
MLLT4_P1400_F	0.0273	0.1542	0.0272	0.0238	0.0187	0.2131	0.1586	0.0252	0.0000	0.0141	0.1881	0.0191	0.2099	0.0005	0.0378	0.0141
MLLT6_P957_F	0.0000	0.0053	0.0087	0.0000	0.0073	0.0042	0.0043	0.0024	0.0000	0.0000	0.0000	0.0000	0.0000	0.3467	0.3936	0.0038
MME_E29_F	0.0242	0.0115	0.0163	0.0000	0.0000	0.0083	0.0000	0.0052	0.0000	0.0000	0.0000	0.0000	0.0000	0.0000	0.0096	0.0009
MME_P388_F	0.0000	0.0176	0.0119	0.0000	0.0000	0.0000	0.0075	0.0000	0.0000	0.0105	0.0000	0.0000	0.0000	0.0000	0.0000	0.0000

MMP19_E274_R	0.0000	0.0000	0.0090	0.9897	0.9839	0.0000	0.9096	0.8424	0.9865	0.0000	0.0099	0.9917	0.0000	0.9845	0.9787	0.9892
MMP2_E21_R	0.0000	0.0000	0.0000	0.0000	0.0024	0.0068	0.0023	0.0061	0.0000	0.0060	0.0000	0.0000	0.0000	0.0000	0.0000	0.0000
MMP2_P197_F	0.0173	0.0084	0.0168	0.0076	0.0095	0.0000	0.0043	0.0123	0.4057	0.0000	0.0000	0.0097	0.0000	0.0000	0.0046	0.0040
MMP2_P303_R	0.0128	0.5041	0.3876	0.0079	0.0134	0.3854	0.0053	0.0136	0.0000	0.0053	0.2642	0.0087	0.0042	0.0000	0.0142	0.0063
MMP3_P16_R	0.0000	0.0000	0.9747	0.9888	0.9857	0.9896	0.9877	0.9854	0.9876	0.0000	0.0053	0.0000	0.9940	0.9124	0.9796	0.9918
MMP7_P613_F	0.1966	0.1019	0.2389	0.9852	0.0554	0.2815	0.9461	0.8840	0.9837	0.8789	0.1982	0.1188	0.0423	0.7540	0.9365	0.9449
MMP9_P189_F	0.0197	0.0101	0.7941	0.0114	0.1210	0.0000	0.0071	0.0098	0.0000	0.0148	0.0000	0.0102	0.0000	0.0000	0.0160	0.0000
MMP9_P237_R	0.0259	0.0104	0.0112	0.4537	0.0175	0.0057	0.0157	0.0082	0.0000	0.0057	0.0000	0.0054	0.0109	0.0000	0.0105	0.0070
MOS_E60_R	0.0140	0.0083	0.0150	0.0138	0.0270	0.0063	0.0069	0.0095	0.0000	0.0565	0.0481	0.0068	0.0073	0.0000	0.3586	0.0000
MPO_P883_R	0.0103	0.0048	0.0075	0.0017	0.0000	0.0058	0.0066	0.0050	0.0000	0.0000	0.0000	0.0054	0.0062	0.0000	0.0029	0.0039
MSH3_E3_F	0.6280	0.0270	0.9190	0.9637	0.8663	0.0088	0.9778	0.8972	0.6689	0.0000	0.0247	0.8141	0.0000	0.8738	0.9866	0.9797
MSH3_P13_R	0.0269	0.6290	0.5766	0.7396	0.0323	0.6971	0.8470	0.8376	0.5075	0.0207	0.5135	0.5467	0.6066	0.6099	0.7378	0.4508
MST1R_P392_F	0.0020	0.0000	0.0000	0.0002	0.0000	0.0000	0.0000	0.0004	0.0000	0.0000	0.0000	0.0000	0.0000	0.0000	0.0000	0.0000
MT1A_E13_R	0.0204	0.0107	0.0113	0.2995	0.0147	0.1833	0.0062	0.0166	0.9846	0.2574	0.0119	0.0670	0.0098	0.0000	0.0113	0.0067
MT1A_P49_R	0.0000	0.0000	0.0010	0.0000	0.0021	0.0000	0.0000	0.0000	0.0000	0.0000	0.0066	0.0000	0.0005	0.0000	0.0000	0.0003
MTA1_P478_F	0.1537	0.1146	0.1538	0.0115	0.0207	0.0087	0.0867	0.0120	0.0000	0.1077	0.0541	0.9734	0.1534	0.5598	0.0193	0.0996
MUC1_E18_R	0.0000	0.0000	0.0480	0.0000	0.9712	0.0000	0.0686	0.8833	0.8437	0.0000	0.0000	0.6700	0.1071	0.0000	0.0000	0.0166
MUC1_P191_F	0.0000	0.0000	0.0000	0.0000	0.0007	0.0000	0.0063	0.0120	0.0000	0.0000	0.0000	0.0000	0.0000	0.0000	0.0038	0.0048
MXI1_P75_R	0.0179	0.0051	0.0135	0.0070	0.0108	0.0050	0.0082	0.0069	0.0000	0.0095	0.0130	0.0064	0.0051	0.0000	0.0000	0.0065
MYBL2_P211_F	0.0125	0.0090	0.0260	0.0042	0.0129	0.0000	0.0000	0.0098	0.0000	0.0000	0.0161	0.0138	0.0089	0.0000	0.0000	0.0030
MYCL1_P502_R	0.0712	0.0106	0.0000	0.0041	0.0000	0.0000	0.9906	0.0000	0.0000	0.0000	0.0101	0.0075	0.0051	0.0000	0.0000	0.0065
MYCN_E77_R	0.0173	0.0171	0.0139	0.0000	0.0297	0.0054	0.0109	0.0166	0.0000	0.0078	0.0488	0.0130	0.1783	0.0056	0.0087	0.0000
MYCN_P464_R	0.0000	0.0029	0.0085	0.0000	0.0017	0.0052	0.0054	0.0083	0.0000	0.0000	0.0000	0.0102	0.0000	0.0000	0.0006	0.0055
MYH11_P22_F	0.0000	0.8381	0.0504	0.0000	0.0084	0.0585	0.0070	0.0069	0.0000	0.1250	0.0417	0.0000	0.0035	0.0000	0.0064	0.0000
MYH11_P236_R	0.0082	0.0052	0.0000	0.0000	0.0000	0.0000	0.0000	0.0000	0.0000	0.0043	0.0000	0.0000	0.0000	0.0000	0.0000	0.0000
MYLK_P469_R	0.4054	0.5404	0.0246	0.0393	0.0140	0.2355	0.0101	0.0138	0.0000	0.0172	0.4740	0.0104	0.0100	0.1578	0.1896	0.2491
MYOD1_E156_F	0.3418	0.0399	0.0077	0.0021	0.2115	0.0000	0.0033	0.0000	0.0000	0.0584	0.0000	0.0064	0.0000	0.0000	0.0000	0.0000
MYOD1_P50_F	0.0013	0.0000	0.0000	0.0000	0.0000	0.0000	0.0000	0.0000	0.0000	0.0007	0.0000	0.0000	0.0000	0.0000	0.0000	0.0075
NBL1_E205_R	0.9178	0.0705	0.7802	0.8431	0.9872	0.0539	0.9912	0.0364	0.7838	0.0634	0.9408	0.8651	0.9043	0.9710	0.9465	0.9910
NBL1_P24_F	0.0000	0.0709	0.8894	0.0000	0.0000	0.9844	0.1867	0.9298	0.9755	0.0000	0.0000	0.1458	0.0694	0.6915	0.9307	0.1824
NCL_P840_R	0.0000	0.0042	0.0065	0.0000	0.0000	0.0000	0.0021	0.0143	0.0000	0.4192	0.0081	0.0000	0.0000	0.0000	0.0083	0.0029
NDN_P1110_F	0.0152	0.9586	0.8889	0.6562	0.6482	0.8873	0.9865	0.9844	0.9160	0.9923	0.0000	0.9865	0.0000	0.9594	0.9754	0.0000
NEFL_E23_R	0.9958	0.9613	0.9947	0.9092	0.9959	0.9853	0.8266	0.6015	0.8978	0.9967	0.9975	0.8687	0.7036	0.5403	0.5692	0.6669

NEFL_P209_R	0.0000	0.0000	0.0000	0.0000	0.0000	0.0064	0.0000	0.0000	0.0000	0.0042	0.0000	0.0000	0.0000	0.0000	0.0000	0.0038
NEO1_P1067_F	0.0000	0.0017	0.0042	0.0000	0.0000	0.0000	0.0000	0.0000	0.0000	0.0000	0.0000	0.0000	0.0000	0.0000	0.0000	0.0000
NES_P239_R	0.2763	0.0100	0.0189	0.0000	0.0000	0.0000	0.0073	0.0010	0.0000	0.0060	0.2230	0.0231	0.0014	0.0000	0.0000	0.0012
NEU1_P745_F	0.0000	0.0050	0.0000	0.0000	0.0059	0.0000	0.0000	0.0058	0.0000	0.0000	0.0000	0.0055	0.0000	0.0000	0.0004	0.0000
NFKB1_P336_R	0.2587	0.0154	0.0000	0.3108	0.0000	0.0261	0.0127	0.0000	0.0000	0.0155	0.0000	0.0152	0.0000	0.0000	0.0009	0.0000
NFKB2_P709_R	0.2848	0.2295	0.2168	0.0280	0.0280	0.4749	0.0674	0.0359	0.0000	0.2710	0.1435	0.2482	0.0157	0.0059	0.0338	0.2420
NGFB_E353_F	0.0000	0.0074	0.0092	0.9882	0.0016	0.0000	0.0030	0.0029	0.0000	0.0000	0.0000	0.0000	0.0000	0.0000	0.0000	0.0047
NGFB_P13_F	0.0890	0.0157	0.0458	0.2369	0.0391	0.3783	0.0237	0.0774	0.0788	0.0135	0.3601	0.3546	0.0332	0.9734	0.1547	0.3656
NGFR_E328_F	0.0000	0.0117	0.0059	0.0047	0.0000	0.1721	0.0000	0.0000	0.0000	0.0083	0.0093	0.0764	0.0000	0.9199	0.0000	0.0000
NGFR_P355_F	0.3975	0.0710	0.1626	0.0846	0.3595	0.2770	0.3038	0.0937	0.0322	0.3682	0.3569	0.0892	0.0736	0.8594	0.6539	0.2553
NKX3-1_P146_F	0.0134	0.0000	0.0147	0.0000	0.0207	0.0078	0.0058	0.0086	0.0000	0.0000	0.0143	0.0000	0.0000	0.0000	0.0072	0.0058
NKX3-1_P871_R	0.0000	0.0000	0.9818	0.9858	0.9881	0.0025	0.0000	0.9747	0.9899	0.0000	0.9952	0.9887	0.9927	0.9805	0.9830	0.0000
NOTCH1_E452_R	0.0000	0.2879	0.0000	0.0000	0.0000	0.0000	0.0000	0.0000	0.0000	0.0000	0.0000	0.0000	0.0000	0.0000	0.0000	0.0000
NOTCH1_P1198_F	0.0066	0.0018	0.0000	0.0000	0.0000	0.0000	0.0000	0.0000	0.9814	0.0073	0.0000	0.0002	0.0000	0.0000	0.0000	0.0050
NOTCH2_P312_R	0.0129	0.0088	0.0089	0.0040	0.0055	0.0075	0.0058	0.0064	0.0000	0.0087	0.0114	0.0053	0.0089	0.0000	0.0020	0.0035
NOTCH3_E403_F	0.0000	0.0010	0.0060	0.0000	0.0000	0.0000	0.0000	0.0000	0.0000	0.0000	0.0000	0.0000	0.0000	0.0000	0.0000	0.0000
NPR2_P618_F	0.0226	0.0000	0.0000	0.0000	0.0000	0.0000	0.0130	0.0000	0.0000	0.0000	0.0000	0.4276	0.0052	0.6994	0.5195	0.0000
NPY_P295_F	0.0445	0.0288	0.0536	0.0362	0.6477	0.0248	0.3791	0.0456	0.0000	0.0450	0.5326	0.0351	0.0272	0.0008	0.0583	0.0252
NPY_P91_F	0.1369	0.0122	0.0161	0.1241	0.0223	0.1291	0.1157	0.0295	0.0000	0.0934	0.0371	0.0132	0.1124	0.0028	0.0236	0.0112
NQO1_E74_R	0.0107	0.0090	0.0088	0.0000	0.0004	0.0000	0.0000	0.0038	0.0000	0.0553	0.0000	0.0032	0.0000	0.0000	0.0021	0.0044
NQO1_P345_R	0.0000	0.0000	0.0055	0.0000	0.0000	0.0000	0.0000	0.0028	0.0000	0.0000	0.0000	0.0000	0.0020	0.0000	0.0000	0.0000
NR2F6_E375_R	0.0133	0.0134	0.0084	0.0000	0.0254	0.1748	0.0103	0.0176	0.0000	0.0142	0.0130	0.3609	0.0063	0.0000	0.0158	0.0102
NRAS_P103_R	0.0065	0.0100	0.0000	0.0000	0.0000	0.0071	0.0000	0.0042	0.0000	0.0000	0.0124	0.0080	0.0000	0.0000	0.0000	0.0044
NRAS_P12_R	0.0000	0.0000	0.0000	0.0000	0.0000	0.0000	0.0000	0.0000	0.0000	0.0000	0.0000	0.0000	0.0000	0.0000	0.0000	0.0002
NRG1_E74_F	0.0000	0.0000	0.0030	0.0033	0.0000	0.0000	0.0044	0.0024	0.0000	0.0000	0.0000	0.0011	0.0000	0.0000	0.0000	0.0017
NRG1_P558_R	0.0121	0.0699	0.0120	0.0038	0.0119	0.0048	0.0039	0.0055	0.0000	0.0000	0.0348	0.0292	0.0215	0.0000	0.0037	0.0000
NTRK2_P10_F	0.0000	0.0970	0.0045	0.0048	0.0000	0.0000	0.0000	0.0094	0.0000	0.0029	0.0000	0.0134	0.0010	0.0000	0.0012	0.0037
NTRK2_P395_R	0.0000	0.0188	0.0000	0.0000	0.0000	0.0000	0.0000	0.0000	0.0000	0.0000	0.0000	0.0000	0.0000	0.0000	0.0000	0.0000
NTRK3_E131_F	0.1139	0.0000	0.0000	0.0000	0.0056	0.0000	0.0000	0.0083	0.0000	0.0147	0.0132	0.0000	0.0082	0.0000	0.0000	0.0016
NTRK3_P636_R	0.0000	0.0029	0.0000	0.0000	0.0000	0.0000	0.0012	0.0000	0.0000	0.0028	0.0037	0.0000	0.0000	0.0000	0.0000	0.0000
NTRK3_P752_F	0.0000	0.0000	0.0025	0.0000	0.0000	0.0000	0.0000	0.0000	0.0000	0.0000	0.0000	0.0020	0.0000	0.0000	0.0000	0.0000
NTSR1_E109_F	0.0145	0.0094	0.0142	0.0032	0.0129	0.0000	0.0065	0.0086	0.0000	0.0000	0.0000	0.0057	0.0000	0.0000	0.0125	0.0042
NTSR1_P318_F	0.0510	0.0000	0.1230	0.7795	0.1233	0.0000	0.0120	0.0223	0.0000	0.4438	0.0000	0.2647	0.1130	0.0000	0.0000	0.0000

OAT_P465_F	0.0186	0.0519	0.0240	0.5120	0.0392	0.4184	0.2752	0.0141	0.0000	0.2074	0.9945	0.0093	0.0100	0.3596	0.2110	0.3143
ODC1_P424_F	0.0000	0.0000	0.0000	0.0000	0.0000	0.0000	0.0000	0.0000	0.0000	0.0000	0.0000	0.0000	0.0000	0.0000	0.0000	0.0000
ONECUT2_E96_F	0.7682	0.0000	0.0564	0.4065	0.4877	0.7024	0.6930	0.0405	0.0000	0.7714	0.2686	0.0000	0.0000	0.0000	0.0328	0.0847
ONECUT2_P315_R	0.0000	0.0000	0.0083	0.0000	0.0000	0.0000	0.0006	0.0060	0.0000	0.0000	0.0000	0.0000	0.0000	0.0000	0.0000	0.0061
OPCML_E219_R	0.2462	0.0000	0.0123	0.0051	0.0073	0.0048	0.0000	0.0079	0.0000	0.0086	0.0000	0.0053	0.0056	0.0000	0.0000	0.0030
OPCML_P71_F	0.0134	0.0051	0.0132	0.0078	0.0000	0.8047	0.0000	0.0064	0.0000	0.0084	0.0000	0.0064	0.0084	0.2896	0.0000	0.0000
OSM_P188_F	0.0827	0.0743	0.3078	0.0102	0.0091	0.1003	0.0698	0.0122	0.0000	0.0064	0.0084	0.0466	0.0670	0.0000	0.0180	0.0054
p16_seq_47_S188_R	0.0172	0.0122	0.0126	0.0911	0.0051	0.1433	0.0000	0.0098	0.0000	0.0062	0.0832	0.0074	0.0901	0.0000	0.0000	0.0040
P2RX7_E323_R	0.0063	0.0000	0.0080	0.0035	0.0066	0.0000	0.0038	0.0051	0.0000	0.0000	0.0000	0.0048	0.2123	0.0000	0.0020	0.0029
P2RX7_P119_R	0.9816	0.1455	0.0290	0.1970	0.0266	0.1509	0.0115	0.0265	0.0502	0.1200	0.1943	0.1729	0.1586	0.0172	0.0270	0.0166
P2RX7_P597_F	0.2847	0.9947	0.9908	0.9456	0.9892	0.9864	0.0132	0.9778	0.9915	0.9959	0.3974	0.2404	0.9941	0.9666	0.9871	0.9927
PALM2-AKAP2_P183_R	0.0605	0.0431	0.0486	0.8352	0.1113	0.7849	0.4606	0.0265	0.0000	0.5736	0.0439	0.0295	0.5807	0.0209	0.0497	0.4921
PALM2-AKAP2_P420_R	0.0257	0.0000	0.0053	0.0000	0.0308	0.0000	0.0103	0.0168	0.0000	0.0000	0.0000	0.0076	0.0140	0.0000	0.2705	0.4137
PARP1_P610_R	0.0146	0.0177	0.0101	0.0070	0.6195	0.0079	0.0000	0.4394	0.0000	0.0078	0.0101	0.0000	0.0000	0.9018	0.8891	0.0057
PAX6_P1121_F	0.0286	0.5487	0.0168	0.0008	0.0000	0.0094	0.0093	0.0172	0.0000	0.0000	0.0115	0.0182	0.0119	0.0000	0.0012	0.0054
PAX6_P50_R	0.0082	0.0000	0.0000	0.0000	0.0000	0.0000	0.0006	0.0017	0.0000	0.0000	0.0100	0.0000	0.0000	0.0000	0.0000	0.0000
PCDH1_E22_F	0.0000	0.3330	0.0120	0.0056	0.0000	0.0000	0.0081	0.0000	0.0000	0.0000	0.0000	0.4622	0.0000	0.0000	0.0000	0.8893
PCGF4_P760_R	0.0147	0.0556	0.0130	0.0082	0.4700	0.1248	0.0846	0.0078	0.0000	0.0598	0.0714	0.0083	0.0781	0.0000	0.0172	0.0720
PCGF4_P92_R	0.0226	0.0251	0.0134	0.0194	0.0030	0.0087	0.0319	0.0229	0.0000	0.0046	0.0257	0.0091	0.0260	0.0000	0.0137	0.0078
PCTK1_E77_R	0.8524	0.9977	0.9209	0.9725	0.9172	0.9968	0.9968	0.8314	0.8595	0.9983	0.9986	0.9950	0.9973	0.9912	0.9795	0.9963
PDE1B_E141_F	0.0219	0.0545	0.4193	0.3295	0.0188	0.0091	0.1985	0.0199	0.0000	0.3991	0.4924	0.0179	0.0106	0.1697	0.0172	0.0229
PDE1B_P263_R	0.0135	0.0106	0.0000	0.0090	0.0060	0.0059	0.0094	0.0148	0.7875	0.0000	0.0061	0.7674	0.0000	0.2734	0.0103	0.0091
PDGFA_P841_R	0.0126	0.0059	0.0039	0.0000	0.0000	0.0000	0.0037	0.0038	0.0000	0.0043	0.0000	0.9710	0.0038	0.0000	0.0000	0.0003
PDGFRB_E195_R	0.0133	0.0000	0.0153	0.0056	0.0062	0.0058	0.0086	0.0119	0.0000	0.0000	0.0000	0.0095	0.0000	0.0000	0.3362	0.7611
PDGFRB_P343_F	0.0100	0.0000	0.0095	0.0014	0.0022	0.0030	0.0000	0.0032	0.0000	0.0000	0.0000	0.0035	0.0039	0.0000	0.0015	0.0003
PEG10_P978_R	0.0156	0.0181	0.1148	0.0000	0.0046	0.0000	0.0579	0.7212	0.0000	0.0000	0.0000	0.8575	0.8684	0.0165	0.0451	0.0398
PENK_E26_F	0.0667	0.0361	0.2559	0.0275	0.0725	0.0212	0.0184	0.0187	0.1848	0.0722	0.0311	0.4025	0.0444	0.0726	0.0326	0.0204
PENK_P447_R	0.0192	0.0000	0.0259	0.0000	0.0198	0.0073	0.0000	0.0072	0.0000	0.0000	0.0129	0.0000	0.0083	0.0000	0.0182	0.0043
PGF_E33_F	0.0235	0.2859	0.0166	0.1803	0.0107	0.3870	0.0842	0.0223	0.0000	0.2840	0.0000	0.1613	0.0081	0.4912	0.0124	0.0490
PHLDA2_P622_F	0.0126	0.0000	0.0104	0.0030	0.0000	0.9910	0.0055	0.0091	0.0000	0.0000	0.0000	0.0064	0.0052	0.0000	0.0018	0.0067
PI3_P1394_R	0.4870	0.4845	0.4010	0.9838	0.6126	0.9881	0.2446	0.8467	0.9858	0.4288	0.3591	0.0249	0.9945	0.7275	0.4471	0.3752
PITX2_E24_R	0.0191	0.1236	0.0571	0.0703	0.0306	0.0109	0.1103	0.1786	0.0000	0.0934	0.1524	0.2225	0.0130	0.0000	0.0190	0.4383
PITX2_P183_R	0.0000	0.0000	0.0021	0.0000	0.0000	0.0000	0.0054	0.0000	0.0000	0.0000	0.0000	0.0000	0.0000	0.0000	0.0000	0.0000

PKD2_P287_R	0.0214	0.1457	0.0000	0.0000	0.0000	0.0082	0.0949	0.0519	0.0000	0.0142	0.0000	0.0490	0.1190	0.0000	0.0063	0.0000
PKD2_P336_R	0.2689	0.0110	0.0239	0.8838	0.0148	0.0086	0.0056	0.0121	0.2396	0.0070	0.2251	0.0094	0.0075	0.0024	0.0101	0.0060
PLAU_P11_F	0.0185	0.0174	0.0277	0.9883	0.0307	0.0123	0.2673	0.0260	0.0000	0.2433	0.3200	0.0119	0.3614	0.0028	0.0219	0.0136
PLAU_P176_R	0.0100	0.0084	0.0000	0.0000	0.0000	0.0000	0.0000	0.0206	0.0000	0.0080	0.0000	0.0078	0.0046	0.0000	0.0000	0.0000
PLAUR_E123_F	0.0118	0.0072	0.0084	0.0000	0.0050	0.0026	0.9849	0.0047	0.0000	0.0045	0.0000	0.3591	0.0000	0.0645	0.0002	0.0015
PLAUR_P82_F	0.0121	0.0000	0.0108	0.0000	0.0000	0.0000	0.0013	0.0012	0.0000	0.0116	0.2663	0.0020	0.0107	0.0000	0.0000	0.0000
PLG_E406_F	0.0000	0.0130	0.7997	0.6422	0.9167	0.9911	0.9133	0.9925	0.9901	0.0572	0.0068	0.9909	0.0005	0.9526	0.9892	0.9927
PLSCR3_P751_R	0.1136	0.0140	0.6107	0.0127	0.0187	0.0087	0.3872	0.0142	0.3110	0.0156	0.0206	0.0127	0.0148	0.1642	0.1452	0.0109
PLXDC1_P236_F	0.0109	0.0000	0.0000	0.0116	0.0051	0.0000	0.0000	0.0113	0.0000	0.0000	0.1289	0.0157	0.0101	0.0000	0.0052	0.0067
PLXDC2_E337_F	0.0000	0.0000	0.0092	0.0017	0.0000	0.0000	0.0064	0.0065	0.0000	0.0130	0.0000	0.0000	0.0111	0.0000	0.0094	0.0079
PLXDC2_P914_R	0.3757	0.3204	0.0470	0.0309	0.0676	0.0219	0.0424	0.0263	0.0000	0.4165	0.3580	0.0442	0.3528	0.0045	0.0380	0.2542
PMP22_P975_F	0.0000	0.0049	0.8948	0.0000	0.0000	0.9917	0.9909	0.0000	0.9074	0.0038	0.0000	0.0000	0.9931	0.8940	0.9741	0.0000
PODXL_P1341_R	0.0148	0.0000	0.0137	0.0050	0.0116	0.0000	0.0055	0.0094	0.0000	0.0545	0.1793	0.0066	0.0066	0.0000	0.0088	0.0064
POMC_E254_F	0.6563	0.0099	0.3639	0.5902	0.9884	0.0113	0.0000	0.0095	0.0000	0.4846	0.0113	0.0000	0.0051	0.0019	0.0123	0.0135
POMC_P400_R	0.1881	0.0000	0.0000	0.0000	0.1244	0.0000	0.0000	0.0107	0.0000	0.0000	0.0000	0.0000	0.0000	0.6582	0.0000	0.0000
POMC_P53_F	0.0204	0.1440	0.0212	0.0091	0.0827	0.1521	0.0127	0.0105	0.0000	0.3657	0.0100	0.0131	0.0883	0.0000	0.0026	0.0071
PPARD_P846_F	0.0000	0.0000	0.0133	0.0064	0.0000	0.0000	0.0000	0.0000	0.0000	0.0000	0.0000	0.0000	0.0000	0.0000	0.9753	0.0000
PPARG_E178_R	0.0615	0.1348	0.0000	0.0064	0.0000	0.0060	0.0044	0.0133	0.0000	0.0000	0.1733	0.0000	0.0000	0.0000	0.0032	0.0045
PPP2R1B_P268_R	0.0000	0.0000	0.0209	0.0050	0.0000	0.0000	0.0042	0.0057	0.0000	0.0043	0.0000	0.0027	0.0000	0.0000	0.0000	0.0000
PRDM2_P1340_R	0.0000	0.0875	0.0000	0.9779	0.7450	0.0040	0.1246	0.8813	0.9777	0.0000	0.0000	0.0000	0.1035	0.9676	0.9666	0.9834
PRKCDBP_E206_F	0.0643	0.0045	0.0086	0.0000	0.0000	0.0073	0.0000	0.0044	0.0000	0.0000	0.0953	0.0000	0.0000	0.0000	0.0000	0.0000
PROK2_E0_F	0.0189	0.0072	0.0118	0.0061	0.0018	0.0000	0.0000	0.0068	0.0000	0.0083	0.0000	0.0040	0.0056	0.0000	0.0000	0.0028
PROK2_P390_F	0.0000	0.0000	0.0000	0.0000	0.0000	0.0017	0.0000	0.0000	0.0000	0.0052	0.1135	0.0039	0.0000	0.0000	0.0000	0.0000
PRSS8_E134_R	0.1738	0.2107	0.9863	0.3065	0.9869	0.9024	0.0000	0.9732	0.0000	0.9946	0.1563	0.0090	0.0084	0.9864	0.9613	0.9905
PSCA_E359_F	0.0000	0.0073	0.0039	0.0000	0.0000	0.0021	0.3378	0.0031	0.0000	0.0000	0.0113	0.0000	0.0000	0.0000	0.0780	0.0000
PSIP1_P163_R	0.1160	0.0000	0.1379	0.0000	0.0108	0.0090	0.0000	0.0104	0.0000	0.0000	0.0000	0.0060	0.0000	0.0000	0.0000	0.0077
PTCH_E42_F	0.0000	0.0093	0.0133	0.0000	0.0085	0.0053	0.0075	0.0135	0.0000	0.0130	0.0000	0.0142	0.0000	0.0000	0.0130	0.0095
PTCH2_P568_R	0.0244	0.0308	0.4292	0.0194	0.0084	0.0218	0.0280	0.0124	0.3895	0.0277	0.0459	0.0224	0.0111	0.3506	0.5703	0.0053
PTEN_P438_F	0.0648	0.0097	0.0126	0.0076	0.0189	0.0019	0.0000	0.0171	0.0000	0.0115	0.0000	0.0175	0.0000	0.0000	0.0053	0.0391
PTGS1_P2_F	0.0248	0.0400	0.0000	0.0111	0.0168	0.0000	0.0499	0.0149	0.0000	0.0000	0.0174	0.0106	0.0000	0.0000	0.0031	0.0072
PTGS2_P308_F	0.0094	0.0478	0.3017	0.0053	0.0137	0.0111	0.0041	0.0068	0.0000	0.1797	0.0117	0.0035	0.1844	0.0000	0.0087	0.0097
PTGS2_P524_R	0.0263	0.0000	0.0210	0.0090	0.0000	0.0000	0.0105	0.0106	0.0000	0.0240	0.0151	0.0000	0.0000	0.0000	0.0046	0.0039
PTHLH_P15_R	0.9803	0.0078	0.0758	0.0005	0.0000	0.0035	0.0013	0.0037	0.0000	0.0000	0.1262	0.0000	0.0000	0.0000	0.7653	0.0015

PTHR1_P170_R	0.0101	0.0225	0.9856	0.0000	0.9910	0.0000	0.0000	0.9119	0.0000	0.0000	0.0000	0.9913	0.0000	0.5997	0.9739	0.0000
PTK2_P735_R	0.0000	0.0108	0.0223	0.2701	0.0085	0.2519	0.0392	0.0272	0.0000	0.0000	0.0794	0.0708	0.0096	0.0000	0.0316	0.0000
PTK2B_P673_R	0.0000	0.0081	0.0070	0.0026	0.0000	0.0000	0.0045	0.0118	0.0000	0.0096	0.0000	0.0046	0.0033	0.0000	0.0013	0.0000
PTPN6_E171_R	0.0087	0.0027	0.0007	0.0000	0.0000	0.0000	0.0000	0.0000	0.0000	0.0029	0.0000	0.0000	0.0000	0.0000	0.0000	0.0000
PTPN6_P282_R	0.0003	0.0000	0.0000	0.0000	0.0000	0.0000	0.0000	0.0000	0.0000	0.0000	0.0000	0.0000	0.0041	0.0000	0.0000	0.0000
PTPNS1_E433_R	0.0059	0.0000	0.0002	0.0000	0.0000	0.0000	0.0000	0.0000	0.0000	0.0000	0.0000	0.0000	0.0008	0.0000	0.0000	0.0011
PTPNS1_P301_R	0.0201	0.4773	0.0115	0.0000	0.0071	0.0000	0.0450	0.0138	0.0000	0.0000	0.0000	0.0000	0.0060	0.0000	0.0075	0.0075
PTPRF_E178_R	0.0064	0.7702	0.0047	0.0020	0.0000	0.0022	0.5585	0.0324	0.4811	0.0000	0.0055	0.0000	0.0034	0.1977	0.2325	0.8541
PTPRG_E40_R	0.1987	0.0097	0.0298	0.3793	0.0545	0.3356	0.0049	0.0140	0.0000	0.0115	0.1365	0.0106	0.1575	0.0000	0.0097	0.2158
PTPRG_P476_F	0.0023	0.2303	0.0118	0.0046	0.0029	0.0064	0.0091	0.0058	0.0000	0.0000	0.0077	0.0079	0.0000	0.0000	0.0000	0.0076
PTPRO_E56_F	0.0118	0.0047	0.0077	0.0024	0.0000	0.0047	0.0034	0.0061	0.0000	0.0000	0.0259	0.0000	0.0239	0.0000	0.0000	0.0052
PURA_P928_R	0.0117	0.0000	0.0000	0.0019	0.0000	0.0000	0.0000	0.0065	0.0000	0.0000	0.0000	0.0000	0.0000	0.0000	0.0000	0.0000
PWCR1_P811_F	0.0000	0.0000	0.9892	0.0042	0.9829	0.0000	0.0000	0.6605	0.0000	0.0000	0.9948	0.9873	0.9739	0.8740	0.6459	0.0000
PYCARD_E87_F	0.0197	0.0099	0.0199	0.1373	0.0376	0.0130	0.0078	0.0179	0.0000	0.0149	0.0745	0.0470	0.1085	0.0000	0.0346	0.0075
PYCARD_P150_F	0.0130	0.0326	0.0148	0.6867	0.0090	0.0000	0.1414	0.0138	0.0000	0.0095	0.0167	0.0095	0.0000	0.0438	0.8074	0.0067
RAB32_E314_R	0.0000	0.0000	0.0129	0.0062	0.0033	0.0052	0.0000	0.0065	0.0000	0.0000	0.0000	0.0000	0.0000	0.1077	0.0000	0.0037
RAB32_P493_R	0.0116	0.0000	0.0081	0.0010	0.0000	0.0000	0.0000	0.0007	0.0000	0.0000	0.0081	0.0000	0.0000	0.0000	0.0000	0.0000
RAF1_P330_F	0.0000	0.0000	0.3470	0.0015	0.0000	0.0001	0.0000	0.0030	0.0000	0.0064	0.0000	0.0033	0.0000	0.0000	0.0000	0.0000
RAN_P581_R	0.0000	0.0000	0.0081	0.0026	0.9871	0.0000	0.0000	0.0078	0.0000	0.0000	0.0000	0.0032	0.0000	0.8555	0.0005	0.0015
RARA_E128_R	0.0000	0.0000	0.0094	0.0057	0.0090	0.0000	0.0000	0.0067	0.0000	0.0530	0.0000	0.0082	0.0000	0.0000	0.0979	0.0046
RARA_P176_R	0.0000	0.7927	0.0000	0.0000	0.0000	0.9617	0.0000	0.0000	0.0000	0.0000	0.0117	0.0004	0.0000	0.0000	0.0000	0.0000
RARB_E114_F	0.0079	0.0000	0.0121	0.0032	0.0021	0.0039	0.0041	0.0080	0.0000	0.0065	0.0000	0.0041	0.0013	0.0000	0.0001	0.0036
RARB_P60_F	0.0048	0.0000	0.0091	0.0042	0.0000	0.0000	0.0033	0.0000	0.0000	0.0000	0.0000	0.0023	0.0036	0.0241	0.0000	0.0000
RARRES1_E235_F	0.0424	0.0172	0.5822	0.1485	0.0107	0.0116	0.0119	0.0245	0.0000	0.0205	0.1672	0.0157	0.0138	0.0000	0.0043	0.0644
RASGRF1_E16_F	0.1784	0.1773	0.1898	0.0080	0.0000	0.0062	0.0000	0.0096	0.0000	0.1470	0.0000	0.0064	0.2343	0.1830	0.0038	0.0060
RASGRF1_P768_F	0.4222	0.0317	0.0150	0.0032	0.0424	0.2173	0.0000	0.0033	0.0000	0.3012	0.3362	0.2187	0.0084	0.0000	0.0086	0.1261
RASSF1_E116_F	0.0428	0.0059	0.0033	0.0000	0.0000	0.2982	0.0000	0.2975	0.0000	0.0000	0.0000	0.0107	0.0000	0.0000	0.0002	0.0057
RASSF1_P244_F	0.0000	0.0000	0.0113	0.0091	0.0000	0.0105	0.0911	0.0136	0.0000	0.0000	0.0000	0.0082	0.0101	0.0000	0.0052	0.1674
RBL2_P250_R	0.0145	0.0130	0.2521	0.1497	0.0053	0.0054	0.0069	0.0164	0.9791	0.0103	0.0000	0.1733	0.0000	0.3284	0.0000	0.0072
RBP1_E158_F	0.0000	0.0043	0.0059	0.0008	0.0000	0.0000	0.0000	0.0000	0.0000	0.0000	0.0000	0.0000	0.0000	0.0000	0.0000	0.0000
RBP1_P150_F	0.0000	0.0000	0.0032	0.0000	0.0000	0.0000	0.0000	0.0000	0.0000	0.0000	0.0000	0.0000	0.0000	0.0000	0.0000	0.0000
RET_P717_F	0.0137	0.0117	0.2593	0.0091	0.0042	0.0228	0.0087	0.0115	0.0000	0.0000	0.0138	0.0092	0.0118	0.0000	0.0027	0.0141
RET_seq_53_S374_F	0.0000	0.7709	0.0170	0.2637	0.0841	0.2182	0.0000	0.0064	0.0000	0.0000	0.0060	0.2514	0.1851	0.0000	0.0261	0.0947

RHOC_P536_F	0.0000	0.0003	0.0000	0.0000	0.0000	0.0006	0.0009	0.7403	0.0000	0.0032	0.0000	0.0000	0.0000	0.0000	0.0000	0.0000
RHOH_P121_F	0.0000	0.0000	0.0000	0.0000	0.0000	0.0049	0.0000	0.0029	0.0000	0.0075	0.0000	0.0000	0.0000	0.0000	0.0000	0.0000
RHOH_P953_R	0.9936	0.0000	0.9816	0.9870	0.9829	0.0000	0.0000	0.9845	0.9845	0.0000	0.0000	0.0000	0.0000	0.9658	0.6962	0.0000
RIPK1_P868_F	0.0412	0.8681	0.0784	0.4558	0.8247	0.9616	0.9907	0.9678	0.9862	0.5632	0.5531	0.0394	0.9903	0.9242	0.9701	0.3587
RIPK2_E123_F	0.0000	0.0000	0.0208	0.0000	0.0110	0.0000	0.0064	0.0099	0.0000	0.0088	0.0000	0.0040	0.0000	0.0000	0.0009	0.0051
RIPK3_P124_F	0.1383	0.0066	0.0148	0.0000	0.0061	0.0146	0.1227	0.8073	0.0000	0.0000	0.0122	0.0117	0.0068	0.0000	0.0046	0.0141
RIPK3_P24_F	0.0000	0.0000	0.0000	0.0000	0.0000	0.0000	0.0000	0.0000	0.0000	0.0000	0.0000	0.0000	0.0059	0.0000	0.0000	0.0000
RIPK4_E166_F	0.7257	0.0000	0.0000	0.0055	0.0000	0.0000	0.0000	0.8565	0.0000	0.0000	0.0000	0.0000	0.0000	0.0000	0.0000	0.0035
RIPK4_P172_F	0.0128	0.0084	0.0126	0.0050	0.0080	0.0000	0.0061	0.0198	0.0000	0.0096	0.0023	0.0111	0.0056	0.0011	0.5340	0.0231
ROR1_P6_F	0.0210	0.0000	0.0191	0.0000	0.0059	0.0000	0.0000	0.0080	0.0000	0.0000	0.0000	0.0000	0.0125	0.0000	0.0017	0.0048
ROR2_P317_R	0.0114	0.0000	0.0000	0.8268	0.0044	0.0030	0.0000	0.0000	0.0000	0.0071	0.0000	0.0000	0.0000	0.0000	0.0000	0.0000
RRAS_P100_R	0.0000	0.0000	0.0000	0.0000	0.0000	0.0022	0.0012	0.9669	0.0000	0.0054	0.0000	0.0000	0.0000	0.0000	0.0000	0.0000
RUNX3_P247_F	0.9920	0.0000	0.0000	0.8778	0.4878	0.0000	0.0000	0.5648	0.5594	0.0000	0.1815	0.0037	0.0000	0.1990	0.4613	0.7480
RYK_P493_F	0.0000	0.0000	0.0000	0.0000	0.0000	0.0000	0.0000	0.0000	0.0000	0.0039	0.0000	0.0000	0.0000	0.0000	0.0000	0.0000
S100A2_E36_R	0.0123	0.0000	0.5013	0.0052	0.0000	0.0000	0.0050	0.5385	0.9544	0.0000	0.0000	0.0000	0.5258	0.0000	0.0000	0.0634
S100A4_E315_F	0.0191	0.3954	0.0171	0.1527	0.0252	0.0133	0.0073	0.0154	0.0000	0.3896	0.5007	0.0120	0.2978	0.0000	0.5005	0.0078
SCGB3A1_E55_R	0.0000	0.0171	0.0000	0.0000	0.0000	0.0000	0.0000	0.0000	0.0000	0.0056	0.0000	0.0000	0.0000	0.0000	0.0000	0.0000
SCGB3A1_P103_R	0.2933	0.0000	0.0000	0.0000	0.0000	0.0000	0.0000	0.0000	0.0000	0.0211	0.0000	0.0000	0.0000	0.0000	0.0000	0.0000
SEMA3A_P343_F	0.0000	0.0000	0.0000	0.0015	0.0000	0.0033	0.0025	0.0001	0.0000	0.0000	0.0000	0.0057	0.0000	0.0000	0.0028	0.0000
SEMA3A_P658_R	0.0298	0.1089	0.9713	0.0989	0.0851	0.0928	0.0125	0.0313	0.0000	0.0862	0.0195	0.0636	0.0726	0.0068	0.1051	0.0525
SEMA3C_E49_R	0.0167	0.0131	0.0245	0.0176	0.4904	0.0158	0.0096	0.0286	0.0000	0.1022	0.1977	0.0169	0.0136	0.4973	0.0320	0.2038
SEMA3C_P642_F	0.0000	0.2191	0.0232	0.0714	0.0000	0.0941	0.0328	0.0134	0.0000	0.0000	0.2609	0.1661	0.2029	0.0000	0.0000	0.1895
SEMA3F_E333_R	0.0000	0.0249	0.0000	0.0058	0.0045	0.0000	0.0000	0.0048	0.0000	0.0000	0.0000	0.0000	0.0000	0.0000	0.0032	0.0000
SEMA3F_P692_R	0.1186	0.0000	0.0040	0.0000	0.9689	0.0000	0.0000	0.0043	0.0000	0.0048	0.0000	0.0063	0.0037	0.0000	0.0000	0.0036
SEPT5_P441_F	0.0147	0.0290	0.0139	0.0059	0.0168	0.0183	0.0330	0.0117	0.9862	0.0000	0.0094	0.0606	0.1517	0.0000	0.9761	0.3394
SEPT9_P58_R	0.9942	0.4402	0.0597	0.9872	0.4029	0.9912	0.2714	0.0328	0.9879	0.9791	0.0139	0.9900	0.9938	0.8045	0.9835	0.9893
SERPINA5_E69_F	0.0303	0.0195	0.9740	0.0300	0.4365	0.3201	0.2270	0.4270	0.6314	0.9822	0.4596	0.3003	0.3703	0.7413	0.8884	0.0137
SERPINB2_P939_F	0.9909	0.9880	0.0000	0.9827	0.9878	0.0000	0.9861	0.7328	0.9863	0.0000	0.9951	0.0000	0.0000	0.9877	0.9792	0.9853
SERPINE1_E189_R	0.0180	0.0133	0.0150	0.0118	0.0138	0.0076	0.0084	0.2546	0.0000	0.0104	0.0148	0.1731	0.0112	0.1938	0.0279	0.0086
SEZ6L_P249_F	0.0061	0.0132	0.0095	0.0279	0.0061	0.0000	0.0000	0.0046	0.0000	0.2521	0.0144	0.0065	0.0530	0.3275	0.0102	0.0000
SEZ6L_P299_F	0.0149	0.0000	0.0017	0.0000	0.6733	0.0000	0.0000	0.0064	0.0000	0.0078	0.0117	0.0053	0.0241	0.0000	0.0000	0.0022
SFN_E118_F	0.6807	0.7210	0.9823	0.6923	0.7900	0.7407	0.9918	0.9874	0.9314	0.6780	0.9956	0.9936	0.9940	0.9467	0.9864	0.9896
SFRP1_E398_R	0.0000	0.0000	0.0019	0.0000	0.0000	0.0000	0.0000	0.0015	0.0000	0.0000	0.0000	0.0000	0.0000	0.0000	0.0015	0.0000

SFRP1_P157_F	0.0132	0.0000	0.0134	0.0051	0.0000	0.0000	0.0037	0.0079	0.0000	0.0043	0.0103	0.0480	0.0000	0.0000	0.0024	0.0000
SFTPA1_E340_R	0.0000	0.0000	0.9889	0.0000	0.0000	0.0000	0.9908	0.9782	0.9894	0.0000	0.0000	0.0000	0.0000	0.9836	0.9804	0.9901
SH3BP2_P771_R	0.0240	0.0062	0.0000	0.0032	0.0000	0.0058	0.0000	0.0044	0.0000	0.0081	0.0000	0.0000	0.0000	0.4076	0.1482	0.0000
SHB_P473_R	0.0116	0.0073	0.0000	0.0045	0.0000	0.0000	0.0000	0.0033	0.0000	0.0000	0.0087	0.4190	0.0031	0.0000	0.0000	0.0000
SHB_P691_R	0.0279	0.1220	0.0184	0.4686	0.7120	0.4975	0.3265	0.0303	0.0000	0.0095	0.0000	0.0104	0.2413	0.2650	0.0100	0.0117
SHH_E328_F	0.0000	0.0000	0.0000	0.0000	0.0000	0.0000	0.0000	0.8579	0.0000	0.0000	0.0058	0.0000	0.0000	0.6419	0.0000	0.0000
SHH_P104_R	0.2523	0.0000	0.0000	0.0098	0.0021	0.0000	0.0068	0.0107	0.0000	0.0106	0.0000	0.0077	0.4516	0.0000	0.0046	0.0107
SIN3B_P514_R	0.9642	0.0000	0.9516	0.0000	0.9523	0.9388	0.9577	0.9564	0.0000	0.0000	0.0000	0.9726	0.9827	0.9706	0.9690	0.0000
SKI_E465_R	0.0000	0.0000	0.0095	0.0086	0.0058	0.0058	0.0000	0.0081	0.0000	0.0000	0.0104	0.0000	0.0058	0.0000	0.0006	0.0043
SLC22A2_E271_R	0.9952	0.0102	0.8732	0.9937	0.0430	0.0000	0.9916	0.9818	0.9903	0.0105	0.0000	0.0000	0.9775	0.9715	0.9865	0.9126
SLC22A3_P634_F	0.8969	0.0544	0.2985	0.0087	0.5318	0.0023	0.0000	0.0115	0.4184	0.0000	0.0000	0.0059	0.0000	0.3922	0.2844	0.0083
SLC6A8_seq_28_S227_F	0.0045	0.0000	0.0000	0.0000	0.0000	0.0000	0.0000	0.0024	0.0000	0.0000	0.0000	0.0057	0.0034	0.0000	0.0000	0.0000
SLIT2_P208_F	0.1521	0.2658	0.2568	0.0052	0.9680	0.0000	0.0042	0.0170	0.0000	0.3570	0.6058	0.0104	0.3143	0.0000	0.0143	0.2600
SMAD2_P708_R	0.0000	0.0022	0.0000	0.0000	0.0037	0.0015	0.0000	0.0103	0.0000	0.0098	0.0030	0.0000	0.0000	0.0000	0.0033	0.0000
SMAD2_P848_R	0.0000	0.0000	0.0103	0.0007	0.0000	0.0000	0.0000	0.0039	0.0000	0.0048	0.0000	0.0049	0.0036	0.0000	0.0003	0.0001
SMAD4_P474_R	0.0058	0.0010	0.0006	0.0000	0.0000	0.0012	0.0000	0.0000	0.0000	0.0000	0.0041	0.0000	0.0029	0.0000	0.0000	0.0000
SMARCA3_E20_F	0.0094	0.0000	0.0082	0.0024	0.0063	0.0000	0.0023	0.0047	0.0000	0.0000	0.0000	0.0046	0.0000	0.0000	0.0002	0.0000
SMARCA3_P109_R	0.4269	0.0773	0.0146	0.0182	0.0292	0.0134	0.4706	0.0218	0.0000	0.4175	0.0072	0.0066	0.6198	0.0000	0.0210	0.0077
SMARCA3_P17_R	0.0000	0.0073	0.0114	0.0057	0.0000	0.0000	0.0000	0.0000	0.0000	0.0000	0.0000	0.0043	0.0000	0.0000	0.0000	0.0000
SMARCA4_P362_R	0.0104	0.0273	0.0523	0.0083	0.0090	0.0298	0.0397	0.0073	0.0000	0.0052	0.0101	0.0060	0.0066	0.0000	0.0075	0.0042
SMO_E57_F	0.0000	0.0000	0.0000	0.0000	0.6584	0.0041	0.0000	0.0016	0.0000	0.0000	0.0000	0.0003	0.0054	0.0000	0.0004	0.0000
SOD3_P225_F	0.0561	0.8531	0.9531	0.9885	0.9293	0.0000	0.0131	0.9309	0.9885	0.1131	0.2987	0.0000	0.1856	0.8983	0.9725	0.9901
SOX1_P1018_R	0.2994	0.0722	0.0070	0.0000	0.0269	0.0000	0.0000	0.0092	0.0000	0.0000	0.0000	0.0418	0.0035	0.0000	0.0076	0.0006
SOX1_P294_F	0.6487	0.0163	0.0236	0.0141	0.0414	0.0179	0.1844	0.0261	0.0000	0.5884	0.6140	0.0170	0.6264	0.0000	0.0365	0.3852
SOX17_P287_R	0.0215	0.0083	0.0012	0.0000	0.0000	0.0000	0.0000	0.0000	0.0000	0.0046	0.0000	0.0000	0.0003	0.0000	0.0000	0.0000
SOX17_P303_F	0.7300	0.7431	0.8659	0.9124	0.4812	0.5858	0.8847	0.0978	0.8744	0.1199	0.7589	0.1517	0.7126	0.1288	0.0888	0.1005
SOX2_P546_F	0.0150	0.0000	0.0157	0.0096	0.0183	0.0068	0.0000	0.0104	0.0000	0.0000	0.0000	0.0050	0.0000	0.0000	0.0087	0.0056
SPARC_E50_R	0.0119	0.0305	0.0480	0.0030	0.0105	0.0044	0.9636	0.0093	0.0000	0.0199	0.0123	0.0070	0.0074	0.0081	0.3332	0.0102
SPARC_P195_F	0.0135	0.0000	0.0000	0.0048	0.0053	0.0064	0.0000	0.0083	0.0000	0.0093	0.0000	0.0055	0.0000	0.8650	0.2222	0.0065
SPDEF_E116_R	0.2866	0.0892	0.0393	0.2335	0.1937	0.2456	0.1933	0.0210	0.0000	0.0110	0.2327	0.1921	0.2100	0.0000	0.9756	0.0101
SPI1_E205_F	0.1359	0.1833	0.0216	0.3621	0.5391	0.9924	0.0193	0.8543	0.8315	0.1580	0.1483	0.6698	0.1812	0.0074	0.5119	0.2710
SPP1_E140_R	0.0000	0.0000	0.5392	0.0069	0.0000	0.0105	0.0060	0.0086	0.0000	0.0099	0.0000	0.4196	0.0063	0.0000	0.0000	0.0069
SRC_P297_F	0.2714	0.0000	0.1087	0.9917	0.9894	0.3294	0.1391	0.9831	0.9832	0.1109	0.9964	0.3015	0.2545	0.9857	0.9846	0.9911

ST6GAL1_P164_R	0.2448	0.0000	0.0155	0.1168	0.0000	0.0000	0.0000	0.0931	0.0000	0.0000	0.0000	0.0683	0.0550	0.0000	0.4914	0.0000
ST6GAL1_P528_F	0.2529	0.0000	0.0078	0.0055	0.9815	0.0055	0.0000	0.0066	0.0000	0.0000	0.0061	0.0077	0.0027	0.0000	0.0000	0.0057
STK23_E182_R	0.1274	0.0000	0.6921	0.0213	0.0000	0.0045	0.0000	0.9630	0.9857	0.0000	0.0000	0.0000	0.0084	0.0000	0.9375	0.0000
SYK_E372_F	0.0880	0.1297	0.0261	0.3855	0.3898	0.0267	0.0000	0.0481	0.0000	0.1963	0.1420	0.0349	0.0752	0.0000	0.0634	0.0068
TAL1_E122_F	0.0152	0.0135	0.0166	0.0092	0.0172	0.1646	0.1990	0.0105	0.0000	0.5606	0.0134	0.4299	0.0111	0.0000	0.0114	0.0074
TAL1_P594_F	0.0000	0.9823	0.0052	0.0000	0.0067	0.0000	0.0000	0.0040	0.0000	0.0000	0.0000	0.0056	0.0000	0.0000	0.0036	0.0021
TAL1_P817_F	0.0000	0.0000	0.0000	0.0000	0.0000	0.0000	0.0000	0.0000	0.0000	0.0000	0.0000	0.0000	0.0000	0.0000	0.0000	0.0000
TCF4_P175_R	0.0000	0.0000	0.0000	0.0000	0.0000	0.0000	0.0000	0.0020	0.0000	0.0000	0.0000	0.0000	0.0000	0.0000	0.0000	0.0000
TCF7L2_E411_F	0.0044	0.0000	0.0000	0.0000	0.0025	0.0000	0.0021	0.0000	0.0000	0.0026	0.0000	0.0000	0.0000	0.0000	0.0000	0.0010
TCF7L2_P193_R	0.0000	0.0000	0.0096	0.0059	0.0002	0.0000	0.0000	0.0078	0.0000	0.0000	0.0095	0.0032	0.0000	0.0000	0.0010	0.0054
TERT_E20_F	0.9950	0.0111	0.0165	0.0103	0.0102	0.0062	0.0060	0.0161	0.0000	0.0085	0.1964	0.0355	0.0065	0.0077	0.0158	0.1200
TERT_P360_R	0.4372	0.7224	0.0201	0.4867	0.0161	0.1734	0.5559	0.0253	0.0000	0.1983	0.0218	0.0215	0.0155	0.3002	0.0344	0.4273
TES_E172_F	0.0000	0.0145	0.0000	0.0107	0.0000	0.0070	0.0145	0.0081	0.0000	0.2262	0.0000	0.0117	0.0045	0.0000	0.0000	0.1590
TESK2_P252_R	0.0133	0.0417	0.0121	0.0868	0.0085	0.0425	0.0072	0.0072	0.0000	0.0107	0.0097	0.0076	0.0434	0.0000	0.0123	0.0234
TFAP2C_E260_F	0.0127	0.0000	0.0000	0.0000	0.0000	0.0000	0.0000	0.0000	0.0000	0.0051	0.0000	0.0079	0.0000	0.0000	0.0000	0.0130
TFAP2C_P765_F	0.0101	0.0000	0.0085	0.0000	0.0006	0.0000	0.0000	0.0096	0.0000	0.0000	0.0000	0.0000	0.0000	0.0000	0.0051	0.0035
TFF2_P178_F	0.8212	0.9830	0.7360	0.9400	0.8846	0.8655	0.9708	0.6175	0.9177	0.8275	0.8273	0.7957	0.8149	0.9789	0.9837	0.7721
TFF2_P557_R	0.0000	0.9959	0.9880	0.9922	0.9878	0.9935	0.0000	0.9857	0.0000	0.2959	0.0000	0.9942	0.0930	0.9881	0.9611	0.0000
TFPI2_E141_F	0.0113	0.0000	0.0000	0.0000	0.0000	0.0000	0.0000	0.0014	0.0000	0.0000	0.0000	0.0015	0.0000	0.0000	0.0000	0.0000
TFPI2_P152_R	0.0362	0.1494	0.0330	0.5021	0.9524	0.0907	0.1054	0.0144	0.0000	0.2945	0.3244	0.3167	0.2507	0.0000	0.0081	0.0143
TFPI2_P9_F	0.0226	0.2700	0.0266	0.5023	0.0285	0.0112	0.0198	0.0153	0.0000	0.0123	0.5441	0.0165	0.0148	0.0000	0.0419	0.0169
TFRC_P414_R	0.0223	0.0091	0.0103	0.0095	0.0151	0.0159	0.3500	0.0159	0.0000	0.2372	0.2066	0.1456	0.3706	0.0000	0.0000	0.0000
TGFA_P558_F	0.0164	0.0047	0.0172	0.0024	0.0000	0.3886	0.0000	0.0633	0.0000	0.0000	0.0000	0.0082	0.3001	0.0000	0.0013	0.0000
TGFA_P642_R	0.0117	0.0000	0.0000	0.0000	0.0043	0.0000	0.0000	0.0171	0.0000	0.0000	0.0000	0.0000	0.0000	0.0000	0.0053	0.0000
TGFB1_P833_R	0.0000	0.1350	0.9894	0.0000	0.9824	0.9829	0.0218	0.7674	0.0000	0.9955	0.9965	0.0000	0.6971	0.9834	0.9811	0.9905
TGFB2_E226_R	0.0000	0.0003	0.0000	0.0000	0.0000	0.0103	0.0000	0.0080	0.0000	0.0029	0.0207	0.0105	0.0000	0.0000	0.0000	0.0000
TGFB2_P632_F	0.0190	0.0000	0.0185	0.0000	0.0099	0.0096	0.0095	0.0115	0.0000	0.1589	0.1003	0.0126	0.0095	0.0000	0.0178	0.0077
TGFB3_E58_R	0.0000	0.9939	0.6890	0.6516	0.7216	0.9865	0.9907	0.9862	0.9507	0.0000	0.0000	0.0000	0.0000	0.8609	0.9896	0.0023
TGFBI_P173_F	0.1626	0.1697	0.0281	0.0134	0.4146	0.0776	0.1291	0.0148	0.0000	0.2975	0.0138	0.0908	0.0065	0.0000	0.9351	0.0097
TGFBI_P31_R	0.2431	0.0064	0.0129	0.0000	0.6426	0.0070	0.9935	0.0139	0.0000	0.0211	0.0000	0.0076	0.0081	0.3297	0.0052	0.1297
TGFBR3_E188_R	0.0000	0.0061	0.0039	0.0000	0.0000	0.0000	0.0000	0.0030	0.0000	0.0000	0.0000	0.0000	0.0054	0.0000	0.0000	0.0016
TGFBR3_P429_F	0.0000	0.0066	0.0000	0.0000	0.0000	0.0000	0.0000	0.0025	0.0000	0.0000	0.0000	0.0043	0.0000	0.0000	0.0000	0.0000
THBS1_E207_R	0.0163	0.0000	0.3206	0.0000	0.0032	0.0096	0.0000	0.0094	0.0000	0.0100	0.1021	0.0000	0.0087	0.0000	0.0036	0.0000

THBS1_P500_F	0.0244	0.0195	0.0264	0.0154	0.0108	0.4440	0.0148	0.2756	0.0000	0.0086	0.0190	0.0000	0.0131	0.0066	0.0165	0.0090
THBS2_E129_F	0.2787	0.0042	0.0089	0.0038	0.0020	0.0058	0.0098	0.0062	0.0000	0.0039	0.0000	0.0035	0.0089	0.0000	0.0000	0.0039
THY1_P149_R	0.2726	0.1432	0.0280	0.5636	0.0501	0.4834	0.0138	0.0252	0.0000	0.1743	0.1357	0.0227	0.2215	0.0194	0.1048	0.2717
TIAM1_P188_R	0.0122	0.0062	0.0067	0.0039	0.0005	0.0022	0.0000	0.0040	0.0000	0.0000	0.0000	0.0000	0.0009	0.0000	0.0000	0.0006
TIMP1_E254_R	0.0369	0.0083	0.2808	0.9889	0.0093	0.0072	0.0472	0.0255	0.6029	0.0077	0.0460	0.0122	0.0452	0.0000	0.0146	0.0094
TIMP1_P615_R	0.0000	0.0000	0.0000	0.3682	0.0000	0.0000	0.9887	0.0000	0.9795	0.0000	0.0000	0.0000	0.0000	0.9660	0.9054	0.6105
TIMP2_E394_R	0.0000	0.0067	0.0160	0.0000	0.0004	0.0000	0.0313	0.1398	0.0000	0.1384	0.0000	0.0048	0.0000	0.0000	0.0000	0.0000
TIMP2_P267_F	0.0102	0.0058	0.0027	0.0003	0.0031	0.0000	0.5624	0.0040	0.0000	0.0034	0.0050	0.0041	0.0078	0.0000	0.6034	0.0043
TIMP3_P1114_R	0.2318	0.6798	0.9852	0.9920	0.8941	0.9926	0.2048	0.9762	0.9890	0.1158	0.9961	0.1525	0.9952	0.9897	0.9862	0.9914
TIMP3_P690_R	0.9962	0.9947	0.9863	0.9888	0.9906	0.9894	0.9909	0.9890	0.9929	0.0000	0.0000	0.9933	0.9940	0.9905	0.9915	0.0000
TIMP3_seq_7_S38_F	0.0208	0.4535	0.0215	0.0081	0.2497	0.3953	0.0000	0.3204	0.0000	0.0614	0.0638	0.5073	0.1469	0.0000	0.0088	0.0000
TJP1_P326_R	0.0233	0.0762	0.0246	0.0709	0.0718	0.0135	0.0661	0.0096	0.0000	0.0260	0.1559	0.0183	0.0304	0.0000	0.0255	0.1218
TJP1_P390_F	0.0000	0.0099	0.0101	0.0000	0.0057	0.2029	0.0000	0.0045	0.0000	0.0000	0.0000	0.0000	0.0000	0.0000	0.0004	0.0062
TK1_E47_F	0.4119	0.2239	0.2761	0.0295	0.3276	0.0160	0.2355	0.0343	0.0000	0.0369	0.0191	0.2698	0.1358	0.0000	0.1810	0.1203
TK1_P62_R	0.0595	0.0000	0.0030	0.0143	0.0115	0.0010	0.0080	0.0118	0.0000	0.0000	0.0122	0.1240	0.0657	0.0000	0.0000	0.4467
TMEFF1_E180_R	0.0164	0.0000	0.0154	0.0000	0.0000	0.0000	0.0000	0.0000	0.0000	0.0070	0.0000	0.7845	0.0050	0.0000	0.0028	0.0060
TMEFF1_P234_F	0.0000	0.0000	0.0000	0.0039	0.0067	0.9920	0.0000	0.0000	0.0000	0.0123	0.0000	0.0000	0.0000	0.0000	0.0000	0.0031
TMEFF2_P152_R	0.3634	0.0486	0.1209	0.3543	0.1086	0.2994	0.0408	0.0525	0.2405	0.0750	0.3371	0.2895	0.2959	0.0838	0.0738	0.0280
TMEFF2_P210_R	0.0000	0.0000	0.0000	0.0000	0.0000	0.0086	0.0000	0.0021	0.0000	0.0000	0.0000	0.0000	0.0000	0.0000	0.0000	0.0000
TMEM63A_E63_F	0.0000	0.0000	0.0000	0.0023	0.0000	0.0000	0.0000	0.0000	0.0000	0.0000	0.0000	0.0079	0.0000	0.0000	0.0000	0.0023
TMPRSS4_E83_F	0.0075	0.9945	0.7244	0.9896	0.9871	0.9934	0.4530	0.0215	0.9890	0.0000	0.0000	0.9908	0.9868	0.9809	0.9843	0.9917
TMPRSS4_P552_F	0.5601	0.9941	0.9870	0.9892	0.8310	0.9918	0.7773	0.9781	0.9440	0.1106	0.0131	0.3590	0.6870	0.8653	0.8749	0.2831
TNC_P198_F	0.0000	0.9636	0.0055	0.0000	0.0051	0.0000	0.0000	0.0043	0.0000	0.0000	0.0000	0.0000	0.0049	0.0000	0.0000	0.0000
TNC_P57_F	0.4390	0.3426	0.0198	0.0099	0.0183	0.0117	0.0083	0.0074	0.0000	0.0174	0.5014	0.1363	0.2963	0.0000	0.0033	0.1892
TNF_P1084_F	0.1840	0.4182	0.9916	0.9454	0.9882	0.9150	0.1212	0.9256	0.9927	0.2663	0.2031	0.9920	0.2678	0.9376	0.9840	0.4314
TNF_P158_F	0.0000	0.0000	0.0000	0.0000	0.0000	0.0102	0.0000	0.0029	0.8797	0.0017	0.0000	0.0010	0.0000	0.0000	0.0000	0.0012
TNFRSF10A_P171_F	0.0233	0.0043	0.0166	0.0178	0.0118	0.0121	0.0076	0.0117	0.0000	0.0237	0.0620	0.0079	0.1176	0.0000	0.0279	0.0092
TNFRSF10A_P91_F	0.0144	0.0000	0.0000	0.0003	0.0000	0.0142	0.0118	0.0069	0.0000	0.0158	0.0000	0.0000	0.0000	0.0000	0.0000	0.0104
TNFRSF10B_P108_R	0.0225	0.0863	0.0741	0.0149	0.0317	0.0100	0.0853	0.0367	0.0000	0.1143	0.0877	0.0948	0.0137	0.0131	0.6959	0.0876
TNFRSF10C_E109_F	0.0112	0.3831	0.0067	0.0038	0.0560	0.0034	0.0026	0.2506	0.0000	0.0058	0.0311	0.0054	0.0044	0.0000	0.0048	0.0023
TNFRSF10D_E27_F	0.0102	0.0075	0.0108	0.0058	0.0062	0.0043	0.0057	0.0067	0.0000	0.0440	0.0094	0.0044	0.0000	0.0000	0.0006	0.0035
TNFRSF10D_P70_F	0.0304	0.0257	0.0744	0.0332	0.0379	0.0206	0.0115	0.0293	0.0000	0.0220	0.0540	0.0280	0.0109	0.0000	0.0268	0.1400
TNFRSF1B_E5_F	0.0000	0.0000	0.0000	0.0000	0.0964	0.0096	0.0078	0.0145	0.0000	0.0000	0.0000	0.0000	0.0117	0.0000	0.0201	0.0000

TNFRSF1B_P167_F	0.0000	0.0055	0.0025	0.0000	0.0000	0.0000	0.0000	0.0000	0.0000	0.0050	0.0139	0.0000	0.0027	0.0000	0.0000	0.0000
TNFSF10_E53_F	0.0000	0.0000	0.0152	0.0174	0.0098	0.0151	0.0062	0.0073	0.0000	0.0000	0.7062	0.3279	0.5044	0.0000	0.0079	0.0000
TNFSF10_P2_R	0.0000	0.0000	0.0117	0.0000	0.0000	0.0000	0.0000	0.0000	0.0000	0.0000	0.0000	0.0022	0.0000	0.0000	0.9715	0.0000
TNFSF8_E258_R	0.0033	0.0000	0.5918	0.0000	0.0000	0.0050	0.6323	0.0023	0.5780	0.0050	0.0000	0.0041	0.0051	0.0000	0.0042	0.0019
TNFSF8_P184_F	0.0648	0.0055	0.0086	0.2981	0.0044	0.0009	0.0064	0.0052	0.0000	0.0000	0.0066	0.0063	0.0000	0.0000	0.0001	0.0040
TNK1_P41_R	0.0139	0.0110	0.6093	0.4697	0.0216	0.4336	0.0000	0.0205	0.0000	0.0326	0.1496	0.0126	0.0071	0.0000	0.0115	0.0000
TP73_E155_F	0.4938	0.0110	0.0196	0.4533	0.0242	0.7936	0.5282	0.0163	0.0000	0.4005	0.6473	0.0064	0.3052	0.0000	0.0207	0.2142
TP73_P496_F	0.0129	0.0000	0.0000	0.0000	0.0031	0.0000	0.0000	0.0083	0.0000	0.0000	0.0000	0.0040	0.0000	0.7636	0.0029	0.0000
TP73_P945_F	0.0130	0.0118	0.4040	0.0042	0.0151	0.0064	0.0000	0.0120	0.0000	0.0143	0.0046	0.0079	0.0000	0.2327	0.0136	0.0106
TPEF_seq_44_S36_F	0.0057	0.1727	0.0187	0.0037	0.0028	0.0056	0.0000	0.0030	0.0000	0.0000	0.1162	0.0000	0.0000	0.0000	0.0022	0.0000
TPEF_seq_44_S88_R	0.0120	0.0310	0.0156	0.0086	0.0166	0.0556	0.0286	0.0136	0.0000	0.0096	0.0132	0.0691	0.0085	0.0000	0.3864	0.0474
TRIM29_P261_F	0.8457	0.8202	0.0695	0.9882	0.9888	0.7362	0.9946	0.9771	0.0000	0.9959	0.8950	0.9909	0.8228	0.9708	0.9868	0.7532
TRIP6_E33_F	0.2808	0.0093	0.0153	0.0093	0.4023	0.0075	0.0931	0.0080	0.0000	0.2234	0.2621	0.0065	0.2566	0.0000	0.0157	0.1383
TSG101_P257_R	0.0091	0.0014	0.0000	0.0000	0.0037	0.0001	0.0000	0.0016	0.0000	0.0010	0.0089	0.0000	0.7819	0.0000	0.7919	0.0000
TUBB3_E91_F	0.0035	0.0027	0.0081	0.2838	0.2239	0.4327	0.4200	0.2412	0.2651	0.9632	0.0058	0.0000	0.0022	0.3601	0.5859	0.0000
TUBB3_P364_F	0.0000	0.0000	0.0000	0.0000	0.0000	0.0000	0.0000	0.0000	0.0000	0.0000	0.0000	0.0000	0.0000	0.0000	0.0000	0.0062
TUBB3_P721_R	0.0156	0.0000	0.0012	0.0029	0.0000	0.0000	0.0000	0.0059	0.0000	0.0053	0.0000	0.0000	0.0008	0.0000	0.0000	0.0000
TUSC3_E29_R	0.0455	0.1010	0.0098	0.0063	0.0055	0.0030	0.0058	0.0097	0.0000	0.0000	0.0000	0.0000	0.1286	0.3839	0.0082	0.1091
TUSC3_P85_R	0.0240	0.0000	0.0090	0.0034	0.1455	0.0108	0.0079	0.0075	0.0000	0.0163	0.0000	0.0057	0.0092	0.2216	0.2936	0.0034
TWIST1_E117_R	0.2409	0.0000	0.0056	0.0000	0.0000	0.0000	0.0000	0.0000	0.0000	0.0000	0.0000	0.0000	0.0000	0.0000	0.0000	0.0005
TWIST1_P355_R	0.0000	0.0000	0.0076	0.0057	0.0000	0.1601	0.0048	0.0060	0.0000	0.2682	0.0000	0.0026	0.0000	0.0000	0.0000	0.0014
TWIST1_P44_R	0.0000	0.0000	0.0000	0.0000	0.9589	0.0000	0.0000	0.0000	0.0000	0.0000	0.0000	0.0000	0.0000	0.0000	0.0000	0.0000
TYRO3_P501_F	0.0103	0.0000	0.0000	0.0000	0.0000	0.0000	0.0015	0.0000	0.0000	0.0000	0.0000	0.0000	0.0000	0.0000	0.0000	0.0017
UBA52_P293_R	0.0000	0.0000	0.0060	0.0000	0.0000	0.0000	0.0000	0.0041	0.0000	0.0000	0.0000	0.0050	0.0000	0.0000	0.0000	0.0000
UGT1A1_E11_F	0.9921	0.0000	0.9843	0.0000	0.9776	0.9917	0.0000	0.9869	0.9851	0.0052	0.0000	0.9885	0.9919	0.9695	0.9840	0.0000
UGT1A1_P564_R	0.5821	0.6318	0.9244	0.7174	0.7102	0.0812	0.9496	0.7633	0.9249	0.1607	0.9607	0.9083	0.9529	0.9507	0.9908	0.9467
UGT1A7_P751_R	0.9926	0.7625	0.9900	0.9934	0.9871	0.9520	0.9918	0.9910	0.9866	0.0000	0.0000	0.9913	0.8174	0.9821	0.9884	0.9873
UNG_P170_F	0.0229	0.0147	0.0244	0.4427	0.1185	0.0000	0.0098	0.0060	0.0000	0.0151	0.9955	0.0000	0.0068	0.3897	0.0000	0.0729
USP29_E274_F	0.0000	0.9953	0.8901	0.0000	0.9788	0.9907	0.9924	0.9048	0.8686	0.8872	0.0000	0.9918	0.9927	0.9708	0.9862	0.9933
USP29_P282_R	0.0000	0.9953	0.9845	0.0000	0.9880	0.9923	0.0016	0.9829	0.0000	0.0000	0.0000	0.0000	0.0000	0.9708	0.9851	0.9900
VAMP8_P114_F	0.0970	0.0159	0.0191	0.0112	0.1147	0.0060	0.1461	0.0179	0.0000	0.0063	0.0845	0.0094	0.1182	0.0056	0.0205	0.0084
VAV1_E9_F	0.0117	0.0073	0.0260	0.0000	0.0122	0.0051	0.0056	0.0103	0.0000	0.0000	0.0000	0.0077	0.0000	0.0000	0.0039	0.0000
VAV1_P317_F	0.0000	0.9919	0.0000	0.0000	0.0030	0.0000	0.0000	0.0048	0.0000	0.0000	0.0000	0.0000	0.0000	0.0000	0.5837	0.0000

VAV2_E58_F	0.1442	0.1796	0.0453	0.2345	0.0332	0.2005	0.0131	0.0255	0.0000	0.1618	0.2119	0.1685	0.0191	0.0350	0.0402	0.0612
VAV2_P1182_F	0.0000	0.0819	0.0000	0.0000	0.0000	0.0009	0.0023	0.0010	0.0000	0.0000	0.0000	0.0012	0.0000	0.0000	0.0000	0.0000
VBP1_P12_R	0.0000	0.9859	0.0092	0.0122	0.0032	0.0000	0.2196	0.5468	0.8144	0.0000	0.0000	0.0292	0.0000	0.0000	0.0000	0.0148
VEGFB_P658_F	0.2015	0.0083	0.0132	0.0236	0.0083	0.0083	0.0256	0.0079	0.0000	0.0656	0.0000	0.0042	0.1498	0.0774	0.0064	0.0127
VIM_P811_R	0.0000	0.0022	0.0000	0.0000	0.0000	0.0000	0.0032	0.0000	0.0000	0.0000	0.0000	0.0000	0.0000	0.0000	0.0000	0.0000
WEE1_P924_R	0.0000	0.0072	0.0053	0.0000	0.9166	0.0014	0.9918	0.9348	0.5974	0.0039	0.9966	0.0000	0.0000	0.9108	0.9895	0.9402
WNT2_E109_R	0.0000	0.0000	0.0000	0.0000	0.0000	0.0000	0.0000	0.0000	0.0000	0.0000	0.0000	0.0000	0.0000	0.0000	0.0000	0.0000
WNT2_P217_F	0.0244	0.0319	0.0333	0.8473	0.0338	0.0543	0.0275	0.0381	0.0000	0.0170	0.2459	0.0331	0.0227	0.0305	0.0461	0.0223
WNT2B_P1185_R	0.0223	0.1102	0.0223	0.0103	0.0341	0.0093	0.0164	0.0238	0.0000	0.0361	0.0101	0.0315	0.0000	0.0000	0.0245	0.3611
WNT5A_E43_F	0.2819	0.0301	0.0708	0.0109	0.0164	0.3625	0.0537	0.0152	0.0000	0.0169	0.2485	0.1297	0.0170	0.0018	0.0185	0.0127
WNT5A_P655_F	0.0947	0.0000	0.1266	0.0068	0.0000	0.0000	0.0000	0.0076	0.0000	0.0000	0.0000	0.0040	0.0000	0.0000	0.0004	0.0000
WNT8B_E487_F	0.6735	0.4500	0.9833	0.9869	0.0709	0.5256	0.9890	0.9798	0.9872	0.2790	0.7894	0.0396	0.0134	0.0000	0.9766	0.4519
WNT8B_P216_R	0.9927	0.9925	0.9774	0.9899	0.0000	0.9913	0.0000	0.9864	0.9861	0.0000	0.0000	0.9902	0.9910	0.9872	0.9832	0.9731
WRN_E57_F	0.2302	0.3034	0.0240	0.0085	0.2294	0.4447	0.2726	0.0439	0.0000	0.2343	0.2415	0.2859	0.2907	0.0078	0.0338	0.0106
WRN_P969_F	0.0167	0.0060	0.7322	0.0000	0.0000	0.0000	0.9877	0.9816	0.0000	0.0061	0.0000	0.0000	0.0000	0.9221	0.9298	0.9929
WT1_E32_F	0.0000	0.0000	0.0201	0.0000	0.1842	0.3945	0.0000	0.0189	0.0000	0.0000	0.0000	0.1249	0.0000	0.0000	0.0000	0.0065
WT1_P853_F	0.2621	0.0056	0.0029	0.0065	0.0000	0.0060	0.0000	0.0000	0.0000	0.0075	0.0000	0.0000	0.0064	0.0000	0.0000	0.0000
XRCC1_P681_R	0.9945	0.2361	0.9848	0.9896	0.9303	0.9907	0.8104	0.9544	0.9876	0.9962	0.8728	0.0000	0.9929	0.9298	0.9808	0.9900
XRCC2_P1077_F	0.0000	0.0000	0.0000	0.9887	0.9872	0.0000	0.9908	0.9878	0.9921	0.0000	0.9946	0.9903	0.9956	0.9906	0.9801	0.9918
YES1_P600_F	0.0114	0.0060	0.0217	0.0100	0.1704	0.0070	0.0056	0.0155	0.0000	0.1513	0.0000	0.0104	0.0074	0.0063	0.0134	0.0096
ZIM3_E203_F	0.9946	0.0000	0.9891	0.9888	0.9867	0.0000	0.0000	0.9152	0.0000	0.0000	0.0000	0.0000	0.0000	0.9822	0.9840	0.0000
ZIM3_P451_R	0.0205	0.9931	0.9894	0.9913	0.9538	0.9921	0.9637	0.6068	0.7328	0.0000	0.9960	0.9896	0.1465	0.9794	0.9875	0.9889
ZIM3_P718_R	0.0000	0.0000	0.9790	0.9920	0.9913	0.9908	0.9900	0.9902	0.9850	0.0000	0.7959	0.9909	0.0000	0.0000	0.9778	0.9929
ZMYND10_E77_R	0.0084	0.0000	0.0013	0.0000	0.0000	0.0000	0.0000	0.0010	0.0000	0.0000	0.0000	0.0000	0.0004	0.0000	0.0000	0.0000
ZNF215_P71_R	0.9628	0.0076	0.0125	0.0068	0.0085	0.0050	0.0016	0.0100	0.0000	0.9841	0.0109	0.9914	0.0066	0.2098	0.0067	0.0063
ZNF264_E48_R	0.0000	0.0000	0.0000	0.0000	0.0000	0.0000	0.0000	0.0000	0.0000	0.0000	0.0000	0.0000	0.0056	0.0000	0.0000	0.0000
ZNFN1A1_E102_F	0.0000	0.6998	0.4648	0.0261	0.0000	0.5234	0.6601	0.8674	0.9250	0.9921	0.0000	0.0050	0.1738	0.8564	0.9774	0.5540
ZNFN1A1_P179_F	0.0164	0.9939	0.9886	0.0000	0.9925	0.9920	0.9898	0.9893	0.0000	0.0000	0.9885	0.9891	0.0000	0.9894	0.9812	0.9901

TargetID	BWS17	BWS18	BWS19	BWS20	BWS21	BWS22 (ART)	BWS23	BWS24	BWS25	BWS26	BWS27	BWS28	BWS29	BWS30	BWS31
AATK_E63_R	0.0101	0.6964	0.9839	0.1008	0.0065	0.9939	0.9948	0.9903	0.9629	0.8605	0.9885	0.0163	0.9850	0.9923	0.0000
ABCA1_E120_R	0.9945	0.3238	0.9838	0.0000	0.9888	0.9927	0.0000	0.9349	0.9868	0.9724	0.9867	0.9914	0.9817	0.0000	0.0000
ABCA1_P45_F	0.0000	0.2495	0.9804	0.0000	0.0000	0.0000	0.9893	0.0000	0.9863	0.9833	0.9746	0.9801	0.9790	0.8898	0.9647
ABCB4_E429_F	0.0000	0.0000	0.0027	0.0052	0.0061	0.0028	0.0026	0.0066	0.0000	0.0000	0.0063	0.0026	0.0000	0.0076	0.1257
ABCC2_P88_F	0.9870	0.0000	0.1586	0.1568	0.0155	0.2469	0.3028	0.1015	0.1071	0.0000	0.6270	0.0165	0.1655	0.0000	0.0129
ABCC5_P444_F	0.1765	0.0000	0.1439	0.0072	0.0068	0.0096	0.0706	0.0046	0.0000	0.0000	0.0115	0.0793	0.1675	0.0154	0.0575
ABCG2_P178_R	0.3504	0.1567	0.0881	0.5453	0.0336	0.0461	0.0522	0.3358	0.8629	0.8621	0.0437	0.0200	0.0241	0.1164	0.0363
ABCG2_P310_R	0.0000	0.4280	0.9805	0.9904	0.0000	0.0000	0.0000	0.0000	0.9715	0.9895	0.9881	0.7614	0.9825	0.0000	0.9889
ABL1_P53_F	0.9795	0.1762	0.9851	0.9903	0.2485	0.1968	0.9887	0.9744	0.9887	0.7561	0.9875	0.9503	0.9819	0.7783	0.2948
ABL2_P459_R	0.0000	0.0000	0.0000	0.0000	0.0075	0.0040	0.0000	0.0000	0.0000	0.0000	0.0000	0.0000	0.0000	0.0000	0.0000
ABO_E110_F	0.1854	0.4281	0.0787	0.0927	0.0056	0.0068	0.0081	0.0073	0.3208	0.0000	0.0099	0.0095	0.1290	0.0588	0.0053
ABO_P312_F	0.0214	0.0000	0.1284	0.0125	0.1580	0.0084	0.0605	0.0000	0.0000	0.0000	0.3753	0.0118	0.1454	0.0107	0.0053
ACTG2_P455_R	0.0065	0.0000	0.0000	0.0000	0.0000	0.0002	0.0056	0.0000	0.0000	0.0000	0.0040	0.0000	0.0000	0.0000	0.0000
ACVR1_P983_F	0.0000	0.0000	0.0070	0.0000	0.0016	0.0000	0.0000	0.0000	0.0000	0.0000	0.0041	0.0017	0.0000	0.0056	0.0000
ACVR1B_E497_R	0.0000	0.0000	0.0058	0.0678	0.0000	0.0130	0.1474	0.1020	0.0000	0.0000	0.0054	0.0005	0.0000	0.0000	0.0119
ACVR1B_P572_R	0.2493	0.0876	0.0594	0.3387	0.0133	0.3934	0.4164	0.0085	0.0000	0.0000	0.0164	0.3242	0.1511	0.7859	0.3864
ACVR1C_P115_R	0.0000	0.0000	0.0000	0.0000	0.0000	0.0015	0.0082	0.0006	0.0000	0.0000	0.2579	0.0000	0.0815	0.0000	0.0063
ACVR1C_P363_F	0.2548	0.6438	0.9845	0.0000	0.0000	0.8625	0.0000	0.9418	0.9824	0.9874	0.9528	0.0569	0.9834	0.9933	0.0012
ACVR2B_E27_R	0.8057	0.0000	0.9802	0.8234	0.0000	0.1489	0.7057	0.6354	0.9840	0.9176	0.9673	0.9885	0.9711	0.0000	0.9896
ACVR2B_P676_F	0.0000	0.0990	0.0351	0.1209	0.0062	0.0000	0.0000	0.0000	0.0000	0.0000	0.0000	0.0000	0.0000	0.0000	0.0884
ADAMTS12_P250_R	0.0000	0.0000	0.0013	0.0000	0.0000	0.0025	0.0000	0.0000	0.0000	0.0000	0.0431	0.0000	0.0000	0.0000	0.0021
ADCYAP1_E163_R	0.2169	0.0000	0.0078	0.0000	0.0522	0.0000	0.0000	0.0100	0.0000	0.0000	0.0075	0.0052	0.0000	0.0000	0.0064
ADCYAP1_P398_F	0.9935	0.9424	0.9743	0.9722	0.9621	0.9934	0.2934	0.9391	0.9852	0.8786	0.8375	0.1350	0.9754	0.9829	0.9217
ADCYAP1_P455_R	0.0134	0.0156	0.0196	0.2752	0.2009	0.6679	0.1625	0.1716	0.0223	0.0047	0.0244	0.0529	0.0160	0.0152	0.0855
AFF3_P122_F	0.0000	0.6458	0.0722	0.0183	0.0000	0.0037	0.0000	0.0062	0.1800	0.0000	0.0073	0.0048	0.0895	0.0732	0.0000
AFF3_P808_F	0.0000	0.0000	0.0031	0.1489	0.3443	0.0008	0.0000	0.0000	0.0000	0.0000	0.3076	0.0000	0.1090	0.0000	0.0000
AFP_P824_F	0.1641	0.0000	0.0128	0.3700	0.3790	0.0100	0.0098	0.0836	0.0000	0.3928	0.3115	0.0089	0.1630	0.3639	0.3199
AGTR1_P154_F	0.0000	0.0000	0.0000	0.0000	0.0000	0.0000	0.0000	0.0000	0.0000	0.0000	0.0000	0.0000	0.0000	0.0077	0.0000
AGTR1_P41_F	0.4471	0.8498	0.0137	0.6487	0.4639	0.3509	0.4604	0.4579	0.0000	0.0000	0.0247	0.0161	0.0129	0.1867	0.0133
AHR_E103_F	0.0000	0.0000	0.9710	0.0000	0.0000	0.0000	0.9916	0.0000	0.9758	0.9638	0.9159	0.0000	0.9771	0.0000	0.0000
AHR_P166_R	0.7627	0.5725	0.8372	0.5873	0.6192	0.7196	0.7159	0.6597	0.7648	0.8392	0.6782	0.8828	0.7429	0.0104	0.0041

AIM2_E208_F	0.0000	0.0000	0.0109	0.0000	0.0110	0.0051	0.0000	0.2319	0.0000	0.0000	0.0156	0.0206	0.0494	0.0166	0.0092
AKT1_P310_R	0.0000	0.0000	0.3053	0.0083	0.0078	0.0029	0.0000	0.0000	0.9675	0.4274	0.0167	0.0094	0.3461	0.0000	0.0037
ALK_P28_F	0.0101	0.0000	0.3078	0.0132	0.0082	0.0099	0.0104	0.0027	0.0000	0.3810	0.1410	0.0000	0.3118	0.0552	0.7872
ALOX12_E85_R	0.0000	0.0000	0.0000	0.0059	0.0000	0.0000	0.0000	0.0000	0.0000	0.0000	0.4217	0.0005	0.0000	0.0064	0.0022
ALOX12_P223_R	0.9120	0.0000	0.9774	0.0000	0.0000	0.0000	0.0088	0.0000	0.9804	0.0000	0.0000	0.3904	0.9737	0.0000	0.9875
APBA1_E99_R	0.0000	0.0000	0.0000	0.0000	0.0000	0.0000	0.0000	0.0000	0.0000	0.0000	0.0000	0.0000	0.8056	0.0000	0.0000
APBA1_P644_F	0.0000	0.0000	0.0000	0.0000	0.0000	0.0000	0.0000	0.0000	0.0000	0.0000	0.0000	0.0000	0.9373	0.0000	0.0000
APBA2_P227_F	0.0000	0.0000	0.0071	0.0000	0.0000	0.0000	0.0805	0.0000	0.0000	0.0000	0.0000	0.0050	0.0606	0.0000	0.0037
APC_P280_R	0.9942	0.0000	0.9798	0.0000	0.0000	0.0000	0.0000	0.0000	0.0000	0.0000	0.9738	0.0000	0.9780	0.0000	0.3678
APOA1_P75_F	0.0099	0.4090	0.4401	0.1397	0.0172	0.0000	0.0000	0.0513	0.0000	0.0000	0.0058	0.0000	0.0000	0.0000	0.0000
APOC1_P406_R	0.0527	0.5728	0.0395	0.0090	0.0000	0.0065	0.0464	0.0034	0.4566	0.0000	0.1520	0.4935	0.1158	0.0049	0.7388
APP_E8_F	0.0000	0.3604	0.0038	0.0000	0.0037	0.0019	0.0000	0.0000	0.0000	0.0000	0.0000	0.0000	0.0000	0.0456	0.0027
APP_P179_R	0.0224	0.0000	0.0180	0.5288	0.0822	0.4714	0.0249	0.6333	0.0000	0.0000	0.0427	0.0230	0.0156	0.5991	0.0082
AR_P189_R	0.0000	0.0000	0.1847	0.1957	0.1180	0.0476	0.1661	0.0085	0.0000	0.0000	0.1911	0.0090	0.3677	0.0000	0.0074
AREG_E25_F	0.0000	0.0000	0.0000	0.0087	0.0000	0.0000	0.0000	0.0000	0.0000	0.0000	0.0000	0.0010	0.0000	0.0026	0.0046
ARHGDIB_P148_R	0.2599	0.0088	0.0343	0.0205	0.0153	0.0195	0.2025	0.0120	0.0097	0.0186	0.0286	0.0454	0.0247	0.0168	0.0183
ARNT_P238_R	0.0000	0.0000	0.0084	0.3681	0.0000	0.0080	0.0067	0.3125	0.0000	0.0000	0.0074	0.3073	0.0000	0.0000	0.0035
ASB4_P391_F	0.0000	0.0000	0.0062	0.0108	0.0055	0.0000	0.0000	0.0000	0.0000	0.0000	0.0048	0.0079	0.0000	0.0000	0.0061
ASB4_P52_R	0.0000	0.0000	0.0041	0.0000	0.0000	0.0000	0.0000	0.0000	0.0000	0.0000	0.0096	0.0000	0.0513	0.0000	0.0000
ASCL1_E24_F	0.0000	0.0000	0.0000	0.0087	0.0093	0.0000	0.0076	0.0000	0.0000	0.0000	0.0000	0.0000	0.0000	0.0000	0.0000
ASCL1_P747_F	0.0000	0.0000	0.1647	0.0000	0.0043	0.1833	0.0063	0.0798	0.0000	0.0000	0.1264	0.1082	0.0000	0.9879	0.0999
ASCL2_E76_R	0.0013	0.0000	0.0000	0.0068	0.0040	0.0007	0.0020	0.0028	0.0000	0.0000	0.0015	0.0000	0.0000	0.0049	0.0000
ASCL2_P360_F	0.0000	0.0000	0.2382	0.0000	0.0000	0.9913	0.0000	0.0037	0.0000	0.0000	0.0000	0.0000	0.0699	0.0000	0.0000
ASCL2_P609_R	0.0210	0.0000	0.0000	0.0000	0.0000	0.0000	0.0000	0.0000	0.0000	0.0000	0.0000	0.0000	0.0000	0.0000	0.0000
ATP10A_P147_F	0.0119	0.0000	0.6184	0.0098	0.0086	0.0027	0.0000	0.0000	0.9635	0.0000	0.0193	0.0110	0.0391	0.0329	0.0108
ATP10A_P524_R	0.1486	0.3948	0.0093	0.1439	0.0094	0.1947	0.0365	0.0134	0.0000	0.0000	0.0165	0.0094	0.0000	0.0108	0.0127
AXIN1_P995_R	0.6950	0.7859	0.0183	0.6701	0.5319	0.3914	0.0172	0.0091	0.0000	0.0000	0.0133	0.3956	0.0086	0.0424	0.0136
AXL_E61_F	0.0000	0.0000	0.0000	0.0000	0.0000	0.0027	0.0034	0.0042	0.0000	0.0000	0.0056	0.0035	0.0000	0.0097	0.0017
BAX_E281_R	0.0264	0.0000	0.0118	0.0929	0.1576	0.0348	0.3376	0.4254	0.0000	0.0000	0.0204	0.0201	0.0089	0.0251	0.4769
BCAM_E100_R	0.0823	0.0000	0.0098	0.2370	0.1552	0.0042	0.0087	0.0740	0.0000	0.0000	0.0546	0.0078	0.0973	0.0000	0.0080
BCAM_P205_F	0.0086	0.0000	0.2380	0.5555	0.5187	0.0000	0.0000	0.0079	0.0000	0.0000	0.9055	0.3355	0.0060	0.0135	0.3842
BCAP31_P1131_F	0.7257	0.9349	0.0129	0.8238	0.0201	0.0245	0.7263	0.6642	0.0000	0.0000	0.0167	0.0216	0.0111	0.0462	0.0219
BCL2L2_E172_F	0.0840	0.0000	0.0000	0.0030	0.0049	0.0000	0.0000	0.0000	0.0000	0.0000	0.0061	0.0018	0.0000	0.0000	0.0000

BCL2L2_P280_F	0.1205	0.0000	0.0064	0.0075	0.0000	0.0051	0.0000	0.0059	0.0000	0.0000	0.0086	0.0055	0.0000	0.0000	0.0032
BCL3_E71_F	0.0059	0.0000	0.0020	0.0060	0.0051	0.0000	0.0062	0.9940	0.0000	0.0000	0.0053	0.0023	0.0000	0.0073	0.0033
BCL3_P1038_R	0.3268	0.1364	0.1581	0.0114	0.0074	0.0147	0.3557	0.0052	0.0000	0.0000	0.0128	0.1313	0.0009	0.0142	0.3221
BCL6_P248_R	0.0000	0.0000	0.0058	0.0000	0.0057	0.0113	0.0000	0.0000	0.0000	0.0000	0.7928	0.0000	0.0000	0.0140	0.0001
BDNF_E19_R	0.0073	0.0000	0.0319	0.0080	0.0061	0.0037	0.0057	0.0031	0.0154	0.0000	0.0142	0.0889	0.0191	0.0108	0.0042
BDNF_P259_R	0.7240	0.8848	0.9841	0.9955	0.9943	0.0204	0.5937	0.9925	0.9892	0.9914	0.9742	0.9878	0.9825	0.9929	0.9911
BGN_E282_R	0.0095	0.0140	0.0081	0.0000	0.0000	0.0021	0.0000	0.0028	0.0000	0.0000	0.0069	0.9889	0.0000	0.0000	0.0017
BGN_P333_R	0.0131	0.7215	0.3188	0.3778	0.0473	0.0076	0.3496	0.0096	0.0023	0.4913	0.0123	0.0024	0.0419	0.0150	0.0000
BIRC4_P122_R	0.2382	0.0000	0.0995	0.1622	0.0123	0.4493	0.3635	0.0102	0.0000	0.2386	0.0165	0.3824	0.2008	0.0608	0.2712
BIRC5_E89_F	0.1175	0.0000	0.0161	0.0968	0.0787	0.0164	0.0751	0.0703	0.0000	0.0000	0.0123	0.0173	0.0066	0.0674	0.0935
BLK_P14_F	0.2761	0.0000	0.0089	0.1128	0.2706	0.0082	0.1829	0.0063	0.0000	0.0000	0.0114	0.2594	0.0006	0.0000	0.0105
BMP2_E48_R	0.3961	0.7606	0.1765	0.5722	0.4225	0.3412	0.9084	0.3037	0.4751	0.0000	0.0097	0.0087	0.0000	0.7042	0.2992
BMP2_P1201_F	0.0000	0.0000	0.0005	0.0000	0.0000	0.0000	0.0050	0.0000	0.0000	0.0000	0.0031	0.0000	0.1674	0.0000	0.0036
BMP3_E147_F	0.0096	0.0000	0.0000	0.0000	0.0000	0.0000	0.0000	0.0000	0.0000	0.0000	0.0005	0.0000	0.0000	0.0000	0.0004
BMP3_P56_R	0.0000	0.0000	0.0034	0.0000	0.0000	0.0000	0.0000	0.0000	0.0000	0.0000	0.0044	0.0038	0.0000	0.0000	0.0010
BMP4_P199_R	0.0924	0.0000	0.2157	0.0335	0.0118	0.9904	0.0989	0.1247	0.0000	0.9472	0.5509	0.9812	0.4898	0.3894	0.1115
BMP6_P398_F	0.9946	0.4310	0.9832	0.9960	0.0000	0.9951	0.0123	0.9947	0.9913	0.9840	0.9866	0.9835	0.9825	0.0046	0.9920
BMPR1A_P956_F	0.1035	0.0000	0.0122	0.0000	0.1177	0.0081	0.1406	0.0057	0.0000	0.2813	0.0130	0.0189	0.0044	0.0178	0.0098
BMPR2_E435_F	0.0000	0.0000	0.0000	0.0000	0.0000	0.0000	0.0000	0.0316	0.0000	0.0000	0.0000	0.0000	0.0000	0.0116	0.0000
BMPR2_P1271_F	0.1217	0.0000	0.0539	0.0000	0.0389	0.0069	0.0000	0.0012	0.0139	0.0000	0.0107	0.0053	0.0053	0.0000	0.0022
BSG_P211_R	0.0556	0.1968	0.0651	0.1300	0.4711	0.1105	0.1743	0.3777	0.4018	0.0516	0.0724	0.0887	0.0647	0.6879	0.1124
BTK_P105_F	0.1152	0.0000	0.0944	0.0078	0.0146	0.1629	0.1897	0.0078	0.0015	0.0000	0.0698	0.0105	0.0072	0.0092	0.0066
C20orf47_P225_R	0.0000	0.1185	0.0028	0.0119	0.0027	0.0000	0.0000	0.0000	0.0000	0.0000	0.0085	0.0075	0.0077	0.9132	0.0017
CALCA_E174_R	0.5620	0.3284	0.0352	0.7037	0.6612	0.6725	0.0898	0.5639	0.0574	0.1007	0.5349	0.0916	0.0735	0.2423	0.6058
CAPG_E228_F	0.0077	0.0000	0.0302	0.0000	0.0026	0.0000	0.0024	0.0069	0.1591	0.0000	0.2244	0.0003	0.0260	0.0060	0.0000
CASP10_E139_F	0.0119	0.7874	0.0055	0.2880	0.1136	0.0050	0.0059	0.0000	0.0000	0.0000	0.0056	0.7046	0.0000	0.0129	0.0000
CASP10_P186_F	0.2407	0.4303	0.8020	0.0000	0.3756	0.0000	0.0654	0.0000	0.9800	0.6282	0.9836	0.8383	0.9737	0.9883	0.0000
CASP2_P192_F	0.3269	0.1255	0.0248	0.5553	0.0495	0.0241	0.0338	0.0288	0.0114	0.3629	0.0194	0.0424	0.0216	0.0940	0.0520
CASP3_P420_R	0.0093	0.0000	0.0838	0.0000	0.0037	0.0038	0.0000	0.0000	0.0000	0.0000	0.0137	0.0030	0.0000	0.0023	0.0000
CASP6_P201_F	0.0000	0.3955	0.3682	0.9902	0.0000	0.0122	0.0268	0.0074	0.7391	0.0000	0.0184	0.3395	0.5615	0.0000	0.0118
CASP6_P230_R	0.0486	0.0000	0.2862	0.0091	0.0087	0.8771	0.0037	0.0057	0.0000	0.7997	0.0113	0.1710	0.0037	0.0148	0.0280
CAV1_P130_R	0.3598	0.1072	0.3237	0.4466	0.2756	0.0120	0.2494	0.0201	0.9346	0.0000	0.0074	0.9886	0.1839	0.3398	0.0087
CAV1_P169_F	0.1560	0.0112	0.0212	0.0203	0.0180	0.1420	0.2065	0.2043	0.0155	0.0337	0.0480	0.0116	0.0163	0.0434	0.0158

CAV2_E33_R	0.0095	0.0000	0.3806	0.0134	0.0261	0.9850	0.1432	0.0071	0.2666	0.4760	0.0171	0.1308	0.3044	0.0416	0.0106
CCKBR_P361_R	0.0133	0.1601	0.0098	0.0215	0.3046	0.0104	0.0184	0.0827	0.0000	0.0000	0.0164	0.0157	0.0070	0.0196	0.0089
CCKBR_P480_F	0.0000	0.0000	0.0000	0.0000	0.0035	0.0028	0.0145	0.0000	0.0000	0.0000	0.0029	0.0026	0.0000	0.1163	0.0033
CCNA1_E7_F	0.4141	0.0000	0.9739	0.9949	0.9950	0.9816	0.0000	0.0291	0.9861	0.9897	0.7807	0.0000	0.9753	0.9943	0.0000
CCNA1_P216_F	0.0000	0.0000	0.0000	0.0106	0.0000	0.0000	0.0017	0.0000	0.0000	0.0000	0.0000	0.0000	0.0000	0.0000	0.0000
CCNC_P132_R	0.0102	0.0000	0.3146	0.0229	0.0066	0.0000	0.0000	0.0000	0.0000	0.0000	0.6926	0.0031	0.1462	0.5049	0.9892
CCND1_E280_R	0.1536	0.0000	0.5821	0.0133	0.0000	0.0000	0.0000	0.0025	0.0000	0.0000	0.1967	0.0055	0.5463	0.0000	0.0013
CCND1_P343_R	0.0000	0.0000	0.0000	0.0000	0.0000	0.0000	0.0000	0.0002	0.0000	0.0000	0.0000	0.0000	0.0000	0.0000	0.0000
CCND2_P887_F	0.0000	0.0000	0.0000	0.0000	0.0000	0.0000	0.0000	0.0000	0.0000	0.0000	0.0000	0.0023	0.0000	0.0067	0.0001
CCND2_P898_R	0.4393	0.6111	0.8362	0.0512	0.5425	0.0473	0.4683	0.5058	0.9027	0.7979	0.9209	0.4461	0.9292	0.7674	0.0726
CCNE1_P683_F	0.0000	0.0000	0.0883	0.0062	0.0000	0.0000	0.0000	0.0000	0.0000	0.0000	0.0000	0.0000	0.0000	0.0000	0.0000
CD1A_P414_R	0.0668	0.0000	0.0082	0.0000	0.1599	0.0137	0.0000	0.0000	0.0000	0.0000	0.0120	0.0077	0.0037	0.0000	0.2538
CD2_P68_F	0.5067	0.4693	0.6228	0.9945	0.8047	0.9940	0.0157	0.0000	0.2404	0.7038	0.4958	0.2919	0.6376	0.0167	0.0049
CD34_P339_R	0.2042	0.0000	0.0226	0.1918	0.1705	0.2244	0.1997	0.0146	0.0000	0.0000	0.0194	0.0459	0.0115	0.3482	0.0175
CD34_P780_R	0.9937	0.3103	0.9012	0.9826	0.9523	0.0497	0.0219	0.0133	0.9025	0.7514	0.6823	0.4531	0.8770	0.9946	0.8070
CD40_E58_R	0.0000	0.0000	0.0013	0.0000	0.0000	0.0353	0.0000	0.0499	0.0000	0.0000	0.0098	0.0000	0.0000	0.0000	0.0036
CD40_P372_R	0.0182	0.0000	0.1337	0.0164	0.1728	0.0344	0.0116	0.2416	0.0168	0.0138	0.0247	0.0313	0.0155	0.1794	0.0184
CD44_E26_F	0.0000	0.0000	0.0675	0.0000	0.0000	0.0065	0.0000	0.0020	0.0000	0.0000	0.0000	0.0058	0.0000	0.0000	0.0000
CD44_P87_F	0.0172	0.0000	0.8771	0.9963	0.0000	0.0000	0.0000	0.0000	0.9718	0.0000	0.0013	0.0000	0.5597	0.0052	0.0000
CD86_P3_F	0.0000	0.0000	0.0000	0.0000	0.0000	0.0000	0.0000	0.0000	0.0000	0.0000	0.0000	0.0099	0.0000	0.0000	0.0000
CDC25B_E83_F	0.0000	0.0000	0.0000	0.0080	0.0000	0.0000	0.0000	0.0000	0.0000	0.0000	0.0016	0.0000	0.0000	0.0000	0.0000
CDC25B_P11_R	0.0000	0.0000	0.0115	0.0000	0.0000	0.0020	0.1433	0.1819	0.0000	0.0000	0.0106	0.0136	0.0029	0.0072	0.0675
CDH1_P52_R	0.0000	0.0000	0.0000	0.0065	0.0000	0.0000	0.0033	0.0000	0.0000	0.0000	0.0020	0.0001	0.0000	0.0045	0.0001
CDH11_E102_R	0.1413	0.0000	0.0023	0.0000	0.0051	0.0000	0.0000	0.0000	0.0000	0.0000	0.0081	0.0026	0.1588	0.0064	0.0000
CDH11_P203_R	0.3303	0.3873	0.0159	0.0184	0.0137	0.0906	0.5288	0.3957	0.0000	0.0000	0.2028	0.0162	0.0072	0.7984	0.5562
CDH11_P354_R	0.0106	0.0000	0.0045	0.0062	0.0000	0.0036	0.0052	0.0000	0.0000	0.0000	0.4400	0.0029	0.0000	0.0000	0.0000
CDH13_E102_F	0.3145	0.8356	0.9859	0.0000	0.0121	0.9938	0.1856	0.9925	0.9875	0.9853	0.9902	0.9878	0.9864	0.9929	0.9919
CDH17_E31_F	0.4089	0.0000	0.5029	0.2589	0.0061	0.0099	0.4980	0.0000	0.9798	0.2830	0.0176	0.0149	0.0905	0.0000	0.2446
CDH17_P532_F	0.9927	0.2994	0.9792	0.7485	0.6302	0.9939	0.6103	0.9873	0.9835	0.9884	0.9803	0.9084	0.9768	0.9918	0.5387
CDH3_E100_R	0.2051	0.0000	0.0327	0.0183	0.1473	0.0182	0.0168	0.2020	0.0175	0.0200	0.0303	0.1683	0.0217	0.1286	0.0215
CDH3_P87_R	0.0000	0.0000	0.0012	0.0085	0.0000	0.0000	0.0039	0.0000	0.0000	0.0000	0.0042	0.0003	0.0000	0.0117	0.0047
CDK10_E74_F	0.5346	0.1532	0.6455	0.5720	0.0222	0.4692	0.5221	0.4123	0.9405	0.9849	0.4814	0.5217	0.9430	0.0701	0.0303
CDK2_P330_R	0.0000	0.0000	0.6122	0.0000	0.0000	0.0000	0.0000	0.9926	0.9851	0.9213	0.0105	0.9858	0.8971	0.0000	0.0034

CDK6_E256_F	0.0089	0.0000	0.1190	0.0000	0.0071	0.0000	0.0100	0.0000	0.0000	0.2747	0.0094	0.0000	0.3720	0.0000	0.0041
CDK6_P291_R	0.0101	0.0000	0.0172	0.2251	0.2620	0.0131	0.2332	0.3122	0.0003	0.2189	0.0478	0.0372	0.0142	0.1009	0.0148
CDKN1A_E101_F	0.0070	0.0000	0.1057	0.0000	0.0000	0.0000	0.0000	0.0955	0.8144	0.0000	0.0123	0.9760	0.1563	0.0102	0.0901
CDKN1A_P242_F	0.0000	0.0000	0.0040	0.0000	0.0070	0.0036	0.0000	0.0919	0.0000	0.0000	0.0068	0.0061	0.0633	0.9606	0.0000
CDKN1B_P1161_F	0.1047	0.2337	0.0000	0.0616	0.0000	0.0000	0.0044	0.0014	0.4799	0.0000	0.0000	0.0000	0.1450	0.0000	0.0000
CDKN2A_E121_R	0.0000	0.0000	0.0982	0.0096	0.0095	0.0043	0.0046	0.0033	0.0000	0.0000	0.0105	0.0064	0.0549	0.0000	0.0063
CDKN2B_E220_F	0.0000	0.4379	0.5241	0.0000	0.0000	0.0000	0.0054	0.0000	0.0000	0.0000	0.0000	0.0031	0.1317	0.0092	0.0000
CDM_seq_21_S260_R	0.0000	0.0000	0.1345	0.0000	0.0000	0.0000	0.0095	0.0000	0.0000	0.0000	0.0851	0.0000	0.0000	0.0170	0.0050
CEACAM1_E57_R	0.0105	0.0000	0.0065	0.0000	0.0068	0.0032	0.0000	0.0028	0.0000	0.0000	0.0079	0.0000	0.0000	0.0000	0.0030
CEACAM1_P44_R	0.0090	0.0000	0.0732	0.0000	0.0000	0.0148	0.2851	0.1521	0.0000	0.0000	0.0109	0.2050	0.0856	0.0079	0.9918
CEBPA_P1163_R	0.0104	0.1857	0.0198	0.0804	0.1700	0.0140	0.0091	0.1311	0.0000	0.0000	0.0155	0.0142	0.0083	0.0116	0.0136
CEBPA_P706_F	0.0090	0.0000	0.0148	0.0099	0.0080	0.0000	0.1396	0.0942	0.2060	0.0000	0.0101	0.0064	0.2958	0.0000	0.7765
CFTR_P115_F	0.0000	0.0000	0.0058	0.1031	0.0037	0.0030	0.0000	0.0000	0.0000	0.0000	0.0076	0.0057	0.0000	0.0000	0.0000
CHD2_P451_F	0.0000	0.0000	0.5727	0.0100	0.0054	0.0000	0.0041	0.0000	0.0000	0.0000	0.0047	0.0000	0.6895	0.0037	0.0000
CHFR_P501_F	0.0000	0.0000	0.0031	0.0063	0.0050	0.0000	0.0087	0.0010	0.0000	0.0000	0.0056	0.0051	0.0000	0.0072	0.0000
CHFR_P635_R	0.0990	0.2466	0.9839	0.9847	0.0000	0.9108	0.0638	0.7563	0.9900	0.9344	0.9785	0.3623	0.9820	0.0018	0.2392
CHGA_E52_F	0.0000	0.0000	0.0017	0.0092	0.0000	0.0014	0.0066	0.0000	0.0000	0.0000	0.0051	0.0000	0.0000	0.0067	0.0000
CHI3L2_E10_F	0.0148	0.2373	0.6301	0.0000	0.0126	0.0004	0.0000	0.0000	0.2531	0.4122	0.1514	0.0060	0.6929	0.0128	0.0066
CLK1_P538_F	0.0000	0.0000	0.0008	0.0000	0.0019	0.0000	0.0000	0.0000	0.0000	0.0000	0.0032	0.0011	0.0000	0.0000	0.0000
COL18A1_P365_R	0.0198	0.7978	0.0102	0.0000	0.2237	0.0225	0.0116	0.2287	0.0000	0.0000	0.0197	0.0138	0.0488	0.0000	0.0112
COL1A1_P5_F	0.0000	0.0000	0.0000	0.0000	0.0000	0.0000	0.0080	0.0000	0.0000	0.0000	0.0042	0.0002	0.0000	0.0000	0.0000
COL1A2_E299_F	0.0186	0.0000	0.4090	0.0129	0.0054	0.0070	0.0000	0.7496	0.2314	0.5438	0.4249	0.0065	0.4443	0.0000	0.0000
COL1A2_P407_R	0.0134	0.0000	0.0000	0.0000	0.0044	0.0000	0.0038	0.0017	0.5875	0.0000	0.0000	0.0000	0.0000	0.0000	0.0000
COL1A2_P48_R	0.1108	0.0000	0.0336	0.0756	0.1386	0.1611	0.1418	0.0729	0.0000	0.0019	0.0293	0.0458	0.0244	0.0296	0.1590
COL4A3_E205_R	0.0000	0.4457	0.1546	0.0000	0.0000	0.0000	0.0053	0.0025	0.0000	0.0000	0.0033	0.0043	0.0994	0.0000	0.1386
COL4A3_P545_F	0.7080	0.4330	0.0177	0.0651	0.0573	0.7948	0.7762	0.0379	0.0074	0.8140	0.0329	0.0360	0.0211	0.4633	0.0633
COL6A1_P283_F	0.0281	0.0000	0.3487	0.0000	0.0596	0.8802	0.9513	0.0447	0.4609	0.0000	0.0242	0.0427	0.2603	0.0018	0.0021
COL6A1_P425_F	0.4578	0.0000	0.0092	0.0000	0.0000	0.0260	0.0000	0.0053	0.0000	0.0000	0.0202	0.1151	0.2771	0.1812	0.0430
COPG2_P298_F	0.1548	0.2834	0.0360	0.1518	0.6359	0.1065	0.1090	0.5577	0.0535	0.0773	0.0637	0.5156	0.0546	0.7905	0.1120
CPA4_P1265_R	0.6992	0.9098	0.0367	0.9919	0.7797	0.0302	0.6669	0.6668	0.0486	0.0022	0.0510	0.0580	0.0300	0.1002	0.0197
CREB1_P819_F	0.0000	0.2804	0.1504	0.0121	0.0097	0.0000	0.0115	0.0000	0.0000	0.0000	0.0118	0.0042	0.1193	0.0157	0.0000
CRIP1_P874_R	0.2212	0.7759	0.1217	0.9909	0.0106	0.0100	0.0143	0.0049	0.0000	0.0000	0.0125	0.3751	0.1344	0.4529	0.0129
CRK_P721_F	0.0078	0.0000	0.0265	0.0106	0.0060	0.0050	0.0052	0.0040	0.0000	0.0000	0.0053	0.0044	0.0214	0.0110	0.0035

CSF1_P217_F	0.1878	0.0000	0.5614	0.0378	0.1379	0.9934	0.9921	0.1093	0.0000	0.0000	0.0208	0.0178	0.7533	0.0550	0.1437
CSF1_P339_F	0.0000	0.0000	0.0000	0.0000	0.0000	0.0000	0.0000	0.0000	0.0000	0.0000	0.0031	0.0000	0.0000	0.0000	0.0000
CSF1R_E26_F	0.1208	0.5216	0.0006	0.0103	0.0068	0.0000	0.0074	0.0000	0.0000	0.0000	0.0079	0.0052	0.0000	0.0071	0.0000
CSF3R_P472_F	0.0267	0.0192	0.1148	0.1662	0.1745	0.1862	0.0301	0.1626	0.0126	0.0438	0.0266	0.0231	0.0243	0.0279	0.0338
CSPG2_E38_F	0.0250	0.0000	0.0035	0.0000	0.0000	0.0087	0.0000	0.0000	0.0000	0.0000	0.0077	0.0178	0.0000	0.0127	0.0047
CSPG2_P82_R	0.0000	0.0000	0.0999	0.0000	0.0000	0.0000	0.5090	0.1174	0.0000	0.0000	0.4646	0.2466	0.0000	0.0098	0.0038
CSTB_E410_F	0.0080	0.5755	0.7835	0.0000	0.9940	0.0062	0.0040	0.0000	0.9835	0.6139	0.8089	0.9176	0.7654	0.0000	0.0000
CTAG1B_P4_R	0.0000	0.0000	0.2422	0.0075	0.0000	0.0000	0.0000	0.0000	0.0000	0.4570	0.0003	0.0000	0.0558	0.0047	0.0000
CTAG1B_P77_F	0.4090	0.7890	0.4731	0.2213	0.3770	0.2795	0.0160	0.7827	0.0690	0.1766	0.8024	0.8255	0.2877	0.0926	0.0447
CTAG2_P1426_F	0.4953	0.4734	0.0144	0.7272	0.0251	0.0305	0.0247	0.6826	0.0000	0.0000	0.0094	0.0168	0.0059	0.8521	0.0138
CTGF_E156_F	0.0089	0.0652	0.0005	0.1432	0.0000	0.0000	0.0079	0.0000	0.0000	0.0000	0.0028	0.0032	0.1032	0.0138	0.0012
CTLA4_P1128_F	0.0316	0.9878	0.3771	0.9958	0.9316	0.4255	0.2379	0.2891	0.7289	0.7186	0.2441	0.9920	0.6709	0.9960	0.9777
CTNNA1_P185_R	0.0000	0.0000	0.0015	0.0000	0.0000	0.0036	0.0078	0.0000	0.0000	0.0000	0.0052	0.0014	0.0000	0.0000	0.0032
CTNNA1_P382_R	0.0069	0.0000	0.0033	0.1315	0.3285	0.0847	0.9931	0.0054	0.0000	0.0000	0.0086	0.0052	0.0000	0.0111	0.0025
CTNNB1_P757_F	0.0723	0.0300	0.0735	0.0324	0.0985	0.0054	0.0614	0.1029	0.0000	0.0000	0.0087	0.0071	0.0032	0.0395	0.0082
CTSD_P726_F	0.0000	0.0000	0.0000	0.0000	0.0000	0.0000	0.0288	0.0000	0.0000	0.0000	0.0000	0.0000	0.0000	0.8212	0.0000
CTSH_P238_F	0.0397	0.0000	0.0000	0.0000	0.1163	0.0000	0.0000	0.0000	0.0000	0.0000	0.0000	0.0000	0.0000	0.0000	0.0000
CTSL_P264_R	0.0013	0.0000	0.0054	0.0000	0.0096	0.0192	0.0000	0.0016	0.0000	0.0000	0.0088	0.0103	0.2097	0.0000	0.0028
CTSL_P81_F	0.0000	0.0000	0.0019	0.0114	0.0000	0.0000	0.0051	0.0051	0.0000	0.0000	0.0078	0.0072	0.0000	0.0000	0.0083
CTTN_E29_R	0.0121	0.0000	0.0034	0.0108	0.0078	0.0082	0.0000	0.0000	0.0000	0.0000	0.0102	0.0154	0.0000	0.0096	0.0000
CYP1A1_P382_F	0.0742	0.0000	0.0350	0.0258	0.5925	0.3221	0.2567	0.5204	0.0000	0.0000	0.0298	0.0798	0.0463	0.0318	0.4801
CYP2E1_P416_F	0.2617	0.0000	0.2850	0.1415	0.0000	0.0013	0.0065	0.1722	0.0000	0.0000	0.0116	0.0000	0.0000	0.0000	0.0054
DAB2_P35_F	0.0203	0.0000	0.2536	0.0163	0.0000	0.0000	0.0134	0.0000	0.0000	0.0000	0.0000	0.0000	0.3438	0.0080	0.0000
DAB2_P468_F	0.9836	0.0084	0.0349	0.2122	0.1534	0.0397	0.1045	0.0392	0.0035	0.0361	0.0211	0.1566	0.2033	0.3110	0.0198
DAB2IP_P9_F	0.0000	0.0000	0.0000	0.0029	0.0000	0.0000	0.0000	0.0000	0.0000	0.0000	0.0000	0.0000	0.3684	0.0000	0.0000
DAPK1_P10_F	0.0089	0.0000	0.2724	0.0067	0.0000	0.0000	0.0000	0.0053	0.0000	0.0000	0.3852	0.0024	0.0000	0.7061	0.1412
DAPK1_P345_R	0.0000	0.0000	0.0000	0.0000	0.0000	0.0000	0.0000	0.0000	0.0000	0.0000	0.0063	0.0009	0.0996	0.0062	0.0020
DBC1_P351_R	0.0000	0.0000	0.0037	0.0000	0.0000	0.0030	0.0000	0.0000	0.0000	0.0000	0.0043	0.0000	0.0675	0.0000	0.0000
DCC_P177_F	0.3130	0.5924	0.0109	0.0106	0.0127	0.0000	0.0054	0.4402	0.0000	0.0000	0.0192	0.3702	0.1214	0.4609	0.0348
DCC_P471_R	0.0068	0.0000	0.0188	0.3556	0.0291	0.0390	0.2375	0.0083	0.0000	0.0000	0.0177	0.0084	0.1038	0.0150	0.2566
DCN_P1320_R	0.0000	0.0000	0.0000	0.0000	0.0000	0.0216	0.0000	0.0000	0.0000	0.0000	0.0000	0.0000	0.0000	0.0000	0.0000
DDB2_P407_F	0.0000	0.0000	0.0000	0.0143	0.0055	0.0018	0.0056	0.0000	0.0000	0.0000	0.0000	0.9810	0.3063	0.0000	0.0000
DDB2_P613_R	0.0140	0.0000	0.0041	0.0610	0.0094	0.4660	0.0000	0.1945	0.0000	0.0000	0.0080	0.0054	0.0000	0.0000	0.0048

DDR1_E23_R	0.0092	0.0000	0.0461	0.0000	0.0000	0.0021	0.0000	0.0000	0.0000	0.0000	0.0073	0.0063	0.0000	0.0000	0.0000
DDR2_E331_F	0.9940	0.0000	0.9744	0.0000	0.1911	0.9932	0.0000	0.9923	0.9640	0.0000	0.9783	0.9860	0.9728	0.0000	0.9936
DES_E228_R	0.0000	0.0000	0.0818	0.0000	0.0000	0.0000	0.0000	0.0054	0.0000	0.0000	0.0087	0.0000	0.0000	0.0000	0.0000
DHCR24_P406_R	0.1237	0.3831	0.3595	0.0134	0.0161	0.0170	0.0298	0.0763	0.0295	0.7304	0.0144	0.0117	0.1120	0.0087	0.1182
DIO3_E230_R	0.0112	0.0000	0.0820	0.0000	0.0087	0.0104	0.0101	0.0000	0.0000	0.0000	0.0098	0.0053	0.0000	0.0069	0.0042
DIO3_P674_F	0.0122	0.6507	0.1346	0.3160	0.0112	0.9863	0.2545	0.1789	0.0000	0.0000	0.5529	0.0127	0.7012	0.9711	0.0063
DIRAS3_E55_R	0.0000	0.0000	0.0270	0.0264	0.0000	0.0056	0.0000	0.0099	0.0000	0.0000	0.0244	0.0160	0.0313	0.0000	0.0183
DKC1_E101_F	0.0000	0.0000	0.2374	0.0000	0.0000	0.0000	0.0000	0.0000	0.0000	0.0000	0.0000	0.0000	0.0000	0.0000	0.0000
DKFZP564O0823_E45_F	0.0000	0.0000	0.2027	0.0115	0.0000	0.0000	0.0000	0.0260	0.3661	0.0000	0.2524	0.0000	0.0000	0.0000	0.0000
DLC1_P88_R	0.0060	0.0000	0.6676	0.0000	0.0000	0.0000	0.0032	0.0012	0.0000	0.6930	0.0029	0.0000	0.5975	0.0008	0.0000
DLK1_E227_R	0.9979	0.9341	0.9916	0.9975	0.9971	0.9972	0.9958	0.9969	0.9950	0.9954	0.9807	0.9414	0.9938	0.8879	0.9966
DLL1_P386_F	0.0000	0.0000	0.9716	0.0000	0.0077	0.0000	0.0000	0.0000	0.9786	0.9481	0.0000	0.0000	0.9137	0.0000	0.8553
DLL1_P832_F	0.0000	0.0000	0.1821	0.0000	0.0000	0.0000	0.0879	0.2936	0.0000	0.0000	0.5235	0.9901	0.2538	0.0051	0.0000
DMP1_P134_F	0.1592	0.2560	0.1162	0.0337	0.0143	0.0120	0.0095	0.2183	0.3398	0.0000	0.0106	0.0097	0.0120	0.1700	0.0166
DNAJC15_E26_R	0.0065	0.0000	0.0675	0.0076	0.0074	0.0049	0.0475	0.0000	0.0000	0.0000	0.0132	0.0066	0.0018	0.0084	0.0044
DNASE1L1_P108_F	0.2849	0.4950	0.9007	0.7708	0.5517	0.0325	0.6562	0.9904	0.9895	0.5808	0.5139	0.0204	0.9245	0.5685	0.5133
DNMT1_P100_R	0.2816	0.0000	0.9852	0.1119	0.9941	0.9952	0.0616	0.4291	0.9896	0.9433	0.9239	0.9843	0.9817	0.0175	0.9901
DSC2_E90_F	0.0000	0.0000	0.9559	0.0000	0.0000	0.9856	0.0000	0.0000	0.9085	0.8946	0.0000	0.0000	0.9536	0.0000	0.0000
DSP_P440_R	0.3213	0.3080	0.9813	0.9949	0.0223	0.3454	0.9912	0.3781	0.9855	0.7141	0.7078	0.9869	0.9359	0.7751	0.9919
DST_E31_F	0.0473	0.7397	0.7755	0.0000	0.9946	0.8282	0.0106	0.9939	0.5249	0.9884	0.9791	0.0214	0.4372	0.0099	0.2824
DST_P262_R	0.5653	0.9293	0.0572	0.0256	0.9130	0.1901	0.6072	0.5654	0.0481	0.0998	0.0814	0.0575	0.0584	0.0594	0.0399
DUSP4_E61_F	0.0000	0.0000	0.0046	0.0000	0.0101	0.0018	0.0000	0.0000	0.0000	0.0000	0.0087	0.0056	0.0000	0.0119	0.0000
DUSP4_P925_R	0.0190	0.2085	0.0250	0.0148	0.0208	0.6869	0.5369	0.4226	0.0039	0.0000	0.4952	0.5178	0.2000	0.0456	0.3992
E2F3_P840_R	0.0100	0.0000	0.7956	0.0000	0.0039	0.0000	0.0049	0.0027	0.0000	0.0000	0.0244	0.7476	0.4589	0.0000	0.6376
E2F5_P516_R	0.9862	0.8705	0.9487	0.1382	0.9860	0.0122	0.0000	0.0000	0.9633	0.9526	0.9150	0.9787	0.9470	0.9799	0.9741
EDN1_E50_R	0.4196	0.2555	0.2720	0.9943	0.9709	0.3439	0.5943	0.9486	0.5341	0.4425	0.4952	0.2122	0.4013	0.8221	0.9669
EDN1_P39_R	0.5194	0.9913	0.0173	0.9307	0.1824	0.0043	0.9012	0.7074	0.0000	0.0514	0.0350	0.9714	0.1857	0.9933	0.4862
EDNRB_P148_R	0.1773	0.0000	0.9776	0.0000	0.0000	0.0000	0.0000	0.0000	0.6996	0.0000	0.6946	0.0000	0.9729	0.0000	0.0000
EFNA1_P591_R	0.3813	0.8059	0.2360	0.3897	0.0094	0.1609	0.2997	0.0578	0.0000	0.0000	0.0041	0.0069	0.1698	0.0000	0.0189
EFNA1_P7_F	0.0000	0.0000	0.7155	0.0060	0.0000	0.0000	0.0061	0.0000	0.0000	0.3794	0.4837	0.0043	0.9740	0.0000	0.0002
EFNB1_E69_F	0.4618	0.0000	0.1489	0.4402	0.1221	0.2950	0.2288	0.2846	0.0000	0.0000	0.0107	0.9877	0.2644	0.0000	0.0076
EFNB3_P442_R	0.5819	0.0000	0.0018	0.0085	0.0001	0.0065	0.0000	0.0007	0.0000	0.0000	0.0078	0.0028	0.0000	0.0107	0.0000
EGF_E339_F	0.9863	0.0000	0.1583	0.0000	0.0000	0.0000	0.0029	0.0000	0.0000	0.0000	0.1789	0.0021	0.5453	0.0000	0.0007

EGFR_E295_R	0.2025	0.0595	0.0032	0.1918	0.0214	0.1214	0.0051	0.0028	0.0000	0.0000	0.0046	0.0050	0.1616	0.0143	0.0039
EGFR_P260_R	0.0000	0.0000	0.5169	0.0000	0.0000	0.0000	0.0000	0.0032	0.9864	0.0000	0.0017	0.0000	0.0000	0.9915	0.0000
EGR4_P479_F	0.4827	0.7726	0.9573	0.0267	0.9944	0.9924	0.5292	0.0104	0.9842	0.8776	0.7672	0.9896	0.9791	0.0655	0.0273
EIF2AK2_E103_R	0.0000	0.0000	0.9793	0.0000	0.0000	0.0000	0.0000	0.0000	0.0000	0.9854	0.9739	0.0000	0.9802	0.0000	0.0000
EIF2AK2_P313_F	0.9942	0.8369	0.9858	0.3420	0.0072	0.0895	0.1723	0.9961	0.9907	0.8820	0.9882	0.9916	0.9865	0.0000	0.0000
ELK1_E156_F	0.0000	0.0000	0.9202	0.0000	0.0106	0.0000	0.7837	0.0000	0.0000	0.0000	0.0000	0.0000	0.9514	0.0000	0.0000
EMR3_P39_R	0.4918	0.0045	0.6325	0.4097	0.3804	0.3487	0.3773	0.0416	0.4770	0.4800	0.8899	0.4238	0.6122	0.3856	0.9932
ENC1_P484_R	0.4311	0.6237	0.9822	0.0939	0.9953	0.0000	0.0000	0.0072	0.9877	0.9777	0.9850	0.9865	0.9846	0.0000	0.0000
EPHA1_E46_R	0.1692	0.0838	0.4910	0.8433	0.9673	0.2634	0.2168	0.0207	0.0042	0.0067	0.6927	0.0171	0.2931	0.2274	0.0463
EPHA1_P119_R	0.0086	0.0000	0.0924	0.0000	0.7583	0.0054	0.0064	0.0066	0.0000	0.0000	0.0052	0.0076	0.1031	0.0072	0.0071
EPHA2_P203_F	0.0106	0.4529	0.4202	0.0078	0.0065	0.0017	0.0053	0.8788	0.0000	0.0000	0.0067	0.0031	0.1022	0.0097	0.0058
EPHA3_E156_R	0.0040	0.0000	0.0001	0.0050	0.0000	0.0000	0.0025	0.0000	0.0000	0.0000	0.0033	0.0000	0.0000	0.0000	0.0000
EPHA7_E6_F	0.0000	0.0000	0.5026	0.0000	0.0000	0.0000	0.9943	0.0023	0.3587	0.8420	0.4240	0.0000	0.5056	0.4787	0.0030
EPHA7_P205_R	0.9950	0.4250	0.9887	0.0000	0.9894	0.9675	0.2156	0.9659	0.9573	0.9784	0.8479	0.9892	0.9869	0.0503	0.9850
EPHA8_P256_F	0.1786	0.0000	0.0996	0.0946	0.0166	0.1779	0.1471	0.0168	0.0027	0.2339	0.0127	0.1565	0.0171	0.0155	0.0675
EPHB1_P503_F	0.4834	0.0000	0.3019	0.0113	0.0086	0.0660	0.0069	0.0053	0.0000	0.0000	0.5375	0.0074	0.1579	0.0116	0.0042
EPHB2_E297_F	0.7615	0.3356	0.0160	0.8670	0.0341	0.0816	0.8465	0.7940	0.0000	0.6680	0.2013	0.0321	0.0180	0.9262	0.7817
EPHB2_P165_R	0.0112	0.0000	0.4536	0.0000	0.0000	0.0038	0.0000	0.0000	0.0000	0.0000	0.9769	0.0024	0.7188	0.0000	0.0000
EPHB3_E0_F	0.0047	0.0000	0.0000	0.0000	0.0000	0.8726	0.0000	0.0000	0.0000	0.0000	0.9269	0.0010	0.5754	0.0000	0.6715
EPHB3_P569_R	0.1128	0.3303	0.0204	0.2692	0.0206	0.2923	0.3443	0.0051	0.0000	0.0000	0.0215	0.2099	0.0099	0.0068	0.0702
EPHB4_P313_R	0.7993	0.4108	0.2341	0.8005	0.0120	0.0000	0.7078	0.6804	0.5706	0.0000	0.2167	0.0385	0.2523	0.2788	0.4124
EPHB6_E342_F	0.8036	0.0000	0.2727	0.0532	0.7840	0.0347	0.0553	0.7907	0.3735	0.0000	0.0780	0.8047	0.0518	0.5690	0.7490
EPHB6_P827_R	0.0126	0.2983	0.0030	0.0000	0.0000	0.0077	0.0094	0.0000	0.4727	0.0000	0.0095	0.0040	0.3497	0.0000	0.0000
EPHX1_P1358_R	0.9121	0.0000	0.9825	0.0000	0.0000	0.0000	0.0000	0.0043	0.9885	0.9690	0.8612	0.0076	0.9703	0.0051	0.9905
EPM2A_P113_F	0.0541	0.9394	0.0403	0.8951	0.0793	0.8518	0.0700	0.8108	0.0343	0.7738	0.0270	0.7104	0.1773	0.0950	0.7982
EPM2A_P64_R	0.3833	0.1073	0.0174	0.6535	0.3771	0.0281	0.5236	0.3878	0.0000	0.0000	0.0205	0.0136	0.0078	0.0365	0.0178
EPO_E244_R	0.0050	0.4421	0.0263	0.0000	0.0000	0.0000	0.0000	0.0000	0.0000	0.0000	0.9593	0.6466	0.0000	0.0082	0.0007
EPO_P162_R	0.0000	0.0000	0.2274	0.0000	0.0037	0.0051	0.0000	0.0000	0.0000	0.2260	0.0033	0.0000	0.0000	0.0048	0.0023
EPS8_E231_F	0.0480	0.2735	0.9205	0.0000	0.1436	0.2222	0.1473	0.0679	0.9613	0.5780	0.0263	0.0000	0.9607	0.0000	0.0000
EPS8_P437_F	0.0138	0.0000	0.7194	0.0000	0.0000	0.0000	0.0000	0.0031	0.0000	0.4327	0.0000	0.0069	0.0000	0.0000	0.0000
ERBB2_P59_R	0.4843	0.0000	0.8302	0.0000	0.0000	0.0031	0.9949	0.7557	0.0000	0.7427	0.9741	0.9893	0.8678	0.9927	0.0000
ERBB3_E331_F	0.9945	0.6237	0.6163	0.6606	0.6077	0.5415	0.6429	0.5903	0.0000	0.1520	0.0155	0.7770	0.4919	0.8610	0.0143
ERBB3_P870_R	0.2111	0.0000	0.0120	0.0088	0.0140	0.0760	0.0000	0.2140	0.0000	0.0000	0.0266	0.1703	0.1278	0.0178	0.0000

ERBB4_P255_F	0.5476	0.0000	0.0185	0.1754	0.0065	0.0065	0.4249	0.5328	0.3104	0.0000	0.0164	0.4992	0.0169	0.0140	0.0158
ERBB4_P541_F	0.0099	0.0000	0.8923	0.0000	0.9958	0.1381	0.0000	0.0114	0.6398	0.9276	0.7897	0.9865	0.8300	0.0000	0.0215
ERCC1_P354_F	0.2683	0.6777	0.8677	0.0000	0.0000	0.8390	0.9939	0.6825	0.9883	0.6588	0.7661	0.9855	0.7677	0.0092	0.9951
ERCC3_P1210_R	0.1767	0.8227	0.7977	0.4976	0.3679	0.2726	0.2970	0.2530	0.9587	0.9882	0.7179	0.2945	0.9816	0.0288	0.8547
ERG_E28_F	0.9903	0.7872	0.5708	0.0289	0.5181	0.0356	0.0264	0.0117	0.0212	0.7481	0.0246	0.0320	0.1875	0.0536	0.6687
ERN1_P809_R	0.0579	0.2961	0.9366	0.1421	0.2179	0.9940	0.3030	0.0049	0.0191	0.0000	0.9802	0.0818	0.9843	0.2898	0.9912
ESR1_E298_R	0.7659	0.9341	0.9595	0.0368	0.9957	0.0854	0.9954	0.7290	0.9401	0.0429	0.6920	0.7965	0.9643	0.1625	0.7295
ESR1_P151_R	0.0109	0.0000	0.4374	0.2256	0.1276	0.0000	0.9944	0.9936	0.9749	0.0000	0.0138	0.9864	0.7165	0.0000	0.0041
ESR2_E66_F	0.3306	0.1597	0.5805	0.4549	0.5276	0.4607	0.2521	0.3478	0.0460	0.3715	0.3391	0.6645	0.1674	0.1083	0.0550
ESR2_P162_F	0.2170	0.1297	0.2350	0.3594	0.0236	0.2975	0.3067	0.2825	0.9343	0.0143	0.0314	0.0175	0.3543	0.0440	0.3117
ETS1_E253_R	0.1308	0.1046	0.4136	0.1399	0.0254	0.0205	0.1297	0.0137	0.0000	0.0000	0.1603	0.0367	0.0223	0.2091	0.1127
ETS1_P559_R	0.0000	0.0000	0.9756	0.0000	0.9946	0.9814	0.0000	0.0905	0.0000	0.9862	0.9796	0.9899	0.9839	0.0000	0.0000
ETS2_P684_F	0.8247	0.8915	0.8328	0.6549	0.5913	0.0183	0.5736	0.0140	0.9845	0.7625	0.9261	0.3929	0.9323	0.0351	0.0092
ETS2_P835_F	0.0444	0.0000	0.0052	0.0000	0.0000	0.0004	0.2490	0.0000	0.0000	0.0000	0.0036	0.0000	0.0000	0.1586	0.1574
ETV1_P235_F	0.0065	0.0000	0.9763	0.9921	0.1762	0.0106	0.0024	0.6941	0.9144	0.8170	0.9762	0.3356	0.9758	0.0000	0.0000
ETV1_P515_F	0.0000	0.0000	0.4000	0.0169	0.0058	0.0000	0.0000	0.0078	0.0000	0.0000	0.0084	0.0000	0.8915	0.0108	0.0101
ETV6_E430_F	0.1990	0.0000	0.3534	0.0104	0.0658	0.0057	0.1068	0.0058	0.3284	0.7428	0.4400	0.3984	0.2165	0.0212	0.0078
EVI1_E47_R	0.9913	0.4473	0.9410	0.9935	0.0179	0.5861	0.5435	0.5503	0.0000	0.0000	0.8620	0.1544	0.9448	0.3507	0.9901
EVI1_P30_R	0.0078	0.0000	0.0512	0.0067	0.0084	0.0026	0.0099	0.9943	0.0000	0.1247	0.5030	0.0072	0.0645	0.4643	0.0000
EVI2A_P94_R	0.0000	0.0000	0.0202	0.0000	0.0070	0.0000	0.2022	0.0000	0.4018	0.0000	0.0084	0.0000	0.0000	0.0115	0.0114
EXT1_E197_F	0.3660	0.3116	0.1177	0.3038	0.4327	0.0104	0.2308	0.2797	0.0000	0.0000	0.3585	0.0151	0.3775	0.5658	0.8035
EYA4_E277_F	0.1146	0.0000	0.1708	0.0000	0.2039	0.0079	0.0000	0.0000	0.0000	0.8636	0.0076	0.9889	0.0824	0.0000	0.0702
EYA4_P508_F	0.0000	0.0000	0.6613	0.9945	0.0000	0.0000	0.0000	0.4525	0.0000	0.5671	0.3248	0.0000	0.9139	0.0068	0.0000
EYA4_P794_F	0.0000	0.0000	0.7133	0.0000	0.0034	0.0000	0.1045	0.0000	0.0356	0.9851	0.6320	0.9901	0.8360	0.0180	0.0000
F2R_P839_F	0.4563	0.0000	0.9838	0.0000	0.0000	0.0272	0.0086	0.8539	0.9729	0.9468	0.9072	0.9665	0.9811	0.0000	0.0000
F2R_P88_F	0.0372	0.0000	0.7357	0.0091	0.0000	0.0030	0.0108	0.0019	0.0000	0.6185	0.3570	0.0000	0.9677	0.0000	0.0052
FABP3_E113_F	0.6637	0.0000	0.5467	0.0106	0.7897	0.0045	0.0027	0.7198	0.6750	0.0000	0.4113	0.0081	0.5347	0.0000	0.0130
FABP3_P598_F	0.6731	0.9983	0.2536	0.3480	0.8167	0.4079	0.9942	0.9060	0.5627	0.2958	0.2666	0.7468	0.4047	0.8420	0.9766
FANCE_P356_R	0.0000	0.0000	0.6607	0.0000	0.0000	0.0017	0.0121	0.2039	0.5137	0.0000	0.0043	0.0000	0.7364	0.0000	0.0000
FANCF_P13_F	0.6168	0.8621	0.8173	0.6797	0.7635	0.6322	0.6690	0.6207	0.0000	0.1054	0.5917	0.0883	0.6299	0.8024	0.6425
FANCG_E207_R	0.0793	0.9905	0.0859	0.9748	0.1051	0.0786	0.9631	0.0394	0.2239	0.2463	0.0585	0.1157	0.0799	0.5798	0.2201
FAS_P322_R	0.6152	0.0000	0.7776	0.9802	0.0000	0.0000	0.6475	0.0000	0.4212	0.9557	0.6516	0.1897	0.7686	0.0000	0.0273
FASTK_P257_F	0.1062	0.0172	0.7634	0.0724	0.9143	0.0947	0.0963	0.0862	0.0415	0.7355	0.0883	0.0758	0.6074	0.0535	0.0945

FAT_P973_R	0.0990	0.0000	0.3510	0.0000	0.0000	0.0000	0.4910	0.2558	0.9274	0.0000	0.0197	0.0119	0.6430	0.0068	0.0000
FER_P581_F	0.0867	0.6376	0.9428	0.3016	0.0629	0.1953	0.9930	0.9922	0.9798	0.2280	0.9708	0.2129	0.8882	0.1323	0.2258
FES_P223_R	0.0000	0.0000	0.9650	0.0000	0.0000	0.0000	0.0000	0.0000	0.9803	0.9795	0.0000	0.0000	0.9713	0.0000	0.9865
FGF1_E5_F	0.4215	0.0367	0.8985	0.1983	0.3242	0.1156	0.1120	0.1227	0.9785	0.8686	0.9679	0.7066	0.2931	0.1223	0.8220
FGF12_E61_R	0.4012	0.2732	0.3669	0.2488	0.0067	0.0093	0.4691	0.0070	0.7523	0.0000	0.0175	0.0135	0.2189	0.2755	0.0113
FGF12_P210_R	0.2514	0.0000	0.9834	0.4070	0.3716	0.4965	0.0000	0.9946	0.9879	0.9866	0.9851	0.9900	0.9805	0.9907	0.9896
FGF2_P153_F	0.2040	0.1268	0.4412	0.1961	0.1913	0.0533	0.1954	0.0111	0.0000	0.5126	0.0255	0.0112	0.5017	0.0251	0.1790
FGF2_P229_F	0.2530	0.6514	0.5565	0.4911	0.0408	0.0575	0.0339	0.0324	0.5055	0.0385	0.0470	0.2349	0.4281	0.9938	0.0368
FGF3_E198_R	0.9906	0.9617	0.6545	0.9945	0.9926	0.9907	0.9931	0.9889	0.9721	0.9788	0.6502	0.9801	0.4280	0.9941	0.5515
FGF3_P171_R	0.9865	0.0000	0.8711	0.0000	0.0000	0.0000	0.8201	0.9124	0.9848	0.5708	0.7927	0.7926	0.9628	0.0000	0.0000
FGF5_E16_F	0.9943	0.4764	0.9472	0.9958	0.0000	0.0102	0.0000	0.0307	0.9426	0.5496	0.9751	0.0045	0.8171	0.9897	0.9942
FGF5_P238_R	0.0000	0.0000	0.3333	0.0091	0.0015	0.3590	0.0065	0.0000	0.6053	0.4723	0.4522	0.5550	0.3926	0.0046	0.0000
FGF6_E294_F	0.7643	0.9456	0.3448	0.0767	0.2259	0.7962	0.7399	0.1091	0.0168	0.9727	0.9750	0.6801	0.6806	0.0652	0.0525
FGF7_P44_F	0.0000	0.0969	0.4700	0.0000	0.0108	0.0000	0.0000	0.0000	0.0000	0.0000	0.0000	0.0000	0.6752	0.0065	0.0000
FGF8_E183_F	0.0875	0.6239	0.0543	0.1878	0.0000	0.0000	0.0000	0.0000	0.0000	0.0000	0.0182	0.1125	0.0000	0.1667	0.2384
FGF8_P473_F	0.0000	0.0000	0.8463	0.0000	0.0020	0.0220	0.0000	0.9902	0.3672	0.9686	0.0000	0.0000	0.2640	0.0034	0.0000
FGFR1_E317_F	0.0575	0.0000	0.0708	0.0000	0.0498	0.0210	0.2435	0.0813	0.0000	0.5868	0.0175	0.0105	0.4944	0.0000	0.0101
FGFR2_P266_R	0.1600	0.4548	0.0141	0.0090	0.2280	0.0111	0.6427	0.0083	0.0000	0.0000	0.0140	0.3179	0.0040	0.0148	0.0076
FGFR3_E297_R	0.9926	0.0000	0.6844	0.0000	0.0000	0.0000	0.9925	0.0079	0.9553	0.9820	0.0065	0.0000	0.9673	0.0000	0.0000
FGFR3_P1152_R	0.9911	0.6699	0.8402	0.9976	0.0039	0.3928	0.0041	0.9954	0.8112	0.8827	0.6616	0.7308	0.8864	0.9945	0.9945
FHIT_E19_R	0.1609	0.3932	0.9799	0.0000	0.0000	0.0184	0.0000	0.0000	0.9841	0.9902	0.9341	0.0054	0.9821	0.0000	0.9941
FHIT_P93_R	0.0000	0.0000	0.0736	0.0000	0.0040	0.0000	0.0053	0.0023	0.2084	0.9410	0.6115	0.0067	0.1625	0.0081	0.0000
FHL1_E229_R	0.0055	0.0000	0.1388	0.0000	0.0000	0.0000	0.0000	0.0000	0.0000	0.0000	0.0024	0.0000	0.0697	0.0053	0.0000
FLI1_P620_R	0.9952	0.0000	0.9755	0.0000	0.0000	0.0001	0.0000	0.0000	0.9378	0.9695	0.9802	0.6693	0.9531	0.0000	0.9859
FLT1_E444_F	0.0145	0.5533	0.7309	0.8506	0.5140	0.9929	0.6941	0.0147	0.7035	0.4678	0.5624	0.0141	0.7111	0.0509	0.0177
FLT1_P302_F	0.1407	0.0595	0.0210	0.0263	0.1078	0.0504	0.2116	0.1579	0.0000	0.0000	0.0292	0.0238	0.1301	0.0365	0.1896
FLT1_P615_R	0.1145	0.0462	0.3050	0.2064	0.0126	0.0092	0.1710	0.0065	0.0000	0.0000	0.7018	0.0068	0.0379	0.4595	0.1184
FLT3_E326_R	0.0157	0.0000	0.3917	0.3482	0.0100	0.0000	0.0000	0.0000	0.4368	0.0000	0.0100	0.0067	0.0784	0.0194	0.0056
FLT4_P180_R	0.4257	0.0843	0.9736	0.0516	0.0304	0.8252	0.7536	0.8122	0.9850	0.8235	0.8798	0.9741	0.9817	0.9898	0.9617
FMR1_P62_R	0.9172	0.7703	0.2291	0.9426	0.9369	0.6137	0.6763	0.9048	0.3379	0.8843	0.3574	0.9040	0.3361	0.9707	0.5616
FN1_E469_F	0.0090	0.0000	0.2874	0.0000	0.0057	0.0021	0.0000	0.0000	0.0000	0.0000	0.0007	0.9880	0.0000	0.0038	0.0000
FN1_P229_R	0.0242	0.0000	0.3380	0.2637	0.2402	0.0167	0.8366	0.0082	0.0005	0.1453	0.6351	0.7028	0.2086	0.0074	0.9403
FOSL2_E384_R	0.9952	0.7721	0.9675	0.0000	0.8294	0.9951	0.9956	0.9078	0.9863	0.6552	0.9739	0.1082	0.9853	0.9125	0.9928

FRK_P36_F	0.0114	0.0000	0.0108	0.0140	0.0000	0.0169	0.0061	0.0979	0.0000	0.0000	0.0139	0.0247	0.0802	0.0000	0.0000
FRZB_E186_R	0.0470	0.0000	0.9428	0.0000	0.0000	0.1259	0.0349	0.0000	0.8565	0.9737	0.0058	0.1471	0.9355	0.0000	0.0000
FRZB_P406_F	0.0000	0.3206	0.0011	0.0000	0.0000	0.0000	0.0000	0.0000	0.0000	0.0000	0.0000	0.0000	0.0956	0.1923	0.0000
FVT1_P225_F	0.0131	0.0000	0.2101	0.0096	0.2571	0.0089	0.0061	0.0055	0.0000	0.0000	0.6194	0.0055	0.1462	0.0084	0.0056
FYN_P352_R	0.9927	0.9996	0.9475	0.9969	0.9960	0.9938	0.9969	0.9961	0.9843	0.9888	0.9186	0.9939	0.9882	0.9983	0.9926
FZD7_E296_F	0.0266	0.0000	0.9727	0.0000	0.0000	0.0000	0.0000	0.9909	0.9827	0.0000	0.8393	0.0000	0.9780	0.9935	0.0000
FZD9_E458_F	0.0000	0.0000	0.9612	0.0000	0.9929	0.0000	0.0000	0.0000	0.8851	0.0000	0.9044	0.0000	0.9648	0.0000	0.9903
FZD9_P15_R	0.0115	0.1900	0.1230	0.3619	0.0252	0.0062	0.0000	0.0000	0.0000	0.0000	0.0070	0.0000	0.0000	0.0093	0.0000
FZD9_P175_F	0.1456	0.0491	0.4803	0.6757	0.0141	0.0088	0.0098	0.0082	0.7530	0.3849	0.4058	0.1273	0.3895	0.0971	0.0057
G6PD_E190_F	0.0000	0.0000	0.6588	0.0000	0.0000	0.0000	0.0000	0.0000	0.0000	0.0543	0.0030	0.9886	0.6139	0.9942	0.0000
G6PD_P196_F	0.6915	0.8462	0.4985	0.7132	0.0178	0.6296	0.5878	0.5734	0.1521	0.6772	0.0134	0.5106	0.5085	0.7600	0.5672
GABRB3_E42_F	0.0000	0.0000	0.0058	0.0008	0.0058	0.8848	0.0000	0.0000	0.0000	0.0000	0.0028	0.0073	0.0567	0.0000	0.0000
GADD45A_P737_R	0.3694	0.8349	0.2019	0.0365	0.0191	0.0207	0.8871	0.0197	0.0047	0.0341	0.0309	0.9902	0.0721	0.7912	0.0233
GALR1_E52_F	0.3384	0.0000	0.0126	0.0301	0.0151	0.1689	0.4344	0.3120	0.9753	0.0000	0.0243	0.1819	0.0000	0.0098	0.4238
GALR1_P80_F	0.0353	0.0000	0.0071	0.0000	0.3117	0.0120	0.0613	0.0000	0.0000	0.0000	0.0113	0.0065	0.0011	0.0112	0.0067
GAS1_E22_F	0.0092	0.0000	0.0087	0.0127	0.0000	0.0664	0.0099	0.0000	0.0000	0.0000	0.0109	0.0061	0.0021	0.0137	0.0681
GAS1_P754_R	0.0000	0.2955	0.0318	0.0000	0.0000	0.0000	0.0059	0.0009	0.0000	0.0000	0.0045	0.0016	0.0000	0.0000	0.0022
GAS7_E148_F	0.5197	0.0000	0.0137	0.0137	0.0146	0.4471	0.5262	0.5729	0.0000	0.0000	0.0260	0.0181	0.0064	0.0000	0.0103
GAS7_P622_R	0.0000	0.0000	0.0000	0.0000	0.0000	0.0000	0.0000	0.0000	0.0000	0.0000	0.0000	0.0000	0.0000	0.0000	0.0000
GATA6_P21_R	0.0000	0.0000	0.0068	0.0000	0.0048	0.0072	0.0043	0.0030	0.0000	0.0000	0.2346	0.0042	0.0014	0.0124	0.0000
GATA6_P726_F	0.1911	0.0000	0.0099	0.0454	0.1848	0.0198	0.0148	0.4171	0.0000	0.0000	0.0204	0.2226	0.0043	0.4147	0.0105
GFI1_E136_F	0.2538	0.0000	0.3718	0.2356	0.1704	0.0000	0.0000	0.0000	0.0000	0.0000	0.0575	0.1421	0.0000	0.0040	0.0103
GFI1_P45_R	0.0113	0.0000	0.0058	0.0098	0.0038	0.0029	0.0100	0.0049	0.0000	0.2614	0.0099	0.0054	0.0004	0.0000	0.0034
GJB2_E43_F	0.1405	0.0000	0.0152	0.1206	0.0000	0.0066	0.0000	0.0060	0.0000	0.0000	0.0136	0.0122	0.2995	0.0000	0.1779
GJB2_P791_R	0.2004	0.0623	0.0097	0.2392	0.0207	0.1845	0.1121	0.1182	0.0000	0.0016	0.0096	0.1121	0.0025	0.6170	0.1869
GJB2_P931_R	0.3106	0.1432	0.0275	0.3805	0.3645	0.0510	0.3686	0.2842	0.0307	0.2171	0.6468	0.0220	0.0304	0.1054	0.3034
GLA_P112_F	0.0270	0.1862	0.8198	0.0045	0.0090	0.3390	0.0000	0.0247	0.0000	0.9875	0.0494	0.0338	0.9064	0.0000	0.0193
GLI2_E90_F	0.0855	0.0000	0.9797	0.0000	0.0000	0.9874	0.0000	0.0000	0.9830	0.9872	0.9773	0.9937	0.9840	0.0000	0.9936
GLI3_E148_R	0.1579	0.0685	0.9622	0.1100	0.9862	0.9845	0.0788	0.9781	0.9857	0.9389	0.7858	0.1028	0.9754	0.2259	0.8257
GML_P281_R	0.9947	0.6393	0.8867	0.9962	0.0000	0.0000	0.0000	0.0000	0.8833	0.9893	0.7530	0.9888	0.9763	0.0000	0.9919
GNAS_E58_F	0.9165	0.4679	0.9534	0.0000	0.0000	0.0029	0.0000	0.0000	0.0000	0.0000	0.4435	0.0070	0.9780	0.0000	0.0000
GP1BB_E23_F	0.0104	0.0000	0.0083	0.0125	0.9948	0.0020	0.0063	0.0989	0.0000	0.0000	0.0088	0.0059	0.0013	0.0053	0.0053
GPC3_P235_R	0.0000	0.4331	0.4290	0.0000	0.0084	0.0053	0.0000	0.0000	0.0000	0.0000	0.0071	0.0053	0.6001	0.0056	0.0000

GPR116_E328_R	0.9940	0.4965	0.9866	0.0000	0.9965	0.0000	0.0000	0.9933	0.9910	0.9893	0.9856	0.9848	0.9845	0.0000	0.9914
GPX1_E46_R	0.0123	0.0000	0.0061	0.1308	0.0416	0.0044	0.0748	0.2204	0.0000	0.0000	0.0116	0.1721	0.0033	0.2193	0.0819
GPX1_P194_F	0.4962	0.3584	0.4462	0.8168	0.1439	0.9945	0.5212	0.0312	0.0000	0.0167	0.7829	0.3518	0.1504	0.0924	0.4260
GPX3_E178_F	0.0140	0.0448	0.1730	0.0106	0.0137	0.0106	0.0104	0.0096	0.1221	0.2245	0.2672	0.0131	0.1593	0.0132	0.0119
GRB10_E85_R	0.0149	0.5733	0.0077	0.0087	0.0000	0.0061	0.0105	0.0102	0.0000	0.0000	0.0070	0.0000	0.0000	0.0074	0.0001
GRB10_P260_F	0.2807	0.0000	0.0000	0.0000	0.0845	0.0227	0.1737	0.3069	0.0000	0.0000	0.0205	0.0061	0.0000	0.0133	0.0099
GRB10_P496_R	0.0000	0.7353	0.1598	0.3978	0.2281	0.2726	0.0000	0.0000	0.0000	0.0000	0.4033	0.0104	0.2757	0.0231	0.0925
GRPR_P200_R	0.1939	0.1914	0.9415	0.2269	0.1700	0.1607	0.2164	0.1764	0.6324	0.7363	0.1992	0.9812	0.8795	0.2924	0.1773
GSTM1_P363_F	0.0913	0.0676	0.5413	0.1650	0.2371	0.1167	0.0334	0.0604	0.9782	0.0000	0.7226	0.0056	0.9449	0.1407	0.0000
GSTM2_E153_F	0.0070	0.0000	0.2060	0.0000	0.0042	0.0000	0.0049	0.0000	0.0000	0.0000	0.0046	0.0041	0.0830	0.6452	0.0008
GSTM2_P109_R	0.0000	0.0000	0.0212	0.0103	0.0000	0.0025	0.0000	0.0000	0.0000	0.0000	0.0000	0.0038	0.0000	0.0000	0.0000
GSTM2_P453_R	0.0912	0.0000	0.8451	0.0000	0.0000	0.9277	0.0000	0.0063	0.9681	0.8778	0.9833	0.0005	0.7426	0.0000	0.0007
GSTP1_E322_R	0.0000	0.0000	0.1204	0.0000	0.0000	0.0000	0.0069	0.0000	0.0000	0.0000	0.0139	0.0058	0.1039	0.0000	0.2825
GSTP1_P74_F	0.2697	0.7347	0.2693	0.0590	0.1601	0.0163	0.1433	0.2474	0.0000	0.0000	0.2607	0.0526	0.1197	0.0000	0.0250
GSTP1_seq_38_S153_R	0.0000	0.0000	0.0102	0.0000	0.0000	0.0000	0.0000	0.0000	0.0000	0.0000	0.0086	0.0000	0.0000	0.0000	0.0000
GUCY2D_E419_R	0.0000	0.0000	0.0017	0.0000	0.0000	0.0020	0.0000	0.0029	0.0000	0.0000	0.0071	0.0146	0.1813	0.0238	0.0048
HBEGF_P32_R	0.0000	0.0000	0.0012	0.0000	0.0000	0.0023	0.0000	0.0000	0.0000	0.0000	0.0036	0.0144	0.0000	0.0000	0.0041
HBII-13_E48_F	0.0000	0.0000	0.9599	0.0000	0.0000	0.0000	0.0062	0.0000	0.0000	0.9707	0.7084	0.8566	0.9635	0.0000	0.9896
HBII-52_P563_F	0.0426	0.1520	0.7971	0.6984	0.6775	0.0743	0.6112	0.0335	0.0569	0.0310	0.4500	0.5721	0.9143	0.0396	0.7194
HCK_P46_R	0.0417	0.0000	0.0760	0.0000	0.0000	0.0130	0.0000	0.0000	0.0000	0.0000	0.0180	0.0000	0.0007	0.0188	0.0135
HCK_P858_F	0.5190	0.2183	0.0102	0.6956	0.0228	0.6200	0.6542	0.0198	0.0000	0.7950	0.0446	0.0353	0.0063	0.8571	0.0374
HDAC1_P414_R	0.0000	0.0000	0.3725	0.0133	0.0089	0.0080	0.0000	0.0048	0.0000	0.0000	0.0083	0.0048	0.2776	0.0000	0.1122
HDAC11_P556_F	0.0000	0.0000	0.0023	0.0125	0.0104	0.0000	0.0072	0.0000	0.0000	0.0000	0.0048	0.0000	0.0000	0.4304	0.0044
HDAC9_P137_R	0.0018	0.0000	0.1434	0.0000	0.0000	0.0000	0.0000	0.0000	0.0000	0.0000	0.8690	0.0028	0.0894	0.0000	0.0000
HFE_E273_R	0.0000	0.0000	0.0004	0.0000	0.0066	0.0000	0.0000	0.0041	0.0000	0.0000	0.0061	0.0000	0.0000	0.5719	0.0000
HHIP_P307_R	0.1939	0.0312	0.0218	0.0564	0.0183	0.0460	0.0140	0.0234	0.0144	0.0226	0.2844	0.0598	0.0220	0.0389	0.0371
HIC1_E151_F	0.0000	0.0000	0.2429	0.0000	0.0000	0.0000	0.0000	0.0000	0.0000	0.0000	0.0000	0.0000	0.2222	0.0000	0.0000
HIC1_P565_R	0.0246	0.0000	0.0094	0.0000	0.0070	0.0000	0.0000	0.0081	0.0000	0.0000	0.0246	0.0000	0.0025	0.0107	0.0000
HIC2_P498_F	0.0000	0.0000	0.0046	0.0084	0.0092	0.0048	0.0000	0.0000	0.0000	0.0000	0.0065	0.0071	0.0000	0.0000	0.0000
HIC2_P528_R	0.0000	0.0000	0.0488	0.0212	0.0138	0.0090	0.0148	0.0000	0.0000	0.0000	0.0152	0.0053	0.0000	0.0202	0.0081
HIF1A_P488_F	0.0000	0.0000	0.0026	0.0000	0.0000	0.0000	0.0000	0.0093	0.0000	0.0000	0.0085	0.0000	0.0000	0.0000	0.0071
HLA-DOB_P357_R	0.9913	0.0000	0.9769	0.9955	0.0000	0.9874	0.0000	0.9895	0.9844	0.9735	0.9347	0.0000	0.9763	0.9781	0.9922
HLA-DPA1_P205_R	0.0091	0.0000	0.1024	0.1434	0.1177	0.1552	0.1329	0.1552	0.0000	0.0000	0.0141	0.9890	0.0077	0.4858	0.1395

HLA-DPA1_P28_R	0.0075	0.0000	0.1718	0.0000	0.0000	0.0029	0.0000	0.0000	0.0000	0.0000	0.0091	0.0000	0.0000	0.0095	0.0000
HLA-DPB1_E2_R	0.0535	0.0000	0.4063	0.0739	0.2877	0.0475	0.5408	0.0219	0.0000	0.0000	0.0347	0.0371	0.0273	0.0000	0.0485
HLA-DRA_P132_R	0.0000	0.0000	0.1393	0.0000	0.0000	0.0000	0.0000	0.0011	0.0000	0.0000	0.0000	0.0000	0.0000	0.0000	0.0000
HLA-F_E402_F	0.0000	0.0000	0.0000	0.0000	0.0000	0.0000	0.0006	0.0000	0.0000	0.0000	0.0000	0.0000	0.0000	0.0031	0.0000
HLF_E192_F	0.6177	0.5196	0.0123	0.0322	0.7062	0.0286	0.0237	0.5608	0.0012	0.3623	0.0195	0.0248	0.0096	0.0636	0.0299
HOXA11_E35_F	0.0000	0.0000	0.0018	0.0000	0.0079	0.0000	0.1259	0.0000	0.0807	0.0000	0.0026	0.0039	0.0457	0.0045	0.0064
HOXA11_P698_F	0.5799	0.9047	0.0170	0.7403	0.6702	0.1643	0.6602	0.5083	0.0000	0.0000	0.0274	0.4893	0.0098	0.7956	0.5822
HOXA5_E187_F	0.9943	0.0000	0.9830	0.0000	0.0000	0.9100	0.0043	0.0015	0.9859	0.9898	0.9734	0.9873	0.9782	0.0000	0.0000
HOXA9_E252_R	0.0164	0.0000	0.0091	0.0000	0.0000	0.0529	0.0000	0.0000	0.0000	0.0000	0.0169	0.0000	0.1648	0.0098	0.0115
HOXA9_P1141_R	0.1943	0.0521	0.0207	0.0225	0.1664	0.0226	0.1900	0.0089	0.1739	0.0000	0.0366	0.1832	0.0114	0.0134	0.0132
HOXA9_P303_F	0.0000	0.0000	0.0000	0.0000	0.0030	0.0000	0.0000	0.0000	0.0000	0.0000	0.0028	0.0009	0.0000	0.0040	0.0000
HOXB13_E21_F	0.0069	0.0000	0.0044	0.0087	0.0047	0.0020	0.0000	0.0037	0.0000	0.0000	0.1180	0.0039	0.0236	0.0056	0.0000
HOXB13_P17_R	0.0064	0.0000	0.2602	0.0000	0.0030	0.0000	0.0000	0.0000	0.0000	0.1808	0.0061	0.0000	0.0747	0.0008	0.0000
HOXC6_P456_R	0.5373	0.5634	0.2137	0.0461	0.6858	0.5560	0.4158	0.4474	0.0229	0.0472	0.0613	0.0318	0.1399	0.2089	0.5895
HPN_P374_R	0.0000	0.2621	0.2909	0.0000	0.0000	0.0000	0.0000	0.0045	0.0000	0.0000	0.0042	0.0000	0.2752	0.5926	0.0000
HPN_P823_F	0.0062	0.0000	0.8688	0.0000	0.0000	0.0031	0.7286	0.9926	0.0000	0.0000	0.0000	0.9870	0.5143	0.0011	0.0019
HPSE_P29_F	0.0312	0.8715	0.0201	0.7277	0.0282	0.5343	0.5315	0.0207	0.0000	0.0000	0.0266	0.0221	0.0595	0.9838	0.0173
HPSE_P93_F	0.0093	0.1717	0.1380	0.0000	0.0000	0.0000	0.0000	0.0058	0.0000	0.0000	0.0394	0.0000	0.0000	0.0000	0.0040
HRASLS_E72_R	0.0061	0.0000	0.0007	0.0000	0.0000	0.0000	0.0000	0.0008	0.0000	0.0000	0.0020	0.0021	0.1799	0.0024	0.0000
HS3ST2_E145_R	0.0000	0.0000	0.0000	0.0000	0.0000	0.0000	0.0041	0.0000	0.0000	0.0000	0.0064	0.0007	0.0647	0.0088	0.0012
HS3ST2_P546_F	0.0073	0.0000	0.0936	0.0108	0.0095	0.0023	0.0000	0.0000	0.0000	0.0000	0.0114	0.0013	0.0000	0.0062	0.0000
HSD17B12_E145_R	0.3285	0.0000	0.0154	0.1488	0.0000	0.0047	0.2335	0.5025	0.0000	0.0000	0.0209	0.0261	0.0109	0.9753	0.0000
HTR1B_P222_F	0.0000	0.5275	0.0000	0.1087	0.0065	0.0000	0.0042	0.0000	0.0000	0.0000	0.0042	0.0000	0.0000	0.1554	0.0196
HTR2A_P853_F	0.0098	0.4784	0.1721	0.0000	0.0049	0.0069	0.0000	0.0054	0.0000	0.0000	0.0046	0.0071	0.0000	0.0000	0.0036
IAPP_E280_F	0.0053	0.0000	0.9759	0.0000	0.0000	0.0000	0.0000	0.9712	0.8625	0.9839	0.9225	0.9880	0.9741	0.0000	0.0000
ICA1_P61_F	0.0000	0.0000	0.0000	0.0285	0.0000	0.0000	0.0211	0.0118	0.0000	0.0000	0.0000	0.0000	0.0000	0.0000	0.0062
ICA1_P72_R	0.0000	0.0000	0.0000	0.0000	0.0000	0.0040	0.0068	0.0029	0.5912	0.0000	0.0044	0.0000	0.0000	0.0137	0.0021
ICAM1_E242_F	0.4422	0.0000	0.0127	0.4292	0.0115	0.0106	0.2972	0.2071	0.0000	0.0000	0.0122	0.0118	0.1522	0.1039	0.7810
ICAM1_P119_R	0.0000	0.0000	0.0004	0.0000	0.2731	0.0000	0.0070	0.0000	0.7057	0.0000	0.0062	0.0022	0.0543	0.0087	0.0010
ICAM1_P386_R	0.0000	0.0000	0.0000	0.0000	0.0000	0.0000	0.0071	0.0056	0.0000	0.0000	0.0091	0.0000	0.0000	0.0000	0.0000
ID1_P659_R	0.0000	0.0214	0.0039	0.0000	0.0055	0.0000	0.0000	0.0009	0.0000	0.0000	0.0052	0.0000	0.0000	0.0000	0.0039
IFNG_P459_R	0.0504	0.2812	0.9768	0.9956	0.2994	0.2263	0.4543	0.1935	0.8994	0.9693	0.8120	0.8872	0.9780	0.1675	0.9925
IFNGR1_P307_F	0.1456	0.1945	0.0093	0.1484	0.1235	0.0092	0.0105	0.0339	0.0000	0.0000	0.0108	0.0098	0.0775	0.2029	0.0111

IFNGR2_E164_F	0.0149	0.0000	0.1341	0.0232	0.0338	0.0185	0.1115	0.1295	0.0177	0.0578	0.0292	0.0482	0.0218	0.0128	0.1175
IFNGR2_P377_R	0.0000	0.0000	0.2788	0.9960	0.0000	0.0016	0.0000	0.0000	0.9522	0.0000	0.8809	0.0000	0.1071	0.0000	0.9161
IGF1_E394_F	0.7861	0.0000	0.9348	0.0043	0.0092	0.0000	0.0000	0.9946	0.8985	0.5777	0.6183	0.0083	0.8954	0.7494	0.8906
IGF1_P933_F	0.1896	0.0000	0.2697	0.0000	0.0000	0.0000	0.0051	0.0000	0.5675	0.8463	0.9573	0.0000	0.4407	0.0000	0.9903
IGF1R_E186_R	0.0000	0.0000	0.0000	0.0086	0.0000	0.0000	0.0040	0.0000	0.0000	0.0000	0.0043	0.0000	0.0000	0.0000	0.0014
IGF1R_P325_R	0.1751	0.5281	0.0136	0.3896	0.4506	0.3675	0.5873	0.0108	0.0000	0.0000	0.0168	0.4583	0.0059	0.5986	0.3301
IGF2_E134_R	0.0000	0.0000	0.0000	0.0000	0.0000	0.0070	0.0028	0.0000	0.0000	0.0000	0.0020	0.0000	0.0000	0.0119	0.0000
IGF2_P1036_R	0.0276	0.1133	0.1099	0.0864	0.0079	0.9777	0.0104	0.0104	0.0028	0.5902	0.0152	0.0109	0.0534	0.0144	0.0068
IGF2_P36_R	0.1216	0.0142	0.0151	0.1188	0.7662	0.0694	0.4342	0.0000	0.0000	0.0000	0.1224	0.1421	0.0052	0.4091	0.1073
IGF2AS_P203_F	0.0000	0.0000	0.4285	0.0000	0.0000	0.0000	0.0000	0.0000	0.0000	0.0000	0.0000	0.0000	0.0000	0.0000	0.0000
IGF2R_P396_R	0.9530	0.8949	0.1015	0.3307	0.2930	0.9655	0.6652	0.9387	0.1786	0.2525	0.0951	0.2821	0.2008	0.9896	0.1611
IGFBP1_E48_R	0.0000	0.0000	0.5874	0.0128	0.0089	0.0000	0.0060	0.7978	0.0000	0.0000	0.0087	0.0050	0.0000	0.0227	0.0154
IGFBP1_P12_R	0.0087	0.8639	0.4167	0.4179	0.0093	0.0297	0.0162	0.0031	0.0000	0.0000	0.0134	0.3577	0.0000	0.5744	0.5022
IGFBP2_P306_F	0.0000	0.0044	0.0000	0.0090	0.0072	0.0000	0.0034	0.0071	0.0000	0.0000	0.0000	0.0000	0.0543	0.0036	0.0000
IGFBP3_E65_R	0.5370	0.2199	0.0132	0.5267	0.0192	0.0095	0.0165	0.4702	0.0000	0.0000	0.0128	0.0183	0.0116	0.0148	0.0111
IGFBP3_P1035_F	0.0000	0.0000	0.0000	0.0000	0.0000	0.0000	0.0000	0.0020	0.0000	0.0000	0.0026	0.0000	0.0000	0.0000	0.0000
IGFBP3_P423_R	0.1494	0.0000	0.0102	0.0967	0.0093	0.1129	0.0047	0.0060	0.0000	0.0000	0.0108	0.0042	0.0228	0.0696	0.1274
IGFBP7_P371_F	0.4533	0.0000	0.0045	0.4383	0.0093	0.1433	0.0099	0.0180	0.0000	0.0000	0.0122	0.0047	0.0000	0.0169	0.0102
IGSF4_P454_F	0.0000	0.5123	0.0481	0.0000	0.0074	0.0051	0.0000	0.0000	0.0000	0.0000	0.0069	0.0103	0.0002	0.0090	0.0000
IGSF4_P86_R	0.2460	0.9917	0.0280	0.0099	0.0163	0.1349	0.7013	0.0056	0.0000	0.0000	0.0236	0.0116	0.0122	0.2236	0.2788
IGSF4C_E65_F	0.9956	0.3565	0.8906	0.0000	0.0000	0.9962	0.0000	0.9951	0.9810	0.0000	0.7367	0.0000	0.9285	0.0070	0.7506
IGSF4C_P533_R	0.0425	0.0000	0.0757	0.0000	0.0000	0.0070	0.0000	0.0000	0.0000	0.0000	0.0106	0.0086	0.0015	0.0091	0.0183
IHH_E186_F	0.0000	0.0000	0.0031	0.0000	0.0000	0.0042	0.0060	0.0000	0.0000	0.0000	0.0007	0.0026	0.0000	0.0000	0.0030
IHH_P246_R	0.0000	0.0000	0.3258	0.0000	0.0144	0.0245	0.6264	0.0840	0.0000	0.0000	0.0119	0.3188	0.0812	0.0000	0.0252
IHH_P529_F	0.1077	0.4703	0.0096	0.0121	0.0000	0.0000	0.1193	0.0057	0.0000	0.0000	0.0137	0.0129	0.0014	0.0000	0.0052
IL10_P85_F	0.0000	0.0000	0.2818	0.0000	0.0026	0.0012	0.9889	0.0000	0.0000	0.0000	0.0026	0.5029	0.1791	0.0000	0.0000
IL11_P11_R	0.0000	0.0000	0.0088	0.0709	0.0000	0.0072	0.0000	0.0000	0.0000	0.0000	0.0175	0.0054	0.0000	0.0000	0.0000
IL12A_E287_R	0.0000	0.0000	0.0335	0.0000	0.0000	0.0019	0.0000	0.0000	0.0000	0.0000	0.0059	0.0069	0.0000	0.0000	0.0023
IL12B_P392_R	0.0088	0.0027	0.6663	0.0053	0.0038	0.0042	0.1142	0.0026	0.0000	0.1690	0.0005	0.0015	0.1544	0.0020	0.0019
IL13_E75_R	0.9943	0.6793	0.9741	0.8290	0.7481	0.7561	0.6871	0.7519	0.6028	0.9799	0.7300	0.9854	0.9847	0.9233	0.6794
IL17RB_E164_R	0.0000	0.0000	0.0053	0.0043	0.0000	0.0061	0.0830	0.0000	0.0000	0.0000	0.0000	0.0049	0.1844	0.0000	0.0000
IL17RB_P788_R	0.0063	0.0000	0.0000	0.0000	0.9951	0.0000	0.0000	0.0000	0.7045	0.0000	0.0000	0.0000	0.0000	0.0000	0.0000
IL18BP_P51_R	0.0301	0.2356	0.0264	0.3014	0.2689	0.0230	0.0561	0.0123	0.0121	0.0201	0.0472	0.0476	0.0244	0.3893	0.0497

IL1A_E113_R	0.9943	0.2958	0.9857	0.9956	0.9946	0.0000	0.9934	0.9947	0.9902	0.8592	0.9867	0.6890	0.9856	0.9873	0.9911
IL1RN_E42_F	0.1114	0.0000	0.9802	0.0000	0.9919	0.9801	0.0000	0.9944	0.9808	0.9871	0.9890	0.7553	0.9825	0.0087	0.9887
IL3_P556_F	0.0000	0.5330	0.9762	0.0000	0.0000	0.0000	0.0000	0.0000	0.9827	0.9886	0.9870	0.9883	0.9800	0.2976	0.9932
IL6_E168_F	0.2128	0.4573	0.0088	0.2646	0.0161	0.0068	0.0100	0.0106	0.0000	0.0000	0.4323	0.0000	0.0731	0.9868	0.0070
IL8_E118_R	0.0000	0.0000	0.3152	0.0000	0.9946	0.0000	0.0000	0.0032	0.0000	0.0000	0.0021	0.0000	0.0000	0.0086	0.0000
IL8_P83_F	0.0000	0.0000	0.2475	0.0000	0.0000	0.0000	0.0000	0.0000	0.0000	0.0000	0.0000	0.0000	0.0000	0.0000	0.0000
IMPACT_P186_F	0.0068	0.0000	0.0000	0.0000	0.0000	0.0000	0.0000	0.0000	0.0000	0.0000	0.0000	0.0000	0.0000	0.0000	0.0000
IMPACT_P234_R	0.0000	0.0000	0.1215	0.0000	0.0053	0.0024	0.0059	0.0018	0.0000	0.0000	0.0059	0.0000	0.0000	0.0000	0.0000
INSR_P1063_R	0.0119	0.0000	0.0000	0.0000	0.0000	0.0000	0.0000	0.0000	0.0000	0.0000	0.0000	0.0000	0.1997	0.0000	0.0000
IPF1_P234_F	0.0000	0.0005	0.2158	0.0081	0.0000	0.0000	0.0045	0.0000	0.0000	0.7680	0.0089	0.0018	0.1645	0.0087	0.0051
IRAK1_P455_R	0.0000	0.0000	0.2658	0.0000	0.0861	0.0784	0.0082	0.0578	0.0000	0.0000	0.0101	0.0416	0.4013	0.0000	0.0081
IRAK3_E130_F	0.0088	0.0000	0.0077	0.0000	0.0000	0.0004	0.0058	0.0033	0.0000	0.0000	0.0127	0.0114	0.0033	0.0111	0.0063
IRAK3_P13_F	0.0000	0.0000	0.0067	0.0000	0.0000	0.0114	0.1667	0.0000	0.0000	0.0000	0.0186	0.0058	0.0000	0.0066	0.0035
IRF5_P123_F	0.0140	0.0349	0.0436	0.0070	0.0056	0.0070	0.0000	0.0772	0.2089	0.0232	0.0115	0.0094	0.0173	0.0000	0.9285
IRF7_P277_R	0.0132	0.0692	0.0526	0.0258	0.0279	0.0181	0.2711	0.1964	0.0000	0.0000	0.0151	0.0146	0.0087	0.0304	0.0202
ISL1_E87_R	0.0000	0.0000	0.0094	0.0000	0.0000	0.0049	0.0000	0.0000	0.0000	0.0000	0.0091	0.0000	0.0480	0.0088	0.0046
ISL1_P379_F	0.0526	0.0000	0.0037	0.0114	0.0085	0.0039	0.0038	0.0000	0.0850	0.0000	0.0059	0.0074	0.1974	0.0120	0.0044
ISL1_P554_F	0.0000	0.0000	0.0000	0.0000	0.0069	0.0000	0.0000	0.0002	0.0000	0.0000	0.0000	0.0000	0.0000	0.0000	0.0008
ITGA2_E120_F	0.0127	0.0000	0.0085	0.0084	0.0070	0.0080	0.4295	0.3367	0.0000	0.0000	0.0119	0.2924	0.0008	0.0101	0.0048
ITGA2_P26_R	0.0116	0.0000	0.0000	0.0000	0.0290	0.0035	0.0000	0.0000	0.0000	0.0000	0.0000	0.0000	0.0000	0.0012	0.0021
ITGB4_E144_F	0.0000	0.0000	0.0042	0.0113	0.0000	0.0075	0.0134	0.0000	0.0000	0.0000	0.0102	0.0000	0.0269	0.0075	0.0054
ITGB4_P517_F	0.0000	0.0000	0.0000	0.0026	0.0000	0.0000	0.0038	0.0000	0.0000	0.0000	0.0005	0.0001	0.3014	0.0029	0.0000
ITPR3_E86_R	0.0000	0.0000	0.0000	0.0000	0.0000	0.0000	0.0000	0.0000	0.0000	0.0000	0.0000	0.0025	0.0000	0.0006	0.0057
ITPR3_P1112_F	0.0000	0.0000	0.0000	0.0000	0.0000	0.0014	0.0000	0.0000	0.0000	0.0000	0.0010	0.0021	0.0000	0.0000	0.0000
JAG2_E54_F	0.0091	0.0000	0.0053	0.0000	0.0000	0.0000	0.0064	0.0000	0.5595	0.0000	0.0075	0.0130	0.0000	0.0030	0.0000
JAG2_P264_F	0.4793	0.0000	0.0152	0.6053	0.0046	0.0061	0.0083	0.0087	0.0000	0.0000	0.0197	0.1739	0.0060	0.0095	0.0063
JAK2_P772_R	0.4446	0.0000	0.0108	0.0233	0.2117	0.1101	0.0155	0.2149	0.0000	0.0000	0.0137	0.0087	0.0014	0.1766	0.0290
JAK3_E64_F	0.0000	0.0000	0.0089	0.2407	0.0067	0.0020	0.0000	0.0000	0.0000	0.0000	0.0099	0.0061	0.0055	0.0053	0.0030
JAK3_P156_R	0.0099	0.0000	0.0180	0.1164	0.1323	0.0061	0.0122	0.1188	0.0000	0.0000	0.0147	0.0081	0.1164	0.0633	0.0103
JUNB_P1149_R	0.5531	0.4492	0.0531	0.3416	0.1311	0.5820	0.5649	0.4774	0.0687	0.1743	0.0839	0.4721	0.0564	0.1763	0.5172
KCNK4_E3_F	0.0076	0.0000	0.1244	0.0054	0.0042	0.0003	0.0000	0.0000	0.0000	0.5565	0.0211	0.0000	0.1412	0.0000	0.0023
KDR_E79_F	0.7582	0.5589	0.0414	0.9074	0.7142	0.0541	0.7483	0.0315	0.0000	0.0209	0.0150	0.0352	0.1035	0.0907	0.0387
KDR_P445_R	0.0000	0.0000	0.0069	0.0000	0.0000	0.0188	0.0000	0.0000	0.0000	0.0000	0.2689	0.0037	0.0693	0.0053	0.0000

KIAA1804_P689_R	0.0096	0.0000	0.0116	0.0171	0.0082	0.0707	0.0089	0.0040	0.0000	0.0000	0.0149	0.0060	0.0406	0.0081	0.0060
KIT_P367_R	0.0000	0.0000	0.0058	0.0000	0.3018	0.0000	0.0060	0.0000	0.0000	0.0000	0.0078	0.0000	0.0000	0.0015	0.0041
KIT_P405_F	0.0095	0.0000	0.0059	0.0000	0.0000	0.0060	0.4401	0.0065	0.2565	0.0000	0.0101	0.0055	0.0915	0.0089	0.0046
KLF5_E190_R	0.0000	0.0000	0.1599	0.0000	0.0000	0.0022	0.0000	0.0000	0.0000	0.0000	0.0046	0.0052	0.0000	0.0751	0.0031
KLK11_P103_R	0.3732	0.0000	0.9670	0.0000	0.0052	0.9938	0.9947	0.9921	0.7108	0.9837	0.8976	0.9869	0.9009	0.0000	0.9789
KLK11_P1290_F	0.0000	0.0550	0.9787	0.0000	0.0000	0.0000	0.0000	0.0000	0.9823	0.9448	0.9851	0.0447	0.9522	0.2944	0.9832
KRAS_E82_F	0.0000	0.0000	0.7624	0.0080	0.0070	0.0000	0.1834	0.0047	0.0000	0.8556	0.1783	0.0437	0.6245	0.0084	0.0031
KRAS_P651_F	0.4929	0.2206	0.0824	0.5853	0.5374	0.4671	0.5573	0.4664	0.0820	0.1375	0.0744	0.4359	0.0691	0.4337	0.4749
KRT13_P341_R	0.0000	0.0000	0.8836	0.0000	0.0000	0.9941	0.0000	0.0000	0.8720	0.9855	0.6723	0.0000	0.9815	0.4206	0.0000
KRT5_P308_F	0.2740	0.6163	0.7516	0.1906	0.1091	0.5589	0.2044	0.2110	0.9513	0.1642	0.8721	0.8994	0.9388	0.1982	0.9281
L1CAM_P148_R	0.0000	0.0000	0.0476	0.0084	0.0061	0.0058	0.0000	0.0719	0.0000	0.0000	0.0086	0.0100	0.0000	0.0000	0.0045
L1CAM_P19_F	0.9942	0.0000	0.2684	0.0000	0.0080	0.0000	0.0000	0.0647	0.0000	0.7507	0.0139	0.0000	0.1934	0.0000	0.0035
LAMC1_E466_R	0.1265	0.0000	0.0039	0.0748	0.0073	0.0487	0.0000	0.1486	0.0000	0.0000	0.0242	0.0045	0.0000	0.0179	0.0026
LIF_E208_F	0.0175	0.0000	0.0134	0.0121	0.0210	0.0053	0.0000	0.0035	0.0000	0.0000	0.0153	0.0111	0.0023	0.0144	0.2181
LIF_P383_R	0.0000	0.0000	0.0857	0.0000	0.3694	0.0000	0.0000	0.0039	0.8843	0.0000	0.1416	0.1811	0.3829	0.1105	0.0000
LIG4_P194_F	0.2800	0.0000	0.0126	0.0248	0.2677	0.0074	0.0000	0.0190	0.0000	0.0000	0.0100	0.0097	0.0025	0.0000	0.1777
LMO1_E265_R	0.0000	0.0000	0.0000	0.9847	0.0041	0.0000	0.0044	0.0000	0.0000	0.0000	0.0019	0.0000	0.1281	0.0016	0.0000
LMO1_P169_F	0.0000	0.0000	0.0073	0.0000	0.0053	0.0013	0.0074	0.0041	0.0000	0.0000	0.0096	0.0000	0.0016	0.0000	0.0057
LOX_P313_R	0.1021	0.1685	0.0163	0.0167	0.1278	0.0129	0.0209	0.0093	0.0000	0.0000	0.0407	0.8734	0.0099	0.0308	0.0804
LOX_P71_F	0.0000	0.0000	0.0009	0.0068	0.0000	0.0038	0.0000	0.0321	0.0000	0.0000	0.0044	0.0031	0.0000	0.0081	0.0000
LRP2_E20_F	0.5492	0.9519	0.0267	0.6014	0.0463	0.5035	0.5174	0.5517	0.1447	0.0461	0.0233	0.0301	0.0261	0.8627	0.4658
LRRC32_P865_R	0.0000	0.0000	0.0046	0.0000	0.0000	0.0000	0.0041	0.0000	0.0000	0.0000	0.0198	0.0064	0.0000	0.0000	0.0042
LTB4R_E64_R	0.0554	0.5275	0.1611	0.9047	0.2608	0.4585	0.6125	0.0164	0.0000	0.0000	0.0390	0.0697	0.2062	0.8248	0.0321
LYN_E353_F	0.0000	0.0000	0.7264	0.0000	0.0000	0.0000	0.0043	0.0000	0.0000	0.0000	0.0000	0.0000	0.0000	0.0000	0.0028
LYN_P241_F	0.3095	0.1022	0.0258	0.4185	0.0557	0.0708	0.0626	0.2563	0.0466	0.0361	0.2542	0.0519	0.0383	0.0437	0.0380
MAF_P826_R	0.0060	0.0000	0.0000	0.0000	0.0033	0.0000	0.0000	0.0022	0.0000	0.0000	0.0000	0.0000	0.0000	0.0000	0.0000
MAGEC3_P903_F	0.0000	0.0000	0.3335	0.0000	0.0000	0.0000	0.0105	0.0074	0.0000	0.7008	0.0000	0.0000	0.9128	0.9892	0.9904
MAGEL2_E166_R	0.0000	0.0000	0.5368	0.0000	0.0149	0.0000	0.9934	0.0000	0.0000	0.0000	0.0107	0.0000	0.9291	0.0090	0.0000
MALT1_P406_R	0.0888	0.0000	0.1812	0.0000	0.0000	0.0123	0.0000	0.0000	0.0000	0.0000	0.6508	0.0000	0.0000	0.0139	0.0032
MAP2K6_E297_F	0.0000	0.0000	0.0000	0.0000	0.0028	0.0000	0.0000	0.0000	0.0000	0.0000	0.0022	0.0000	0.1057	0.0000	0.0000
MAP2K6_P297_R	0.0157	0.0000	0.1027	0.0161	0.1261	0.0170	0.1161	0.1367	0.0019	0.2832	0.0424	0.0127	0.1140	0.0154	0.0157
MAP3K1_P7_F	0.4375	0.0000	0.0333	0.8300	0.0601	0.0063	0.1209	0.1691	0.0232	0.1051	0.0227	0.4741	0.0192	0.7592	0.0128
MAP3K9_E17_R	0.5152	0.0000	0.0245	0.0000	0.0392	0.0283	0.0000	0.4380	0.0000	0.0000	0.0144	0.0065	0.0071	0.0000	0.4433

MAPK12_E165_R	0.0000	0.0000	0.0080	0.0000	0.0078	0.0000	0.0064	0.0072	0.0000	0.0000	0.0170	0.6402	0.0029	0.0091	0.1562
MAPK12_P416_F	0.0000	0.0057	0.0000	0.0000	0.0000	0.0000	0.0000	0.0000	0.0000	0.0000	0.0077	0.0000	0.0000	0.0000	0.0000
MAPK14_P327_R	0.0121	0.8689	0.0048	0.1672	0.0000	0.3887	0.0000	0.0000	0.0000	0.0000	0.0060	0.8291	0.0179	0.9621	0.3801
MAPK4_E273_R	0.1836	0.0000	0.9416	0.0000	0.0000	0.0000	0.0050	0.0027	0.9507	0.0000	0.8846	0.0000	0.8845	0.0018	0.6726
MAPK9_P1175_F	0.0067	0.1352	0.9735	0.0060	0.0000	0.9946	0.0000	0.0000	0.7425	0.0000	0.9826	0.8064	0.9749	0.0000	0.0000
MAS1_P469_R	0.1814	0.1653	0.9741	0.1962	0.3785	0.4413	0.2439	0.0214	0.9838	0.8757	0.9809	0.2126	0.9757	0.7941	0.9908
MATK_P64_F	0.0918	0.0021	0.0070	0.0099	0.0072	0.0146	0.0084	0.0933	0.0000	0.0000	0.0094	0.0665	0.0442	0.0156	0.0064
MC2R_P1025_F	0.0000	0.0000	0.0000	0.0000	0.0000	0.0000	0.0000	0.0000	0.6513	0.9695	0.0000	0.0000	0.4538	0.0000	0.0000
MCAM_P169_R	0.0000	0.0000	0.0077	0.0090	0.0000	0.1698	0.0000	0.0043	0.0000	0.0000	0.0063	0.0027	0.0005	0.1201	0.0151
MCAM_P265_R	0.0981	0.2433	0.4253	0.9945	0.1018	0.0079	0.0077	0.0898	0.0091	0.0168	0.0266	0.0150	0.1938	0.0111	0.0078
MCC_P196_R	0.0000	0.0000	0.0000	0.0024	0.0015	0.0000	0.0033	0.0029	0.0000	0.0000	0.9687	0.0000	0.0000	0.0040	0.0000
MCM2_P241_R	0.0148	0.1042	0.0066	0.3952	0.0117	0.0111	0.0143	0.0074	0.0000	0.0000	0.0078	0.2356	0.0896	0.0146	0.0076
MDR1_seq_42_S300_R	0.0138	0.0341	0.0031	0.0012	0.0368	0.0550	0.0132	0.0085	0.0000	0.0000	0.0088	0.0580	0.0000	0.0067	0.0149
MDS1_E45_F	0.0110	0.0000	0.0157	0.0000	0.0000	0.0000	0.0000	0.0067	0.0000	0.0000	0.0156	0.0081	0.0086	0.0118	0.0000
MECP2_E90_R	0.0000	0.0000	0.3045	0.0100	0.0000	0.9949	0.0070	0.0000	0.0000	0.0000	0.2563	0.2995	0.2196	0.0099	0.0049
MEG3_E91_F	0.0000	0.0000	0.1467	0.0000	0.0000	0.0035	0.0000	0.0000	0.0000	0.5456	0.0045	0.0000	0.1905	0.0085	0.0000
MEST_E150_F	0.0056	0.0000	0.0879	0.0000	0.0002	0.0000	0.0000	0.0011	0.0000	0.0000	0.0037	0.0017	0.0698	0.0041	0.0009
MEST_P4_F	0.1169	0.0000	0.0026	0.0000	0.0000	0.0000	0.0000	0.0000	0.0000	0.0000	0.0033	0.0018	0.2345	0.9270	0.0000
MEST_P62_R	0.0082	0.0000	0.0515	0.0000	0.0034	0.0000	0.0000	0.0000	0.0000	0.0000	0.0010	0.0000	0.0485	0.0032	0.0000
MET_E333_F	0.0617	0.4040	0.0271	0.0184	0.0148	0.2324	0.2697	0.0092	0.0000	0.7868	0.0636	0.2186	0.7563	0.0336	0.0000
MFAP4_P197_F	0.0121	0.0000	0.1083	0.0000	0.0072	0.0000	0.0000	0.0000	0.0000	0.0000	0.0063	0.5142	0.0000	0.0158	0.0005
MGMT_P272_R	0.0000	0.0000	0.0024	0.0000	0.0050	0.0054	0.0055	0.0000	0.0000	0.0000	0.0056	0.0015	0.0050	0.0051	0.0047
MGMT_P281_F	0.6336	0.8767	0.0149	0.6520	0.5877	0.4695	0.0629	0.5265	0.0000	0.0000	0.0174	0.0189	0.0095	0.0250	0.4010
MKRN3_P108_F	0.1763	0.4610	0.8313	0.0000	0.0000	0.9873	0.0000	0.0000	0.9697	0.9665	0.9564	0.0000	0.9720	0.0000	0.0000
MLF1_P97_F	0.0000	0.0163	0.0064	0.0085	0.0000	0.0054	0.0115	0.0000	0.0000	0.0000	0.0100	0.0057	0.0000	0.0367	0.0754
MLH1_P381_F	0.0089	0.0000	0.0077	0.3457	0.0146	0.0130	0.4378	0.0000	0.0000	0.0000	0.0122	0.2732	0.0006	0.0000	0.0055
MLH3_E72_F	0.0196	0.0000	0.0036	0.0930	0.0000	0.0080	0.0000	0.0000	0.0000	0.0000	0.0061	0.0000	0.0000	0.0000	0.0053
MLH3_P25_F	0.2266	0.0000	0.1022	0.0069	0.0042	0.0000	0.0101	0.0047	0.0000	0.0000	0.0063	0.0063	0.0746	0.0079	0.0040
MLLT3_E93_R	0.0000	0.0000	0.0033	0.0000	0.0000	0.0057	0.0000	0.0000	0.0000	0.0000	0.0079	0.0018	0.0000	0.0000	0.0000
MLLT4_P1400_F	0.0236	0.8665	0.0159	0.1393	0.1911	0.0241	0.1859	0.2100	0.0030	0.0000	0.0158	0.0147	0.0167	0.0208	0.0209
MLLT6_P957_F	0.0000	0.0000	0.0015	0.0106	0.0062	0.0000	0.0000	0.0000	0.0000	0.0000	0.0040	0.7613	0.0000	0.7884	0.0032
MME_E29_F	0.0000	0.0000	0.0010	0.0000	0.0000	0.0082	0.0010	0.0000	0.0000	0.0000	0.0082	0.0052	0.0000	0.0057	0.0067
MME_P388_F	0.0000	0.6641	0.0000	0.0000	0.0183	0.0000	0.0000	0.0000	0.0000	0.0000	0.2528	0.0000	0.0868	0.0000	0.0000

MMP19_E274_R	0.4531	0.1976	0.9805	0.0101	0.9329	0.9960	0.9936	0.0000	0.9833	0.9916	0.9741	0.8758	0.9394	0.9936	0.0000
MMP2_E21_R	0.0000	0.0000	0.0011	0.0000	0.0000	0.0000	0.0035	0.0000	0.0000	0.7220	0.0073	0.0052	0.0000	0.0033	0.9921
MMP2_P197_F	0.0000	0.0665	0.0057	0.0000	0.0090	0.0000	0.0094	0.0057	0.0000	0.0000	0.0150	0.0000	0.0006	0.0145	0.0021
MMP2_P303_R	0.4050	0.0000	0.0104	0.0065	0.1599	0.0070	0.0513	0.0036	0.0000	0.0000	0.3015	0.0058	0.0040	0.0067	0.0020
MMP3_P16_R	0.9904	0.4500	0.9779	0.0000	0.0000	0.0000	0.0000	0.0011	0.5354	0.4558	0.9851	0.9851	0.9734	0.9909	0.0000
MMP7_P613_F	0.2200	0.0793	0.8444	0.2680	0.0323	0.0847	0.2749	0.9613	0.7923	0.0417	0.4495	0.1096	0.8657	0.6920	0.0993
MMP9_P189_F	0.0000	0.0000	0.0552	0.0000	0.0000	0.0032	0.1568	0.0000	0.0000	0.0000	0.4879	0.0075	0.3138	0.0161	0.0000
MMP9_P237_R	0.2716	0.0000	0.0113	0.0103	0.0082	0.1877	0.0104	0.0034	0.0000	0.0000	0.0123	0.0085	0.0029	0.0133	0.1695
MOS_E60_R	0.0113	0.0237	0.0039	0.0690	0.0128	0.0089	0.0074	0.0144	0.0000	0.0000	0.0088	0.0062	0.0000	0.0176	0.0306
MPO_P883_R	0.0000	0.0000	0.0858	0.0335	0.0000	0.0000	0.0000	0.0000	0.0000	0.0000	0.0137	0.0022	0.0707	0.0059	0.0044
MSH3_E3_F	0.0000	0.0000	0.9178	0.9912	0.9935	0.9872	0.0000	0.0000	0.9803	0.6986	0.9329	0.9294	0.9815	0.0295	0.9897
MSH3_P13_R	0.9963	0.2950	0.7773	0.5648	0.0660	0.0113	0.0156	0.8337	0.8831	0.7784	0.7756	0.8305	0.5999	0.0237	0.0193
MST1R_P392_F	0.0095	0.0000	0.1099	0.0000	0.0000	0.0000	0.0067	0.0000	0.0000	0.0000	0.0005	0.0000	0.0000	0.0019	0.0000
MT1A_E13_R	0.0134	0.3637	0.0073	0.0486	0.2250	0.0018	0.0000	0.0059	0.0000	0.0000	0.3244	0.0382	0.0425	0.0000	0.0088
MT1A_P49_R	0.0000	0.0000	0.0000	0.0000	0.0000	0.0000	0.0000	0.0000	0.0000	0.0000	0.0000	0.0018	0.0000	0.0000	0.0000
MTA1_P478_F	0.1374	0.0000	0.0141	0.0862	0.0090	0.0788	0.1189	0.1644	0.0000	0.0000	0.0272	0.0113	0.0060	0.1485	0.0945
MUC1_E18_R	0.0000	0.0000	0.0000	0.0000	0.3200	0.0000	0.0000	0.0000	0.0000	0.0000	0.0043	0.0000	0.6206	0.0000	0.0132
MUC1_P191_F	0.0000	0.0000	0.1274	0.0000	0.0000	0.0000	0.0000	0.0000	0.9773	0.0000	0.0000	0.0073	0.1450	0.0000	0.0000
MXI1_P75_R	0.0095	0.0055	0.0216	0.0168	0.0064	0.0070	0.0057	0.0055	0.0000	0.0000	0.0064	0.0053	0.1015	0.0137	0.0064
MYBL2_P211_F	0.3245	0.0000	0.0108	0.0000	0.0000	0.0013	0.3114	0.0329	0.0000	0.0000	0.0100	0.0000	0.0020	0.0020	0.0078
MYCL1_P502_R	0.0144	0.0000	0.0029	0.0000	0.0000	0.0000	0.0000	0.0000	0.0000	0.0000	0.0049	0.0040	0.0000	0.0144	0.0076
MYCN_E77_R	0.0000	0.0000	0.0444	0.0000	0.0000	0.0165	0.0582	0.0000	0.0000	0.0000	0.0179	0.0074	0.0100	0.0198	0.0068
MYCN_P464_R	0.0000	0.0000	0.0048	0.0000	0.0099	0.0012	0.0000	0.0068	0.0000	0.0000	0.0167	0.0033	0.0000	0.0071	0.0060
MYH11_P22_F	0.2154	0.1162	0.0080	0.0195	0.0000	0.0045	0.0077	0.0000	0.0000	0.0000	0.0121	0.0063	0.1054	0.2890	0.0037
MYH11_P236_R	0.0000	0.0000	0.0000	0.0000	0.0000	0.0000	0.0000	0.0000	0.0000	0.0000	0.0000	0.0000	0.0000	0.0000	0.0000
MYLK_P469_R	0.3479	0.6332	0.2788	0.5497	0.0112	0.1204	0.3714	0.2557	0.1838	0.0000	0.0106	0.0154	0.1004	0.0282	0.0117
MYOD1_E156_F	0.0056	0.4422	0.0022	0.0144	0.0037	0.0041	0.0726	0.0000	0.0000	0.3587	0.0050	0.0043	0.0174	0.2976	0.0000
MYOD1_P50_F	0.0000	0.0000	0.1907	0.0000	0.0000	0.0053	0.0000	0.0117	0.6825	0.0000	0.0026	0.0000	0.0000	0.0000	0.0000
NBL1_E205_R	0.8840	0.9858	0.9808	0.9500	0.9196	0.1032	0.9265	0.9922	0.8594	0.9185	0.9870	0.9282	0.9430	0.7779	0.8665
NBL1_P24_F	0.1710	0.0000	0.8587	0.0000	0.1534	0.7925	0.0000	0.9880	0.9669	0.2655	0.1042	0.0000	0.9611	0.0000	0.9863
NCL_P840_R	0.0107	0.0000	0.0087	0.0070	0.0000	0.0069	0.0000	0.0000	0.0000	0.0000	0.0137	0.0238	0.0038	0.0000	0.0042
NDN_P1110_F	0.1104	0.6230	0.9738	0.9942	0.0000	0.0000	0.0000	0.6878	0.9827	0.9797	0.8116	0.0000	0.9696	0.9048	0.9888
NEFL_E23_R	0.7280	0.9995	0.1338	0.9981	0.9845	0.9466	0.9435	0.9949	0.7069	0.8216	0.7792	0.9429	0.9278	0.9981	0.5703

NEFL_P209_R	0.0085	0.0000	0.0000	0.0040	0.0020	0.0000	0.0000	0.0000	0.0000	0.0000	0.0000	0.0046	0.1171	0.0000	0.0002
NEO1_P1067_F	0.0000	0.0000	0.2494	0.0074	0.4649	0.0000	0.0000	0.0000	0.0000	0.0000	0.0000	0.0000	0.0000	0.0050	0.0000
NES_P239_R	0.0093	0.0000	0.2184	0.0108	0.0070	0.3631	0.0239	0.0065	0.0000	0.0000	0.0088	0.0138	0.1896	0.0148	0.0168
NEU1_P745_F	0.0000	0.0000	0.0033	0.0000	0.0000	0.0055	0.0000	0.0000	0.0000	0.0000	0.0059	0.0038	0.1505	0.0135	0.0034
NFKB1_P336_R	0.1191	0.4113	0.0119	0.0209	0.0000	0.0000	0.0000	0.2737	0.7273	0.0000	0.0151	0.0000	0.0042	0.1317	0.2732
NFKB2_P709_R	0.1913	0.1736	0.0276	0.0096	0.2728	0.0081	0.2232	0.0460	0.0012	0.0000	0.5383	0.0875	0.0138	0.4464	0.0181
NGFB_E353_F	0.0000	0.0000	0.1253	0.0000	0.0050	0.0000	0.0000	0.0020	0.0000	0.0000	0.0028	0.0037	0.0000	0.0112	0.0000
NGFB_P13_F	0.3304	0.0000	0.1887	0.0140	0.0171	0.3154	0.9114	0.3346	0.1230	0.3479	0.1189	0.0164	0.1626	0.9920	0.4456
NGFR_E328_F	0.0000	0.0000	0.1497	0.0000	0.0000	0.0650	0.0000	0.0040	0.0000	0.0000	0.0066	0.0060	0.0000	0.0026	0.0035
NGFR_P355_F	0.2881	0.1314	0.0538	0.1237	0.2206	0.3429	0.4086	0.2957	0.0565	0.1098	0.1469	0.0876	0.2244	0.1711	0.0620
NKX3-1_P146_F	0.0291	0.0000	0.1043	0.0000	0.0000	0.0000	0.0000	0.1030	0.0000	0.0000	0.0101	0.0000	0.0000	0.0000	0.0040
NKX3-1_P871_R	0.0000	0.6091	0.9803	0.0000	0.9829	0.9931	0.9935	0.0000	0.9865	0.9009	0.9855	0.9881	0.9826	0.9909	0.9901
NOTCH1_E452_R	0.0000	0.0000	0.0000	0.0000	0.0000	0.0000	0.0000	0.0000	0.0000	0.0000	0.0000	0.0000	0.0000	0.0000	0.0000
NOTCH1_P1198_F	0.0000	0.0000	0.1524	0.0000	0.0000	0.0000	0.0052	0.0000	0.0000	0.0000	0.0066	0.0000	0.0713	0.0000	0.0000
NOTCH2_P312_R	0.0127	0.1243	0.0017	0.3119	0.0091	0.0062	0.0048	0.0038	0.0000	0.0000	0.0054	0.0050	0.0000	0.0154	0.0042
NOTCH3_E403_F	0.0000	0.0000	0.0000	0.0000	0.0046	0.0009	0.0000	0.0006	0.9756	0.0000	0.0009	0.0000	0.0000	0.0000	0.0000
NPR2_P618_F	0.0000	0.0000	0.2926	0.0000	0.0000	0.9851	0.0074	0.0000	0.7013	0.0000	0.3875	0.4823	0.1381	0.0000	0.0000
NPY_P295_F	0.0538	0.2499	0.0182	0.0431	0.0398	0.0397	0.5783	0.4710	0.0000	0.0000	0.0287	0.0588	0.0218	0.0554	0.5088
NPY_P91_F	0.0113	0.0000	0.0162	0.0367	0.0108	0.0161	0.0878	0.0104	0.0000	0.0000	0.0176	0.0188	0.0132	0.0533	0.0123
NQO1_E74_R	0.0000	0.0000	0.0062	0.0000	0.0000	0.0000	0.0066	0.0000	0.0000	0.0000	0.4389	0.0000	0.0517	0.0117	0.0056
NQO1_P345_R	0.0083	0.0000	0.0012	0.0000	0.0017	0.0000	0.0000	0.0000	0.0000	0.0000	0.0046	0.0000	0.0000	0.4037	0.0018
NR2F6_E375_R	0.0567	0.0000	0.0450	0.0153	0.0106	0.0102	0.0000	0.0000	0.0000	0.0000	0.0152	0.1730	0.0039	0.0100	0.0221
NRAS_P103_R	0.0126	0.2201	0.0000	0.0094	0.0000	0.0003	0.0085	0.0000	0.0000	0.0000	0.0000	0.0000	0.0000	0.0000	0.0022
NRAS_P12_R	0.0041	0.0000	0.0000	0.0000	0.0000	0.0000	0.0000	0.0000	0.0000	0.0000	0.0000	0.0000	0.0000	0.0000	0.0000
NRG1_E74_F	0.0000	0.0000	0.1463	0.0094	0.0000	0.0013	0.0000	0.0000	0.7536	0.0000	0.0000	0.0000	0.1181	0.0000	0.0000
NRG1_P558_R	0.0095	0.0300	0.1359	0.9964	0.0058	0.0724	0.0070	0.0346	0.0000	0.0000	0.0070	0.0054	0.0000	0.0100	0.0759
NTRK2_P10_F	0.0000	0.0000	0.0072	0.0000	0.0000	0.0902	0.1327	0.0932	0.0000	0.0000	0.0054	0.0000	0.0000	0.0000	0.0024
NTRK2_P395_R	0.0040	0.0000	0.0000	0.0000	0.0000	0.0002	0.0021	0.0010	0.0000	0.0000	0.0009	0.0000	0.0000	0.0000	0.0000
NTRK3_E131_F	0.0000	0.0000	0.1889	0.0000	0.0000	0.0000	0.0120	0.0043	0.0000	0.0000	0.0031	0.0055	0.0000	0.0000	0.0000
NTRK3_P636_R	0.0000	0.0000	0.0000	0.0047	0.0000	0.0011	0.0000	0.0000	0.0000	0.0000	0.0000	0.0003	0.0000	0.0000	0.0000
NTRK3_P752_F	0.0000	0.0000	0.0000	0.0000	0.0000	0.0020	0.0064	0.0000	0.0000	0.0000	0.0001	0.0025	0.1712	0.0064	0.0000
NTSR1_E109_F	0.0106	0.1272	0.0059	0.0098	0.0000	0.0078	0.0121	0.0000	0.0000	0.0000	0.0101	0.0089	0.1001	0.0090	0.0056
NTSR1_P318_F	0.1181	0.0000	0.0131	0.0000	0.0075	0.0101	0.0967	0.0000	0.0000	0.0000	0.0390	0.8661	0.0130	0.6903	0.0075

OAT_P465_F	0.2630	0.1501	0.1596	0.5420	0.2423	0.0176	0.1097	0.3039	0.2001	0.0889	0.2627	0.0137	0.1065	0.0177	0.0099
ODC1_P424_F	0.0073	0.0000	0.0000	0.0080	0.0000	0.0018	0.0000	0.0000	0.0000	0.0000	0.0016	0.0000	0.0000	0.0059	0.0000
ONECUT2_E96_F	0.0084	0.0000	0.0242	0.6858	0.3544	0.0524	0.0000	0.0000	0.0000	0.0000	0.0547	0.0071	0.0100	0.0142	0.0408
ONECUT2_P315_R	0.0000	0.0000	0.0000	0.0000	0.0106	0.0000	0.0000	0.0000	0.0000	0.0000	0.0000	0.0039	0.0000	0.0002	0.0000
OPCML_E219_R	0.0108	0.0000	0.0106	0.0083	0.0000	0.0000	0.0077	0.0031	0.0000	0.0000	0.0072	0.0074	0.0000	0.0162	0.0057
OPCML_P71_F	0.0088	0.0000	0.0039	0.0078	0.0005	0.0000	0.0055	0.0000	0.4518	0.0000	0.0102	0.0000	0.0000	0.0000	0.0032
OSM_P188_F	0.1080	0.0290	0.0127	0.0633	0.0228	0.0559	0.0517	0.0880	0.0000	0.5764	0.3491	0.0082	0.0060	0.0075	0.0092
p16_seq_47_S188_R	0.0276	0.0000	0.0049	0.0891	0.0366	0.0710	0.1178	0.0421	0.0000	0.0000	0.0075	0.0065	0.0000	0.0132	0.0539
P2RX7_E323_R	0.0070	0.0000	0.0033	0.0000	0.0030	0.1358	0.3315	0.0000	0.0000	0.0000	0.0050	0.0058	0.0000	0.0000	0.0088
P2RX7_P119_R	0.2254	0.0000	0.2674	0.1091	0.1626	0.1835	0.0130	0.1801	0.0502	0.1629	0.0288	0.0153	0.3300	0.1142	0.2087
P2RX7_P597_F	0.2812	0.9257	0.9824	0.2005	0.3799	0.2003	0.2633	0.0124	0.9892	0.9923	0.9400	0.8335	0.9821	0.5177	0.9920
PALM2-AKAP2_P183_R	0.5115	0.2808	0.0205	0.6747	0.0481	0.0333	0.5972	0.5103	0.0190	0.0396	0.0241	0.0202	0.0928	0.0795	0.0287
PALM2-AKAP2_P420_R	0.0000	0.0000	0.0053	0.9836	0.0000	0.0029	0.0131	0.0066	0.0000	0.0000	0.0010	0.0157	0.0471	0.0155	0.0007
PARP1_P610_R	0.0000	0.0000	0.8643	0.0000	0.0132	0.9943	0.0000	0.9930	0.7622	0.9565	0.9855	0.5582	0.8931	0.0000	0.9908
PAX6_P1121_F	0.0135	0.0000	0.0094	0.0172	0.0000	0.0104	0.0226	0.0058	0.0000	0.0000	0.0098	0.0101	0.0000	0.0000	0.0000
PAX6_P50_R	0.0000	0.0000	0.0000	0.0075	0.0000	0.0000	0.0000	0.0000	0.0000	0.0000	0.0042	0.0000	0.0000	0.0000	0.0000
PCDH1_E22_F	0.0000	0.0000	0.0130	0.0084	0.0000	0.0116	0.0000	0.0576	0.0000	0.0000	0.0143	0.0066	0.0047	0.0104	0.2505
PCGF4_P760_R	0.0981	0.0000	0.0100	0.0155	0.0799	0.0075	0.0101	0.0810	0.0000	0.0000	0.0144	0.0033	0.0907	0.0731	0.0086
PCGF4_P92_R	0.0484	0.0097	0.0101	0.0098	0.0214	0.0074	0.0094	0.0331	0.0000	0.0000	0.0219	0.0442	0.0000	0.0107	0.0112
PCTK1_E77_R	0.9976	0.9939	0.6937	0.9986	0.9587	0.9346	0.9982	0.9969	0.8526	0.9903	0.8645	0.9313	0.7954	0.9984	0.9967
PDE1B_E141_F	0.0147	0.1847	0.0109	0.0159	0.0301	0.0236	0.4547	0.0204	0.0000	0.0000	0.0189	0.7209	0.0074	0.0187	0.0135
PDE1B_P263_R	0.0000	0.0000	0.0686	0.0000	0.0079	0.0041	0.0116	0.0051	0.0953	0.0000	0.0110	0.0000	0.0039	0.0134	0.0000
PDGFA_P841_R	0.0000	0.0000	0.0001	0.0000	0.0038	0.0007	0.0000	0.0000	0.0000	0.0000	0.0030	0.0000	0.0000	0.0000	0.0000
PDGFRB_E195_R	0.0133	0.0000	0.2095	0.0000	0.0081	0.0065	0.0086	0.0000	0.0000	0.0000	0.0134	0.0170	0.0702	0.0169	0.0000
PDGFRB_P343_F	0.0000	0.0000	0.0000	0.0064	0.0000	0.0017	0.0078	0.0000	0.0000	0.0000	0.0038	0.6834	0.0000	0.0077	0.0000
PEG10_P978_R	0.0000	0.0000	0.0415	0.0000	0.9113	0.8087	0.0091	0.0000	0.0000	0.0000	0.0705	0.0041	0.0497	0.0000	0.0529
PENK_E26_F	0.0600	0.1190	0.0838	0.0417	0.0420	0.0586	0.0370	0.0203	0.0183	0.0511	0.3880	0.0211	0.1237	0.1503	0.0295
PENK_P447_R	0.0096	0.7843	0.0044	0.1694	0.4916	0.0000	0.0945	0.0460	0.0000	0.0000	0.0089	0.0090	0.0001	0.0247	0.1693
PGF_E33_F	0.0125	0.0000	0.0132	0.0075	0.0000	0.0170	0.0077	0.0369	0.0000	0.0000	0.0164	0.0186	0.0037	0.0134	0.0825
PHLDA2_P622_F	0.0000	0.0000	0.1640	0.0092	0.0085	0.6581	0.0000	0.0000	0.7012	0.0000	0.0051	0.0037	0.1617	0.0000	0.0000
PI3_P1394_R	0.3699	0.7150	0.9690	0.0246	0.3246	0.2706	0.9936	0.9894	0.3748	0.0000	0.3136	0.4475	0.6206	0.6024	0.3442
PITX2_E24_R	0.0121	0.0000	0.0126	0.3446	0.0595	0.0071	0.1456	0.0084	0.0000	0.0000	0.0176	0.0079	0.0041	0.0107	0.3826
PITX2_P183_R	0.0000	0.0000	0.0000	0.0000	0.0000	0.0000	0.0000	0.0012	0.0000	0.0000	0.0000	0.0021	0.2858	0.0000	0.0000

PKD2_P287_R	0.0093	0.0000	0.0916	0.0118	0.0092	0.1127	0.1722	0.0078	0.0000	0.0000	0.0122	0.0091	0.0000	0.0178	0.2954
PKD2_P336_R	0.0082	0.0000	0.0458	0.0065	0.0095	0.0058	0.2916	0.0053	0.0021	0.0000	0.0126	0.0092	0.1111	0.3795	0.0066
PLAU_P11_F	0.3430	0.0000	0.1152	0.2875	0.0155	0.0241	0.3018	0.3425	0.0007	0.0000	0.0238	0.0185	0.0156	0.0174	0.0183
PLAU_P176_R	0.0000	0.0000	0.0005	0.0000	0.0000	0.0009	0.0000	0.0000	0.0000	0.0000	0.0039	0.0126	0.2778	0.0048	0.0000
PLAUR_E123_F	0.0000	0.0000	0.0022	0.0000	0.0054	0.0000	0.0000	0.0015	0.1558	0.0000	0.0034	0.0073	0.0000	0.0000	0.0051
PLAUR_P82_F	0.0000	0.1023	0.0000	0.1688	0.0098	0.0059	0.0060	0.0003	0.0000	0.0000	0.0024	0.0000	0.0000	0.0134	0.0051
PLG_E406_F	0.0178	0.2413	0.9358	0.0000	0.0036	0.0184	0.9417	0.5905	0.9906	0.9172	0.7473	0.9872	0.9898	0.9933	0.0000
PLSCR3_P751_R	0.0284	0.0000	0.3541	0.1290	0.0133	0.1737	0.0145	0.0168	0.1915	0.0000	0.0111	0.0073	0.1033	0.0178	0.0106
PLXDC1_P236_F	0.2621	0.0000	0.0123	0.0133	0.0118	0.1511	0.0145	0.0052	0.0000	0.0000	0.0128	0.1272	0.0056	0.0000	0.0899
PLXDC2_E337_F	0.0177	0.0000	0.0039	0.0152	0.0000	0.0000	0.0000	0.0000	0.0000	0.0000	0.0078	0.0000	0.0000	0.0000	0.0055
PLXDC2_P914_R	0.0270	0.2256	0.0276	0.4361	0.3458	0.0309	0.4746	0.0192	0.4017	0.0273	0.0261	0.0222	0.0265	0.4143	0.0220
PMP22_P975_F	0.3214	0.0000	0.9775	0.0000	0.0022	0.0000	0.9940	0.9932	0.9866	0.7882	0.8365	0.9902	0.9745	0.0000	0.0000
PODXL_P1341_R	0.0610	0.0110	0.0656	0.0147	0.0444	0.0061	0.0169	0.2678	0.0000	0.0000	0.0081	0.0086	0.0470	0.0000	0.0478
POMC_E254_F	0.6030	0.0000	0.0353	0.7228	0.0264	0.0156	0.0000	0.0047	0.0000	0.0000	0.0160	0.0064	0.0621	0.0000	0.0076
POMC_P400_R	0.0000	0.6129	0.0039	0.0000	0.0000	0.0000	0.0000	0.0000	0.0000	0.0000	0.0003	0.0110	0.3783	0.0123	0.0303
POMC_P53_F	0.0117	0.0000	0.0080	0.0086	0.0111	0.1237	0.0089	0.0073	0.0000	0.0000	0.0119	0.0460	0.0005	0.0182	0.0041
PPARD_P846_F	0.0000	0.0000	0.2503	0.0000	0.0000	0.0000	0.0000	0.0000	0.0000	0.9481	0.0087	0.0000	0.0000	0.0000	0.0047
PPARG_E178_R	0.0000	0.0000	0.1483	0.0000	0.0000	0.0000	0.0000	0.0062	0.0000	0.0000	0.0091	0.0070	0.1889	0.0000	0.0000
PPP2R1B_P268_R	0.0000	0.0000	0.0033	0.0000	0.2745	0.0028	0.0754	0.0030	0.0000	0.0000	0.0047	0.0000	0.0000	0.0000	0.0030
PRDM2_P1340_R	0.9905	0.0000	0.9640	0.0000	0.0000	0.0000	0.0052	0.0000	0.9778	0.9784	0.9706	0.0000	0.9674	0.9850	0.0000
PRKCDBP_E206_F	0.0000	0.0000	0.0809	0.0000	0.0000	0.0022	0.0049	0.0027	0.0000	0.0000	0.3522	0.0015	0.0000	0.0000	0.0000
PROK2_E0_F	0.0000	0.0000	0.0030	0.0000	0.0000	0.0000	0.0000	0.0000	0.0017	0.0000	0.0135	0.0053	0.0000	0.0238	0.0019
PROK2_P390_F	0.0000	0.1653	0.0000	0.0169	0.0000	0.0000	0.0096	0.0000	0.0000	0.0000	0.0000	0.0011	0.0000	0.0155	0.0011
PRSS8_E134_R	0.0000	0.6273	0.9826	0.9962	0.0498	0.1975	0.1339	0.9936	0.9860	0.9872	0.9886	0.9920	0.9879	0.9926	0.9917
PSCA_E359_F	0.0000	0.0000	0.1104	0.3904	0.0287	0.0000	0.0204	0.0000	0.0000	0.0000	0.0000	0.0040	0.0521	0.0000	0.0000
PSIP1_P163_R	0.0278	0.0000	0.0000	0.0000	0.0115	0.0035	0.0144	0.0057	0.0000	0.0000	0.0760	0.0081	0.0000	0.0090	0.0000
PTCH_E42_F	0.0112	0.0000	0.0886	0.5019	0.0000	0.0018	0.1834	0.0000	0.0000	0.0000	0.0127	0.0097	0.0655	0.0000	0.0143
PTCH2_P568_R	0.9737	0.0494	0.4829	0.0120	0.9811	0.0096	0.0344	0.0504	0.2780	0.0000	0.3477	0.6546	0.2707	0.0217	0.0056
PTEN_P438_F	0.0000	0.0000	0.0035	0.0821	0.0074	0.0061	0.1598	0.0000	0.0000	0.0000	0.0579	0.0055	0.0000	0.0000	0.0046
PTGS1_P2_F	0.7940	0.0000	0.0071	0.0175	0.2652	0.1591	0.0188	0.0097	0.0000	0.3279	0.0118	0.0117	0.0021	0.0000	0.0000
PTGS2_P308_F	0.0077	0.2142	0.0040	0.1760	0.1262	0.0227	0.0136	0.0085	0.0000	0.0000	0.0085	0.0063	0.0000	0.4590	0.0000
PTGS2_P524_R	0.2032	0.0000	0.0067	0.0000	0.0142	0.0068	0.0000	0.0073	0.0000	0.0000	0.0079	0.0038	0.0731	0.3892	0.0062
PTHLH_P15_R	0.0000	0.4709	0.2016	0.0000	0.0000	0.0045	0.0486	0.0017	0.0000	0.0000	0.9009	0.0000	0.9173	0.0091	0.8477

PTHR1_P170_R	0.2197	0.1143	0.9785	0.0000	0.0000	0.0000	0.0108	0.0059	0.9770	0.0000	0.9832	0.8918	0.9759	0.0000	0.0000
PTK2_P735_R	0.0154	0.0000	0.0088	0.0000	0.0078	0.0130	0.0153	0.3640	0.0000	0.0000	0.2337	0.0371	0.0000	0.0134	0.0159
PTK2B_P673_R	0.0000	0.0000	0.0039	0.0065	0.0000	0.0000	0.0090	0.0000	0.0000	0.0000	0.0078	0.0095	0.1418	0.0000	0.0064
PTPN6_E171_R	0.0081	0.0000	0.0000	0.0068	0.0076	0.0000	0.0000	0.0000	0.0000	0.0000	0.0000	0.0016	0.0000	0.0000	0.0000
PTPN6_P282_R	0.0091	0.0000	0.0000	0.0000	0.0000	0.0000	0.0043	0.0000	0.9188	0.0000	0.0000	0.0000	0.2823	0.0000	0.0000
PTPNS1_E433_R	0.0000	0.0000	0.0000	0.0000	0.0000	0.0000	0.0000	0.0000	0.0000	0.0000	0.0000	0.0000	0.0000	0.0059	0.0000
PTPNS1_P301_R	0.0000	0.0000	0.0094	0.3710	0.0895	0.0139	0.0000	0.0083	0.0000	0.0000	0.0107	0.0050	0.0025	0.0062	0.0074
PTPRF_E178_R	0.0096	0.0000	0.0347	0.0000	0.0049	0.0026	0.0000	0.0020	0.0000	0.0000	0.0078	0.3843	0.2888	0.0014	0.0017
PTPRG_E40_R	0.1020	0.0272	0.0133	0.2122	0.0126	0.0179	0.0096	0.1398	0.0000	0.0000	0.0143	0.0184	0.0037	0.4619	0.2515
PTPRG_P476_F	0.1049	0.0000	0.0049	0.0046	0.1361	0.0000	0.0346	0.1326	0.0000	0.0000	0.0122	0.0103	0.0000	0.0000	0.0975
PTPRO_E56_F	0.0070	0.2100	0.0000	0.0541	0.0156	0.0034	0.0758	0.0045	0.0000	0.0000	0.0018	0.0000	0.0000	0.0131	0.0097
PURA_P928_R	0.0000	0.0000	0.0000	0.9946	0.0000	0.0000	0.3815	0.0000	0.0000	0.0000	0.0000	0.0020	0.0000	0.0054	0.0000
PWCR1_P811_F	0.9901	0.9942	0.9798	0.0000	0.0000	0.0000	0.0830	0.0000	0.9808	0.9902	0.9699	0.8861	0.9734	0.9869	0.7218
PYCARD_E87_F	0.0382	0.0000	0.0140	0.0706	0.0736	0.0842	0.0106	0.0579	0.0000	0.0000	0.0164	0.5007	0.0044	0.0928	0.0093
PYCARD_P150_F	0.0124	0.0120	0.3148	0.0000	0.0095	0.0080	0.0082	0.0230	0.0000	0.0000	0.0352	0.0152	0.3540	0.0048	0.0075
RAB32_E314_R	0.0000	0.0000	0.0000	0.0214	0.0000	0.0042	0.0000	0.0000	0.2882	0.0000	0.0107	0.0068	0.0235	0.0069	0.0091
RAB32_P493_R	0.0000	0.0000	0.0000	0.0000	0.0028	0.0013	0.0000	0.0000	0.0000	0.0000	0.0012	0.0000	0.0000	0.0000	0.0000
RAF1_P330_F	0.0071	0.0000	0.0580	0.0071	0.0000	0.0019	0.0000	0.0002	0.0000	0.0000	0.0023	0.3948	0.0000	0.0007	0.0000
RAN_P581_R	0.0000	0.0000	0.8204	0.0000	0.0000	0.0000	0.0000	0.0016	0.0000	0.0000	0.7029	0.0000	0.7326	0.0000	0.9919
RARA_E128_R	0.0222	0.0000	0.1043	0.0080	0.0000	0.0039	0.0302	0.0000	0.0000	0.3695	0.0086	0.0000	0.0639	0.0000	0.4620
RARA_P176_R	0.0000	0.0000	0.0004	0.0098	0.0000	0.0000	0.0000	0.0000	0.6368	0.0000	0.0009	0.0013	0.0882	0.0000	0.0000
RARB_E114_F	0.0064	0.0000	0.0012	0.0000	0.0000	0.0020	0.0056	0.0000	0.0000	0.2373	0.0057	0.0029	0.0193	0.0057	0.0000
RARB_P60_F	0.0100	0.0000	0.0000	0.0000	0.0000	0.0000	0.0064	0.0028	0.0000	0.0000	0.0000	0.0000	0.1065	0.0000	0.0000
RARRES1_E235_F	0.0142	0.1221	0.0420	0.0198	0.0219	0.0095	0.0833	0.1388	0.0000	0.0000	0.0123	0.0097	0.0490	0.0193	0.0065
RASGRF1_E16_F	0.0000	0.0000	0.0057	0.0096	0.2269	0.0037	0.0060	0.1998	0.0000	0.0000	0.0124	0.3263	0.0030	0.0148	0.1659
RASGRF1_P768_F	0.0104	0.7780	0.0049	0.5499	0.0064	0.0000	0.0111	0.0579	0.0000	0.0000	0.0082	0.0973	0.0000	0.0244	0.0948
RASSF1_E116_F	0.0113	0.0000	0.0048	0.0000	0.4270	0.0081	0.0104	0.4126	0.0000	0.0000	0.0095	0.0000	0.0000	0.0067	0.1568
RASSF1_P244_F	0.3503	0.0000	0.0087	0.0000	0.0000	0.1548	0.3182	0.0000	0.0000	0.2784	0.0089	0.0108	0.0001	0.0000	0.0054
RBL2_P250_R	0.0104	0.0000	0.0994	0.1823	0.0052	0.3950	0.0000	0.0000	0.0000	0.0000	0.0121	0.0000	0.0000	0.0000	0.1811
RBP1_E158_F	0.0000	0.0000	0.0000	0.0000	0.0000	0.0000	0.0000	0.0002	0.0000	0.0000	0.0000	0.0000	0.0000	0.0000	0.0000
RBP1_P150_F	0.0000	0.0000	0.0000	0.0000	0.0000	0.0052	0.0020	0.0000	0.0000	0.0000	0.0000	0.0000	0.0000	0.0000	0.0000
RET_P717_F	0.0151	0.0000	0.0752	0.0000	0.7537	0.0065	0.3278	0.0029	0.0000	0.0000	0.0108	0.0164	0.0000	0.0111	0.0048
RET_seq_53_S374_F	0.1997	0.0000	0.0053	0.0000	0.0031	0.0767	0.0045	0.0044	0.0000	0.0000	0.2759	0.5292	0.0000	0.0000	0.4108

RHOC_P536_F	0.0030	0.0000	0.0000	0.0000	0.0029	0.0000	0.0000	0.0021	0.0000	0.0000	0.0044	0.0004	0.1081	0.0000	0.0000
RHOH_P121_F	0.0084	0.0000	0.0000	0.0000	0.0000	0.0000	0.0000	0.0013	0.0000	0.0000	0.0000	0.0000	0.0000	0.0000	0.0000
RHOH_P953_R	0.9932	0.0829	0.9534	0.0000	0.0000	0.0000	0.0000	0.0000	0.9849	0.7826	0.9820	0.0023	0.9674	0.0000	0.0000
RIPK1_P868_F	0.6499	0.4905	0.9689	0.7103	0.5821	0.3870	0.6455	0.4902	0.9729	0.9832	0.9563	0.3863	0.9729	0.8148	0.0435
RIPK2_E123_F	0.0000	0.0000	0.1041	0.0052	0.0000	0.0000	0.0093	0.0000	0.0000	0.0000	0.0073	0.0062	0.0000	0.0000	0.0000
RIPK3_P124_F	0.3551	0.0000	0.0074	0.0111	0.2688	0.0040	0.0121	0.0000	0.0000	0.0000	0.0113	0.2683	0.0001	0.0039	0.0069
RIPK3_P24_F	0.0118	0.0000	0.0000	0.0000	0.0000	0.0000	0.0000	0.0000	0.0000	0.0000	0.0015	0.0000	0.0000	0.0000	0.0014
RIPK4_E166_F	0.0077	0.0000	0.0013	0.0000	0.0000	0.0000	0.0052	0.0038	0.3480	0.0000	0.0038	0.0000	0.0970	0.0046	0.0000
RIPK4_P172_F	0.0169	0.0000	0.0748	0.0087	0.2293	0.0076	0.0089	0.0046	0.0000	0.5091	0.0094	0.0075	0.0854	0.0154	0.0094
ROR1_P6_F	0.0000	0.0000	0.0025	0.0000	0.0000	0.0000	0.0000	0.0000	0.0000	0.0000	0.0000	0.0000	0.0000	0.0000	0.0000
ROR2_P317_R	0.0000	0.0000	0.0000	0.0000	0.0047	0.0000	0.0000	0.0000	0.0000	0.0000	0.0005	0.0000	0.0000	0.0000	0.0076
RRAS_P100_R	0.0000	0.0000	0.0000	0.0000	0.0041	0.0026	0.0000	0.0027	0.0000	0.0000	0.0000	0.0000	0.0000	0.0071	0.0000
RUNX3_P247_F	0.0000	0.0215	0.9229	0.0000	0.0000	0.0059	0.0000	0.0000	0.9647	0.7343	0.0082	0.0000	0.9622	0.0187	0.0052
RYK_P493_F	0.0000	0.0000	0.0000	0.0000	0.0000	0.0000	0.0000	0.0000	0.0000	0.0000	0.8118	0.0052	0.1058	0.0000	0.0000
S100A2_E36_R	0.0000	0.0000	0.6422	0.0064	0.0025	0.0000	0.0000	0.0037	0.1957	0.0000	0.6307	0.8141	0.5313	0.0115	0.0000
S100A4_E315_F	0.0000	0.7271	0.0095	0.4692	0.2136	0.0118	0.3320	0.0077	0.5425	0.0000	0.0129	0.0103	0.1693	0.3194	0.0074
SCGB3A1_E55_R	0.0000	0.0000	0.1222	0.0000	0.0000	0.0000	0.0000	0.0000	0.0000	0.0000	0.0018	0.0000	0.0000	0.0060	0.0000
SCGB3A1_P103_R	0.1729	0.0029	0.0039	0.0000	0.0000	0.0000	0.0000	0.0000	0.0000	0.0000	0.0139	0.0000	0.0000	0.0000	0.3353
SEMA3A_P343_F	0.0000	0.0000	0.0001	0.0000	0.0000	0.0000	0.0000	0.0046	0.0000	0.0000	0.0039	0.0042	0.0000	0.0000	0.0000
SEMA3A_P658_R	0.0357	0.0000	0.2032	0.1013	0.0339	0.0154	0.0732	0.0979	0.0000	0.7055	0.0336	0.0291	0.2389	0.0385	0.0892
SEMA3C_E49_R	0.0149	0.0000	0.0990	0.0188	0.0139	0.0153	0.2015	0.0098	0.0011	0.0000	0.0172	0.0109	0.0112	0.0095	0.1271
SEMA3C_P642_F	0.1230	0.0000	0.0167	0.0000	0.0000	0.0000	0.0000	0.0000	0.0000	0.0000	0.0435	0.0130	0.0000	0.0000	0.0541
SEMA3F_E333_R	0.0190	0.0000	0.0048	0.0000	0.0138	0.0000	0.0221	0.0000	0.0000	0.0000	0.0061	0.0075	0.0000	0.0000	0.0007
SEMA3F_P692_R	0.0000	0.0000	0.1419	0.0000	0.0000	0.0000	0.0000	0.0000	0.0000	0.5322	0.0000	0.0070	0.0000	0.0000	0.0000
SEPT5_P441_F	0.0121	0.0000	0.4513	0.0483	0.1287	0.0000	0.1267	0.1199	0.0000	0.5529	0.6562	0.0150	0.4007	0.0000	0.0722
SEPT9_P58_R	0.0000	0.0000	0.7643	0.0000	0.5284	0.3401	0.1520	0.0000	0.9733	0.6630	0.4127	0.0337	0.6912	0.0000	0.9939
SERPINA5_E69_F	0.3779	0.8474	0.6761	0.0223	0.2790	0.9406	0.3443	0.0129	0.3408	0.8073	0.9801	0.3279	0.6640	0.6152	0.9592
SERPINB2_P939_F	0.9919	0.5315	0.9753	0.0000	0.9932	0.9924	0.9912	0.0000	0.9874	0.9875	0.9844	0.9823	0.9714	0.0000	0.0000
SERPINE1_E189_R	0.0118	0.0000	0.2988	0.0148	0.2658	0.1430	0.0117	0.0068	0.2083	0.0000	0.0176	0.0087	0.0223	0.0146	0.0125
SEZ6L_P249_F	0.0000	0.3418	0.0032	0.0967	0.1577	0.0013	0.0089	0.0036	0.0000	0.0000	0.6391	0.0024	0.0715	0.0000	0.0000
SEZ6L_P299_F	0.0000	0.0000	0.0051	0.0123	0.0074	0.0000	0.0000	0.0000	0.0000	0.0000	0.0039	0.0022	0.0000	0.0085	0.0037
SFN_E118_F	0.9956	0.3671	0.9702	0.9962	0.5358	0.9935	0.9299	0.7532	0.8734	0.7979	0.9816	0.9912	0.9875	0.8813	0.9929
SFRP1_E398_R	0.0748	0.0000	0.0018	0.0000	0.0000	0.0000	0.0000	0.0000	0.0000	0.0000	0.0023	0.0000	0.0000	0.0000	0.0000

SFRP1_P157_F	0.0114	0.0000	0.0023	0.0080	0.0082	0.0099	0.0058	0.0025	0.0000	0.0000	0.0074	0.0030	0.0000	0.0096	0.0097
SFTPA1_E340_R	0.1046	0.0000	0.9765	0.0000	0.9948	0.0000	0.0000	0.0000	0.9861	0.0000	0.9869	0.9869	0.9819	0.6128	0.0000
SH3BP2_P771_R	0.0110	0.0000	0.0597	0.0105	0.0000	0.0039	0.0000	0.0000	0.6281	0.0000	0.0000	0.0102	0.2511	0.0100	0.0054
SHB_P473_R	0.0072	0.7063	0.0024	0.0000	0.1229	0.0028	0.0000	0.0110	0.0000	0.0000	0.0052	0.0051	0.0000	0.0142	0.0028
SHB_P691_R	0.1075	0.0000	0.0120	0.0000	0.1237	0.1529	0.0000	0.0107	0.0000	0.0000	0.0113	0.0156	0.0510	0.0102	0.0102
SHH_E328_F	0.0000	0.0000	0.0000	0.0000	0.0000	0.0000	0.0000	0.0000	0.0000	0.0000	0.0000	0.0000	0.0465	0.0000	0.0000
SHH_P104_R	0.0000	0.0000	0.0063	0.0118	0.0089	0.0075	0.0082	0.0000	0.7661	0.0000	0.0190	0.0089	0.1265	0.0089	0.4134
SIN3B_P514_R	0.0000	0.0000	0.9796	0.0097	0.0000	0.0000	0.0000	0.0015	0.9708	0.9268	0.8262	0.9836	0.9754	0.0000	0.0000
SKI_E465_R	0.0084	0.0000	0.0802	0.0118	0.0116	0.0027	0.0000	0.0000	0.0000	0.0000	0.4869	0.0045	0.0000	0.0109	0.0000
SLC22A2_E271_R	0.4306	0.0000	0.9836	0.0000	0.9951	0.9925	0.0000	0.4040	0.9840	0.9079	0.9793	0.9903	0.9818	0.0000	0.9289
SLC22A3_P634_F	0.0096	0.0000	0.4399	0.0101	0.0000	0.0152	0.9963	0.0000	0.5862	0.6345	0.0138	0.0057	0.4065	0.5320	0.0044
SLC6A8_seq_28_S227_F	0.0000	0.0000	0.5016	0.0000	0.0000	0.0000	0.0000	0.0000	0.0000	0.9789	0.8849	0.0000	0.0680	0.0000	0.0000
SLIT2_P208_F	0.0081	0.5666	0.0093	0.5144	0.4674	0.0989	0.2601	0.3760	0.0000	0.0000	0.6874	0.1086	0.0000	0.5164	0.0061
SMAD2_P708_R	0.0082	0.0029	0.0045	0.0023	0.0066	0.0000	0.0047	0.0061	0.0000	0.0000	0.0059	0.0000	0.0000	0.0058	0.0000
SMAD2_P848_R	0.0091	0.0000	0.0850	0.0000	0.0000	0.0000	0.0000	0.0000	0.0000	0.0000	0.0043	0.0034	0.0977	0.0000	0.0000
SMAD4_P474_R	0.0000	0.0000	0.0000	0.0000	0.0000	0.0000	0.0021	0.0000	0.0000	0.0000	0.4003	0.0000	0.0000	0.0000	0.0000
SMARCA3_E20_F	0.0000	0.0000	0.0021	0.0000	0.0000	0.0000	0.0025	0.0000	0.0000	0.0000	0.0043	0.0024	0.1116	0.0000	0.0004
SMARCA3_P109_R	0.0466	0.0000	0.1181	0.4276	0.0182	0.0116	0.6355	0.0121	0.0000	0.0000	0.0132	0.0290	0.0426	0.0000	0.1940
SMARCA3_P17_R	0.0205	0.0000	0.0025	0.0000	0.0000	0.0000	0.0000	0.0089	0.0000	0.0000	0.0025	0.0000	0.0000	0.0000	0.0000
SMARCA4_P362_R	0.0056	0.0000	0.0064	0.0043	0.0600	0.0058	0.1028	0.0550	0.0000	0.4835	0.0080	0.0053	0.0004	0.0088	0.0042
SMO_E57_F	0.0000	0.0000	0.0014	0.0000	0.0000	0.0012	0.0000	0.0000	0.0000	0.0000	0.0033	0.0000	0.0000	0.0069	0.0000
SOD3_P225_F	0.0000	0.3995	0.8638	0.3592	0.2043	0.0000	0.2571	0.0000	0.9743	0.9859	0.0097	0.0456	0.9718	0.9928	0.1020
SOX1_P1018_R	0.0315	0.0316	0.0048	0.0063	0.0460	0.0000	0.0000	0.0287	0.0000	0.0000	0.6055	0.0031	0.0000	0.0368	0.0023
SOX1_P294_F	0.4347	0.8265	0.1794	0.6090	0.0165	0.0127	0.0110	0.3075	0.0000	0.0000	0.0114	0.0109	0.1147	0.6625	0.0191
SOX17_P287_R	0.0000	0.0000	0.0000	0.0000	0.0000	0.0000	0.0000	0.0000	0.8369	0.0000	0.0000	0.0000	0.0000	0.0000	0.0000
SOX17_P303_F	0.6832	0.4651	0.0908	0.7955	0.2256	0.8147	0.4089	0.7266	0.6987	0.3909	0.1227	0.1644	0.1543	0.9923	0.7405
SOX2_P546_F	0.0191	0.0000	0.0105	0.0216	0.0151	0.2649	0.0098	0.9929	0.0000	0.0000	0.0090	0.0531	0.0029	0.0273	0.2671
SPARC_E50_R	0.0101	0.0000	0.0314	0.0096	0.0085	0.0027	0.0077	0.0041	0.5239	0.0000	0.2312	0.0060	0.0401	0.0134	0.6712
SPARC_P195_F	0.1370	0.4576	0.2124	0.0090	0.0000	0.0000	0.0050	0.0039	0.0000	0.0000	0.2258	0.0052	0.3742	0.0000	0.0028
SPDEF_E116_R	0.2035	0.8343	0.5245	0.2262	0.1767	0.1287	0.2603	0.1766	0.1592	0.0181	0.0268	0.1445	0.0215	0.5980	0.0124
SPI1_E205_F	0.1283	0.0000	0.2879	0.1031	0.0173	0.1768	0.3022	0.0200	0.6393	0.0000	0.0279	0.2235	0.3724	0.1721	0.0202
SPP1_E140_R	0.0125	0.0000	0.0034	0.0000	0.0115	0.0000	0.0138	0.0083	0.0000	0.0000	0.0086	0.0000	0.0000	0.7966	0.0000
SRC_P297_F	0.9957	0.0000	0.9801	0.0000	0.1607	0.3559	0.3394	0.0954	0.9882	0.9890	0.1730	0.9902	0.9804	0.0000	0.9926

ST6GAL1_P164_R	0.0000	0.4799	0.0159	0.0000	0.0572	0.0093	0.0248	0.0000	0.0000	0.0000	0.0526	0.0000	0.0018	0.0147	0.1237
ST6GAL1_P528_F	0.0000	0.4240	0.1275	0.0000	0.0000	0.0000	0.0051	0.0000	0.0000	0.0000	0.5484	0.0024	0.1634	0.0171	0.0035
STK23_E182_R	0.0043	0.0000	0.8890	0.0000	0.0000	0.9950	0.0000	0.0000	0.8344	0.8751	0.4721	0.0051	0.9819	0.0310	0.0000
SYK_E372_F	0.1250	0.0000	0.0173	0.0000	0.0099	0.0076	0.1563	0.0116	0.0000	0.0000	0.0131	0.0094	0.0165	0.0616	0.2183
TAL1_E122_F	0.4588	0.7790	0.0050	0.6080	0.0109	0.0110	0.5089	0.4346	0.0000	0.0000	0.0103	0.0062	0.0000	0.1210	0.0120
TAL1_P594_F	0.0934	0.2513	0.0619	0.0000	0.0000	0.0000	0.1439	0.1623	0.0000	0.0000	0.0016	0.0022	0.0000	0.0000	0.0000
TAL1_P817_F	0.0095	0.0000	0.0000	0.0000	0.0000	0.0000	0.0000	0.0000	0.0000	0.0000	0.0000	0.0000	0.6572	0.0000	0.0000
TCF4_P175_R	0.0000	0.0171	0.0000	0.0000	0.0000	0.0000	0.0000	0.0000	0.0000	0.0000	0.0000	0.0000	0.0000	0.0000	0.0000
TCF7L2_E411_F	0.0000	0.0000	0.0011	0.0055	0.0059	0.0011	0.0000	0.0000	0.0000	0.0000	0.0040	0.0021	0.0000	0.0039	0.0049
TCF7L2_P193_R	0.0000	0.0000	0.0021	0.0100	0.0047	0.0059	0.0046	0.0053	0.0000	0.0000	0.0040	0.0057	0.0000	0.0069	0.0000
TERT_E20_F	0.1479	0.0000	0.0103	0.0115	0.0087	0.0051	0.0141	0.0046	0.0000	0.0000	0.0165	0.0091	0.0545	0.0851	0.2859
TERT_P360_R	0.2114	0.5722	0.0680	0.6651	0.0871	0.0275	0.4190	0.6262	0.0000	0.7139	0.0149	0.0223	0.1999	0.0265	0.2075
TES_E172_F	0.0146	0.0000	0.0217	0.0000	0.0078	0.0000	0.0000	0.0000	0.0000	0.0000	0.0047	0.0023	0.3549	0.0000	0.0000
TESK2_P252_R	0.0093	0.0184	0.1141	0.0172	0.0138	0.0551	0.0416	0.0042	0.0000	0.0000	0.0078	0.0382	0.0000	0.0084	0.0058
TFAP2C_E260_F	0.0114	0.0000	0.0000	0.0000	0.0575	0.0000	0.0088	0.0000	0.0000	0.0000	0.3959	0.0029	0.0000	0.0000	0.0000
TFAP2C_P765_F	0.0000	0.0000	0.0088	0.0056	0.0080	0.0546	0.0089	0.0000	0.0000	0.0000	0.0134	0.0136	0.0000	0.0081	0.0000
TFF2_P178_F	0.7642	0.5828	0.9698	0.8819	0.8254	0.9860	0.8412	0.7927	0.9810	0.2984	0.9818	0.9855	0.9809	0.9959	0.7759
TFF2_P557_R	0.9950	0.7073	0.9844	0.2232	0.1555	0.2945	0.0000	0.1037	0.9901	0.0000	0.9006	0.0835	0.9868	0.9945	0.9930
TFPI2_E141_F	0.0084	0.0000	0.0000	0.0068	0.0000	0.0000	0.0000	0.0008	0.0000	0.0000	0.0000	0.0002	0.0000	0.0064	0.0000
TFPI2_P152_R	0.5317	0.1369	0.0162	0.0353	0.1569	0.0122	0.3732	0.4195	0.0000	0.0000	0.0331	0.0133	0.1166	0.3193	0.4587
TFPI2_P9_F	0.0304	0.7308	0.0153	0.5801	0.5716	0.0094	0.0373	0.5992	0.0000	0.0000	0.0195	0.0125	0.0045	0.4412	0.5407
TFRC_P414_R	0.4307	0.2433	0.0047	0.0137	0.1728	0.1142	0.2214	0.0559	0.0000	0.0000	0.0192	0.0000	0.0000	0.0000	0.0000
TGFA_P558_F	0.0137	0.0000	0.0080	0.0176	0.0000	0.4038	0.0000	0.3961	0.0000	0.0000	0.0131	0.0119	0.0060	0.0138	0.4419
TGFA_P642_R	0.0088	0.0000	0.0013	0.0000	0.0000	0.0000	0.0000	0.0000	0.0000	0.0000	0.0054	0.0027	0.0000	0.0000	0.0000
TGFB1_P833_R	0.0000	0.0000	0.9837	0.0000	0.0000	0.0000	0.0032	0.0000	0.9660	0.9813	0.9837	0.9894	0.9813	0.9948	0.8623
TGFB2_E226_R	0.0249	0.0000	0.3731	0.0000	0.0000	0.0144	0.0000	0.0079	0.0000	0.0000	0.0063	0.0081	0.1176	0.0000	0.0036
TGFB2_P632_F	0.0113	0.1004	0.0137	0.5514	0.0092	0.0179	0.0145	0.0088	0.0000	0.0000	0.0223	0.0097	0.0083	0.0123	0.0000
TGFB3_E58_R	0.9943	0.6837	0.9885	0.0000	0.9867	0.0000	0.0000	0.9937	0.9829	0.8566	0.9871	0.9906	0.9875	0.9949	0.0000
TGFBI_P173_F	0.2708	0.0000	0.1721	0.0000	0.0114	0.1465	0.0099	0.0072	0.0000	0.0000	0.0179	0.0106	0.6370	0.0000	0.1534
TGFBI_P31_R	0.0135	0.0000	0.3185	0.0000	0.0000	0.0046	0.0076	0.0038	0.0000	0.0000	0.2146	0.0052	0.1275	0.0070	0.0142
TGFBR3_E188_R	0.0059	0.0000	0.0007	0.0000	0.0000	0.0040	0.0076	0.0000	0.0000	0.0000	0.0080	0.0053	0.0000	0.0000	0.0000
TGFBR3_P429_F	0.0000	0.0000	0.0000	0.0113	0.0044	0.0004	0.0000	0.0061	0.0000	0.4660	0.0041	0.0040	0.0000	0.0034	0.0028
THBS1_E207_R	0.0960	0.0000	0.0037	0.0000	0.0000	0.0000	0.0089	0.0000	0.0000	0.0000	0.0067	0.0075	0.0000	0.0117	0.0008

THBS1_P500_F	0.0181	0.0000	0.0938	0.0109	0.0168	0.0076	0.0030	0.0145	0.0018	0.0000	0.0240	0.0153	0.0935	0.0284	0.0099
THBS2_E129_F	0.0061	0.0000	0.0023	0.0000	0.0000	0.0033	0.0692	0.0000	0.0000	0.0000	0.0058	0.0037	0.0000	0.0000	0.0042
THY1_P149_R	0.0172	0.3485	0.0349	0.0233	0.0159	0.0204	0.3143	0.0116	0.0149	0.0000	0.0260	0.0199	0.0197	0.3188	0.3707
TIAM1_P188_R	0.0000	0.0000	0.0000	0.0000	0.0000	0.0031	0.0064	0.0000	0.0000	0.0000	0.7408	0.0000	0.0000	0.0000	0.0042
TIMP1_E254_R	0.0095	0.0000	0.4533	0.0461	0.0526	0.0081	0.0084	0.0528	0.0000	0.0000	0.0170	0.0098	0.5798	0.0450	0.0482
TIMP1_P615_R	0.9893	0.0000	0.9717	0.0000	0.0090	0.0000	0.9927	0.0000	0.9717	0.7428	0.9745	0.0040	0.9713	0.9898	0.0000
TIMP2_E394_R	0.2627	0.0000	0.0081	0.0000	0.0000	0.0000	0.0000	0.0020	0.0000	0.0000	0.0082	0.0015	0.0000	0.0057	0.0000
TIMP2_P267_F	0.0126	0.0030	0.0757	0.0064	0.0028	0.0000	0.0039	0.0000	0.0000	0.0000	0.0013	0.0000	0.0000	0.0023	0.7687
TIMP3_P1114_R	0.9948	0.8011	0.9871	0.9956	0.2758	0.2075	0.9941	0.9935	0.9881	0.9078	0.9882	0.9904	0.9853	0.9933	0.9940
TIMP3_P690_R	0.0929	0.7452	0.9885	0.0000	0.8722	0.0000	0.9953	0.9960	0.9816	0.9914	0.9920	0.9891	0.9881	0.0000	0.9046
TIMP3_seq_7_S38_F	0.0367	0.0000	0.1208	0.3739	0.1202	0.0000	0.1183	0.0000	0.0000	0.0000	0.0097	0.1161	0.0002	0.0133	0.0060
TJP1_P326_R	0.0668	0.0211	0.0143	0.1761	0.0262	0.0294	0.1000	0.1009	0.0000	0.0000	0.0264	0.0581	0.0070	0.0395	0.1074
TJP1_P390_F	0.0349	0.0000	0.0055	0.0000	0.0052	0.0023	0.0069	0.0000	0.0000	0.0000	0.0105	0.0074	0.0000	0.0147	0.0038
TK1_E47_F	0.3533	0.7331	0.0369	0.3466	0.4452	0.5075	0.0063	0.3938	0.8716	0.0000	0.9478	0.2422	0.2920	0.2207	0.2121
TK1_P62_R	0.0000	0.0008	0.0074	0.0191	0.0000	0.0025	0.4780	0.3678	0.0000	0.0000	0.0086	0.0117	0.0018	0.0993	0.2223
TMEFF1_E180_R	0.0000	0.0000	0.0057	0.0000	0.0000	0.0000	0.0000	0.0000	0.0000	0.0000	0.0000	0.0114	0.0000	0.0132	0.0000
TMEFF1_P234_F	0.0000	0.0000	0.0000	0.0000	0.0000	0.0000	0.0060	0.0000	0.0000	0.0000	0.0022	0.0000	0.0000	0.0000	0.0004
TMEFF2_P152_R	0.2762	0.1297	0.1003	0.3305	0.3780	0.3956	0.0539	0.3235	0.0518	0.0763	0.0690	0.0458	0.0632	0.4124	0.0694
TMEFF2_P210_R	0.0000	0.0000	0.4695	0.0000	0.0000	0.0000	0.0085	0.0034	0.0000	0.0000	0.0000	0.0000	0.0000	0.0086	0.0000
TMEM63A_E63_F	0.0000	0.0000	0.0000	0.0000	0.0006	0.0000	0.0000	0.0000	0.0000	0.0000	0.0013	0.0000	0.0000	0.0000	0.0000
TMPRSS4_E83_F	0.9911	0.0000	0.9826	0.0000	0.0281	0.0027	0.0194	0.0000	0.9833	0.9923	0.9323	0.0010	0.9854	0.0051	0.0018
TMPRSS4_P552_F	0.6838	0.0746	0.9782	0.5643	0.6200	0.0106	0.0668	0.0108	0.9777	0.0000	0.0361	0.9882	0.4324	0.6643	0.9803
TNC_P198_F	0.0000	0.1069	0.4526	0.0000	0.0000	0.0000	0.0065	0.0029	0.8778	0.0000	0.0000	0.0034	0.0000	0.0279	0.0000
TNC_P57_F	0.0155	0.1832	0.0217	0.0169	0.0099	0.0108	0.4787	0.0078	0.0000	0.0000	0.0090	0.0106	0.0858	0.5002	0.1407
TNF_P1084_F	0.9943	0.6551	0.9865	0.9966	0.3522	0.2327	0.9969	0.9956	0.9867	0.9487	0.9802	0.9904	0.9737	0.9942	0.9930
TNF_P158_F	0.0000	0.0000	0.0000	0.0072	0.0043	0.0000	0.0665	0.0000	0.0000	0.0000	0.0076	0.0000	0.0000	0.0049	0.0000
TNFRSF10A_P171_F	0.1054	0.0000	0.0074	0.0000	0.0127	0.0064	0.0077	0.1543	0.0000	0.0000	0.0078	0.0104	0.0029	0.0119	0.0072
TNFRSF10A_P91_F	0.0269	0.0000	0.0002	0.0000	0.0000	0.0032	0.0000	0.0000	0.0000	0.0000	0.0078	0.0026	0.0053	0.0177	0.0025
TNFRSF10B_P108_R	0.0863	0.0000	0.0228	0.0143	0.1105	0.0176	0.0684	0.0761	0.4198	0.0145	0.0198	0.0134	0.0679	0.0366	0.0159
TNFRSF10C_E109_F	0.0304	0.0000	0.0027	0.0325	0.0052	0.0218	0.2744	0.0500	0.0000	0.0000	0.0025	0.0000	0.0000	0.0100	0.0000
TNFRSF10D_E27_F	0.0000	0.0000	0.0041	0.0000	0.0067	0.0136	0.0026	0.0023	0.0000	0.0000	0.0103	0.0064	0.0510	0.0066	0.0000
TNFRSF10D_P70_F	0.0672	0.0000	0.0245	0.3099	0.0188	0.0298	0.1282	0.1779	0.0000	0.0000	0.0373	0.1038	0.0121	0.0116	0.0697
TNFRSF1B_E5_F	0.0134	0.0000	0.0125	0.0000	0.0159	0.0000	0.0000	0.0000	0.0000	0.0000	0.3713	0.0000	0.0023	0.0000	0.0055

TNFRSF1B_P167_F	0.0043	0.0000	0.0036	0.0055	0.0053	0.0000	0.0000	0.0000	0.0000	0.0000	0.0038	0.0000	0.0851	0.0000	0.0042
TNFSF10_E53_F	0.0096	0.0000	0.0603	0.0105	0.0058	0.0043	0.0287	0.0000	0.1578	0.0000	0.0114	0.0089	0.0035	0.0000	0.3424
TNFSF10_P2_R	0.0000	0.0000	0.0002	0.0000	0.0000	0.0000	0.4654	0.0000	0.4972	0.0000	0.9741	0.2564	0.0000	0.0167	0.0000
TNFSF8_E258_R	0.0119	0.0000	0.0703	0.0000	0.0059	0.0034	0.0120	0.0020	0.0000	0.0000	0.0063	0.0065	0.1042	0.9533	0.0028
TNFSF8_P184_F	0.0070	0.0000	0.0026	0.0128	0.0062	0.0009	0.0033	0.0109	0.0000	0.0000	0.8955	0.0044	0.0000	0.0090	0.0000
TNK1_P41_R	0.0285	0.6664	0.2705	0.7987	0.3241	0.1273	0.7353	0.0135	0.0000	0.0000	0.0189	0.0220	0.2965	0.0155	0.0052
TP73_E155_F	0.4460	0.2826	0.0675	0.6217	0.6192	0.0064	0.9954	0.5094	0.5424	0.0000	0.0138	0.0063	0.0052	0.0197	0.0076
TP73_P496_F	0.0000	0.0000	0.0367	0.0000	0.0064	0.0053	0.0000	0.0000	0.6443	0.0000	0.0138	0.0067	0.0000	0.0099	0.0038
TP73_P945_F	0.0097	0.0000	0.0738	0.0126	0.0088	0.8963	0.0473	0.0000	0.0000	0.0000	0.0097	0.0073	0.1064	0.0173	0.0721
TPEF_seq_44_S36_F	0.0780	0.0000	0.0065	0.4524	0.0000	0.0000	0.0070	0.0038	0.0000	0.0000	0.0195	0.0080	0.0000	0.0000	0.0000
TPEF_seq_44_S88_R	0.1780	0.0000	0.0129	0.0450	0.0242	0.0081	0.0089	0.0639	0.0000	0.0000	0.0093	0.5230	0.0079	0.5984	0.0270
TRIM29_P261_F	0.8563	0.9703	0.9804	0.9035	0.9957	0.8908	0.8412	0.9920	0.9850	0.9872	0.9856	0.6685	0.9827	0.9146	0.8177
TRIP6_E33_F	0.1726	0.3890	0.0071	0.3858	0.0101	0.1648	0.0077	0.0145	0.0000	0.0000	0.0128	0.2272	0.1579	0.1067	0.0074
TSG101_P257_R	0.0086	0.0000	0.1409	0.0000	0.0035	0.0008	0.0007	0.0000	0.0000	0.9535	0.0063	0.0028	0.1498	0.9832	0.0000
TUBB3_E91_F	0.9691	0.0000	0.3690	0.0027	0.9745	0.0008	0.5716	0.0013	0.1306	0.0000	0.0090	0.6471	0.0421	0.0000	0.0011
TUBB3_P364_F	0.0000	0.0000	0.0000	0.0000	0.0000	0.0000	0.0082	0.0000	0.0000	0.0000	0.0000	0.0000	0.0000	0.0000	0.0000
TUBB3_P721_R	0.0000	0.0000	0.0000	0.0000	0.0000	0.0083	0.0084	0.0000	0.0000	0.0000	0.0027	0.0000	0.0000	0.0003	0.0042
TUSC3_E29_R	0.0000	0.0000	0.0046	0.0000	0.0061	0.0035	0.0091	0.0000	0.0000	0.0000	0.0155	0.0064	0.0000	0.0100	0.0799
TUSC3_P85_R	0.0000	0.0000	0.0085	0.0000	0.0000	0.0116	0.0184	0.0089	0.0000	0.0000	0.0076	0.0000	0.0000	0.0211	0.0062
TWIST1_E117_R	0.0000	0.0000	0.0000	0.0000	0.0000	0.0000	0.0000	0.0000	0.0000	0.0000	0.0000	0.0008	0.0000	0.0000	0.0000
TWIST1_P355_R	0.0000	0.0000	0.0031	0.0065	0.2822	0.0152	0.0046	0.0000	0.0000	0.0000	0.0106	0.0057	0.0000	0.0074	0.0000
TWIST1_P44_R	0.0000	0.0000	0.0000	0.0058	0.0175	0.0000	0.0000	0.0000	0.0000	0.0000	0.0000	0.0000	0.0000	0.0000	0.0000
TYRO3_P501_F	0.0000	0.0000	0.0000	0.0000	0.0000	0.0033	0.0030	0.0000	0.0000	0.0000	0.0036	0.0023	0.0000	0.0000	0.0000
UBA52_P293_R	0.0095	0.0000	0.0000	0.0000	0.0000	0.0000	0.9877	0.0000	0.0000	0.0000	0.0073	0.0000	0.0000	0.0000	0.0000
UGT1A1_E11_F	0.9949	0.0000	0.9860	0.9949	0.0000	0.9939	0.0000	0.9943	0.9889	0.9825	0.9811	0.9092	0.9846	0.7074	0.9938
UGT1A1_P564_R	0.1020	0.3490	0.9462	0.9479	0.9424	0.1755	0.9620	0.5939	0.9925	0.8772	0.8736	0.9205	0.9233	0.7617	0.9217
UGT1A7_P751_R	0.9940	0.0152	0.9859	0.0000	0.9942	0.7819	0.0000	0.9926	0.9889	0.9889	0.9829	0.1702	0.9826	0.0000	0.0000
UNG_P170_F	0.0000	0.0000	0.0444	0.0154	0.0256	0.0046	0.0000	0.0000	0.0000	0.0000	0.0160	0.0377	0.0009	0.0000	0.0108
USP29_E274_F	0.9945	0.6210	0.9861	0.0000	0.9946	0.0000	0.6143	0.0000	0.9895	0.9883	0.9892	0.9887	0.9834	0.9927	0.9923
USP29_P282_R	0.0000	0.0000	0.9721	0.9970	0.0000	0.0000	0.0000	0.0000	0.9194	0.9772	0.9818	0.0000	0.9775	0.0000	0.0000
VAMP8_P114_F	0.0110	0.0000	0.0124	0.1856	0.0572	0.0087	0.0093	0.1411	0.0010	0.0000	0.0195	0.0090	0.0086	0.0742	0.1037
VAV1_E9_F	0.0960	0.0000	0.0118	0.0097	0.0000	0.0074	0.0000	0.0000	0.0000	0.0000	0.0142	0.0116	0.0056	0.0000	0.0032
VAV1_P317_F	0.0000	0.1522	0.0006	0.0000	0.0000	0.7699	0.0000	0.0000	0.0000	0.0000	0.0064	0.0000	0.1072	0.0127	0.0000

VAV2_E58_F	0.0182	0.2317	0.0223	0.1820	0.1287	0.0091	0.0119	0.0099	0.0086	0.1211	0.0240	0.0094	0.0707	0.0466	0.0277
VAV2_P1182_F	0.0000	0.0000	0.0000	0.0000	0.0000	0.0000	0.0000	0.0000	0.0000	0.0000	0.0000	0.0000	0.0000	0.0034	0.0064
VBP1_P12_R	0.0000	0.0000	0.1831	0.0000	0.9887	0.0212	0.0000	0.0000	0.0000	0.6656	0.0103	0.0000	0.8827	0.0450	0.0000
VEGFB_P658_F	0.0294	0.0000	0.1061	0.0097	0.0000	0.0557	0.0404	0.0305	0.0000	0.0000	0.0266	0.0047	0.0000	0.0125	0.0029
VIM_P811_R	0.0000	0.0000	0.0000	0.0056	0.0000	0.0000	0.0000	0.0024	0.0000	0.0000	0.0024	0.0031	0.0000	0.0000	0.0000
WEE1_P924_R	0.9954	0.6432	0.9847	0.0000	0.0000	0.9949	0.0000	0.9935	0.9900	0.9899	0.8359	0.8033	0.9832	0.9961	0.9960
WNT2_E109_R	0.0000	0.0000	0.0000	0.0000	0.0000	0.0000	0.0000	0.0000	0.0000	0.0000	0.0000	0.0000	0.0000	0.0000	0.0000
WNT2_P217_F	0.2049	0.2822	0.1221	0.2419	0.0773	0.2140	0.2733	0.0306	0.0395	0.0227	0.0317	0.0271	0.0908	0.1910	0.2179
WNT2B_P1185_R	0.4598	0.0000	0.0240	0.3793	0.0000	0.0000	0.0238	0.2058	0.2709	0.0000	0.0578	0.0089	0.0082	0.0000	0.0087
WNT5A_E43_F	0.0207	0.1047	0.0178	0.2999	0.2280	0.0200	0.2774	0.0110	0.0000	0.0118	0.0588	0.0086	0.0345	0.0250	0.0112
WNT5A_P655_F	0.3158	0.0000	0.0044	0.0000	0.0000	0.0051	0.0000	0.0000	0.0000	0.0000	0.0052	0.0055	0.0000	0.0000	0.0021
WNT8B_E487_F	0.4000	0.2575	0.9723	0.5031	0.9943	0.4351	0.3754	0.5689	0.9784	0.9710	0.4290	0.9891	0.9728	0.6941	0.3134
WNT8B_P216_R	0.9919	0.0000	0.9764	0.0000	0.0013	0.0000	0.0000	0.9917	0.9864	0.9867	0.9769	0.0000	0.9739	0.0000	0.0000
WRN_E57_F	0.3149	0.0000	0.1720	0.2019	0.2538	0.3913	0.2511	0.3422	0.3801	0.0000	0.0281	0.0151	0.0211	0.0659	0.3067
WRN_P969_F	0.9946	0.5573	0.9780	0.9960	0.0000	0.0000	0.9951	0.0000	0.9893	0.9728	0.9904	0.0000	0.9596	0.0092	0.0000
WT1_E32_F	0.0000	0.0000	0.0107	0.0000	0.0000	0.0715	0.0113	0.1370	0.0000	0.0000	0.0034	0.3182	0.0015	0.0131	0.0021
WT1_P853_F	0.0110	0.0000	0.0047	0.0000	0.0000	0.0267	0.0000	0.0000	0.0000	0.0000	0.0174	0.0000	0.1901	0.6070	0.0020
XRCC1_P681_R	0.4231	0.3363	0.9816	0.0000	0.8945	0.9157	0.0000	0.0034	0.9782	0.9528	0.9549	0.9890	0.9821	0.9931	0.9900
XRCC2_P1077_F	0.3928	0.8686	0.9863	0.9973	0.9929	0.0000	0.9952	0.9942	0.9885	0.9881	0.9918	0.9912	0.9846	0.9946	0.9866
YES1_P600_F	0.0097	0.0000	0.0140	0.0110	0.0107	0.0078	0.0617	0.0054	0.0000	0.0000	0.0237	0.0175	0.0104	0.0904	0.0146
ZIM3_E203_F	0.9948	0.5259	0.9820	0.9957	0.0000	0.0000	0.0000	0.9921	0.9886	0.9893	0.9853	0.9893	0.9814	0.9927	0.9915
ZIM3_P451_R	0.4833	0.7007	0.9847	0.9956	0.9940	0.9932	0.9955	0.9965	0.9890	0.9777	0.9892	0.8236	0.9856	0.0000	0.9933
ZIM3_P718_R	0.0000	0.1655	0.9793	0.9955	0.0000	0.9932	0.0000	0.9926	0.9856	0.0000	0.9880	0.9869	0.9803	0.5711	0.0000
ZMYND10_E77_R	0.0072	0.0000	0.0008	0.0000	0.0000	0.0000	0.0000	0.0000	0.3206	0.0000	0.5644	0.0000	0.1352	0.0000	0.0000
ZNF215_P71_R	0.0099	0.0000	0.2503	0.0112	0.0000	0.0046	0.0069	0.0008	0.0000	0.0000	0.2554	0.0089	0.2822	0.0112	0.0000
ZNF264_E48_R	0.0000	0.0000	0.0000	0.0016	0.0000	0.0000	0.0037	0.0000	0.0000	0.0000	0.0054	0.0001	0.0000	0.0000	0.0000
ZNFN1A1_E102_F	0.0175	0.1773	0.9217	0.1784	0.9582	0.0000	0.9928	0.9897	0.9821	0.8436	0.7058	0.9866	0.9793	0.9851	0.0115
ZNFN1A1_P179_F	0.9944	0.6668	0.9834	0.0000	0.4021	0.9952	0.3947	0.9180	0.9867	0.9886	0.9841	0.9864	0.9833	0.2555	0.1063

TargetID	BWS32	BWS33	BWS34	BWS35	BWS36	BWS37	BWS38	BWS39	BWS40	BWS41	BWS42	BWS43	BWS44	BWS45	BWS46
AATK_E63_R	0.0074	0.0000	0.0662	0.0000	0.0067	0.0000	0.1740	0.9028	0.9463	0.0096	0.2062	0.0000	0.9929	0.9808	0.7370
ABCA1_E120_R	0.0000	0.9943	0.9913	0.0000	0.9900	0.0000	0.9861	0.9614	0.9838	0.9890	0.9773	0.9917	0.8690	0.9865	0.9881
ABCA1_P45_F	0.0000	0.9932	0.9893	0.0000	0.0000	0.0000	0.9942	0.9847	0.9819	0.0000	0.9461	0.9878	0.0000	0.9758	0.9887
ABCB4_E429_F	0.0042	0.0000	0.0005	0.0000	0.0014	0.0000	0.0000	0.0059	0.0049	0.0000	0.0612	0.0045	0.0047	0.0046	0.0000
ABCC2_P88_F	0.0207	0.0000	0.0171	0.1009	0.0162	0.0141	0.2262	0.0206	0.0196	0.0000	0.9902	0.0428	0.1345	0.9007	0.7021
ABCC5_P444_F	0.0077	0.0260	0.3316	0.0014	0.0062	0.0235	0.0063	0.1078	0.5900	0.0079	0.0065	0.0106	0.0097	0.1688	0.0000
ABCG2_P178_R	0.9822	0.0338	0.0361	0.1235	0.0887	0.3730	0.3867	0.4998	0.4953	0.0329	0.6107	0.0537	0.0319	0.0338	0.0160
ABCG2_P310_R	0.9886	0.0000	0.9715	0.0000	0.0000	0.0000	0.0181	0.9863	0.9774	0.0000	0.0000	0.0000	0.9876	0.9854	0.9845
ABL1_P53_F	0.9758	0.3457	0.4812	0.2672	0.9924	0.4400	0.3765	0.6908	0.8591	0.3107	0.9672	0.9907	0.3339	0.0319	0.9193
ABL2_P459_R	0.0000	0.0000	0.0000	0.0000	0.0000	0.0000	0.0124	0.0000	0.0000	0.0000	0.0045	0.0000	0.0000	0.0000	0.0000
ABO_E110_F	0.0647	0.0061	0.1474	0.0000	0.0050	0.0000	0.0000	0.0090	0.2620	0.0077	0.0090	0.0000	0.1987	0.0063	0.0000
ABO_P312_F	0.0092	0.0106	0.0085	0.0000	0.8947	0.0956	0.0074	0.3841	0.0152	0.0311	0.0090	0.0102	0.0104	0.0107	0.0000
ACTG2_P455_R	0.0000	0.0000	0.0009	0.0000	0.0000	0.0000	0.0000	0.0000	0.0067	0.0000	0.0000	0.0000	0.0000	0.0000	0.0000
ACVR1_P983_F	0.0051	0.0000	0.0000	0.0000	0.0000	0.0000	0.0000	0.0051	0.0022	0.0011	0.0000	0.0000	0.0000	0.0000	0.0000
ACVR1B_E497_R	0.0173	0.0000	0.0180	0.4344	0.2337	0.0000	0.1192	0.0173	0.0334	0.0000	0.2025	0.0170	0.0069	0.0060	0.0000
ACVR1B_P572_R	0.0203	0.3240	0.0222	0.0580	0.9890	0.2976	0.2815	0.0114	0.0184	0.4125	0.0163	0.5687	0.0202	0.0222	0.0000
ACVR1C_P115_R	0.0039	0.0000	0.0003	0.0000	0.0000	0.0027	0.0000	0.0061	0.0065	0.0057	0.0042	0.0048	0.0039	0.0038	0.0000
ACVR1C_P363_F	0.9930	0.7869	0.0433	0.0000	0.9927	0.0074	0.9935	0.9863	0.9845	0.9951	0.0000	0.9929	0.9935	0.9882	0.9871
ACVR2B_E27_R	0.9904	0.0000	0.9910	0.0000	0.9897	0.3282	0.1816	0.9804	0.8036	0.1396	0.6610	0.6307	0.9742	0.9868	0.0000
ACVR2B_P676_F	0.0000	0.0000	0.0049	0.0403	0.0000	0.0000	0.0113	0.0000	0.0000	0.0000	0.0731	0.0000	0.0000	0.0000	0.0000
ADAMTS12_P250_R	0.0060	0.0088	0.0000	0.0000	0.0021	0.0000	0.0000	0.0013	0.0000	0.0000	0.0031	0.0000	0.0000	0.0000	0.0000
ADCYAP1_E163_R	0.0074	0.1380	0.0000	0.0000	0.0096	0.0000	0.0099	0.0168	0.0101	0.0000	0.1273	0.0000	0.0000	0.0035	0.0000
ADCYAP1_P398_F	0.9370	0.9550	0.9239	0.9020	0.9207	0.9746	0.9931	0.9844	0.9844	0.9535	0.9893	0.9901	0.9897	0.8641	0.8404
ADCYAP1_P455_R	0.0397	0.0151	0.0499	0.0366	0.8052	0.1274	0.1889	0.0243	0.0309	0.6713	0.0099	0.0412	0.0102	0.8671	0.0000
AFF3_P122_F	0.7232	0.0000	0.0000	0.5853	0.0033	0.0000	0.0076	0.2375	0.0086	0.0133	0.4450	0.0000	0.0251	0.0039	0.1817
AFF3_P808_F	0.0000	0.0000	0.0000	0.0000	0.0000	0.0000	0.0095	0.0037	0.0077	0.0000	0.0431	0.0088	0.0000	0.0066	0.6400
AFP_P824_F	0.1285	0.0064	0.0191	0.0000	0.5604	0.0075	0.2978	0.0118	0.0137	0.1025	0.2492	0.0132	0.0079	0.0103	0.0000
AGTR1_P154_F	0.0010	0.0000	0.0002	0.0000	0.0023	0.0000	0.0000	0.0000	0.0009	0.0000	0.0025	0.0001	0.0060	0.0000	0.0000
AGTR1_P41_F	0.0165	0.2390	0.3367	0.4594	0.4391	0.4544	0.1629	0.0336	0.0207	0.0226	0.0472	0.0283	0.0152	0.0210	0.0000
AHR_E103_F	0.0124	0.0000	0.0000	0.0000	0.0000	0.9706	0.9889	0.9625	0.9773	0.0000	0.9324	0.0000	0.0000	0.9823	0.9515
AHR_P166_R	0.5832	0.5141	0.6769	0.4645	0.7823	0.0049	0.4115	0.6616	0.7120	0.8947	0.8359	0.6056	0.4220	0.7375	0.6837

AIM2_E208_F	0.3165	0.5724	0.0210	0.0000	0.2937	0.5103	0.0000	0.0182	0.0155	0.0121	0.0074	0.0712	0.0114	0.0119	0.2372
AKT1_P310_R	0.0102	0.0086	0.0096	0.0000	0.0032	0.0000	0.0096	0.0116	0.0091	0.0092	0.0054	0.0046	0.0123	0.5699	0.0000
ALK_P28_F	0.2197	0.0495	0.0035	0.0000	0.0038	0.0059	0.0084	0.0131	0.1460	0.9716	0.0018	0.0089	0.0109	0.5546	0.1091
ALOX12_E85_R	0.0037	0.0000	0.0019	0.0000	0.0000	0.0000	0.0041	0.0015	0.0000	0.0000	0.0000	0.0000	0.0035	0.0012	0.0000
ALOX12_P223_R	0.9895	0.0000	0.6314	0.0000	0.0000	0.9941	0.8653	0.0263	0.9799	0.0000	0.9882	0.9828	0.0000	0.9681	0.9699
APBA1_E99_R	0.0000	0.0000	0.0000	0.0000	0.9854	0.0000	0.0000	0.0000	0.0000	0.0000	0.0000	0.6386	0.0000	0.0000	0.1652
APBA1_P644_F	0.0000	0.0000	0.0000	0.0000	0.0000	0.0000	0.0056	0.0000	0.0000	0.0000	0.0000	0.0000	0.0000	0.0000	0.0000
APBA2_P227_F	0.0047	0.0000	0.0015	0.0000	0.0000	0.0000	0.0000	0.0000	0.0042	0.0000	0.0000	0.0075	0.0042	0.0000	0.0000
APC_P280_R	0.0000	0.9950	0.0000	0.0000	0.0000	0.0000	0.0000	0.0000	0.9275	0.0000	0.0000	0.0000	0.0000	0.9886	0.0000
APOA1_P75_F	0.2098	0.0144	0.0081	0.6319	0.0000	0.1727	0.0000	0.0045	0.0000	0.0000	0.0000	0.0103	0.0000	0.0000	0.0000
APOC1_P406_R	0.0058	0.5776	0.3519	0.5463	0.0037	0.3322	0.1063	0.3602	0.0101	0.0062	0.0087	0.0044	0.0061	0.0098	0.7807
APP_E8_F	0.0108	0.0000	0.0000	0.4438	0.1102	0.0056	0.0056	0.0050	0.0029	0.0084	0.0062	0.0039	0.0031	0.0000	0.0000
APP_P179_R	0.0129	0.2062	0.0212	0.0000	0.5984	0.3429	0.2124	0.0275	0.0261	0.1865	0.3103	0.0220	0.3300	0.0307	0.0000
AR_P189_R	0.1670	0.3965	0.0071	0.0000	0.1492	0.0531	0.0000	0.0104	0.0153	0.0357	0.2153	0.0091	0.2334	0.1109	0.0000
AREG_E25_F	0.0000	0.0000	0.0010	0.0000	0.0000	0.0000	0.0000	0.0046	0.0021	0.0063	0.0000	0.0032	0.0032	0.0032	0.0000
ARHGDIB_P148_R	0.0140	0.1331	0.0323	0.0000	0.2588	0.1586	0.0244	0.2416	0.6803	0.0225	0.0231	0.0277	0.0135	0.4173	0.0033
ARNT_P238_R	0.3832	0.0083	0.0036	0.0614	0.0415	0.0000	0.5160	0.0148	0.0112	0.1022	0.0100	0.0000	0.0028	0.0166	0.0000
ASB4_P391_F	0.0000	0.0000	0.1596	0.0000	0.0522	0.0000	0.0000	0.0046	0.0150	0.1026	0.0042	0.0070	0.0000	0.0026	0.0000
ASB4_P52_R	0.0000	0.0000	0.0072	0.0000	0.0043	0.0000	0.0083	0.0038	0.0061	0.0077	0.0000	0.0072	0.0000	0.0000	0.0000
ASCL1_E24_F	0.0000	0.0000	0.0000	0.0000	0.0000	0.0000	0.0000	0.0000	0.0000	0.0000	0.0000	0.0000	0.0021	0.0000	0.0000
ASCL1_P747_F	0.1832	0.0410	0.0452	0.0000	0.9899	0.0000	0.0897	0.0050	0.4566	0.0000	0.0596	0.1017	0.0064	0.1504	0.0000
ASCL2_E76_R	0.0000	0.0000	0.0000	0.0000	0.0011	0.0000	0.0000	0.0013	0.0000	0.0000	0.0000	0.0000	0.0000	0.0026	0.0000
ASCL2_P360_F	0.0014	0.0034	0.0000	0.0000	0.0000	0.0039	0.0066	0.0014	0.0000	0.0000	0.0057	0.9760	0.0000	0.0000	0.0000
ASCL2_P609_R	0.0000	0.0130	0.0000	0.0125	0.0000	0.0000	0.0131	0.0000	0.0000	0.0127	0.0077	0.0000	0.0017	0.0000	0.0000
ATP10A_P147_F	0.0049	0.0000	0.0068	0.0000	0.0082	0.0000	0.0000	0.0069	0.0069	0.0062	0.0000	0.1305	0.0000	0.0252	0.0000
ATP10A_P524_R	0.0197	0.0000	0.0085	0.2214	0.2617	0.0000	0.0184	0.0225	0.0146	0.0000	0.0000	0.0166	0.0761	0.0105	0.0000
AXIN1_P995_R	0.4376	0.0119	0.0109	0.8226	0.0245	0.5986	0.5200	0.0170	0.0181	0.4529	0.0117	0.0204	0.0129	0.0134	0.0000
AXL_E61_F	0.0045	0.0000	0.0002	0.0000	0.0050	0.0000	0.0042	0.0024	0.0014	0.0058	0.0061	0.0000	0.0041	0.0049	0.0000
BAX_E281_R	0.6125	0.0180	0.0963	0.0000	0.0131	0.0055	0.0288	0.0197	0.0239	0.0210	0.6101	0.0165	0.0119	0.0982	0.0000
BCAM_E100_R	0.0153	0.0068	0.0140	0.0000	0.0090	0.3607	0.0000	0.0114	0.0126	0.0113	0.0091	0.0113	0.0082	0.0103	0.3069
BCAM_P205_F	0.1704	0.0037	0.0097	0.0000	0.0056	0.0046	0.9942	0.0191	0.4488	0.0123	0.0209	0.0250	0.2336	0.0123	0.0000
BCAP31_P1131_F	0.0159	0.0099	0.0150	0.9598	0.0136	0.8495	0.6179	0.0179	0.0198	0.0277	0.0205	0.0305	0.0137	0.0127	0.0000
BCL2L2_E172_F	0.0058	0.0000	0.0000	0.0000	0.0000	0.0000	0.0032	0.0000	0.0013	0.0000	0.0022	0.0000	0.0000	0.0019	0.0000

BCL2L2_P280_F	0.0000	0.0049	0.0000	0.0000	0.0059	0.0000	0.0000	0.1743	0.0085	0.0000	0.0089	0.0055	0.0013	0.0065	0.0000
BCL3_E71_F	0.0042	0.0000	0.0000	0.0000	0.0013	0.0000	0.0000	0.0038	0.0038	0.0074	0.0034	0.0051	0.0042	0.0032	0.0000
BCL3_P1038_R	0.0151	0.2035	0.0110	0.0791	0.2545	0.3906	0.0077	0.0069	0.0122	0.0188	0.0061	0.0118	0.0093	0.0066	0.0000
BCL6_P248_R	0.0000	0.0138	0.0043	0.0000	0.0016	0.0000	0.0038	0.0063	0.0056	0.0025	0.0000	0.0000	0.0057	0.0000	0.0000
BDNF_E19_R	0.0050	0.0800	0.1428	0.0000	0.0021	0.0028	0.0041	0.0474	0.0415	0.0109	0.0326	0.0072	0.1378	0.0307	0.0091
BDNF_P259_R	0.4705	0.5521	0.9909	0.8839	0.5030	0.7871	0.5370	0.9763	0.9841	0.9929	0.9927	0.9918	0.9881	0.9809	0.9875
BGN_E282_R	0.0000	0.0042	0.0023	0.0000	0.0044	0.0000	0.0072	0.0056	0.0051	0.0088	0.0000	0.0032	0.0056	0.0040	0.0000
BGN_P333_R	0.0053	0.0207	0.0095	0.5955	0.0026	0.9966	0.0050	0.2191	0.0049	0.0256	0.0044	0.6976	0.0044	0.2246	0.4794
BIRC4_P122_R	0.2824	0.0161	0.5487	0.0000	0.2508	0.0000	0.4021	0.0122	0.0118	0.0085	0.0110	0.7719	0.2455	0.0155	0.4150
BIRC5_E89_F	0.0579	0.0124	0.0106	0.0000	0.0888	0.0076	0.0098	0.0133	0.0140	0.0617	0.0788	0.0205	0.0135	0.0101	0.0000
BLK_P14_F	0.0056	0.0092	0.4921	0.0000	0.0061	0.0024	0.1593	0.0104	0.0079	0.1612	0.0103	0.2323	0.2163	0.0075	0.0000
BMP2_E48_R	0.3150	0.3914	0.0131	0.8376	0.3313	0.6767	0.2440	0.6087	0.0094	0.5485	0.2776	0.0226	0.0108	0.3033	0.0000
BMP2_P1201_F	0.0009	0.0006	0.0000	0.0000	0.0000	0.0000	0.0000	0.0000	0.0029	0.0000	0.0000	0.0008	0.0028	0.0000	0.0000
BMP3_E147_F	0.0000	0.0028	0.0000	0.0000	0.0000	0.0000	0.0000	0.0000	0.0000	0.0000	0.0000	0.0000	0.0000	0.0001	0.3165
BMP3_P56_R	0.0054	0.0000	0.0000	0.0000	0.0000	0.0035	0.0000	0.0000	0.0063	0.0000	0.0042	0.0020	0.0000	0.0040	0.0000
BMP4_P199_R	0.6136	0.7003	0.0071	0.0000	0.0602	0.1168	0.1032	0.8509	0.0185	0.1079	0.0850	0.0108	0.0417	0.0272	0.0000
BMP6_P398_F	0.9927	0.0000	0.8862	0.0000	0.9922	0.2697	0.9955	0.9889	0.9847	0.0000	0.0000	0.0000	0.9900	0.9731	0.9828
BMPR1A_P956_F	0.0137	0.1365	0.0067	0.0000	0.4141	0.0039	0.0078	0.0100	0.3672	0.1881	0.2212	0.0153	0.7753	0.0269	0.0000
BMPR2_E435_F	0.0000	0.0000	0.0000	0.0000	0.0000	0.0000	0.0000	0.0000	0.0000	0.0000	0.0000	0.0000	0.0000	0.0000	0.0000
BMPR2_P1271_F	0.4342	0.0035	0.0156	0.0000	0.5109	0.6413	0.0000	0.0148	0.2831	0.0000	0.0048	0.0065	0.0186	0.0363	0.0000
BSG_P211_R	0.1257	0.1121	0.0782	0.1695	0.0639	0.4493	0.4843	0.0836	0.0842	0.3253	0.0665	0.1089	0.1185	0.0604	0.0735
BTK_P105_F	0.0119	0.1440	0.0067	0.0000	0.2089	0.2224	0.1250	0.0135	0.0119	0.0097	0.0110	0.0177	0.0138	0.0169	0.0000
C20orf47_P225_R	0.0000	0.0070	0.0000	0.0000	0.0163	0.0000	0.0000	0.0000	0.0000	0.0000	0.0917	0.0085	0.0047	0.0062	0.0000
CALCA_E174_R	0.1526	0.1577	0.7281	0.2586	0.7474	0.0630	0.1485	0.0986	0.0818	0.1636	0.5719	0.8183	0.6437	0.0609	0.0824
CAPG_E228_F	0.0000	0.0027	0.0000	0.0000	0.0000	0.0000	0.0041	0.0087	0.0957	0.9939	0.0032	0.0033	0.0030	0.0077	0.0000
CASP10_E139_F	0.0031	0.0000	0.0157	0.0000	0.0040	0.0000	0.0000	0.0049	0.0050	0.0084	0.0060	0.0117	0.0737	0.0000	0.0000
CASP10_P186_F	0.0000	0.0000	0.0000	0.0000	0.0000	0.9948	0.9921	0.9794	0.9351	0.8567	0.0845	0.0000	0.0000	0.0024	0.9673
CASP2_P192_F	0.0501	0.0228	0.0571	0.1068	0.0353	0.3382	0.0331	0.0368	0.0313	0.0463	0.0243	0.0494	0.0620	0.0263	0.0216
CASP3_P420_R	0.0000	0.0000	0.0049	0.0000	0.0022	0.0000	0.0000	0.0075	0.0074	0.0000	0.0008	0.0000	0.0018	0.0098	0.0000
CASP6_P201_F	0.0351	0.0000	0.0084	0.0000	0.9867	0.0000	0.0108	0.4301	0.8149	0.0670	0.1694	0.8844	0.9862	0.9841	0.0000
CASP6_P230_R	0.0006	0.0167	0.0059	0.0000	0.0620	0.0010	0.0413	0.0095	0.3473	0.0059	0.0353	0.0127	0.0060	0.0073	0.5321
CAV1_P130_R	0.0257	0.3943	0.0101	0.1150	0.0543	0.0052	0.2869	0.0081	0.0062	0.0119	0.2369	0.1001	0.2937	0.0065	0.0000
CAV1_P169_F	0.1682	0.1573	0.1120	0.1139	0.0122	0.1272	0.1822	0.0250	0.0157	0.1643	0.1636	0.2430	0.0182	0.0125	0.0022

CAV2_E33_R	0.6615	0.0106	0.0121	0.0000	0.2010	0.2167	0.0414	0.6324	0.4495	0.1571	0.1673	0.4745	0.0098	0.6286	0.2147
CCKBR_P361_R	0.0104	0.0658	0.0189	0.0534	0.0076	0.2214	0.0108	0.0722	0.0143	0.0117	0.0115	0.0097	0.0107	0.0107	0.0000
CCKBR_P480_F	0.0072	0.0081	0.0000	0.5891	0.0011	0.0000	0.0000	0.0076	0.0000	0.0067	0.0001	0.0000	0.0021	0.0000	0.0000
CCNA1_E7_F	0.0000	0.9334	0.1676	0.0000	0.9886	0.0000	0.0000	0.0177	0.9796	0.0000	0.9881	0.9848	0.7351	0.9839	0.7840
CCNA1_P216_F	0.0000	0.0000	0.0000	0.0000	0.0000	0.0000	0.0078	0.0000	0.8916	0.0064	0.0011	0.0000	0.0100	0.0000	0.0000
CCNC_P132_R	0.0000	0.0000	0.0033	0.0000	0.0000	0.0000	0.0000	0.0069	0.0063	0.0125	0.0024	0.0074	0.0000	0.0017	0.0000
CCND1_E280_R	0.0000	0.0000	0.0000	0.0000	0.0030	0.0015	0.0000	0.6522	0.2044	0.0156	0.0000	0.9847	0.0000	0.0008	0.5605
CCND1_P343_R	0.0000	0.0000	0.0000	0.0000	0.0000	0.0000	0.0000	0.0000	0.0000	0.0000	0.0024	0.0000	0.0000	0.0000	0.0000
CCND2_P887_F	0.0000	0.0000	0.0026	0.0000	0.0015	0.0000	0.0000	0.0000	0.0000	0.0000	0.0000	0.0000	0.0025	0.0000	0.0000
CCND2_P898_R	0.4858	0.4391	0.7699	0.3434	0.7140	0.7433	0.3967	0.9870	0.0790	0.3891	0.5064	0.6865	0.5039	0.0659	0.8093
CCNE1_P683_F	0.0000	0.0000	0.0000	0.0000	0.0000	0.0000	0.0000	0.0000	0.0000	0.0000	0.0002	0.0000	0.0000	0.0000	0.0000
CD1A_P414_R	0.0063	0.2752	0.0072	0.0000	0.3015	0.5808	0.2512	0.0086	0.0112	0.1608	0.2773	0.0082	0.0717	0.0050	0.0000
CD2_P68_F	0.6888	0.0235	0.4415	0.0000	0.3466	0.1887	0.0117	0.9843	0.1435	0.0000	0.6157	0.0117	0.7882	0.4157	0.4675
CD34_P339_R	0.0097	0.1864	0.3127	0.0000	0.4531	0.1795	0.2336	0.0193	0.0121	0.0152	0.2497	0.3792	0.0139	0.0154	0.0000
CD34_P780_R	0.6205	0.8627	0.5973	0.0000	0.5675	0.5874	0.8263	0.8017	0.8551	0.8579	0.7308	0.7634	0.0538	0.6537	0.7144
CD40_E58_R	0.0018	0.0036	0.0038	0.0000	0.0000	0.2201	0.0000	0.0033	0.0027	0.0000	0.0040	0.0063	0.0000	0.0021	0.0000
CD40_P372_R	0.0174	0.0208	0.5351	0.7742	0.2028	0.1983	0.2281	0.0249	0.0234	0.1914	0.0211	0.0165	0.0229	0.0257	0.0017
CD44_E26_F	0.0016	0.2329	0.0002	0.0000	0.0000	0.0000	0.0000	0.0000	0.0040	0.0000	0.0032	0.0000	0.0067	0.0043	0.0000
CD44_P87_F	0.0000	0.0000	0.9908	0.0000	0.0047	0.0000	0.0000	0.0032	0.4341	0.0000	0.6490	0.0000	0.0000	0.0000	0.0000
CD86_P3_F	0.0000	0.0000	0.0000	0.0000	0.0326	0.0000	0.0000	0.0022	0.0030	0.0000	0.0000	0.0000	0.0000	0.0000	0.0000
CDC25B_E83_F	0.0000	0.0052	0.0050	0.0000	0.0000	0.0000	0.0019	0.0000	0.0000	0.0000	0.0000	0.0023	0.0000	0.0025	0.0000
CDC25B_P11_R	0.3754	0.0460	0.0091	0.0000	0.3322	0.2992	0.0054	0.0131	0.0144	0.2915	0.0000	0.0000	0.0000	0.0150	0.0000
CDH1_P52_R	0.0012	0.0000	0.0000	0.0000	0.0000	0.0000	0.0042	0.0000	0.0028	0.0000	0.0036	0.0009	0.0000	0.0002	0.0000
CDH11_E102_R	0.0096	0.0000	0.0051	0.0000	0.0020	0.0000	0.1245	0.0041	0.0046	0.0000	0.0051	0.0000	0.0000	0.0025	0.0021
CDH11_P203_R	0.0219	0.7011	0.0159	0.3894	0.5395	0.5171	0.0167	0.0189	0.0161	0.0216	0.4950	0.6200	0.0165	0.0149	0.0000
CDH11_P354_R	0.0026	0.0000	0.0000	0.0000	0.0036	0.0000	0.0051	0.0049	0.0102	0.0000	0.0033	0.0048	0.0000	0.0068	0.0000
CDH13_E102_F	0.0000	0.9933	0.0000	0.0000	0.9909	0.0000	0.9723	0.2387	0.9811	0.0000	0.9936	0.9877	0.0147	0.9867	0.9853
CDH17_E31_F	0.0074	0.0084	0.0603	0.0000	0.1494	0.0000	0.0000	0.0079	0.0142	0.0000	0.0066	0.0137	0.0000	0.0137	0.0000
CDH17_P532_F	0.6409	0.6459	0.9910	0.2376	0.9912	0.7047	0.5448	0.9814	0.9854	0.9921	0.9900	0.6870	0.9933	0.9863	0.9775
CDH3_E100_R	0.0144	0.2343	0.2032	0.0000	0.2158	0.2340	0.0153	0.0246	0.0176	0.1864	0.1761	0.0289	0.0264	0.0206	0.0043
CDH3_P87_R	0.0039	0.0074	0.0000	0.3701	0.0025	0.0000	0.0000	0.0053	0.0018	0.0000	0.0033	0.0046	0.0050	0.0017	0.0000
CDK10_E74_F	0.9876	0.4262	0.9924	0.1890	0.7398	0.6275	0.4954	0.9844	0.0513	0.4997	0.4426	0.0330	0.4761	0.7238	0.2551
CDK2_P330_R	0.0000	0.0164	0.0000	0.0000	0.0013	0.9955	0.0044	0.9833	0.9755	0.0000	0.0563	0.0000	0.0000	0.0013	0.9823

CDK6_E256_F	0.0000	0.0000	0.0033	0.0000	0.0039	0.0000	0.0058	0.2953	0.0123	0.0000	0.0036	0.0077	0.0073	0.0058	0.0000
CDK6_P291_R	0.0104	0.0100	0.0105	0.0000	0.6034	0.1950	0.3089	0.0135	0.0142	0.0162	0.2744	0.4260	0.0163	0.0182	0.0000
CDKN1A_E101_F	0.0000	0.0000	0.0064	0.0000	0.0082	0.0000	0.0000	0.0157	0.0106	0.0000	0.1345	0.1640	0.0113	0.6638	0.0000
CDKN1A_P242_F	0.0059	0.0082	0.0000	0.0000	0.0000	0.0037	0.0048	0.0157	0.0065	0.0000	0.0000	0.0000	0.0000	0.0000	0.0000
CDKN1B_P1161_F	0.0000	0.0000	0.0000	0.0221	0.0000	0.0000	0.0000	0.0000	0.6549	0.0062	0.0000	0.0000	0.0000	0.0000	0.0000
CDKN2A_E121_R	0.0103	0.0068	0.0067	0.0000	0.0030	0.1969	0.0051	0.0075	0.0089	0.0095	0.0127	0.0132	0.0044	0.1495	0.0000
CDKN2B_E220_F	0.0047	0.9958	0.0000	0.0000	0.0000	0.0000	0.0000	0.0000	0.0043	0.0000	0.0000	0.0061	0.6708	0.0036	0.7406
CDM_seq_21_S260_R	0.0048	0.0093	0.2001	0.0000	0.0000	0.0000	0.0107	0.0067	0.5180	0.9934	0.0000	0.0010	0.0039	0.9834	0.2966
CEACAM1_E57_R	0.0063	0.0044	0.4044	0.0000	0.0060	0.0000	0.0000	0.0057	0.0077	0.2343	0.0000	0.0033	0.0053	0.0000	0.0000
CEACAM1_P44_R	0.0040	0.0199	0.1144	0.0000	0.2707	0.2131	0.0000	0.0093	0.0113	0.0000	0.0122	0.0091	0.0094	0.0061	0.0000
CEBPA_P1163_R	0.1426	0.0086	0.2111	0.0000	0.1744	0.1448	0.1995	0.6659	0.1746	0.0139	0.0116	0.0128	0.1339	0.0148	0.0000
CEBPA_P706_F	0.0053	0.0000	0.0000	0.0000	0.0063	0.0000	0.0000	0.0057	0.3503	0.0089	0.0051	0.0000	0.0037	0.9849	0.0000
CFTR_P115_F	0.0016	0.0000	0.0039	0.0000	0.0025	0.0000	0.0000	0.0047	0.0063	0.0065	0.0088	0.0074	0.0052	0.0000	0.0000
CHD2_P451_F	0.0042	0.0069	0.0000	0.0000	0.0000	0.0000	0.0000	0.0000	0.9807	0.0000	0.0055	0.0037	0.0000	0.0027	0.0000
CHFR_P501_F	0.0053	0.0000	0.0042	0.0000	0.0007	0.2873	0.0102	0.0066	0.0076	0.0048	0.0014	0.0000	0.0000	0.0006	0.0000
CHFR_P635_R	0.0000	0.8598	0.0000	0.4441	0.9904	0.3299	0.9908	0.9832	0.9636	0.0000	0.0000	0.9888	0.0000	0.9845	0.9813
CHGA_E52_F	0.0074	0.0092	0.0000	0.1979	0.0040	0.1912	0.0068	0.0014	0.0027	0.0000	0.0055	0.0037	0.0066	0.0000	0.0000
CHI3L2_E10_F	0.0149	0.7463	0.0081	0.0000	0.0049	0.0000	0.0101	0.4940	0.1304	0.0224	0.0000	0.0109	0.8274	0.7505	0.3448
CLK1_P538_F	0.0000	0.0000	0.0000	0.0000	0.0000	0.0000	0.0038	0.0000	0.0011	0.0025	0.0046	0.0005	0.0007	0.0000	0.0000
COL18A1_P365_R	0.0028	0.0606	0.0144	0.1496	0.1840	0.1035	0.0132	0.0122	0.0206	0.0118	0.0151	0.2822	0.1880	0.9694	0.0000
COL1A1_P5_F	0.0000	0.0053	0.0000	0.0000	0.0000	0.0000	0.0045	0.0000	0.0000	0.0000	0.0002	0.0000	0.0000	0.0000	0.0000
COL1A2_E299_F	0.0000	0.0155	0.5782	0.0000	0.0039	0.0166	0.0102	0.0047	0.0708	0.0000	0.0071	0.2948	0.0071	0.3151	0.3793
COL1A2_P407_R	0.0000	0.0000	0.0000	0.0000	0.0024	0.0000	0.0087	0.0000	0.2250	0.0000	0.0000	0.6808	0.0000	0.0000	0.0000
COL1A2_P48_R	0.1588	0.0284	0.0178	0.1088	0.0508	0.1941	0.0314	0.0171	0.0438	0.1868	0.1060	0.0408	0.0270	0.0670	0.0000
COL4A3_E205_R	0.0000	0.0000	0.0000	0.0000	0.0000	0.0000	0.0000	0.0032	0.0000	0.0000	0.0000	0.0000	0.0000	0.0023	0.0000
COL4A3_P545_F	0.7278	0.0618	0.0426	0.4754	0.0299	0.8102	0.7598	0.7943	0.0301	0.0682	0.0601	0.9187	0.4555	0.0324	0.0244
COL6A1_P283_F	0.0153	0.0244	0.8433	0.0000	0.0234	0.0687	0.0087	0.0104	0.9036	0.1792	0.0194	0.0213	0.0128	0.0081	0.0000
COL6A1_P425_F	0.1129	0.0000	0.0123	0.0000	0.3668	0.0061	0.0061	0.1388	0.1667	0.0142	0.3377	0.0112	0.3451	0.0113	0.0000
COPG2_P298_F	0.1114	0.8483	0.0551	0.2374	0.7068	0.5556	0.5894	0.5259	0.0549	0.6339	0.1014	0.1169	0.6339	0.1252	0.0400
CPA4_P1265_R	0.7083	0.6383	0.0432	0.9360	0.6772	0.8367	0.7094	0.0334	0.0678	0.6804	0.6578	0.0666	0.0330	0.0642	0.0063
CREB1_P819_F	0.0083	0.0000	0.0000	0.0000	0.0654	0.0404	0.0057	0.0109	0.0106	0.0000	0.0020	0.0117	0.0065	0.0047	0.0000
CRIP1_P874_R	0.3798	0.4536	0.0087	0.6652	0.0072	0.0058	0.3733	0.0199	0.0111	0.0133	0.0107	0.0125	0.4644	0.0213	0.0000
CRK_P721_F	0.0054	0.0061	0.0781	0.0000	0.0025	0.0045	0.0063	0.0052	0.0217	0.0067	0.0049	0.0080	0.0047	0.0042	0.0000

CSF1_P217_F	0.0086	0.0686	0.0092	0.0000	0.2477	0.1418	0.1373	0.5584	0.0260	0.0182	0.1386	0.2266	0.9615	0.0338	0.0000
CSF1_P339_F	0.0000	0.0000	0.0000	0.0000	0.0000	0.0000	0.0000	0.0038	0.0000	0.0000	0.0000	0.0000	0.0000	0.0000	0.0000
CSF1R_E26_F	0.1516	0.0000	0.0070	0.2255	0.0018	0.0103	0.0000	0.0076	0.0040	0.0000	0.0043	0.0000	0.0039	0.0000	0.0000
CSF3R_P472_F	0.0330	0.1494	0.1790	0.0000	0.1885	0.1900	0.1249	0.0359	0.0357	0.1891	0.1619	0.0588	0.0280	0.0154	0.0120
CSPG2_E38_F	0.0544	0.0059	0.0050	0.0000	0.0019	0.0000	0.0000	0.0066	0.0068	0.0000	0.0064	0.0067	0.0043	0.0040	0.0000
CSPG2_P82_R	0.0058	0.0000	0.0000	0.0000	0.0000	0.0000	0.0000	0.0099	0.0068	0.0087	0.0070	0.0000	0.0000	0.0016	0.0000
CSTB_E410_F	0.0000	0.0000	0.0000	0.0000	0.0061	0.0000	0.0000	0.0047	0.0107	0.0000	0.1387	0.0000	0.9255	0.8925	0.5441
CTAG1B_P4_R	0.0085	0.0056	0.0032	0.0000	0.0000	0.0053	0.0067	0.0000	0.0000	0.0000	0.0000	0.0256	0.0019	0.0000	0.0000
CTAG1B_P77_F	0.0201	0.3542	0.6210	0.9022	0.9313	0.4445	0.3051	0.9327	0.0628	0.0223	0.2359	0.9052	0.0227	0.0570	0.2597
CTAG2_P1426_F	0.6148	0.0224	0.6182	0.6932	0.0148	0.8132	0.6637	0.0199	0.0151	0.7295	0.2448	0.0231	0.0104	0.0274	0.0000
CTGF_E156_F	0.0029	0.0030	0.0000	0.0000	0.0012	0.0000	0.0000	0.0000	0.0033	0.0000	0.0000	0.0065	0.0008	0.0000	0.0000
CTLA4_P1128_F	0.6553	0.2780	0.7854	0.9954	0.9923	0.9823	0.1694	0.8176	0.1861	0.4384	0.8582	0.8592	0.9863	0.8271	0.4305
CTNNA1_P185_R	0.0039	0.0023	0.0037	0.0000	0.0044	0.0059	0.4801	0.0014	0.0061	0.0000	0.0038	0.0055	0.0050	0.0029	0.0000
CTNNA1_P382_R	0.0064	0.1801	0.0000	0.3374	0.0055	0.9958	0.0065	0.0074	0.0078	0.9175	0.0068	0.0079	0.0044	0.0040	0.0000
CTNNB1_P757_F	0.0073	0.0556	0.0141	0.5398	0.0043	0.0520	0.1022	0.0158	0.0129	0.0648	0.0280	0.0179	0.0559	0.0656	0.0000
CTSD_P726_F	0.0000	0.0000	0.0000	0.0000	0.0000	0.0061	0.0000	0.0000	0.0000	0.0000	0.0000	0.0000	0.0000	0.0000	0.0000
CTSH_P238_F	0.0023	0.0000	0.0000	0.0000	0.0000	0.0000	0.0000	0.0000	0.0000	0.0000	0.0000	0.0000	0.0000	0.0000	0.0000
CTSL_P264_R	0.0123	0.0166	0.0039	0.0000	0.0041	0.0000	0.0052	0.0115	0.0060	0.0000	0.0000	0.1491	0.0000	0.0008	0.0000
CTSL_P81_F	0.0000	0.0036	0.0080	0.3660	0.0059	0.0000	0.0000	0.0078	0.0050	0.0097	0.0042	0.0075	0.0044	0.0019	0.0000
CTTN_E29_R	0.0055	0.0100	0.0000	0.0000	0.0000	0.0000	0.0000	0.0000	0.0126	0.0000	0.0000	0.0000	0.0050	0.0023	0.0000
CYP1A1_P382_F	0.0112	0.1453	0.5968	0.5110	0.0287	0.6112	0.4506	0.4837	0.0427	0.1814	0.4273	0.3859	0.0366	0.5301	0.0000
CYP2E1_P416_F	0.0000	0.0142	0.0750	0.0000	0.2702	0.0372	0.0079	0.0083	0.0593	0.0804	0.0028	0.0000	0.0861	0.2492	0.0000
DAB2_P35_F	0.0000	0.0159	0.0000	0.0000	0.0024	0.0000	0.0000	0.0000	0.0000	0.0000	0.0044	0.0004	0.0000	0.0000	0.0000
DAB2_P468_F	0.4037	0.1391	0.0265	0.0000	0.6907	0.0652	0.1664	0.0238	0.0550	0.0164	0.2336	0.0235	0.2378	0.0207	0.0082
DAB2IP_P9_F	0.0000	0.0000	0.0021	0.1666	0.0000	0.0000	0.0000	0.0000	0.0000	0.0000	0.0000	0.0000	0.0000	0.0000	0.0000
DAPK1_P10_F	0.0000	0.0000	0.0000	0.0000	0.0018	0.0000	0.0000	0.0000	0.0033	0.0045	0.0000	0.0000	0.0036	0.0005	0.9739
DAPK1_P345_R	0.0023	0.0000	0.0000	0.0000	0.0013	0.0000	0.0010	0.0000	0.7213	0.0000	0.0000	0.0000	0.0000	0.0000	0.0000
DBC1_P351_R	0.0000	0.0000	0.0000	0.0000	0.0009	0.0000	0.0000	0.0000	0.0000	0.0000	0.0033	0.0000	0.0046	0.0000	0.0000
DCC_P177_F	0.2592	0.4483	0.0532	0.7515	0.0133	0.5231	0.0199	0.3373	0.0136	0.0108	0.1647	0.0065	0.0082	0.4921	0.0000
DCC_P471_R	0.0126	0.2467	0.3388	0.1612	0.6404	0.0048	0.0094	0.0127	0.0112	0.0117	0.0121	0.0112	0.0149	0.0183	0.0000
DCN_P1320_R	0.0000	0.0000	0.0096	0.0000	0.0000	0.0000	0.0000	0.0038	0.0000	0.0000	0.0000	0.0000	0.0000	0.0000	0.0000
DDB2_P407_F	0.0000	0.0000	0.0000	0.0000	0.0011	0.0037	0.0000	0.0059	0.4952	0.0000	0.0036	0.0011	0.0000	0.9731	0.0000
DDB2_P613_R	0.0000	0.0104	0.0076	0.1445	0.2888	0.4339	0.2935	0.0091	0.0058	0.0087	0.1261	0.0073	0.1243	0.0050	0.0000

DDR1_E23_R	0.0042	0.0072	0.0000	0.0000	0.0032	0.0000	0.0081	0.0085	0.0256	0.0095	0.0000	0.0056	0.0063	0.0126	0.0000
DDR2_E331_F	0.0000	0.0000	0.0065	0.0000	0.9869	0.0687	0.0000	0.9797	0.9781	0.6980	0.0204	0.0764	0.9941	0.9824	0.9610
DES_E228_R	0.0000	0.0000	0.0000	0.0000	0.0000	0.0000	0.0000	0.0149	0.0106	0.0000	0.0000	0.0000	0.0000	0.0000	0.0000
DHCR24_P406_R	0.0138	0.0123	0.0074	0.0000	0.3734	0.0377	0.0536	0.0440	0.2249	0.0399	0.9252	0.0111	0.1070	0.9731	0.3549
DIO3_E230_R	0.0088	0.0000	0.0072	0.0000	0.0067	0.0000	0.0080	0.0047	0.0093	0.0000	0.0000	0.0000	0.0054	0.0094	0.0000
DIO3_P674_F	0.1538	0.1399	0.0163	0.1808	0.4550	0.3166	0.1214	0.0108	0.0144	0.0166	0.3174	0.3393	0.1757	0.0755	0.0000
DIRAS3_E55_R	0.0156	0.0218	0.0150	0.0000	0.0092	0.3222	0.1118	0.0209	0.0288	0.0181	0.0177	0.0000	0.0221	0.0000	0.0106
DKC1_E101_F	0.0000	0.0089	0.0000	0.0000	0.0000	0.0000	0.0000	0.0000	0.0000	0.0025	0.0000	0.0000	0.0000	0.0000	0.0000
DKFZP564O0823_E45_F	0.0000	0.0000	0.0000	0.0000	0.0041	0.0018	0.0080	0.0101	0.0007	0.0000	0.0000	0.0000	0.0026	0.0022	0.0000
DLC1_P88_R	0.0000	0.0000	0.0000	0.0000	0.0000	0.0040	0.0000	0.4705	0.0003	0.0000	0.0000	0.0000	0.0000	0.0014	0.2197
DLK1_E227_R	0.9452	0.9977	0.9856	0.7537	0.9965	0.9980	0.9978	0.9950	0.9940	0.9679	0.9961	0.9728	0.9966	0.9953	0.9882
DLL1_P386_F	0.0000	0.0021	0.9896	0.0000	0.0000	0.9939	0.0000	0.9813	0.9094	0.0000	0.0000	0.9830	0.0000	0.0000	0.9743
DLL1_P832_F	0.0043	0.0000	0.0076	0.0000	0.1679	0.2454	0.9944	0.0000	0.2856	0.9950	0.0000	0.0000	0.0000	0.0109	0.0000
DMP1_P134_F	0.0095	0.0120	0.6210	0.0000	0.9910	0.1839	0.2196	0.0135	0.1521	0.0123	0.0128	0.0192	0.0128	0.0158	0.0195
DNAJC15_E26_R	0.0000	0.0438	0.0039	0.0000	0.0041	0.0000	0.0000	0.0432	0.0105	0.0065	0.0068	0.0073	0.0062	0.0054	0.0000
DNASE1L1_P108_F	0.5511	0.5129	0.7760	0.4522	0.0147	0.6387	0.0247	0.9864	0.6923	0.6265	0.5269	0.8506	0.9097	0.0217	0.8673
DNMT1_P100_R	0.9873	0.4359	0.9909	0.0000	0.2920	0.0689	0.2634	0.9873	0.9818	0.4569	0.4668	0.9915	0.9924	0.9785	0.9893
DSC2_E90_F	0.0000	0.0000	0.9751	0.0000	0.0000	0.0000	0.0000	0.0000	0.8907	0.0000	0.0000	0.9597	0.9729	0.0000	0.9579
DSP_P440_R	0.3897	0.4034	0.5942	0.4274	0.0188	0.5591	0.4631	0.9810	0.9847	0.5253	0.9911	0.6554	0.9890	0.9673	0.9008
DST_E31_F	0.0114	0.0371	0.8344	0.0000	0.8270	0.0053	0.0316	0.9773	0.5648	0.8446	0.7867	0.9917	0.0382	0.9839	0.3769
DST_P262_R	0.0273	0.5627	0.0895	0.9452	0.0253	0.0158	0.4958	0.8470	0.0663	0.5151	0.5243	0.0696	0.0806	0.7351	0.0823
DUSP4_E61_F	0.0123	0.0000	0.0065	0.0000	0.0063	0.0000	0.0000	0.0073	0.0066	0.0095	0.0104	0.0084	0.0057	0.0081	0.0000
DUSP4_P925_R	0.1761	0.9938	0.0226	0.0000	0.0202	0.6242	0.0123	0.0241	0.0257	0.3231	0.0352	0.4529	0.6464	0.0184	0.4208
E2F3_P840_R	0.0017	0.0061	0.0015	0.0000	0.0000	0.0000	0.0058	0.6006	0.0072	0.9930	0.0000	0.9731	0.0192	0.0030	0.4642
E2F5_P516_R	0.9766	0.0000	0.9693	0.0000	0.9662	0.0000	0.0000	0.9412	0.9394	0.9814	0.9773	0.0720	0.0880	0.6237	0.9551
EDN1_E50_R	0.5860	0.9887	0.9847	0.9462	0.3009	0.3680	0.9820	0.5877	0.2980	0.9731	0.3976	0.9881	0.6461	0.4955	0.4371
EDN1_P39_R	0.4554	0.9359	0.9336	0.9730	0.5791	0.1909	0.9328	0.5274	0.1157	0.9907	0.0300	0.6673	0.9609	0.0058	0.5615
EDNRB_P148_R	0.0000	0.0000	0.0033	0.0000	0.9927	0.0000	0.0000	0.1044	0.8041	0.9930	0.0000	0.0042	0.0000	0.9780	0.0000
EFNA1_P591_R	0.0434	0.0324	0.1006	0.8272	0.1021	0.2410	0.2398	0.0119	0.0060	0.0124	0.0000	0.0283	0.0343	0.0000	0.8335
EFNA1_P7_F	0.9791	0.0000	0.0000	0.0000	0.0000	0.0000	0.0000	0.2364	0.0027	0.0028	0.0000	0.0031	0.0000	0.9683	0.8480
EFNB1_E69_F	0.9923	0.0047	0.2033	0.0000	0.0062	0.0000	0.0105	0.0087	0.0088	0.0000	0.0000	0.0000	0.0918	0.0064	0.2631
EFNB3_P442_R	0.0000	0.0000	0.0077	0.0000	0.0000	0.0000	0.0000	0.0086	0.0089	0.0017	0.0000	0.0020	0.0056	0.0057	0.0000
EGF_E339_F	0.0000	0.0067	0.0000	0.0000	0.0045	0.0000	0.0055	0.5914	0.3758	0.0000	0.0000	0.8432	0.0000	0.0000	0.8169

EGFR_E295_R	0.0068	0.0875	0.0063	0.0045	0.0073	0.0000	0.0153	0.0100	0.0097	0.7306	0.0070	0.0709	0.0056	0.0027	0.0000
EGFR_P260_R	0.0000	0.0000	0.0000	0.0000	0.0000	0.0000	0.0000	0.9799	0.0000	0.0000	0.0000	0.0022	0.0000	0.0000	0.7373
EGR4_P479_F	0.0185	0.9944	0.8808	0.4553	0.1281	0.6857	0.3499	0.9852	0.9861	0.0212	0.9894	0.9824	0.9835	0.8829	0.8343
EIF2AK2_E103_R	0.9904	0.9948	0.0000	0.0000	0.9913	0.0000	0.0000	0.9803	0.9779	0.0000	0.0000	0.0000	0.0000	0.0000	0.9716
EIF2AK2_P313_F	0.9943	0.0319	0.9931	0.0000	0.9927	0.0032	0.0103	0.9880	0.9854	0.9943	0.9921	0.1563	0.0321	0.9828	0.9873
ELK1_E156_F	0.0000	0.0000	0.0000	0.0000	0.0000	0.0000	0.0000	0.9780	0.9751	0.0000	0.9904	0.9878	0.0000	0.0000	0.9042
EMR3_P39_R	0.7952	0.4514	0.0139	0.2599	0.9930	0.5683	0.3180	0.9824	0.7703	0.0120	0.0234	0.0141	0.4408	0.0286	0.6473
ENC1_P484_R	0.9913	0.9943	0.9916	0.0000	0.9877	0.0000	0.3266	0.9879	0.9821	0.7758	0.0078	0.9931	0.2006	0.9865	0.9731
EPHA1_E46_R	0.0392	0.0550	0.0250	0.0648	0.0189	0.2227	0.1860	0.0236	0.6130	0.0548	0.0182	0.4373	0.0325	0.7795	0.6034
EPHA1_P119_R	0.0080	0.0081	0.0059	0.0000	0.0041	0.0000	0.0000	0.0056	0.0080	0.0124	0.0000	0.0072	0.0071	0.0046	0.0000
EPHA2_P203_F	0.0053	0.0062	0.9915	0.0000	0.5865	0.0000	0.0058	0.0051	0.5255	0.0077	0.0000	0.0953	0.0041	0.4702	0.0000
EPHA3_E156_R	0.0000	0.0000	0.0000	0.0000	0.0005	0.9956	0.0026	0.0004	0.0016	0.0000	0.0017	0.0000	0.0000	0.4373	0.0000
EPHA7_E6_F	0.4303	0.0030	0.9943	0.0000	0.3271	0.0000	0.0430	0.5241	0.4365	0.0000	0.4795	0.8410	0.0047	0.0119	0.0505
EPHA7_P205_R	0.9860	0.9942	0.3448	0.0000	0.9947	0.1667	0.1057	0.9854	0.9888	0.0467	0.8256	0.9619	0.9952	0.9861	0.9479
EPHA8_P256_F	0.0197	0.0125	0.0139	0.0000	0.1423	0.1531	0.0578	0.0280	0.0194	0.0165	0.1628	0.0237	0.0237	0.0183	0.0008
EPHB1_P503_F	0.0758	0.0035	0.0073	0.0000	0.0044	0.0000	0.0066	0.0105	0.2587	0.0120	0.0076	0.0293	0.5867	0.0061	0.1226
EPHB2_E297_F	0.8016	0.8466	0.7460	0.3511	0.7019	0.8835	0.7904	0.0358	0.1084	0.7897	0.7794	0.1031	0.0792	0.0348	0.0000
EPHB2_P165_R	0.0000	0.0000	0.0000	0.4363	0.0000	0.0000	0.0000	0.2177	0.9530	0.0101	0.0000	0.0000	0.0000	0.0000	0.9607
EPHB3_E0_F	0.0000	0.0051	0.0000	0.0000	0.0000	0.0000	0.0000	0.0000	0.0000	0.0000	0.8677	0.0000	0.0000	0.0019	0.0000
EPHB3_P569_R	0.1966	0.0769	0.3500	0.0729	0.3638	0.0000	0.2838	0.0601	0.4161	0.0125	0.3757	0.0105	0.0104	0.0602	0.0000
EPHB4_P313_R	0.0499	0.5534	0.5818	0.0000	0.0488	0.0000	0.0119	0.0386	0.0427	0.0029	0.0541	0.0389	0.5108	0.0226	0.3318
EPHB6_E342_F	0.7768	0.7992	0.1424	0.1295	0.5908	0.0000	0.8702	0.1025	0.1358	0.0000	0.0102	0.7571	0.2705	0.6913	0.0000
EPHB6_P827_R	0.0000	0.0068	0.0006	0.0000	0.0000	0.0000	0.0000	0.0024	0.0008	0.0000	0.0041	0.0075	0.0000	0.0016	0.0000
EPHX1_P1358_R	0.0000	0.0276	0.0000	0.0000	0.9920	0.9894	0.0000	0.0000	0.9843	0.9937	0.0000	0.4306	0.9887	0.9786	0.9867
EPM2A_P113_F	0.8499	0.8588	0.8206	0.9811	0.7624	0.9266	0.4495	0.0644	0.6812	0.8468	0.0647	0.9923	0.7810	0.2064	0.3668
EPM2A_P64_R	0.0346	0.4943	0.0160	0.1172	0.5975	0.5153	0.3591	0.5114	0.5542	0.0199	0.0150	0.0210	0.0187	0.0155	0.0000
EPO_E244_R	0.0000	0.0000	0.0000	0.0000	0.0002	0.0000	0.0000	0.5450	0.0000	0.0000	0.0005	0.0000	0.9876	0.7294	0.0000
EPO_P162_R	0.0000	0.0000	0.0000	0.3626	0.0000	0.0000	0.0000	0.0000	0.5175	0.0000	0.0021	0.0000	0.0000	0.0000	0.0000
EPS8_E231_F	0.2320	0.0000	0.0172	0.5810	0.9874	0.9966	0.0000	0.9831	0.0094	0.9948	0.1096	0.3795	0.0292	0.0072	0.9771
EPS8_P437_F	0.0000	0.0000	0.0000	0.4778	0.0018	0.0000	0.0000	0.0000	0.0000	0.0000	0.0000	0.0000	0.0025	0.0000	0.6776
ERBB2_P59_R	0.9816	0.0000	0.9925	0.0000	0.9886	0.0000	0.0515	0.9217	0.8764	0.0000	0.0000	0.0000	0.8171	0.9835	0.4557
ERBB3_E331_F	0.5455	0.4916	0.0337	0.7079	0.6169	0.8428	0.5771	0.5111	0.5400	0.5339	0.5178	0.7008	0.4905	0.4697	0.0000
ERBB3_P870_R	0.1953	0.2108	0.0045	0.0000	0.1886	0.2549	0.0081	0.0056	0.0062	0.0070	0.0091	0.0416	0.0078	0.0115	0.0000

ERBB4_P255_F	0.3843	0.0059	0.0104	0.0000	0.0087	0.5297	0.2762	0.0166	0.6358	0.1023	0.0091	0.0092	0.0195	0.0178	0.0000
ERBB4_P541_F	0.0000	0.0323	0.1762	0.0000	0.0394	0.0000	0.0000	0.9806	0.9665	0.0234	0.2530	0.0266	0.9078	0.7539	0.5498
ERCC1_P354_F	0.7676	0.9935	0.0058	0.0000	0.4669	0.0000	0.9931	0.8745	0.9326	0.8268	0.7050	0.0044	0.0000	0.4644	0.7164
ERCC3_P1210_R	0.9941	0.2926	0.9364	0.8081	0.5642	0.2318	0.3786	0.0144	0.0194	0.2533	0.2633	0.9869	0.2849	0.6338	0.8631
ERG_E28_F	0.0381	0.3186	0.7033	0.6346	0.6473	0.9961	0.0184	0.3633	0.5276	0.5496	0.0299	0.0328	0.4102	0.4882	0.2288
ERN1_P809_R	0.0000	0.2403	0.0057	0.0000	0.9888	0.9960	0.9740	0.0857	0.9763	0.3531	0.2187	0.9870	0.9905	0.0000	0.9803
ESR1_E298_R	0.9925	0.7816	0.7735	0.9482	0.7250	0.8697	0.6828	0.8340	0.0484	0.7419	0.0278	0.8042	0.9907	0.9880	0.9805
ESR1_P151_R	0.0079	0.1844	0.9907	0.0000	0.0912	0.1847	0.0565	0.1081	0.0806	0.9937	0.6156	0.0052	0.0036	0.8078	0.0000
ESR2_E66_F	0.1006	0.4379	0.3876	0.1345	0.5237	0.4226	0.9684	0.3355	0.0416	0.3934	0.3723	0.6658	0.6397	0.0367	0.0441
ESR2_P162_F	0.2788	0.0203	0.0211	0.0966	0.6729	0.2963	0.2837	0.9779	0.0194	0.0419	0.0276	0.0343	0.0221	0.9880	0.0058
ETS1_E253_R	0.0276	0.0960	0.0176	0.0092	0.1841	0.1121	0.1106	0.1466	0.0546	0.1135	0.0198	0.0243	0.0226	0.7991	0.0000
ETS1_P559_R	0.0000	0.9953	0.0407	0.0319	0.9923	0.0000	0.0000	0.9875	0.9826	0.0000	0.9915	0.9887	0.9901	0.9866	0.9871
ETS2_P684_F	0.0150	0.5634	0.0127	0.8898	0.0114	0.5936	0.9941	0.3712	0.7394	0.9940	0.0750	0.6709	0.5331	0.9661	0.7996
ETS2_P835_F	0.0000	0.0000	0.2909	0.0000	0.4037	0.4835	0.0000	0.0076	0.1201	0.3161	0.2201	0.0000	0.0013	0.2543	0.0000
ETV1_P235_F	0.9918	0.0000	0.9867	0.0000	0.9837	0.7273	0.9915	0.9876	0.9619	0.0000	0.2647	0.0788	0.0000	0.9864	0.9473
ETV1_P515_F	0.0036	0.0000	0.0000	0.0000	0.0000	0.0000	0.0000	0.1894	0.0248	0.1106	0.0103	0.0004	0.5734	0.0000	0.0000
ETV6_E430_F	0.6618	0.7013	0.0091	0.0000	0.0056	0.0084	0.0104	0.6031	0.1127	0.0141	0.9208	0.9384	0.1394	0.0089	0.2079
EVI1_E47_R	0.9904	0.1033	0.3931	0.0527	0.6687	0.2887	0.5783	0.8899	0.1508	0.0193	0.0278	0.5627	0.5656	0.9561	0.9742
EVI1_P30_R	0.0000	0.0000	0.0000	0.0000	0.0025	0.0000	0.0000	0.0000	0.3559	0.0093	0.0031	0.0092	0.0085	0.0021	0.0000
EVI2A_P94_R	0.7760	0.0083	0.0045	0.0000	0.0051	0.0000	0.9938	0.0153	0.0138	0.0000	0.0049	0.0093	0.9875	0.0031	0.0000
EXT1_E197_F	0.4031	0.4272	0.5182	0.6455	0.5442	0.4151	0.4493	0.0168	0.5661	0.2675	0.0207	0.0201	0.3069	0.0281	0.4675
EYA4_E277_F	0.0334	0.0001	0.1939	0.0000	0.0019	0.0047	0.9938	0.0052	0.2538	0.0037	0.0061	0.5584	0.1261	0.0091	0.0000
EYA4_P508_F	0.0000	0.0000	0.0028	0.0000	0.0000	0.0000	0.0000	0.0000	0.3235	0.4865	0.0000	0.9790	0.0000	0.4593	0.6757
EYA4_P794_F	0.9931	0.9944	0.5834	0.0000	0.0000	0.0000	0.0000	0.0002	0.0000	0.0050	0.0000	0.0091	0.5378	0.9796	0.9757
F2R_P839_F	0.0000	0.0000	0.3288	0.0000	0.9907	0.0050	0.9944	0.9886	0.9777	0.0000	0.9908	0.1629	0.0000	0.0018	0.8818
F2R_P88_F	0.0034	0.0000	0.0000	0.0000	0.6624	0.2017	0.0034	0.3379	0.8146	0.0000	0.0069	0.0124	0.0038	0.0001	0.7033
FABP3_E113_F	0.0055	0.8679	0.0129	0.3825	0.7502	0.0000	0.0086	0.6330	0.4104	0.0000	0.0193	0.4154	0.0093	0.6132	0.4953
FABP3_P598_F	0.6371	0.9896	0.2053	0.9927	0.9889	0.3485	0.3722	0.3079	0.1535	0.9947	0.9822	0.6898	0.6221	0.3416	0.4234
FANCE_P356_R	0.0123	0.0000	0.0000	0.0000	0.0000	0.0000	0.0000	0.1524	0.0000	0.9907	0.0085	0.0000	0.9240	0.5679	0.8016
FANCF_P13_F	0.6090	0.6642	0.9827	0.9022	0.6394	0.7808	0.5997	0.6437	0.9141	0.0514	0.6633	0.1641	0.6423	0.5542	0.0121
FANCG_E207_R	0.1400	0.1192	0.1492	0.9932	0.1124	0.9342	0.0600	0.0844	0.0780	0.2225	0.0579	0.3791	0.4041	0.0512	0.1054
FAS_P322_R	0.0032	0.1502	0.9874	0.0000	0.0112	0.9957	0.0051	0.9740	0.9628	0.0000	0.3968	0.1155	0.7100	0.9699	0.0000
FASTK_P257_F	0.9054	0.7704	0.0766	0.0007	0.0780	0.0969	0.0871	0.0312	0.3715	0.0875	0.0876	0.0822	0.8268	0.0831	0.7259

FAT_P973_R	0.0000	0.0000	0.2524	0.0000	0.2910	0.7271	0.1201	0.0125	0.0174	0.4471	0.0000	0.0735	0.3212	0.0144	0.0000
FER_P581_F	0.9786	0.2413	0.9806	0.0000	0.2822	0.2174	0.3252	0.9229	0.8824	0.9795	0.8753	0.9882	0.9550	0.3425	0.7516
FES_P223_R	0.0000	0.0000	0.0000	0.0000	0.9898	0.0000	0.0000	0.0000	0.8354	0.0000	0.0000	0.9819	0.9851	0.9725	0.9826
FGF1_E5_F	0.4000	0.3717	0.0736	0.2418	0.9900	0.4883	0.9597	0.8744	0.8915	0.0435	0.3478	0.3128	0.9090	0.8586	0.9253
FGF12_E61_R	0.6496	0.3451	0.0066	0.0000	0.5944	0.3927	0.0074	0.0159	0.0121	0.2094	0.6959	0.9202	0.0107	0.8479	0.0000
FGF12_P210_R	0.0047	0.0634	0.6375	0.0000	0.9929	0.3996	0.0080	0.9857	0.9828	0.1727	0.9929	0.1367	0.1895	0.9851	0.9808
FGF2_P153_F	0.0160	0.1367	0.2192	0.8356	0.0128	0.2186	0.1631	0.3852	0.0250	0.1584	0.0322	0.2621	0.9561	0.0182	0.0053
FGF2_P229_F	0.0609	0.0438	0.4919	0.6978	0.0239	0.5225	0.0415	0.7173	0.0463	0.4751	0.2328	0.0473	0.0424	0.3817	0.3210
FGF3_E198_R	0.9893	0.9920	0.9875	0.9498	0.9845	0.9935	0.9906	0.9776	0.5286	0.9909	0.9875	0.9881	0.9855	0.9769	0.7411
FGF3_P171_R	0.7033	0.0096	0.8902	0.0000	0.9923	0.0216	0.8149	0.8758	0.9327	0.8261	0.0000	0.5026	0.8397	0.0009	0.6095
FGF5_E16_F	0.0000	0.9944	0.0387	0.0000	0.8658	0.9961	0.0000	0.9879	0.3246	0.0000	0.9907	0.3269	0.9871	0.9796	0.6335
FGF5_P238_R	0.7281	0.0078	0.0012	0.0000	0.0000	0.0000	0.9948	0.8745	0.2141	0.0000	0.0143	0.0077	0.4429	0.0272	0.0545
FGF6_E294_F	0.7395	0.7766	0.7804	0.9504	0.0467	0.8350	0.9932	0.9756	0.0575	0.7765	0.0541	0.0434	0.9913	0.7901	0.9814
FGF7_P44_F	0.7765	0.0000	0.0015	0.0000	0.0020	0.0000	0.0000	0.6615	0.0050	0.0000	0.0000	0.0000	0.0000	0.8763	0.2742
FGF8_E183_F	0.0000	0.3374	0.3779	0.0000	0.1889	0.0765	0.1897	0.0628	0.0416	0.0000	0.2281	0.0000	0.1169	0.0142	0.0000
FGF8_P473_F	0.0000	0.0000	0.0000	0.0000	0.6345	0.0000	0.0000	0.0000	0.9739	0.0000	0.0000	0.0040	0.0028	0.0000	0.1481
FGFR1_E317_F	0.0122	0.0085	0.1579	0.0000	0.1360	0.0000	0.0000	0.0613	0.0210	0.0130	0.0135	0.0157	0.0000	0.1615	0.9716
FGFR2_P266_R	0.0108	0.2509	0.0159	0.0000	0.4110	0.2444	0.0122	0.0073	0.0100	0.3064	0.1784	0.0114	0.0070	0.0107	0.0000
FGFR3_E297_R	0.0083	0.9954	0.0000	0.0000	0.0603	0.0000	0.0000	0.0000	0.9034	0.0000	0.0000	0.0000	0.0155	0.9752	0.9737
FGFR3_P1152_R	0.0000	0.7837	0.3309	0.0000	0.9907	0.6886	0.6487	0.9443	0.9339	0.0024	0.8794	0.9888	0.5965	0.9195	0.8363
FHIT_E19_R	0.9923	0.0000	0.0000	0.0000	0.0000	0.0000	0.9920	0.9755	0.6865	0.0000	0.9908	0.9833	0.0000	0.9873	0.9329
FHIT_P93_R	0.0040	0.0849	0.0055	0.0000	0.0000	0.0000	0.0000	0.0057	0.0088	0.0000	0.0000	0.0000	0.0044	0.0085	0.5870
FHL1_E229_R	0.0000	0.0028	0.0000	0.0000	0.0028	0.0000	0.0034	0.3966	0.0014	0.0000	0.0000	0.0000	0.0007	0.0025	0.0000
FLI1_P620_R	0.9863	0.9824	0.0502	0.0000	0.0000	0.2338	0.0000	0.0206	0.9626	0.0000	0.0000	0.9788	0.8063	0.9588	0.7576
FLT1_E444_F	0.0166	0.7793	0.6803	0.8685	0.0133	0.8961	0.0177	0.0171	0.8053	0.7954	0.6841	0.0393	0.4384	0.7938	0.8375
FLT1_P302_F	0.8212	0.2242	0.0227	0.0543	0.0147	0.2583	0.1695	0.0516	0.4261	0.2282	0.6887	0.0325	0.0271	0.0298	0.0059
FLT1_P615_R	0.1381	0.1599	0.2167	0.0496	0.3969	0.2128	0.0111	0.9817	0.0096	0.1306	0.0258	0.0146	0.9757	0.1382	0.3678
FLT3_E326_R	0.0074	0.3251	0.0318	0.0000	0.0121	0.3503	0.2214	0.0108	0.1411	0.0135	0.0067	0.0067	0.0051	0.3820	0.0000
FLT4_P180_R	0.9795	0.9840	0.0851	0.0808	0.9923	0.9844	0.1219	0.9590	0.9868	0.0424	0.9727	0.2556	0.9328	0.8127	0.9614
FMR1_P62_R	0.5518	0.5385	0.5227	0.7504	0.4233	0.9239	0.4794	0.6224	0.3405	0.6625	0.4770	0.6789	0.5775	0.2650	0.4611
FN1_E469_F	0.0064	0.0000	0.0032	0.0000	0.0000	0.0000	0.0000	0.0000	0.0000	0.0000	0.0000	0.0073	0.0052	0.0000	0.0000
FN1_P229_R	0.0128	0.9951	0.0072	0.0000	0.0110	0.4506	0.2744	0.0188	0.0178	0.0133	0.4136	0.0170	0.0141	0.0221	0.2009
FOSL2_E384_R	0.0082	0.1072	0.7509	0.0000	0.9922	0.2581	0.2519	0.0084	0.9714	0.9956	0.7474	0.9924	0.9927	0.0062	0.9050

FRK_P36_F	0.0102	0.0000	0.0063	0.0000	0.0057	0.0000	0.0022	0.0191	0.4128	0.0000	0.0000	0.0144	0.0094	0.0084	0.0946
FRZB_E186_R	0.0000	0.0000	0.1279	0.0000	0.0000	0.0000	0.0000	0.0000	0.6707	0.1363	0.0000	0.0000	0.0000	0.9740	0.8986
FRZB_P406_F	0.0000	0.0000	0.0016	0.5784	0.0000	0.0000	0.0000	0.0000	0.0016	0.0051	0.9890	0.0033	0.0001	0.0000	0.0000
FVT1_P225_F	0.0091	0.0000	0.0205	0.0000	0.0085	0.3616	0.0107	0.0092	0.0104	0.0000	0.0043	0.0142	0.0107	0.0059	0.3665
FYN_P352_R	0.9947	0.9947	0.9936	0.9992	0.9930	0.9970	0.6616	0.9901	0.9852	0.9972	0.9941	0.9968	0.9965	0.9855	0.9923
FZD7_E296_F	0.0000	0.0000	0.9925	0.0000	0.0366	0.0000	0.0761	0.9768	0.9661	0.9921	0.9901	0.0000	0.0106	0.0000	0.9864
FZD9_E458_F	0.0000	0.0000	0.9913	0.0000	0.9897	0.0000	0.9921	0.9576	0.9116	0.0000	0.0000	0.0000	0.0000	0.0000	0.9734
FZD9_P15_R	0.0053	0.0000	0.0037	0.2192	0.0000	0.0018	0.0000	0.0000	0.0064	0.0119	0.0000	0.0068	0.0000	0.0007	0.0000
FZD9_P175_F	0.0157	0.1749	0.1974	0.0823	0.0069	0.1966	0.9861	0.3839	0.6022	0.0220	0.1453	0.1781	0.3498	0.0080	0.6410
G6PD_E190_F	0.0000	0.0039	0.0000	0.0000	0.0000	0.0000	0.0000	0.6745	0.0000	0.0000	0.0000	0.0000	0.0000	0.0020	0.3557
G6PD_P196_F	0.5340	0.5931	0.5550	0.8700	0.0089	0.7761	0.6143	0.4828	0.5826	0.0144	0.5121	0.0415	0.0140	0.5021	0.0000
GABRB3_E42_F	0.0055	0.0000	0.0000	0.0000	0.0000	0.0000	0.0054	0.0000	0.9648	0.0043	0.0000	0.0059	0.0038	0.0013	0.0000
GADD45A_P737_R	0.0266	0.0320	0.6443	0.9118	0.0153	0.5100	0.0173	0.5622	0.5931	0.0235	0.3269	0.0366	0.3665	0.0240	0.0004
GALR1_E52_F	0.0093	0.0000	0.0277	0.0000	0.0170	0.0000	0.2511	0.0253	0.0149	0.0102	0.0060	0.0069	0.1181	0.0139	0.0000
GALR1_P80_F	0.5504	0.1296	0.3976	0.0000	0.0089	0.0000	0.0093	0.0094	0.0122	0.0136	0.0014	0.0095	0.1548	0.0000	0.0000
GAS1_E22_F	0.0000	0.0826	0.2580	0.0000	0.1420	0.2774	0.0000	0.4144	0.0117	0.0000	0.0060	0.0109	0.0051	0.0056	0.0000
GAS1_P754_R	0.0000	0.0000	0.0000	0.0000	0.0000	0.0000	0.0050	0.0034	0.0015	0.0000	0.0020	0.0039	0.0038	0.0036	0.0000
GAS7_E148_F	0.0076	0.4877	0.0151	0.0000	0.3512	0.3345	0.2857	0.3957	0.5024	0.0162	0.0065	0.0141	0.0564	0.0124	0.0000
GAS7_P622_R	0.0000	0.0000	0.0000	0.0000	0.0000	0.0000	0.0031	0.0000	0.0000	0.0000	0.0000	0.0000	0.0000	0.0000	0.0000
GATA6_P21_R	0.0061	0.0045	0.0064	0.0000	0.0000	0.0000	0.0044	0.0069	0.1766	0.0044	0.0057	0.0000	0.0055	0.0047	0.0000
GATA6_P726_F	0.0090	0.0820	0.1839	0.0000	0.4387	0.1106	0.1163	0.4533	0.0350	0.2003	0.2059	0.0252	0.2166	0.0105	0.0000
GFI1_E136_F	0.1029	0.1408	0.2679	0.6061	0.2072	0.0000	0.2258	0.0963	0.0092	0.0000	0.0129	0.0226	0.0059	0.0129	0.0000
GFI1_P45_R	0.0012	0.7759	0.0052	0.0000	0.0018	0.0000	0.0000	0.0046	0.2110	0.0065	0.0053	0.0032	0.0000	0.0000	0.0000
GJB2_E43_F	0.0000	0.0000	0.0052	0.0000	0.0000	0.0000	0.0036	0.0149	0.0109	0.0000	0.0124	0.0052	0.0073	0.0000	0.0000
GJB2_P791_R	0.1447	0.2101	0.0122	0.0596	0.0065	0.1678	0.1192	0.0123	0.0102	0.0150	0.0108	0.0119	0.1623	0.0108	0.0102
GJB2_P931_R	0.3154	0.3423	0.0410	0.1211	0.0244	0.2928	0.3346	0.0449	0.0410	0.3172	0.0420	0.0743	0.0423	0.1108	0.0253
GLA_P112_F	0.0127	0.0047	0.1993	0.0000	0.0081	0.0000	0.0109	0.9004	0.0277	0.0157	0.1575	0.0133	0.0271	0.0122	0.6942
GLI2_E90_F	0.9441	0.9947	0.9906	0.0000	0.0000	0.0000	0.0000	0.9812	0.9829	0.0000	0.9889	0.9855	0.9880	0.9845	0.9654
GLI3_E148_R	0.8800	0.0679	0.9900	0.0940	0.0778	0.1344	0.0245	0.6854	0.8981	0.1279	0.9797	0.9864	0.7363	0.8743	0.9126
GML_P281_R	0.0000	0.0000	0.0000	0.0000	0.9382	0.0000	0.9942	0.7191	0.9821	0.0000	0.9914	0.9862	0.0000	0.5284	0.9843
GNAS_E58_F	0.0000	0.0000	0.0000	0.0000	0.0000	0.0000	0.0080	0.8241	0.7433	0.0000	0.0000	0.1961	0.0069	0.9841	0.8283
GP1BB_E23_F	0.0056	0.0085	0.0048	0.0000	0.0056	0.0764	0.0073	0.0091	0.4989	0.0000	0.0060	0.0098	0.0098	0.0244	0.0000
GPC3_P235_R	0.0093	0.1236	0.0005	0.7451	0.0582	0.0000	0.0000	0.0068	0.0056	0.0000	0.0000	0.0000	0.2350	0.4409	0.0000

GPR116_E328_R	0.9932	0.9172	0.9910	0.0000	0.9901	0.0000	0.9936	0.8701	0.9855	0.0000	0.0000	0.9896	0.9897	0.9902	0.9901
GPX1_E46_R	0.0113	0.0052	0.3372	0.0000	0.0000	0.0000	0.1781	0.1240	0.0089	0.0608	0.1632	0.0064	0.0049	0.0085	0.0000
GPX1_P194_F	0.0455	0.6473	0.7215	0.3761	0.0317	0.7538	0.0304	0.0226	0.0268	0.6734	0.0273	0.0361	0.0439	0.0452	0.0000
GPX3_E178_F	0.0100	0.0088	0.0084	0.0645	0.1571	0.0055	0.0092	0.2831	0.0128	0.0221	0.0075	0.0159	0.6127	0.0120	0.0615
GRB10_E85_R	0.0090	0.9746	0.0000	0.0000	0.0032	0.0000	0.1329	0.0065	0.6931	0.0000	0.0078	0.0061	0.0060	0.0052	0.0000
GRB10_P260_F	0.0000	0.0154	0.0000	0.0000	0.0042	0.1636	0.0000	0.0157	0.0040	0.0000	0.0053	0.0000	0.1029	0.0078	0.0000
GRB10_P496_R	0.1344	0.0000	0.0052	0.0000	0.0000	0.1170	0.0000	0.1315	0.0078	0.0076	0.1531	0.1418	0.1287	0.0031	0.0000
GRPR_P200_R	0.1858	0.9806	0.2160	0.5716	0.2003	0.1741	0.9802	0.8962	0.9562	0.2218	0.1749	0.2708	0.9455	0.9767	0.9318
GSTM1_P363_F	0.1449	0.0831	0.0051	0.0306	0.1880	0.1172	0.0093	0.9843	0.6486	0.0138	0.0096	0.6566	0.0872	0.9837	0.0000
GSTM2_E153_F	0.0000	0.0000	0.0062	0.0000	0.0007	0.0000	0.0000	0.0073	0.2512	0.0000	0.0050	0.0070	0.0000	0.0000	0.0396
GSTM2_P109_R	0.0000	0.0000	0.0000	0.0000	0.0014	0.0000	0.0000	0.0056	0.0039	0.0099	0.0047	0.0046	0.0000	0.0000	0.0000
GSTM2_P453_R	0.9911	0.0000	0.0021	0.0000	0.9902	0.0000	0.9920	0.0000	0.7480	0.0085	0.0000	0.6550	0.0072	0.0000	0.9804
GSTP1_E322_R	0.0000	0.0000	0.0003	0.0000	0.0000	0.4388	0.0000	0.2090	0.0089	0.2984	0.1866	0.0099	0.0982	0.0000	0.0000
GSTP1_P74_F	0.0287	0.3086	0.0479	0.0000	0.2347	0.6115	0.0231	0.0298	0.0613	0.7141	0.2526	0.4364	0.1584	0.0378	0.0000
GSTP1_seq_38_S153_R	0.0087	0.0000	0.0000	0.0000	0.0000	0.0000	0.0000	0.0065	0.0038	0.0000	0.0091	0.0000	0.9872	0.0005	0.0000
GUCY2D_E419_R	0.0038	0.0000	0.0053	0.0000	0.0019	0.0000	0.0071	0.0109	0.0069	0.0000	0.0000	0.0063	0.0054	0.0000	0.0000
HBEGF_P32_R	0.0065	0.0000	0.0028	0.0000	0.0000	0.0000	0.0000	0.0045	0.0000	0.0070	0.0076	0.0043	0.0000	0.0018	0.0000
HBII-13_E48_F	0.0000	0.0000	0.0000	0.4333	0.0000	0.9942	0.0000	0.9852	0.9790	0.0000	0.0000	0.9848	0.9831	0.9784	0.0000
HBII-52_P563_F	0.9871	0.5949	0.8458	0.0987	0.9934	0.6508	0.6529	0.7172	0.7808	0.9945	0.5755	0.0427	0.6219	0.5960	0.9326
HCK_P46_R	0.1899	0.1637	0.0113	0.0000	0.2968	0.0000	0.0064	0.0160	0.0142	0.0145	0.0065	0.0000	0.0000	0.0072	0.0000
HCK_P858_F	0.0907	0.5830	0.0365	0.2427	0.6229	0.5047	0.0411	0.0190	0.0161	0.5794	0.0270	0.0483	0.0381	0.0299	0.0000
HDAC1_P414_R	0.0000	0.0149	0.0067	0.0000	0.0068	0.2000	0.0076	0.5569	0.2985	0.0074	0.0073	0.6814	0.0000	0.0072	0.0000
HDAC11_P556_F	0.0000	0.0113	0.0000	0.0000	0.0000	0.0000	0.0000	0.0016	0.0000	0.0000	0.0050	0.0000	0.0059	0.0007	0.0000
HDAC9_P137_R	0.0000	0.0030	0.0006	0.0000	0.0031	0.0000	0.0000	0.2100	0.0029	0.0046	0.7257	0.0034	0.0024	0.4259	0.0000
HFE_E273_R	0.0054	0.0000	0.0015	0.0000	0.0086	0.0000	0.0117	0.0057	0.0042	0.0000	0.0076	0.0703	0.0000	0.0008	0.0000
HHIP_P307_R	0.1344	0.1103	0.0206	0.0120	0.0119	0.1150	0.0136	0.0202	0.0230	0.0205	0.1742	0.1495	0.1338	0.0291	0.0081
HIC1_E151_F	0.0000	0.0000	0.0003	0.0000	0.0000	0.0042	0.0000	0.0000	0.0000	0.0000	0.0000	0.0000	0.0000	0.0000	0.0000
HIC1_P565_R	0.0000	0.0000	0.0000	0.0000	0.0098	0.0000	0.0000	0.0000	0.0225	0.0000	0.0000	0.0052	0.0000	0.0223	0.0000
HIC2_P498_F	0.0000	0.0000	0.0076	0.0000	0.0033	0.0000	0.0000	0.0074	0.0045	0.0000	0.0047	0.0000	0.0063	0.0047	0.0000
HIC2_P528_R	0.0000	0.0000	0.0000	0.0000	0.0067	0.0000	0.0000	0.0105	0.0080	0.0000	0.0073	0.0000	0.0083	0.0043	0.0000
HIF1A_P488_F	0.0102	0.0000	0.0000	0.0000	0.0000	0.0000	0.0000	0.0000	0.0000	0.0000	0.0020	0.0127	0.0000	0.0006	0.0000
HLA-DOB_P357_R	0.9852	0.9910	0.0000	0.0000	0.9729	0.0000	0.0000	0.9856	0.9824	0.0085	0.0000	0.9781	0.0000	0.9761	0.9720
HLA-DPA1_P205_R	0.0067	0.1003	0.0076	0.0000	0.0063	0.0911	0.1280	0.0118	0.4729	0.1059	0.1075	0.0115	0.1449	0.0103	0.0000

HLA-DPA1_P28_R	0.0073	0.0000	0.0000	0.0000	0.0029	0.0000	0.0084	0.0066	0.0098	0.0000	0.0000	0.0039	0.0000	0.6945	0.2826
HLA-DPB1_E2_R	0.0000	0.0000	0.0670	0.0000	0.0315	0.0000	0.5606	0.1012	0.5298	0.0000	0.0465	0.1285	0.4863	0.0135	0.0000
HLA-DRA_P132_R	0.0000	0.0000	0.0000	0.0000	0.0000	0.0036	0.0000	0.0000	0.0000	0.0034	0.0000	0.0000	0.0045	0.0000	0.0000
HLA-F_E402_F	0.0000	0.7293	0.0000	0.0000	0.0000	0.0002	0.0000	0.0000	0.0000	0.0000	0.0032	0.0000	0.0000	0.0000	0.0000
HLF_E192_F	0.0435	0.0335	0.7724	0.4426	0.7845	0.6481	0.6712	0.0260	0.0165	0.0377	0.0228	0.0510	0.6405	0.0168	0.0065
HOXA11_E35_F	0.0100	0.0000	0.0024	0.0388	0.0000	0.0000	0.0000	0.0049	0.0048	0.0055	0.0031	0.0020	0.0035	0.0022	0.0000
HOXA11_P698_F	0.0252	0.6252	0.0220	0.9147	0.5669	0.7652	0.5501	0.0261	0.0198	0.5405	0.0348	0.0460	0.0278	0.0405	0.0000
HOXA5_E187_F	0.0000	0.0000	0.0000	0.0000	0.9901	0.0000	0.0000	0.0000	0.9822	0.0000	0.0017	0.3238	0.9909	0.9788	0.9568
HOXA9_E252_R	0.0000	0.1106	0.1606	0.0000	0.0000	0.0087	0.1146	0.0100	0.0060	0.0125	0.0086	0.0000	0.0137	0.0083	0.0000
HOXA9_P1141_R	0.0172	0.1884	0.0183	0.0000	0.1954	0.2285	0.1887	0.0137	0.0211	0.0152	0.0142	0.0184	0.5280	0.0298	0.0000
HOXA9_P303_F	0.0000	0.0028	0.0000	0.0000	0.0000	0.0000	0.0000	0.0021	0.0006	0.0017	0.0000	0.0005	0.0000	0.0000	0.0000
HOXB13_E21_F	0.0063	0.0000	0.0054	0.0000	0.0028	0.0073	0.0000	0.0028	0.0021	0.0000	0.0035	0.0064	0.0034	0.0020	0.1196
HOXB13_P17_R	0.0039	0.0000	0.0000	0.0000	0.0000	0.0026	0.0000	0.0039	0.0011	0.0000	0.0026	0.0044	0.0025	0.0032	0.0000
HOXC6_P456_R	0.0375	0.9718	0.0247	0.9139	0.0259	0.7402	0.6371	0.0359	0.0365	0.0422	0.0650	0.7516	0.0813	0.0306	0.0938
HPN_P374_R	0.0119	0.0113	0.0000	0.0000	0.0047	0.0000	0.0000	0.0007	0.0102	0.0000	0.0000	0.0861	0.0086	0.0015	0.0000
HPN_P823_F	0.0000	0.0000	0.0000	0.0000	0.0000	0.0000	0.6695	0.0000	0.0000	0.0000	0.0000	0.0000	0.0026	0.7725	0.0000
HPSE_P29_F	0.5322	0.6704	0.7175	0.9324	0.7293	0.6749	0.0214	0.0306	0.0232	0.0310	0.0279	0.0362	0.0342	0.5741	0.0031
HPSE_P93_F	0.0031	0.0064	0.0000	0.0000	0.0032	0.0000	0.0000	0.0037	0.3064	0.0075	0.0048	0.0014	0.0061	0.0023	0.0000
HRASLS_E72_R	0.0000	0.0000	0.0018	0.0000	0.0000	0.0000	0.0000	0.0000	0.0000	0.0000	0.0000	0.0000	0.0011	0.0000	0.0000
HS3ST2_E145_R	0.0012	0.0000	0.0000	0.0000	0.0000	0.0000	0.0000	0.0025	0.0000	0.0000	0.0000	0.4683	0.0000	0.0002	0.0000
HS3ST2_P546_F	0.0250	0.0098	0.0025	0.0000	0.0023	0.0000	0.0000	0.0115	0.3832	0.0084	0.0000	0.0069	0.0000	0.0111	0.0000
HSD17B12_E145_R	0.4093	0.3856	0.0124	0.0000	0.6528	0.0000	0.3879	0.5212	0.0283	0.0129	0.0000	0.0119	0.0297	0.0222	0.0000
HTR1B_P222_F	0.0000	0.0505	0.0066	0.0000	0.0000	0.2110	0.0033	0.0046	0.0009	0.0098	0.0017	0.0000	0.0000	0.0001	0.0000
HTR2A_P853_F	0.0000	0.0036	0.0022	0.0000	0.0000	0.0060	0.0000	0.0057	0.0039	0.0000	0.0000	0.1069	0.0000	0.0014	0.0000
IAPP_E280_F	0.0000	0.0000	0.0000	0.0000	0.9924	0.9963	0.9946	0.9805	0.9845	0.0000	0.0000	0.0000	0.9882	0.9768	0.9379
ICA1_P61_F	0.0000	0.0000	0.0000	0.0000	0.0000	0.0000	0.0000	0.0000	0.0000	0.0000	0.0000	0.0000	0.0000	0.0000	0.0000
ICA1_P72_R	0.0071	0.0000	0.0000	0.1762	0.0000	0.0000	0.0066	0.0047	0.0000	0.0000	0.0028	0.0000	0.0000	0.0014	0.0000
ICAM1_E242_F	0.0138	0.0116	0.0099	0.4493	0.0032	0.0046	0.2972	0.4827	0.0099	0.0081	0.0093	0.4891	0.2774	0.0126	0.0000
ICAM1_P119_R	0.0059	0.0000	0.0056	0.0000	0.0000	0.0000	0.0000	0.0057	0.0092	0.0062	0.0046	0.0000	0.0000	0.0024	0.0000
ICAM1_P386_R	0.0000	0.0000	0.0000	0.0000	0.0000	0.0000	0.0000	0.0000	0.0000	0.0000	0.0049	0.0051	0.6517	0.0000	0.0000
ID1_P659_R	0.0000	0.0000	0.0000	0.0000	0.0000	0.0000	0.0000	0.0044	0.0049	0.0000	0.0027	0.0000	0.0039	0.0079	0.0000
IFNG_P459_R	0.3215	0.2301	0.7399	0.0173	0.9898	0.2253	0.6618	0.8665	0.9856	0.0107	0.2815	0.6098	0.9779	0.7085	0.8633
IFNGR1_P307_F	0.1248	0.1123	0.0080	0.7521	0.0054	0.0056	0.1520	0.1751	0.3752	0.1301	0.0136	0.0121	0.0079	0.0110	0.0000

IFNGR2_E164_F	0.0276	0.0368	0.0685	0.0000	0.0156	0.1498	0.1576	0.0248	0.0261	0.0150	0.0175	0.0206	0.0353	0.0288	0.0238
IFNGR2_P377_R	0.8546	0.0040	0.0000	0.4974	0.0000	0.0000	0.0000	0.0010	0.0000	0.0050	0.9229	0.0000	0.0036	0.0000	0.6835
IGF1_E394_F	0.0000	0.0000	0.9907	0.0000	0.9917	0.0000	0.9934	0.8983	0.8554	0.0000	0.0000	0.0000	0.8051	0.9810	0.9360
IGF1_P933_F	0.1375	0.0074	0.0548	0.0000	0.0037	0.0000	0.9913	0.9417	0.9705	0.0000	0.9902	0.8116	0.9891	0.9850	0.2859
IGF1R_E186_R	0.0013	0.0000	0.0046	0.0000	0.0000	0.0000	0.0052	0.0047	0.0057	0.0000	0.0041	0.0000	0.0000	0.0000	0.0000
IGF1R_P325_R	0.3409	0.4804	0.0072	0.6081	0.0189	0.2494	0.4347	0.3545	0.0227	0.4797	0.3374	0.4909	0.0151	0.0451	0.0000
IGF2_E134_R	0.0000	0.0000	0.0000	0.0000	0.0000	0.0000	0.0049	0.0000	0.0000	0.0008	0.0000	0.0000	0.0062	0.0000	0.0000
IGF2_P1036_R	0.1121	0.0089	0.0112	0.0000	0.0072	0.0058	0.1405	0.0151	0.0221	0.0397	0.0128	0.0152	0.0188	0.0187	0.0000
IGF2_P36_R	0.7861	0.0092	0.3004	0.0000	0.4127	0.4919	0.0841	0.0124	0.0191	0.3382	0.0133	0.2367	0.0568	0.1442	0.9702
IGF2AS_P203_F	0.0000	0.0000	0.0000	0.0000	0.0000	0.0000	0.0000	0.0000	0.0000	0.0000	0.0000	0.0000	0.0000	0.0000	0.0000
IGF2R_P396_R	0.8544	0.9567	0.2613	0.8701	0.9784	0.9454	0.4110	0.1221	0.2329	0.3981	0.4140	0.3388	0.9396	0.2336	0.1572
IGFBP1_E48_R	0.0045	0.0085	0.0048	0.0000	0.1417	0.4013	0.0604	0.0053	0.0083	0.0196	0.0509	0.0053	0.0104	0.0076	0.0000
IGFBP1_P12_R	0.3079	0.0202	0.0000	0.8048	0.1433	0.4285	0.2409	0.0134	0.0096	0.0102	0.0063	0.1502	0.0788	0.0038	0.0000
IGFBP2_P306_F	0.0001	0.0133	0.0022	0.0028	0.0000	0.0059	0.0000	0.0000	0.0000	0.0044	0.0023	0.0005	0.0021	0.0000	0.0000
IGFBP3_E65_R	0.0104	0.0122	0.0133	0.0000	0.0082	0.6581	0.0130	0.0182	0.0186	0.1795	0.5724	0.0179	0.1678	0.0247	0.0000
IGFBP3_P1035_F	0.0000	0.0039	0.0000	0.0034	0.0000	0.0000	0.0052	0.0018	0.0009	0.0000	0.0025	0.0016	0.0038	0.0000	0.0000
IGFBP3_P423_R	0.0928	0.0043	0.1250	0.0000	0.0038	0.1372	0.0787	0.0101	0.0117	0.1043	0.0048	0.0103	0.0088	0.0089	0.0000
IGFBP7_P371_F	0.1339	0.0061	0.0645	0.0405	0.0051	0.5104	0.2391	0.0150	0.0092	0.0000	0.0091	0.0072	0.1669	0.0186	0.0000
IGSF4_P454_F	0.0086	0.0000	0.2658	0.0000	0.0000	0.3829	0.0041	0.0081	0.0076	0.0082	0.0095	0.0075	0.0054	0.0208	0.0000
IGSF4_P86_R	0.0324	0.2650	0.0141	0.0000	0.0129	0.1907	0.0108	0.0446	0.0146	0.0640	0.0100	0.0147	0.5166	0.0208	0.0000
IGSF4C_E65_F	0.9933	0.0000	0.0000	0.0000	0.4736	0.0000	0.9810	0.6286	0.9471	0.0000	0.8314	0.0000	0.0038	0.9874	0.8157
IGSF4C_P533_R	0.0075	0.0065	0.9903	0.0000	0.0097	0.0000	0.0000	0.0073	0.0097	0.0103	0.0000	0.0106	0.0067	0.0065	0.0000
IHH_E186_F	0.0000	0.0000	0.0002	0.0000	0.0000	0.1361	0.0000	0.0000	0.0029	0.0000	0.0000	0.0024	0.0000	0.0035	0.0000
IHH_P246_R	0.0145	0.0000	0.0269	0.0000	0.9894	0.0000	0.0102	0.0000	0.0734	0.0070	0.0000	0.0000	0.9887	0.0104	0.0000
IHH_P529_F	0.0000	0.0000	0.0000	0.0000	0.0000	0.0000	0.0000	0.0000	0.0133	0.0114	0.0054	0.0070	0.0177	0.0107	0.0000
IL10_P85_F	0.8460	0.0000	0.0000	0.0000	0.0000	0.0000	0.0058	0.0043	0.5212	0.0045	0.0040	0.0000	0.0000	0.4126	0.0000
IL11_P11_R	0.0106	0.0000	0.0129	0.0000	0.0000	0.0000	0.0000	0.0040	0.0103	0.0099	0.0106	0.0000	0.0000	0.0120	0.0000
IL12A_E287_R	0.0030	0.0002	0.0032	0.0000	0.0016	0.0049	0.0040	0.0027	0.0029	0.0033	0.0000	0.0039	0.0000	0.0014	0.0000
IL12B_P392_R	0.0289	0.0062	0.0015	0.0018	0.0014	0.0064	0.0047	0.2192	0.0867	0.0033	0.2074	0.0013	0.0016	0.3805	0.0610
IL13_E75_R	0.7336	0.9950	0.8373	0.3665	0.0862	0.8123	0.6684	0.7963	0.9770	0.7355	0.0636	0.9247	0.9927	0.9845	0.9824
IL17RB_E164_R	0.0001	0.0000	0.0084	0.0000	0.0000	0.0000	0.0000	0.0046	0.0000	0.0000	0.0120	0.0061	0.0066	0.1109	0.0000
IL17RB_P788_R	0.0000	0.0000	0.0000	0.0000	0.0000	0.0000	0.0000	0.0000	0.0000	0.0000	0.0000	0.0000	0.0000	0.0000	0.0000
IL18BP_P51_R	0.0399	0.0191	0.2889	0.1761	0.0201	0.2941	0.2704	0.0251	0.0272	0.2457	0.0274	0.1060	0.2462	0.0458	0.0018

IL1A_E113_R	0.0096	0.0786	0.9914	0.5219	0.9910	0.9957	0.2081	0.9909	0.9850	0.1160	0.2713	0.9867	0.8079	0.9873	0.9906
IL1RN_E42_F	0.9898	0.0000	0.0000	0.0000	0.9860	0.9953	0.0000	0.9841	0.9442	0.0000	0.6661	0.9766	0.9901	0.9895	0.9788
IL3_P556_F	0.8770	0.0000	0.0000	0.0000	0.0000	0.9962	0.0000	0.9870	0.9758	0.9933	0.9916	0.8853	0.9897	0.2989	0.9824
IL6_E168_F	0.0751	0.0783	0.1117	0.6638	0.0000	0.3423	0.0241	0.6075	0.0104	0.0153	0.0110	0.0213	0.1341	0.5016	0.0000
IL8_E118_R	0.0000	0.0000	0.0000	0.0000	0.0000	0.0000	0.0000	0.0047	0.0000	0.0000	0.0053	0.0056	0.0000	0.0000	0.7561
IL8_P83_F	0.0000	0.0000	0.0000	0.0000	0.0000	0.0000	0.0000	0.0000	0.8733	0.0000	0.0000	0.0000	0.0000	0.0000	0.0000
IMPACT_P186_F	0.0000	0.0000	0.0000	0.0000	0.0000	0.0000	0.0000	0.0007	0.0000	0.0000	0.0000	0.0000	0.0000	0.0000	0.0000
IMPACT_P234_R	0.0000	0.0000	0.0000	0.0000	0.0000	0.0000	0.0046	0.0054	0.0025	0.0012	0.0000	0.0000	0.0011	0.0000	0.0000
INSR_P1063_R	0.0000	0.0000	0.0068	0.0000	0.0000	0.0000	0.0000	0.0000	0.0010	0.0000	0.0000	0.0000	0.0000	0.0000	0.0000
IPF1_P234_F	0.0000	0.9908	0.0000	0.0000	0.0000	0.0000	0.0000	0.0035	0.0089	0.0000	0.0000	0.0064	0.0020	0.0042	0.0000
IRAK1_P455_R	0.0068	0.0054	0.0042	0.0000	0.0651	0.0000	0.0253	0.1051	0.0106	0.0054	0.0023	0.0141	0.5318	0.0092	0.3849
IRAK3_E130_F	0.0123	0.0000	0.0086	0.0000	0.0103	0.0000	0.4078	0.0101	0.0169	0.0086	0.0100	0.0101	0.0061	0.0226	0.0000
IRAK3_P13_F	0.0104	0.0076	0.2489	0.0000	0.0000	0.0424	0.0000	0.0000	0.0097	0.0000	0.0000	0.0137	0.0052	0.0020	0.0000
IRF5_P123_F	0.0156	0.0114	0.0164	0.0000	0.0472	0.0025	0.0030	0.0119	0.0116	0.0121	0.0056	0.6649	0.0118	0.0085	0.0000
IRF7_P277_R	0.5856	0.0675	0.0179	0.1016	0.0093	0.2261	0.1778	0.0166	0.0167	0.0197	0.0128	0.0712	0.0227	0.0144	0.0055
ISL1_E87_R	0.0071	0.0000	0.0036	0.0000	0.0000	0.0000	0.0075	0.0106	0.0060	0.0000	0.0056	0.0000	0.0021	0.0034	0.0000
ISL1_P379_F	0.0042	0.0060	0.0049	0.0000	0.3435	0.0056	0.0116	0.0111	0.0041	0.0000	0.0000	0.0000	0.0000	0.0025	0.0000
ISL1_P554_F	0.0000	0.0027	0.0001	0.0000	0.0000	0.0000	0.0000	0.0000	0.0000	0.0000	0.0000	0.0000	0.0000	0.0000	0.0000
ITGA2_E120_F	0.0069	0.0000	0.0059	0.0000	0.0042	0.3232	0.0072	0.0178	0.0100	0.0090	0.0070	0.0242	0.0076	0.0088	0.0000
ITGA2_P26_R	0.0000	0.0000	0.0000	0.0000	0.0046	0.0000	0.0000	0.0024	0.0003	0.0000	0.0051	0.0000	0.0055	0.0000	0.0000
ITGB4_E144_F	0.0326	0.0092	0.0106	0.0000	0.0000	0.0000	0.0000	0.0096	0.0064	0.0116	0.0000	0.0098	0.0072	0.0010	0.0000
ITGB4_P517_F	0.0000	0.0000	0.0000	0.0000	0.0000	0.0000	0.0000	0.0000	0.5730	0.0000	0.0000	0.4839	0.0000	0.0000	0.0000
ITPR3_E86_R	0.0041	0.0078	0.0000	0.0000	0.0000	0.0035	0.0000	0.0000	0.0000	0.0000	0.0000	0.0052	0.0032	0.0000	0.0000
ITPR3_P1112_F	0.0048	0.0029	0.0000	0.0000	0.0015	0.0000	0.0063	0.0000	0.0002	0.0055	0.0000	0.0051	0.0013	0.0016	0.5554
JAG2_E54_F	0.0063	0.0000	0.0044	0.0000	0.0058	0.0000	0.0714	0.0089	0.0086	0.0000	0.0131	0.0290	0.0056	0.0043	0.0000
JAG2_P264_F	0.3990	0.0000	0.0745	0.0000	0.5653	0.0000	0.2550	0.3992	0.0183	0.1812	0.0107	0.3580	0.1607	0.2260	0.0000
JAK2_P772_R	0.0172	0.2162	0.0117	0.0000	0.2192	0.2150	0.0140	0.0213	0.0175	0.0155	0.1287	0.0140	0.0936	0.1841	0.0000
JAK3_E64_F	0.0000	0.0000	0.0082	0.0000	0.0064	0.0000	0.0000	0.0071	0.0079	0.0000	0.0066	0.0064	0.0000	0.0516	0.0000
JAK3_P156_R	0.1330	0.0737	0.0102	0.0000	0.3215	0.1568	0.1317	0.0114	0.0132	0.0157	0.0084	0.3173	0.0128	0.0113	0.0000
JUNB_P1149_R	0.9739	0.1147	0.0955	0.4068	0.5221	0.5069	0.6106	0.2396	0.0744	0.1367	0.0703	0.6646	0.1045	0.0786	0.1043
KCNK4_E3_F	0.0201	0.0047	0.0035	0.0000	0.0000	0.0000	0.0038	0.0163	0.0060	0.9109	0.0017	0.0058	0.0066	0.9443	0.0000
KDR_E79_F	0.8027	0.8160	0.7832	0.5954	0.7768	0.7895	0.7874	0.0352	0.0212	0.7580	0.0183	0.0226	0.0286	0.0322	0.0012
KDR_P445_R	0.0103	0.0107	0.0071	0.0000	0.0056	0.0000	0.0000	0.0099	0.0029	0.0079	0.0000	0.0000	0.0140	0.0059	0.0000

KIAA1804_P689_R	0.1131	0.1127	0.0080	0.0000	0.3394	0.0961	0.1100	0.0079	0.0114	0.0846	0.1413	0.1065	0.0073	0.3031	0.0000
KIT_P367_R	0.0057	0.0000	0.2100	0.0293	0.0000	0.0000	0.0012	0.0000	0.0075	0.0000	0.0057	0.0046	0.0000	0.0038	0.0000
KIT_P405_F	0.5997	0.0049	0.0038	0.0000	0.0000	0.0000	0.0062	0.1358	0.2316	0.0094	0.0082	0.0086	0.6727	0.0094	0.0000
KLF5_E190_R	0.0000	0.0000	0.0027	0.0000	0.0007	0.0024	0.0000	0.0046	0.0033	0.0000	0.0047	0.0034	0.0000	0.0025	0.0000
KLK11_P103_R	0.0000	0.0000	0.9161	0.0000	0.0000	0.0000	0.0000	0.0003	0.9740	0.0000	0.0000	0.9868	0.0000	0.9797	0.7525
KLK11_P1290_F	0.0066	0.0000	0.0000	0.0000	0.9893	0.0000	0.9875	0.0079	0.9540	0.0000	0.0000	0.9912	0.0000	0.9797	0.9699
KRAS_E82_F	0.0082	0.0039	0.4566	0.0000	0.0000	0.0000	0.4987	0.8287	0.4765	0.0000	0.0113	0.0154	0.0625	0.5821	0.4866
KRAS_P651_F	0.4254	0.1413	0.1281	0.1631	0.3892	0.4415	0.0946	0.0904	0.1756	0.1975	0.4459	0.2091	0.5116	0.3502	0.1582
KRT13_P341_R	0.9934	0.0000	0.0000	0.0000	0.9902	0.0000	0.9912	0.8958	0.9844	0.0000	0.9911	0.0071	0.0000	0.8710	0.9319
KRT5_P308_F	0.1569	0.1957	0.3226	0.7372	0.9622	0.2523	0.9456	0.2370	0.8563	0.2592	0.2440	0.9354	0.2066	0.2648	0.6496
L1CAM_P148_R	0.0000	0.0070	0.0000	0.3780	0.0425	0.0000	0.0149	0.0069	0.0034	0.0075	0.0000	0.0088	0.0090	0.0026	0.0000
L1CAM_P19_F	0.0000	0.0000	0.0026	0.0000	0.0000	0.0000	0.0000	0.6866	0.0064	0.0000	0.0342	0.0000	0.0000	0.0030	0.5332
LAMC1_E466_R	0.0000	0.0068	0.0045	0.0000	0.0041	0.0000	0.0092	0.0052	0.0028	0.0000	0.0345	0.0094	0.0155	0.0013	0.0000
LIF_E208_F	0.0126	0.2736	0.0089	0.0000	0.2888	0.3124	0.3374	0.2569	0.0091	0.1741	0.2214	0.0091	0.0289	0.0107	0.0000
LIF_P383_R	0.0000	0.0000	0.0000	0.0000	0.0000	0.0000	0.2163	0.0000	0.6447	0.0060	0.0000	0.0000	0.6279	0.0189	0.1926
LIG4_P194_F	0.0095	0.0000	0.0001	0.0000	0.0087	0.0000	0.0572	0.0000	0.0132	0.0000	0.0000	0.0000	0.0088	0.0115	0.0000
LMO1_E265_R	0.0029	0.0000	0.0007	0.0000	0.0056	0.0037	0.0000	0.0020	0.0040	0.0000	0.0000	0.0005	0.0000	0.5726	0.0000
LMO1_P169_F	0.0000	0.0000	0.0000	0.0000	0.0040	0.0000	0.0057	0.0098	0.0069	0.0000	0.0000	0.0000	0.0051	0.0067	0.0000
LOX_P313_R	0.0256	0.0597	0.2060	0.0123	0.0119	0.1463	0.0667	0.0155	0.2105	0.0524	0.0963	0.0243	0.1295	0.0252	0.0000
LOX_P71_F	0.0049	0.0000	0.0038	0.0000	0.0053	0.0000	0.0024	0.0071	0.0047	0.0070	0.0000	0.0000	0.0047	0.0000	0.0000
LRP2_E20_F	0.0407	0.5413	0.0351	0.9652	0.0677	0.6900	0.0512	0.0500	0.0348	0.0385	0.0288	0.0352	0.4922	0.0304	0.0047
LRRC32_P865_R	0.0086	0.0000	0.0000	0.6420	0.0055	0.0000	0.0000	0.0082	0.0132	0.0000	0.0071	0.0000	0.0000	0.0214	0.0000
LTB4R_E64_R	0.0337	0.0790	0.6020	0.0000	0.9843	0.4647	0.0226	0.0307	0.0311	0.0290	0.4797	0.0961	0.0408	0.5620	0.0375
LYN_E353_F	0.0000	0.0062	0.0000	0.0000	0.0000	0.0000	0.0000	0.0000	0.0000	0.0000	0.0012	0.9791	0.0028	0.0000	0.0000
LYN_P241_F	0.2923	0.3963	0.0383	0.0920	0.0437	0.3017	0.2993	0.0784	0.0399	0.0585	0.2572	0.0532	0.0646	0.0464	0.0257
MAF_P826_R	0.0000	0.0000	0.0000	0.0000	0.0000	0.0000	0.0000	0.0000	0.0000	0.0000	0.0000	0.0020	0.0102	0.0000	0.0000
MAGEC3_P903_F	0.0000	0.0599	0.0000	0.0000	0.0000	0.2323	0.0000	0.0000	0.0035	0.0000	0.0000	0.0000	0.0080	0.0000	0.0000
MAGEL2_E166_R	0.0000	0.0000	0.0000	0.0136	0.0000	0.0000	0.0000	0.0000	0.9781	0.0000	0.0000	0.0000	0.0000	0.9589	0.0000
MALT1_P406_R	0.0000	0.0038	0.0025	0.0000	0.0047	0.0000	0.0058	0.0046	0.0076	0.0000	0.0069	0.0141	0.0000	0.0000	0.0000
MAP2K6_E297_F	0.0000	0.0012	0.0015	0.0000	0.0000	0.0056	0.0057	0.0001	0.0010	0.0000	0.0052	0.0021	0.0007	0.7481	0.0000
MAP2K6_P297_R	0.0201	0.1155	0.0103	0.0000	0.2861	0.1346	0.0121	0.0122	0.0132	0.1312	0.0116	0.0151	0.0125	0.0088	0.0000
MAP3K1_P7_F	0.2954	0.2272	0.1791	0.4734	0.7477	0.0055	0.3581	0.3600	0.0335	0.0130	0.0314	0.0249	0.6227	0.0245	0.0000
MAP3K9_E17_R	0.3613	0.0000	0.0000	0.0000	0.2383	0.0000	0.0000	0.0171	0.0293	0.0000	0.1549	0.0000	0.0029	0.0265	0.0000

MAPK12_E165_R	0.1108	0.1256	0.0000	0.0000	0.0034	0.3776	0.0000	0.0108	0.0086	0.0123	0.0057	0.0089	0.0090	0.0068	0.0000
MAPK12_P416_F	0.0000	0.0000	0.0000	0.0000	0.0000	0.0000	0.0050	0.0029	0.0000	0.0000	0.0000	0.0059	0.0033	0.0004	0.0000
MAPK14_P327_R	0.0000	0.0887	0.0010	0.0000	0.0053	0.0000	0.0099	0.0297	0.0044	0.0049	0.0000	0.2309	0.1855	0.0064	0.0000
MAPK4_E273_R	0.0000	0.0000	0.0031	0.0000	0.0000	0.9965	0.0000	0.0000	0.9819	0.0000	0.9940	0.0013	0.0026	0.5651	0.8260
MAPK9_P1175_F	0.0000	0.0000	0.9933	0.0000	0.0000	0.0000	0.0000	0.8493	0.9805	0.0000	0.0443	0.0000	0.0000	0.9810	0.9722
MAS1_P469_R	0.8212	0.3808	0.6694	0.1516	0.9889	0.9790	0.1479	0.9889	0.9825	0.6081	0.9926	0.6680	0.0208	0.9851	0.9785
MATK_P64_F	0.0073	0.0701	0.0689	0.0678	0.0051	0.0237	0.0059	0.0096	0.0097	0.0083	0.0086	0.0462	0.0530	0.0034	0.0000
MC2R_P1025_F	0.0000	0.0000	0.0000	0.0000	0.0000	0.0000	0.6729	0.0000	0.9555	0.0000	0.0000	0.0000	0.0000	0.0000	0.0000
MCAM_P169_R	0.0104	0.0045	0.0000	0.0000	0.0051	0.0000	0.0000	0.0123	0.0061	0.0238	0.0056	0.0000	0.0040	0.0071	0.0000
MCAM_P265_R	0.0328	0.0662	0.1447	0.0000	0.0083	0.0519	0.0438	0.6540	0.0203	0.0104	0.0089	0.0214	0.0293	0.0290	0.5526
MCC_P196_R	0.0000	0.0000	0.0000	0.0000	0.0000	0.0000	0.0000	0.0000	0.0000	0.0000	0.0015	0.0000	0.0035	0.0000	0.0000
MCM2_P241_R	0.1870	0.0094	0.0077	0.0761	0.0079	0.4389	0.0111	0.0091	0.0087	0.0135	0.0101	0.0095	0.0114	0.0047	0.0000
MDR1_seq_42_S300_R	0.0118	0.0066	0.0099	0.0000	0.0060	0.0406	0.0073	0.0107	0.0054	0.0095	0.0097	0.0086	0.0074	0.0088	0.0000
MDS1_E45_F	0.0056	0.0000	0.0055	0.0000	0.0000	0.0000	0.3079	0.0125	0.0201	0.0000	0.2448	0.0094	0.0371	0.0042	0.0000
MECP2_E90_R	0.0067	0.0094	0.6858	0.0000	0.0042	0.0064	0.0054	0.0120	0.0083	0.0125	0.0033	0.0093	0.0065	0.2039	0.0000
MEG3_E91_F	0.0065	0.0000	0.9889	0.3898	0.0063	0.0000	0.0217	0.0000	0.0032	0.0000	0.0000	0.0000	0.0028	0.0000	0.0000
MEST_E150_F	0.0003	0.0000	0.0011	0.0000	0.0019	0.0000	0.0004	0.0044	0.0000	0.0054	0.0000	0.0024	0.0000	0.0029	0.0000
MEST_P4_F	0.0000	0.0000	0.0081	0.0000	0.0008	0.0000	0.0013	0.0039	0.0037	0.0029	0.0000	0.0014	0.0032	0.0005	0.0000
MEST_P62_R	0.0001	0.0000	0.0000	0.0000	0.0000	0.0112	0.5115	0.0062	0.0000	0.0000	0.0000	0.0094	0.0040	0.0009	0.0000
MET_E333_F	0.0352	0.0000	0.5871	0.6686	0.0000	0.0000	0.9924	0.5071	0.0147	0.0146	0.0126	0.0142	0.3287	0.0242	0.0000
MFAP4_P197_F	0.0028	0.0000	0.0039	0.0000	0.0000	0.0000	0.0000	0.0035	0.0007	0.0000	0.0063	0.0000	0.0049	0.0000	0.0000
MGMT_P272_R	0.0000	0.0000	0.0036	0.0000	0.0000	0.0000	0.0000	0.0000	0.0036	0.0000	0.0054	0.0042	0.0000	0.0000	0.0000
MGMT_P281_F	0.0104	0.5668	0.0131	0.8867	0.4812	0.7048	0.4919	0.0118	0.0179	0.0179	0.0150	0.0193	0.0144	0.0266	0.0000
MKRN3_P108_F	0.0000	0.9927	0.0000	0.0000	0.9879	0.0000	0.0219	0.3858	0.5242	0.0000	0.9836	0.0000	0.0000	0.9693	0.9822
MLF1_P97_F	0.0095	0.0000	0.0053	0.0287	0.1465	0.0050	0.0000	0.0076	0.0091	0.0113	0.0000	0.0097	0.0067	0.0050	0.0000
MLH1_P381_F	0.3441	0.2120	0.3637	0.0000	0.3367	0.0000	0.0105	0.0084	0.0119	0.0000	0.3587	0.0090	0.0081	0.1298	0.0000
MLH3_E72_F	0.0000	0.1112	0.0040	0.0000	0.0000	0.0000	0.0067	0.0054	0.0060	0.0000	0.0034	0.0000	0.0000	0.0002	0.0000
MLH3_P25_F	0.3636	0.0000	0.7411	0.0000	0.6454	0.0000	0.0000	0.2053	0.0060	0.0028	0.7923	0.0034	0.0029	0.4638	0.0818
MLLT3_E93_R	0.0048	0.0000	0.0000	0.0000	0.0000	0.0000	0.0044	0.0000	0.0000	0.0000	0.0088	0.0071	0.0000	0.0000	0.0000
MLLT4_P1400_F	0.1332	0.1987	0.0118	0.8161	0.0135	0.1902	0.2125	0.0184	0.0192	0.0159	0.1624	0.0210	0.1582	0.0182	0.0000
MLLT6_P957_F	0.0049	0.0000	0.0053	0.0000	0.0000	0.0000	0.0000	0.0132	0.0011	0.0059	0.0000	0.0034	0.0000	0.0009	0.0000
MME_E29_F	0.0000	0.0000	0.0016	0.0000	0.0053	0.3531	0.0000	0.0082	0.0063	0.0000	0.0000	0.0000	0.0050	0.0000	0.0000
MME_P388_F	0.0000	0.0000	0.0000	0.0000	0.0000	0.0000	0.0000	0.6487	0.0000	0.0000	0.0058	0.0000	0.0000	0.0000	0.0000

MMP19_E274_R	0.0000	0.9950	0.0000	0.0000	0.0000	0.0000	0.0000	0.7876	0.9535	0.9923	0.7789	0.7762	0.9894	0.7422	0.9323
MMP2_E21_R	0.0033	0.0000	0.0000	0.0000	0.0046	0.0000	0.0071	0.0030	0.0057	0.0064	0.0000	0.0082	0.0007	0.0040	0.0000
MMP2_P197_F	0.0066	0.0092	0.0070	0.4922	0.0000	0.0043	0.0000	0.0092	0.0097	0.0000	0.0080	0.0000	0.0000	0.0060	0.0000
MMP2_P303_R	0.0567	0.3453	0.0116	0.0000	0.3025	0.2204	0.3767	0.0114	0.0102	0.0109	0.0111	0.0136	0.0110	0.0224	0.0000
MMP3_P16_R	0.0000	0.9932	0.0000	0.0000	0.9874	0.0000	0.9917	0.9815	0.8487	0.0000	0.0000	0.9875	0.0000	0.6880	0.9877
MMP7_P613_F	0.0284	0.0748	0.9117	0.0718	0.9927	0.9641	0.1879	0.7847	0.9330	0.9323	0.0381	0.7074	0.1968	0.8011	0.8846
MMP9_P189_F	0.0000	0.0000	0.0000	0.0000	0.0000	0.0000	0.0076	0.0098	0.0137	0.0090	0.0079	0.4235	0.0000	0.8683	0.0000
MMP9_P237_R	0.3190	0.0000	0.0073	0.3875	0.5569	0.4137	0.1340	0.0052	0.0104	0.0088	0.0053	0.0067	0.0159	0.0118	0.0000
MOS_E60_R	0.0331	0.0762	0.0093	0.0000	0.0066	0.1040	0.0000	0.0097	0.6429	0.0089	0.0449	0.0108	0.0067	0.0069	0.0000
MPO_P883_R	0.0000	0.0000	0.0041	0.0000	0.0020	0.0000	0.0000	0.0074	0.0054	0.0071	0.0000	0.0055	0.0046	0.0041	0.3968
MSH3_E3_F	0.0192	0.0286	0.9814	0.0000	0.0000	0.9894	0.0000	0.8470	0.9858	0.0000	0.7844	0.8763	0.0000	0.7695	0.9632
MSH3_P13_R	0.0174	0.9938	0.8584	0.2153	0.0382	0.0128	0.7462	0.7065	0.8312	0.8400	0.7991	0.5069	0.0157	0.6950	0.8118
MST1R_P392_F	0.0000	0.0000	0.0000	0.0000	0.0023	0.0000	0.0007	0.0000	0.0016	0.0041	0.0000	0.0000	0.0000	0.0000	0.0000
MT1A_E13_R	0.0183	0.1558	0.0092	0.0000	0.1918	0.0127	0.0091	0.0098	0.0082	0.0089	0.0188	0.0100	0.0085	0.1693	0.0000
MT1A_P49_R	0.0013	0.0034	0.0000	0.0000	0.0000	0.0000	0.0000	0.0000	0.0000	0.0028	0.0002	0.0000	0.0009	0.0000	0.0000
MTA1_P478_F	0.1064	0.0069	0.0076	0.3719	0.0082	0.1988	0.0096	0.4776	0.0130	0.1084	0.0891	0.0106	0.0905	0.0108	0.2706
MUC1_E18_R	0.9767	0.0000	0.1210	0.0000	0.0000	0.0000	0.0000	0.0370	0.9309	0.0294	0.0000	0.0000	0.0000	0.0000	0.0000
MUC1_P191_F	0.0000	0.0000	0.0000	0.0000	0.0028	0.0000	0.0000	0.0055	0.0000	0.0000	0.0000	0.0105	0.0053	0.0139	0.0000
MXI1_P75_R	0.0071	0.0068	0.0047	0.0034	0.0028	0.0057	0.0103	0.0053	0.0063	0.0096	0.0049	0.0102	0.0049	0.0047	0.0000
MYBL2_P211_F	0.0000	0.0079	0.0031	0.0000	0.2935	0.0038	0.0000	0.0110	0.0067	0.0000	0.0060	0.0000	0.0154	0.0109	0.0000
MYCL1_P502_R	0.0000	0.0083	0.0000	0.0000	0.0034	0.0000	0.0104	0.0000	0.0034	0.0000	0.0066	0.0035	0.0041	0.0040	0.0000
MYCN_E77_R	0.0149	0.0141	0.0242	0.0000	0.0738	0.0179	0.0082	0.0166	0.3192	0.0170	0.0031	0.0096	0.0114	0.0075	0.0000
MYCN_P464_R	0.0091	0.0063	0.0026	0.0000	0.0000	0.0103	0.0000	0.0063	0.0051	0.0000	0.0067	0.0052	0.1180	0.0065	0.0000
MYH11_P22_F	0.0000	0.0065	0.0044	0.0000	0.0030	0.0000	0.0051	0.0855	0.0093	0.0092	0.0110	0.0083	0.0913	0.0040	0.6164
MYH11_P236_R	0.0000	0.0046	0.0000	0.0000	0.0000	0.0000	0.0000	0.0000	0.0000	0.0000	0.0004	0.0002	0.0029	0.0023	0.0000
MYLK_P469_R	0.9905	0.0091	0.0072	0.6713	0.3716	0.4965	0.3149	0.0141	0.2082	0.0199	0.0094	0.0150	0.0069	0.0083	0.0000
MYOD1_E156_F	0.9920	0.0052	0.0008	0.0000	0.0317	0.0010	0.0000	0.0000	0.0043	0.0042	0.0000	0.0033	0.0000	0.0000	0.0000
MYOD1_P50_F	0.0000	0.0000	0.0000	0.0000	0.0000	0.0000	0.0000	0.0000	0.0000	0.0000	0.0054	0.0034	0.0000	0.0000	0.0000
NBL1_E205_R	0.9203	0.9252	0.8667	0.9885	0.9930	0.9527	0.0483	0.9872	0.8969	0.9167	0.0691	0.9901	0.8694	0.9825	0.6581
NBL1_P24_F	0.0831	0.9935	0.9864	0.0000	0.9836	0.9838	0.9865	0.0085	0.5822	0.9939	0.0000	0.7168	0.0076	0.9854	0.9727
NCL_P840_R	0.0055	0.0000	0.0000	0.0000	0.0052	0.4537	0.0000	0.0156	0.0144	0.0132	0.0060	0.0096	0.0020	0.0221	0.0000
NDN_P1110_F	0.0000	0.0000	0.0000	0.0000	0.9887	0.0000	0.0000	0.9812	0.7412	0.8695	0.5902	0.9820	0.0000	0.9660	0.9079
NEFL_E23_R	0.9055	0.6334	0.9936	0.9994	0.8487	0.9921	0.9961	0.9733	0.9218	0.9966	0.9947	0.9961	0.9963	0.9159	0.9859

NEFL_P209_R	0.0142	0.0000	0.0012	0.0000	0.0000	0.0000	0.0000	0.0020	0.0000	0.0040	0.0011	0.0000	0.0000	0.0000	0.0000
NEO1_P1067_F	0.0000	0.0000	0.0000	0.0000	0.0000	0.0000	0.0000	0.0000	0.0000	0.0000	0.0000	0.0000	0.0000	0.0042	0.0000
NES_P239_R	0.9830	0.0494	0.0039	0.0000	0.0017	0.0092	0.0000	0.0000	0.0063	0.0000	0.0000	0.0308	0.0000	0.0034	0.0000
NEU1_P745_F	0.0570	0.0000	0.0000	0.0000	0.0000	0.0000	0.0000	0.0087	0.0042	0.0000	0.0000	0.0072	0.0045	0.0068	0.0000
NFKB1_P336_R	0.0000	0.0000	0.2492	0.0000	0.0144	0.5676	0.0000	0.2337	0.0850	0.0000	0.0254	0.0000	0.0000	0.0163	0.0000
NFKB2_P709_R	0.2090	0.0087	0.0196	0.0000	0.0094	0.2057	0.0098	0.0322	0.0256	0.0216	0.1397	0.0128	0.0314	0.0252	0.0000
NGFB_E353_F	0.0049	0.0037	0.0000	0.0000	0.0018	0.0000	0.0059	0.0015	0.6058	0.0000	0.0000	0.0044	0.0050	0.0019	0.0000
NGFB_P13_F	0.8275	0.0171	0.2062	0.4715	0.8820	0.3154	0.3756	0.3457	0.0954	0.0506	0.1652	0.2525	0.0506	0.0358	0.0254
NGFR_E328_F	0.0000	0.0000	0.0041	0.0000	0.0024	0.0000	0.0049	0.0060	0.0067	0.0080	0.0000	0.0044	0.0028	0.7310	0.0000
NGFR_P355_F	0.1099	0.1009	0.3079	0.0793	0.0408	0.3297	0.3130	0.0821	0.1097	0.8243	0.2891	0.1305	0.0962	0.0675	0.0536
NKX3-1_P146_F	0.0000	0.0000	0.0075	0.0000	0.0150	0.1904	0.0000	0.0000	0.0100	0.0000	0.0000	0.0000	0.0076	0.0000	0.0000
NKX3-1_P871_R	0.9915	0.9938	0.0000	0.0000	0.0000	0.9954	0.0000	0.9856	0.9791	0.9932	0.0000	0.9856	0.0000	0.9882	0.9872
NOTCH1_E452_R	0.0000	0.0000	0.0000	0.0000	0.0000	0.0000	0.0000	0.0000	0.0000	0.0000	0.0000	0.0000	0.0000	0.0000	0.0000
NOTCH1_P1198_F	0.0000	0.0000	0.0000	0.0000	0.0001	0.0000	0.0000	0.0000	0.0000	0.0000	0.0000	0.0001	0.0028	0.0000	0.0000
NOTCH2_P312_R	0.0043	0.0063	0.0039	0.8604	0.0045	0.3543	0.0056	0.0039	0.0041	0.0100	0.0053	0.0051	0.0035	0.0014	0.0000
NOTCH3_E403_F	0.0000	0.0000	0.0000	0.0000	0.0000	0.0000	0.0040	0.0000	0.0000	0.0000	0.0000	0.0000	0.0000	0.0003	0.0000
NPR2_P618_F	0.2566	0.6192	0.0000	0.0000	0.0000	0.0000	0.0000	0.0000	0.0098	0.1508	0.3011	0.0030	0.2414	0.0000	0.3301
NPY_P295_F	0.0434	0.0312	0.7039	0.1838	0.6827	0.4900	0.0320	0.5638	0.0337	0.5443	0.0390	0.0676	0.0248	0.6001	0.3409
NPY_P91_F	0.0095	0.0660	0.0159	0.0000	0.1567	0.1803	0.0102	0.0199	0.0247	0.0124	0.0972	0.0137	0.0943	0.0192	0.0000
NQO1_E74_R	0.0151	0.0000	0.0065	0.6744	0.0000	0.0056	0.0000	0.0070	0.0059	0.0000	0.0000	0.0055	0.0056	0.0000	0.0000
NQO1_P345_R	0.0019	0.0053	0.0000	0.0000	0.0030	0.0000	0.0000	0.6303	0.1868	0.0000	0.0000	0.0000	0.0040	0.0011	0.0000
NR2F6_E375_R	0.0109	0.0000	0.0103	0.0000	0.3907	0.0524	0.0859	0.0097	0.0536	0.0194	0.0164	0.0263	0.5955	0.0087	0.0000
NRAS_P103_R	0.0000	0.0000	0.0000	0.0000	0.0034	0.0218	0.0087	0.0000	0.0000	0.0019	0.0073	0.0052	0.0045	0.0023	0.0000
NRAS_P12_R	0.0000	0.0000	0.0000	0.0000	0.0000	0.0000	0.0000	0.0000	0.0000	0.0000	0.0000	0.0000	0.0000	0.0000	0.0000
NRG1_E74_F	0.0000	0.0090	0.0014	0.0000	0.0000	0.0000	0.0000	0.0010	0.0000	0.0050	0.0017	0.0086	0.0038	0.0001	0.0000
NRG1_P558_R	0.0055	0.0045	0.1265	0.0693	0.2604	0.0589	0.0000	0.0045	0.0044	0.0275	0.0051	0.0058	0.0054	0.0033	0.0000
NTRK2_P10_F	0.0133	0.2762	0.0116	0.0000	0.5058	0.0039	0.0869	0.0000	0.0109	0.0252	0.0000	0.0030	0.0069	0.0064	0.0000
NTRK2_P395_R	0.0000	0.0000	0.0000	0.0000	0.0000	0.0000	0.0000	0.0000	0.0000	0.0000	0.0000	0.0000	0.0000	0.0000	0.0000
NTRK3_E131_F	0.0058	0.0091	0.0061	0.0000	0.0000	0.0000	0.0000	0.0000	0.0029	0.0113	0.0000	0.0090	0.0089	0.0000	0.0000
NTRK3_P636_R	0.8659	0.0032	0.0032	0.0000	0.0000	0.0000	0.0028	0.0000	0.0000	0.0023	0.0000	0.0000	0.0000	0.0000	0.0000
NTRK3_P752_F	0.0000	0.0017	0.0032	0.0000	0.0000	0.0000	0.0000	0.0000	0.0003	0.0029	0.0000	0.0040	0.0000	0.0013	0.0000
NTSR1_E109_F	0.0070	0.0073	0.0000	0.0000	0.0000	0.0000	0.0000	0.0068	0.0095	0.0090	0.0098	0.0000	0.0095	0.0093	0.0000
NTSR1_P318_F	0.0149	0.1953	0.3818	0.0000	0.0069	0.0000	0.0000	0.0274	0.0113	0.0825	0.0970	0.5080	0.0083	0.0120	0.0000

OAT_P465_F	0.3489	0.0133	0.4405	0.1166	0.3916	0.4103	0.0104	0.0113	0.0111	0.0132	0.0114	0.0205	0.0095	0.5473	0.0000
ODC1_P424_F	0.0000	0.0000	0.0000	0.0000	0.0000	0.0000	0.0000	0.0000	0.0003	0.0000	0.0032	0.0014	0.0023	0.0000	0.0000
ONECUT2_E96_F	0.0000	0.0134	0.0107	0.0000	0.7572	0.0083	0.7489	0.0071	0.0295	0.0000	0.6411	0.1418	0.0339	0.3216	0.0000
ONECUT2_P315_R	0.0000	0.0121	0.0000	0.0000	0.0000	0.0000	0.0000	0.0000	0.0000	0.0000	0.0067	0.0000	0.0000	0.0044	0.0000
OPCML_E219_R	0.0043	0.0047	0.0030	0.5627	0.0033	0.0043	0.0068	0.0045	0.0076	0.0095	0.0000	0.7040	0.0000	0.0033	0.0000
OPCML_P71_F	0.0134	0.0096	0.0078	0.0000	0.0073	0.0000	0.0000	0.0083	0.0049	0.0000	0.0156	0.0051	0.0023	0.0000	0.3557
OSM_P188_F	0.0059	0.0698	0.0105	0.0000	0.0047	0.0775	0.0867	0.0936	0.0124	0.0387	0.0115	0.0079	0.9631	0.0087	0.0000
p16_seq_47_S188_R	0.0187	0.0115	0.0029	0.0078	0.0000	0.0042	0.0000	0.0140	0.0110	0.0525	0.0047	0.0050	0.0164	0.0065	0.0000
P2RX7_E323_R	0.0000	0.0000	0.0030	0.0000	0.0023	0.1902	0.1327	0.0077	0.0026	0.0043	0.0022	0.0075	0.0039	0.0028	0.4160
P2RX7_P119_R	0.1246	0.0135	0.0138	0.6962	0.1498	0.1913	0.1980	0.0206	0.0901	0.0203	0.0152	0.0182	0.0197	0.0193	0.0017
P2RX7_P597_F	0.2700	0.9957	0.5527	0.8134	0.8207	0.3958	0.3701	0.6501	0.9829	0.9941	0.8378	0.3369	0.9899	0.8631	0.7461
PALM2-AKAP2_P183_R	0.7266	0.0299	0.0297	0.3142	0.3525	0.7146	0.5395	0.0308	0.0276	0.5468	0.0329	0.0626	0.0279	0.0226	0.0154
PALM2-AKAP2_P420_R	0.0066	0.0128	0.0000	0.0000	0.0130	0.0000	0.0245	0.0093	0.2056	0.0083	0.0040	0.0000	0.0019	0.0160	0.1459
PARP1_P610_R	0.8904	0.0065	0.9536	0.0000	0.9895	0.0000	0.0000	0.6431	0.8187	0.4631	0.0089	0.9103	0.5604	0.8162	0.9830
PAX6_P1121_F	0.0111	0.0135	0.0082	0.0000	0.0051	0.2121	0.1197	0.0111	0.0144	0.0000	0.2776	0.0268	0.0026	0.0072	0.0000
PAX6_P50_R	0.0000	0.0000	0.0005	0.0000	0.0000	0.0000	0.0000	0.0000	0.0000	0.0016	0.0000	0.0000	0.0000	0.0000	0.0000
PCDH1_E22_F	0.0140	0.0079	0.1218	0.0000	0.1188	0.0081	0.0000	0.2213	0.0212	0.1671	0.0000	0.2205	0.0106	0.0219	0.0000
PCGF4_P760_R	0.0051	0.0827	0.9910	0.0000	0.0047	0.0084	0.0091	0.0103	0.0118	0.0924	0.0767	0.0079	0.1006	0.9797	0.0000
PCGF4_P92_R	0.0069	0.0277	0.0463	0.0000	0.0082	0.0000	0.0168	0.0280	0.7178	0.0243	0.0275	0.0260	0.0144	0.0143	0.0000
PCTK1_E77_R	0.9974	0.9980	0.9345	0.9907	0.9466	0.9984	0.9619	0.9652	0.9490	0.9978	0.9967	0.9876	0.9450	0.8896	0.9219
PDE1B_E141_F	0.0110	0.0071	0.0127	0.5141	0.0107	0.8067	0.0200	0.0138	0.0153	0.0148	0.0072	0.0418	0.0057	0.0144	0.0000
PDE1B_P263_R	0.0055	0.0000	0.0111	0.0000	0.1431	0.0073	0.0000	0.5954	0.3605	0.0125	0.0094	0.0119	0.0086	0.0069	0.0000
PDGFA_P841_R	0.0000	0.0000	0.0027	0.0000	0.0000	0.0000	0.0000	0.3819	0.0009	0.0000	0.0009	0.0000	0.0000	0.0002	0.0000
PDGFRB_E195_R	0.0094	0.0061	0.0163	0.0000	0.0000	0.0067	0.0000	0.0000	0.0102	0.0000	0.0042	0.8711	0.0000	0.0070	0.0000
PDGFRB_P343_F	0.0011	0.0035	0.0000	0.0052	0.0000	0.0000	0.0000	0.0031	0.0033	0.0032	0.0000	0.0053	0.0036	0.0041	0.0000
PEG10_P978_R	0.0921	0.0000	0.8706	0.0000	0.0036	0.0000	0.0066	0.0707	0.0022	0.0079	0.0067	0.0459	0.0054	0.0448	0.0000
PENK_E26_F	0.0274	0.0262	0.2596	0.0981	0.0386	0.0489	0.0252	0.2318	0.1340	0.0273	0.0456	0.0307	0.0298	0.0302	0.1201
PENK_P447_R	0.0000	0.0000	0.1306	0.5350	0.0039	0.2619	0.0069	0.0271	0.0072	0.0080	0.0080	0.0119	0.3378	0.0986	0.0000
PGF_E33_F	0.0088	0.0063	0.0109	0.0000	0.1497	0.4036	0.0000	0.0211	0.0109	0.0105	0.0133	0.1956	0.0100	0.7540	0.0000
PHLDA2_P622_F	0.0088	0.0065	0.0053	0.0000	0.8205	0.0000	0.0072	0.0041	0.0070	0.0096	0.0044	0.0106	0.0000	0.0063	0.0000
PI3_P1394_R	0.3062	0.4307	0.5573	0.8139	0.0148	0.6564	0.0183	0.8935	0.6712	0.0284	0.9870	0.3406	0.2973	0.0133	0.9726
PITX2_E24_R	0.0070	0.2185	0.0056	0.0000	0.2003	0.1954	0.0052	0.0113	0.0101	0.1483	0.0117	0.6151	0.2504	0.0224	0.0000
PITX2_P183_R	0.0000	0.0088	0.0027	0.0000	0.0000	0.0000	0.0057	0.0000	0.0000	0.0000	0.0000	0.0030	0.0000	0.0000	0.0000

PKD2_P287_R	0.1708	0.0000	0.3779	0.0000	0.3293	0.1851	0.0092	0.0000	0.0101	0.0347	0.1898	0.0190	0.0075	0.0045	0.0000
PKD2_P336_R	0.3694	0.0133	0.0065	0.5580	0.5372	0.0040	0.1869	0.4153	0.0111	0.2075	0.0061	0.0111	0.0087	0.0150	0.0814
PLAU_P11_F	0.1286	0.0233	0.0143	0.0000	0.0120	0.2483	0.3740	0.0195	0.0223	0.3730	0.0141	0.0190	0.3108	0.0185	0.4611
PLAU_P176_R	0.0068	0.0069	0.0086	0.0000	0.0000	0.0031	0.0000	0.0068	0.0000	0.0000	0.0000	0.0000	0.0042	0.0000	0.0000
PLAUR_E123_F	0.0000	0.0025	0.8745	0.0000	0.0010	0.0000	0.0040	0.4506	0.0058	0.0000	0.0019	0.0054	0.0019	0.0011	0.0000
PLAUR_P82_F	0.0070	0.0046	0.0007	0.7304	0.0030	0.0045	0.0051	0.0037	0.0011	0.0080	0.0068	0.0000	0.0033	0.0039	0.0000
PLG_E406_F	0.0000	0.7916	0.4641	0.0000	0.8951	0.9960	0.8730	0.9877	0.9894	0.9936	0.9938	0.9880	0.6829	0.9716	0.8092
PLSCR3_P751_R	0.1361	0.0198	0.0127	0.0000	0.0135	0.0089	0.0095	0.0134	0.4036	0.0103	0.0127	0.0210	0.0406	0.3208	0.1620
PLXDC1_P236_F	0.1931	0.0053	0.0037	0.0000	0.0000	0.0000	0.0000	0.0139	0.0111	0.0000	0.0105	0.0141	0.0000	0.0088	0.0000
PLXDC2_E337_F	0.0077	0.0000	0.0087	0.0000	0.0069	0.4921	0.0000	0.0000	0.0037	0.0522	0.0093	0.0071	0.0000	0.0016	0.2256
PLXDC2_P914_R	0.3183	0.0329	0.0289	0.2438	0.0268	0.4287	0.0217	0.0381	0.0287	0.0461	0.0431	0.0523	0.3332	0.4978	0.0077
PMP22_P975_F	0.9919	0.0000	0.0000	0.0000	0.0000	0.0000	0.0000	0.0341	0.0000	0.0000	0.9918	0.9894	0.0000	0.0000	0.4654
PODXL_P1341_R	0.6478	0.0096	0.0079	0.2103	0.0043	0.0547	0.0077	0.0076	0.0053	0.1173	0.0077	0.0116	0.0000	0.0056	0.0000
POMC_E254_F	0.0064	0.0036	0.5830	0.0000	0.0588	0.0000	0.7016	0.0170	0.0122	0.0000	0.0153	0.0118	0.0000	0.0132	0.0373
POMC_P400_R	0.0000	0.0000	0.0000	0.0000	0.0085	0.1826	0.1787	0.0081	0.0080	0.0000	0.0061	0.0083	0.0000	0.0064	0.0000
POMC_P53_F	0.0125	0.0073	0.0030	0.0000	0.0108	0.0000	0.0000	0.0180	0.0107	0.3061	0.1210	0.0137	0.0060	0.0103	0.0000
PPARD_P846_F	0.0000	0.0062	0.0000	0.5330	0.0000	0.0039	0.0000	0.0000	0.7359	0.0000	0.0000	0.0000	0.0074	0.0000	0.0000
PPARG_E178_R	0.0117	0.0000	0.0000	0.0000	0.0688	0.0000	0.0000	0.0113	0.0049	0.1499	0.0079	0.0074	0.0033	0.0029	0.0000
PPP2R1B_P268_R	0.0000	0.0000	0.0050	0.0000	0.0052	0.0876	0.0000	0.0059	0.0049	0.0119	0.0000	0.0035	0.0002	0.0084	0.0000
PRDM2_P1340_R	0.0000	0.0000	0.0339	0.0000	0.0273	0.0000	0.9875	0.1501	0.0106	0.9839	0.0080	0.0000	0.9761	0.0718	0.9645
PRKCDBP_E206_F	0.0000	0.0000	0.0000	0.0000	0.0000	0.0000	0.0059	0.0000	0.0020	0.0058	0.0000	0.0067	0.0000	0.0041	0.0000
PROK2_E0_F	0.0061	0.0000	0.0000	0.0000	0.0022	0.0000	0.0000	0.0000	0.0051	0.0000	0.0065	0.0000	0.0000	0.0007	0.0000
PROK2_P390_F	0.0101	0.0060	0.0000	0.7176	0.0000	0.0089	0.0000	0.0000	0.0000	0.0000	0.0000	0.0000	0.0000	0.0000	0.0000
PRSS8_E134_R	0.9934	0.1469	0.9923	0.1377	0.3895	0.1531	0.9952	0.9885	0.9875	0.9939	0.9913	0.3297	0.2280	0.9884	0.9889
PSCA_E359_F	0.0001	0.0000	0.0000	0.0000	0.0000	0.0000	0.0000	0.0035	0.0064	0.0000	0.0105	0.0080	0.0000	0.0000	0.0000
PSIP1_P163_R	0.0088	0.0058	0.0000	0.0000	0.0000	0.0000	0.0122	0.0152	0.5102	0.0000	0.0048	0.0113	0.0064	0.0000	0.0000
PTCH_E42_F	0.3550	0.0051	0.0096	0.0000	0.2846	0.0000	0.0450	0.2302	0.0121	0.0093	0.0089	0.0271	0.0528	0.0000	0.0000
PTCH2_P568_R	0.0086	0.0047	0.0435	0.0000	0.0083	0.0001	0.0085	0.0323	0.3265	0.0289	0.8482	0.0100	0.4959	0.5213	0.0000
PTEN_P438_F	0.0117	0.0075	0.0063	0.0000	0.0053	0.0046	0.0000	0.0060	0.0081	0.0116	0.0078	0.0098	0.0000	0.0094	0.0000
PTGS1_P2_F	0.0131	0.0157	0.0211	0.0000	0.0024	0.0000	0.0091	0.0080	0.4216	0.0094	0.0139	0.0000	0.0000	0.0080	0.0000
PTGS2_P308_F	0.0048	0.1221	0.0078	0.2673	0.0037	0.0229	0.0084	0.0037	0.0075	0.0097	0.0888	0.1474	0.1427	0.0028	0.0000
PTGS2_P524_R	0.0081	0.0094	0.0000	0.0000	0.0000	0.3107	0.0000	0.0094	0.1319	0.0170	0.0075	0.0121	0.0092	0.0072	0.3242
PTHLH_P15_R	0.0000	0.0000	0.0678	0.1891	0.0000	0.0040	0.0000	0.0000	0.0018	0.0000	0.0020	0.0019	0.0556	0.0000	0.0000

PTHR1_P170_R	0.2567	0.0000	0.9923	0.0816	0.8859	0.0000	0.2640	0.0000	0.9491	0.0000	0.0000	0.0000	0.0059	0.0361	0.8552
PTK2_P735_R	0.0120	0.4294	0.2154	0.0000	0.0088	0.0000	0.0000	0.0091	0.0120	0.0138	0.2028	0.0091	0.0052	0.0093	0.0000
PTK2B_P673_R	0.0000	0.0035	0.0054	0.0000	0.0000	0.0000	0.0000	0.0000	0.0077	0.0000	0.0045	0.0099	0.0027	0.0057	0.0000
PTPN6_E171_R	0.0055	0.0058	0.0000	0.0809	0.0019	0.0000	0.0026	0.0000	0.0000	0.0000	0.0045	0.0066	0.0021	0.0000	0.0000
PTPN6_P282_R	0.0000	0.0035	0.0000	0.0000	0.0000	0.0000	0.0000	0.0000	0.0000	0.0000	0.0000	0.0000	0.0000	0.0000	0.0000
PTPNS1_E433_R	0.0000	0.0000	0.0006	0.0000	0.0015	0.0000	0.0000	0.0000	0.0000	0.0000	0.0000	0.0000	0.0000	0.0000	0.0000
PTPNS1_P301_R	0.0000	0.0000	0.0000	0.0000	0.0368	0.0033	0.3122	0.1233	0.0135	0.2414	0.0128	0.0000	0.0094	0.0058	0.0000
PTPRF_E178_R	0.8050	0.0088	0.0031	0.0000	0.0000	0.0028	0.0014	0.3804	0.0506	0.0079	0.0000	0.0015	0.0060	0.0043	0.0000
PTPRG_E40_R	0.0075	0.2310	0.0079	0.0288	0.1635	0.2664	0.1813	0.0099	0.0103	0.0124	0.6408	0.0076	0.3239	0.3307	0.0000
PTPRG_P476_F	0.0000	0.1433	0.2095	0.0000	0.0000	0.0000	0.0060	0.0067	0.0084	0.0080	0.0078	0.0072	0.0000	0.0047	0.0000
PTPRO_E56_F	0.0461	0.0608	0.0001	0.0455	0.0026	0.0896	0.0301	0.0018	0.0000	0.0069	0.0053	0.0000	0.0019	0.0010	0.0000
PURA_P928_R	0.0024	0.0063	0.0000	0.0000	0.0000	0.0000	0.0000	0.0000	0.0015	0.0000	0.0000	0.0000	0.0000	0.0000	0.0000
PWCR1_P811_F	0.9887	0.0000	0.0000	0.0000	0.0000	0.0000	0.0000	0.9705	0.9779	0.0070	0.9882	0.0000	0.0000	0.9792	0.9804
PYCARD_E87_F	0.0747	0.0731	0.0127	0.0363	0.2205	0.1217	0.0642	0.0085	0.0134	0.0274	0.0611	0.0139	0.0969	0.0189	0.0000
PYCARD_P150_F	0.0662	0.0077	0.0091	0.0000	0.9334	0.0000	0.0072	0.7829	0.0149	0.0105	0.0000	0.0177	0.0168	0.0085	0.1780
RAB32_E314_R	0.0068	0.0000	0.0000	0.0000	0.0053	0.0000	0.0000	0.0022	0.0003	0.0123	0.0000	0.0108	0.0000	0.0063	0.0000
RAB32_P493_R	0.0000	0.0032	0.0003	0.0000	0.0651	0.0000	0.0000	0.0018	0.0000	0.0048	0.0038	0.0000	0.0000	0.0000	0.0000
RAF1_P330_F	0.0000	0.0000	0.0000	0.0000	0.0000	0.0032	0.0000	0.9489	0.0033	0.0068	0.0000	0.0041	0.0013	0.0000	0.4779
RAN_P581_R	0.0000	0.0000	0.7350	0.0000	0.9917	0.0000	0.0000	0.0038	0.0039	0.9935	0.9895	0.0031	0.9908	0.0023	0.0000
RARA_E128_R	0.0035	0.0000	0.0033	0.0000	0.0019	0.0000	0.0000	0.0074	0.0078	0.0058	0.0050	0.0080	0.0580	0.2530	0.0000
RARA_P176_R	0.0010	0.0031	0.0000	0.0000	0.0018	0.0000	0.0000	0.0000	0.0036	0.0000	0.0000	0.9249	0.0000	0.0000	0.0000
RARB_E114_F	0.0033	0.0000	0.6047	0.0000	0.0008	0.0000	0.0046	0.0035	0.0045	0.0064	0.0035	0.0922	0.0041	0.0031	0.0000
RARB_P60_F	0.0000	0.9152	0.0013	0.0000	0.0000	0.0041	0.0068	0.7404	0.0000	0.0005	0.9304	0.0000	0.0022	0.0031	0.3402
RARRES1_E235_F	0.0112	0.0789	0.0081	0.0879	0.0093	0.1231	0.0141	0.0101	0.1511	0.0146	0.0857	0.0095	0.0084	0.0069	0.0000
RASGRF1_E16_F	0.0115	0.1351	0.0091	0.0000	0.0004	0.0028	0.0493	0.0073	0.0082	0.0078	0.2852	0.9883	0.0000	0.0061	0.0000
RASGRF1_P768_F	0.0000	0.1764	0.2118	0.7023	0.0064	0.0102	0.3278	0.0016	0.0091	0.0066	0.0097	0.0149	0.0041	0.0040	0.0000
RASSF1_E116_F	0.0010	0.0023	0.0062	0.0000	0.1215	0.0000	0.0000	0.0030	0.0068	0.2972	0.1901	0.0099	0.0060	0.0000	0.0000
RASSF1_P244_F	0.0000	0.0000	0.2588	0.4203	0.2575	0.0000	0.0066	0.1096	0.0105	0.0079	0.2493	0.0000	0.0132	0.0124	0.0000
RBL2_P250_R	0.0000	0.0000	0.0041	0.0000	0.0052	0.0000	0.0943	0.0066	0.0059	0.0109	0.0000	0.0026	0.0329	0.0128	0.0000
RBP1_E158_F	0.0000	0.0000	0.0011	0.0000	0.0000	0.0031	0.0000	0.0001	0.0000	0.0015	0.0020	0.0000	0.0016	0.0000	0.0000
RBP1_P150_F	0.0000	0.0000	0.0000	0.0000	0.0000	0.0000	0.0000	0.0000	0.0000	0.0000	0.0000	0.0000	0.0000	0.0000	0.0000
RET_P717_F	0.1874	0.0081	0.1534	0.0000	0.0071	0.2830	0.0000	0.0121	0.0107	0.0339	0.0081	0.0090	0.0097	0.0080	0.0000
RET_seq_53_S374_F	0.0071	0.0030	0.3214	0.0000	0.2945	0.1730	0.2906	0.0114	0.0053	0.1547	0.2356	0.0036	0.0068	0.8243	0.0000

RHOC_P536_F	0.0000	0.0000	0.0000	0.0000	0.0017	0.0000	0.0000	0.0000	0.0000	0.0000	0.0005	0.0000	0.0000	0.0000	0.0000
RHOH_P121_F	0.0022	0.0059	0.0000	0.0000	0.0000	0.0045	0.0000	0.0000	0.0000	0.0000	0.0000	0.0027	0.0000	0.0000	0.0000
RHOH_P953_R	0.9909	0.1778	0.0000	0.0000	0.0047	0.0000	0.0000	0.9779	0.8630	0.0000	0.0000	0.0057	0.0000	0.9798	0.9739
RIPK1_P868_F	0.9456	0.5477	0.9907	0.5983	0.9902	0.6204	0.4032	0.9842	0.9725	0.6852	0.0243	0.0961	0.5052	0.0523	0.9798
RIPK2_E123_F	0.6083	0.0000	0.0000	0.0000	0.0065	0.0000	0.0090	0.0090	0.9389	0.0000	0.0000	0.0000	0.0000	0.0059	0.0000
RIPK3_P124_F	0.0336	0.0519	0.1203	0.0000	0.0104	0.0041	0.0938	0.0096	0.0113	0.0000	0.0082	0.0093	0.0094	0.0000	0.0000
RIPK3_P24_F	0.0000	0.0000	0.0000	0.0000	0.0000	0.0000	0.0000	0.0000	0.0020	0.0052	0.0000	0.0000	0.0000	0.0000	0.0000
RIPK4_E166_F	0.0000	0.0046	0.0021	0.0000	0.0000	0.1263	0.0000	0.0031	0.0012	0.0000	0.0000	0.0033	0.0000	0.0000	0.3241
RIPK4_P172_F	0.0103	0.0057	0.4100	0.0000	0.0096	0.0039	0.0139	0.2891	0.4576	0.0107	0.0068	0.5374	0.0082	0.0083	0.0000
ROR1_P6_F	0.0000	0.0000	0.0128	0.0000	0.0505	0.0000	0.0000	0.5442	0.0062	0.0000	0.0000	0.0111	0.0083	0.0068	0.0000
ROR2_P317_R	0.0000	0.0000	0.0000	0.0000	0.0000	0.9958	0.0089	0.0000	0.0000	0.0000	0.0000	0.0006	0.0037	0.0000	0.0000
RRAS_P100_R	0.0000	0.0000	0.0000	0.0000	0.0000	0.0000	0.0000	0.0000	0.0016	0.0000	0.0000	0.0000	0.0000	0.0000	0.0000
RUNX3_P247_F	0.7629	0.0309	0.0000	0.0000	0.0000	0.0000	0.0000	0.0000	0.9653	0.9886	0.0038	0.6194	0.0000	0.9858	0.8919
RYK_P493_F	0.0024	0.0000	0.0000	0.0000	0.0000	0.0000	0.0011	0.0000	0.0000	0.0000	0.0000	0.0000	0.8207	0.0000	0.0000
S100A2_E36_R	0.0112	0.0000	0.0000	0.0000	0.0000	0.0000	0.0000	0.0050	0.4568	0.0000	0.0000	0.0000	0.0000	0.0000	0.7465
S100A4_E315_F	0.2206	0.0172	0.0151	0.8264	0.2323	0.5692	0.1086	0.0095	0.0083	0.0111	0.0176	0.0120	0.0113	0.0072	0.2892
SCGB3A1_E55_R	0.0030	0.0000	0.0000	0.0000	0.0000	0.0000	0.0000	0.0035	0.0000	0.0000	0.0050	0.0035	0.0000	0.0000	0.0000
SCGB3A1_P103_R	0.0000	0.0000	0.0000	0.0000	0.0000	0.0000	0.0000	0.0000	0.0000	0.0000	0.0036	0.0000	0.0000	0.0000	0.0000
SEMA3A_P343_F	0.0000	0.9942	0.0016	0.0000	0.0053	0.0048	0.0000	0.0020	0.0000	0.0000	0.0000	0.0000	0.0000	0.0060	0.0000
SEMA3A_P658_R	0.0911	0.0557	0.1115	0.0000	0.0091	0.1204	0.8083	0.0383	0.8919	0.0480	0.0830	0.8978	0.0858	0.0143	0.0000
SEMA3C_E49_R	0.1588	0.1491	0.0117	0.0000	0.0071	0.1514	0.0119	0.0136	0.0115	0.0121	0.0129	0.0126	0.1703	0.0152	0.0000
SEMA3C_P642_F	0.0038	0.1795	0.0000	0.0000	0.0000	0.0000	0.1285	0.0282	0.9214	0.0111	0.1537	0.0000	0.0044	0.0000	0.0000
SEMA3F_E333_R	0.0000	0.0000	0.0194	0.0000	0.0053	0.0000	0.0112	0.0132	0.0072	0.0000	0.0000	0.0072	0.0047	0.0068	0.0000
SEMA3F_P692_R	0.0000	0.0000	0.0000	0.0000	0.0000	0.0000	0.0050	0.0039	0.0042	0.0057	0.0015	0.0000	0.0000	0.7681	0.0000
SEPT5_P441_F	0.0235	0.0108	0.9918	0.0000	0.0491	0.2022	0.0220	0.5954	0.4910	0.0000	0.0709	0.0097	0.0081	0.0099	0.6918
SEPT9_P58_R	0.0123	0.4591	0.0034	0.0000	0.0082	0.7027	0.1406	0.0373	0.8934	0.5968	0.9899	0.9909	0.7780	0.9801	0.6888
SERPINA5_E69_F	0.9903	0.8533	0.0130	0.8254	0.0113	0.5809	0.0200	0.6304	0.8336	0.3360	0.0220	0.9843	0.3171	0.9822	0.0000
SERPINB2_P939_F	0.0000	0.0000	0.9879	0.0000	0.9873	0.9952	0.0000	0.9893	0.9794	0.9918	0.9894	0.9839	0.0000	0.9921	0.9399
SERPINE1_E189_R	0.2281	0.0102	0.0093	0.0000	0.9930	0.1513	0.4651	0.3088	0.1892	0.0113	0.0161	0.0139	0.3273	0.0112	0.2459
SEZ6L_P249_F	0.0048	0.0052	0.0093	0.0261	0.0027	0.0048	0.0000	0.0000	0.0062	0.0062	0.0000	0.0000	0.0044	0.0043	0.0000
SEZ6L_P299_F	0.0013	0.0000	0.0121	0.0000	0.0000	0.0000	0.0000	0.0000	0.0044	0.0000	0.0000	0.0047	0.0033	0.0071	0.0000
SFN_E118_F	0.9239	0.0872	0.7797	0.6634	0.7648	0.9135	0.0411	0.7122	0.9853	0.5804	0.5028	0.9906	0.8880	0.9899	0.9901
SFRP1_E398_R	0.0000	0.0000	0.0011	0.0000	0.0000	0.0000	0.0000	0.0000	0.0020	0.0045	0.0171	0.0000	0.0000	0.0000	0.0000

SFRP1_P157_F	0.0088	0.0056	0.4076	0.0000	0.0000	0.0000	0.0000	0.0034	0.0053	0.0000	0.0009	0.3988	0.0000	0.0033	0.0000
SFTPA1_E340_R	0.0000	0.9950	0.0010	0.0000	0.0000	0.0000	0.9940	0.9805	0.9869	0.0000	0.0000	0.0000	0.9925	0.0000	0.9856
SH3BP2_P771_R	0.0000	0.0000	0.0060	0.0000	0.0000	0.0000	0.0045	0.0000	0.5264	0.0075	0.0000	0.0088	0.0000	0.0000	0.0000
SHB_P473_R	0.0078	0.0000	0.0000	0.5290	0.0000	0.0018	0.0000	0.0050	0.0053	0.0056	0.0000	0.0066	0.0065	0.0059	0.0000
SHB_P691_R	0.0082	0.0107	0.5261	0.4359	0.4462	0.2986	0.4044	0.0206	0.0123	0.1033	0.4493	0.0159	0.0157	0.0080	0.0000
SHH_E328_F	0.0000	0.0000	0.0000	0.0000	0.0000	0.0000	0.0000	0.0000	0.0000	0.9888	0.0000	0.0000	0.0011	0.0000	0.0000
SHH_P104_R	0.0119	0.0000	0.0073	0.0000	0.0095	0.0000	0.0000	0.0138	0.0107	0.0000	0.0048	0.0128	0.0085	0.0037	0.0000
SIN3B_P514_R	0.0000	0.0000	0.9781	0.0000	0.9719	0.0000	0.0000	0.7939	0.9799	0.0000	0.9778	0.9584	0.9710	0.9735	0.9539
SKI_E465_R	0.0000	0.0079	0.0000	0.0000	0.0081	0.0000	0.0067	0.0123	0.0071	0.0080	0.0054	0.0058	0.0070	0.0088	0.0000
SLC22A2_E271_R	0.0000	0.0000	0.0564	0.0000	0.9915	0.0000	0.5408	0.9853	0.9890	0.0000	0.3293	0.9905	0.0000	0.9859	0.9823
SLC22A3_P634_F	0.8515	0.0152	0.1636	0.0000	0.0110	0.0000	0.0000	0.4644	0.1209	0.0084	0.0057	0.0099	0.0024	0.4354	0.1665
SLC6A8_seq_28_S227_F	0.0000	0.1147	0.0000	0.0000	0.0000	0.0000	0.0000	0.0000	0.0048	0.0062	0.0260	0.0013	0.0000	0.7710	0.0000
SLIT2_P208_F	0.9933	0.3646	0.2193	0.6948	0.0098	0.0053	0.3509	0.1561	0.0095	0.0105	0.0097	0.0265	0.0087	0.2562	0.0000
SMAD2_P708_R	0.0000	0.0080	0.0017	0.0011	0.0000	0.0000	0.0000	0.0022	0.0076	0.0059	0.0038	0.0046	0.0011	0.0086	0.0000
SMAD2_P848_R	0.0059	0.0034	0.0004	0.0000	0.0000	0.0000	0.0000	0.0002	0.0000	0.0000	0.0022	0.0050	0.0025	0.0000	0.0000
SMAD4_P474_R	0.0011	0.0011	0.0000	0.0000	0.0000	0.0000	0.0006	0.0000	0.0028	0.0000	0.0000	0.0038	0.0000	0.0004	0.0000
SMARCA3_E20_F	0.0000	0.0063	0.0000	0.0000	0.0016	0.0000	0.0000	0.0047	0.0036	0.0054	0.0041	0.0058	0.0000	0.0040	0.0000
SMARCA3_P109_R	0.6137	0.0061	0.3427	0.3783	0.5125	0.3198	0.0095	0.0083	0.0139	0.0084	0.0389	0.5638	0.0132	0.0102	0.0000
SMARCA3_P17_R	0.0000	0.0073	0.0000	0.0000	0.0000	0.0038	0.0000	0.0135	0.0027	0.0000	0.0000	0.0000	0.0029	0.0028	0.0000
SMARCA4_P362_R	0.0051	0.0544	0.0111	0.0000	0.0050	0.0954	0.0568	0.0082	0.0071	0.0069	0.0048	0.0086	0.0077	0.0042	0.0000
SMO_E57_F	0.0030	0.0000	0.0000	0.0000	0.0028	0.0000	0.0000	0.0000	0.0078	0.0027	0.0019	0.0000	0.0000	0.0018	0.0000
SOD3_P225_F	0.0000	0.1316	0.0964	0.4844	0.9910	0.9960	0.2362	0.0000	0.9781	0.0100	0.0870	0.0208	0.2073	0.0000	0.9731
SOX1_P1018_R	0.0005	0.1310	0.0783	0.0000	0.0000	0.0000	0.0273	0.0049	0.0010	0.0176	0.0025	0.0025	0.0000	0.0000	0.0000
SOX1_P294_F	0.5392	0.0218	0.0093	0.8563	0.0110	0.7875	0.4651	0.0194	0.0139	0.5052	0.0111	0.5313	0.0102	0.0106	0.0000
SOX17_P287_R	0.0000	0.0000	0.0000	0.0000	0.0000	0.0007	0.0023	0.0000	0.0000	0.0000	0.0025	0.0000	0.0000	0.0000	0.0000
SOX17_P303_F	0.7342	0.7903	0.2215	0.5453	0.9069	0.8048	0.7428	0.7251	0.1117	0.7796	0.7471	0.2310	0.7226	0.1436	0.1419
SOX2_P546_F	0.0000	0.0064	0.1893	0.0000	0.0068	0.0216	0.0097	0.0000	0.0113	0.0101	0.0000	0.0097	0.0053	0.0092	0.0000
SPARC_E50_R	0.0099	0.0069	0.0068	0.0000	0.0042	0.0000	0.0049	0.3476	0.1182	0.0101	0.0043	0.2535	0.5445	0.0079	0.0053
SPARC_P195_F	0.0054	0.0075	0.6208	0.0000	0.0044	0.0000	0.0265	0.0072	0.0021	0.0000	0.0000	0.9792	0.7786	0.0036	0.0000
SPDEF_E116_R	0.1655	0.1813	0.2039	0.8385	0.4154	0.2878	0.1556	0.7076	0.0940	0.2223	0.1953	0.5883	0.1361	0.2131	0.0014
SPI1_E205_F	0.0129	0.1865	0.0161	0.0000	0.0091	0.2362	0.1726	0.0218	0.6423	0.2041	0.1832	0.0115	0.1524	0.0219	0.0000
SPP1_E140_R	0.2215	0.0000	0.0074	0.0000	0.0084	0.0000	0.0117	0.0069	0.0159	0.0000	0.0056	0.0054	0.0040	0.0018	0.0000
SRC_P297_F	0.0000	0.3202	0.0402	0.0000	0.9912	0.3901	0.0000	0.1862	0.9873	0.9955	0.0100	0.0446	0.1676	0.9501	0.9862

ST6GAL1_P164_R	0.0000	0.0926	0.0000	0.0000	0.0507	0.0000	0.0000	0.0000	0.0608	0.0042	0.0000	0.0000	0.0626	0.1553	0.0000
ST6GAL1_P528_F	0.0000	0.0000	0.0000	0.0000	0.0036	0.0000	0.1340	0.0067	0.0077	0.0077	0.0044	0.0000	0.0000	0.0015	0.0000
STK23_E182_R	0.0073	0.0064	0.0000	0.0000	0.0055	0.0000	0.0000	0.0000	0.7833	0.0000	0.0000	0.0116	0.9496	0.9870	0.9199
SYK_E372_F	0.0049	0.0120	0.2874	0.2748	0.1887	0.4056	0.0129	0.0186	0.0162	0.0148	0.0668	0.0102	0.0912	0.0208	0.0000
TAL1_E122_F	0.4446	0.0124	0.0079	0.7959	0.0086	0.5807	0.0094	0.2790	0.0077	0.0133	0.0089	0.0161	0.3449	0.0059	0.0000
TAL1_P594_F	0.0000	0.0071	0.0000	0.0371	0.0057	0.0000	0.3072	0.0056	0.0011	0.0000	0.0000	0.0000	0.0000	0.0036	0.0000
TAL1_P817_F	0.0000	0.0000	0.0000	0.1187	0.0000	0.0000	0.0000	0.0000	0.0000	0.0000	0.0000	0.0000	0.0000	0.0000	0.0000
TCF4_P175_R	0.0000	0.0000	0.0000	0.0000	0.0000	0.0000	0.0000	0.0000	0.0000	0.0000	0.0000	0.0000	0.0000	0.0000	0.0000
TCF7L2_E411_F	0.0000	0.0000	0.0004	0.0000	0.0033	0.0057	0.0000	0.0008	0.0001	0.0000	0.0045	0.0035	0.0000	0.0000	0.3989
TCF7L2_P193_R	0.0059	0.0000	0.0000	0.0000	0.0086	0.0000	0.0000	0.6295	0.0039	0.0000	0.0037	0.0030	0.0048	0.0048	0.0000
TERT_E20_F	0.0085	0.0089	0.0104	0.0000	0.0043	0.1646	0.0104	0.0128	0.0115	0.0119	0.0109	0.0161	0.0116	0.0090	0.0009
TERT_P360_R	0.0123	0.0163	0.0174	0.8310	0.3011	0.0295	0.5313	0.0405	0.0133	0.5658	0.5776	0.4907	0.0170	0.0303	0.0000
TES_E172_F	0.0015	0.0000	0.0044	0.0000	0.0000	0.0000	0.0000	0.0000	0.0051	0.2146	0.0024	0.0000	0.0049	0.0095	0.8685
TESK2_P252_R	0.0065	0.0634	0.0058	0.0000	0.0534	0.0112	0.0059	0.0107	0.7062	0.0075	0.0053	0.0086	0.0050	0.0061	0.0000
TFAP2C_E260_F	0.0000	0.0000	0.9921	0.0568	0.0047	0.0000	0.0000	0.0000	0.0047	0.0000	0.0000	0.0000	0.0000	0.0061	0.0000
TFAP2C_P765_F	0.0000	0.0000	0.0108	0.0000	0.0000	0.0000	0.0000	0.0102	0.0093	0.0000	0.0000	0.0000	0.0000	0.0098	0.0000
TFF2_P178_F	0.9791	0.1598	0.9271	0.5445	0.9572	0.0861	0.8079	0.9752	0.9375	0.8392	0.1346	0.2148	0.9830	0.8670	0.6908
TFF2_P557_R	0.0000	0.9956	0.9956	0.7486	0.9927	0.2852	0.1858	0.9900	0.9788	0.0000	0.9941	0.9904	0.1175	0.9915	0.8616
TFPI2_E141_F	0.0000	0.0004	0.0000	0.0000	0.0000	0.0000	0.0000	0.0000	0.0007	0.0000	0.0000	0.0000	0.0000	0.0028	0.0000
TFPI2_P152_R	0.0866	0.3552	0.0172	0.0000	0.0184	0.5514	0.2046	0.0146	0.0305	0.3062	0.0189	0.3324	0.1688	0.0317	0.0000
TFPI2_P9_F	0.5792	0.0088	0.5279	0.7182	0.5039	0.6332	0.4438	0.0167	0.0135	0.6850	0.5275	0.0158	0.0200	0.0189	0.0000
TFRC_P414_R	0.3905	0.3707	0.0288	0.1518	0.0193	0.2068	0.0054	0.0182	0.0082	0.0945	0.0135	0.0056	0.0020	0.0027	0.0000
TGFA_P558_F	0.0000	0.0000	0.0017	0.0000	0.0000	0.0036	0.0003	0.0138	0.0149	0.0000	0.4317	0.0000	0.0050	0.0123	0.0000
TGFA_P642_R	0.0000	0.0000	0.0000	0.0000	0.0030	0.1394	0.0000	0.0072	0.0077	0.9785	0.0043	0.0029	0.0052	0.0060	0.0000
TGFB1_P833_R	0.0000	0.0000	0.6719	0.0000	0.0000	0.0000	0.0000	0.0012	0.9430	0.9921	0.0000	0.9920	0.0000	0.9837	0.0000
TGFB2_E226_R	0.0000	0.0000	0.0000	0.0000	0.0000	0.0000	0.0000	0.0000	0.0000	0.0000	0.0015	0.0000	0.0000	0.0000	0.0000
TGFB2_P632_F	0.0073	0.0136	0.0101	0.4986	0.0154	0.3287	0.0092	0.0181	0.0135	0.0114	0.0094	0.0110	0.2474	0.0166	0.0000
TGFB3_E58_R	0.9922	0.9957	0.8712	0.0000	0.7930	0.0000	0.9884	0.9877	0.9879	0.9944	0.9917	0.9893	0.0001	0.8269	0.7803
TGFBI_P173_F	0.1850	0.1119	0.0497	0.7122	0.1087	0.1162	0.0113	0.0127	0.8905	0.0555	0.0092	0.0267	0.0084	0.0125	0.0000
TGFBI_P31_R	0.0071	0.0000	0.0104	0.0000	0.0061	0.0000	0.0000	0.0086	0.0514	0.0091	0.2004	0.0081	0.0067	0.5891	0.2052
TGFBR3_E188_R	0.0046	0.0068	0.0018	0.0000	0.0000	0.0000	0.0052	0.0035	0.0030	0.0000	0.0032	0.0020	0.0000	0.0029	0.0000
TGFBR3_P429_F	0.0042	0.0000	0.0000	0.0000	0.0011	0.0000	0.0000	0.0000	0.0019	0.0000	0.0032	0.0000	0.0000	0.0038	0.0000
THBS1_E207_R	0.0000	0.0000	0.0000	0.0000	0.0000	0.0067	0.1748	0.0000	0.0055	0.0000	0.0056	0.0039	0.0122	0.0000	0.0000

THBS1_P500_F	0.0055	0.0128	0.0077	0.0000	0.0095	0.0111	0.0147	0.0183	0.0143	0.0089	0.0065	0.0231	0.0038	0.0101	0.0000
THBS2_E129_F	0.0032	0.0057	0.0000	0.0000	0.0024	0.1100	0.0028	0.0059	0.0079	0.0014	0.0027	0.0000	0.0028	0.0013	0.0000
THY1_P149_R	0.1412	0.1916	0.0235	0.0000	0.0263	0.3446	0.2602	0.0216	0.0296	0.3436	0.3218	0.6395	0.0101	0.0169	0.0000
TIAM1_P188_R	0.0030	0.0041	0.0033	0.0000	0.0004	0.0000	0.0000	0.0095	0.0000	0.0000	0.0017	0.0022	0.0000	0.0026	0.0000
TIMP1_E254_R	0.8030	0.0068	0.0066	0.0000	0.0094	0.0566	0.0182	0.0164	0.0152	0.0116	0.0085	0.0154	0.0151	0.9677	0.7087
TIMP1_P615_R	0.0000	0.0000	0.0000	0.0000	0.0000	0.0000	0.0000	0.1421	0.0000	0.0000	0.9836	0.0000	0.9624	0.0000	0.0115
TIMP2_E394_R	0.0000	0.0767	0.0015	0.0000	0.0000	0.0000	0.0559	0.1191	0.0141	0.0015	0.0081	0.0000	0.0026	0.0987	0.0000
TIMP2_P267_F	0.0055	0.0076	0.0030	0.0022	0.0008	0.0083	0.0060	0.0032	0.0024	0.0044	0.0000	0.0032	0.0007	0.0000	0.0000
TIMP3_P1114_R	0.9936	0.7403	0.9922	0.0000	0.9877	0.3668	0.3713	0.9879	0.9824	0.1307	0.9933	0.9895	0.9899	0.9870	0.9910
TIMP3_P690_R	0.0000	0.0000	0.9931	0.0000	0.9932	0.0000	0.0000	0.9846	0.9880	0.9952	0.9919	0.9889	0.6185	0.9918	0.9722
TIMP3_seq_7_S38_F	0.2197	0.2248	0.3185	0.5966	0.0064	0.6055	0.0000	0.0103	0.0093	0.0550	0.1465	0.1435	0.0060	0.0052	0.2951
TJP1_P326_R	0.0817	0.0823	0.0129	0.0179	0.1245	0.0815	0.0099	0.0133	0.0136	0.0247	0.0154	0.0258	0.0096	0.0191	0.0000
TJP1_P390_F	0.0047	0.0553	0.0111	0.0000	0.0042	0.0000	0.0000	0.0122	0.0089	0.0000	0.0072	0.0049	0.0102	0.0018	0.0000
TK1_E47_F	0.4157	0.0264	0.2337	0.2363	0.1325	0.4297	0.4087	0.0578	0.0422	0.0169	0.2796	0.3684	0.0327	0.0103	0.0000
TK1_P62_R	0.0025	0.0111	0.0070	0.0000	0.0000	0.4550	0.3628	0.0108	0.0078	0.0000	0.0090	0.0115	0.0000	0.0079	0.0000
TMEFF1_E180_R	0.0133	0.0000	0.0095	0.0000	0.0000	0.0000	0.0000	0.0000	0.0097	0.0000	0.0000	0.0000	0.0040	0.0069	0.0000
TMEFF1_P234_F	0.0000	0.0000	0.0062	0.0000	0.0000	0.0000	0.0000	0.4234	0.0032	0.0000	0.0049	0.0038	0.0000	0.0000	0.0000
TMEFF2_P152_R	0.0624	0.3437	0.3178	0.1420	0.3483	0.3959	0.3844	0.0860	0.0617	0.3701	0.0574	0.0946	0.0563	0.0757	0.7989
TMEFF2_P210_R	0.0000	0.0000	0.0574	0.0000	0.0000	0.0000	0.0036	0.0000	0.0000	0.0000	0.0000	0.0000	0.0000	0.0000	0.0000
TMEM63A_E63_F	0.0000	0.0000	0.0024	0.0000	0.0000	0.0000	0.0015	0.0000	0.0000	0.0000	0.0000	0.0026	0.0000	0.0000	0.0000
TMPRSS4_E83_F	0.0107	0.0000	0.9915	0.0000	0.0908	0.0000	0.9945	0.4510	0.9840	0.0000	0.9895	0.9891	0.0819	0.9831	0.9852
TMPRSS4_P552_F	0.9929	0.6335	0.9923	0.3394	0.0188	0.7087	0.1337	0.0474	0.9220	0.4997	0.9925	0.8593	0.9901	0.0174	0.9548
TNC_P198_F	0.0000	0.0862	0.0027	0.0000	0.0000	0.0000	0.0000	0.0000	0.0000	0.0000	0.0000	0.0000	0.0000	0.6251	0.0000
TNC_P57_F	0.0120	0.0131	0.0109	0.2090	0.1758	0.6553	0.0083	0.0114	0.0104	0.0136	0.0084	0.0134	0.0109	0.4558	0.7431
TNF_P1084_F	0.6783	0.3617	0.0142	0.0000	0.5296	0.9971	0.2195	0.9885	0.9791	0.0247	0.9922	0.9902	0.3216	0.9927	0.9326
TNF_P158_F	0.0000	0.0000	0.0016	0.0000	0.0017	0.0000	0.0000	0.0055	0.0045	0.0000	0.0000	0.0068	0.0079	0.0000	0.5531
TNFRSF10A_P171_F	0.0065	0.1119	0.0798	0.0000	0.0092	0.2454	0.0804	0.0103	0.0106	0.0079	0.0065	0.0144	0.0083	0.0063	0.0000
TNFRSF10A_P91_F	0.0000	0.0000	0.0000	0.0000	0.0070	0.0000	0.0000	0.0064	0.0045	0.0000	0.0047	0.0033	0.0000	0.0053	0.0000
TNFRSF10B_P108_R	0.0184	0.0154	0.0907	0.0000	0.1113	0.0074	0.0637	0.0226	0.0251	0.0684	0.0195	0.0776	0.0140	0.0222	0.0085
TNFRSF10C_E109_F	0.0028	0.2078	0.0000	0.0000	0.0015	0.2198	0.1401	0.0050	0.0028	0.0064	0.0458	0.0073	0.0062	0.0057	0.0000
TNFRSF10D_E27_F	0.0000	0.0100	0.0013	0.0119	0.0040	0.0059	0.0055	0.0092	0.0068	0.0076	0.0053	0.0112	0.0028	0.0083	0.0000
TNFRSF10D_P70_F	0.0047	0.0076	0.0108	0.0000	0.0079	0.0875	0.0130	0.1382	0.0297	0.0144	0.0149	0.0228	0.0122	0.0351	0.0000
TNFRSF1B_E5_F	0.0000	0.0130	0.0000	0.0000	0.0000	0.0000	0.0000	0.0121	0.0131	0.0112	0.0112	0.9762	0.0097	0.0000	0.0000

TNFRSF1B_P167_F	0.0052	0.0021	0.0000	0.0000	0.0011	0.0000	0.0071	0.0058	0.0063	0.0017	0.0000	0.0033	0.9881	0.0019	0.0000
TNFSF10_E53_F	0.0803	0.1160	0.4934	0.0000	0.0032	0.0155	0.0000	0.0090	0.0143	0.5704	0.0052	0.4775	0.0155	0.0000	0.0000
TNFSF10_P2_R	0.0008	0.0000	0.0000	0.0000	0.0000	0.0000	0.0000	0.0083	0.0000	0.0000	0.0000	0.0000	0.0058	0.0000	0.0000
TNFSF8_E258_R	0.0000	0.0053	0.0000	0.0000	0.0012	0.0000	0.0000	0.0062	0.0045	0.0058	0.0054	0.0058	0.0000	0.0001	0.0000
TNFSF8_P184_F	0.0018	0.0000	0.0000	0.0000	0.0021	0.0031	0.0040	0.0056	0.0035	0.0000	0.0034	0.0067	0.0176	0.0025	0.0000
TNK1_P41_R	0.0238	0.0109	0.0092	0.2412	0.0229	0.4487	0.0103	0.0134	0.0229	0.0107	0.5649	0.0393	0.0036	0.0045	0.6841
TP73_E155_F	0.4352	0.0094	0.0209	0.6125	0.5410	0.6639	0.0081	0.0381	0.0102	0.3329	0.0110	0.0129	0.4474	0.0076	0.0000
TP73_P496_F	0.0000	0.0076	0.0013	0.0000	0.0047	0.0041	0.0000	0.0040	0.5259	0.0077	0.0000	0.0078	0.0079	0.0000	0.0000
TP73_P945_F	0.0090	0.0087	0.1797	0.0000	0.0039	0.0059	0.0097	0.0110	0.0084	0.0000	0.0081	0.0112	0.0448	0.0100	0.0000
TPEF_seq_44_S36_F	0.0052	0.0000	0.0000	0.0000	0.0031	0.0000	0.0000	0.0142	0.0067	0.0095	0.0000	0.0078	0.0000	0.0000	0.0000
TPEF_seq_44_S88_R	0.0083	0.0048	0.0056	0.0000	0.0064	0.0813	0.0319	0.0092	0.5013	0.0148	0.0082	0.2121	0.0841	0.5827	0.0000
TRIM29_P261_F	0.0413	0.9948	0.8020	0.9775	0.9897	0.9057	0.0307	0.9614	0.9827	0.9939	0.7746	0.9882	0.7546	0.6019	0.9704
TRIP6_E33_F	0.0083	0.2820	0.0432	0.0000	0.2148	0.0000	0.0000	0.0138	0.0108	0.0069	0.2233	0.1907	0.0052	0.0084	0.0000
TSG101_P257_R	0.0000	0.0057	0.0000	0.0000	0.0000	0.0000	0.0030	0.0024	0.0012	0.0024	0.0071	0.0000	0.0000	0.0000	0.0000
TUBB3_E91_F	0.0000	0.0024	0.0022	0.1580	0.0022	0.0000	0.0000	0.0011	0.2299	0.0012	0.0007	0.0009	0.0016	0.2276	0.1037
TUBB3_P364_F	0.0000	0.0000	0.0000	0.0000	0.0000	0.0000	0.0000	0.0000	0.0000	0.0000	0.0000	0.0000	0.0000	0.0000	0.0000
TUBB3_P721_R	0.0000	0.0000	0.0026	0.0000	0.0000	0.0000	0.0000	0.0000	0.0084	0.0012	0.0015	0.0045	0.0000	0.0000	0.0000
TUSC3_E29_R	0.0077	0.0000	0.0102	0.0000	0.0271	0.3595	0.0000	0.0069	0.0086	0.0106	0.0531	0.0084	0.0034	0.0055	0.0000
TUSC3_P85_R	0.0000	0.0000	0.0059	0.0000	0.0029	0.0000	0.0000	0.0121	0.0167	0.0156	0.0180	0.0063	0.0000	0.0021	0.0000
TWIST1_E117_R	0.0000	0.0000	0.0000	0.0000	0.0000	0.0000	0.0030	0.0000	0.0000	0.0000	0.0000	0.0000	0.0024	0.0000	0.0000
TWIST1_P355_R	0.0063	0.0058	0.0142	0.0000	0.2625	0.0000	0.0000	0.0015	0.0093	0.0000	0.0004	0.1605	0.0036	0.0064	0.0000
TWIST1_P44_R	0.0000	0.0000	0.0000	0.0000	0.0000	0.0000	0.0000	0.0000	0.0000	0.0000	0.0011	0.0000	0.0000	0.0000	0.0000
TYRO3_P501_F	0.0000	0.0000	0.0001	0.0000	0.0000	0.0000	0.0034	0.0000	0.0033	0.0000	0.0000	0.0036	0.0034	0.0018	0.0000
UBA52_P293_R	0.0000	0.0037	0.0000	0.0000	0.0000	0.0000	0.0000	0.0000	0.0034	0.0000	0.0000	0.0096	0.0000	0.0003	0.0000
UGT1A1_E11_F	0.9440	0.0000	0.0000	0.0000	0.0000	0.0000	0.0000	0.9862	0.9852	0.0000	0.9921	0.9861	0.9350	0.9871	0.9730
UGT1A1_P564_R	0.9477	0.6649	0.9557	0.3289	0.9318	0.6901	0.8416	0.9061	0.9077	0.6708	0.9217	0.9630	0.9350	0.8976	0.8635
UGT1A7_P751_R	0.9913	0.9945	0.0000	0.0000	0.9914	0.0000	0.9927	0.9875	0.9853	0.0000	0.9922	0.9867	0.9922	0.9869	0.0000
UNG_P170_F	0.0008	0.1234	0.0038	0.0000	0.0067	0.0000	0.0090	0.0070	0.0068	0.0109	0.0383	0.0087	0.0035	0.0046	0.0000
USP29_E274_F	0.9929	0.9950	0.9906	0.0000	0.9924	0.9897	0.9941	0.9849	0.9848	0.0000	0.9914	0.9445	0.9921	0.9838	0.8925
USP29_P282_R	0.0000	0.0000	0.9921	0.0000	0.9909	0.0000	0.0000	0.8233	0.9853	0.0000	0.0000	0.9881	0.9643	0.9869	0.9883
VAMP8_P114_F	0.0129	0.0095	0.0136	0.0000	0.2805	0.0625	0.1421	0.0327	0.0123	0.0112	0.0101	0.0114	0.0126	0.0100	0.0000
VAV1_E9_F	0.0000	0.0000	0.0000	0.0000	0.0000	0.0000	0.7440	0.0000	0.0128	0.0222	0.0053	0.0092	0.0075	0.0044	0.0000
VAV1_P317_F	0.0033	0.0000	0.0000	0.0000	0.0000	0.0000	0.0000	0.0023	0.0051	0.0059	0.0000	0.0000	0.0013	0.0018	0.0000

VAV2_E58_F	0.1587	0.0333	0.1639	0.0000	0.1960	0.1726	0.1921	0.0171	0.0143	0.0142	0.0212	0.0983	0.1045	0.1040	0.0000
VAV2_P1182_F	0.0000	0.0000	0.0000	0.0000	0.9858	0.0000	0.0000	0.0049	0.0070	0.0000	0.0000	0.0008	0.0000	0.0076	0.0000
VBP1_P12_R	0.0295	0.0000	0.0000	0.0000	0.0000	0.0171	0.0000	0.0044	0.0003	0.0221	0.0286	0.0107	0.9735	0.0000	0.0000
VEGFB_P658_F	0.0064	0.0055	0.0048	0.0026	0.0067	0.0071	0.3226	0.0101	0.0090	0.0060	0.0346	0.0094	0.7680	0.0052	0.0000
VIM_P811_R	0.0000	0.0000	0.0000	0.0000	0.0000	0.0000	0.0000	0.0000	0.0000	0.0000	0.0040	0.0000	0.0011	0.0068	0.0000
WEE1_P924_R	0.0000	0.0509	0.9923	0.0000	0.0000	0.0000	0.9950	0.9236	0.9103	0.0000	0.0000	0.7337	0.9912	0.6975	0.9769
WNT2_E109_R	0.0000	0.0000	0.0000	0.0000	0.0000	0.0000	0.0000	0.0000	0.0000	0.0000	0.0000	0.0000	0.0000	0.0000	0.0000
WNT2_P217_F	0.1963	0.2358	0.2094	0.6473	0.0209	0.0110	0.0341	0.0313	0.0262	0.0279	0.2072	0.0294	0.2029	0.0275	0.0103
WNT2B_P1185_R	0.4902	0.0000	0.0138	0.0000	0.0150	0.0000	0.0227	0.0163	0.0238	0.0000	0.0191	0.0150	0.0000	0.0408	0.0000
WNT5A_E43_F	0.0165	0.2159	0.0483	0.0563	0.7264	0.2707	0.0112	0.0132	0.0139	0.0184	0.0124	0.0125	0.0185	0.0153	0.0000
WNT5A_P655_F	0.0045	0.0000	0.0043	0.0000	0.0026	0.0058	0.0000	0.5126	0.0063	0.0000	0.0074	0.0045	0.0020	0.0051	0.0000
WNT8B_E487_F	0.4761	0.0138	0.2937	0.6900	0.4918	0.9943	0.0299	0.0244	0.9346	0.0201	0.4120	0.5890	0.3773	0.3631	0.0000
WNT8B_P216_R	0.0000	0.0000	0.0000	0.0000	0.0000	0.0000	0.0000	0.9862	0.9816	0.0000	0.0000	0.9844	0.0000	0.9787	0.9646
WRN_E57_F	0.2539	0.2122	0.5556	0.0224	0.0099	0.1574	0.2400	0.4195	0.0210	0.2492	0.0196	0.0133	0.3640	0.0234	0.9699
WRN_P969_F	0.0070	0.0074	0.0000	0.0000	0.9896	0.3328	0.0000	0.9825	0.9696	0.0000	0.0000	0.9879	0.0000	0.8146	0.9876
WT1_E32_F	0.0068	0.0108	0.0084	0.0000	0.0000	0.0000	0.5083	0.0192	0.0072	0.0000	0.0036	0.0000	0.0072	0.0190	0.0000
WT1_P853_F	0.0030	0.0076	0.0047	0.0000	0.0033	0.0060	0.0000	0.0024	0.0042	0.0000	0.0000	0.0083	0.0039	0.0021	0.0000
XRCC1_P681_R	0.9921	0.0223	0.9877	0.0000	0.8705	0.9955	0.0000	0.9811	0.7805	0.0069	0.9917	0.9885	0.0081	0.9849	0.9619
XRCC2_P1077_F	0.0000	0.9935	0.3067	0.0000	0.0000	0.9964	0.7527	0.9902	0.9855	0.0032	0.9904	0.9909	0.0000	0.9918	0.9882
YES1_P600_F	0.0000	0.2416	0.0091	0.0000	0.0088	0.4837	0.0453	0.0165	0.0149	0.0141	0.1825	0.0176	0.0210	0.0206	0.0000
ZIM3_E203_F	0.0000	0.0000	0.9913	0.0000	0.9905	0.0000	0.9926	0.9860	0.9856	0.0000	0.9910	0.9909	0.9897	0.9883	0.8720
ZIM3_P451_R	0.9896	0.0000	0.9889	0.0000	0.9909	0.9968	0.9918	0.9852	0.9852	0.9930	0.9931	0.9858	0.9879	0.9862	0.9899
ZIM3_P718_R	0.0000	0.0000	0.0000	0.0000	0.0000	0.0019	0.0000	0.9859	0.9857	0.0000	0.9916	0.8033	0.0008	0.9821	0.9155
ZMYND10_E77_R	0.0000	0.0034	0.0019	0.0000	0.0000	0.0000	0.0022	0.0001	0.0000	0.0012	0.0000	0.8672	0.0000	0.0032	0.0000
ZNF215_P71_R	0.9922	0.0309	0.0048	0.0000	0.0143	0.0042	0.0059	0.0069	0.4338	0.0000	0.0671	0.0070	0.0074	0.0037	0.0000
ZNF264_E48_R	0.0000	0.0000	0.0000	0.0000	0.0000	0.0000	0.0023	0.0000	0.0000	0.0070	0.0000	0.0000	0.0000	0.0000	0.0000
ZNFN1A1_E102_F	0.9826	0.2710	0.1093	0.0000	0.7436	0.0000	0.4674	0.0294	0.9216	0.0000	0.9876	0.4258	0.9859	0.8596	0.9756
ZNFN1A1_P179_F	0.0000	0.9951	0.9941	0.4896	0.1650	0.0000	0.9946	0.9866	0.9845	0.9942	0.9925	0.9935	0.9906	0.9886	0.9522

TargetID	BWS47	BWS48	BWS49	BWS50	BWS51	CONTROL_1	CONTROL_2	CONTROL_3a	CONTROL_3b	CONTROL_4	CONTROL_5	CONTROL_6
AATK_E63_R	0.9867	0.0707	0.9121	0.9833	0.0143	0.9851	0.0000	0.9883	0.9881	0.0000	0.9876	0.1147
ABCA1_E120_R	0.9793	0.9922	0.9849	0.9833	0.9802	0.9847	0.9950	0.9815	0.9776	0.9906	0.9879	0.9922
ABCA1_P45_F	0.9836	0.9887	0.9865	0.9835	0.0000	0.9814	0.9927	0.0000	0.9840	0.9886	0.9771	0.0000
ABCB4_E429_F	0.0017	0.0191	0.0016	0.0054	0.0053	0.0043	0.0000	0.0046	0.0011	0.0042	0.0069	0.0011
ABCC2_P88_F	0.0160	0.0200	0.0186	0.2939	0.0217	0.1765	0.1587	0.0379	0.7520	0.0784	0.0263	0.9906
ABCC5_P444_F	0.0094	0.0093	0.0083	0.0088	0.0104	0.1014	0.0051	0.1738	0.1168	0.0065	0.0087	0.0058
ABCG2_P178_R	0.5071	0.4678	0.0333	0.4095	0.4459	0.0267	0.0508	0.6394	0.0322	0.5401	0.0549	0.3815
ABCG2_P310_R	0.9855	0.9903	0.9882	0.9715	0.9802	0.9794	0.0000	0.9865	0.9846	0.0000	0.9802	0.9678
ABL1_P53_F	0.9897	0.7812	0.9888	0.9751	0.9742	0.9786	0.9882	0.9927	0.9846	0.9862	0.4724	0.9651
ABL2_P459_R	0.6818	0.0000	0.0000	0.0000	0.0000	0.1443	0.0079	0.0017	0.0000	0.0024	0.0000	0.0000
ABO_E110_F	0.7102	0.9892	0.0055	0.0292	0.0444	0.2694	0.0000	0.8201	0.0050	0.0075	0.0155	0.0044
ABO_P312_F	0.0071	0.0103	0.2329	0.2342	0.3203	0.1037	0.0096	0.0110	0.4583	0.0113	0.3331	0.0664
ACTG2_P455_R	0.0000	0.0000	0.0000	0.0000	0.0000	0.0000	0.8437	0.0000	0.0000	0.0888	0.0055	0.2316
ACVR1_P983_F	0.0000	0.0000	0.0000	0.0014	0.0050	0.0000	0.0019	0.0000	0.0000	0.0022	0.0000	0.0022
ACVR1B_E497_R	0.0073	0.0057	0.0170	0.0075	0.0084	0.0063	0.0078	0.0000	0.0001	0.0049	0.0065	0.1800
ACVR1B_P572_R	0.0138	0.0101	0.0768	0.0242	0.0216	0.0114	0.0138	0.0170	0.0106	0.0146	0.0160	0.0121
ACVR1C_P115_R	0.0049	0.0046	0.0000	0.0036	0.0070	0.0000	0.0068	0.0000	0.0019	0.0058	0.0054	0.0000
ACVR1C_P363_F	0.9899	0.9883	0.9557	0.9822	0.9921	0.9857	0.9961	0.9176	0.9894	0.9900	0.9928	0.9919
ACVR2B_E27_R	0.9855	0.9832	0.7840	0.9814	0.8076	0.9711	0.9931	0.6073	0.5586	0.9818	0.0284	0.0373
ACVR2B_P676_F	0.0000	0.0000	0.0000	0.0000	0.0000	0.0000	0.0000	0.0000	0.0000	0.0000	0.0000	0.0000
ADAMTS12_P250_R	0.0008	0.0000	0.0017	0.0000	0.0087	0.0000	0.0000	0.0038	0.0000	0.0000	0.0040	0.0000
ADCYAP1_E163_R	0.0000	0.1341	0.0040	0.0110	0.0841	0.0062	0.0000	0.0000	0.0051	0.0085	0.0064	0.0679
ADCYAP1_P398_F	0.9848	0.2664	0.9830	0.9112	0.9614	0.9728	0.9557	0.8948	0.9880	0.9847	0.3783	0.9272
ADCYAP1_P455_R	0.0241	0.0113	0.0144	0.0791	0.0112	0.0644	0.0155	0.7583	0.2390	0.0206	0.5657	0.0189
AFF3_P122_F	0.0031	0.0063	0.0034	0.1982	0.0092	0.0722	0.0070	0.0079	0.0006	0.0043	0.0086	0.0000
AFF3_P808_F	0.0000	0.0044	0.0027	0.4923	0.5419	0.2218	0.0135	0.0000	0.0038	0.0000	0.0168	0.0000
AFP_P824_F	0.2970	0.2823	0.0106	0.6047	0.9867	0.6055	0.1249	0.0074	0.7848	0.0122	0.0229	0.1551
AGTR1_P154_F	0.0000	0.0000	0.0000	0.0000	0.0000	0.0000	0.0039	0.0009	0.0000	0.0000	0.0000	0.0000
AGTR1_P41_F	0.0208	0.0169	0.0258	0.0176	0.4324	0.0155	0.5808	0.0164	0.0184	0.0394	0.0197	0.0084
AHR_E103_F	0.9700	0.9713	0.9580	0.9749	0.9892	0.9610	0.0000	0.0000	0.0000	0.0000	0.0000	0.0000
AHR_P166_R	0.7842	0.6125	0.8008	0.7968	0.8249	0.7836	0.4679	0.6423	0.5894	0.7501	0.6195	0.2325

AIM2_E208_F	0.0045	0.0000	0.0108	0.0147	0.0132	0.0090	0.0000	0.0094	0.0078	0.0159	0.0120	0.0024
AKT1_P310_R	0.0126	0.0000	0.3952	0.2166	0.4632	0.1933	0.0100	0.0177	0.0009	0.0067	0.5081	0.9890
ALK_P28_F	0.3095	0.0055	0.3830	0.4395	0.5159	0.2382	0.0135	0.0760	0.0033	0.0180	0.0094	0.0069
ALOX12_E85_R	0.0000	0.0000	0.0000	0.0003	0.0018	0.0552	0.0032	0.0053	0.0000	0.0020	0.0058	0.0000
ALOX12_P223_R	0.2047	0.8946	0.9823	0.9781	0.8614	0.9800	0.6500	0.0000	0.9805	0.9447	0.9852	0.0300
APBA1_E99_R	0.0000	0.9763	0.0000	0.0000	0.0000	0.9089	0.0000	0.0000	0.0000	0.0000	0.0000	0.0000
APBA1_P644_F	0.0000	0.0000	0.0000	0.0000	0.0000	0.0000	0.0000	0.0000	0.0000	0.0000	0.0000	0.0000
APBA2_P227_F	0.8589	0.0000	0.4674	0.0045	0.0049	0.0685	0.0000	0.0042	0.0037	0.0022	0.0073	0.0000
APC_P280_R	0.0000	0.0000	0.2498	0.9796	0.9804	0.9823	0.9949	0.9864	0.9827	0.0000	0.9821	0.0000
APOA1_P75_F	0.0021	0.4029	0.0081	0.0000	0.0000	0.0000	0.1734	0.0000	0.0032	0.0000	0.0000	0.0000
APOC1_P406_R	0.6583	0.0080	0.2989	0.1391	0.0394	0.3689	0.0085	0.0466	0.0137	0.0073	0.8035	0.6256
APP_E8_F	0.0000	0.0011	0.0046	0.0044	0.0050	0.0015	0.0000	0.0032	0.0000	0.9093	0.0228	0.0031
APP_P179_R	0.0310	0.9712	0.0243	0.0342	0.0170	0.0223	0.0202	0.0464	0.0160	0.0222	0.0723	0.1000
AR_P189_R	0.1536	0.1469	0.0063	0.0128	0.1212	0.0116	0.1547	0.2274	0.2628	0.0089	0.0000	0.0619
AREG_E25_F	0.0000	0.0000	0.0000	0.0000	0.0061	0.0000	0.0000	0.0063	0.0000	0.0052	0.0043	0.0000
ARHGDIB_P148_R	0.0251	0.0171	0.0189	0.0282	0.0275	0.0754	0.0175	0.0599	0.0287	0.0314	0.0284	0.0179
ARNT_P238_R	0.0095	0.0061	0.0031	0.0071	0.0112	0.0039	0.1478	0.0057	0.0026	0.0095	0.0086	0.1339
ASB4_P391_F	0.0077	0.0000	0.0042	0.4329	0.0020	0.0039	0.0000	0.0000	0.2859	0.0000	0.0000	0.0273
ASB4_P52_R	0.0017	0.0056	0.0019	0.0830	0.0076	0.1591	0.0000	0.0067	0.0000	0.0067	0.0067	0.0048
ASCL1_E24_F	0.0000	0.0000	0.0000	0.0000	0.0021	0.0000	0.0000	0.0000	0.0000	0.0000	0.0000	0.0000
ASCL1_P747_F	0.0075	0.0067	0.0037	0.0048	0.1051	0.0042	0.0000	0.0000	0.0043	0.0043	0.0094	0.1086
ASCL2_E76_R	0.0000	0.0000	0.0000	0.0000	0.0000	0.0000	0.0081	0.0000	0.0000	0.0036	0.0038	0.0012
ASCL2_P360_F	0.0000	0.0000	0.0000	0.0000	0.0105	0.0000	0.0000	0.0000	0.0000	0.0000	0.0000	0.0000
ASCL2_P609_R	0.0000	0.0021	0.0000	0.0000	0.0020	0.0000	0.0161	0.0069	0.0000	0.0087	0.0000	0.0069
ATP10A_P147_F	0.0075	0.0000	0.0092	0.0072	0.2578	0.0039	0.0000	0.0000	0.0060	0.0033	0.0030	0.0000
ATP10A_P524_R	0.0115	0.0057	0.0073	0.0094	0.0196	0.0077	0.0000	0.0187	0.0109	0.0163	0.0154	0.1059
AXIN1_P995_R	0.0500	0.0092	0.0300	0.0205	0.4188	0.0118	0.0264	0.0177	0.0270	0.0157	0.0220	0.0108
AXL_E61_F	0.0055	0.0081	0.0021	0.0472	0.0048	0.1166	0.0020	0.0046	0.0000	0.0020	0.0030	0.0001
BAX_E281_R	0.0968	0.0088	0.0228	0.0154	0.5497	0.0158	0.4773	0.0140	0.0146	0.0248	0.5218	0.0224
BCAM_E100_R	0.0087	0.0700	0.0108	0.1170	0.7114	0.0090	0.0095	0.0084	0.0053	0.1848	0.5406	0.0052
BCAM_P205_F	0.0149	0.1865	0.0174	0.0172	0.0128	0.0140	0.0153	0.0091	0.4269	0.0140	0.0139	0.4217
BCAP31_P1131_F	0.0158	0.7488	0.0161	0.0128	0.0160	0.0101	0.0265	0.0161	0.0195	0.0190	0.0252	0.5821
BCL2L2_E172_F	0.0000	0.0022	0.0000	0.0000	0.0059	0.0000	0.0000	0.0000	0.0000	0.0017	0.0060	0.0000

BCL2L2_P280_F	0.0048	0.0000	0.0081	0.0077	0.0098	0.0037	0.8696	0.0000	0.0154	0.0000	0.0081	0.0000
BCL3_E71_F	0.0004	0.0054	0.0002	0.0024	0.0049	0.0015	0.0075	0.0055	0.0000	0.0021	0.0037	0.0000
BCL3_P1038_R	0.0075	0.0000	0.0074	0.1998	0.0101	0.0082	0.1022	0.0060	0.0048	0.0000	0.5549	0.0000
BCL6_P248_R	0.0038	0.0000	0.0013	0.0040	0.0051	0.0045	0.0039	0.0000	0.0000	0.0069	0.0073	0.0000
BDNF_E19_R	0.0021	0.0594	0.0122	0.0160	0.0672	0.0439	0.0078	0.0630	0.0211	0.0040	0.0055	0.0024
BDNF_P259_R	0.9890	0.9854	0.9881	0.9842	0.4806	0.9790	0.9932	0.8121	0.9894	0.9203	0.9002	0.9918
BGN_E282_R	0.0002	0.0008	0.0060	0.0044	0.0073	0.0019	0.0097	0.0095	0.0005	0.0054	0.0042	0.0047
BGN_P333_R	0.0051	0.1329	0.0040	0.1231	0.0072	0.4523	0.0067	0.0042	0.0449	0.0077	0.0054	0.7562
BIRC4_P122_R	0.6600	0.0057	0.0155	0.2247	0.4168	0.0949	0.1806	0.0140	0.0052	0.0155	0.0178	0.3491
BIRC5_E89_F	0.0118	0.0277	0.0160	0.0182	0.0184	0.0219	0.0482	0.0215	0.0136	0.0188	0.0818	0.0991
BLK_P14_F	0.0063	0.0709	0.0164	0.0078	0.0095	0.0075	0.1006	0.0142	0.0062	0.0150	0.4529	0.0083
BMP2_E48_R	0.0101	0.0178	0.0592	0.0077	0.0113	0.1243	0.0195	0.0077	0.0133	0.0155	0.0114	0.2864
BMP2_P1201_F	0.0007	0.0000	0.0000	0.0028	0.0000	0.0827	0.0000	0.0000	0.0000	0.0000	0.0004	0.0021
BMP3_E147_F	0.0000	0.0000	0.0000	0.0000	0.0008	0.0000	0.0000	0.0021	0.0000	0.0018	0.0000	0.0000
BMP3_P56_R	0.0000	0.0000	0.0053	0.0021	0.0045	0.0036	0.0000	0.0000	0.0000	0.0024	0.0000	0.0000
BMP4_P199_R	0.0205	0.9583	0.0118	0.1905	0.0180	0.2647	0.0129	0.0195	0.0196	0.7958	0.0341	0.1017
BMP6_P398_F	0.9685	0.8724	0.8980	0.9838	0.9844	0.9792	0.9930	0.9913	0.7875	0.8168	0.9888	0.9831
BMPR1A_P956_F	0.0984	0.2280	0.0081	0.0159	0.2633	0.0114	0.0130	0.0133	0.0244	0.0131	0.0197	0.0081
BMPR2_E435_F	0.0000	0.0000	0.0000	0.0000	0.0000	0.0000	0.0000	0.0000	0.0000	0.0000	0.0000	0.0000
BMPR2_P1271_F	0.0019	0.0114	0.0185	0.2049	0.0049	0.0159	0.0000	0.0156	0.0979	0.0155	0.0115	0.1007
BSG_P211_R	0.0553	0.4992	0.4001	0.0694	0.0688	0.0573	0.5198	0.1158	0.0712	0.7088	0.0919	0.1117
BTK_P105_F	0.0127	0.0105	0.0129	0.0146	0.0179	0.0137	0.0094	0.0089	0.0106	0.0386	0.0181	0.1028
C20orf47_P225_R	0.1457	0.0000	0.0069	0.0097	0.0079	0.0033	0.0075	0.0094	0.0071	0.0000	0.0000	0.0000
CALCA_E174_R	0.1359	0.1317	0.1076	0.0434	0.2830	0.0410	0.7007	0.0823	0.0564	0.6676	0.0746	0.5877
CAPG_E228_F	0.0035	0.0000	0.0012	0.0677	0.0041	0.0018	0.0040	0.0000	0.0000	0.0031	0.0130	0.0008
CASP10_E139_F	0.0030	0.0000	0.9710	0.0068	0.0000	0.0006	0.0885	0.0000	0.0022	0.0049	0.0099	0.0000
CASP10_P186_F	0.9829	0.0000	0.7792	0.9788	0.9895	0.9552	0.0000	0.9621	0.9134	0.9865	0.9149	0.9888
CASP2_P192_F	0.0223	0.3977	0.0715	0.0290	0.0477	0.0228	0.0659	0.5257	0.0380	0.0376	0.0344	0.3543
CASP3_P420_R	0.0000	0.0056	0.0046	0.0019	0.0074	0.0014	0.0053	0.0050	0.0000	0.0096	0.0000	0.0000
CASP6_P201_F	0.9809	0.9823	0.8206	0.6057	0.9840	0.9639	0.9922	0.1208	0.9769	0.7085	0.9751	0.0105
CASP6_P230_R	0.0091	0.0245	0.9859	0.0089	0.0155	0.3023	0.0304	0.0674	0.0045	0.0087	0.0149	0.0178
CAV1_P130_R	0.2138	0.0000	0.0000	0.0041	0.0081	0.0014	0.2319	0.0078	0.0000	0.0101	0.0117	0.1416
CAV1_P169_F	0.6241	0.0236	0.0208	0.0168	0.0325	0.0173	0.1603	0.0284	0.0167	0.0270	0.0233	0.0205

CAV2_E33_R	0.0145	0.0126	0.1649	0.2359	0.1144	0.1187	0.1390	0.0102	0.0137	0.0200	0.7283	0.0107
CCKBR_P361_R	0.0114	0.0076	0.0093	0.0113	0.0118	0.0104	0.0122	0.0097	0.0059	0.0117	0.0201	0.0100
CCKBR_P480_F	0.0011	0.0000	0.0011	0.0000	0.0039	0.0000	0.0000	0.0014	0.0000	0.0059	0.0059	0.0035
CCNA1_E7_F	0.9791	0.0000	0.9792	0.9739	0.9855	0.9799	0.9923	0.0000	0.9816	0.9822	0.9782	0.9856
CCNA1_P216_F	0.0000	0.0000	0.0000	0.0000	0.0000	0.0002	0.0000	0.0000	0.0028	0.0006	0.0000	0.0003
CCNC_P132_R	0.6758	0.0000	0.0000	0.0090	0.0000	0.3245	0.0000	0.0059	0.0000	0.0013	0.0000	0.0000
CCND1_E280_R	0.5400	0.0014	0.0000	0.4822	0.0000	0.2338	0.0000	0.0008	0.0000	0.5582	0.8404	0.0002
CCND1_P343_R	0.0000	0.0000	0.0000	0.0000	0.0000	0.0000	0.0000	0.0000	0.0000	0.0000	0.0000	0.0000
CCND2_P887_F	0.0000	0.0000	0.0000	0.0000	0.0008	0.0000	0.0000	0.0000	0.0000	0.0007	0.0000	0.0000
CCND2_P898_R	0.0836	0.1802	0.9688	0.8383	0.1116	0.6953	0.9907	0.9289	0.9854	0.0917	0.8685	0.5145
CCNE1_P683_F	0.0000	0.0000	0.0005	0.0000	0.0013	0.0435	0.0000	0.0000	0.0000	0.0000	0.0005	0.0000
CD1A_P414_R	0.0051	0.0088	0.0061	0.0105	0.0099	0.0080	0.0487	0.0295	0.8004	0.0419	0.0129	0.0025
CD2_P68_F	0.9886	0.0095	0.3075	0.6413	0.5140	0.6137	0.9917	0.0087	0.0614	0.2836	0.1777	0.6777
CD34_P339_R	0.0083	0.3197	0.0141	0.0213	0.0151	0.0148	0.0145	0.0179	0.2958	0.0134	0.0156	0.0109
CD34_P780_R	0.8555	0.5650	0.8872	0.8190	0.7135	0.7985	0.9862	0.8703	0.8263	0.8356	0.9017	0.9554
CD40_E58_R	0.0014	0.0000	0.0001	0.0010	0.0000	0.0035	0.0000	0.0000	0.0022	0.0055	0.0049	0.0059
CD40_P372_R	0.0255	0.2046	0.0156	0.1365	0.0420	0.0192	0.0143	0.0336	0.0179	0.0271	0.0293	0.0151
CD44_E26_F	0.0000	0.0000	0.0000	0.0021	0.0051	0.0000	0.0000	0.1253	0.0000	0.0018	0.0016	0.0000
CD44_P87_F	0.8616	0.0000	0.9362	0.2119	0.1607	0.8885	0.0000	0.0084	0.0000	0.0043	0.0250	0.0024
CD86_P3_F	0.0000	0.0000	0.0000	0.0000	0.0000	0.0000	0.0000	0.0037	0.0000	0.0000	0.0000	0.0023
CDC25B_E83_F	0.0000	0.0000	0.0000	0.0000	0.0003	0.0000	0.0055	0.0000	0.0000	0.0000	0.0000	0.0014
CDC25B_P11_R	0.0063	0.0000	0.0000	0.0085	0.0196	0.0099	0.0000	0.0078	0.0000	0.0018	0.0124	0.0000
CDH1_P52_R	0.0000	0.0000	0.0000	0.0009	0.0000	0.1044	0.0000	0.0000	0.0000	0.0008	0.0000	0.0031
CDH11_E102_R	0.0010	0.0000	0.0025	0.1510	0.0045	0.1046	0.0000	0.0333	0.0013	0.0048	0.0000	0.0149
CDH11_P203_R	0.8748	0.0268	0.0219	0.0159	0.0211	0.2027	0.0129	0.0165	0.0119	0.0182	0.0193	0.0131
CDH11_P354_R	0.0042	0.0057	0.2478	0.0383	0.0059	0.0023	0.0081	0.0063	0.0008	0.0000	0.0074	0.5198
CDH13_E102_F	0.9853	0.9903	0.9862	0.9846	0.0038	0.9839	0.0000	0.9912	0.9869	0.9866	0.9866	0.9920
CDH17_E31_F	0.0239	0.0000	0.0039	0.3357	0.0324	0.0115	0.1867	0.0447	0.0478	0.0105	0.0140	0.0059
CDH17_P532_F	0.9873	0.9868	0.9847	0.9767	0.9682	0.9810	0.9955	0.1524	0.9877	0.9539	0.9864	0.9910
CDH3_E100_R	0.0210	0.0276	0.0223	0.0255	0.0410	0.0244	0.0250	0.0280	0.0234	0.0266	0.0236	0.1947
CDH3_P87_R	0.0017	0.0034	0.0011	0.0001	0.0060	0.3256	0.0000	0.0012	0.0000	0.0039	0.0112	0.0026
CDK10_E74_F	0.9864	0.0844	0.9828	0.8958	0.4910	0.7699	0.7833	0.0249	0.0214	0.9880	0.0541	0.4622
CDK2_P330_R	0.0000	0.0000	0.9632	0.7865	0.8148	0.9653	0.0000	0.9810	0.9733	0.0000	0.9343	0.0047

CDK6_E256_F	0.0000	0.1107	0.0051	0.2224	0.3680	0.3224	0.0046	0.0000	0.0006	0.0096	0.0091	0.0000
CDK6_P291_R	0.0219	0.0205	0.0161	0.0206	0.0157	0.0171	0.2240	0.0147	0.0164	0.0121	0.0280	0.2121
CDKN1A_E101_F	0.0087	0.0017	0.0046	0.0072	0.0073	0.0081	0.1030	0.0000	0.0040	0.0000	0.7984	0.0039
CDKN1A_P242_F	0.0027	0.0000	0.0093	0.0078	0.0085	0.1444	0.0000	0.0071	0.0024	0.0136	0.0095	0.0000
CDKN1B_P1161_F	0.0000	0.0000	0.0000	0.0000	0.0000	0.0000	0.0158	0.0000	0.3123	0.0000	0.0000	0.0000
CDKN2A_E121_R	0.0067	0.0048	0.0064	0.0933	0.0080	0.0095	0.0087	0.0043	0.0049	0.0064	0.0163	0.0033
CDKN2B_E220_F	0.0000	0.0166	0.0000	0.0406	0.0056	0.4118	0.0000	0.0000	0.0000	0.0053	0.0091	0.0000
CDM_seq_21_S260_R	0.0056	0.0000	0.0041	0.0556	0.0203	0.0971	0.0000	0.0071	0.0010	0.0093	0.0072	0.0000
CEACAM1_E57_R	0.0035	0.0000	0.0652	0.0053	0.0036	0.0042	0.0000	0.0042	0.1554	0.0036	0.0106	0.0000
CEACAM1_P44_R	0.0069	0.2510	0.0080	0.0096	0.0155	0.0063	0.0000	0.0144	0.0034	0.2300	0.0104	0.0060
CEBPA_P1163_R	0.0210	0.0353	0.0135	0.3105	0.0217	0.0688	0.0661	0.2027	0.0097	0.0164	0.0160	0.1849
CEBPA_P706_F	0.0052	0.0000	0.3352	0.4207	0.0054	0.0047	0.0000	0.0076	0.0039	0.0000	0.0127	0.0025
CFTR_P115_F	0.0019	0.0052	0.0047	0.0050	0.0067	0.1139	0.0000	0.0085	0.0013	0.0064	0.0099	0.1242
CHD2_P451_F	0.0000	0.0000	0.0000	0.2268	0.0000	0.6881	0.0000	0.0000	0.0000	0.0000	0.1021	0.0000
CHFR_P501_F	0.0021	0.0000	0.0035	0.0051	0.0144	0.0040	0.0103	0.0035	0.0006	0.0053	0.0010	0.0000
CHFR_P635_R	0.9794	0.9085	0.9875	0.9638	0.0003	0.9832	0.9922	0.8306	0.9387	0.8010	0.9835	0.1270
CHGA_E52_F	0.0000	0.0006	0.0038	0.0038	0.0085	0.0021	0.0000	0.0001	0.0000	0.0062	0.0035	0.0000
CHI3L2_E10_F	0.0093	0.5724	0.0063	0.6345	0.7358	0.6176	0.5778	0.0076	0.0098	0.6045	0.0115	0.0038
CLK1_P538_F	0.0003	0.0000	0.0000	0.0026	0.0031	0.0010	0.0000	0.0000	0.0000	0.0000	0.0000	0.0000
COL18A1_P365_R	0.0105	0.0000	0.0083	0.0132	0.0156	0.0905	0.0119	0.0086	0.0357	0.0154	0.0154	0.2788
COL1A1_P5_F	0.0000	0.0000	0.0000	0.0000	0.0027	0.0000	0.0000	0.0000	0.0000	0.0000	0.0000	0.0000
COL1A2_E299_F	0.0045	0.4672	0.4166	0.4343	0.9245	0.3533	0.0000	0.0048	0.0048	0.0078	0.3986	0.0071
COL1A2_P407_R	0.0014	0.0000	0.0000	0.0000	0.0000	0.0000	0.0000	0.0000	0.0000	0.0000	0.0002	0.0000
COL1A2_P48_R	0.0270	0.0318	0.0293	0.0342	0.0200	0.0249	0.0748	0.0201	0.0321	0.0229	0.0336	0.2231
COL4A3_E205_R	0.0000	0.0000	0.0000	0.0012	0.7867	0.0000	0.0076	0.0000	0.0000	0.0028	0.0043	0.0856
COL4A3_P545_F	0.0332	0.8732	0.0418	0.0216	0.0699	0.0148	0.0938	0.0490	0.0372	0.8689	0.7798	0.7727
COL6A1_P283_F	0.0350	0.0328	0.0172	0.0131	0.5811	0.2780	0.0005	0.0078	0.0078	0.0498	0.0119	0.0157
COL6A1_P425_F	0.0137	0.0063	0.0204	0.0088	0.0160	0.0079	0.1094	0.0000	0.0099	0.0098	0.0252	0.0170
COPG2_P298_F	0.6455	0.1431	0.2734	0.0406	0.1003	0.0408	0.1441	0.0748	0.1556	0.0725	0.0639	0.5456
CPA4_P1265_R	0.0427	0.7852	0.0339	0.0615	0.0355	0.1637	0.7286	0.0335	0.0256	0.0766	0.0310	0.0249
CREB1_P819_F	0.0052	0.9718	0.0045	0.0078	0.0093	0.0061	0.0172	0.0065	0.0000	0.0073	0.0052	0.0000
CRIP1_P874_R	0.0184	0.0058	0.0072	0.0128	0.0185	0.0087	0.0173	0.0093	0.0061	0.0101	0.0120	0.3280
CRK_P721_F	0.0051	0.0057	0.0042	0.0126	0.0602	0.0178	0.0067	0.0105	0.0595	0.0435	0.0403	0.0034

CSF1_P217_F	0.0172	0.1959	0.8797	0.5760	0.9823	0.4311	0.1015	0.0161	0.0570	0.1756	0.0193	0.1927
CSF1_P339_F	0.0000	0.0000	0.0000	0.0000	0.0000	0.0000	0.0026	0.0000	0.0000	0.0000	0.0000	0.0000
CSF1R_E26_F	0.0055	0.0000	0.0000	0.0042	0.0076	0.0048	0.1733	0.0014	0.0044	0.0070	0.0000	0.0000
CSF3R_P472_F	0.0349	0.1857	0.0324	0.0313	0.0343	0.0268	0.1341	0.0399	0.0343	0.0450	0.0236	0.1755
CSPG2_E38_F	0.0005	0.0034	0.0040	0.0064	0.0060	0.0022	0.0000	0.0000	0.0000	0.0073	0.0061	0.0014
CSPG2_P82_R	0.0022	0.0037	0.0044	0.0056	0.0062	0.0027	0.0000	0.0000	0.0015	0.0068	0.0057	0.0000
CSTB_E410_F	0.9887	0.9213	0.0000	0.9287	0.5924	0.9320	0.0000	0.0080	0.0000	0.9757	0.9436	0.0067
CTAG1B_P4_R	0.0000	0.0043	0.7317	0.0000	0.0026	0.4339	0.0051	0.0022	0.0000	0.0000	0.0000	0.0000
CTAG1B_P77_F	0.8818	0.3213	0.9756	0.3648	0.1917	0.1391	0.0221	0.0181	0.0194	0.9916	0.0259	0.9903
CTAG2_P1426_F	0.5837	0.6867	0.0470	0.0119	0.0268	0.0108	0.7703	0.0262	0.0142	0.0321	0.0191	0.5403
CTGF_E156_F	0.0004	0.0000	0.0027	0.0009	0.0041	0.0000	0.0048	0.0032	0.0000	0.0000	0.0031	0.0000
CTLA4_P1128_F	0.9892	0.7165	0.2150	0.2247	0.4382	0.3128	0.3161	0.8629	0.8093	0.6424	0.2623	0.2846
CTNNA1_P185_R	0.0000	0.0063	0.0025	0.0003	0.0032	0.0018	0.0000	0.0000	0.0000	0.0018	0.0034	0.0000
CTNNA1_P382_R	0.0008	0.0947	0.0040	0.0034	0.0142	0.0021	0.1074	0.0039	0.0038	0.0058	0.0054	0.0035
CTNNB1_P757_F	0.0080	0.0074	0.0060	0.0087	0.0409	0.0070	0.0759	0.0070	0.0033	0.0119	0.0090	0.5781
CTSD_P726_F	0.0000	0.0000	0.0000	0.0000	0.0000	0.0000	0.0000	0.0000	0.0000	0.0000	0.0000	0.0000
CTSH_P238_F	0.0000	0.0000	0.0000	0.0000	0.0000	0.0000	0.0000	0.0000	0.0000	0.0000	0.0000	0.0000
CTSL_P264_R	0.0051	0.0000	0.0063	0.0051	0.0122	0.1447	0.0000	0.0041	0.0012	0.0096	0.0070	0.0013
CTSL_P81_F	0.0036	0.0000	0.0028	0.2618	0.0009	0.0010	0.0074	0.0146	0.0037	0.0091	0.0128	0.0007
CTTN_E29_R	0.0054	0.0000	0.0040	0.0050	0.0000	0.0015	0.0092	0.0058	0.0000	0.0000	0.0090	0.0036
CYP1A1_P382_F	0.0142	0.6161	0.0215	0.0288	0.0468	0.0369	0.4654	0.0301	0.0542	0.0665	0.0265	0.5779
CYP2E1_P416_F	0.0200	0.0100	0.0067	0.0131	0.0064	0.0076	0.0032	0.1147	0.0473	0.0082	0.0082	0.0792
DAB2_P35_F	0.0000	0.0000	0.0000	0.0563	0.0000	0.0000	0.0000	0.0000	0.0000	0.8098	0.0000	0.0000
DAB2_P468_F	0.0612	0.2655	0.0240	0.0301	0.0232	0.0264	0.0180	0.0100	0.0263	0.0184	0.0222	0.2393
DAB2IP_P9_F	0.0000	0.0000	0.0000	0.0000	0.0000	0.2701	0.0000	0.0000	0.0000	0.0000	0.0000	0.0000
DAPK1_P10_F	0.0010	0.0000	0.0000	0.0016	0.0175	0.4446	0.0000	0.0049	0.0000	0.0058	0.0106	0.0000
DAPK1_P345_R	0.0000	0.0003	0.0000	0.0000	0.0000	0.0000	0.0000	0.0000	0.0000	0.0000	0.0042	0.0022
DBC1_P351_R	0.0000	0.0000	0.0000	0.0000	0.0104	0.0001	0.0000	0.0000	0.0000	0.0023	0.0000	0.0000
DCC_P177_F	0.0059	0.1373	0.0076	0.2403	0.0065	0.0785	0.0070	0.0112	0.0064	0.0110	0.4634	0.0049
DCC_P471_R	0.0085	0.2407	0.0116	0.0155	0.2341	0.0256	0.0086	0.0295	0.0158	0.0159	0.0312	0.0133
DCN_P1320_R	0.0000	0.0000	0.0000	0.0000	0.0000	0.0000	0.0000	0.0000	0.0000	0.9374	0.0068	0.9859
DDB2_P407_F	0.0000	0.0000	0.0000	0.4981	0.0018	0.0000	0.0000	0.0000	0.0000	0.0000	0.3508	0.0000
DDB2_P613_R	0.0069	0.0053	0.0034	0.0056	0.0755	0.0039	0.0378	0.0057	0.0051	0.0066	0.0082	0.0246

DDR1_E23_R	0.0000	0.0094	0.0000	0.0058	0.0058	0.1561	0.0000	0.0058	0.0029	0.0095	0.0090	0.0037
DDR2_E331_F	0.0000	0.9897	0.9864	0.9791	0.0000	0.9739	0.0000	0.9828	0.9865	0.0000	0.9864	0.0552
DES_E228_R	0.0000	0.0000	0.0044	0.0093	0.0000	0.0040	0.0000	0.0089	0.0003	0.0000	0.0000	0.0000
DHCR24_P406_R	0.2701	0.0158	0.2217	0.2216	0.4215	0.1208	0.0160	0.0107	0.0167	0.0127	0.0150	0.0096
DIO3_E230_R	0.0033	0.0000	0.0034	0.0056	0.0075	0.0037	0.0000	0.0057	0.0020	0.0050	0.0117	0.0000
DIO3_P674_F	0.8231	0.1682	0.0081	0.6804	0.0117	0.5733	0.0120	0.0147	0.0095	0.0103	0.0215	0.0085
DIRAS3_E55_R	0.0000	0.0000	0.0329	0.0222	0.0134	0.0235	0.0000	0.1108	0.8700	0.0000	0.0141	0.0000
DKC1_E101_F	0.0000	0.0000	0.0000	0.0000	0.0000	0.0000	0.0000	0.0000	0.0000	0.0000	0.0000	0.0000
DKFZP564O0823_E45_F	0.0014	0.0015	0.0000	0.0037	0.0660	0.0017	0.0000	0.0034	0.0000	0.0052	0.0000	0.0034
DLC1_P88_R	0.0000	0.0000	0.0000	0.7241	0.0019	0.6244	0.0000	0.0051	0.0000	0.0000	0.0000	0.0000
DLK1_E227_R	0.9948	0.9965	0.9798	0.9921	0.9875	0.9916	0.9975	0.9788	0.9951	0.9706	0.9949	0.9973
DLL1_P386_F	0.9820	0.0000	0.8229	0.8584	0.8542	0.9669	0.9907	0.9683	0.9187	0.9815	0.0000	0.0000
DLL1_P832_F	0.9861	0.0091	0.0050	0.1616	0.0098	0.0745	0.0000	0.0060	0.0045	0.0022	0.0115	0.0036
DMP1_P134_F	0.0767	0.0145	0.0233	0.0191	0.0246	0.0722	0.0175	0.0117	0.0149	0.0150	0.7014	0.0079
DNAJC15_E26_R	0.0042	0.0674	0.0062	0.0079	0.0073	0.0064	0.1413	0.0055	0.0019	0.0063	0.0157	0.0033
DNASE1L1_P108_F	0.7510	0.1202	0.5574	0.9372	0.9428	0.9857	0.0338	0.0296	0.9490	0.5664	0.0301	0.0198
DNMT1_P100_R	0.9852	0.4950	0.9531	0.9835	0.9824	0.9829	0.4471	0.9857	0.9890	0.7531	0.9885	0.4549
DSC2_E90_F	0.9575	0.9631	0.9219	0.9494	0.0000	0.9657	0.0000	0.0000	0.9529	0.0000	0.0000	0.0000
DSP_P440_R	0.0283	0.7541	0.9249	0.9234	0.0235	0.9789	0.4091	0.8905	0.6371	0.9873	0.9171	0.9881
DST_E31_F	0.8949	0.0296	0.9826	0.6062	0.0620	0.7421	0.0091	0.0269	0.4846	0.0207	0.0161	0.7993
DST_P262_R	0.0745	0.0455	0.0620	0.0736	0.0862	0.0609	0.5266	0.0359	0.1220	0.0861	0.0602	0.5546
DUSP4_E61_F	0.0000	0.0000	0.0071	0.0083	0.0060	0.0047	0.0000	0.0000	0.0049	0.0000	0.0116	0.0804
DUSP4_P925_R	0.0329	0.0206	0.0373	0.2367	0.0241	0.1178	0.3971	0.0133	0.3983	0.6158	0.0270	0.4690
E2F3_P840_R	0.0166	0.9824	0.8734	0.6441	0.0000	0.1857	0.8182	0.0061	0.0000	0.0153	0.6761	0.0000
E2F5_P516_R	0.9696	0.9520	0.9365	0.9373	0.9615	0.9551	0.1323	0.9239	0.9665	0.9634	0.2905	0.9716
EDN1_E50_R	0.4686	0.9861	0.5227	0.3612	0.4237	0.2636	0.9785	0.9827	0.5762	0.6827	0.7176	0.4737
EDN1_P39_R	0.0126	0.6352	0.2426	0.2276	0.0910	0.0121	0.7417	0.3035	0.0006	0.6234	0.0979	0.9899
EDNRB_P148_R	0.0000	0.0000	0.9822	0.9170	0.0000	0.9762	0.0010	0.0000	0.9889	0.2283	0.8071	0.0000
EFNA1_P591_R	0.0117	0.0000	0.0038	0.0038	0.0000	0.3878	0.2092	0.0000	0.0000	0.0000	0.0076	0.8012
EFNA1_P7_F	0.0000	0.0000	0.0000	0.5287	0.7944	0.8367	0.0000	0.0000	0.0000	0.0000	0.0028	0.0000
EFNB1_E69_F	0.0055	0.0000	0.0032	0.3060	0.0085	0.5464	0.0109	0.1857	0.0006	0.0000	0.0110	0.0062
EFNB3_P442_R	0.0050	0.0040	0.0011	0.0018	0.0038	0.1557	0.0080	0.0048	0.0000	0.0054	0.0083	0.8750
EGF_E339_F	0.0080	0.0000	0.6565	0.2509	0.0001	0.1959	0.0000	0.0000	0.0000	0.0010	0.0000	0.5746

EGFR_E295_R	0.0037	0.1406	0.0034	0.0679	0.0053	0.0074	0.0819	0.0062	0.0017	0.0073	0.0061	0.0027
EGFR_P260_R	0.9835	0.0000	0.9835	0.3181	0.5308	0.9735	0.0000	0.0000	0.9839	0.0044	0.0063	0.0020
EGR4_P479_F	0.9855	0.7907	0.9827	0.8926	0.9094	0.8730	0.9909	0.5143	0.9651	0.5658	0.0271	0.3569
EIF2AK2_E103_R	0.0000	0.0000	0.9775	0.9744	0.0000	0.9802	0.0000	0.0000	0.7896	0.6690	0.0000	0.0000
EIF2AK2_P313_F	0.9755	0.9940	0.8024	0.9866	0.9108	0.9870	0.4736	0.6326	0.9900	0.2631	0.9895	0.1462
ELK1_E156_F	0.0000	0.0000	0.9860	0.9367	0.0018	0.9694	0.0000	0.9857	0.9788	0.0000	0.9814	0.9900
EMR3_P39_R	0.0111	0.0112	0.9798	0.7044	0.7671	0.5818	0.4742	0.9080	0.9774	0.0093	0.0340	0.9918
ENC1_P484_R	0.9859	0.0085	0.9884	0.9841	0.9927	0.9852	0.2083	0.9878	0.0027	0.9830	0.9133	0.0051
EPHA1_E46_R	0.0225	0.1439	0.0160	0.4951	0.0299	0.3086	0.2518	0.2468	0.0222	0.7931	0.6671	0.5977
EPHA1_P119_R	0.5335	0.0000	0.0009	0.0844	0.0093	0.1120	0.0110	0.7345	0.0000	0.0056	0.0091	0.0000
EPHA2_P203_F	0.7789	0.0047	0.0037	0.1144	0.0061	0.2394	0.0000	0.0103	0.0000	0.0055	0.0094	0.0019
EPHA3_E156_R	0.0000	0.0000	0.0000	0.0016	0.0000	0.1340	0.0000	0.0000	0.0000	0.0020	0.0664	0.0000
EPHA7_E6_F	0.0159	0.0000	0.5510	0.4816	0.0059	0.5658	0.6899	0.5023	0.5169	0.0057	0.4261	0.0029
EPHA7_P205_R	0.9612	0.9783	0.9704	0.9847	0.9881	0.9855	0.0795	0.2417	0.9026	0.9921	0.9781	0.9916
EPHA8_P256_F	0.0212	0.0255	0.0109	0.1162	0.0233	0.0185	0.1446	0.0288	0.0188	0.0521	0.0278	0.1922
EPHB1_P503_F	0.6034	0.1109	0.0064	0.3163	0.6065	0.3142	0.0129	0.2201	0.0041	0.5382	0.2841	0.0053
EPHB2_E297_F	0.0610	0.1263	0.0628	0.0171	0.0263	0.2984	0.8299	0.6938	0.1073	0.6911	0.5634	0.0677
EPHB2_P165_R	0.0054	0.0608	0.0075	0.5044	0.0000	0.4263	0.0152	0.0000	0.0000	0.0102	0.0110	0.0073
EPHB3_E0_F	0.9221	0.0000	0.7252	0.2601	0.0000	0.5177	0.0000	0.0002	0.0000	0.0010	0.0012	0.0000
EPHB3_P569_R	0.0142	0.3269	0.0088	0.0219	0.1915	0.0227	0.1873	0.1009	0.3502	0.0257	0.0264	0.2961
EPHB4_P313_R	0.0397	0.0204	0.0864	0.2083	0.0391	0.2965	0.1175	0.6422	0.0350	0.9822	0.0216	0.6679
EPHB6_E342_F	0.0864	0.6494	0.0677	0.1221	0.0286	0.1508	0.0000	0.9583	0.0764	0.5450	0.5740	0.0493
EPHB6_P827_R	0.0017	0.0000	0.0007	0.3246	0.0044	0.2897	0.0084	0.0000	0.0000	0.0054	0.0029	0.0002
EPHX1_P1358_R	0.0000	0.6263	0.9836	0.9604	0.0000	0.9757	0.0000	0.0000	0.8875	0.8884	0.9309	0.0000
EPM2A_P113_F	0.0921	0.1722	0.1452	0.5435	0.7327	0.2747	0.8524	0.7835	0.0986	0.2743	0.0704	0.0735
EPM2A_P64_R	0.5569	0.7461	0.0194	0.0192	0.0224	0.0149	0.5166	0.6289	0.6582	0.0188	0.0204	0.4254
EPO_E244_R	0.0000	0.0000	0.0000	0.0000	0.0014	0.1433	0.0038	0.0021	0.0000	0.0003	0.0023	0.0002
EPO_P162_R	0.6103	0.0000	0.0000	0.5255	0.0000	0.6081	0.0000	0.0000	0.4066	0.0000	0.5650	0.0000
EPS8_E231_F	0.9800	0.9834	0.9695	0.5407	0.9774	0.9638	0.0318	0.0000	0.1810	0.0634	0.0665	0.0920
EPS8_P437_F	0.0000	0.0000	0.0000	0.3359	0.0053	0.5200	0.7628	0.0000	0.0000	0.0000	0.7552	0.0000
ERBB2_P59_R	0.6283	0.1567	0.4789	0.7146	0.9886	0.9599	0.9965	0.8694	0.0000	0.9135	0.9822	0.0024
ERBB3_E331_F	0.0168	0.9896	0.0131	0.5237	0.0217	0.6064	0.0257	0.0168	0.5787	0.0256	0.6671	0.0174
ERBB3_P870_R	0.0542	0.4561	0.1759	0.2628	0.0129	0.0145	0.2158	0.5795	0.0075	0.1452	0.0194	0.3246

ERBB4_P255_F	0.0124	0.4692	0.0269	0.0176	0.0176	0.1214	0.2158	0.4134	0.0098	0.0145	0.0192	0.0079
ERBB4_P541_F	0.2737	0.2094	0.9756	0.8899	0.0303	0.6562	0.0000	0.0000	0.1196	0.8147	0.0213	0.9906
ERCC1_P354_F	0.8548	0.4958	0.4422	0.7641	0.8198	0.7480	0.0000	0.7670	0.5644	0.8275	0.4802	0.9927
ERCC3_P1210_R	0.9886	0.3794	0.5808	0.6255	0.5941	0.9566	0.3075	0.0244	0.0391	0.0197	0.8458	0.2564
ERG_E28_F	0.5359	0.0226	0.0166	0.4935	0.3536	0.5852	0.7254	0.0261	0.9085	0.0227	0.3743	0.0105
ERN1_P809_R	0.9331	0.0075	0.9877	0.9677	0.9869	0.9760	0.9945	0.9816	0.0000	0.0000	0.9892	0.1771
ESR1_E298_R	0.0211	0.7776	0.6912	0.9147	0.9865	0.9121	0.8297	0.9879	0.5866	0.7048	0.1019	0.7099
ESR1_P151_R	0.5178	0.0000	0.8751	0.7994	0.6342	0.9651	0.1328	0.0071	0.0030	0.0266	0.0241	0.0062
ESR2_E66_F	0.4405	0.0890	0.8303	0.1152	0.0781	0.5330	0.1930	0.9570	0.7937	0.4063	0.0342	0.3589
ESR2_P162_F	0.0356	0.0305	0.0294	0.1763	0.0160	0.4291	0.0296	0.0374	0.0559	0.0333	0.0341	0.0170
ETS1_E253_R	0.0264	0.0244	0.0313	0.0332	0.1890	0.3936	0.1570	0.0398	0.1501	0.1432	0.8406	0.0334
ETS1_P559_R	0.9881	0.0000	0.0000	0.9800	0.9873	0.9806	0.0000	0.0000	0.9860	0.0000	0.9844	0.9834
ETS2_P684_F	0.0148	0.9924	0.5229	0.6634	0.6933	0.7906	0.9942	0.9886	0.7400	0.3948	0.0201	0.3700
ETS2_P835_F	0.2092	0.0000	0.0044	0.0140	0.0097	0.0058	0.0000	0.0170	0.0000	0.1714	0.0117	0.4913
ETV1_P235_F	0.9797	0.8719	0.9848	0.9668	0.0197	0.9801	0.3814	0.3027	0.0000	0.8745	0.9872	0.6504
ETV1_P515_F	0.0072	0.0000	0.0056	0.9706	0.0056	0.0021	0.0000	0.0003	0.1117	0.0000	0.0000	0.0092
ETV6_E430_F	0.1388	0.6174	0.5879	0.2934	0.4937	0.3884	0.0136	0.0030	0.4218	0.0078	0.0116	0.0041
EVI1_E47_R	0.3897	0.3271	0.2736	0.9722	0.6583	0.9734	0.2561	0.2891	0.4814	0.2958	0.9697	0.0194
EVI1_P30_R	0.0000	0.0000	0.0041	0.0038	0.0050	0.0536	0.0076	0.6098	0.0000	0.0000	0.0123	0.0028
EVI2A_P94_R	0.7505	0.0000	0.0000	0.3700	0.0057	0.2201	0.0000	0.0000	0.0000	0.0000	0.0082	0.0000
EXT1_E197_F	0.0261	0.0188	0.0072	0.0167	0.0221	0.2070	0.3220	0.5376	0.0144	0.5745	0.4435	0.2636
EYA4_E277_F	0.0056	0.0745	0.0100	0.0108	0.0095	0.0083	0.0000	0.9885	0.0026	0.0123	0.0618	0.0041
EYA4_P508_F	0.9877	0.3233	0.0000	0.6369	0.4534	0.6685	0.9915	0.0053	0.9112	0.0000	0.0013	0.0000
EYA4_P794_F	0.0000	0.0000	0.7176	0.8638	0.0011	0.9777	0.0738	0.0000	0.9892	0.0000	0.9805	0.0000
F2R_P839_F	0.7832	0.0045	0.0000	0.9805	0.2967	0.9819	0.0000	0.0000	0.3546	0.9888	0.9855	0.8546
F2R_P88_F	0.4758	0.3454	0.5970	0.6020	0.0051	0.8974	0.7210	0.0080	0.8057	0.0000	0.7881	0.0004
FABP3_E113_F	0.4657	0.0064	0.0098	0.3944	0.3352	0.6492	0.9927	0.0146	0.4306	0.9852	0.5188	0.0062
FABP3_P598_F	0.1348	0.3780	0.3095	0.1880	0.5641	0.5217	0.9961	0.8480	0.3978	0.9689	0.7777	0.6182
FANCE_P356_R	0.0000	0.0000	0.0022	0.8389	0.0000	0.8456	0.0000	0.4430	0.0000	0.9875	0.0135	0.0000
FANCF_P13_F	0.0728	0.7590	0.2466	0.5717	0.5790	0.6084	0.6241	0.5241	0.9654	0.6185	0.5824	0.6797
FANCG_E207_R	0.6629	0.0931	0.0954	0.0352	0.0552	0.2076	0.9677	0.1299	0.0528	0.1452	0.1303	0.0527
FAS_P322_R	0.9677	0.1562	0.7099	0.7986	0.9812	0.6581	0.9880	0.9851	0.8671	0.4103	0.0108	0.3794
FASTK_P257_F	0.0856	0.0873	0.0915	0.7999	0.7363	0.6913	0.0762	0.0841	0.0767	0.0877	0.0942	0.0211

FAT_P973_R	0.0215	0.0093	0.0175	0.2231	0.0329	0.4482	0.0123	0.0261	0.0016	0.0447	0.0116	0.0126
FER_P581_F	0.6601	0.0387	0.5947	0.8828	0.5559	0.9184	0.2328	0.9876	0.9790	0.9887	0.5894	0.3246
FES_P223_R	0.0000	0.0000	0.0000	0.9539	0.0000	0.9697	0.0000	0.9599	0.0000	0.9806	0.9757	0.0000
FGF1_E5_F	0.3706	0.7763	0.9684	0.9358	0.8548	0.9699	0.3192	0.9907	0.0750	0.4972	0.4491	0.9162
FGF12_E61_R	0.0075	0.5492	0.0110	0.0944	0.0215	0.1349	0.1947	0.3951	0.5857	0.0159	0.6508	0.0142
FGF12_P210_R	0.9798	0.9904	0.9824	0.9816	0.9896	0.9843	0.0000	0.7822	0.9911	0.9884	0.9781	0.9901
FGF2_P153_F	0.0301	0.3326	0.0270	0.2735	0.0181	0.2323	0.0171	0.0240	0.0328	0.7523	0.3474	0.0146
FGF2_P229_F	0.1287	0.4618	0.0472	0.5696	0.7783	0.6237	0.4513	0.0213	0.0301	0.1165	0.5087	0.6346
FGF3_E198_R	0.3698	0.4100	0.6962	0.7299	0.9779	0.6056	0.9919	0.5126	0.9772	0.7820	0.6654	0.6339
FGF3_P171_R	0.7812	0.9299	0.9712	0.8238	0.6728	0.9138	0.0043	0.0007	0.4233	0.5126	0.8708	0.0032
FGF5_E16_F	0.9834	0.0082	0.9889	0.9795	0.9826	0.9713	0.0000	0.9916	0.0043	0.9848	0.0076	0.9904
FGF5_P238_R	0.2373	0.0000	0.4357	0.3998	0.2930	0.4421	0.0056	0.6970	0.0000	0.4739	0.6167	0.0000
FGF6_E294_F	0.0647	0.1661	0.0464	0.6115	0.8570	0.8642	0.8011	0.5620	0.0551	0.0566	0.6504	0.0380
FGF7_P44_F	0.0028	0.0000	0.0000	0.6255	0.5999	0.5547	0.0000	0.0000	0.0000	0.0000	0.0057	0.0000
FGF8_E183_F	0.0251	0.0000	0.1924	0.2177	0.0165	0.0271	0.0000	0.0000	0.0359	0.0291	0.1803	0.0000
FGF8_P473_F	0.0000	0.0000	0.4557	0.4708	0.9753	0.1555	0.0000	0.0000	0.7536	0.0000	0.9737	0.0000
FGFR1_E317_F	0.5899	0.0000	0.0162	0.2566	0.0842	0.0114	0.9923	0.0097	0.0085	0.0000	0.0089	0.0120
FGFR2_P266_R	0.0092	0.0192	0.2727	0.0102	0.0132	0.0085	0.1298	0.2785	0.0055	0.0095	0.0116	0.0057
FGFR3_E297_R	0.0000	0.0142	0.0137	0.9789	0.8269	0.9677	0.0553	0.0000	0.9782	0.8875	0.0002	0.0000
FGFR3_P1152_R	0.9512	0.8451	0.8186	0.9006	0.8458	0.8048	0.0000	0.8332	0.9926	0.9566	0.9484	0.0312
FHIT_E19_R	0.9892	0.0000	0.9494	0.9307	0.9841	0.9759	0.0000	0.9893	0.9790	0.9256	0.9820	0.0000
FHIT_P93_R	0.0078	0.0055	0.0044	0.0934	0.6170	0.1995	0.0089	0.0037	0.0015	0.0048	0.0285	0.0029
FHL1_E229_R	0.0088	0.0000	0.9823	0.1326	0.0000	0.2327	0.0000	0.0000	0.0000	0.5301	0.0034	0.0000
FLI1_P620_R	0.0813	0.1535	0.7926	0.8241	0.9758	0.8442	0.0944	0.0000	0.9670	0.0148	0.0059	0.0000
FLT1_E444_F	0.1831	0.0451	0.4088	0.5557	0.4114	0.7391	0.9938	0.9458	0.3817	0.0330	0.8585	0.1015
FLT1_P302_F	0.0260	0.0265	0.1202	0.0233	0.0368	0.1332	0.0195	0.7622	0.0218	0.0273	0.0211	0.0170
FLT1_P615_R	0.0073	0.0054	0.6322	0.4210	0.0105	0.0043	0.1446	0.0106	0.0056	0.0095	0.2446	0.1071
FLT3_E326_R	0.0061	0.0081	0.1990	0.1202	0.0179	0.1726	0.0128	0.0121	0.0031	0.0505	0.0112	0.1232
FLT4_P180_R	0.9864	0.9715	0.9887	0.9263	0.9678	0.9802	0.0188	0.8573	0.9793	0.9832	0.8686	0.9445
FMR1_P62_R	0.3164	0.9128	0.3604	0.2665	0.4338	0.2319	0.9404	0.4414	0.3087	0.4183	0.3060	0.9013
FN1_E469_F	0.0000	0.0033	0.6066	0.1566	0.0047	0.2922	0.0087	0.0070	0.0000	0.0057	0.0066	0.0023
FN1_P229_R	0.0121	0.0203	0.7168	0.1919	0.0209	0.0473	0.0209	0.6265	0.4887	0.0139	0.5772	0.0385
FOSL2_E384_R	0.9865	0.4207	0.9887	0.8994	0.8482	0.9637	0.0675	0.0048	0.7979	0.9924	0.8925	0.9940

FRK_P36_F	0.0135	0.9880	0.0098	0.2236	0.0127	0.1229	0.0111	0.4647	0.0064	0.0000	0.1086	0.9119
FRZB_E186_R	0.9731	0.0000	0.0000	0.9291	0.5810	0.7210	0.5933	0.0000	0.0000	0.9697	0.0000	0.0000
FRZB_P406_F	0.0000	0.0000	0.5686	0.0019	0.2626	0.1119	0.0088	0.0000	0.0000	0.0059	0.0053	0.0000
FVT1_P225_F	0.0048	0.0000	0.0037	0.0544	0.4196	0.0068	0.0000	0.0000	0.0044	0.0057	0.0133	0.9902
FYN_P352_R	0.9872	0.9935	0.9912	0.9793	0.9921	0.5691	0.9976	0.9922	0.4527	0.9575	0.9024	0.9923
FZD7_E296_F	0.9780	0.9898	0.9850	0.9764	0.0000	0.9713	0.0000	0.0000	0.9868	0.0089	0.9643	0.0000
FZD9_E458_F	0.0000	0.0000	0.8800	0.9678	0.9803	0.9728	0.0000	0.0000	0.9402	0.0000	0.0000	0.1982
FZD9_P15_R	0.0004	0.0066	0.0000	0.2802	0.0065	0.0000	0.0000	0.0025	0.0000	0.0000	0.0013	0.0000
FZD9_P175_F	0.4815	0.9902	0.3675	0.3912	0.1369	0.5266	0.1428	0.5329	0.0103	0.9842	0.2586	0.7920
G6PD_E190_F	0.0000	0.0000	0.0000	0.5927	0.0000	0.5792	0.0000	0.0000	0.0000	0.9871	0.0000	0.0000
G6PD_P196_F	0.6129	0.0478	0.0120	0.4996	0.4207	0.3008	0.0158	0.0146	0.0069	0.8061	0.0191	0.4698
GABRB3_E42_F	0.0000	0.0000	0.0028	0.0828	0.0083	0.0044	0.0015	0.0000	0.0000	0.0048	0.0019	0.1131
GADD45A_P737_R	0.0207	0.5588	0.4098	0.0220	0.0487	0.0277	0.0237	0.6943	0.0197	0.0293	0.0273	0.0225
GALR1_E52_F	0.0162	0.2032	0.0064	0.0191	0.0093	0.0121	0.0650	0.0092	0.0084	0.0055	0.0217	0.3090
GALR1_P80_F	0.0076	0.0066	0.0111	0.0484	0.0082	0.0066	0.0000	0.0082	0.0022	0.0089	0.0097	0.9882
GAS1_E22_F	0.0079	0.0379	0.0071	0.0086	0.0157	0.0614	0.0117	0.0065	0.3205	0.0103	0.0162	0.0001
GAS1_P754_R	0.0000	0.0000	0.0000	0.0028	0.0000	0.0000	0.0044	0.0022	0.0000	0.0048	0.0061	0.0022
GAS7_E148_F	0.0149	0.0093	0.0114	0.2289	0.0103	0.0115	0.0140	0.2048	0.0131	0.0181	0.0198	0.4138
GAS7_P622_R	0.0000	0.0000	0.0000	0.0000	0.0000	0.0000	0.0000	0.0000	0.0000	0.0000	0.0000	0.0000
GATA6_P21_R	0.0040	0.0053	0.0046	0.0783	0.0084	0.0055	0.0000	0.0052	0.0022	0.0045	0.0080	0.0000
GATA6_P726_F	0.0189	0.5473	0.0107	0.0118	0.0137	0.0079	0.0723	0.0095	0.0155	0.3733	0.0105	0.0113
GFI1_E136_F	0.0726	0.0006	0.0057	0.0076	0.1150	0.0020	0.0067	0.0059	0.0002	0.1587	0.0081	0.1953
GFI1_P45_R	0.0042	0.0000	0.0006	0.0051	0.0071	0.0489	0.0072	0.0043	0.0024	0.0061	0.0109	0.0058
GJB2_E43_F	0.0063	0.0037	0.0048	0.0136	0.0057	0.0159	0.0000	0.0099	0.0109	0.0093	0.0146	0.0058
GJB2_P791_R	0.0146	0.1925	0.0101	0.0115	0.0137	0.1006	0.0138	0.0091	0.0112	0.0104	0.0106	0.1504
GJB2_P931_R	0.2717	0.3068	0.0645	0.0298	0.0407	0.0283	0.1685	0.1900	0.2238	0.0443	0.0568	0.0246
GLA_P112_F	0.0224	0.0074	0.0113	0.3706	0.9384	0.8185	0.2538	0.0188	0.0000	0.0251	0.0447	0.0499
GLI2_E90_F	0.9857	0.9882	0.9850	0.9820	0.9834	0.9815	0.9239	0.9894	0.9888	0.9786	0.9858	0.0000
GLI3_E148_R	0.9484	0.7261	0.9770	0.9038	0.6459	0.9824	0.0962	0.9468	0.9820	0.6950	0.9914	0.9769
GML_P281_R	0.9837	0.9907	0.9874	0.9537	0.0000	0.9443	0.9960	0.0000	0.0000	0.0000	0.0013	0.0000
GNAS_E58_F	0.1057	0.0000	0.9868	0.8129	0.8574	0.9465	0.0000	0.8017	0.8975	0.0000	0.7127	0.9912
GP1BB_E23_F	0.0073	0.0094	0.0060	0.0083	0.9889	0.0650	0.0079	0.0070	0.0054	0.0055	0.0125	0.1397
GPC3_P235_R	0.0008	0.0000	0.0025	0.4484	0.0025	0.6221	0.0079	0.0016	0.0000	0.6964	0.0061	0.0000

GPR116_E328_R	0.9874	0.9077	0.9851	0.9824	0.9853	0.9833	0.9937	0.9817	0.9900	0.9927	0.9907	0.0000
GPX1_E46_R	0.0086	0.0060	0.0129	0.0088	0.0095	0.0056	0.0000	0.0096	0.0056	0.0086	0.0126	0.0000
GPX1_P194_F	0.0242	0.8028	0.0270	0.0216	0.5742	0.0152	0.6610	0.0267	0.0413	0.0270	0.0291	0.4175
GPX3_E178_F	0.0106	0.0162	0.2877	0.1310	0.0115	0.1074	0.0094	0.0093	0.0085	0.0083	0.0159	0.0068
GRB10_E85_R	0.0174	0.0020	0.4575	0.0084	0.0156	0.0048	0.0225	0.0113	0.0026	0.0062	0.0101	0.0000
GRB10_P260_F	0.0158	0.0039	0.0116	0.0048	0.0076	0.0060	0.0126	0.0104	0.0037	0.0086	0.0208	0.0000
GRB10_P496_R	0.0069	0.0056	0.0132	0.3020	0.0414	0.0028	0.2787	0.0106	0.0128	0.0000	0.0107	0.2333
GRPR_P200_R	0.9811	0.9410	0.1789	0.9198	0.7824	0.8826	0.2085	0.1873	0.1767	0.8060	0.9812	0.1805
GSTM1_P363_F	0.9851	0.0000	0.0110	0.8289	0.0134	0.0089	0.1083	0.0087	0.0055	0.9841	0.0116	0.1669
GSTM2_E153_F	0.0011	0.0036	0.0000	0.0029	0.0065	0.0017	0.0000	0.0052	0.0000	0.0038	0.0055	0.0011
GSTM2_P109_R	0.0030	0.0044	0.0052	0.0020	0.0064	0.0013	0.0085	0.5065	0.0001	0.0044	0.0065	0.0000
GSTM2_P453_R	0.9612	0.0000	0.0008	0.5335	0.9824	0.7529	0.0000	0.9832	0.9826	0.0000	0.9777	0.0000
GSTP1_E322_R	0.0002	0.0000	0.0126	0.0062	0.0084	0.0077	0.0000	0.0349	0.0033	0.0101	0.0080	0.0021
GSTP1_P74_F	0.5104	0.0878	0.0204	0.0197	0.0334	0.1056	0.4572	0.0253	0.0196	0.0198	0.0293	0.1166
GSTP1_seq_38_S153_R	0.2127	0.0098	0.0056	0.0086	0.0000	0.0068	0.0000	0.0000	0.0000	0.0132	0.0076	0.0000
GUCY2D_E419_R	0.0057	0.0012	0.0000	0.0061	0.0071	0.0034	0.0000	0.0039	0.0036	0.0029	0.0066	0.0000
HBEGF_P32_R	0.0007	0.0000	0.0000	0.0018	0.0000	0.0015	0.0000	0.0000	0.0000	0.0000	0.0031	0.0000
HBII-13_E48_F	0.0000	0.0000	0.0000	0.9582	0.9796	0.9670	0.0000	0.9896	0.9720	0.9809	0.0000	0.0000
HBII-52_P563_F	0.0461	0.0367	0.8476	0.8280	0.6827	0.6687	0.0512	0.6958	0.7248	0.0353	0.4069	0.5413
HCK_P46_R	0.0090	0.0096	0.0123	0.0100	0.2154	0.1641	0.0000	0.0109	0.0148	0.0139	0.0000	0.2443
HCK_P858_F	0.0250	0.6909	0.0644	0.0130	0.0297	0.0118	0.0694	0.0339	0.0295	0.6289	0.0331	0.4479
HDAC1_P414_R	0.7793	0.0000	0.0045	0.0029	0.0000	0.3836	0.0120	0.0106	0.0017	0.0070	0.5713	0.0056
HDAC11_P556_F	0.0030	0.0000	0.0000	0.0000	0.0000	0.0005	0.0000	0.0000	0.0000	0.0000	0.0038	0.0000
HDAC9_P137_R	0.4487	0.0030	0.0010	0.1035	0.0026	0.0010	0.0000	0.0020	0.0017	0.0026	0.0036	0.0031
HFE_E273_R	0.0033	0.0019	0.0025	0.0000	0.0015	0.0000	0.0000	0.0024	0.0009	0.0011	0.0054	0.0062
HHIP_P307_R	0.0209	0.1679	0.0220	0.2154	0.0321	0.1233	0.1009	0.0380	0.0243	0.0378	0.0325	0.6276
HIC1_E151_F	0.0000	0.0000	0.0000	0.2606	0.0000	0.0000	0.0000	0.0000	0.9726	0.0000	0.0000	0.0000
HIC1_P565_R	0.0033	0.0000	0.0124	0.0161	0.0049	0.0107	0.0000	0.0000	0.0000	0.0000	0.0000	0.0000
HIC2_P498_F	0.0042	0.0000	0.0000	0.0057	0.0095	0.0038	0.0091	0.0000	0.0000	0.0000	0.0110	0.0000
HIC2_P528_R	0.0031	0.0000	0.0000	0.0037	0.0113	0.0035	0.0237	0.0062	0.0036	0.0112	0.0140	0.0075
HIF1A_P488_F	0.0077	0.0000	0.0000	0.0000	0.0036	0.0000	0.0000	0.0025	0.0000	0.0000	0.0087	0.0105
HLA-DOB_P357_R	0.9830	0.9722	0.9862	0.9688	0.9704	0.9739	0.0000	0.0000	0.0000	0.9706	0.9845	0.9893
HLA-DPA1_P205_R	0.0175	0.0195	0.0203	0.3179	0.0098	0.0221	0.1260	0.1592	0.0069	0.0174	0.0164	0.1387

HLA-DPA1_P28_R	0.0013	0.0053	0.0022	0.0069	0.0075	0.2285	0.0031	0.0055	0.0045	0.0072	0.0062	0.0000
HLA-DPB1_E2_R	0.0404	0.0146	0.0405	0.0323	0.2689	0.0720	0.5559	0.0000	0.0315	0.0230	0.0338	0.6237
HLA-DRA_P132_R	0.0000	0.0000	0.0000	0.0000	0.0000	0.0000	0.0000	0.0000	0.0000	0.0000	0.0000	0.0000
HLA-F_E402_F	0.0000	0.0000	0.0000	0.0000	0.0031	0.0000	0.0009	0.0106	0.0000	0.0007	0.0000	0.0000
HLF_E192_F	0.0316	0.5947	0.0289	0.0158	0.0339	0.0656	0.0252	0.0300	0.0253	0.7703	0.0189	0.0234
HOXA11_E35_F	0.0000	0.0047	0.0034	0.0056	0.0052	0.0020	0.0000	0.0000	0.0023	0.0033	0.0066	0.0342
HOXA11_P698_F	0.0173	0.6380	0.5885	0.0223	0.1776	0.0209	0.6728	0.0284	0.0444	0.6169	0.0867	0.0117
HOXA5_E187_F	0.9872	0.9850	0.9385	0.9409	0.9915	0.9763	0.0000	0.9883	0.9886	0.0000	0.9908	0.0000
HOXA9_E252_R	0.0090	0.0000	0.0062	0.0241	0.0100	0.1004	0.0184	0.0418	0.0070	0.0060	0.0114	0.1089
HOXA9_P1141_R	0.0641	0.0259	0.0166	0.0161	0.0162	0.0189	0.1995	0.0196	0.0201	0.0213	0.0140	0.1610
HOXA9_P303_F	0.0000	0.0015	0.0000	0.0000	0.0012	0.0000	0.0000	0.0010	0.0000	0.2876	0.0021	0.0000
HOXB13_E21_F	0.0032	0.0070	0.0030	0.0036	0.0045	0.1464	0.0067	0.0033	0.0016	0.0031	0.4119	0.0020
HOXB13_P17_R	0.0023	0.0000	0.3232	0.5824	0.0046	0.2646	0.0000	0.0033	0.0000	0.0037	0.0047	0.0026
HOXC6_P456_R	0.0348	0.1200	0.0300	0.0340	0.3663	0.0766	0.6830	0.0294	0.0479	0.7230	0.0351	0.6154
HPN_P374_R	0.0000	0.0077	0.0000	0.0049	0.0000	0.1848	0.0000	0.0051	0.0105	0.0000	0.0171	0.9899
HPN_P823_F	0.7858	0.0000	0.0000	0.6951	0.0000	0.8435	0.9938	0.0005	0.0000	0.9847	0.0081	0.0000
HPSE_P29_F	0.5876	0.0333	0.0321	0.0252	0.0266	0.0192	0.9933	0.0364	0.0230	0.0204	0.0290	0.4965
HPSE_P93_F	0.0017	0.0039	0.0000	0.2959	0.0078	0.1406	0.0000	0.0013	0.0009	0.0046	0.0061	0.0027
HRASLS_E72_R	0.0000	0.0000	0.0000	0.2109	0.0000	0.0220	0.0000	0.0000	0.0000	0.0000	0.0000	0.0000
HS3ST2_E145_R	0.0000	0.9733	0.0000	0.1987	0.0000	0.0000	0.0000	0.4132	0.0000	0.0036	0.0073	0.0000
HS3ST2_P546_F	0.0000	0.0017	0.3539	0.2099	0.1014	0.0952	0.0101	0.0000	0.0000	0.0000	0.0000	0.0000
HSD17B12_E145_R	0.0256	0.0140	0.0116	0.0176	0.0131	0.0173	0.0109	0.5458	0.0567	0.0166	0.0170	0.0000
HTR1B_P222_F	0.0063	0.0000	0.0000	0.0018	0.0006	0.0000	0.0081	0.0000	0.0000	0.0028	0.0000	0.0024
HTR2A_P853_F	0.0015	0.0015	0.0000	0.1835	0.0000	0.0000	0.0089	0.0023	0.0000	0.0065	0.0069	0.0031
IAPP_E280_F	0.9742	0.0000	0.9766	0.9799	0.0000	0.9757	0.0000	0.9847	0.9846	0.9832	0.0000	0.9904
ICA1_P61_F	0.0000	0.0000	0.0000	0.0000	0.0000	0.0000	0.0000	0.0000	0.0000	0.0000	0.0000	0.0003
ICA1_P72_R	0.0009	0.0000	0.0023	0.0001	0.0000	0.0000	0.0069	0.0015	0.0000	0.0027	0.0000	0.0000
ICAM1_E242_F	0.0082	0.4900	0.3699	0.0099	0.0205	0.0113	0.0586	0.0121	0.0078	0.0089	0.0643	0.2875
ICAM1_P119_R	0.0082	0.0000	0.0025	0.0008	0.0134	0.0903	0.0075	0.0062	0.0000	0.0067	0.0036	0.0000
ICAM1_P386_R	0.0004	0.0000	0.0023	0.0017	0.0045	0.0000	0.0050	0.0000	0.0000	0.0041	0.0000	0.7256
ID1_P659_R	0.0000	0.0000	0.0022	0.0027	0.0039	0.0023	0.0057	0.0000	0.0000	0.0018	0.0058	0.0000
IFNG_P459_R	0.9854	0.8526	0.0203	0.9734	0.2880	0.9610	0.1840	0.6255	0.9858	0.9882	0.9750	0.8967
IFNGR1_P307_F	0.0039	0.1359	0.0105	0.0091	0.0121	0.0088	0.0167	0.0134	0.0026	0.0077	0.0110	0.0070

IFNGR2_E164_F	0.0311	0.0462	0.0321	0.0209	0.0348	0.0296	0.0212	0.0330	0.1145	0.1171	0.0352	0.1569
IFNGR2_P377_R	0.4330	0.0015	0.0000	0.0003	0.0029	0.3136	0.0000	0.0001	0.0008	0.0000	0.0005	0.0000
IGF1_E394_F	0.9873	0.0000	0.7646	0.7907	0.0000	0.9594	0.0000	0.7081	0.0000	0.0000	0.8656	0.8399
IGF1_P933_F	0.0000	0.9862	0.9762	0.9195	0.9836	0.8994	0.0000	0.0000	0.9835	0.0039	0.8359	0.0454
IGF1R_E186_R	0.0011	0.0005	0.0019	0.0023	0.0000	0.0004	0.0083	0.0006	0.0000	0.0002	0.0050	0.0000
IGF1R_P325_R	0.3783	0.0121	0.0147	0.0172	0.4374	0.0175	0.0253	0.1126	0.0134	0.0211	0.0155	0.4474
IGF2_E134_R	0.0000	0.0000	0.0000	0.4705	0.0000	0.0000	0.0000	0.0054	0.0000	0.0006	0.0145	0.0000
IGF2_P1036_R	0.2477	0.0123	0.0121	0.1627	0.0159	0.0149	0.0147	0.0440	0.0176	0.0161	0.0207	0.1815
IGF2_P36_R	0.2683	0.0055	0.2204	0.0120	0.0133	0.0213	0.0117	0.1949	0.0070	0.0180	0.0119	0.1043
IGF2AS_P203_F	0.0000	0.0000	0.0000	0.0000	0.0000	0.1541	0.0000	0.0000	0.0000	0.0000	0.0000	0.0000
IGF2R_P396_R	0.1573	0.2472	0.1840	0.1235	0.1412	0.0706	0.6156	0.2455	0.2494	0.2873	0.2830	0.3669
IGFBP1_E48_R	0.0085	0.4308	0.0053	0.0064	0.0088	0.4240	0.0122	0.0111	0.0125	0.0000	0.0000	0.0027
IGFBP1_P12_R	0.0000	0.3252	0.0123	0.5235	0.0098	0.0028	0.0217	0.0384	0.1450	0.0124	0.0301	0.0042
IGFBP2_P306_F	0.0000	0.0000	0.0000	0.0000	0.0000	0.0000	0.0049	0.0037	0.0000	0.0003	0.0056	0.0007
IGFBP3_E65_R	0.0171	0.0169	0.0305	0.0140	0.0171	0.0152	0.0205	0.1738	0.0111	0.3420	0.0148	0.5800
IGFBP3_P1035_F	0.0000	0.0009	0.0000	0.0000	0.0011	0.0000	0.0000	0.0032	0.0000	0.0000	0.0019	0.0000
IGFBP3_P423_R	0.0080	0.0105	0.2521	0.0944	0.0123	0.0084	0.0103	0.1870	0.0102	0.0122	0.0148	0.0047
IGFBP7_P371_F	0.0045	0.0125	0.0043	0.0056	0.0070	0.0061	0.0078	0.0097	0.1343	0.0131	0.0072	0.0041
IGSF4_P454_F	0.0028	0.0000	0.0063	0.0053	0.0093	0.0036	0.0000	0.0010	0.0001	0.0069	0.0087	0.0062
IGSF4_P86_R	0.0211	0.8851	0.0378	0.0195	0.0130	0.0179	0.8047	0.0198	0.0099	0.0242	0.0176	0.4631
IGSF4C_E65_F	0.9830	0.0000	0.9388	0.8493	0.0000	0.9264	0.0000	0.9898	0.9228	0.9944	0.9861	0.0035
IGSF4C_P533_R	0.0150	0.0000	0.0094	0.0084	0.0126	0.0049	0.0000	0.0093	0.0107	0.0066	0.0080	0.0000
IHH_E186_F	0.0000	0.0000	0.0000	0.0037	0.0033	0.2724	0.0000	0.0000	0.0065	0.0168	0.0141	0.0000
IHH_P246_R	0.0496	0.9855	0.0544	0.1580	0.0140	0.0090	0.0060	0.0067	0.0055	0.0090	0.0338	0.0000
IHH_P529_F	0.0080	0.0000	0.0053	0.0108	0.0100	0.0075	0.0120	0.0184	0.0067	0.0149	0.0103	0.0000
IL10_P85_F	0.0000	0.0026	0.3579	0.1762	0.0047	0.2844	0.0067	0.0000	0.0000	0.0000	0.0044	0.0000
IL11_P11_R	0.2258	0.0050	0.0109	0.0086	0.0000	0.0094	0.0000	0.0000	0.0000	0.0000	0.0000	0.0000
IL12A_E287_R	0.0019	0.0047	0.0020	0.0595	0.0060	0.0013	0.0065	0.0660	0.0895	0.0047	0.0068	0.9615
IL12B_P392_R	0.0000	0.0013	0.1253	0.1900	0.0009	0.5908	0.0028	0.0821	0.0000	0.0002	0.0000	0.0014
IL13_E75_R	0.9872	0.9872	0.9869	0.9808	0.6904	0.9832	0.9938	0.9898	0.9906	0.6660	0.7614	0.9916
IL17RB_E164_R	0.0005	0.0005	0.0005	0.0026	0.0077	0.0885	0.0000	0.0000	0.0000	0.0000	0.0000	0.0000
IL17RB_P788_R	0.0000	0.0000	0.0000	0.0000	0.0000	0.0000	0.0021	0.0000	0.0000	0.0021	0.0000	0.0000
IL18BP_P51_R	0.0210	0.0677	0.0320	0.0271	0.0434	0.0310	0.0464	0.0490	0.0263	0.2795	0.0756	0.0309

IL1A_E113_R	0.9898	0.9929	0.9294	0.9858	0.1398	0.9866	0.9941	0.9907	0.9781	0.8761	0.9456	0.8855
IL1RN_E42_F	0.9852	0.9782	0.9850	0.9821	0.9865	0.9629	0.0000	0.9910	0.9894	0.9679	0.6686	0.9735
IL3_P556_F	0.9844	0.0000	0.9862	0.9753	0.9853	0.9748	0.0000	0.9240	0.8317	0.9877	0.9814	0.0000
IL6_E168_F	0.0071	0.0955	0.0113	0.0086	0.0114	0.2974	0.0172	0.0112	0.0000	0.0099	0.0157	0.0424
IL8_E118_R	0.9859	0.0031	0.0009	0.0019	0.0000	0.0000	0.0000	0.0000	0.0000	0.0057	0.0065	0.0000
IL8_P83_F	0.0000	0.0000	0.9044	0.0000	0.0000	0.4760	0.0000	0.0000	0.0000	0.0000	0.0000	0.0000
IMPACT_P186_F	0.0000	0.0000	0.0000	0.0000	0.0000	0.0000	0.0000	0.0000	0.0000	0.0000	0.0000	0.0018
IMPACT_P234_R	0.0000	0.0000	0.0023	0.0000	0.0000	0.0027	0.0000	0.0000	0.0000	0.0044	0.0051	0.0000
INSR_P1063_R	0.0000	0.0000	0.0000	0.4835	0.0000	0.0000	0.0000	0.0000	0.0017	0.0010	0.7857	0.0026
IPF1_P234_F	0.0004	0.0000	0.0001	0.0066	0.0053	0.5335	0.0000	0.0038	0.0000	0.0000	0.0076	0.0000
IRAK1_P455_R	0.0063	0.0000	0.0112	0.0125	0.0071	0.4136	0.2342	0.0047	0.0042	0.0054	0.0074	0.0000
IRAK3_E130_F	0.0031	0.0000	0.0065	0.0080	0.0119	0.0076	0.0082	0.0073	0.0000	0.0085	0.0135	0.0000
IRAK3_P13_F	0.0068	0.0085	0.0000	0.0056	0.0000	0.0063	0.0138	0.0000	0.0000	0.0088	0.0127	0.0000
IRF5_P123_F	0.0113	0.0054	0.0057	0.0119	0.0097	0.0600	0.0064	0.0162	0.5741	0.0094	0.0215	0.0016
IRF7_P277_R	0.0189	0.4175	0.0093	0.0095	0.3647	0.0409	0.3359	0.0271	0.0052	0.0203	0.0129	0.1797
ISL1_E87_R	0.0089	0.0000	0.0019	0.0063	0.0109	0.1462	0.0106	0.0000	0.0004	0.0100	0.0146	0.0068
ISL1_P379_F	0.0038	0.0000	0.0042	0.0576	0.0071	0.1097	0.0000	0.0000	0.0025	0.0060	0.0815	0.0031
ISL1_P554_F	0.0000	0.0000	0.0000	0.0000	0.0000	0.0000	0.0000	0.0000	0.0000	0.0000	0.0000	0.0000
ITGA2_E120_F	0.0079	0.0119	0.0068	0.0088	0.0112	0.0080	0.0000	0.0057	0.6743	0.0099	0.0126	0.0000
ITGA2_P26_R	0.0017	0.0000	0.0023	0.0000	0.0000	0.0000	0.0000	0.0000	0.0000	0.0000	0.0010	0.0000
ITGB4_E144_F	0.0115	0.0000	0.0017	0.0078	0.0110	0.0035	0.0000	0.0119	0.0000	0.0084	0.0143	0.0000
ITGB4_P517_F	0.0000	0.9696	0.0000	0.3610	0.0000	0.0000	0.0000	0.0000	0.0000	0.0016	0.0000	0.0000
ITPR3_E86_R	0.0000	0.0000	0.0000	0.0000	0.0002	0.0000	0.0046	0.0000	0.0000	0.0046	0.0000	0.0000
ITPR3_P1112_F	0.0000	0.0000	0.0000	0.0021	0.0057	0.0000	0.0000	0.0004	0.0000	0.0017	0.0012	0.0000
JAG2_E54_F	0.0028	0.0045	0.0057	0.0063	0.0072	0.0508	0.0000	0.0092	0.0049	0.0066	0.0053	0.0000
JAG2_P264_F	0.0138	0.2809	0.0109	0.0137	0.0177	0.0114	0.0134	0.0269	0.0083	0.0107	0.0070	0.3762
JAK2_P772_R	0.0291	0.0249	0.0103	0.0120	0.2785	0.0127	0.0000	0.0105	0.0055	0.0130	0.0000	0.1813
JAK3_E64_F	0.0043	0.0000	0.0049	0.0150	0.0060	0.0947	0.0000	0.0100	0.0012	0.0053	0.0134	0.0000
JAK3_P156_R	0.0129	0.0130	0.0084	0.1803	0.0115	0.0169	0.1043	0.0142	0.0066	0.0136	0.0127	0.0115
JUNB_P1149_R	0.5006	0.4723	0.5447	0.0686	0.1725	0.0657	0.1306	0.1616	0.0637	0.5992	0.1199	0.5234
KCNK4_E3_F	0.7023	0.0002	0.5012	0.0066	0.0068	0.1866	0.0038	0.0031	0.0000	0.8213	0.0204	0.0019
KDR_E79_F	0.0298	0.0621	0.0137	0.0087	0.6966	0.0101	0.7983	0.0225	0.0681	0.0246	0.0187	0.0300
KDR_P445_R	0.0004	0.0000	0.0050	0.0088	0.0057	0.0050	0.0000	0.0000	0.0000	0.0066	0.0000	0.0052

KIAA1804_P689_R	0.0099	0.0125	0.0361	0.0122	0.0171	0.0077	0.0124	0.0072	0.0036	0.0109	0.0117	0.1507
KIT_P367_R	0.0074	0.0027	0.0027	0.0061	0.0065	0.0046	0.0057	0.0046	0.0000	0.0056	0.3413	0.0000
KIT_P405_F	0.0056	0.0010	0.0056	0.0601	0.4956	0.0664	0.0000	0.4450	0.0017	0.0094	0.0109	0.0068
KLF5_E190_R	0.0020	0.0000	0.7250	0.0048	0.0080	0.0877	0.0000	0.0039	0.0000	0.0043	0.0050	0.0025
KLK11_P103_R	0.0000	0.8502	0.8759	0.7935	0.0000	0.9673	0.0000	0.0064	0.9811	0.0000	0.0050	0.0002
KLK11_P1290_F	0.9875	0.0000	0.9826	0.9783	0.0000	0.9304	0.0000	0.0000	0.0011	0.9848	0.8817	0.0000
KRAS_E82_F	0.8426	0.9465	0.5058	0.6217	0.8815	0.6901	0.1859	0.7822	0.2635	0.0026	0.5727	0.9899
KRAS_P651_F	0.0640	0.5145	0.1043	0.0692	0.0872	0.0459	0.2082	0.0640	0.1509	0.1103	0.4065	0.1466
KRT13_P341_R	0.9885	0.9743	0.6710	0.9816	0.0100	0.9757	0.9942	0.0000	0.9861	0.9533	0.9422	0.9903
KRT5_P308_F	0.7887	0.2776	0.8153	0.8545	0.8783	0.8466	0.2094	0.1432	0.1405	0.7817	0.2439	0.9703
L1CAM_P148_R	0.0044	0.0329	0.0094	0.0032	0.0114	0.0027	0.0000	0.0000	0.0006	0.0049	0.0075	0.0036
L1CAM_P19_F	0.5987	0.0000	0.0030	0.6788	0.3817	0.1756	0.0000	0.0021	0.0060	0.0075	0.0085	0.0000
LAMC1_E466_R	0.0063	0.0048	0.0060	0.0028	0.0000	0.0011	0.0000	0.0044	0.0008	0.0054	0.0066	0.0054
LIF_E208_F	0.1198	0.2127	0.0094	0.0121	0.0083	0.0068	0.1876	0.0135	0.0068	0.0094	0.0285	0.0094
LIF_P383_R	0.0237	0.0056	0.0000	0.0094	0.0018	0.3670	0.0059	0.0000	0.0000	0.0093	0.0107	0.0000
LIG4_P194_F	0.0056	0.0063	0.0084	0.0098	0.0085	0.0098	0.0099	0.0109	0.0010	0.0092	0.0211	0.0000
LMO1_E265_R	0.0000	0.0000	0.0027	0.0044	0.0034	0.0000	0.0000	0.0056	0.0000	0.0004	0.8148	0.0025
LMO1_P169_F	0.0068	0.0049	0.0079	0.0075	0.0093	0.0063	0.0087	0.0061	0.0069	0.0075	0.0118	0.0000
LOX_P313_R	0.0142	0.1133	0.0113	0.0174	0.0220	0.1581	0.1390	0.0174	0.0081	0.0231	0.0205	0.0992
LOX_P71_F	0.0011	0.0023	0.0013	0.0031	0.0067	0.0013	0.0058	0.0077	0.0033	0.0019	0.0073	0.0041
LRP2_E20_F	0.0333	0.0545	0.0682	0.0320	0.0996	0.0276	0.0283	0.0288	0.0246	0.0415	0.0253	0.5462
LRRC32_P865_R	0.0025	0.0068	0.0048	0.0050	0.0098	0.0540	0.0103	0.0081	0.0005	0.0105	0.0104	0.0059
LTB4R_E64_R	0.0624	0.4781	0.0262	0.1958	0.6274	0.0420	0.6093	0.0830	0.5796	0.0534	0.0329	0.0290
LYN_E353_F	0.0000	0.0000	0.0000	0.4026	0.0000	0.0000	0.0000	0.0000	0.0000	0.0085	0.0000	0.0000
LYN_P241_F	0.2973	0.0585	0.0388	0.0787	0.0347	0.0318	0.0846	0.0327	0.1145	0.0405	0.0308	0.0509
MAF_P826_R	0.0000	0.0000	0.0000	0.0000	0.0000	0.0000	0.0007	0.0000	0.0000	0.0000	0.0015	0.0000
MAGEC3_P903_F	0.0000	0.0000	0.0000	0.6315	0.0000	0.9449	0.0000	0.0000	0.0000	0.0000	0.0000	0.0000
MAGEL2_E166_R	0.0000	0.0000	0.0000	0.9262	0.9818	0.9642	0.0000	0.0179	0.0012	0.9789	0.0088	0.0993
MALT1_P406_R	0.0004	0.0027	0.0023	0.0042	0.0028	0.0019	0.0000	0.0000	0.0000	0.0055	0.0036	0.0060
MAP2K6_E297_F	0.0000	0.0023	0.0000	0.0003	0.0029	0.1601	0.0000	0.0000	0.0000	0.0025	0.0050	0.0000
MAP2K6_P297_R	0.0127	0.0136	0.3409	0.0153	0.0162	0.0700	0.1246	0.3619	0.0159	0.7351	0.0185	0.0119
MAP3K1_P7_F	0.5941	0.0104	0.6030	0.0289	0.0317	0.0297	0.6257	0.0157	0.0271	0.0217	0.0189	0.1076
MAP3K9_E17_R	0.0000	0.0000	0.0372	0.0199	0.0118	0.0216	0.0081	0.0120	0.4527	0.0681	0.2668	0.0000

MAPK12_E165_R	0.0066	0.0080	0.0077	0.0079	0.0116	0.0055	0.0081	0.0042	0.0044	0.0067	0.0142	0.0044
MAPK12_P416_F	0.0000	0.0000	0.0000	0.0004	0.0000	0.0000	0.0000	0.0000	0.0000	0.0000	0.0013	0.0000
MAPK14_P327_R	0.0125	0.0023	0.0040	0.0027	0.0047	0.0000	0.8790	0.0150	0.0000	0.0621	0.0508	0.0080
MAPK4_E273_R	0.7201	0.0072	0.2240	0.8860	0.0000	0.8615	0.9954	0.9826	0.9882	0.0000	0.9840	0.0000
MAPK9_P1175_F	0.0000	0.9885	0.9830	0.9810	0.0000	0.9724	0.9921	0.9908	0.9288	0.0000	0.0000	0.0000
MAS1_P469_R	0.9747	0.9701	0.7388	0.9825	0.5564	0.9815	0.9918	0.6472	0.7309	0.9895	0.9790	0.9595
MATK_P64_F	0.0056	0.0631	0.0060	0.0078	0.0085	0.0711	0.0665	0.0067	0.0058	0.0059	0.0097	0.0570
MC2R_P1025_F	0.0000	0.0000	0.9703	0.9187	0.7144	0.9413	0.0000	0.0000	0.0000	0.0000	0.0294	0.0000
MCAM_P169_R	0.0044	0.0091	0.0051	0.1072	0.0108	0.0048	0.0041	0.0000	0.0014	0.0089	0.0054	0.0000
MCAM_P265_R	0.4624	0.0095	0.6482	0.1119	0.0165	0.0166	0.0073	0.0598	0.0073	0.0143	0.0376	0.0583
MCC_P196_R	0.0000	0.0000	0.0010	0.0000	0.0000	0.0000	0.0038	0.0000	0.0000	0.0015	0.0000	0.0000
MCM2_P241_R	0.0071	0.0000	0.0046	0.0617	0.0110	0.0055	0.0673	0.0090	0.0032	0.0088	0.0128	0.0058
MDR1_seq_42_S300_R	0.0058	0.0071	0.0103	0.0078	0.0087	0.0035	0.0380	0.0000	0.0063	0.0095	0.0085	0.0000
MDS1_E45_F	0.0036	0.0000	0.0062	0.0077	0.0140	0.0113	0.3593	0.0064	0.0169	0.0124	0.0117	0.0137
MECP2_E90_R	0.8400	0.0071	0.0060	0.1201	0.8119	0.2628	0.0000	0.0088	0.0025	0.0148	0.0092	0.0050
MEG3_E91_F	0.0000	0.0000	0.0000	0.0001	0.0083	0.2906	0.0000	0.0000	0.0000	0.0000	0.0064	0.0000
MEST_E150_F	0.0006	0.0000	0.0000	0.0013	0.0011	0.0003	0.0034	0.9872	0.0000	0.0009	0.0072	0.0000
MEST_P4_F	0.0006	0.0000	0.0000	0.0027	0.0047	0.0027	0.0000	0.0000	0.0000	0.0030	0.0043	0.0000
MEST_P62_R	0.0000	0.0000	0.0667	0.0004	0.0037	0.0000	0.0000	0.0000	0.0000	0.0000	0.0083	0.0010
MET_E333_F	0.9788	0.0000	0.0201	0.7155	0.9817	0.2008	0.0000	0.0143	0.0463	0.5997	0.0245	0.0313
MFAP4_P197_F	0.0009	0.0000	0.0000	0.3249	0.0047	0.0257	0.0000	0.0000	0.0010	0.0064	0.0037	0.0000
MGMT_P272_R	0.0000	0.0000	0.0057	0.3282	0.0015	0.0006	0.0000	0.0011	0.0000	0.0000	0.0000	0.0036
MGMT_P281_F	0.0155	0.5380	0.0091	0.1495	0.0110	0.1084	0.0167	0.4366	0.0139	0.0150	0.0177	0.0081
MKRN3_P108_F	0.9730	0.0000	0.9750	0.9715	0.9732	0.9653	0.0000	0.9718	0.9835	0.9744	0.9800	0.0000
MLF1_P97_F	0.0130	0.0105	0.0000	0.0956	0.0143	0.0074	0.0099	0.0089	0.0068	0.0096	0.0100	0.0354
MLH1_P381_F	0.0722	0.0036	0.0058	0.0050	0.0139	0.0033	0.0000	0.3792	0.0069	0.0091	0.0114	0.0035
MLH3_E72_F	0.0000	0.0000	0.0011	0.0046	0.0095	0.0009	0.0000	0.0043	0.0000	0.0047	0.0052	0.0052
MLH3_P25_F	0.0005	0.0024	0.1084	0.1855	0.0037	0.1322	0.0000	0.0064	0.2765	0.0029	0.0095	0.9907
MLLT3_E93_R	0.0015	0.0014	0.0000	0.0022	0.0057	0.0000	0.0076	0.0000	0.0000	0.0049	0.0081	0.0044
MLLT4_P1400_F	0.0140	0.0196	0.0178	0.0258	0.0218	0.0183	0.0192	0.0122	0.0150	0.1826	0.0311	0.0139
MLLT6_P957_F	0.0004	0.0000	0.0000	0.0010	0.0000	0.0000	0.0000	0.0000	0.0000	0.0000	0.0029	0.0000
MME_E29_F	0.0076	0.0129	0.0040	0.0000	0.0059	0.0000	0.0009	0.0041	0.9587	0.0046	0.0086	0.0000
MME_P388_F	0.0000	0.0000	0.0000	0.0000	0.0000	0.0000	0.0000	0.0000	0.0000	0.0000	0.0003	0.6987

MMP19_E274_R	0.9909	0.9624	0.9872	0.9482	0.9514	0.9838	0.9926	0.9350	0.8758	0.9921	0.8495	0.9930
MMP2_E21_R	0.9150	0.0000	0.0020	0.1738	0.0042	0.2329	0.0050	0.0000	0.0000	0.0087	0.0223	0.0018
MMP2_P197_F	0.0074	0.0000	0.0052	0.0071	0.0074	0.0040	0.0000	0.0092	0.0026	0.0088	0.0087	0.0000
MMP2_P303_R	0.0056	0.0081	0.0080	0.0085	0.0133	0.0169	0.0058	0.2215	0.0065	0.0085	0.3249	0.3380
MMP3_P16_R	0.9836	0.9860	0.8357	0.9804	0.0000	0.9758	0.0000	0.9868	0.9087	0.9913	0.9861	0.0000
MMP7_P613_F	0.9203	0.9304	0.9528	0.6960	0.3283	0.8639	0.1837	0.8906	0.9388	0.8726	0.9703	0.2254
MMP9_P189_F	0.0060	0.0150	0.0071	0.0120	0.0155	0.2663	0.0000	0.0075	0.0042	0.0000	0.0154	0.1754
MMP9_P237_R	0.0095	0.0125	0.0079	0.1239	0.3669	0.0101	0.0079	0.0077	0.0101	0.4092	0.0130	0.0041
MOS_E60_R	0.0036	0.0129	0.0043	0.3473	0.0088	0.1560	0.0551	0.0082	0.0175	0.0145	0.0083	0.0035
MPO_P883_R	0.0068	0.0024	0.0021	0.1175	0.0047	0.0848	0.0000	0.0016	0.0035	0.0023	0.0060	0.9902
MSH3_E3_F	0.9684	0.9884	0.9863	0.8797	0.9828	0.9416	0.9825	0.0000	0.9693	0.6604	0.8826	0.0000
MSH3_P13_R	0.8387	0.8834	0.7348	0.6582	0.0129	0.5041	0.7799	0.0112	0.6962	0.8508	0.2835	0.9869
MST1R_P392_F	0.0000	0.0000	0.0000	0.0000	0.0022	0.0000	0.0016	0.0000	0.0000	0.0000	0.0000	0.0000
MT1A_E13_R	0.0364	0.0083	0.0070	0.0434	0.0115	0.1081	0.0131	0.0666	0.2284	0.0085	0.4128	0.0048
MT1A_P49_R	0.0000	0.0000	0.0000	0.0999	0.0008	0.0000	0.0000	0.0000	0.0000	0.0000	0.0023	0.0000
MTA1_P478_F	0.1860	0.0156	0.0084	0.2498	0.0928	0.0134	0.0108	0.0112	0.1352	0.1505	0.0220	0.1082
MUC1_E18_R	0.9449	0.0047	0.0000	0.7682	0.0000	0.7017	0.0000	0.0000	0.0000	0.0603	0.0054	0.0000
MUC1_P191_F	0.0018	0.0000	0.0000	0.0068	0.0000	0.0052	0.0000	0.0083	0.0000	0.0000	0.0058	0.0049
MXI1_P75_R	0.0016	0.0064	0.0054	0.0029	0.0082	0.2648	0.0154	0.0076	0.0000	0.0070	0.0098	0.0010
MYBL2_P211_F	0.0000	0.8672	0.0000	0.0089	0.0128	0.0273	0.0094	0.0787	0.0000	0.0181	0.0115	0.0065
MYCL1_P502_R	0.0000	0.0000	0.0000	0.0000	0.0070	0.1523	0.0094	0.0042	0.0000	0.0027	0.0059	0.0000
MYCN_E77_R	0.0102	0.0074	0.0113	0.1009	0.0293	0.0734	0.0127	0.0107	0.0038	0.0163	0.0222	0.0000
MYCN_P464_R	0.0057	0.0037	0.0058	0.0075	0.0080	0.0032	0.0000	0.0129	0.0009	0.0059	0.0072	0.0000
MYH11_P22_F	0.0027	0.0000	0.0035	0.0048	0.0074	0.1496	0.0750	0.0095	0.0035	0.0000	0.0090	0.0348
MYH11_P236_R	0.0000	0.0000	0.0000	0.0000	0.0000	0.0000	0.0000	0.0000	0.0000	0.0000	0.0000	0.0000
MYLK_P469_R	0.0086	0.0160	0.0095	0.0114	0.9836	0.1941	0.0093	0.0109	0.0064	0.0097	0.0097	0.4278
MYOD1_E156_F	0.0014	0.0000	0.0021	0.0023	0.0063	0.0686	0.0000	0.0000	0.0000	0.0039	0.0048	0.0006
MYOD1_P50_F	0.0000	0.0000	0.0000	0.0000	0.0013	0.0000	0.0000	0.0000	0.0000	0.0000	0.0000	0.0025
NBL1_E205_R	0.9884	0.1284	0.8571	0.8578	0.9400	0.9821	0.9193	0.0583	0.0369	0.9879	0.7605	0.8561
NBL1_P24_F	0.9873	0.0000	0.0416	0.8084	0.1297	0.9349	0.9891	0.0117	0.4603	0.9741	0.7766	0.9838
NCL_P840_R	0.0096	0.0000	0.0041	0.0071	0.0116	0.0070	0.0160	0.0150	0.0012	0.0107	0.0105	0.0118
NDN_P1110_F	0.9736	0.9498	0.9805	0.9640	0.9779	0.9694	0.0000	0.0000	0.0000	0.9772	0.9784	0.9816
NEFL_E23_R	0.6641	0.9939	0.9702	0.5682	0.8697	0.4783	0.9616	0.9941	0.9863	0.9950	0.8536	0.9920

NEFL_P209_R	0.0000	0.0000	0.0000	0.0000	0.0000	0.0000	0.0033	0.0000	0.0000	0.0007	0.0018	0.0000
NEO1_P1067_F	0.0019	0.0000	0.0000	0.4649	0.0000	0.0000	0.0213	0.0000	0.0000	0.0000	0.0000	0.0000
NES_P239_R	0.0000	0.0000	0.0000	0.0000	0.0000	0.3304	0.0000	0.0000	0.0029	0.0000	0.0048	0.0023
NEU1_P745_F	0.0083	0.0051	0.0000	0.0047	0.0028	0.0021	0.0000	0.0018	0.0041	0.0053	0.0028	0.0000
NFKB1_P336_R	0.0120	0.0279	0.0098	0.0209	0.0286	0.0070	0.3971	0.0000	0.0000	0.0149	0.9801	0.0000
NFKB2_P709_R	0.0195	0.0126	0.3416	0.0287	0.0528	0.0267	0.1832	0.0133	0.0118	0.0295	0.0355	0.2536
NGFB_E353_F	0.0020	0.0015	0.0019	0.0742	0.0069	0.0000	0.0000	0.0014	0.0000	0.0068	0.0083	0.6968
NGFB_P13_F	0.5225	0.3535	0.1448	0.1143	0.0338	0.1879	0.1522	0.1104	0.1800	0.0394	0.1643	0.2920
NGFR_E328_F	0.0000	0.0000	0.0045	0.0018	0.0202	0.1544	0.0000	0.0000	0.0000	0.0013	0.0104	0.0000
NGFR_P355_F	0.0595	0.1152	0.0545	0.3012	0.0756	0.0550	0.0795	0.0531	0.2816	0.0818	0.0647	0.0451
NKX3-1_P146_F	0.0072	0.0000	0.0042	0.0074	0.7643	0.0078	0.0000	0.0000	0.0022	0.0000	0.0129	0.0000
NKX3-1_P871_R	0.9876	0.9769	0.7536	0.9753	0.9826	0.9812	0.0049	0.9854	0.9851	0.9783	0.9848	0.9922
NOTCH1_E452_R	0.0000	0.0000	0.0000	0.0000	0.0000	0.0000	0.0000	0.0000	0.0000	0.0000	0.0000	0.0000
NOTCH1_P1198_F	0.8108	0.0000	0.0000	0.1045	0.0000	0.1223	0.0000	0.0000	0.0000	0.0000	0.0048	0.0003
NOTCH2_P312_R	0.0040	0.0058	0.0021	0.0035	0.0055	0.0017	0.0089	0.0049	0.0009	0.0047	0.0060	0.0034
NOTCH3_E403_F	0.0000	0.0000	0.0000	0.5305	0.0000	0.0000	0.0000	0.0000	0.0000	0.0000	0.0000	0.0000
NPR2_P618_F	0.0000	0.0000	0.9530	0.6402	0.0201	0.2963	0.0000	0.0051	0.0072	0.0046	0.0105	0.0000
NPY_P295_F	0.0230	0.8069	0.5498	0.0324	0.0540	0.0199	0.5564	0.0519	0.0198	0.0318	0.0311	0.4415
NPY_P91_F	0.0079	0.1114	0.0216	0.0206	0.0187	0.0148	0.1063	0.0151	0.0092	0.1074	0.0168	0.0127
NQO1_E74_R	0.0000	0.0000	0.0034	0.0064	0.0077	0.0057	0.0084	0.0042	0.0001	0.0119	0.0105	0.0000
NQO1_P345_R	0.0000	0.0030	0.0000	0.0703	0.0041	0.0000	0.0004	0.0042	0.0000	0.0000	0.0039	0.0021
NR2F6_E375_R	0.0054	0.0104	0.0116	0.0099	0.0094	0.0557	0.0107	0.0171	0.0300	0.0050	0.0098	0.0158
NRAS_P103_R	0.0000	0.0021	0.0023	0.0005	0.0034	0.0000	0.0000	0.0000	0.0017	0.0000	0.0000	0.0000
NRAS_P12_R	0.0000	0.0000	0.0000	0.0000	0.0000	0.0000	0.0000	0.0000	0.0000	0.0000	0.0000	0.0000
NRG1_E74_F	0.0000	0.0000	0.0000	0.0034	0.0001	0.0872	0.0000	0.0001	0.0000	0.7531	0.0069	0.0000
NRG1_P558_R	0.0051	0.0114	0.0064	0.0056	0.0056	0.0025	0.0067	0.0750	0.0023	0.0056	0.5759	0.0277
NTRK2_P10_F	0.0000	0.0480	0.0088	0.0072	0.0102	0.0061	0.0028	0.0052	0.0101	0.0046	0.0076	0.0040
NTRK2_P395_R	0.0000	0.0000	0.0000	0.0866	0.0045	0.0000	0.0000	0.0019	0.0000	0.0016	0.0000	0.0000
NTRK3_E131_F	0.0034	0.0000	0.0004	0.0016	0.0088	0.0000	0.0000	0.0008	0.0000	0.0065	0.0083	0.0032
NTRK3_P636_R	0.0000	0.0000	0.0000	0.1512	0.0000	0.0000	0.0000	0.0000	0.0000	0.0000	0.0000	0.0000
NTRK3_P752_F	0.0000	0.0050	0.0011	0.0000	0.0000	0.0000	0.0000	0.0025	0.0000	0.0000	0.0025	0.0000
NTSR1_E109_F	0.0097	0.0078	0.0042	0.0082	0.0084	0.0053	0.0084	0.0071	0.0020	0.0054	0.0160	0.0097
NTSR1_P318_F	0.0190	0.0268	0.5585	0.0166	0.0071	0.0127	0.0845	0.0093	0.0040	0.0113	0.0437	0.7313

OAT_P465_F	0.7767	0.0110	0.9072	0.4833	0.0138	0.2500	0.3295	0.0165	0.0057	0.0105	0.4973	0.3166
ODC1_P424_F	0.0000	0.0000	0.0000	0.0000	0.0040	0.0000	0.0000	0.0000	0.0000	0.0046	0.0046	0.0004
ONECUT2_E96_F	0.0582	0.0090	0.0343	0.0304	0.0000	0.0258	0.0000	0.0000	0.4845	0.3684	0.0439	0.2188
ONECUT2_P315_R	0.0000	0.0000	0.0000	0.0011	0.0081	0.0000	0.0050	0.0059	0.0000	0.0000	0.0000	0.0038
OPCML_E219_R	0.0010	0.0063	0.0035	0.1117	0.0066	0.1395	0.0000	0.0045	0.0001	0.0051	0.0085	0.0038
OPCML_P71_F	0.0083	0.0049	0.0000	0.2971	0.3891	0.0013	0.0095	0.0062	0.0022	0.0091	0.0063	0.0022
OSM_P188_F	0.0113	0.0085	0.0109	0.0117	0.0176	0.0862	0.1048	0.0091	0.0113	0.0097	0.0136	0.0071
p16_seq_47_S188_R	0.0057	0.0060	0.0043	0.0047	0.1874	0.0034	0.0075	0.0063	0.0036	0.0097	0.0125	0.0000
P2RX7_E323_R	0.0431	0.0024	0.0036	0.2173	0.0071	0.0106	0.0019	0.0083	0.0037	0.0037	0.0048	0.0022
P2RX7_P119_R	0.2153	0.2401	0.0173	0.2365	0.0346	0.0345	0.0161	0.0192	0.0185	0.0213	0.0379	0.0118
P2RX7_P597_F	0.9898	0.3653	0.9207	0.9870	0.9898	0.9855	0.9947	0.9796	0.9924	0.8061	0.9216	0.9919
PALM2-AKAP2_P183_R	0.0256	0.0611	0.0273	0.0255	0.0291	0.0188	0.5818	0.0321	0.0303	0.0366	0.0514	0.5514
PALM2-AKAP2_P420_R	0.0000	0.0164	0.0000	0.1067	0.6042	0.0024	0.0000	0.0000	0.0311	0.0000	0.0259	0.0000
PARP1_P610_R	0.4700	0.9312	0.9717	0.6959	0.5200	0.5257	0.4737	0.8244	0.3954	0.5192	0.7635	0.1985
PAX6_P1121_F	0.0017	0.0000	0.0191	0.0056	0.0102	0.0069	0.0135	0.0129	0.0108	0.0101	0.0214	0.0159
PAX6_P50_R	0.0000	0.0000	0.0016	0.0000	0.0068	0.1438	0.0000	0.0000	0.0000	0.0040	0.0017	0.0000
PCDH1_E22_F	0.0063	0.0000	0.0115	0.0117	0.0146	0.0100	0.0000	0.0134	0.0024	0.0000	0.0886	0.3027
PCGF4_P760_R	0.0123	0.0108	0.0095	0.2728	0.0171	0.0116	0.0394	0.0086	0.0111	0.0074	0.0172	0.0725
PCGF4_P92_R	0.0485	0.0358	0.0108	0.0095	0.0084	0.0046	0.0244	0.0061	0.0122	0.0108	0.0096	0.0164
PCTK1_E77_R	0.9942	0.9442	0.9418	0.7150	0.9486	0.6759	0.9980	0.9644	0.9933	0.9766	0.9944	0.8989
PDE1B_E141_F	0.0115	0.0217	0.0112	0.0140	0.0121	0.0747	0.0152	0.0120	0.0097	0.0168	0.0223	0.3140
PDE1B_P263_R	0.0109	0.0073	0.0061	0.1004	0.0087	0.0095	0.0062	0.0533	0.0013	0.0000	0.0166	0.0065
PDGFA_P841_R	0.0000	0.0000	0.0000	0.0034	0.0050	0.0000	0.0081	0.0000	0.0008	0.0000	0.0068	0.0000
PDGFRB_E195_R	0.0064	0.0089	0.0090	0.0082	0.0072	0.0046	0.0099	0.0078	0.0018	0.0075	0.0123	0.0061
PDGFRB_P343_F	0.0034	0.0034	0.0000	0.0000	0.0033	0.0000	0.0056	0.0019	0.0000	0.0012	0.0016	0.0017
PEG10_P978_R	0.6985	0.0000	0.7297	0.0374	0.6380	0.0186	0.0000	0.0124	0.0358	0.8514	0.0821	0.0000
PENK_E26_F	0.0328	0.1867	0.3197	0.0907	0.0293	0.1844	0.0459	0.0342	0.0774	0.0257	0.2957	0.0370
PENK_P447_R	0.0080	0.0808	0.0187	0.0049	0.0078	0.0033	0.2741	0.0056	0.0035	0.0082	0.0125	0.0073
PGF_E33_F	0.0053	0.0136	0.0119	0.0131	0.0258	0.0344	0.0295	0.0179	0.0497	0.0192	0.0216	0.0085
PHLDA2_P622_F	0.7440	0.0000	0.0016	0.0056	0.0033	0.1284	0.0084	0.0000	0.0018	0.0078	0.0000	0.0000
PI3_P1394_R	0.0180	0.0192	0.9699	0.9627	0.0362	0.9455	0.4396	0.2967	0.3464	0.0462	0.9486	0.4685
PITX2_E24_R	0.4689	0.0168	0.8399	0.0130	0.6490	0.0158	0.0136	0.0090	0.0170	0.0110	0.0109	0.0693
PITX2_P183_R	0.0000	0.0000	0.0000	0.0000	0.0001	0.1027	0.0000	0.0000	0.0000	0.0017	0.0000	0.0000

PKD2_P287_R	0.0109	0.0065	0.0109	0.0119	0.0126	0.0081	0.0000	0.0112	0.0110	0.1805	0.0255	0.0024
PKD2_P336_R	0.0083	0.0091	0.0149	0.0983	0.0136	0.0665	0.0074	0.0093	0.0084	0.0096	0.0147	0.0057
PLAU_P11_F	0.0266	0.3616	0.0312	0.0234	0.3174	0.1265	0.0177	0.0193	0.0191	0.0296	0.0300	0.2728
PLAU_P176_R	0.0026	0.0000	0.0000	0.0027	0.0059	0.0000	0.0000	0.0000	0.0017	0.0000	0.0000	0.0000
PLAUR_E123_F	0.0021	0.0000	0.0008	0.1847	0.0053	0.0661	0.0058	0.0036	0.0031	0.0038	0.0069	0.0024
PLAUR_P82_F	0.0000	0.0032	0.0000	0.0015	0.0000	0.0000	0.0000	0.0000	0.0005	0.0034	0.0014	0.0014
PLG_E406_F	0.9228	0.8817	0.8390	0.9425	0.6937	0.9894	0.9170	0.9519	0.9833	0.9494	0.8597	0.9953
PLSCR3_P751_R	0.0090	0.0144	0.0117	0.2639	0.0203	0.2671	0.0209	0.0181	0.0077	0.5214	0.2407	0.0121
PLXDC1_P236_F	0.0149	0.0000	0.0077	0.0096	0.0134	0.0100	0.0697	0.0093	0.0110	0.0143	0.0208	0.6695
PLXDC2_E337_F	0.0016	0.0000	0.0034	0.1036	0.0113	0.0409	0.0130	0.0068	0.0011	0.0058	0.0067	0.0061
PLXDC2_P914_R	0.0408	0.3804	0.0903	0.0275	0.0414	0.0284	0.3574	0.0209	0.0280	0.0301	0.0882	0.3783
PMP22_P975_F	0.9178	0.0000	0.0000	0.9374	0.9844	0.9781	0.0000	0.0000	0.0000	0.0000	0.0000	0.0000
PODXL_P1341_R	0.0060	0.0000	0.0083	0.0971	0.0082	0.0233	0.1880	0.0000	0.0051	0.0088	0.0109	0.0638
POMC_E254_F	0.0122	0.0098	0.0096	0.0147	0.2741	0.0631	0.0000	0.0058	0.9870	0.7380	0.0123	0.0000
POMC_P400_R	0.0000	0.0067	0.0047	0.0067	0.0163	0.3420	0.0000	0.0043	0.0000	0.0051	0.0049	0.0000
POMC_P53_F	0.0198	0.0498	0.0059	0.0091	0.1942	0.0094	0.0000	0.0089	0.0074	0.0649	0.0146	0.1460
PPARD_P846_F	0.0000	0.0000	0.0000	0.7909	0.0000	0.0000	0.0000	0.0053	0.0000	0.0000	0.0000	0.0000
PPARG_E178_R	0.0051	0.0000	0.0051	0.0057	0.0046	0.0042	0.0336	0.0000	0.0000	0.0018	0.0024	0.0033
PPP2R1B_P268_R	0.0000	0.0000	0.0008	0.4512	0.0092	0.0006	0.8292	0.0060	0.0000	0.0059	0.0087	0.0000
PRDM2_P1340_R	0.9633	0.0000	0.9778	0.9560	0.9843	0.9689	0.0000	0.9892	0.9861	0.9740	0.0756	0.9887
PRKCDBP_E206_F	0.0000	0.0000	0.0030	0.0000	0.0069	0.0015	0.0000	0.0045	0.0013	0.0057	0.0155	0.0000
PROK2_E0_F	0.0000	0.0000	0.0019	0.0009	0.0071	0.1092	0.0000	0.0043	0.0000	0.0025	0.0072	0.0000
PROK2_P390_F	0.0000	0.0000	0.0000	0.0000	0.0023	0.0000	0.0000	0.0010	0.0000	0.0020	0.0000	0.0000
PRSS8_E134_R	0.9875	0.6316	0.9867	0.9857	0.9168	0.9846	0.0655	0.9890	0.9863	0.2095	0.6670	0.1539
PSCA_E359_F	0.0000	0.0000	0.0025	0.1950	0.0157	0.2283	0.0054	0.0000	0.0000	0.0600	0.0102	0.0013
PSIP1_P163_R	0.0000	0.0022	0.0000	0.0028	0.0055	0.1574	0.0081	0.0109	0.0025	0.0095	0.0076	0.0067
PTCH_E42_F	0.0016	0.0000	0.0075	0.0110	0.0116	0.0091	0.0081	0.0216	0.0126	0.0112	0.0118	0.0039
PTCH2_P568_R	0.0068	0.5184	0.8829	0.3014	0.7090	0.2260	0.0121	0.0095	0.0467	0.0097	0.4141	0.0325
PTEN_P438_F	0.0041	0.0000	0.0073	0.0064	0.0102	0.0027	0.1741	0.0069	0.0067	0.0075	0.0084	0.0000
PTGS1_P2_F	0.0082	0.0000	0.0053	0.0101	0.0000	0.0054	0.0190	0.0134	0.0052	0.0123	0.0094	0.0029
PTGS2_P308_F	0.0056	0.0107	0.0030	0.0060	0.0035	0.0025	0.1328	0.0045	0.0020	0.0047	0.0055	0.0048
PTGS2_P524_R	0.0043	0.0000	0.0086	0.0496	0.0109	0.0061	0.0129	0.0091	0.0036	0.0057	0.0121	0.0051
PTHLH_P15_R	0.0000	0.0041	0.0000	0.0012	0.0000	0.4256	0.0000	0.0000	0.0000	0.0000	0.0001	0.0016

PTHR1_P170_R	0.9743	0.6961	0.0000	0.9832	0.8959	0.8831	0.9917	0.0108	0.0052	0.0000	0.7122	0.0000
PTK2_P735_R	0.0084	0.0077	0.1588	0.0116	0.0139	0.0081	0.0084	0.0118	0.2682	0.0114	0.0198	0.0037
PTK2B_P673_R	0.0111	0.0002	0.0030	0.0048	0.0182	0.0036	0.0000	0.0056	0.0002	0.0000	0.0088	0.0000
PTPN6_E171_R	0.0000	0.0000	0.0000	0.0000	0.0009	0.0000	0.0000	0.0000	0.0000	0.0000	0.0000	0.0000
PTPN6_P282_R	0.0000	0.0000	0.0000	0.4304	0.0000	0.2526	0.0000	0.0000	0.0000	0.0000	0.9807	0.0000
PTPNS1_E433_R	0.0000	0.0000	0.0000	0.0000	0.0008	0.0000	0.0000	0.0000	0.0000	0.0000	0.0000	0.0000
PTPNS1_P301_R	0.0091	0.0000	0.0070	0.0095	0.0095	0.0072	0.0000	0.0097	0.0013	0.0076	0.0000	0.0000
PTPRF_E178_R	0.0000	0.0000	0.0030	0.0057	0.0063	0.2216	0.0000	0.5770	0.6739	0.0071	0.7506	0.0000
PTPRG_E40_R	0.0064	0.5054	0.0222	0.0096	0.3515	0.0074	0.2464	0.3515	0.2673	0.4148	0.0222	0.1666
PTPRG_P476_F	0.0053	0.0000	0.0058	0.0066	0.0132	0.0035	0.1146	0.0056	0.0067	0.0057	0.0053	0.0000
PTPRO_E56_F	0.0007	0.0010	0.0016	0.0000	0.0017	0.0000	0.0065	0.0000	0.0000	0.0045	0.0000	0.0042
PURA_P928_R	0.0000	0.0000	0.0012	0.0029	0.0000	0.0000	0.0000	0.0000	0.0000	0.0052	0.0010	0.0030
PWCR1_P811_F	0.3487	0.5643	0.9849	0.9774	0.0000	0.9759	0.0000	0.7197	0.7549	0.9820	0.9868	0.9888
PYCARD_E87_F	0.0191	0.1377	0.0107	0.0145	0.0101	0.0140	0.0645	0.0173	0.0135	0.0690	0.0259	0.0607
PYCARD_P150_F	0.0111	0.8167	0.0126	0.3971	0.4825	0.5193	0.0158	0.0066	0.0177	0.0134	0.0150	0.0171
RAB32_E314_R	0.0000	0.0052	0.0045	0.0955	0.0000	0.0216	0.0000	0.0060	0.0020	0.0059	0.0088	0.0049
RAB32_P493_R	0.0000	0.0000	0.0000	0.1280	0.0033	0.1677	0.0000	0.0000	0.0000	0.0000	0.0000	0.0000
RAF1_P330_F	0.0000	0.9873	0.0017	0.0010	0.0052	0.0000	0.0027	0.0004	0.0000	0.0017	0.0323	0.0004
RAN_P581_R	0.0008	0.0000	0.1767	0.5429	0.0000	0.6439	0.0019	0.0037	0.0000	0.0049	0.0054	0.7766
RARA_E128_R	0.5246	0.0055	0.0057	0.2386	0.0082	0.0841	0.0090	0.0060	0.0032	0.0047	0.0106	0.0020
RARA_P176_R	0.0025	0.0000	0.0013	0.2714	0.0000	0.6898	0.0000	0.0000	0.0000	0.0000	0.0000	0.0000
RARB_E114_F	0.0039	0.0031	0.0016	0.0042	0.0056	0.0119	0.0000	0.0039	0.0000	0.0059	0.0110	0.0038
RARB_P60_F	0.0098	0.0000	0.0000	0.0036	0.0036	0.0000	0.0000	0.0014	0.0000	0.0010	0.0120	0.0000
RARRES1_E235_F	0.0087	0.0171	0.0066	0.0593	0.0112	0.0074	0.0621	0.0108	0.0053	0.0113	0.0111	0.1092
RASGRF1_E16_F	0.0043	0.0030	0.0075	0.0074	0.0067	0.0049	0.0000	0.0050	0.0060	0.0117	0.0089	0.0003
RASGRF1_P768_F	0.0071	0.4108	0.0047	0.0037	0.1019	0.0022	0.4393	0.0066	0.0039	0.0000	0.0096	0.0846
RASSF1_E116_F	0.0000	0.0036	0.0054	0.0070	0.0004	0.0670	0.0000	0.0019	0.0693	0.0093	0.0100	0.0080
RASSF1_P244_F	0.0133	0.0063	0.0416	0.0073	0.0533	0.0050	0.3837	0.0059	0.0052	0.0143	0.0112	0.0058
RBL2_P250_R	0.0310	0.0000	0.0072	0.0024	0.0101	0.1224	0.0000	0.0072	0.0126	0.0043	0.0093	0.0053
RBP1_E158_F	0.0005	0.0000	0.0000	0.1281	0.0000	0.0844	0.0000	0.0000	0.0000	0.0000	0.0039	0.0019
RBP1_P150_F	0.0000	0.0000	0.1827	0.0000	0.0000	0.0000	0.0000	0.0000	0.0000	0.0000	0.0000	0.0000
RET_P717_F	0.0048	0.0040	0.0097	0.0088	0.0100	0.0043	0.0101	0.0138	0.0000	0.0109	0.0123	0.0046
RET_seq_53_S374_F	0.3879	0.0034	0.2282	0.0080	0.0053	0.0065	0.1354	0.0000	0.1239	0.1104	0.0089	0.0048

RHOC_P536_F	0.0000	0.0042	0.0001	0.0008	0.0000	0.0000	0.0000	0.0000	0.0000	0.0000	0.0000	0.0000
RHOH_P121_F	0.0000	0.0000	0.0000	0.0000	0.0000	0.0000	0.0000	0.0000	0.0000	0.0000	0.0000	0.0000
RHOH_P953_R	0.9799	0.9899	0.0000	0.8809	0.0000	0.9689	0.0000	0.0000	0.0000	0.9880	0.0000	0.0000
RIPK1_P868_F	0.0306	0.7979	0.9845	0.9746	0.4398	0.9747	0.9937	0.4411	0.9304	0.9845	0.9816	0.4541
RIPK2_E123_F	0.0033	0.0000	0.0040	0.0055	0.0087	0.0904	0.0066	0.0053	0.0007	0.0093	0.0521	0.0000
RIPK3_P124_F	0.0077	0.0066	0.3376	0.0088	0.0127	0.0069	0.0075	0.0098	0.4639	0.1037	0.5060	0.2055
RIPK3_P24_F	0.0000	0.0000	0.0000	0.0000	0.0034	0.0000	0.0000	0.0000	0.0000	0.0010	0.0000	0.0000
RIPK4_E166_F	0.0005	0.0000	0.0019	0.0000	0.0003	0.0599	0.0000	0.0000	0.0000	0.0000	0.0047	0.0000
RIPK4_P172_F	0.0040	0.0947	0.0072	0.0807	0.0116	0.0502	0.0129	0.0078	0.0034	0.0097	0.0094	0.2819
ROR1_P6_F	0.0000	0.0038	0.0057	0.0002	0.0000	0.0000	0.0000	0.0047	0.0000	0.0008	0.0118	0.0000
ROR2_P317_R	0.0000	0.0000	0.0002	0.0016	0.0031	0.0336	0.0000	0.0000	0.0000	0.0000	0.0000	0.0000
RRAS_P100_R	0.0000	0.0000	0.0000	0.0000	0.0005	0.0000	0.0000	0.0000	0.0000	0.0000	0.0000	0.0000
RUNX3_P247_F	0.9161	0.0000	0.9403	0.6537	0.8772	0.8050	0.7174	0.8208	0.0016	0.8156	0.9739	0.0000
RYK_P493_F	0.0000	0.0000	0.0383	0.0000	0.0464	0.0000	0.0019	0.0000	0.0000	0.0000	0.0000	0.0000
S100A2_E36_R	0.0011	0.0000	0.4085	0.2113	0.0004	0.4121	0.0000	0.0103	0.0303	0.0097	0.9766	0.0071
S100A4_E315_F	0.0079	0.0158	0.0115	0.0104	0.0104	0.0795	0.0145	0.0099	0.0042	0.0098	0.0237	0.1780
SCGB3A1_E55_R	0.0000	0.0000	0.0000	0.0048	0.0000	0.0000	0.0000	0.0000	0.0000	0.0000	0.0000	0.0000
SCGB3A1_P103_R	0.0000	0.0000	0.0000	0.0000	0.0000	0.0000	0.0000	0.0000	0.0000	0.0000	0.0000	0.0000
SEMA3A_P343_F	0.0000	0.0000	0.0000	0.0000	0.0000	0.2968	0.0037	0.0000	0.0000	0.0000	0.0041	0.0000
SEMA3A_P658_R	0.1010	0.0303	0.6663	0.2793	0.0582	0.3594	0.0828	0.0398	0.0443	0.0274	0.0376	0.0118
SEMA3C_E49_R	0.0145	0.0258	0.0094	0.0149	0.0154	0.0157	0.0417	0.4181	0.0110	0.0175	0.0175	0.1025
SEMA3C_P642_F	0.2230	0.0000	0.2068	0.5968	0.1554	0.0552	0.2420	0.0094	0.0259	0.0173	0.0172	0.2200
SEMA3F_E333_R	0.0026	0.0097	0.0029	0.0069	0.0000	0.1506	0.0000	0.0000	0.0000	0.0018	0.0155	0.1973
SEMA3F_P692_R	0.0017	0.0016	0.6848	0.0044	0.0000	0.0012	0.0000	0.0021	0.8989	0.0047	0.0054	0.0000
SEPT5_P441_F	0.9835	0.9891	0.0120	0.4129	0.0075	0.4051	0.0073	0.0642	0.1106	0.0277	0.0113	0.0030
SEPT9_P58_R	0.0113	0.0096	0.0237	0.6258	0.5369	0.8306	0.1298	0.9878	0.9849	0.9887	0.0359	0.0146
SERPINA5_E69_F	0.9829	0.9850	0.9597	0.5388	0.7163	0.7637	0.9944	0.1081	0.0086	0.9764	0.5011	0.9895
SERPINB2_P939_F	0.9847	0.9939	0.9746	0.9713	0.0000	0.9799	0.0000	0.9882	0.9887	0.9881	0.9865	0.9886
SERPINE1_E189_R	0.2775	0.0127	0.0071	0.2793	0.0129	0.2065	0.0124	0.0129	0.0082	0.0176	0.3681	0.0086
SEZ6L_P249_F	0.0065	0.2965	0.0021	0.0036	0.0101	0.0670	0.0092	0.0051	0.0012	0.0043	0.0078	0.0035
SEZ6L_P299_F	0.0000	0.0000	0.0054	0.0021	0.0036	0.0023	0.0069	0.0000	0.0008	0.0079	0.0108	0.0000
SFN_E118_F	0.9888	0.8697	0.5816	0.9348	0.8004	0.9710	0.9901	0.9915	0.9710	0.6033	0.9888	0.0198
SFRP1_E398_R	0.0000	0.0030	0.5183	0.0031	0.0033	0.0024	0.0000	0.0033	0.0000	0.0000	0.0064	0.0000

SFRP1_P157_F	0.0044	0.0047	0.0042	0.0032	0.0095	0.0024	0.0000	0.0048	0.0015	0.9758	0.0053	0.0029
SFTPA1_E340_R	0.0000	0.9870	0.9794	0.9822	0.9044	0.9788	0.0000	0.9841	0.9852	0.9891	0.9872	0.9887
SH3BP2_P771_R	0.0037	0.0000	0.8487	0.0977	0.0030	0.1307	0.0000	0.0000	0.0000	0.0000	0.0118	0.0000
SHB_P473_R	0.0056	0.0000	0.0033	0.0039	0.0080	0.0025	0.1727	0.0000	0.0000	0.0039	0.0045	0.0019
SHB_P691_R	0.0107	0.0119	0.0122	0.0117	0.0202	0.0118	0.3474	0.0102	0.0258	0.0186	0.0210	0.4037
SHH_E328_F	0.0000	0.0000	0.0000	0.0000	0.0000	0.0000	0.0000	0.0000	0.0000	0.9595	0.0000	0.0000
SHH_P104_R	0.0000	0.0050	0.0064	0.0099	0.0177	0.0068	0.0000	0.0000	0.4746	0.0272	0.4305	0.0080
SIN3B_P514_R	0.9462	0.9847	0.9419	0.9682	0.9816	0.9622	0.9912	0.9641	0.9777	0.9847	0.8955	0.6387
SKI_E465_R	0.0047	0.0074	0.0040	0.0078	0.0000	0.0065	0.0000	0.0072	0.0026	0.0044	0.0080	0.0029
SLC22A2_E271_R	0.8782	0.9882	0.9815	0.9815	0.9872	0.9846	0.0128	0.9911	0.9855	0.3544	0.9540	0.9932
SLC22A3_P634_F	0.6697	0.0316	0.2312	0.3566	0.4646	0.2465	0.0000	0.0175	0.0243	0.9866	0.9275	0.3346
SLC6A8_seq_28_S227_F	0.0000	0.0000	0.0000	0.6142	0.0000	0.6864	0.0000	0.0000	0.0000	0.0052	0.0016	0.9892
SLIT2_P208_F	0.0089	0.0132	0.1506	0.0084	0.1084	0.0084	0.2288	0.0077	0.0054	0.0053	0.0110	0.0044
SMAD2_P708_R	0.0000	0.0000	0.0000	0.0033	0.0077	0.0000	0.0000	0.0027	0.0004	0.0031	0.0040	0.0054
SMAD2_P848_R	0.0000	0.0000	0.0000	0.0021	0.0000	0.2762	0.0000	0.0015	0.0000	0.0067	0.0000	0.0025
SMAD4_P474_R	0.0000	0.0000	0.0000	0.1750	0.0000	0.0000	0.0000	0.0000	0.0000	0.0021	0.0030	0.0006
SMARCA3_E20_F	0.0000	0.0019	0.0012	0.1274	0.6165	0.0012	0.0000	0.0000	0.0000	0.0088	0.0056	0.0000
SMARCA3_P109_R	0.0143	0.0057	0.0080	0.2807	0.0164	0.0189	0.2319	0.0155	0.0131	0.0133	0.0149	0.0000
SMARCA3_P17_R	0.0000	0.0000	0.0020	0.0054	0.0107	0.0071	0.0060	0.0000	0.0000	0.0000	0.0001	0.0000
SMARCA4_P362_R	0.0054	0.0061	0.0050	0.0063	0.0079	0.0058	0.0070	0.0045	0.0041	0.0081	0.0222	0.0062
SMO_E57_F	0.0026	0.0000	0.0000	0.0080	0.0031	0.0000	0.0000	0.0138	0.0000	0.0068	0.0225	0.0000
SOD3_P225_F	0.9850	0.0617	0.1073	0.9738	0.9847	0.9703	0.2030	0.0000	0.0044	0.0536	0.9878	0.1839
SOX1_P1018_R	0.1487	0.0124	0.0557	0.0045	0.0062	0.0055	0.0010	0.0000	0.0000	0.0000	0.0020	0.0000
SOX1_P294_F	0.0140	0.9888	0.0120	0.2571	0.0249	0.1052	0.0095	0.0160	0.0128	0.0176	0.0218	0.4060
SOX17_P287_R	0.0000	0.0000	0.0000	0.0000	0.0000	0.0709	0.0000	0.0000	0.0000	0.0005	0.8197	0.0000
SOX17_P303_F	0.0987	0.1973	0.1281	0.0891	0.6944	0.1175	0.8211	0.0901	0.1263	0.8691	0.1192	0.1514
SOX2_P546_F	0.0084	0.0000	0.0070	0.1599	0.0086	0.0082	0.0216	0.0097	0.0080	0.0082	0.0136	0.0000
SPARC_E50_R	0.0083	0.0070	0.3602	0.1345	0.0133	0.1582	0.0118	0.0078	0.2534	0.0115	0.0150	0.0000
SPARC_P195_F	0.0019	0.0000	0.0055	0.3214	0.0064	0.0376	0.0116	0.0061	0.0016	0.7777	0.0103	0.0030
SPDEF_E116_R	0.3853	0.2759	0.0205	0.0288	0.0247	0.0562	0.0135	0.4884	0.1433	0.0699	0.2332	0.1742
SPI1_E205_F	0.9845	0.7588	0.0200	0.0192	0.0190	0.3510	0.7731	0.0456	0.0115	0.0268	0.0236	0.0106
SPP1_E140_R	0.0000	0.4135	0.6456	0.1484	0.0037	0.0015	0.0146	0.0078	0.0018	0.0066	0.0073	0.0000
SRC_P297_F	0.9858	0.9918	0.9860	0.9770	0.9432	0.9805	0.9937	0.9887	0.9904	0.9887	0.9811	0.0086

ST6GAL1_P164_R	0.0153	0.0126	0.0077	0.0659	0.1645	0.0124	0.0000	0.0272	0.1078	0.0000	0.0439	0.0000
ST6GAL1_P528_F	0.0022	0.0000	0.0020	0.4608	0.0110	0.1430	0.0052	0.0005	0.0020	0.0072	0.0109	0.0000
STK23_E182_R	0.0107	0.0000	0.0000	0.9020	0.0172	0.9791	0.0166	0.0003	0.0000	0.0110	0.0057	0.0000
SYK_E372_F	0.0300	0.0153	0.0114	0.0162	0.0161	0.0085	0.2150	0.0115	0.0129	0.0149	0.0277	0.0059
TAL1_E122_F	0.0099	0.0143	0.0242	0.0087	0.0080	0.0042	0.0131	0.0102	0.0098	0.0115	0.0139	0.2278
TAL1_P594_F	0.0000	0.0000	0.0000	0.0060	0.0068	0.0016	0.1995	0.0019	0.0000	0.0073	0.0056	0.0032
TAL1_P817_F	0.0000	0.0000	0.0000	0.0000	0.9241	0.7875	0.0000	0.0000	0.0000	0.9753	0.0000	0.0000
TCF4_P175_R	0.0000	0.0000	0.0000	0.0000	0.0000	0.0000	0.0000	0.0000	0.0000	0.0000	0.0037	0.0016
TCF7L2_E411_F	0.0013	0.0016	0.0000	0.1186	0.0019	0.0000	0.0011	0.0035	0.0000	0.0006	0.0097	0.0020
TCF7L2_P193_R	0.0098	0.0000	0.0000	0.0029	0.0066	0.0025	0.0091	0.0000	0.0000	0.0080	0.0072	0.0047
TERT_E20_F	0.0086	0.0107	0.0072	0.0869	0.0133	0.0333	0.0111	0.0110	0.0049	0.0099	0.0163	0.0074
TERT_P360_R	0.0210	0.3833	0.0103	0.1271	0.0169	0.0168	0.0220	0.0165	0.8366	0.0171	0.0119	0.0112
TES_E172_F	0.0002	0.0024	0.9274	0.0068	0.0039	0.0003	0.0090	0.0053	0.0034	0.0075	0.0086	0.2023
TESK2_P252_R	0.0079	0.0072	0.0045	0.0064	0.0113	0.0067	0.0068	0.0503	0.0044	0.0333	0.0079	0.0372
TFAP2C_E260_F	0.0018	0.0000	0.0006	0.0012	0.0135	0.0000	0.0000	0.0046	0.0000	0.0000	0.0066	0.0000
TFAP2C_P765_F	0.0046	0.0000	0.0000	0.0095	0.0081	0.0061	0.0067	0.0000	0.0042	0.0104	0.0251	0.0056
TFF2_P178_F	0.9846	0.8755	0.9817	0.9628	0.6350	0.9643	0.8457	0.9533	0.9900	0.8758	0.1391	0.8083
TFF2_P557_R	0.9680	0.3525	0.9516	0.9856	0.9897	0.9784	0.9950	0.9898	0.9537	0.9815	0.8036	0.0000
TFPI2_E141_F	0.0000	0.0000	0.0000	0.0000	0.0000	0.0000	0.0040	0.0024	0.0000	0.0013	0.0000	0.0000
TFPI2_P152_R	0.0108	0.2583	0.0153	0.2409	0.0182	0.0157	0.1065	0.0071	0.3900	0.0136	0.0335	0.2232
TFPI2_P9_F	0.0147	0.6350	0.0160	0.0144	0.0153	0.0107	0.0171	0.0121	0.0095	0.0176	0.0156	0.5080
TFRC_P414_R	0.0000	0.0032	0.2151	0.0103	0.0000	0.0082	0.6038	0.0000	0.0147	0.0244	0.1910	0.0012
TGFA_P558_F	0.0083	0.0000	0.0143	0.0106	0.0092	0.0076	0.0070	0.0087	0.3623	0.0114	0.0135	0.0095
TGFA_P642_R	0.0032	0.0000	0.0064	0.0044	0.0067	0.0034	0.0070	0.0000	0.0022	0.0000	0.0091	0.0000
TGFB1_P833_R	0.9844	0.0000	0.9836	0.9562	0.7459	0.9786	0.0060	0.9833	0.0000	0.0000	0.0061	0.7518
TGFB2_E226_R	0.0000	0.0000	0.0000	0.0000	0.0000	0.0000	0.0000	0.0000	0.0000	0.0000	0.0009	0.0021
TGFB2_P632_F	0.0124	0.0000	0.0099	0.0151	0.0223	0.0110	0.2142	0.0134	0.0162	0.0103	0.0211	0.1670
TGFB3_E58_R	0.4871	0.0000	0.7791	0.9719	0.0000	0.9846	0.0000	0.8580	0.9854	0.9887	0.6392	0.0044
TGFBI_P173_F	0.0132	0.3385	0.1517	0.0154	0.0867	0.2345	0.0000	0.0456	0.0061	0.0609	0.0620	0.1443
TGFBI_P31_R	0.0069	0.0048	0.4143	0.1386	0.0100	0.1555	0.0109	0.0065	0.0034	0.5588	0.0983	0.0000
TGFBR3_E188_R	0.0001	0.0000	0.0140	0.0043	0.0099	0.0017	0.0072	0.0047	0.0000	0.0029	0.0056	0.0000
TGFBR3_P429_F	0.0016	0.0000	0.0008	0.0011	0.0040	0.0000	0.0023	0.0000	0.0000	0.0010	0.0000	0.0000
THBS1_E207_R	0.0063	0.0000	0.0040	0.0048	0.0039	0.0025	0.0084	0.0033	0.0038	0.0000	0.0067	0.0389

THBS1_P500_F	0.0167	0.0126	0.0438	0.1219	0.0202	0.0102	0.0057	0.0171	0.0131	0.0119	0.5076	0.0075
THBS2_E129_F	0.0105	0.0051	0.0009	0.0036	0.0042	0.0035	0.0000	0.0043	0.0016	0.0119	0.0092	0.0000
THY1_P149_R	0.0269	0.1689	0.1585	0.2342	0.0173	0.0236	0.0250	0.0190	0.0820	0.0169	0.0249	0.3021
TIAM1_P188_R	0.0000	0.0000	0.0000	0.0019	0.0047	0.1517	0.0000	0.0000	0.0000	0.0018	0.0003	0.9910
TIMP1_E254_R	0.0094	0.0602	0.0531	0.5520	0.0236	0.1967	0.0403	0.0267	0.0132	0.0082	0.0081	0.0693
TIMP1_P615_R	0.0000	0.0000	0.0000	0.9598	0.9780	0.9001	0.0000	0.0000	0.0000	0.0000	0.9731	0.0000
TIMP2_E394_R	0.0000	0.0000	0.0050	0.0072	0.0133	0.0045	0.0077	0.0050	0.0000	0.0063	0.0000	0.0909
TIMP2_P267_F	0.8816	0.0029	0.2147	0.0012	0.0014	0.0005	0.0084	0.0046	0.0000	0.0031	0.4022	0.0015
TIMP3_P1114_R	0.9901	0.9900	0.9888	0.9607	0.2223	0.9845	0.9943	0.9938	0.9902	0.9838	0.9731	0.9923
TIMP3_P690_R	0.9549	0.9898	0.9883	0.9879	0.9871	0.9887	0.0000	0.9692	0.9910	0.9645	0.9908	0.0000
TIMP3_seq_7_S38_F	0.0048	0.0586	0.0073	0.0073	0.0116	0.0043	0.0782	0.0153	0.0166	0.0059	0.0069	0.0023
TJP1_P326_R	0.0416	0.1141	0.1147	0.0177	0.0542	0.0111	0.1197	0.0180	0.0197	0.0165	0.0164	0.0702
TJP1_P390_F	0.0094	0.0000	0.0086	0.0078	0.0766	0.0051	0.0069	0.0057	0.0055	0.0000	0.0127	0.1558
TK1_E47_F	0.1250	0.2638	0.0083	0.0134	0.0481	0.0161	0.0512	0.3462	0.0384	0.2727	0.0372	0.1974
TK1_P62_R	0.0088	0.0129	0.0059	0.0076	0.0084	0.0045	0.0124	0.0070	0.0050	0.0111	0.0151	0.1903
TMEFF1_E180_R	0.0061	0.0000	0.0081	0.0071	0.0085	0.0044	0.0000	0.0000	0.0033	0.0094	0.0088	0.0057
TMEFF1_P234_F	0.0011	0.0000	0.0059	0.1547	0.0000	0.1258	0.0052	0.0040	0.0000	0.0000	0.0038	0.0000
TMEFF2_P152_R	0.3843	0.4232	0.0632	0.2324	0.3790	0.0697	0.3424	0.0590	0.0777	0.0741	0.1811	0.2659
TMEFF2_P210_R	0.0000	0.0000	0.0058	0.0000	0.0000	0.0004	0.0078	0.0000	0.0000	0.0007	0.0000	0.0051
TMEM63A_E63_F	0.0000	0.0000	0.0000	0.0000	0.0051	0.0000	0.0000	0.0065	0.0000	0.0007	0.0029	0.0000
TMPRSS4_E83_F	0.8758	0.9879	0.9859	0.9855	0.9860	0.9773	0.9906	0.9892	0.9852	0.9757	0.9078	0.0000
TMPRSS4_P552_F	0.8954	0.4149	0.9778	0.7676	0.9774	0.8887	0.0541	0.0428	0.0284	0.9115	0.9848	0.9917
TNC_P198_F	0.0000	0.0031	0.0000	0.0827	0.0000	0.0000	0.0000	0.0074	0.0000	0.0000	0.0039	0.0000
TNC_P57_F	0.0063	0.0092	0.0075	0.4231	0.0100	0.0038	0.0142	0.0081	0.0026	0.0096	0.0097	0.0071
TNF_P1084_F	0.9883	0.9923	0.9833	0.9747	0.9940	0.9888	0.9952	0.9905	0.9918	0.9900	0.9913	0.0030
TNF_P158_F	0.0000	0.0000	0.0054	0.0007	0.0000	0.0000	0.0000	0.0000	0.0000	0.0013	0.0003	0.0665
TNFRSF10A_P171_F	0.0068	0.0095	0.0077	0.0102	0.0128	0.0094	0.0118	0.0221	0.0079	0.0160	0.0162	0.1390
TNFRSF10A_P91_F	0.0024	0.0128	0.0055	0.0016	0.0071	0.0000	0.0000	0.0088	0.0000	0.0000	0.0085	0.0000
TNFRSF10B_P108_R	0.0155	0.1050	0.0129	0.0252	0.0168	0.0479	0.0684	0.0260	0.1010	0.0858	0.0323	0.0129
TNFRSF10C_E109_F	0.0038	0.0077	0.0093	0.0037	0.0077	0.0037	0.0000	0.0052	0.0031	0.0052	0.0092	0.2597
TNFRSF10D_E27_F	0.0030	0.0076	0.0036	0.0040	0.0020	0.0030	0.0000	0.0074	0.0035	0.0098	0.0069	0.0041
TNFRSF10D_P70_F	0.0159	0.0150	0.0207	0.0172	0.0149	0.0206	0.0398	0.1328	0.0284	0.0132	0.0291	0.0065
TNFRSF1B_E5_F	0.0087	0.0000	0.0000	0.0121	0.0152	0.0100	0.0000	0.0061	0.0039	0.0089	0.0139	0.0000

TNFRSF1B_P167_F	0.0032	0.0000	0.0003	0.0016	0.0068	0.0849	0.0039	0.0000	0.0000	0.0038	0.0085	0.0000
TNFSF10_E53_F	0.0241	0.0159	0.0114	0.0177	0.0154	0.1076	0.2267	0.0099	0.0000	0.0160	0.0150	0.3176
TNFSF10_P2_R	0.0000	0.0000	0.0000	0.0000	0.0000	0.0000	0.0000	0.0000	0.0000	0.0000	0.0000	0.9867
TNFSF8_E258_R	0.0221	0.0000	0.0000	0.0067	0.0000	0.0032	0.0000	0.0008	0.0000	0.0115	0.0099	0.0021
TNFSF8_P184_F	0.0037	0.0040	0.0006	0.0028	0.0063	0.0015	0.0068	0.0000	0.0013	0.0037	0.0065	0.0037
TNK1_P41_R	0.0167	0.0105	0.0234	0.0157	0.0358	0.2870	0.0476	0.0327	0.0238	0.0178	0.0245	0.0159
TP73_E155_F	0.0255	0.5007	0.0086	0.0092	0.0084	0.0071	0.0127	0.3235	0.0064	0.4094	0.0163	0.6357
TP73_P496_F	0.0000	0.0000	0.0037	0.2215	0.0033	0.0251	0.0111	0.0066	0.0000	0.0051	0.0050	0.9902
TP73_P945_F	0.0077	0.0086	0.0055	0.3676	0.0487	0.0659	0.0000	0.0000	0.0078	0.0083	0.0101	0.0165
TPEF_seq_44_S36_F	0.0052	0.0000	0.0095	0.0061	0.0047	0.0044	0.0000	0.0078	0.0000	0.7724	0.0125	0.0000
TPEF_seq_44_S88_R	0.0105	0.0534	0.0089	0.0112	0.0127	0.0130	0.0083	0.0610	0.0098	0.0088	0.0116	0.0169
TRIM29_P261_F	0.9858	0.8500	0.9878	0.9777	0.9804	0.9786	0.9917	0.0394	0.9837	0.6978	0.9867	0.7645
TRIP6_E33_F	0.0116	0.0131	0.0065	0.0707	0.2082	0.0082	0.1247	0.0069	0.0088	0.0091	0.3714	0.0072
TSG101_P257_R	0.0020	0.0000	0.0000	0.0001	0.0043	0.0198	0.0000	0.0043	0.6138	0.0000	0.0068	0.0000
TUBB3_E91_F	0.0023	0.0024	0.4935	0.0028	0.0032	0.2208	0.0000	0.0030	0.0000	0.0044	0.0053	0.0000
TUBB3_P364_F	0.0000	0.0000	0.0000	0.0000	0.0000	0.0000	0.0000	0.0000	0.0000	0.0011	0.0000	0.0000
TUBB3_P721_R	0.0016	0.0000	0.0000	0.0047	0.0060	0.0000	0.0000	0.0038	0.0000	0.0043	0.0092	0.0000
TUSC3_E29_R	0.0099	0.0075	0.0057	0.1553	0.0088	0.0054	0.0086	0.0000	0.0000	0.0110	0.0126	0.0000
TUSC3_P85_R	0.0794	0.0090	0.0048	0.1060	0.0048	0.0790	0.0114	0.0073	0.0031	0.0131	0.0172	0.0000
TWIST1_E117_R	0.0000	0.0000	0.0000	0.0000	0.0000	0.0000	0.0000	0.0000	0.0000	0.0000	0.0000	0.0000
TWIST1_P355_R	0.0000	0.0000	0.0031	0.2045	0.0066	0.0046	0.0036	0.0048	0.0062	0.0021	0.0082	0.0000
TWIST1_P44_R	0.0000	0.0000	0.0000	0.0000	0.0000	0.0000	0.0000	0.0000	0.0000	0.0000	0.0000	0.0000
TYRO3_P501_F	0.0000	0.0000	0.0000	0.0000	0.0001	0.0000	0.0000	0.0013	0.0000	0.0000	0.0022	0.0000
UBA52_P293_R	0.0000	0.0000	0.0000	0.0000	0.0000	0.0000	0.1050	0.0000	0.0000	0.0072	0.0034	0.0000
UGT1A1_E11_F	0.8306	0.9438	0.9905	0.9850	0.9832	0.9852	0.0000	0.9927	0.9906	0.9884	0.9915	0.9917
UGT1A1_P564_R	0.9634	0.9385	0.8955	0.9327	0.7379	0.9503	0.9598	0.5854	0.8273	0.8516	0.8598	0.9559
UGT1A7_P751_R	0.9656	0.9905	0.9903	0.9839	0.7581	0.9837	0.0000	0.9848	0.9054	0.9903	0.9897	0.9895
UNG_P170_F	0.0119	0.0000	0.0137	0.0058	0.0061	0.1434	0.0000	0.0000	0.0017	0.0081	0.0062	0.0059
USP29_E274_F	0.9920	0.9923	0.9761	0.9309	0.8715	0.9634	0.0000	0.9920	0.9898	0.9897	0.8828	0.9905
USP29_P282_R	0.9840	0.9908	0.9843	0.9818	0.0000	0.9758	0.7066	0.9910	0.9875	0.0000	0.9895	0.4405
VAMP8_P114_F	0.0161	0.1160	0.0139	0.0105	0.0162	0.0154	0.0125	0.0154	0.0111	0.0086	0.0141	0.1734
VAV1_E9_F	0.0739	0.0000	0.0066	0.0127	0.0082	0.0100	0.0069	0.0078	0.0087	0.0084	0.0114	0.0000
VAV1_P317_F	0.0021	0.0050	0.0000	0.0048	0.0037	0.0000	0.0000	0.0035	0.0007	0.0040	0.0079	0.0000

VAV2_E58_F	0.0176	0.1986	0.0212	0.0206	0.1808	0.0146	0.1335	0.1725	0.2158	0.2049	0.0157	0.2062
VAV2_P1182_F	0.0000	0.0000	0.0000	0.0000	0.0021	0.0000	0.0024	0.0000	0.0000	0.0000	0.0000	0.0000
VBP1_P12_R	0.0000	0.0000	0.0010	0.5147	0.0089	0.3503	0.0000	0.0000	0.0000	0.0037	0.0015	0.0000
VEGFB_P658_F	0.0052	0.0072	0.0144	0.0064	0.0073	0.1430	0.0116	0.0060	0.0033	0.0074	0.0095	0.0114
VIM_P811_R	0.0005	0.0000	0.0000	0.0000	0.0034	0.0000	0.0059	0.0008	0.0000	0.0000	0.0055	0.0000
WEE1_P924_R	0.8405	0.0000	0.9606	0.8858	0.9901	0.9846	0.9945	0.9890	0.0000	0.9881	0.9895	0.0000
WNT2_E109_R	0.0000	0.0000	0.0000	0.0000	0.0000	0.0000	0.0000	0.0000	0.0000	0.0000	0.0000	0.0000
WNT2_P217_F	0.0471	0.2638	0.0335	0.0371	0.0407	0.0405	0.1870	0.0252	0.0291	0.0251	0.0319	0.2203
WNT2B_P1185_R	0.0801	0.0023	0.0054	0.3029	0.0257	0.0124	0.0385	0.0092	0.0148	0.0251	0.0153	0.0543
WNT5A_E43_F	0.0217	0.0314	0.0109	0.0190	0.0152	0.0706	0.0146	0.5313	0.5275	0.0118	0.0633	0.0295
WNT5A_P655_F	0.0000	0.0000	0.0067	0.0042	0.0093	0.0046	0.2398	0.0000	0.0060	0.0076	0.0027	0.1402
WNT8B_E487_F	0.9814	0.7363	0.9747	0.9470	0.9903	0.9672	0.3223	0.2895	0.1911	0.3676	0.9839	0.0196
WNT8B_P216_R	0.0000	0.0000	0.9604	0.9769	0.9882	0.9706	0.9939	0.0000	0.0000	0.9867	0.9794	0.0000
WRN_E57_F	0.7112	0.4181	0.0299	0.0414	0.0231	0.0490	0.1710	0.0121	0.0448	0.4148	0.0256	0.2339
WRN_P969_F	0.9828	0.9886	0.0001	0.9501	0.7654	0.9772	0.0000	0.0000	0.0000	0.9872	0.8658	0.0000
WT1_E32_F	0.0045	0.0053	0.0035	0.0102	0.0382	0.1204	0.0198	0.0000	0.0007	0.0153	0.0000	0.0000
WT1_P853_F	0.0029	0.0015	0.0019	0.0044	0.0000	0.2061	0.0000	0.0014	0.0000	0.0060	0.0087	0.0240
XRCC1_P681_R	0.8248	0.0000	0.8981	0.9793	0.0000	0.9816	0.0000	0.9898	0.9904	0.0000	0.9549	0.9900
XRCC2_P1077_F	0.9894	0.9941	0.9873	0.9872	0.9872	0.9875	0.9942	0.9881	0.9837	0.9389	0.9859	0.9902
YES1_P600_F	0.0095	0.1592	0.0139	0.0124	0.0181	0.0132	0.0808	0.0136	0.0074	0.0122	0.0172	0.2027
ZIM3_E203_F	0.9880	0.9930	0.9883	0.9836	0.9741	0.9806	0.9901	0.0000	0.9898	0.9867	0.9866	0.9912
ZIM3_P451_R	0.9879	0.9936	0.9873	0.9823	0.0217	0.9836	0.9935	0.9912	0.9916	0.9835	0.9890	0.9904
ZIM3_P718_R	0.9036	0.9858	0.9334	0.9811	0.0000	0.9851	0.0000	0.9903	0.9880	0.0000	0.9871	0.0000
ZMYND10_E77_R	0.0006	0.0000	0.0000	0.0000	0.0080	0.0000	0.0000	0.0031	0.0000	0.0000	0.0007	0.0000
ZNF215_P71_R	0.0059	0.0076	0.0038	0.0077	0.7072	0.0051	0.0000	0.0102	0.0031	0.0116	0.0130	0.0057
ZNF264_E48_R	0.0000	0.0002	0.0000	0.0000	0.0000	0.0000	0.0000	0.0000	0.0000	0.0005	0.0018	0.0005
ZNFN1A1_E102_F	0.9872	0.5194	0.5090	0.9286	0.2918	0.9728	0.0000	0.9791	0.9777	0.9838	0.6441	0.9862
ZNFN1A1_P179_F	0.0763	0.9896	0.9813	0.9831	0.9905	0.9806	0.0000	0.9892	0.9911	0.8565	0.2437	0.9934

CONTROL_7	CONTROL_8	TargetID	CONTROL_9	CONTROL_10	CONTROL_11	CONTROL_12	CONTROL_13	CONTROL_14
0.9884	0.9884	AATK_E63_R	0.9787	0.9836	0.9835	0.9902	0.0000	0.8770
0.9832	0.9944	ABCA1_E120_R	0.9860	0.9833	0.9853	0.9901	0.0000	0.9858
0.9737	0.9446	ABCA1_P45_F	0.9815	0.9849	0.9793	0.0000	0.9892	0.9820
0.0045	0.0725	ABCB4_E429_F	0.0032	0.0069	0.0000	0.0191	0.1130	0.0051
0.9818	0.1543	ABCC2_P88_F	0.0168	0.3889	0.0053	0.0901	0.0262	0.0194
0.0096	0.0063	ABCC5_P444_F	0.2352	0.0117	0.0627	0.0050	0.0000	0.0063
0.0273	0.4622	ABCG2_P178_R	0.0317	0.0369	0.0102	0.4704	0.6365	0.0481
0.9816	0.9837	ABCG2_P310_R	0.9787	0.9830	0.9801	0.9883	0.9865	0.9814
0.9620	0.2525	ABL1_P53_F	0.9826	0.8949	0.9815	0.2811	0.8339	0.9825
0.0000	0.0041	ABL2_P459_R	0.0000	0.0000	0.0000	0.0000	0.0028	0.0000
0.0090	0.0038	ABO_E110_F	0.0093	0.2038	0.2460	0.0061	0.0062	0.0163
0.0112	0.0069	ABO_P312_F	0.0360	0.1819	0.0849	0.0069	0.1682	0.0169
0.0016	0.0011	ACTG2_P455_R	0.0032	0.0034	0.0000	0.0017	0.0064	0.0000
0.0060	0.0015	ACVR1_P983_F	0.0010	0.0041	0.0000	0.0000	0.0000	0.0000
0.0058	0.0727	ACVR1B_E497_R	0.4991	0.0066	0.0000	0.0112	0.0000	0.0044
0.0143	0.9912	ACVR1B_P572_R	0.0104	0.0148	0.0274	0.0142	0.0129	0.2966
0.0050	0.0047	ACVR1C_P115_R	0.0052	0.0062	0.0356	0.5985	0.0034	0.0007
0.9858	0.9933	ACVR1C_P363_F	0.9875	0.9871	0.9859	0.9931	0.9935	0.9834
0.9722	0.3279	ACVR2B_E27_R	0.9806	0.9788	0.9750	0.0143	0.8397	0.9750
0.0000	0.0000	ACVR2B_P676_F	0.0000	0.0000	0.0000	0.0000	0.0000	0.0000
0.0000	0.0000	ADAMTS12_P250_R	0.0000	0.0023	0.0000	0.0000	0.0000	0.0000
0.0038	0.0087	ADCYAP1_E163_R	0.0051	0.0093	0.0000	0.0006	0.0023	0.0091
0.8089	0.9914	ADCYAP1_P398_F	0.9824	0.9811	0.9783	0.9207	0.9248	0.9805
0.0123	0.0128	ADCYAP1_P455_R	0.0227	0.0210	0.0169	0.0137	0.5180	0.0206
0.1852	0.0032	AFF3_P122_F	0.0054	0.1199	0.1109	0.6774	0.0000	0.1781
0.0089	0.0055	AFF3_P808_F	0.4031	0.3354	0.0541	0.0000	0.0000	0.0028
0.0088	0.0081	AFP_P824_F	0.4445	0.2801	0.0678	0.1351	0.5917	0.4002
0.0000	0.0000	AGTR1_P154_F	0.0000	0.0000	0.0848	0.0000	0.0000	0.0000
0.0178	0.3178	AGTR1_P41_F	0.0123	0.0138	0.0141	0.5666	0.4411	0.0143
0.5370	0.9863	AHR_E103_F	0.9027	0.4410	0.9597	0.0000	0.0000	0.9788
0.7903	0.4705	AHR_P166_R	0.7914	0.8258	0.8381	0.7215	0.4914	0.8721

0.0103	0.1872	AIM2_E208_F	0.0126	0.1765	0.0035	0.1251	0.0000	0.0140
0.0930	0.0052	AKT1_P310_R	0.2252	0.0113	0.1781	0.0076	0.0052	0.0090
0.7422	0.2104	ALK_P28_F	0.1390	0.5021	0.4430	0.0626	0.0041	0.2199
0.0002	0.0025	ALOX12_E85_R	0.0001	0.0005	0.0000	0.0000	0.0015	0.0000
0.9823	0.5702	ALOX12_P223_R	0.9752	0.9771	0.9794	0.0094	0.6328	0.9770
0.9493	0.0000	APBA1_E99_R	0.9065	0.0000	0.9502	0.0000	0.0000	0.9360
0.0000	0.0000	APBA1_P644_F	0.8259	0.0000	0.5226	0.0000	0.0000	0.0000
0.0046	0.0000	APBA2_P227_F	0.0045	0.0037	0.0000	0.0000	0.0031	0.3353
0.9833	0.0000	APC_P280_R	0.9815	0.9811	0.9830	0.0000	0.0000	0.7264
0.0000	0.0104	APOA1_P75_F	0.0000	0.0000	0.0000	0.0106	0.0000	0.3651
0.0862	0.8619	APOC1_P406_R	0.3681	0.5974	0.3680	0.0062	0.0039	0.0368
0.0013	0.0000	APP_E8_F	0.0000	0.0026	0.0000	0.1638	0.0040	0.0007
0.0145	0.0184	APP_P179_R	0.0176	0.0213	0.0138	0.0160	0.5798	0.0226
0.0080	0.1962	AR_P189_R	0.2283	0.0094	0.3830	0.2899	0.0074	0.0093
0.0055	0.0000	AREG_E25_F	0.0014	0.0060	0.0000	0.0000	0.0023	0.0033
0.3524	0.0141	ARHGDIB_P148_R	0.2367	0.0391	0.1594	0.0177	0.0106	0.0333
0.0078	0.0015	ARNT_P238_R	0.0135	0.0067	0.0003	0.0835	0.0026	0.0087
0.0128	0.0270	ASB4_P391_F	0.0116	0.0071	0.0000	0.0038	0.1647	0.0290
0.0052	0.0000	ASB4_P52_R	0.0036	0.1794	0.0162	0.0053	0.0000	0.0042
0.0000	0.0000	ASCL1_E24_F	0.0000	0.0000	0.0000	0.0000	0.0000	0.0000
0.0073	0.0000	ASCL1_P747_F	0.0047	0.0074	0.0000	0.0038	0.1681	0.0049
0.0000	0.0000	ASCL2_E76_R	0.0000	0.0000	0.0000	0.0000	0.0000	0.0000
0.0000	0.0000	ASCL2_P360_F	0.1027	0.0000	0.2046	0.0000	0.0015	0.0000
0.7384	0.0000	ASCL2_P609_R	0.0000	0.0000	0.0000	0.0000	0.0000	0.0000
0.0059	0.0000	ATP10A_P147_F	0.0014	0.0151	0.0000	0.0000	0.0000	0.3798
0.0099	0.0000	ATP10A_P524_R	0.0095	0.0152	0.0000	0.0000	0.0112	0.0108
0.0131	0.0147	AXIN1_P995_R	0.0152	0.0164	0.0117	0.0180	0.0245	0.0201
0.0034	0.0011	AXL_E61_F	0.0039	0.0042	0.0000	0.0057	0.0069	0.0976
0.0134	0.4720	BAX_E281_R	0.0149	0.0226	0.1471	0.0239	0.0126	0.0169
0.1540	0.4328	BCAM_E100_R	0.0084	0.0123	0.0039	0.0758	0.0039	0.2174
0.0134	0.0062	BCAM_P205_F	0.0164	0.0209	0.1155	0.0177	0.3328	0.5054
0.0143	0.0152	BCAP31_P1131_F	0.0160	0.0145	0.0128	0.2068	0.6013	0.0135
0.0000	0.0000	BCL2L2_E172_F	0.0000	0.0000	0.0000	0.0000	0.0029	0.0000

0.0083	0.0037	BCL2L2_P280_F	0.0062	0.0101	0.0000	0.0064	0.0000	0.0040
0.0030	0.0015	BCL3_E71_F	0.0022	0.0493	0.0000	0.0039	0.0011	0.0022
0.0124	0.0908	BCL3_P1038_R	0.1071	0.2707	0.0492	0.0075	0.0000	0.0092
0.0059	0.0000	BCL6_P248_R	0.0039	0.0076	0.0000	0.0036	0.0000	0.0053
0.0446	0.0026	BDNF_E19_R	0.0288	0.0377	0.0179	0.1032	0.0031	0.0245
0.9861	0.9923	BDNF_P259_R	0.9859	0.9863	0.9861	0.9913	0.9926	0.9878
0.0051	0.9927	BGN_E282_R	0.0031	0.0059	0.0000	0.0049	0.0036	0.0052
0.0104	0.0067	BGN_P333_R	0.0016	0.0078	0.0260	0.0036	0.0000	0.0033
0.0136	0.3872	BIRC4_P122_R	0.0120	0.1585	0.0323	0.0094	0.3929	0.1392
0.0112	0.0701	BIRC5_E89_F	0.2670	0.0247	0.0695	0.0097	0.0088	0.0155
0.0123	0.0153	BLK_P14_F	0.0174	0.0115	0.0019	0.2593	0.0066	0.1781
0.0110	0.3152	BMP2_E48_R	0.0057	0.0110	0.0754	0.0130	0.0099	0.0100
0.0282	0.0000	BMP2_P1201_F	0.0034	0.0033	0.0103	0.0000	0.0000	0.0000
0.0000	0.0000	BMP3_E147_F	0.0000	0.0000	0.0000	0.0000	0.0000	0.0000
0.0066	0.0000	BMP3_P56_R	0.4039	0.0078	0.0000	0.0027	0.0000	0.0068
0.0249	0.5737	BMP4_P199_R	0.1756	0.5418	0.5093	0.5150	0.0077	0.5501
0.9844	0.9927	BMP6_P398_F	0.9856	0.9856	0.9838	0.0012	0.9934	0.9857
0.0099	0.5757	BMPR1A_P956_F	0.2464	0.0135	0.1622	0.0069	0.4384	0.0115
0.0000	0.0000	BMPR2_E435_F	0.0000	0.0000	0.0000	0.0000	0.0000	0.0000
0.0284	0.2048	BMPR2_P1271_F	0.1171	0.0375	0.0111	0.0040	0.0021	0.2021
0.1122	0.1078	BSG_P211_R	0.1300	0.0827	0.1046	0.0950	0.0738	0.0817
0.5400	0.0101	BTK_P105_F	0.0148	0.0188	0.0890	0.0101	0.0087	0.0147
0.0097	0.4814	C20orf47_P225_R	0.0007	0.0132	0.0000	0.0177	0.1413	0.0023
0.4133	0.1224	CALCA_E174_R	0.0596	0.0680	0.0349	0.5787	0.0605	0.0791
0.0043	0.0000	CAPG_E228_F	0.0704	0.0042	0.0900	0.8261	0.5882	0.0941
0.0140	0.0000	CASP10_E139_F	0.0039	0.0031	0.0000	0.2447	0.0031	0.0040
0.7308	0.1878	CASP10_P186_F	0.9698	0.9813	0.9782	0.0000	0.0000	0.9802
0.0289	0.0399	CASP2_P192_F	0.0330	0.0279	0.0222	0.0308	0.0285	0.0346
0.0084	0.0000	CASP3_P420_R	0.0027	0.0058	0.0511	0.0058	0.2302	0.0009
0.7276	0.0000	CASP6_P201_F	0.9039	0.9075	0.8225	0.0267	0.0000	0.9334
0.2853	0.0040	CASP6_P230_R	0.1167	0.0146	0.2311	0.0050	0.0000	0.1812
0.0000	0.1945	CAV1_P130_R	0.3693	0.0000	0.0948	0.0107	0.1911	0.0052
0.0477	0.0460	CAV1_P169_F	0.1337	0.0242	0.0358	0.0208	0.0100	0.0221

0.3354	0.5928	CAV2_E33_R	0.1005	0.0924	0.4430	0.9837	0.6867	0.3180
0.0152	0.0565	CCKBR_P361_R	0.0111	0.0194	0.0050	0.0044	0.4734	0.0163
0.0000	0.0020	CCKBR_P480_F	0.0000	0.0000	0.0000	0.0000	0.7415	0.0000
0.9813	0.0000	CCNA1_E7_F	0.9789	0.9828	0.9763	0.0000	0.0701	0.9813
0.0000	0.0000	CCNA1_P216_F	0.0000	0.0000	0.1177	0.0000	0.0017	0.0019
0.0000	0.0018	CCNC_P132_R	0.3235	0.5717	0.0693	0.0000	0.0045	0.3246
0.5834	0.0025	CCND1_E280_R	0.2153	0.2973	0.7044	0.0000	0.0048	0.2747
0.0000	0.0000	CCND1_P343_R	0.0000	0.0000	0.0000	0.0000	0.0000	0.0000
0.0000	0.0000	CCND2_P887_F	0.0000	0.0017	0.0000	0.0000	0.0000	0.0000
0.0844	0.4363	CCND2_P898_R	0.6110	0.8581	0.7000	0.0254	0.6204	0.8365
0.0000	0.0006	CCNE1_P683_F	0.0000	0.0000	0.0066	0.0000	0.0017	0.0000
0.0051	0.0067	CD1A_P414_R	0.0103	0.0135	0.0138	0.0051	0.0067	0.0101
0.7629	0.0097	CD2_P68_F	0.7387	0.5671	0.7971	0.0994	0.0051	0.1137
0.0301	0.0133	CD34_P339_R	0.0137	0.0201	0.0112	0.0082	0.6001	0.0226
0.5218	0.9872	CD34_P780_R	0.7631	0.8819	0.9269	0.6845	0.9684	0.9316
0.0062	0.0023	CD40_E58_R	0.0027	0.0011	0.0000	0.2258	0.0000	0.0025
0.0222	0.0170	CD40_P372_R	0.0204	0.0312	0.0516	0.0228	0.0069	0.0241
0.7849	0.0095	CD44_E26_F	0.1377	0.0055	0.0813	0.0024	0.0000	0.0000
0.9863	0.0000	CD44_P87_F	0.8833	0.0019	0.5712	0.0018	0.0039	0.1507
0.0104	0.0000	CD86_P3_F	0.0000	0.0000	0.0000	0.0000	0.0000	0.0000
0.0000	0.0000	CDC25B_E83_F	0.0000	0.0000	0.0000	0.0000	0.0000	0.0000
0.0383	0.0000	CDC25B_P11_R	0.0131	0.0090	0.0023	0.0039	0.0611	0.0151
0.0000	0.0006	CDH1_P52_R	0.0000	0.0196	0.0122	0.0000	0.0000	0.0000
0.0023	0.2200	CDH11_E102_R	0.0035	0.0041	0.0000	0.0064	0.0000	0.0048
0.0137	0.0090	CDH11_P203_R	0.0129	0.1660	0.1411	0.2508	0.4782	0.0154
0.0078	0.0044	CDH11_P354_R	0.0065	0.0075	0.0000	0.7696	0.0018	0.0067
0.9133	0.0045	CDH13_E102_F	0.9863	0.9837	0.9839	0.9923	0.9925	0.9818
0.0074	0.0000	CDH17_E31_F	0.0111	0.0140	0.0996	0.0090	0.0067	0.0132
0.1084	0.5892	CDH17_P532_F	0.9755	0.9817	0.9752	0.5456	0.9906	0.9789
0.0208	0.1903	CDH3_E100_R	0.0248	0.0334	0.0300	0.1943	0.1888	0.0263
0.0037	0.0018	CDH3_P87_R	0.0030	0.0049	0.0000	0.0022	0.0000	0.0024
0.9816	0.9926	CDK10_E74_F	0.5089	0.8658	0.7374	0.3979	0.9924	0.6323
0.0000	0.0000	CDK2_P330_R	0.6388	0.8031	0.9715	0.9887	0.0000	0.9649

0.0080	0.0055	CDK6_E256_F	0.0129	0.0061	0.2419	0.9745	0.0000	0.0062
0.0219	0.0179	CDK6_P291_R	0.0218	0.0229	0.0112	0.3073	0.6740	0.0189
0.0036	0.0408	CDKN1A_E101_F	0.7742	0.5989	0.3992	0.0001	0.0000	0.0055
0.0048	0.0000	CDKN1A_P242_F	0.0070	0.0061	0.0252	0.0000	0.9848	0.0077
0.0000	0.0000	CDKN1B_P1161_F	0.0000	0.0000	0.0000	0.0016	0.0000	0.5240
0.0104	0.1437	CDKN2A_E121_R	0.0072	0.0088	0.0445	0.0050	0.0047	0.2308
0.0062	0.0000	CDKN2B_E220_F	0.0039	0.0047	0.2683	0.6017	0.0000	0.0009
0.0107	0.0000	CDM_seq_21_S260_R	0.6383	0.0055	0.0565	0.0022	0.0000	0.0058
0.0023	0.0000	CEACAM1_E57_R	0.0066	0.0074	0.0000	0.0068	0.0000	0.0053
0.0101	0.0071	CEACAM1_P44_R	0.0081	0.0111	0.0011	0.0067	0.1451	0.0092
0.0190	0.1496	CEBPA_P1163_R	0.0174	0.0228	0.0560	0.0094	0.0053	0.0141
0.3534	0.0000	CEBPA_P706_F	0.0020	0.0124	0.1186	0.0023	0.0038	0.0079
0.0042	0.0005	CFTR_P115_F	0.0028	0.0093	0.0000	0.0043	0.0000	0.0041
0.7292	0.0014	CHD2_P451_F	0.0001	0.4902	0.2652	0.0028	0.0000	0.9737
0.1610	0.0018	CHFR_P501_F	0.0036	0.0075	0.0000	0.0026	0.0018	0.0029
0.9879	0.0049	CHFR_P635_R	0.9843	0.9827	0.9840	0.4945	0.8395	0.9858
0.0019	0.0048	CHGA_E52_F	0.0000	0.0039	0.0000	0.0054	0.0000	0.0000
0.2974	0.0050	CHI3L2_E10_F	0.3886	0.0402	0.5533	0.4350	0.0000	0.2560
0.0054	0.0000	CLK1_P538_F	0.0000	0.0810	0.0000	0.0000	0.0000	0.0014
0.0109	0.0195	COL18A1_P365_R	0.0100	0.0125	0.0019	0.0119	0.1461	0.0101
0.0000	0.0000	COL1A1_P5_F	0.0000	0.0000	0.0000	0.0000	0.0000	0.0000
0.3785	0.1003	COL1A2_E299_F	0.3244	0.3160	0.3655	0.2481	0.5229	0.3498
0.0000	0.0000	COL1A2_P407_R	0.6301	0.0000	0.0671	0.0000	0.0015	0.0000
0.0426	0.0730	COL1A2_P48_R	0.0245	0.0319	0.0205	0.0206	0.0189	0.0337
0.0000	0.0000	COL4A3_E205_R	0.0000	0.2174	0.0481	0.0000	0.0000	0.0000
0.0161	0.7462	COL4A3_P545_F	0.0391	0.0296	0.0093	0.7174	0.0297	0.1354
0.0161	0.0027	COL6A1_P283_F	0.4517	0.3314	0.4911	0.0436	0.0252	0.0124
0.0060	0.2018	COL6A1_P425_F	0.0093	0.0148	0.0000	0.0000	0.1036	0.0094
0.1109	0.6062	COPG2_P298_F	0.1096	0.0419	0.0499	0.9682	0.7021	0.0378
0.0251	0.7051	CPA4_P1265_R	0.0344	0.1316	0.0262	0.0412	0.0629	0.0538
0.0105	0.0000	CREB1_P819_F	0.0044	0.0086	0.0000	0.0000	0.0147	0.0068
0.0104	0.0077	CRIP1_P874_R	0.0105	0.0105	0.1069	0.0118	0.4153	0.0100
0.0149	0.0021	CRK_P721_F	0.0035	0.0121	0.0079	0.0043	0.0039	0.0141

0.4713	0.1318	CSF1_P217_F	0.3361	0.2583	0.4322	0.1446	0.4252	0.6678
0.0000	0.0000	CSF1_P339_F	0.0000	0.0000	0.0984	0.0000	0.0000	0.0000
0.0000	0.0000	CSF1R_E26_F	0.0000	0.0005	0.0000	0.0041	0.0025	0.0000
0.0283	0.0141	CSF3R_P472_F	0.0631	0.0404	0.0794	0.0371	0.0124	0.0384
0.0033	0.0055	CSPG2_E38_F	0.0040	0.0076	0.0000	0.0000	0.0000	0.0046
0.0039	0.2452	CSPG2_P82_R	0.0037	0.0061	0.0000	0.0000	0.7544	0.0040
0.7513	0.0000	CSTB_E410_F	0.7995	0.3204	0.7426	0.0052	0.9916	0.9816
0.0000	0.0000	CTAG1B_P4_R	0.0000	0.3044	0.1413	0.0009	0.0021	0.0000
0.0822	0.2046	CTAG1B_P77_F	0.3137	0.2088	0.2828	0.0152	0.9763	0.5957
0.0111	0.0300	CTAG2_P1426_F	0.0107	0.0161	0.0012	0.0230	0.7007	0.0203
0.0000	0.0002	CTGF_E156_F	0.1965	0.0032	0.0253	0.7353	0.0032	0.0004
0.6979	0.9899	CTLA4_P1128_F	0.5796	0.4660	0.3911	0.5249	0.5116	0.2282
0.0031	0.0021	CTNNA1_P185_R	0.0956	0.0049	0.0000	0.0000	0.0051	0.0010
0.0037	0.0040	CTNNA1_P382_R	0.0017	0.0047	0.0134	0.0042	0.0031	0.0037
0.0107	0.0077	CTNNB1_P757_F	0.0068	0.0151	0.0539	0.0045	0.0043	0.0126
0.0000	0.0000	CTSD_P726_F	0.0000	0.0000	0.0000	0.0000	0.0000	0.0000
0.0044	0.0073	CTSH_P238_F	0.0000	0.0000	0.0000	0.0000	0.0000	0.0000
0.0025	0.0000	CTSL_P264_R	0.0029	0.0144	0.0000	0.0043	0.0053	0.0052
0.0055	0.0000	CTSL_P81_F	0.0033	0.0099	0.0000	0.0000	0.0063	0.0031
0.0104	0.0000	CTTN_E29_R	0.0080	0.0064	0.0000	0.0000	0.0037	0.0052
0.0166	0.2021	CYP1A1_P382_F	0.0679	0.0664	0.0257	0.0236	0.0096	0.0606
0.0072	0.0001	CYP2E1_P416_F	0.0104	0.0375	0.0000	0.0116	0.0000	0.0106
0.0000	0.0000	DAB2_P35_F	0.0000	0.0000	0.0000	0.0000	0.0000	0.0000
0.6117	0.0119	DAB2_P468_F	0.0296	0.0312	0.0262	0.0092	0.6996	0.0366
0.0000	0.0012	DAB2IP_P9_F	0.0000	0.0000	0.0000	0.0000	0.0000	0.0000
0.0028	0.0000	DAPK1_P10_F	0.4021	0.1319	0.1407	0.0000	0.0000	0.3675
0.0000	0.0000	DAPK1_P345_R	0.0000	0.0000	0.0478	0.0000	0.0000	0.0000
0.0000	0.0000	DBC1_P351_R	0.0000	0.0000	0.0000	0.0008	0.0000	0.0033
0.0122	0.0119	DCC_P177_F	0.2934	0.0138	0.1461	0.0109	0.0036	0.0127
0.0130	0.0078	DCC_P471_R	0.0122	0.0221	0.0139	0.2367	0.0069	0.0199
0.0000	0.0000	DCN_P1320_R	0.0000	0.0000	0.8129	0.0000	0.0000	0.8746
0.0000	0.0015	DDB2_P407_F	0.6449	0.0000	0.1897	0.0022	0.0000	0.0000
0.0065	0.0031	DDB2_P613_R	0.0049	0.0085	0.0000	0.0000	0.1439	0.0061

0.0061	0.0000	DDR1_E23_R	0.3313	0.0071	0.0644	0.0000	0.0000	0.0072
0.9818	0.0408	DDR2_E331_F	0.9783	0.9616	0.9775	0.9844	0.1436	0.9845
0.0068	0.0000	DES_E228_R	0.0046	0.0121	0.0000	0.0000	0.0038	0.0073
0.0143	0.0117	DHCR24_P406_R	0.5522	0.1501	0.1853	0.0084	0.0138	0.2608
0.0068	0.0018	DIO3_E230_R	0.0054	0.0099	0.0604	0.0070	0.0000	0.6277
0.0138	0.9923	DIO3_P674_F	0.5410	0.0177	0.4509	0.7095	0.4630	0.6319
0.0181	0.0144	DIRAS3_E55_R	0.0229	0.0229	0.0242	0.0174	0.0079	0.0271
0.0000	0.0000	DKC1_E101_F	0.0000	0.0000	0.1956	0.0000	0.0000	0.7826
0.0000	0.0000	DKFZP564O0823_E45_F	0.0029	0.0045	0.0627	0.0000	0.0045	0.0021
0.0041	0.0003	DLC1_P88_R	0.1590	0.0000	0.4689	0.0000	0.0017	0.0000
0.9943	0.9499	DLK1_E227_R	0.9933	0.9928	0.9918	0.9964	0.9969	0.9934
0.9617	0.8414	DLL1_P386_F	0.9675	0.9760	0.9731	0.0000	0.0000	0.9041
0.0061	0.0031	DLL1_P832_F	0.4154	0.0106	0.1155	0.0026	0.0014	0.2278
0.0188	0.2116	DMP1_P134_F	0.2063	0.0614	0.0504	0.3167	0.4762	0.1412
0.0090	0.0116	DNAJC15_E26_R	0.0064	0.0098	0.0000	0.5189	0.0726	0.0098
0.7552	0.5280	DNASE1L1_P108_F	0.6409	0.9871	0.9529	0.9929	0.8158	0.9363
0.9786	0.3103	DNMT1_P100_R	0.9827	0.9825	0.9819	0.9891	0.9913	0.9838
0.9295	0.0000	DSC2_E90_F	0.9699	0.9674	0.9641	0.0000	0.0000	0.8348
0.9820	0.5800	DSP_P440_R	0.9790	0.6949	0.9024	0.4497	0.6168	0.9557
0.5043	0.9929	DST_E31_F	0.5301	0.2169	0.4665	0.0316	0.0000	0.3677
0.0616	0.0273	DST_P262_R	0.0992	0.0677	0.1033	0.1355	0.9027	0.0687
0.0042	0.0000	DUSP4_E61_F	0.0051	0.0066	0.0000	0.0033	0.0000	0.0043
0.0757	0.0128	DUSP4_P925_R	0.4553	0.0261	0.0680	0.6285	0.0130	0.0286
0.6007	0.1040	E2F3_P840_R	0.5455	0.5272	0.4067	0.0000	0.0385	0.0037
0.9476	0.9724	E2F5_P516_R	0.8987	0.9466	0.9582	0.1065	0.4327	0.9653
0.3622	0.5080	EDN1_E50_R	0.3212	0.2359	0.1937	0.5461	0.9842	0.2882
0.0292	0.0108	EDN1_P39_R	0.0129	0.0305	0.0423	0.1719	0.4805	0.0328
0.9223	0.0000	EDNRB_P148_R	0.9694	0.9841	0.9781	0.9876	0.0015	0.9226
0.0058	0.0308	EFNA1_P591_R	0.3888	0.0137	0.4174	0.0000	0.0104	0.0000
0.0000	0.0000	EFNA1_P7_F	0.6134	0.0028	0.9206	0.0017	0.7326	0.2697
0.5112	0.2298	EFNB1_E69_F	0.5714	0.3026	0.1955	0.0019	0.0076	0.0044
0.0000	0.0032	EFNB3_P442_R	0.1457	0.0030	0.0719	0.0000	0.0000	0.0002
0.0000	0.0012	EGF_E339_F	0.0000	0.3402	0.5261	0.0000	0.0011	0.3482

0.5280	0.0040	EGFR_E295_R	0.0032	0.1900	0.0786	0.0058	0.0030	0.0034
0.0000	0.0000	EGFR_P260_R	0.7010	0.0000	0.7594	0.0000	0.0000	0.5214
0.9717	0.5591	EGR4_P479_F	0.6471	0.8609	0.9802	0.9908	0.6392	0.7263
0.9790	0.0000	EIF2AK2_E103_R	0.9619	0.9454	0.9713	0.0000	0.9377	0.9763
0.9866	0.0045	EIF2AK2_P313_F	0.9871	0.9861	0.9864	0.9922	0.0046	0.9847
0.9741	0.0000	ELK1_E156_F	0.9655	0.9274	0.9760	0.9870	0.0000	0.8024
0.6355	0.3478	EMR3_P39_R	0.3183	0.6789	0.6248	0.0097	0.4740	0.7234
0.9843	0.0000	ENC1_P484_R	0.9792	0.9807	0.9801	0.8164	0.0000	0.9804
0.3127	0.0183	EPHA1_E46_R	0.2485	0.3248	0.3971	0.0228	0.0178	0.4144
0.0051	0.0034	EPHA1_P119_R	0.0039	0.0067	0.0137	0.0037	0.0000	0.0061
0.0028	0.0000	EPHA2_P203_F	0.0640	0.0055	0.1221	0.0018	0.9924	0.1665
0.0267	0.0000	EPHA3_E156_R	0.0011	0.0022	0.0646	0.0000	0.0008	0.1353
0.6925	0.9443	EPHA7_E6_F	0.3725	0.5913	0.5752	0.5644	0.0017	0.2419
0.9824	0.9946	EPHA7_P205_R	0.9855	0.9868	0.9888	0.9854	0.9910	0.9842
0.0178	0.0204	EPHA8_P256_F	0.0213	0.0247	0.0399	0.0255	0.0116	0.0294
0.5089	0.0092	EPHB1_P503_F	0.0090	0.2422	0.2593	0.0756	0.0000	0.1657
0.0263	0.7993	EPHB2_E297_F	0.3639	0.0849	0.0403	0.0746	0.7456	0.0520
0.9003	0.1249	EPHB2_P165_R	0.9702	0.4617	0.3300	0.0135	0.0000	0.9766
0.0030	0.0000	EPHB3_E0_F	0.0000	0.0036	0.1993	0.0000	0.0003	0.0000
0.0197	0.1602	EPHB3_P569_R	0.0195	0.0176	0.0075	0.4723	0.0000	0.0218
0.0453	0.0083	EPHB4_P313_R	0.4069	0.3549	0.4571	0.2680	0.0212	0.3555
0.9449	0.0593	EPHB6_E342_F	0.2056	0.5648	0.2445	0.0193	0.6390	0.0579
0.0042	0.0000	EPHB6_P827_R	0.0000	0.0044	0.0814	0.0000	0.0000	0.0000
0.9684	0.0000	EPHX1_P1358_R	0.9779	0.9851	0.9769	0.9915	0.0000	0.9783
0.1054	0.7874	EPM2A_P113_F	0.0426	0.2971	0.1555	0.8263	0.7651	0.1831
0.0244	0.0198	EPM2A_P64_R	0.2824	0.0855	0.0120	0.4115	0.4775	0.0165
0.0060	0.0002	EPO_E244_R	0.0001	0.0018	0.0000	0.0041	0.0000	0.0708
0.0000	0.0000	EPO_P162_R	0.0305	0.5212	0.2316	0.0000	0.0000	0.3149
0.9602	0.1144	EPS8_E231_F	0.9470	0.6759	0.9672	0.9913	0.0000	0.9720
0.3640	0.0000	EPS8_P437_F	0.6368	0.0000	0.2984	0.0040	0.0000	0.2109
0.6812	0.0000	ERBB2_P59_R	0.9379	0.9273	0.9245	0.0000	0.0000	0.9833
0.0162	0.0101	ERBB3_E331_F	0.4767	0.4456	0.3970	0.0289	0.0101	0.6739
0.0105	0.0500	ERBB3_P870_R	0.1807	0.3752	0.2054	0.0040	0.0979	0.2243

0.0142	0.3795	ERBB4_P255_F	0.0172	0.0229	0.1336	0.6466	0.2276	0.0153
0.9133	0.0000	ERBB4_P541_F	0.9398	0.8825	0.9395	0.0874	0.9925	0.9135
0.4046	0.7003	ERCC1_P354_F	0.5963	0.7223	0.8744	0.9240	0.0000	0.8247
0.7438	0.2339	ERCC3_P1210_R	0.8349	0.6987	0.7899	0.0306	0.9933	0.7161
0.4422	0.0157	ERG_E28_F	0.3591	0.4800	0.3927	0.0248	0.0324	0.1225
0.9286	0.1362	ERN1_P809_R	0.9738	0.9768	0.9749	0.1953	0.0000	0.9817
0.9792	0.7382	ESR1_E298_R	0.8809	0.9768	0.9666	0.0342	0.7111	0.8790
0.8560	0.9923	ESR1_P151_R	0.8038	0.4495	0.6844	0.0028	0.0039	0.6834
0.8514	0.4218	ESR2_E66_F	0.4214	0.5031	0.5503	0.0487	0.0309	0.5994
0.4143	0.9800	ESR2_P162_F	0.0314	0.1723	0.1576	0.0319	0.5218	0.4277
0.1639	0.0503	ETS1_E253_R	0.6898	0.0495	0.3234	0.1596	0.2387	0.5538
0.9797	0.0000	ETS1_P559_R	0.9767	0.9818	0.9793	0.0000	0.0420	0.9822
0.6428	0.3406	ETS2_P684_F	0.7446	0.7085	0.9716	0.9900	0.9923	0.9798
0.0140	0.1346	ETS2_P835_F	0.0109	0.1446	0.1521	0.0109	0.0000	0.0116
0.9600	0.9931	ETV1_P235_F	0.9773	0.9766	0.9735	0.0000	0.0000	0.9745
0.0063	0.0000	ETV1_P515_F	0.6748	0.0173	0.0000	0.1423	0.0000	0.0114
0.2635	0.0046	ETV6_E430_F	0.4959	0.4884	0.3328	0.0089	0.0059	0.3542
0.9496	0.1453	EVI1_E47_R	0.6351	0.9309	0.9661	0.3169	0.9880	0.4603
0.0028	0.0000	EVI1_P30_R	0.4542	0.0061	0.1925	0.0036	0.0000	0.2157
0.0056	0.0000	EVI2A_P94_R	0.0044	0.0055	0.3434	0.0000	0.0000	0.4294
0.0441	0.3314	EXT1_E197_F	0.2570	0.0239	0.1253	0.0126	0.5846	0.0166
0.3221	0.8913	EYA4_E277_F	0.0058	0.1988	0.0592	0.0447	0.0085	0.4499
0.1841	0.0000	EYA4_P508_F	0.8178	0.6163	0.6035	0.0057	0.0024	0.7119
0.9620	0.0000	EYA4_P794_F	0.9435	0.9693	0.9567	0.0000	0.0000	0.9835
0.9829	0.0000	F2R_P839_F	0.9786	0.9833	0.9806	0.0000	0.0000	0.9485
0.4713	0.2160	F2R_P88_F	0.8075	0.8599	0.8780	0.0037	0.0037	0.5570
0.4737	0.8028	FABP3_E113_F	0.5308	0.6035	0.6342	0.0731	0.0059	0.7402
0.2085	0.9851	FABP3_P598_F	0.1141	0.1633	0.3215	0.3371	0.3487	0.1497
0.7997	0.0004	FANCE_P356_R	0.8228	0.0089	0.7316	0.0000	0.0016	0.0063
0.5508	0.6504	FANCF_P13_F	0.2066	0.5926	0.6155	0.6969	0.0184	0.6388
0.0533	0.0624	FANCG_E207_R	0.0341	0.4996	0.1738	0.1746	0.0744	0.5556
0.9619	0.5974	FAS_P322_R	0.6138	0.8311	0.8008	0.6559	0.8258	0.6962
0.0279	0.0911	FASTK_P257_F	0.5727	0.0614	0.7464	0.6001	0.0156	0.0526

0.2275	0.0000	FAT_P973_R	0.4450	0.0047	0.3600	0.0102	0.0000	0.5232
0.9833	0.2604	FER_P581_F	0.7200	0.9546	0.9599	0.2316	0.3431	0.9722
0.9795	0.0000	FES_P223_R	0.9731	0.9634	0.9690	0.0000	0.0000	0.9659
0.9407	0.8407	FGF1_E5_F	0.9770	0.9578	0.9793	0.0473	0.3868	0.9822
0.0127	0.4228	FGF12_E61_R	0.4139	0.5039	0.2651	0.3393	0.4465	0.0712
0.9817	0.8158	FGF12_P210_R	0.9821	0.9737	0.9836	0.9800	0.9913	0.9849
0.6432	0.2371	FGF2_P153_F	0.0290	0.0395	0.4549	0.0120	0.0107	0.1963
0.7549	0.0417	FGF2_P229_F	0.1680	0.1005	0.6056	0.9059	0.6173	0.0535
0.9640	0.9870	FGF3_E198_R	0.7675	0.3906	0.4163	0.9846	0.9872	0.8631
0.7341	0.0001	FGF3_P171_R	0.7655	0.8050	0.9497	0.0000	0.0000	0.9772
0.8725	0.9912	FGF5_E16_F	0.9609	0.9809	0.9768	0.7054	0.0572	0.9805
0.0849	0.9933	FGF5_P238_R	0.4301	0.5875	0.4368	0.0000	0.9912	0.5694
0.6547	0.7153	FGF6_E294_F	0.1009	0.0429	0.6129	0.7399	0.6776	0.0528
0.3202	0.0000	FGF7_P44_F	0.5587	0.0022	0.6330	0.0002	0.9865	0.5520
0.1543	0.0056	FGF8_E183_F	0.0245	0.0295	0.0000	0.0000	0.3362	0.2802
0.5665	0.0000	FGF8_P473_F	0.9247	0.4944	0.5042	0.0000	0.0000	0.4552
0.2789	0.0000	FGFR1_E317_F	0.0079	0.2038	0.3565	0.2374	0.0083	0.7159
0.0096	0.0073	FGFR2_P266_R	0.0085	0.1248	0.0171	0.0065	0.0072	0.1421
0.9686	0.9900	FGFR3_E297_R	0.9601	0.9037	0.8731	0.0000	0.7883	0.8227
0.9777	0.8352	FGFR3_P1152_R	0.9615	0.9024	0.9162	0.8729	0.9951	0.9397
0.9830	0.0019	FHIT_E19_R	0.9817	0.9725	0.9795	0.9928	0.9911	0.9789
0.7139	0.0000	FHIT_P93_R	0.1663	0.1391	0.1983	0.0045	0.0035	0.0075
0.0018	0.0004	FHL1_E229_R	0.4213	0.1998	0.2231	0.0031	0.0000	0.0000
0.9446	0.0610	FLI1_P620_R	0.8871	0.9185	0.9652	0.0000	0.0039	0.9656
0.1117	0.8931	FLT1_E444_F	0.3085	0.6289	0.7157	0.0250	0.0198	0.5292
0.0262	0.2229	FLT1_P302_F	0.1990	0.0465	0.0503	0.0190	0.5301	0.0445
0.0046	0.0133	FLT1_P615_R	0.0033	0.1895	0.2729	0.0077	0.4221	0.5761
0.4740	0.0138	FLT3_E326_R	0.1718	0.3920	0.1343	0.0070	0.0043	0.3767
0.9859	0.8749	FLT4_P180_R	0.9840	0.9835	0.9810	0.9789	0.9928	0.9238
0.5261	0.9218	FMR1_P62_R	0.2654	0.3103	0.2351	0.5127	0.9388	0.2761
0.0031	0.0000	FN1_E469_F	0.1824	0.6317	0.2576	0.0000	0.0000	0.0009
0.3828	0.3199	FN1_P229_R	0.2327	0.4065	0.1918	0.0127	0.5883	0.9053
0.6939	0.0003	FOSL2_E384_R	0.8281	0.9510	0.9854	0.0044	0.9929	0.8960

0.0110	0.0075	FRK_P36_F	0.3124	0.0195	0.1236	0.2076	0.0000	0.2121
0.7267	0.1252	FRZB_E186_R	0.0000	0.9667	0.9116	0.9739	0.0000	0.8724
0.5991	0.0000	FRZB_P406_F	0.0008	0.2127	0.1344	0.0042	0.0000	0.0000
0.0196	0.0000	FVT1_P225_F	0.3295	0.2463	0.1667	0.0061	0.3587	0.0084
0.9312	0.9909	FYN_P352_R	0.9735	0.8635	0.7988	0.9945	0.9945	0.3916
0.0000	0.9931	FZD7_E296_F	0.9774	0.9848	0.9770	0.0289	0.0000	0.9811
0.9756	0.9741	FZD9_E458_F	0.9791	0.9749	0.9690	0.0000	0.9928	0.9703
0.0024	0.0072	FZD9_P15_R	0.0000	0.0010	0.0346	0.0000	0.0045	0.0002
0.2465	0.2639	FZD9_P175_F	0.3781	0.4956	0.4073	0.0839	0.9910	0.2200
0.0000	0.0017	G6PD_E190_F	0.3625	0.4127	0.8021	0.9909	0.0024	0.0000
0.0147	0.0106	G6PD_P196_F	0.5144	0.2542	0.7262	0.6585	0.5156	0.3832
0.0022	0.0028	GABRB3_E42_F	0.2922	0.0044	0.0000	0.0063	0.0000	0.4369
0.0243	0.3865	GADD45A_P737_R	0.1365	0.1606	0.1507	0.4138	0.0202	0.0249
0.0184	0.1919	GALR1_E52_F	0.0163	0.0152	0.0070	0.0088	0.0000	0.0215
0.0086	0.0040	GALR1_P80_F	0.0082	0.2176	0.0260	0.9874	0.0000	0.1626
0.0078	0.3083	GAS1_E22_F	0.0068	0.1861	0.0014	0.0467	0.0066	0.0102
0.0017	0.0031	GAS1_P754_R	0.0000	0.0028	0.0198	0.0028	0.0000	0.0000
0.0120	0.3219	GAS7_E148_F	0.8467	0.0149	0.0470	0.0165	0.0094	0.0108
0.0000	0.0000	GAS7_P622_R	0.0000	0.0354	0.0000	0.0000	0.0000	0.0000
0.4748	0.0045	GATA6_P21_R	0.0045	0.1280	0.0673	0.0020	0.0024	0.1559
0.0175	0.0068	GATA6_P726_F	0.0120	0.0126	0.0053	0.1656	0.0051	0.0297
0.0038	0.0750	GFI1_E136_F	0.0021	0.0052	0.1099	0.0293	0.0000	0.0039
0.0088	0.0039	GFI1_P45_R	0.0064	0.0547	0.0289	0.0018	0.0056	0.0500
0.0136	0.0064	GJB2_E43_F	0.0083	0.0182	0.1454	0.0000	0.0000	0.0139
0.5015	0.1493	GJB2_P791_R	0.2802	0.1377	0.1052	0.0132	0.0059	0.0088
0.0416	0.2777	GJB2_P931_R	0.7675	0.0369	0.1029	0.2741	0.3366	0.0311
0.0204	0.0050	GLA_P112_F	0.9289	0.3793	0.8948	0.8351	0.4490	0.1763
0.9815	0.0000	GLI2_E90_F	0.9720	0.9788	0.9800	0.9912	0.0000	0.9841
0.8236	0.6182	GLI3_E148_R	0.9027	0.7918	0.9263	0.9678	0.2995	0.8221
0.9791	0.8038	GML_P281_R	0.8775	0.9827	0.9734	0.9906	0.6402	0.9815
0.9800	0.0000	GNAS_E58_F	0.8566	0.8676	0.8855	0.0000	0.0000	0.4482
0.0136	0.0030	GP1BB_E23_F	0.0082	0.0664	0.0669	0.0500	0.0040	0.0082
0.1598	0.0000	GPC3_P235_R	0.5700	0.0046	0.5937	0.0033	0.0000	0.0068

0.9790	0.0000	GPR116_E328_R	0.9864	0.9868	0.9840	0.9923	0.9799	0.9863
0.0092	0.0926	GPX1_E46_R	0.0088	0.0102	0.0003	0.0061	0.2641	0.0092
0.0483	0.5512	GPX1_P194_F	0.0193	0.0274	0.0777	0.4630	0.0257	0.0281
0.0915	0.0105	GPX3_E178_F	0.1375	0.3278	0.2431	0.0075	0.0092	0.2234
0.0057	0.0886	GRB10_E85_R	0.0077	0.0073	0.0000	0.0054	0.0042	0.0052
0.0032	0.0045	GRB10_P260_F	0.0020	0.0144	0.0000	0.0857	0.1329	0.0058
0.0042	0.0905	GRB10_P496_R	0.0046	0.2809	0.1885	0.0086	0.0000	0.8290
0.9817	0.1442	GRPR_P200_R	0.9378	0.8434	0.9296	0.1665	0.9925	0.9811
0.9431	0.9908	GSTM1_P363_F	0.8647	0.9355	0.8053	0.9914	0.1561	0.5734
0.0044	0.6247	GSTM2_E153_F	0.0045	0.0062	0.0268	0.1100	0.0011	0.1922
0.0030	0.0000	GSTM2_P109_R	0.0000	0.0081	0.0203	0.0031	0.0000	0.2922
0.0000	0.0016	GSTM2_P453_R	0.0009	0.5901	0.6631	0.0000	0.0000	0.8519
0.0073	0.0000	GSTP1_E322_R	0.0058	0.0063	0.0000	0.0007	0.0000	0.0076
0.0193	0.3359	GSTP1_P74_F	0.0291	0.0494	0.0661	0.2060	0.4374	0.0315
0.0077	0.0000	GSTP1_seq_38_S153_R	0.0072	0.0092	0.0005	0.0000	0.0000	0.0095
0.0041	0.0000	GUCY2D_E419_R	0.0069	0.0072	0.0000	0.0001	0.0040	0.0038
0.0000	0.0000	HBEGF_P32_R	0.0000	0.0000	0.0348	0.0000	0.0000	0.0006
0.9747	0.0000	HBII-13_E48_F	0.9626	0.9657	0.9705	0.9853	0.0000	0.9600
0.0405	0.5549	HBII-52_P563_F	0.8253	0.6689	0.8742	0.9888	0.0242	0.7150
0.0132	0.2967	HCK_P46_R	0.0088	0.1921	0.0137	0.0073	0.0087	0.0107
0.0165	0.5826	HCK_P858_F	0.0162	0.0200	0.1128	0.0308	0.0448	0.0198
0.0093	0.0066	HDAC1_P414_R	0.6869	0.4060	0.2645	0.0000	0.4955	0.0598
0.0000	0.0061	HDAC11_P556_F	0.0017	0.0000	0.0041	0.0043	0.0032	0.0637
0.0016	0.0010	HDAC9_P137_R	0.0000	0.0041	0.0761	0.0000	0.0072	0.0054
0.0014	0.0044	HFE_E273_R	0.0011	0.0040	0.0000	0.0037	0.0033	0.0000
0.0209	0.1777	HHIP_P307_R	0.0239	0.0769	0.0506	0.0205	0.0104	0.0849
0.9229	0.0000	HIC1_E151_F	0.0000	0.0000	0.0581	0.0000	0.0000	0.0000
0.0235	0.0128	HIC1_P565_R	0.0158	0.0191	0.0042	0.0081	0.0000	0.0220
0.0053	0.0040	HIC2_P498_F	0.0044	0.0057	0.0000	0.0000	0.0041	0.0058
0.0590	0.0071	HIC2_P528_R	0.0043	0.0110	0.0000	0.0035	0.0000	0.0052
0.0000	0.0086	HIF1A_P488_F	0.0019	0.0000	0.0000	0.0064	0.0000	0.0026
0.9836	0.0000	HLA-DOB_P357_R	0.9825	0.9715	0.9756	0.9754	0.0000	0.9748
0.0157	0.1568	HLA-DPA1_P205_R	0.6511	0.0177	0.0686	0.1164	0.1035	0.0110

0.0042	0.0243	HLA-DPA1_P28_R	0.0031	0.0071	0.0744	0.0049	0.0000	0.0052
0.0401	0.5597	HLA-DPB1_E2_R	0.0368	0.0815	0.0210	0.2183	0.0000	0.0667
0.0000	0.0000	HLA-DRA_P132_R	0.0000	0.0000	0.0413	0.0000	0.0000	0.0000
0.0000	0.0000	HLA-F_E402_F	0.0000	0.0000	0.0000	0.0000	0.0000	0.0000
0.0173	0.0285	HLF_E192_F	0.0144	0.0167	0.0084	0.0294	0.7702	0.0191
0.0028	0.0000	HOXA11_E35_F	0.0032	0.0043	0.0000	0.0000	0.0000	0.0023
0.0238	0.5713	HOXA11_P698_F	0.0243	0.0222	0.0088	0.0196	0.0113	0.0200
0.9831	0.0000	HOXA5_E187_F	0.9664	0.9832	0.9795	0.9900	0.5594	0.9760
0.0114	0.0000	HOXA9_E252_R	0.0142	0.0256	0.0733	0.2450	0.0000	0.0268
0.0794	0.1646	HOXA9_P1141_R	0.2787	0.1845	0.0179	0.0078	0.3624	0.0190
0.0010	0.0000	HOXA9_P303_F	0.0000	0.0007	0.0000	0.0012	0.0000	0.0000
0.3867	0.0037	HOXB13_E21_F	0.0746	0.0685	0.0326	0.0044	0.0020	0.0024
0.0041	0.0000	HOXB13_P17_R	0.0023	0.1552	0.0920	0.0008	0.0010	0.3144
0.0285	0.0734	HOXC6_P456_R	0.0271	0.0464	0.0495	0.0290	0.7854	0.0377
0.0061	0.0000	HPN_P374_R	0.0000	0.0039	0.0451	0.0000	0.0000	0.0027
0.0000	0.9919	HPN_P823_F	0.6024	0.0000	0.4197	0.0000	0.0000	0.0000
0.0211	0.5087	HPSE_P29_F	0.3606	0.0270	0.0328	0.0260	0.7187	0.0258
0.0036	0.0029	HPSE_P93_F	0.0050	0.0055	0.0000	0.0057	0.0000	0.0044
0.0011	0.0000	HRASLS_E72_R	0.0000	0.0007	0.0883	0.0000	0.0000	0.0000
0.0000	0.0018	HS3ST2_E145_R	0.0000	0.0000	0.0330	0.0000	0.0026	0.0000
0.1824	0.0046	HS3ST2_P546_F	0.3322	0.7274	0.0251	0.0000	0.0050	0.0028
0.0594	0.4124	HSD17B12_E145_R	0.0152	0.0194	0.0117	0.0000	0.0128	0.0194
0.0000	0.0008	HTR1B_P222_F	0.0004	0.0006	0.0000	0.0000	0.0000	0.4503
0.0011	0.0046	HTR2A_P853_F	0.0010	0.0015	0.1442	0.9871	0.0069	0.0000
0.1214	0.9858	IAPP_E280_F	0.9775	0.9749	0.9774	0.9881	0.9906	0.9749
0.0000	0.0000	ICA1_P61_F	0.0000	0.0000	0.0000	0.0000	0.0028	0.0000
0.0000	0.0008	ICA1_P72_R	0.0000	0.0019	0.0292	0.0000	0.0012	0.0016
0.0109	0.3173	ICAM1_E242_F	0.0115	0.0183	0.0592	0.3415	0.0042	0.4373
0.0000	0.0000	ICAM1_P119_R	0.0005	0.0037	0.0000	0.0031	0.0048	0.0000
0.0000	0.0006	ICAM1_P386_R	0.0000	0.0030	0.0000	0.0000	0.0033	0.0000
0.0040	0.0007	ID1_P659_R	0.0047	0.0054	0.0239	0.0000	0.0003	0.0000
0.8125	0.0075	IFNG_P459_R	0.9384	0.9720	0.9775	0.2222	0.0058	0.7642
0.2424	0.1326	IFNGR1_P307_F	0.0073	0.0137	0.0051	0.1404	0.5449	0.0040

0.0343	0.1139	IFNGR2_E164_F	0.0320	0.0251	0.0274	0.0217	0.1178	0.0465
0.7763	0.0000	IFNGR2_P377_R	0.4460	0.0018	0.2198	0.0010	0.9134	0.0000
0.9063	0.0000	IGF1_E394_F	0.9719	0.8477	0.9682	0.0000	0.0000	0.9759
0.7458	0.0000	IGF1_P933_F	0.9760	0.5342	0.7897	0.0000	0.0833	0.9536
0.0021	0.0015	IGF1R_E186_R	0.0000	0.0033	0.0000	0.0007	0.0019	0.0036
0.0285	0.2742	IGF1R_P325_R	0.0121	0.0179	0.0061	0.0122	0.1257	0.0226
0.0000	0.0000	IGF2_E134_R	0.0037	0.5059	0.1337	0.0014	0.0000	0.0000
0.0212	0.0145	IGF2_P1036_R	0.0319	0.1218	0.0132	0.1241	0.0076	0.0198
0.0146	0.0688	IGF2_P36_R	0.0131	0.0174	0.3727	0.0097	0.2368	0.0214
0.0000	0.0000	IGF2AS_P203_F	0.0000	0.0000	0.0000	0.0000	0.0000	0.0000
0.1313	0.9197	IGF2R_P396_R	0.0998	0.1456	0.1063	0.9259	0.1961	0.2345
0.9820	0.0682	IGFBP1_E48_R	0.6475	0.1999	0.1865	0.0162	0.0000	0.4754
0.9065	0.0344	IGFBP1_P12_R	0.0055	0.7070	0.4647	0.1851	0.0266	0.8408
0.0000	0.0008	IGFBP2_P306_F	0.0000	0.0000	0.0535	0.0000	0.0000	0.0000
0.0295	0.0108	IGFBP3_E65_R	0.0140	0.0251	0.0679	0.0125	0.1922	0.0179
0.0068	0.0000	IGFBP3_P1035_F	0.0000	0.0000	0.0000	0.0000	0.0036	0.0000
0.0173	0.1522	IGFBP3_P423_R	0.0117	0.0159	0.0267	0.0039	0.0049	0.2386
0.0076	0.4008	IGFBP7_P371_F	0.0056	0.0083	0.0000	0.1318	0.3860	0.0092
0.0077	0.0321	IGSF4_P454_F	0.0051	0.0075	0.0000	0.0048	0.0020	0.0011
0.0214	0.1842	IGSF4_P86_R	0.0142	0.0329	0.0105	0.0880	0.0077	0.0156
0.9853	0.0039	IGSF4C_E65_F	0.9829	0.9559	0.8668	0.0948	0.7547	0.8607
0.0050	0.0136	IGSF4C_P533_R	0.0065	0.0134	0.0054	0.0109	0.0000	0.0102
0.0035	0.0017	IHH_E186_F	0.0019	0.0082	0.0000	0.0000	0.0000	0.0012
0.0341	0.0071	IHH_P246_R	0.3172	0.2915	0.1561	0.0000	0.0074	0.0049
0.0137	0.1404	IHH_P529_F	0.0166	0.0138	0.0034	0.0000	0.1175	0.0069
0.0010	0.0000	IL10_P85_F	0.2069	0.0026	0.0713	0.0000	0.0000	0.1431
0.0000	0.0000	IL11_P11_R	0.0072	0.5281	0.1040	0.0000	0.0000	0.0086
0.0014	0.0024	IL12A_E287_R	0.0044	0.0054	0.0189	0.0025	0.0000	0.0027
0.0000	0.5443	IL12B_P392_R	0.1985	0.1754	0.7588	0.0016	0.2091	0.7410
0.9789	0.9586	IL13_E75_R	0.9764	0.9586	0.9821	0.1559	0.0421	0.9809
0.0047	0.0015	IL17RB_E164_R	0.0007	0.0030	0.0643	0.0020	0.0029	0.0000
0.0000	0.0000	IL17RB_P788_R	0.0000	0.0000	0.0000	0.0000	0.0000	0.0000
0.0299	0.2332	IL18BP_P51_R	0.0305	0.0346	0.0246	0.0180	0.3717	0.0411

0.9721	0.9916	IL1A_E113_R	0.9852	0.9876	0.9725	0.9890	0.9915	0.9854
0.8583	0.9889	IL1RN_E42_F	0.9485	0.9870	0.9837	0.9862	0.0000	0.9764
0.9787	0.0000	IL3_P556_F	0.9755	0.9148	0.9753	0.1976	0.0000	0.9706
0.3873	0.0078	IL6_E168_F	0.0066	0.4279	0.2542	0.0974	0.0513	0.5395
0.0000	0.0006	IL8_E118_R	0.0026	0.0042	0.0378	0.0031	0.0000	0.0015
0.0000	0.0000	IL8_P83_F	0.0000	0.0000	0.0000	0.0000	0.0000	0.0000
0.0000	0.0000	IMPACT_P186_F	0.0000	0.0000	0.0000	0.0000	0.0000	0.0000
0.0000	0.0018	IMPACT_P234_R	0.0028	0.0081	0.0000	0.0000	0.0000	0.0025
0.4867	0.0046	INSR_P1063_R	0.0000	0.4436	0.1370	0.0000	0.0019	0.0000
0.0000	0.0023	IPF1_P234_F	0.0030	0.0034	0.1127	0.0003	0.0000	0.0037
0.6021	0.0638	IRAK1_P455_R	0.6239	0.0083	0.3828	0.0070	0.0019	0.0080
0.0089	0.0101	IRAK3_E130_F	0.0086	0.0126	0.0020	0.0000	0.0051	0.0105
0.0000	0.0053	IRAK3_P13_F	0.0098	0.0105	0.0000	0.0200	0.0000	0.0065
0.0092	0.0000	IRF5_P123_F	0.0178	0.1057	0.0883	0.0000	0.0054	0.2104
0.1263	0.0098	IRF7_P277_R	0.0084	0.0106	0.0031	0.0143	0.6154	0.1062
0.0084	0.0000	ISL1_E87_R	0.3019	0.0058	0.0608	0.0032	0.0000	0.1843
0.1349	0.0000	ISL1_P379_F	0.1437	0.0132	0.1374	0.0000	0.0006	0.0050
0.9615	0.0000	ISL1_P554_F	0.0000	0.0000	0.0000	0.6363	0.0000	0.0000
0.0083	0.2109	ITGA2_E120_F	0.0076	0.0092	0.0021	0.0097	0.0000	0.2672
0.0019	0.0000	ITGA2_P26_R	0.0037	0.0005	0.0000	0.0025	0.0000	0.0000
0.5856	0.0025	ITGB4_E144_F	0.0054	0.0108	0.0230	0.0073	0.0000	0.1293
0.0000	0.0015	ITGB4_P517_F	0.2244	0.0000	0.0803	0.0000	0.0000	0.0000
0.0000	0.0000	ITPR3_E86_R	0.0000	0.0000	0.0000	0.0000	0.0000	0.0000
0.0000	0.0000	ITPR3_P1112_F	0.0000	0.0000	0.0000	0.0000	0.0017	0.0000
0.0054	0.0000	JAG2_E54_F	0.0073	0.0098	0.0000	0.0000	0.0000	0.0064
0.0115	0.0049	JAG2_P264_F	0.0148	0.0145	0.0058	0.2143	0.1445	0.0171
0.0838	0.0115	JAK2_P772_R	0.0089	0.0134	0.0992	0.0127	0.1009	0.0133
0.0081	0.0000	JAK3_E64_F	0.0065	0.0102	0.0086	0.0058	0.0035	0.0043
0.3342	0.0088	JAK3_P156_R	0.0125	0.0187	0.0371	0.0066	0.0043	0.0121
0.1210	0.0915	JUNB_P1149_R	0.0558	0.0631	0.0507	0.0693	0.5627	0.1111
0.0104	0.0038	KCNK4_E3_F	0.0025	0.3625	0.1630	0.9915	0.0000	0.2340
0.0133	0.0364	KDR_E79_F	0.0152	0.0123	0.0017	0.0188	0.0178	0.0137
0.0015	0.0026	KDR_P445_R	0.0046	0.0095	0.0040	0.0052	0.0000	0.0063

0.0093	0.0000	KIAA1804_P689_R	0.0102	0.0183	0.0023	0.0036	0.1450	0.0103
0.0038	0.1229	KIT_P367_R	0.0055	0.0118	0.0004	0.1185	0.0000	0.0077
0.0082	0.0030	KIT_P405_F	0.1099	0.0084	0.0848	0.0050	0.0023	0.1809
0.0066	0.0031	KLF5_E190_R	0.0041	0.0064	0.1517	0.0132	0.0000	0.2512
0.0000	0.0000	KLK11_P103_R	0.9771	0.8242	0.9684	0.9891	0.0018	0.8466
0.9646	0.0000	KLK11_P1290_F	0.8523	0.8761	0.9756	0.9891	0.0000	0.9775
0.2349	0.6555	KRAS_E82_F	0.8492	0.7450	0.6173	0.0278	0.0137	0.4987
0.0762	0.4897	KRAS_P651_F	0.0728	0.0659	0.0432	0.0610	0.4797	0.0757
0.8413	0.9917	KRT13_P341_R	0.9778	0.9804	0.9789	0.0000	0.9919	0.9762
0.6905	0.1910	KRT5_P308_F	0.9658	0.8469	0.9548	0.2245	0.3285	0.6424
0.0073	0.1784	L1CAM_P148_R	0.0030	0.0067	0.0123	0.0000	0.0000	0.0023
0.6724	0.0053	L1CAM_P19_F	0.6583	0.2325	0.5316	0.0000	0.0000	0.0070
0.0093	0.0131	LAMC1_E466_R	0.1463	0.0037	0.0000	0.0025	0.0029	0.0041
0.0118	0.0244	LIF_E208_F	0.0109	0.0164	0.0162	0.0258	0.3908	0.0155
0.0022	0.0000	LIF_P383_R	0.0057	0.5621	0.3763	0.0946	0.3619	0.5034
0.0170	0.0000	LIG4_P194_F	0.0093	0.0135	0.0013	0.1115	0.0000	0.0080
0.0031	0.0011	LMO1_E265_R	0.0000	0.0038	0.0000	0.0034	0.0000	0.0000
0.0111	0.0032	LMO1_P169_F	0.0054	0.0092	0.0000	0.0110	0.0000	0.0085
0.0120	0.0712	LOX_P313_R	0.0122	0.0235	0.0516	0.0171	0.2486	0.0162
0.0030	0.0000	LOX_P71_F	0.0022	0.0053	0.0000	0.0046	0.0016	0.0017
0.0208	0.4991	LRP2_E20_F	0.2089	0.0411	0.0271	0.0212	0.8089	0.0332
0.0059	0.0034	LRRC32_P865_R	0.0068	0.0101	0.0146	0.0022	0.0000	0.0065
0.0308	0.4914	LTB4R_E64_R	0.0559	0.0471	0.0287	0.4480	0.0209	0.0375
0.0000	0.0000	LYN_E353_F	0.0717	0.4238	0.0016	0.0000	0.0000	0.0000
0.0705	0.0548	LYN_P241_F	0.0792	0.0338	0.0563	0.0350	0.0220	0.0394
0.0000	0.0000	MAF_P826_R	0.0000	0.0000	0.0000	0.0000	0.0000	0.0000
0.0000	0.0000	MAGEC3_P903_F	0.8931	0.9327	0.5343	0.0133	0.0000	0.4888
0.9451	0.0000	MAGEL2_E166_R	0.1969	0.9600	0.9547	0.2589	0.1422	0.0000
0.4608	0.0029	MALT1_P406_R	0.0000	0.0023	0.0760	0.0036	0.0000	0.0000
0.0000	0.0000	MAP2K6_E297_F	0.0000	0.3535	0.0000	0.0011	0.0000	0.0000
0.0100	0.0098	MAP2K6_P297_R	0.0491	0.0144	0.1544	0.0259	0.0104	0.1136
0.0234	0.0077	MAP3K1_P7_F	0.0285	0.0353	0.0251	0.0132	0.0081	0.0431
0.0217	0.0066	MAP3K9_E17_R	0.0366	0.1201	0.1234	0.1806	0.3818	0.0097

0.1087	0.0089	MAPK12_E165_R	0.1343	0.0106	0.0000	0.0374	0.0000	0.0082
0.0000	0.0000	MAPK12_P416_F	0.0000	0.0000	0.0000	0.0000	0.0029	0.0000
0.0057	0.0019	MAPK14_P327_R	0.0078	0.2490	0.0000	0.9741	0.0000	0.0044
0.9783	0.0000	MAPK4_E273_R	0.7900	0.9797	0.9034	0.0000	0.0000	0.7938
0.9731	0.6188	MAPK9_P1175_F	0.8109	0.9762	0.9722	0.0000	0.9851	0.9192
0.9821	0.2532	MAS1_P469_R	0.9790	0.9823	0.9784	0.9922	0.5757	0.9802
0.0059	0.0041	MATK_P64_F	0.0073	0.0094	0.0024	0.0511	0.0840	0.0085
0.0000	0.0000	MC2R_P1025_F	0.9401	0.9269	0.9333	0.0000	0.0008	0.0000
0.0133	0.0059	MCAM_P169_R	0.0030	0.4371	0.0692	0.0000	0.0000	0.0082
0.0848	0.0402	MCAM_P265_R	0.3184	0.0235	0.4392	0.0062	0.0061	0.5024
0.0000	0.0000	MCC_P196_R	0.0000	0.0000	0.0000	0.0000	0.0000	0.0000
0.0087	0.0144	MCM2_P241_R	0.0065	0.0093	0.0185	0.0087	0.1996	0.0077
0.0058	0.0765	MDR1_seq_42_S300_R	0.0036	0.0084	0.0000	0.0080	0.0704	0.0041
0.0100	0.0000	MDS1_E45_F	0.0056	0.0142	0.0051	0.0234	0.0030	0.0171
0.0087	0.0043	MECP2_E90_R	0.0788	0.0107	0.1204	0.0066	0.0046	0.0061
0.0001	0.0000	MEG3_E91_F	0.3689	0.7260	0.0788	0.0005	0.0000	0.0000
0.0026	0.0000	MEST_E150_F	0.0009	0.0008	0.0000	0.0000	0.0000	0.0000
0.0051	0.0000	MEST_P4_F	0.0013	0.0063	0.0739	0.0024	0.0000	0.0021
0.0000	0.0005	MEST_P62_R	0.0000	0.0027	0.0597	0.0001	0.0000	0.1202
0.0237	0.0086	MET_E333_F	0.6221	0.8289	0.8306	0.5111	0.9907	0.8484
0.6168	0.0000	MFAP4_P197_F	0.0042	0.0052	0.2124	0.0000	0.0818	0.3562
0.0000	0.0080	MGMT_P272_R	0.0000	0.0042	0.0200	0.0000	0.0000	0.0016
0.0144	0.5040	MGMT_P281_F	0.2841	0.2072	0.0464	0.0097	0.4282	0.5212
0.9696	0.9922	MKRN3_P108_F	0.9790	0.9718	0.9698	0.0000	0.0000	0.9750
0.0253	0.0000	MLF1_P97_F	0.2456	0.4475	0.1286	0.0071	0.0039	0.5152
0.0085	0.0037	MLH1_P381_F	0.0060	0.0085	0.0013	0.0028	0.0042	0.0103
0.0017	0.0037	MLH3_E72_F	0.0000	0.0038	0.0000	0.0000	0.0067	0.0048
0.3019	0.0017	MLH3_P25_F	0.0156	0.0631	0.0514	0.0033	0.0000	0.1014
0.0000	0.0000	MLLT3_E93_R	0.0000	0.0029	0.0000	0.0033	0.0000	0.0000
0.4403	0.1738	MLLT4_P1400_F	0.0154	0.0418	0.0134	0.0145	0.0105	0.0188
0.0044	0.0020	MLLT6_P957_F	0.0031	0.0031	0.0000	0.0000	0.0034	0.0022
0.0000	0.0000	MME_E29_F	0.0008	0.5611	0.0000	0.0048	0.0000	0.0041
0.0000	0.0000	MME_P388_F	0.0000	0.0000	0.0354	0.0000	0.0061	0.0000

0.7000	0.9911	MMP19_E274_R	0.9819	0.9824	0.9834	0.0000	0.0000	0.9804
0.0050	0.0000	MMP2_E21_R	0.2699	0.0045	0.0591	0.0047	0.0027	0.0019
0.0064	0.0042	MMP2_P197_F	0.0060	0.0093	0.0000	0.0063	0.0000	0.2468
0.0085	0.0249	MMP2_P303_R	0.0090	0.0110	0.0032	0.0070	0.0019	0.0099
0.9818	0.8259	MMP3_P16_R	0.8826	0.9659	0.9785	0.0000	0.9906	0.9808
0.7618	0.1181	MMP7_P613_F	0.7476	0.6949	0.8300	0.3319	0.9930	0.9106
0.0102	0.0507	MMP9_P189_F	0.0137	0.0163	0.2693	0.0581	0.0053	0.6789
0.0173	0.0051	MMP9_P237_R	0.0111	0.0116	0.0058	0.0093	0.0582	0.0169
0.0076	0.0051	MOS_E60_R	0.2862	0.0081	0.0000	0.0050	0.0206	0.0082
0.0043	0.0046	MPO_P883_R	0.1772	0.0041	0.1433	0.0127	0.0034	0.0047
0.9806	0.9899	MSH3_E3_F	0.9323	0.9812	0.9424	0.9847	0.0000	0.9878
0.4870	0.4789	MSH3_P13_R	0.7186	0.7285	0.4898	0.0098	0.3794	0.7004
0.0000	0.0000	MST1R_P392_F	0.0000	0.0000	0.0000	0.0000	0.0000	0.0000
0.5263	0.0089	MT1A_E13_R	0.0109	0.0112	0.1562	0.0844	0.1466	0.0631
0.0981	0.0002	MT1A_P49_R	0.0000	0.0000	0.0105	0.0000	0.0000	0.0000
0.0154	0.0078	MTA1_P478_F	0.0319	0.3303	0.1079	0.0098	0.6374	0.2932
0.8995	0.0000	MUC1_E18_R	0.8339	0.0000	0.8933	0.0000	0.0000	0.0000
0.0102	0.0000	MUC1_P191_F	0.0047	0.2642	0.0000	0.0059	0.0000	0.0053
0.0026	0.0000	MXI1_P75_R	0.0048	0.0065	0.0000	0.0057	0.6039	0.0054
0.4052	0.0071	MYBL2_P211_F	0.0078	0.0140	0.0000	0.0000	0.0000	0.2650
0.0000	0.0000	MYCL1_P502_R	0.0040	0.0019	0.0000	0.0000	0.0031	0.3021
0.0212	0.0118	MYCN_E77_R	0.0127	0.0248	0.0694	0.0068	0.0012	0.0140
0.0058	0.0046	MYCN_P464_R	0.0024	0.0092	0.0000	0.0044	0.0000	0.0035
0.4833	0.0098	MYH11_P22_F	0.0024	0.0080	0.0000	0.0024	0.0000	0.0049
0.0000	0.0000	MYH11_P236_R	0.0000	0.0000	0.0000	0.0000	0.0000	0.0000
0.2044	0.3731	MYLK_P469_R	0.0077	0.0120	0.1295	0.0149	0.3936	0.0116
0.0031	0.0063	MYOD1_E156_F	0.0030	0.0049	0.0303	0.1186	0.0000	0.0020
0.0000	0.0000	MYOD1_P50_F	0.6050	0.0000	0.0713	0.0000	0.0000	0.0000
0.9620	0.9948	NBL1_E205_R	0.9796	0.0310	0.9753	0.0752	0.9940	0.9788
0.8651	0.0000	NBL1_P24_F	0.7201	0.9803	0.9606	0.0000	0.0000	0.9743
0.0055	0.0029	NCL_P840_R	0.0081	0.0150	0.0073	0.0000	0.0010	0.0148
0.0000	0.8800	NDN_P1110_F	0.9721	0.9750	0.9742	0.9888	0.0000	0.9712
0.5416	0.9931	NEFL_E23_R	0.4420	0.6749	0.2338	0.8563	0.8841	0.9697

0.0000	0.0000	NEFL_P209_R	0.0000	0.0000	0.0000	0.0000	0.0000	0.0000
0.0000	0.0009	NEO1_P1067_F	0.0000	0.0000	0.0000	0.0000	0.0000	0.0000
0.0000	0.0000	NES_P239_R	0.0000	0.0044	0.1977	0.0086	0.0032	0.0000
0.0000	0.0055	NEU1_P745_F	0.0025	0.0061	0.0000	0.0093	0.0000	0.0032
0.0000	0.0058	NFKB1_P336_R	0.0000	0.1194	0.0000	0.0173	0.0000	0.4579
0.0227	0.1638	NFKB2_P709_R	0.0231	0.0291	0.0216	0.3018	0.0057	0.0302
0.0000	0.0000	NGFB_E353_F	0.6967	0.4198	0.0633	0.0000	0.4153	0.0004
0.1387	0.0371	NGFB_P13_F	0.1266	0.1795	0.1708	0.1720	0.3173	0.0634
0.8791	0.0035	NGFR_E328_F	0.0037	0.0051	0.0936	0.0055	0.0000	0.0023
0.7311	0.3387	NGFR_P355_F	0.3422	0.0577	0.0435	0.0489	0.0525	0.6715
0.0099	0.0000	NKX3-1_P146_F	0.0077	0.0098	0.0331	0.0061	0.0055	0.0112
0.9871	0.0000	NKX3-1_P871_R	0.9814	0.9802	0.9802	0.0000	0.0000	0.9822
0.0000	0.0000	NOTCH1_E452_R	0.0000	0.0000	0.0000	0.0000	0.0000	0.0000
0.0000	0.0005	NOTCH1_P1198_F	0.0000	0.0000	0.0099	0.0000	0.0002	0.0000
0.0031	0.0041	NOTCH2_P312_R	0.0297	0.0051	0.0000	0.0075	0.0037	0.0029
0.0000	0.0000	NOTCH3_E403_F	0.0000	0.0013	0.0317	0.0000	0.0015	0.0000
0.9570	0.2878	NPR2_P618_F	0.8144	0.0989	0.2129	0.0050	0.5803	0.0027
0.0304	0.4597	NPY_P295_F	0.0189	0.0327	0.0407	0.0262	0.8174	0.0291
0.0176	0.1125	NPY_P91_F	0.0148	0.0191	0.0134	0.0145	0.1746	0.0158
0.0059	0.0000	NQO1_E74_R	0.0049	0.0067	0.0000	0.0000	0.0000	0.0079
0.0009	0.0000	NQO1_P345_R	0.0006	0.0991	0.0000	0.0021	0.0000	0.0000
0.0096	0.0070	NR2F6_E375_R	0.0101	0.0133	0.0605	0.0110	0.2116	0.0284
0.0000	0.0067	NRAS_P103_R	0.0000	0.0018	0.0000	0.0035	0.0036	0.0000
0.0000	0.0000	NRAS_P12_R	0.0000	0.0000	0.0000	0.0000	0.0000	0.0000
0.0004	0.0000	NRG1_E74_F	0.0019	0.0033	0.0000	0.0003	0.0022	0.0006
0.0071	0.0117	NRG1_P558_R	0.0024	0.0064	0.0089	0.0054	0.1009	0.1279
0.0045	0.0026	NTRK2_P10_F	0.0086	0.0086	0.0404	0.1859	0.0000	0.0055
0.0000	0.0080	NTRK2_P395_R	0.0134	0.0030	0.0000	0.0000	0.0000	0.4802
0.0000	0.0038	NTRK3_E131_F	0.0000	0.0045	0.0338	0.0001	0.0000	0.0000
0.0000	0.0000	NTRK3_P636_R	0.0000	0.0000	0.0000	0.0000	0.0000	0.0000
0.0000	0.0000	NTRK3_P752_F	0.0000	0.0005	0.0000	0.0000	0.0043	0.0000
0.0077	0.0082	NTSR1_E109_F	0.0056	0.0102	0.0000	0.0043	0.0029	0.0096
0.0215	0.0000	NTSR1_P318_F	0.0094	0.0183	0.0050	0.0150	0.0000	0.0157

0.0108	0.3068	OAT_P465_F	0.2042	0.1874	0.2046	0.0082	0.4080	0.0591
0.0019	0.0007	ODC1_P424_F	0.0000	0.0034	0.0000	0.0000	0.0000	0.0000
0.0577	0.7107	ONECUT2_E96_F	0.4811	0.0343	0.0146	0.0121	0.0000	0.0284
0.0000	0.0000	ONECUT2_P315_R	0.0000	0.0000	0.0000	0.0000	0.0026	0.0000
0.0043	0.0026	OPCML_E219_R	0.0043	0.0059	0.0000	0.0494	0.0024	0.0039
0.0078	0.0021	OPCML_P71_F	0.4408	0.0837	0.2167	0.0000	0.0000	0.0057
0.0147	0.0052	OSM_P188_F	0.0126	0.0811	0.0053	0.0069	0.0051	0.0135
0.0127	0.0487	p16_seq_47_S188_R	0.0049	0.0068	0.0000	0.0043	0.0149	0.0036
0.1830	0.0032	P2RX7_E323_R	0.1300	0.4748	0.0248	0.0044	0.0040	0.0057
0.0292	0.1831	P2RX7_P119_R	0.0220	0.0373	0.0829	0.0123	0.1495	0.0342
0.8270	0.1908	P2RX7_P597_F	0.9493	0.9842	0.9861	0.9903	0.5246	0.9884
0.0254	0.4441	PALM2-AKAP2_P183_R	0.0248	0.1177	0.0559	0.0266	0.0197	0.0250
0.0026	0.0067	PALM2-AKAP2_P420_R	0.0047	0.0061	0.0575	0.0063	0.0000	0.0030
0.8666	0.2583	PARP1_P610_R	0.9765	0.6450	0.6799	0.0045	0.0060	0.9383
0.0116	0.0000	PAX6_P1121_F	0.0083	0.0152	0.0009	0.0125	0.0156	0.0115
0.0000	0.0038	PAX6_P50_R	0.0000	0.0000	0.0000	0.0000	0.0000	0.0000
0.0114	0.0055	PCDH1_E22_F	0.0137	0.0161	0.0039	0.0032	0.0000	0.0144
0.0113	0.1054	PCGF4_P760_R	0.2012	0.2129	0.0636	0.0073	0.0049	0.0123
0.0027	0.0041	PCGF4_P92_R	0.0112	0.0179	0.0000	0.0106	0.0213	0.0156
0.7370	0.8799	PCTK1_E77_R	0.7566	0.8929	0.6113	0.9789	0.8609	0.9030
0.0220	0.0117	PDE1B_E141_F	0.0148	0.2550	0.0071	0.3157	0.0077	0.0151
0.0101	0.0046	PDE1B_P263_R	0.1956	0.0120	0.0258	0.0065	0.0117	0.2072
0.0006	0.0000	PDGFA_P841_R	0.1651	0.0011	0.0000	0.0000	0.0000	0.0021
0.0088	0.0035	PDGFRB_E195_R	0.0070	0.0073	0.0813	0.0060	0.0000	0.0061
0.0017	0.0026	PDGFRB_P343_F	0.0007	0.0015	0.0000	0.0052	0.0015	0.0003
0.0026	0.0000	PEG10_P978_R	0.5129	0.1284	0.1807	0.0000	0.0029	0.0640
0.1611	0.0267	PENK_E26_F	0.0736	0.1726	0.1291	0.0464	0.0176	0.1889
0.0051	0.0000	PENK_P447_R	0.8922	0.0064	0.1102	0.0240	0.0910	0.0016
0.0212	0.0092	PGF_E33_F	0.0143	0.0135	0.0055	0.0110	0.2949	0.0161
0.0108	0.0040	PHLDA2_P622_F	0.0045	0.1582	0.1698	0.0045	0.0012	0.2695
0.2847	0.4845	PI3_P1394_R	0.0463	0.4628	0.8803	0.4780	0.3908	0.8435
0.0182	0.1226	PITX2_E24_R	0.0124	0.0199	0.1903	0.0126	0.3474	0.0128
0.0000	0.0016	PITX2_P183_R	0.3832	0.0000	0.0578	0.0029	0.0000	0.5006

0.0130	0.1550	PKD2_P287_R	0.0078	0.0095	0.0000	0.0039	0.0000	0.0107
0.0112	0.0081	PKD2_P336_R	0.0089	0.1056	0.0472	0.8382	0.0025	0.0287
0.0187	0.4108	PLAU_P11_F	0.3618	0.0264	0.0397	0.0115	0.6570	0.0442
0.0064	0.0003	PLAU_P176_R	0.0018	0.0100	0.2305	0.0000	0.9914	0.9315
0.0025	0.0019	PLAUR_E123_F	0.0041	0.0068	0.0160	0.0028	0.0016	0.0032
0.0009	0.0000	PLAUR_P82_F	0.0000	0.0016	0.0000	0.0025	0.0000	0.0000
0.9235	0.9919	PLG_E406_F	0.9894	0.9702	0.9878	0.9926	0.9935	0.9911
0.1276	0.0672	PLSCR3_P751_R	0.1544	0.1776	0.1941	0.0402	0.0059	0.1003
0.0109	0.0032	PLXDC1_P236_F	0.0121	0.0152	0.0038	0.1347	0.1758	0.0130
0.0144	0.0009	PLXDC2_E337_F	0.0034	0.0074	0.0000	0.0070	0.0055	0.0033
0.0381	0.3756	PLXDC2_P914_R	0.0271	0.0386	0.0247	0.0447	0.0145	0.5267
0.9821	0.0000	PMP22_P975_F	0.9810	0.9742	0.9736	0.9125	0.9915	0.9849
0.1089	0.1256	PODXL_P1341_R	0.0040	0.0096	0.0196	0.0071	0.0033	0.0067
0.0148	0.5153	POMC_E254_F	0.0107	0.0778	0.0066	0.0130	0.0000	0.0134
0.0033	0.0025	POMC_P400_R	0.0085	0.0072	0.0037	0.0000	0.0058	0.0041
0.0089	0.1817	POMC_P53_F	0.0084	0.0107	0.0033	0.0060	0.0077	0.0114
0.0130	0.0000	PPARD_P846_F	0.0025	0.0063	0.3255	0.0000	0.0000	0.7231
0.0000	0.0000	PPARG_E178_R	0.0033	0.0063	0.0000	0.0174	0.0000	0.0005
0.0068	0.0000	PPP2R1B_P268_R	0.0020	0.0062	0.0000	0.0040	0.0000	0.0028
0.9773	0.9915	PRDM2_P1340_R	0.9682	0.9510	0.9662	0.0000	0.5776	0.9586
0.0010	0.0000	PRKCDBP_E206_F	0.0005	0.0010	0.0000	0.0000	0.0000	0.2892
0.0039	0.0000	PROK2_E0_F	0.0034	0.0072	0.0000	0.0000	0.0000	0.0061
0.0000	0.0000	PROK2_P390_F	0.0000	0.0015	0.0000	0.0000	0.0000	0.0000
0.9861	0.9923	PRSS8_E134_R	0.9859	0.9850	0.9818	0.0095	0.9927	0.9850
0.0000	0.0000	PSCA_E359_F	0.6443	0.0022	0.1191	0.0000	0.0000	0.0002
0.0023	0.0060	PSIP1_P163_R	0.0028	0.0270	0.0000	0.0000	0.0034	0.0030
0.0142	0.0000	PTCH_E42_F	0.0168	0.0131	0.0049	0.2019	0.0000	0.0164
0.3562	0.6050	PTCH2_P568_R	0.1651	0.6725	0.4021	0.0077	0.0291	0.4873
0.0084	0.0054	PTEN_P438_F	0.0059	0.0062	0.0000	0.0000	0.0046	0.0039
0.0068	0.0081	PTGS1_P2_F	0.0081	0.0097	0.0492	0.0000	0.0000	0.0080
0.0042	0.4318	PTGS2_P308_F	0.0022	0.0060	0.0000	0.0063	0.0099	0.0025
0.0074	0.0000	PTGS2_P524_R	0.0052	0.0116	0.0000	0.0000	0.0000	0.0081
0.0000	0.0000	PTHLH_P15_R	0.0000	0.2240	0.3811	0.0000	0.0000	0.6465

0.0509	0.0759	PTHR1_P170_R	0.9739	0.9709	0.9745	0.9903	0.9927	0.9782
0.0186	0.0108	PTK2_P735_R	0.0082	0.0090	0.0007	0.1391	0.2824	0.0098
0.0086	0.0005	PTK2B_P673_R	0.0049	0.0078	0.0287	0.0000	0.0027	0.0024
0.0000	0.0000	PTPN6_E171_R	0.0000	0.0000	0.0000	0.0030	0.0000	0.0000
0.0000	0.0000	PTPN6_P282_R	0.0000	0.0000	0.0851	0.0000	0.0000	0.4359
0.0000	0.0000	PTPNS1_E433_R	0.0000	0.0000	0.0000	0.0000	0.0016	0.0000
0.0110	0.0029	PTPNS1_P301_R	0.0110	0.0144	0.0027	0.0045	0.0000	0.0103
0.2648	0.0000	PTPRF_E178_R	0.0025	0.1533	0.1879	0.0000	0.0009	0.2521
0.0085	0.3025	PTPRG_E40_R	0.0242	0.0256	0.0639	0.0064	0.4719	0.0060
0.0073	0.0000	PTPRG_P476_F	0.0053	0.0074	0.0000	0.0046	0.0064	0.0083
0.0000	0.0031	PTPRO_E56_F	0.0000	0.0021	0.0000	0.0000	0.0000	0.0000
0.0000	0.0000	PURA_P928_R	0.0000	0.0000	0.0000	0.0000	0.0000	0.0000
0.9772	0.0000	PWCR1_P811_F	0.9722	0.9586	0.9826	0.0000	0.0000	0.9738
0.0205	0.0077	PYCARD_E87_F	0.0150	0.0195	0.0069	0.0118	0.3034	0.0152
0.3209	0.0355	PYCARD_P150_F	0.0055	0.6695	0.6008	0.0065	0.0067	0.5553
0.0014	0.0000	RAB32_E314_R	0.0000	0.0064	0.0000	0.0000	0.0000	0.3968
0.0000	0.0000	RAB32_P493_R	0.0000	0.0000	0.0000	0.0000	0.0000	0.0000
0.0047	0.0000	RAF1_P330_F	0.2327	0.2062	0.0119	0.0000	0.0000	0.0000
0.6286	0.9921	RAN_P581_R	0.9742	0.8145	0.8943	0.0000	0.0000	0.0949
0.0094	0.0026	RARA_E128_R	0.2189	0.3360	0.2147	0.0038	0.0030	0.2964
0.4792	0.0000	RARA_P176_R	0.0034	0.0026	0.1601	0.0000	0.0011	0.0000
0.0019	0.0000	RARB_E114_F	0.0013	0.0060	0.0067	0.0032	0.0000	0.0047
0.0000	0.0000	RARB_P60_F	0.0000	0.0016	0.2649	0.0000	0.0000	0.0000
0.0109	0.0135	RARRES1_E235_F	0.0056	0.0205	0.0293	0.0074	0.0157	0.0614
0.0128	0.0919	RASGRF1_E16_F	0.0049	0.0089	0.0000	0.0000	0.1459	0.0061
0.0065	0.0073	RASGRF1_P768_F	0.0023	0.0057	0.0278	0.0032	0.0493	0.0058
0.0034	0.0000	RASSF1_E116_F	0.3208	0.0066	0.0334	0.0000	0.0000	0.0000
0.0046	0.3110	RASSF1_P244_F	0.0051	0.0102	0.0000	0.0000	0.1667	0.0094
0.0161	0.1674	RBL2_P250_R	0.6326	0.0101	0.0542	0.0030	0.2152	0.0075
0.0020	0.0018	RBP1_E158_F	0.0016	0.0029	0.0000	0.0002	0.0000	0.0000
0.0000	0.0000	RBP1_P150_F	0.0000	0.0000	0.0000	0.0000	0.0000	0.0000
0.0051	0.4852	RET_P717_F	0.0084	0.0094	0.0004	0.0120	0.0000	0.0614
0.0056	0.0000	RET_seq_53_S374_F	0.0088	0.0068	0.0000	0.2643	0.1780	0.0070

0.0000	0.8706	RHOC_P536_F	0.0000	0.0018	0.0000	0.0000	0.0000	0.0000
0.0000	0.0000	RHOH_P121_F	0.0000	0.0000	0.0000	0.0000	0.0000	0.0000
0.9766	0.0430	RHOH_P953_R	0.9482	0.8948	0.9693	0.0000	0.9917	0.9810
0.0470	0.4046	RIPK1_P868_F	0.9725	0.9734	0.9749	0.6051	0.5608	0.9778
0.0071	0.0857	RIPK2_E123_F	0.0007	0.3399	0.0000	0.0266	0.0488	0.1359
0.0061	0.1774	RIPK3_P124_F	0.0076	0.0590	0.0012	0.0095	0.0042	0.0106
0.0000	0.0000	RIPK3_P24_F	0.0000	0.0000	0.0000	0.0000	0.0044	0.0000
0.0018	0.0000	RIPK4_E166_F	0.3537	0.0044	0.0896	0.0000	0.0000	0.0006
0.3543	0.0058	RIPK4_P172_F	0.0076	0.1940	0.0836	0.0095	0.0076	0.1845
0.0059	0.0077	ROR1_P6_F	0.0000	0.0000	0.0000	0.0000	0.0000	0.0027
0.0000	0.0046	ROR2_P317_R	0.0008	0.0053	0.0255	0.0007	0.0000	0.0000
0.0000	0.0000	RRAS_P100_R	0.0000	0.0000	0.0000	0.0019	0.0011	0.0000
0.9175	0.0000	RUNX3_P247_F	0.7309	0.9647	0.9320	0.0047	0.0000	0.9010
0.8062	0.0000	RYK_P493_F	0.0000	0.0000	0.0000	0.0000	0.0000	0.0000
0.2481	0.0000	S100A2_E36_R	0.4363	0.0103	0.2732	0.0000	0.0000	0.3804
0.0093	0.0057	S100A4_E315_F	0.0083	0.1592	0.0734	0.0102	0.1098	0.0095
0.0000	0.0000	SCGB3A1_E55_R	0.1813	0.0016	0.0000	0.0000	0.0000	0.0000
0.0017	0.0000	SCGB3A1_P103_R	0.0108	0.0000	0.0000	0.0000	0.0000	0.0000
0.0024	0.0000	SEMA3A_P343_F	0.0011	0.0036	0.0000	0.0000	0.0000	0.0014
0.8322	0.0787	SEMA3A_P658_R	0.7753	0.0411	0.2263	0.0812	0.0616	0.3927
0.2381	0.0141	SEMA3C_E49_R	0.0163	0.0134	0.0276	0.1597	0.3523	0.0155
0.0287	0.0533	SEMA3C_P642_F	0.0075	0.0260	0.1006	0.0171	0.0036	0.0106
0.0040	0.0061	SEMA3F_E333_R	0.0052	0.2594	0.0000	0.0037	0.0000	0.2795
0.4528	0.0000	SEMA3F_P692_R	0.6032	0.4335	0.1798	0.0228	0.0000	0.0044
0.3242	0.0090	SEPT5_P441_F	0.0079	0.0100	0.5195	0.0727	0.0064	0.1799
0.8404	0.0111	SEPT9_P58_R	0.1228	0.6269	0.8117	0.9898	0.4664	0.8510
0.8963	0.1962	SERPINA5_E69_F	0.8350	0.9395	0.7597	0.4202	0.3375	0.9627
0.9875	0.0028	SERPINB2_P939_F	0.9864	0.9819	0.9784	0.9844	0.9930	0.9840
0.1261	0.0078	SERPINE1_E189_R	0.0125	0.1451	0.1182	0.0120	0.5427	0.2783
0.0043	0.0000	SEZ6L_P249_F	0.0038	0.0056	0.0404	0.0058	0.0025	0.0040
0.0054	0.0000	SEZ6L_P299_F	0.2988	0.0047	0.0000	0.0035	0.0000	0.0002
0.5958	0.0215	SFN_E118_F	0.9840	0.9462	0.9817	0.9930	0.7161	0.9824
0.0024	0.0000	SFRP1_E398_R	0.0000	0.0035	0.0000	0.0000	0.0017	0.0024

0.0048	0.0024	SFRP1_P157_F	0.0058	0.0070	0.0000	0.0031	0.0068	0.0039
0.9717	0.0000	SFTPA1_E340_R	0.9821	0.9859	0.9753	0.0000	0.0000	0.9785
0.0013	0.0053	SH3BP2_P771_R	0.0031	0.2606	0.1408	0.0000	0.0000	0.0066
0.0028	0.0000	SHB_P473_R	0.0018	0.0048	0.0000	0.0050	0.2542	0.0015
0.0206	0.0087	SHB_P691_R	0.0114	0.0136	0.0058	0.2406	0.0042	0.0144
0.0000	0.0000	SHH_E328_F	0.0000	0.4757	0.2238	0.0000	0.0000	0.0000
0.6312	0.0075	SHH_P104_R	0.0057	0.0123	0.0916	0.0091	0.0108	0.0089
0.9543	0.0000	SIN3B_P514_R	0.9769	0.9825	0.9749	0.0000	0.0000	0.9698
0.0077	0.0045	SKI_E465_R	0.0096	0.0097	0.0000	0.0090	0.0000	0.0070
0.9864	0.7514	SLC22A2_E271_R	0.9764	0.9827	0.9809	0.9886	0.9929	0.9848
0.0167	0.6092	SLC22A3_P634_F	0.6323	0.0176	0.5043	0.0036	0.3817	0.4859
0.7740	0.0000	SLC6A8_seq_28_S227_F	0.0000	0.0000	0.6648	0.0000	0.0000	0.0000
0.0073	0.3540	SLIT2_P208_F	0.0079	0.0110	0.0477	0.0099	0.1583	0.0055
0.0000	0.0000	SMAD2_P708_R	0.0065	0.0053	0.0000	0.0000	0.0025	0.0051
0.0189	0.4836	SMAD2_P848_R	0.0048	0.0034	0.1301	0.0000	0.0008	0.4221
0.0016	0.0000	SMAD4_P474_R	0.0000	0.0000	0.0000	0.0000	0.1103	0.0000
0.0042	0.0038	SMARCA3_E20_F	0.0021	0.0290	0.0109	0.0017	0.0033	0.0027
0.0173	0.0000	SMARCA3_P109_R	0.0172	0.0201	0.0744	0.0077	0.0067	0.1949
0.0109	0.0000	SMARCA3_P17_R	0.0040	0.3545	0.3718	0.0000	0.0122	0.3262
0.0054	0.0071	SMARCA4_P362_R	0.0048	0.0107	0.0008	0.0053	0.0033	0.0065
0.0009	0.0000	SMO_E57_F	0.0069	0.0138	0.0539	0.0021	0.0000	0.0080
0.0118	0.2910	SOD3_P225_F	0.9440	0.9694	0.9620	0.9908	0.0000	0.9697
0.0055	0.0000	SOX1_P1018_R	0.0013	0.0054	0.0000	0.0000	0.1106	0.0009
0.0151	0.0092	SOX1_P294_F	0.0148	0.0149	0.0912	0.5684	0.4662	0.0130
0.0000	0.0000	SOX17_P287_R	0.0000	0.5560	0.0071	0.0000	0.0000	0.1813
0.0808	0.7184	SOX17_P303_F	0.0938	0.0802	0.0736	0.1243	0.2979	0.0955
0.0089	0.0000	SOX2_P546_F	0.0086	0.2391	0.0028	0.0059	0.0575	0.0097
0.0109	0.0846	SPARC_E50_R	0.1423	0.1408	0.1092	0.0055	0.0034	0.2261
0.0048	0.0017	SPARC_P195_F	0.0672	0.0068	0.0668	0.0064	0.0035	0.0054
0.3304	0.1790	SPDEF_E116_R	0.0240	0.0552	0.5977	0.1563	0.6074	0.9426
0.0179	0.9908	SPI1_E205_F	0.6634	0.2675	0.2652	0.0174	0.5017	0.0240
0.0047	0.0040	SPP1_E140_R	0.1587	0.1118	0.1064	0.0000	0.0089	0.0055
0.9825	0.9943	SRC_P297_F	0.9828	0.9787	0.9804	0.2786	0.9929	0.9812

0.0745	0.0000	ST6GAL1_P164_R	0.0115	0.0718	0.0000	0.0281	0.0000	0.0258
0.0053	0.0054	ST6GAL1_P528_F	0.0031	0.0069	0.0375	0.0034	0.0000	0.0041
0.9092	0.0042	STK23_E182_R	0.9792	0.2693	0.9820	0.7812	0.9924	0.2951
0.0144	0.1365	SYK_E372_F	0.0121	0.0089	0.0319	0.0117	0.0717	0.0182
0.0089	0.5180	TAL1_E122_F	0.0054	0.0094	0.0000	0.4529	0.2831	0.0120
0.0012	0.0068	TAL1_P594_F	0.0000	0.0017	0.1112	0.0000	0.0000	0.0000
0.0000	0.0000	TAL1_P817_F	0.9084	0.7449	0.0000	0.0000	0.0000	0.9083
0.0000	0.0000	TCF4_P175_R	0.0000	0.0000	0.2406	0.0000	0.0000	0.0000
0.0059	0.0000	TCF7L2_E411_F	0.0013	0.0043	0.0000	0.0014	0.0014	0.0000
0.0368	0.0000	TCF7L2_P193_R	0.0035	0.0047	0.0000	0.0011	0.0000	0.0021
0.0104	0.0066	TERT_E20_F	0.0114	0.0139	0.0322	0.0066	0.0062	0.0128
0.4223	0.0137	TERT_P360_R	0.3138	0.3630	0.1623	0.0157	0.1279	0.0200
0.0000	0.0000	TES_E172_F	0.0009	0.0031	0.4270	0.0000	0.0036	0.8092
0.0049	0.0170	TESK2_P252_R	0.0058	0.0100	0.0015	0.9789	0.2889	0.0408
0.0000	0.0029	TFAP2C_E260_F	0.0000	0.1355	0.0000	0.0000	0.0000	0.0000
0.0079	0.0034	TFAP2C_P765_F	0.0065	0.0095	0.0383	0.0000	0.0037	0.0117
0.9852	0.8276	TFF2_P178_F	0.9596	0.7853	0.9770	0.9785	0.9945	0.9814
0.8819	0.0762	TFF2_P557_R	0.9691	0.9863	0.9857	0.9891	0.9942	0.9851
0.0000	0.0000	TFPI2_E141_F	0.0000	0.0018	0.0000	0.0000	0.0000	0.0000
0.0146	0.0615	TFPI2_P152_R	0.0127	0.0313	0.0384	0.0086	0.2265	0.0366
0.0099	0.5209	TFPI2_P9_F	0.0139	0.0165	0.0057	0.0226	0.3022	0.0165
0.0010	0.0016	TFRC_P414_R	0.0131	0.0140	0.0000	0.0000	0.1310	0.0110
0.8520	0.5195	TGFA_P558_F	0.0077	0.0110	0.0021	0.0971	0.0000	0.0093
0.0040	0.0000	TGFA_P642_R	0.0029	0.0128	0.0000	0.0896	0.0011	0.0052
0.9886	0.0000	TGFB1_P833_R	0.8855	0.9763	0.9795	0.0000	0.0000	0.8139
0.0000	0.0000	TGFB2_E226_R	0.0001	0.0050	0.0000	0.0000	0.0000	0.0000
0.0117	0.0068	TGFB2_P632_F	0.0150	0.0160	0.0385	0.0093	0.0062	0.0133
0.3694	0.9926	TGFB3_E58_R	0.4138	0.9851	0.9851	0.9902	0.9924	0.9852
0.0146	0.1285	TGFBI_P173_F	0.0132	0.0189	0.2103	0.0130	0.0711	0.2712
0.0066	0.8131	TGFBI_P31_R	0.0070	0.0789	0.0798	0.0051	0.0486	0.1247
0.3007	0.0031	TGFBR3_E188_R	0.0031	0.0030	0.0000	0.0013	0.0056	0.0019
0.0016	0.0000	TGFBR3_P429_F	0.0000	0.0032	0.0000	0.0000	0.0000	0.0000
0.0021	0.0000	THBS1_E207_R	0.0032	0.0043	0.0000	0.0000	0.0000	0.0033

0.0175	0.0000	THBS1_P500_F	0.1168	0.1512	0.0350	0.0045	0.0051	0.0215
0.0058	0.0185	THBS2_E129_F	0.0033	0.0032	0.0000	0.0000	0.0019	0.0022
0.0216	0.0356	THY1_P149_R	0.0280	0.0387	0.0641	0.0138	0.5761	0.0293
0.0010	0.0000	TIAM1_P188_R	0.0000	0.0032	0.0156	0.0020	0.0000	0.0000
0.2306	0.0514	TIMP1_E254_R	0.0604	0.4389	0.3434	0.0504	0.0216	0.0284
0.9728	0.9892	TIMP1_P615_R	0.9398	0.6805	0.9675	0.9828	0.0000	0.9443
0.0063	0.0128	TIMP2_E394_R	0.0059	0.0083	0.0114	0.0000	0.0000	0.0072
0.0026	0.0000	TIMP2_P267_F	0.0010	0.0000	0.0232	0.0053	0.0029	0.0011
0.9899	0.2528	TIMP3_P1114_R	0.9805	0.9875	0.9856	0.9935	0.9917	0.9863
0.9883	0.9935	TIMP3_P690_R	0.9889	0.9881	0.9878	0.0000	0.9945	0.9872
0.4361	0.0031	TIMP3_seq_7_S38_F	0.0090	0.0098	0.0013	0.0566	0.0001	0.7899
0.0122	0.0272	TJP1_P326_R	0.0068	0.0138	0.0060	0.0105	0.0086	0.0135
0.0072	0.0060	TJP1_P390_F	0.0043	0.0080	0.0000	0.0046	0.0000	0.0062
0.0862	0.3824	TK1_E47_F	0.1382	0.0238	0.4083	0.0000	0.0046	0.0317
0.0057	0.0016	TK1_P62_R	0.0086	0.0076	0.0269	0.0092	0.4099	0.0074
0.0091	0.0000	TMEFF1_E180_R	0.0003	0.0084	0.0000	0.0000	0.0000	0.0068
0.2673	0.0000	TMEFF1_P234_F	0.0000	0.0038	0.0409	0.0000	0.0000	0.0005
0.0541	0.0311	TMEFF2_P152_R	0.0865	0.0630	0.1616	0.2966	0.0237	0.3919
0.0000	0.0000	TMEFF2_P210_R	0.0093	0.0000	0.2289	0.0000	0.0000	0.0000
0.0000	0.0000	TMEM63A_E63_F	0.0000	0.0000	0.0000	0.0000	0.0000	0.0000
0.9829	0.9933	TMPRSS4_E83_F	0.9778	0.9882	0.9837	0.0483	0.9925	0.9855
0.7266	0.0549	TMPRSS4_P552_F	0.8069	0.9762	0.8390	0.0273	0.4753	0.8968
0.0001	0.0000	TNC_P198_F	0.0004	0.3998	0.3396	0.5693	0.0000	0.0000
0.0073	0.0115	TNC_P57_F	0.0039	0.0068	0.0315	0.0103	0.0084	0.2856
0.9888	0.9962	TNF_P1084_F	0.9859	0.9889	0.9863	0.9939	0.0089	0.9813
0.0000	0.0000	TNF_P158_F	0.1320	0.0035	0.0000	0.0000	0.0000	0.0000
0.0062	0.0052	TNFRSF10A_P171_F	0.0064	0.0112	0.0024	0.0075	0.0085	0.0101
0.0042	0.0020	TNFRSF10A_P91_F	0.0007	0.0029	0.0000	0.0000	0.0100	0.0041
0.0253	0.0115	TNFRSF10B_P108_R	0.0282	0.0264	0.0209	0.0172	0.0076	0.0462
0.0045	0.1018	TNFRSF10C_E109_F	0.0032	0.0040	0.0000	0.0040	0.0033	0.0042
0.0109	0.0094	TNFRSF10D_E27_F	0.0079	0.0088	0.0459	0.0037	0.0044	0.0088
0.0218	0.1659	TNFRSF10D_P70_F	0.0216	0.0205	0.1003	0.1110	0.0396	0.0190
0.2409	0.0000	TNFRSF1B_E5_F	0.3428	0.2543	0.0462	0.0000	0.0000	0.0141

0.0000	0.0002	TNFRSF1B_P167_F	0.0025	0.0038	0.0458	0.0030	0.0000	0.0005
0.0066	0.0000	TNFSF10_E53_F	0.0096	0.2061	0.0004	0.3884	0.0000	0.0102
0.0000	0.0000	TNFSF10_P2_R	0.0039	0.0000	0.0000	0.9907	0.0020	0.0000
0.4002	0.0072	TNFSF8_E258_R	0.3527	0.0064	0.0274	0.0131	0.0000	0.0037
0.0058	0.0015	TNFSF8_P184_F	0.0043	0.0054	0.0000	0.0051	0.0023	0.0034
0.0163	0.0081	TNK1_P41_R	0.0144	0.2252	0.1062	0.0071	0.1259	0.0166
0.0084	0.6760	TP73_E155_F	0.0156	0.0138	0.0420	0.0095	0.0067	0.0134
0.0056	0.0043	TP73_P496_F	0.0057	0.0050	0.1451	0.0000	0.0033	0.0043
0.0113	0.0219	TP73_P945_F	0.3140	0.0901	0.0846	0.0000	0.0049	0.0075
0.0066	0.0000	TPEF_seq_44_S36_F	0.0067	0.0074	0.0000	0.0089	0.0000	0.0072
0.0130	0.0553	TPEF_seq_44_S88_R	0.0122	0.0126	0.0283	0.0066	0.0045	0.0111
0.9801	0.9910	TRIM29_P261_F	0.9757	0.9732	0.9812	0.7549	0.9919	0.9791
0.1124	0.0067	TRIP6_E33_F	0.0080	0.0706	0.0431	0.0043	0.0048	0.0157
0.0014	0.0000	TSG101_P257_R	0.8511	0.2026	0.3505	0.0038	0.0000	0.0000
0.0412	0.0656	TUBB3_E91_F	0.0037	0.0357	0.2951	0.0000	0.0000	0.3204
0.0000	0.0000	TUBB3_P364_F	0.0000	0.0000	0.0000	0.0000	0.0022	0.0000
0.0000	0.0000	TUBB3_P721_R	0.0000	0.0054	0.0820	0.0000	0.0012	0.0000
0.0054	0.0402	TUSC3_E29_R	0.0060	0.0107	0.0000	0.0032	0.0048	0.3027
0.3390	0.0092	TUSC3_P85_R	0.1586	0.1066	0.0656	0.0056	0.0119	0.4990
0.0000	0.0369	TWIST1_E117_R	0.0000	0.0000	0.0000	0.0000	0.0000	0.0000
0.0027	0.0000	TWIST1_P355_R	0.0273	0.0075	0.1221	0.0316	0.0034	0.0004
0.0000	0.0000	TWIST1_P44_R	0.0000	0.0000	0.0228	0.0000	0.0000	0.0000
0.0043	0.0005	TYRO3_P501_F	0.0016	0.0040	0.0000	0.0000	0.0000	0.0000
0.0000	0.0000	UBA52_P293_R	0.0000	0.0000	0.0000	0.0000	0.0000	0.0000
0.9893	0.9948	UGT1A1_E11_F	0.9853	0.9848	0.9852	0.7295	0.0000	0.9869
0.8835	0.8951	UGT1A1_P564_R	0.9075	0.9132	0.9185	0.9461	0.5691	0.9170
0.9828	0.9949	UGT1A7_P751_R	0.9887	0.9868	0.9836	0.9880	0.9904	0.9862
0.0055	0.0000	UNG_P170_F	0.2903	0.0037	0.0824	0.0065	0.0076	0.0016
0.9782	0.9907	USP29_E274_F	0.9883	0.9889	0.9856	0.9886	0.0000	0.9858
0.9848	0.0000	USP29_P282_R	0.9810	0.9793	0.9750	0.9606	0.0000	0.9740
0.0166	0.0061	VAMP8_P114_F	0.0110	0.0144	0.0280	0.0104	0.0055	0.0115
0.0131	0.0032	VAV1_E9_F	0.0109	0.0091	0.0054	0.0044	0.0000	0.0138
0.0003	0.0000	VAV1_P317_F	0.0000	0.0053	0.0897	0.0039	0.0020	0.0000

0.5273	0.0172	VAV2_E58_F	0.0349	0.0269	0.0171	0.0363	0.2650	0.0270
0.0000	0.0000	VAV2_P1182_F	0.4403	0.3487	0.0000	0.0000	0.0000	0.0045
0.3330	0.0000	VBP1_P12_R	0.4262	0.0000	0.5652	0.6462	0.0214	0.0000
0.0073	0.0105	VEGFB_P658_F	0.0041	0.0074	0.0230	0.0206	0.0041	0.0095
0.0000	0.0000	VIM_P811_R	0.0000	0.0000	0.0000	0.0000	0.0000	0.0000
0.7092	0.0000	WEE1_P924_R	0.9339	0.9867	0.9861	0.9770	0.4940	0.9840
0.0000	0.0000	WNT2_E109_R	0.0000	0.0000	0.0000	0.0000	0.0000	0.0000
0.0360	0.2316	WNT2_P217_F	0.0330	0.0399	0.0359	0.2253	0.0142	0.0376
0.0161	0.0161	WNT2B_P1185_R	0.0157	0.0154	0.0951	0.0110	0.2084	0.0168
0.0132	0.0118	WNT5A_E43_F	0.1613	0.0146	0.0587	0.6934	0.0101	0.0140
0.0041	0.0000	WNT5A_P655_F	0.0047	0.0076	0.0000	0.0064	0.0000	0.0000
0.9413	0.9915	WNT8B_E487_F	0.9649	0.9610	0.9650	0.0065	0.5938	0.9773
0.9750	0.0000	WNT8B_P216_R	0.9744	0.9775	0.9752	0.9886	0.0062	0.9766
0.0257	0.3319	WRN_E57_F	0.5328	0.0406	0.1140	0.3406	0.6820	0.0437
0.9799	0.0437	WRN_P969_F	0.9832	0.9492	0.9827	0.0000	0.0000	0.9727
0.0108	0.0000	WT1_E32_F	0.0073	0.0035	0.0000	0.5427	0.0000	0.0036
0.0058	0.0030	WT1_P853_F	0.0055	0.0042	0.0000	0.0081	0.0018	0.0020
0.9798	0.0000	XRCC1_P681_R	0.9789	0.9684	0.9801	0.0000	0.9911	0.9779
0.9916	0.1184	XRCC2_P1077_F	0.9879	0.9843	0.9855	0.9919	0.9945	0.9852
0.2287	0.1114	YES1_P600_F	0.0151	0.0154	0.0098	0.3193	0.0000	0.0129
0.9902	0.0847	ZIM3_E203_F	0.9860	0.9883	0.9817	0.9907	0.9931	0.9852
0.9870	0.1748	ZIM3_P451_R	0.9888	0.9882	0.9857	0.9886	0.9936	0.9842
0.9817	0.0000	ZIM3_P718_R	0.9826	0.9878	0.9782	0.9940	0.9625	0.9883
0.0000	0.0000	ZMYND10_E77_R	0.0000	0.0021	0.0000	0.0000	0.0000	0.0010
0.0173	0.0042	ZNF215_P71_R	0.0104	0.3858	0.1692	0.0081	0.0000	0.0056
0.0000	0.0000	ZNF264_E48_R	0.0000	0.0000	0.0000	0.0000	0.0000	0.0000
0.9030	0.9924	ZNFN1A1_E102_F	0.8875	0.8967	0.9751	0.6758	0.0000	0.9253
0.9822	0.0000	ZNFN1A1_P179_F	0.9823	0.9824	0.9804	0.9898	0.3349	0.9845
