# Supplementary figures and images for: Epimutation profiling in Beckwith-Wiedemann syndrome: relationship with assisted reproductive technology
Source: Clin Epigenetics. 2013 Dec 10;5(1):23. doi: 10.1186/1868-7083-5-23 (PMC3878854; doi:10.1186/1868-7083-5-23)

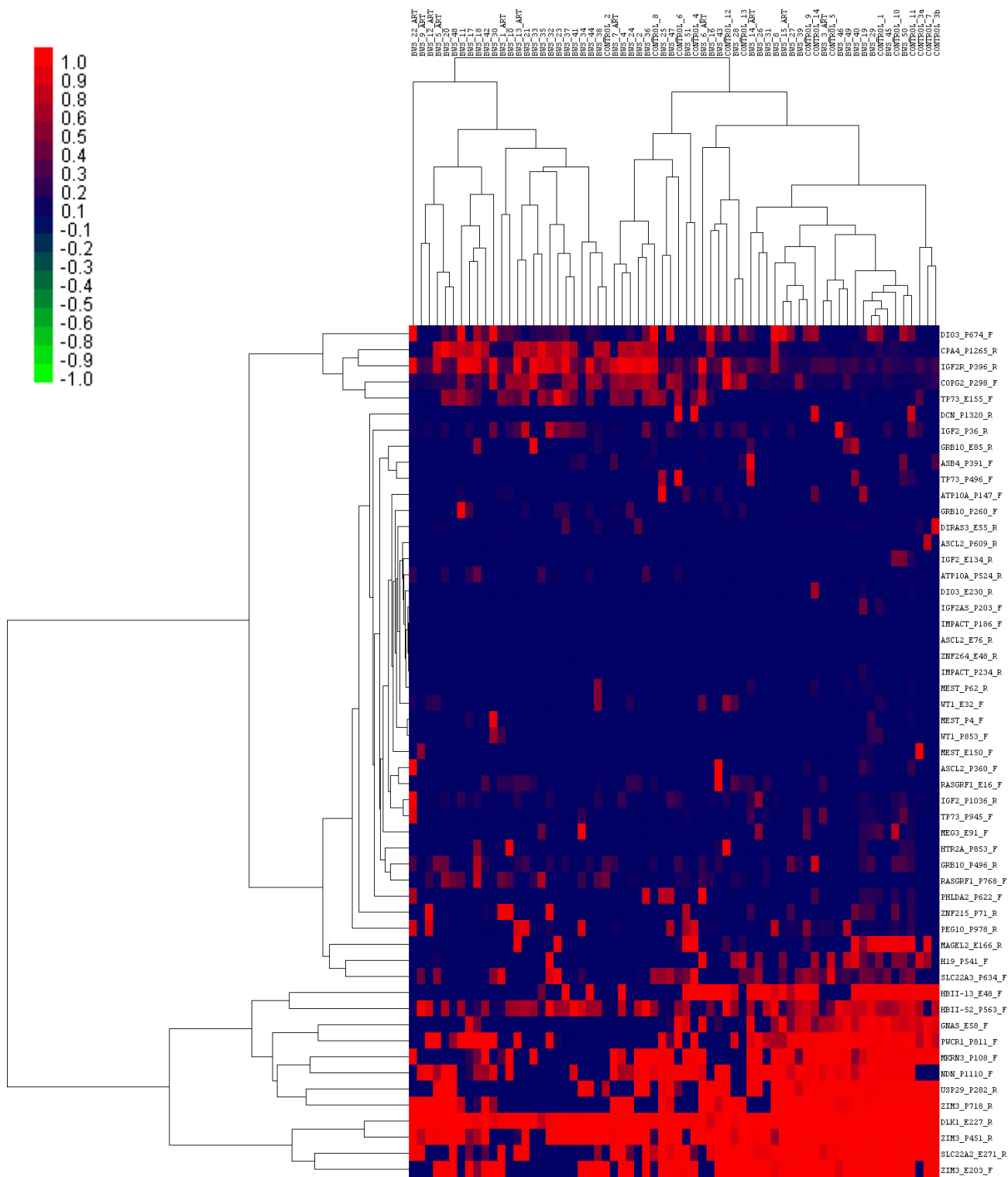

Supplementary figure 1

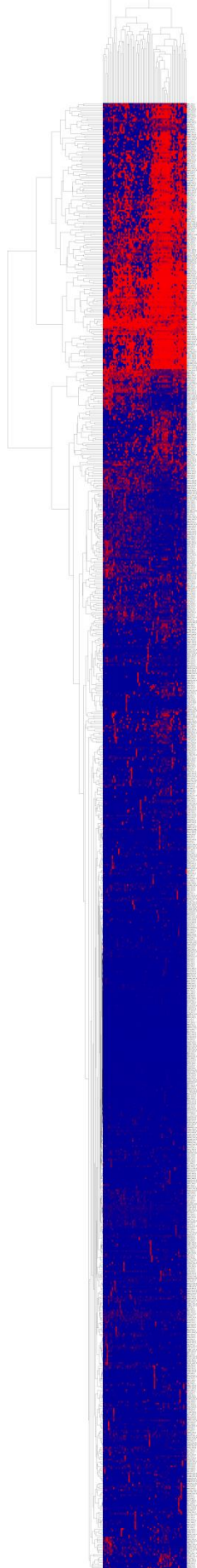

Supplementary figure 2

Supplement: Additional file 2: Figure S1 — Analysis of the imprinted gene CpG methylation data for Beckwith-Wiedemann syndrome (BWS) patients and controls demonstrated two principal clusters (see Results for details). The colours indicate methylation levels, ranging from red (hypermethylated) to blue (hypomethylated). Figure S2. Significant clustering of controls versus BWS seen when methylation at non-imprinted CpG sites was analysed (P = 0.0402) (see Results for details). The colours indicate methylation levels, ranging from red (hypermethylated) to blue (hypomethylated). [file 1868-7083-5-23-S2.pdf]
